# An ultra-low thiourea catalyzed strain-release glycosylation and a multicatalytic diversification strategy

**DOI:** 10.1038/s41467-018-06329-4

**Published:** 2018-10-03

**Authors:** Chunfa Xu, Charles C. J. Loh

**Affiliations:** 1 Fakultät für Chemie und Chemische Biologie, Technische Universität Dortmund,, Otto-Hahn-Straße 4a, 44227 Dortmund, Germany; 2 Abteilung Chemische Biologie, Max Planck Institut für Molekulare Physiologie, Otto-Hahn-Straße 11, 44227 Dortmund, Germany

## Abstract

The utility of thiourea catalysis in selective glycosylation strategies has gained significant momentum lately due to its versatility in hydrogen bonding or anionic recognition activation modes. The use of these non-covalent interactions constitute a powerful means to construct glycosidic linkages as it mimics physiologically occurring glycosyltransferases. However, glycosyl donor activation through the currently employed catalysts is moderate such that, in general, catalyst loadings are rather high in these transformations. In addition, thiourea catalysis has not been well explored for the synthesis of furanosides. Herein, we demonstrate an ultra-low loadings stereoselective and stereospecific thiourea catalyzed strain-release furanosylation and pyranosylation strategy. Our ultra-low organocatalyzed furanosylation enables a multicatalytic strategy, which opens up a unique avenue towards rapid diversification of synthetic glycosides. In-situ NMR monitoring unravel insights into unknown reaction intermediates and initial rate kinetic studies reveal a plausible synergistic hydrogen bonding/Brønsted acid activation mode.

## Abstract (web summary)

Non-covalent glycosyl donor activation often requires high organocatalyst loadings. Here, the authors demonstrate that strain-release glycosylations can take place at very low thiourea catalyst loadings. In addition, the authors developed a one-pot multicatalytic strategy that can diversify glycosides rapidly.

## Introduction

Carbohydrates are among the most prevalent biomolecules that spans a wide spectrum of physiological processes including cellular respiration, cell-cell interaction and adhesion^1,2^, in modulating transcription and complex signal transduction cascades^3^, inflammation and post-translational modifications^4^. Carbohydrates are also pivotal in modulating pathways crucial in cancer research^5,6^. Interestingly, the transfer of nature’s catalytic ingenuity in the utility of non-covalent interactions in enzymes such as glycosyltransferases into the synthesis lab^7^, to construct a plethora of *O*, *N*, *C*, and *S* glycosides has proven to be challenging^8–15^. Very recently, large attention has been given to hydrogen-bonding catalyzed glycosylations especially in thiourea catalysis which provides a miniaturized organocatalyst mimic to glycosyltransferases^16,17^. Thiourea catalysis uniquely leverages mild and highly directional non-covalent hydrogen bonding or anionic recognition interactions to effectively construct mainly *O*- and to a smaller extent *S*-glycosidic linkages on pyranoses^18^.

**Fig. 1:**
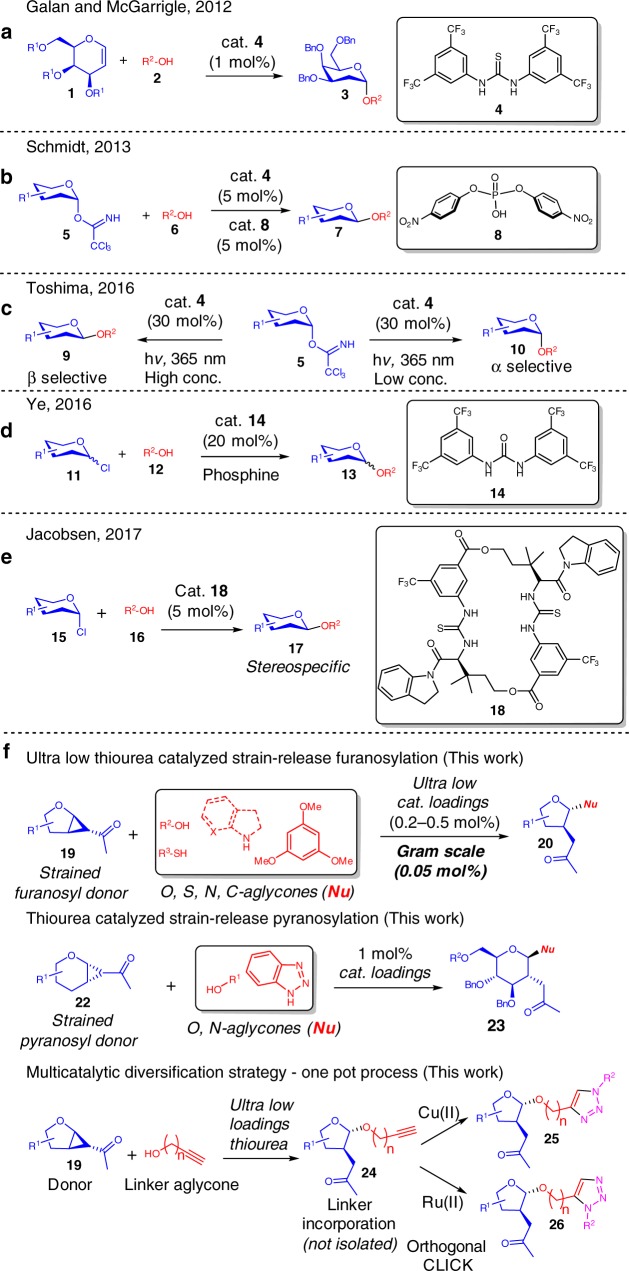
Representative precedents in thiourea catalyzed pyranosylations. **a** Thiourea catalyzed access of 2-deoxyglycosides. **b** Co-catalysis of phosphoric acid and thiourea on trichloroacetimidate donors. **c** Concentration dependent thiourea catalyzed photoinduced catalysis. **d** Urea catalyzed Koenigs–Knorr glycosylation. **e** Macrocyclic thiourea catalyzed stereospecific Koenigs–Knorr glycosylation. **f** Our reported methodology of an ultra-low thiourea catalyzed strain-release glycosylation and a one-pot multicatalytic diversification strategy

The first demonstration of a thiourea catalyzed glycosylation was elegantly reported by Galan and McGarrigle et. al. in 2012, where *D*-Galactal derivatives functionalized by a wide range of protecting groups were converted to 2-deoxyglycosides selectively (Fig. 1a) by the employment of the Schreiner’s thiourea catalyst **4**^19–21^. The interest in this activation mode was later picked up by Schmidt et. al. in 2013 (Fig. 1b), where **4** was effective as a co-catalyst in the presence of a phosphoric acid catalyst **8** in glycosylations utilizing Schmidt’s trichloroacetimidate donors^22^. While the thiourea itself does not catalyse the glycosylation, it is critical in accelerating the reaction, improving the yields and α/β selectivities. This cooperativity effect was utilized by Galan et al. in 2017, where the substrate scope of the glycal addition was expanded towards *D*-Glucal and *L*-Rhamnal derivatives when thiourea **4** was used in conjunction with an axial chiral BINOL based phosphoric acid catalyst^23^. Galan et al., Yoshida and Takao et al., as well as Schmidt et al. contributed to thiourea catalyzed *O* and *S*-glycosylation of 2-nitroglycals^24–26^. In 2016, Toshima et al. reported a photoinduced glycosylation by the utility of Schreiner’s thiourea catalyst **4** (30 mol%) as an organophotoacid on a Schmidt’s trichloroacetimidate donor (Fig. 1c)^27^.

Another significant breakthrough was reported by Ye et al. in 2016, where the first Koenigs–Knorr glycosylation catalyzed by a hydrogen-bonding urea catalyst (20 mol%) was demonstrated (Fig. 1d), and a marked α/β selectivity improvement was observed upon addition of a phosphine on glucose derived donors^28^. Jacobsen disclosed in 2017 a powerful example utilizing a specially designed macrocyclic *bis*-thiourea catalyst **18** (5 mol%) to stereospecifically catalyse the Koenigs–Knorr glycosylation of glycosyl-chlorides (Fig. 1e), and demonstrated effectively the access of *cis*-1,2, *trans*-1,2-, and 2-deoxy-β-glycosidic linkages^29^. Noteworthy is also the recent spike in the utility of chiral phosphoric acid catalysis in stereoselective glycosylations^30–32^.

While great strides in thiourea catalysis are exemplified in the above mentioned reports, considerable challenges still remain in the employment of thiourea/urea catalysis on a broader scale in carbohydrate chemistry. The glycosyl donors employed in thiourea catalysis have remained in the pyranose realm and thiourea catalysis is rarely known to effectively catalyse glycosylations with donors bearing the furanose scaffold^33–36^. In fact, effective furanosylation protocols are scarce in catalysis^34–36^. This is a fundamental synthetic gap since furanosides are highly prevalent in nature and are ubiquitous in the ribose-phosphate backbones of nucleic acids such as DNA, RNA, and signaling molecules such as ATP. Furanosides are also important components in the cell-wall biosynthesis of pathogens, such as *Mycobacterium tuberculosis*^37–40^. In addition, both (*D*)- and (*L*)- furanonucleoside motifs have found utility in anti-viral therapeutics^41^. Hence, effective furanosylation at ultra-low catalyst loadings will be a significant step towards accessing furanoside derivatives efficiently. Moreover, another unsolved challenge lies in the plausibility of thioureas to catalyze *N-* and *C*-glycosylations, an important transformation to access nucleoside analogs.

Herein, we describe a highly selective and stereospecific ultra-low thiourea catalyzed strain-release furanosylation that enables *O*, *N*, *S*, and *C*-glycosylations on a unique class of strained derivatized furanosyl donors (Fig. 1f)^42–46^. A low thiourea catalyzed strain-release *O* and *N*-pyranosylation is also demonstrated. Significantly, by incorporating multicatalysis and alkyne-azide cycloaddition (AAC) or CLICK chemistry into our furanosylation protocol^47–49^, a sugar diversification concept is developed. This opens up a general path to access synthetic glycosides bearing varying donor structure, linker length, biological warheads, and molecular shapes with potential biological utility. Deeper NMR investigations of the reaction profile and initial rate kinetics studies also shed light into the mechanism of the strain-release glycosylation.

## Results

### Optimization of strain-release glycosylation

This investigation was initiated by utilizing a (*D*)-furanoxylose, (*D*)-Xyl*f* derived strained glycosyl donor **19a** and a (*D*)-Galactose derived acceptor **21a** as the model furanosylation reaction (Table 1)^50–52^. We hypothesize that utilizing such cyclopropane fused furanosides might provide an advantage in activating the furanosyl donor via thermodynamic strain release, and the ketone functionality provides a functional handle for thiourea to activate via bidendate hydrogen bonding. Initial screening of various thiourea, urea, and squaramide catalysts (Entry 1–5, Table 1) revealed the superiority of the Kass catalyst **A**^53^. This recently reported charge enhanced thiourea not yet employed in glycosylations gave 77% yields and a very high α/β ratio at 5% catalyst loadings at RT. The effect of different solvents was probed (see Supplementary Table 1) and the solvent screen revealed that fluorobenzene is optimal.

**Table 1:** Representative reaction optimization and screening

For full optimization, see Supplementary Table 1

**19a** (0.1 mmol, 1 equiv), **21a **(0.2 mmol, 2 equiv), and catalyst in solvent (0.6 mL), temperature, 6–16 h

^a*^The yield and α/β ratio was determined by crude ^1^H NMR using CH_2_Br_2_ as an internal standard

^b$^Isomer was not observed

BAr^F^_4_^*−*^: tetrakis[3,5-*bis*(trifluoromethyl) phenyl]borate, PhF: fluorobenzene, Me: Methyl, RT: Room temperature

With promising results in hand, we further studied the temperature effect (see Supplementary Table 1), reducing the temperature to 0 °C almost completely stopped the reaction, however, an increase in temperature to 50–70 °C gave almost quantitative yields with very high α/β ratios. To our delight, reduction to 1 mol% of the charged enhanced thiourea **A** gave quantitative yields preserving the α/β ratios. The optimal conditions were achieved when we reduced the catalyst loadings to 0.2 mol%. A further control experiment at the exact reaction conditions in the absence of catalyst **A** showed negligible conversion.

To deepen our understanding of the nature of catalysis by **A**, since pyridinium salts are known to also catalyse alcohol addition to glycals^54^, catalyst **F**, the charged *bis*-fragment of catalyst **A**, was synthesized, and subjected (0.4 mol%, Entry 8, Table 1) to the reaction mixture, which yielded negligible product. This control experiment revealed the criticality of the thiourea component in this methodology. We also tried the Schreiner thiourea **D** in similar conditions (Entry 9, Table 1) as our conditions in catalyst **A**. A slightly lower yield of 86% is obtained with slightly elevated α/β ratios of 97:3, which provided a good alternative protocol in our subsequent investigation to accommodate a wider variety of substrates.

### Substrate scope of strain-release furanosylation

With an optimized furanosylation protocol in hand, we further proceeded to expand the substrate scope of this ultra-low loadings thiourea catalyzed reaction.

Our investigation revealed that the *O*-glycosylation proceeded with good to excellent anomeric selectivity. This protocol tolerates glycosyl donors such as (*D,L*)-furanoxylose, (*D*)-Xyl*f* derived **19a**, (*L*)-Xyl*f* derived ***ent*-19a** and (*D*)-furanoarabinose, (*D*)-Ara*f* derived **19b**. We discovered in our investigation that the anomeric selectivity was highly stereospecific^29^. When **19a** or ***ent*-19a** were employed, anomeric selectivity was consistently biased towards the α-anomer. When **19b** was utilized, the major anomer obtained was the β-anomer.

The anomeric preference observed pointed strongly towards a mechanism that proceed via anchimeric assistance from the C2- ketone moiety. Moreover, we were delighted with the broad range of *O*-acceptors that were tolerated which include various protected monosaccharides containing unprotected hydroxyl groups in varying positions (Table 2). Even monosaccharides bearing two unprotected alcohols showed excellent regioselectivity on the less sterically hindered primary alcohol with complete α-selectivity to form **20d**. We also demonstrated a rare furanosylation on an anomeric C1-hydroxyl acceptor which proceeded with diminished yields to give **20e**. Other biologically relevant acceptors such as protected amino-acid derivatives of (*L*)-Serine and (*L*)-Threonine showed excellent reactivity to generate **20** **l** and **20** **m**, which would potentially open up interesting avenues in glycopeptide research. Lipids such as cholesterol and testosterone bearing hydroxyls in different positions also proceeded smoothly, generating glycolipid type derivatives **20p-20s** that has applications in glycolipids as well as cardiac glycoside research^55^.

**Table 2:** *O*-Furanosylation substrate scope using sub-molar catalyst loadings

Reaction conditions: Furanosyl donor **19** (0.2 mmol, 1 equiv.), *O*-acceptor **21** (0.4 mmol, 2 equiv.), and 0.2 mol% catalyst **A**, in PhF (1.2 mL), 50 °C, 16 h; α:β ratio determined by ^1^H NMR on the crude reaction mixture. Isolated yields are indicated below each structure unless otherwise stated. ^*^Microwave, 4 h, 1 mol% catalyst **D**. ^+^Furanosyl donor **19** (0.3 mmol, 1.5 equiv.), *O*-acceptor **21 (**0.2 mmol, 1 equiv.), 0.5 mol% catalyst **A**. ^§^ 0.5 mol% catalyst **A**

Significantly, we extended our *O*-glycosylation methodology to include a multitude of alcohols with important functional handles. The tolerable range of these alcohols spans from sterically less hindered primary alcohols, including allyl alcohols, propargyl alcohols, and azido containing alcohols, which opens up numerous opportunities to incorporate CLICK chemistry for our proposed multicatalytic diversification^49^, and allows facile tethering of various structural motifs via multicatalysis. Interestingly, sterically more hindered secondary alcohol acceptors such as *L*-menthol, 2-adamantanol as well as highly hindered tertiary alcohols like 1-adamantanol proceeded smoothly in this protocol, generating *O*-furanosides **20w-20y** with good to excellent anomeric selectivities. Utility of *p*-bromo-benzylalcohol as an *O*-acceptor allows access of **20n**, which facilities further transformation using Pd-catalysis on the C-Br functionality. Hydroxylamine, a rarely utilized acceptor can also be used in this protocol to generate **20o**^56^. There is an exceptional case where bicyclic **20k** is obtained instead of a direct attack on the anomeric carbon, when donor **19b** is used. It is postulated that the unusual regioselectivity observed might be due to a dual steric clash effect of a congested anomeric carbon due to the anchimeric effect of the C2 ketone and a bulky acceptor, which then allowed preferential attack on the less congested distal ketone, (See Supplementary Figure 3 and Supplementary Discussion 1).

Furthermore, we probed the limits of our methodology by investigating *S*-furanosylation and *N*-furanosylation (Table 3), two important yet understudied aspects in catalytic glycosylations. The *N*-glycosylation study revealed that our protocol works excellently with bicyclic heterocyclic *N*-acceptors, generating the majority of *N*-glycosides **20aa-ah** with complete anomeric selectivity except for **20ag** (Table 3). The *N*-acceptors tolerable in this protocol include derivatized purines and pyrimidines, as well as other structurally similar mimetics of purines, such as benzotriazoles and indolines. We also demonstrated that *S*-furanosylation works well to generate protected cysteine derivatives **20ak** and thiophenol analogs **20ai**, **20aj**, and **20al**, providing excellent anomeric selectivity on (*L*,*D*)-Xyl*f* and (*D*)-Ara*f* derived donors.

**Table 3:** *N*, *S*, *C* -Furanosylation substrate scope

Reaction conditions: **19** (0.2 mmol, 1 equiv.), **21** (0.24 mmol, 1.2 equiv.), and 0.5 mol% catalyst **A**, in PhF (1.2 mL), 50 °C, 16 h; α/β ratio determined by ^1^H NMR on the crude reaction mixture. ^*^0.2 mol% catalyst **A**. ^+^TMS protected substrate (0.4 mmol) was used. ^§^Acceptor **21** (0.4 mmol, 2 equiv.). ^#^RT. ^†^**21** (0.2 mmol, 1 equiv.), **19** (0.3 mmol, 1.5 equiv.), LiClO_4_ (1 equiv.), 0.5 mol% catalyst **A**, 80 °C, 16 h

Significantly, expanding our concept into *S*-furanosylation opens up potential opportunities for cysteine glycol-tagging in proteins, especially with recent discovery of *S*-glycosylation also as a vital post-translational modification^57,58^. The isolation of a by-product **20al-side** further augments the intermediacy of C2-anchimeric assistance in the reaction mechanism. While the search for a suitable acceptor in the *C*-furanosylation was extremely challenging, we managed to obtain a rare case of a Friedel–Crafts-type *C*-furanoside by subjecting 1,3,5-trimethoxylbenzene to our reaction conditions, which generates the corresponding *C*-glycosides **20am-ao** with good yields and selectivity. Other less reactive *C*-nucleophiles, such as mesitylene, 1,3-dimethylbenzene, 3-cyanocoumarin, and anthracene gave no observable product (See Supplementary Note 4).

### Substrate scope of strain-release pyranosylation

In addition, we were curious to understand the wider applicability of our thiourea catalyzed protocol in strain-release pyranosylations (Table 4). Initial optimizations on our strained pyranosyl donor revealed subtleties in reaction conditions distinct from furanosylation which we needed to take into account (see Supplementary Table 2).

**Table 4:** *O*, *N*-Pyranosylation substrate scope

Reaction conditions: **22** (0.1 mmol, 1 equiv.), **21** (0.2 mmol, 2 equiv), catalyst **A** (1 mol%) in PhF (1 mL), Ar, RT, 12 h; α:β ratio determined by ^1^H NMR on the crude reaction mixture. ^*^Catalyst **A** (5 mol%), 4 Å MS (43 mg), PhF (1 mL), Ar, 70 °C, 12 h

While the pyranosylation reaction was still rather facile which proceeded well at room temperature (RT), we needed to increase the catalyst loadings to 1% possibly due to the increased donor stability as a result of ring strain relief in six-membered ring containing substrates **22a-c**. A range of pyransoyl donors **22a-22c** with varying stereogenic information and protecting groups were well tolerated (Table 4). Moreover, different *O* and *N*-acceptors were found to be suitable for this methodology, generally providing the β-anomer of **23** stereoselectively and stereospecifically.

When di-*O*-isopropylidene-α-D-glucofuranose was used as the glycosyl acceptor, bicyclic product **23e** (Table 4) was generated analogous to the formation of **20k** from arabinose derived furanoside donor **19b** (Table 2). We postulate that the *trans* relative stereochemistry between C1 and C5 of the pyranoside and furanoside donors **22a-c** and **19b** seems to be the key contributory factor to the observed distal regioselectivity to ease steric congestion, which is rendered more pronounced by such bulky acceptors (See Supplementary Figure 3 and Supplementary Discussion 1).

### Mechanistic studies and control experiments

**Fig. 2:**
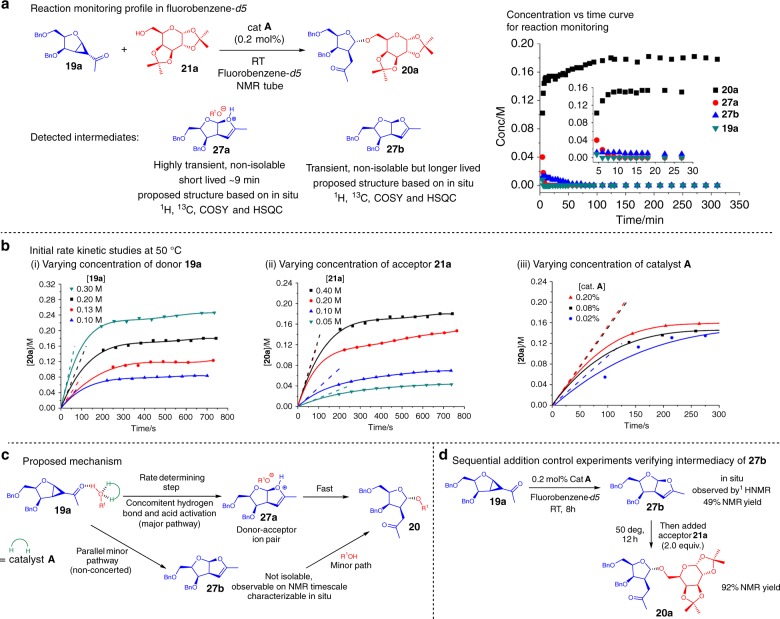
Mechanistic and kinetic studies. **a** In-situ NMR monitoring of strain-release furanosylation of **19a** at RT. **b** Initial rate kinetic studies of strain-release furanosylation. **c** Proposed mechanistic model of the strain-release furanosylation. **d** Sequential addition control experiments to verify **27b**

In order to gain a deeper understanding of the mechanistic intricacies of the thiourea catalyzed strain-release furanosylation, NMR monitoring of the reaction progress and preliminary initial rate kinetic studies were conducted. In-situ NMR monitoring of the reaction revealed an unexpected finding (Fig. 2a and see Supplementary Figure 6).

Two different intermediates **27a** and **27b** detected during the RT in-situ studies appear to be responsible for the strain-release furanosylation. As **27a** and **27b** are both non-isolable intermediates, the postulated structures drawn (Fig. 2a) are based on in-situ ^1^H, ^13^C, COSY, HSQC elucidation from the reaction mixture (see Supplementary Figure 12–15 and Supplementary Note 4).

In the reaction monitoring profile at RT (Fig. 2a and see Supplementary Figure 6), there was almost rapid disappearance of donor **19a** in less than 5 min into the reaction, which also corresponded to the rapid formation of product **20a**. Concomitantly, we noticed the depletion of the short lived transient intermediate **27a** in the initial rapid phase till ~9 min into the reaction. **27a** seems to be responsible for the steep increase in **20a** formation till almost 75% conversion at 9 min, where intermediate **27a** completely disappeared.

After the initial rapid phase, there is a more gradual formation of **20a** and depletion of a second non-isolable intermediate **27b** at a similar gradual rate (Fig. 2a and see Supplementary Figure 6).

The bulk of the obtained product **20a** (till 83% conversion) is postulated to arise from intermediate **27a** and **27b**. It must also be mentioned that trace amounts of transient unidentifiable intermediates bearing doublets in the 5.30–5.85 ppm range were detected (See Supplementary Figure 8, 9), but their identities could not be determined from the crude NMR mixture.

Moreover, to better understand the effect of the strained glycosyl acceptor, the glycosyl donor, as well as the catalyst on the rate determining step, initial rate kinetics were performed by varying the concentration of each of the above mentioned reagents and monitoring the reaction using ^1^H NMR spectroscopy (Fig. 2b and see Supplementary Figure 10).

Our preliminary kinetic studies revealed that modifying the concentrations in each of these three reagents had a corresponding initial rate effect, with the initial rate (slope at *t* = 0) increasing with increasing reagent concentration. Further deriving the slope of the ln rate vs the ln concentration plots (See Supplementary Figure 11) revealed a first order kinetics dependence for acceptor **19a**, as well as donor **21a**. Interestingly, we arrived at a fractional order kinetics dependence for catalyst **A**. These early studies are also indicative of a termolecular mechanism in the rate determining step, which suggest a close interplay between hydrogen bonding and Brønsted acid mechanisms in the catalytic cycle.

We propose according to these results a concomitant hydrogen bond activation of the alcohol oxygen on the acceptor by the thiourea catalyst, which then weakens the O-H bond, resulting in a subsequent relay of the acidic alcohol proton towards carbonyl activation of **19a** in the rate determining step (Fig. 2c)^19^. The interplay between hydrogen bonds and proton shuttling is also indicative of the biomimetic nature of the thiourea mechanism similar to glycosyltransferases, whereby acidic amino acid side chains and hydrogen bonding in the active enzyme pocket play pivotal roles in glycosylation^18^.

Additionally, a series of control experiments (See Supplementary Table 3 and Supplementary Discussion 2) using various bidentate hydrogen-bonding catalysts on the strain-release furanosylation such as thioureas and squaramides were conducted to further understand the cruciality of the bidentate hydrogen bond alignment in the methodology, since hydrogen bonds are directional. We also attempted thiourea derivatives with hydrogen bond alignments distorted from the conventional bidentate mode to access monodentate hydrogen-bonding catalysts, by either protecting one thiourea N-H with a methyl group or by utilizing 2-thiouracil, which constraints the two thiourea N-H in a cyclic system, hence precluding a bidentate hydrogen bond activation. These studies demonstrated that both bidentate and monodentate hydrogen-bonding thiourea catalysts can catalyze the strain-release furanosylation, although the bidentate congeners provided superior yields, further augmenting the participation of hydrogen-bonding activation in the mechanism.

Non-hydrogen-bonding-based Brønsted acids with slightly higher and lower p*K*_a_ compared to the Schreiner’s thiourea (p*K*_a_ = 8.4 in DMSO), such as triethylammonium chloride (p*K*_a_ = 9.0 in DMSO) and meldrum acid (p*K*_a_ = 7.3 in DMSO) were also conducted as orthogonal experiments to probe acidic effects^19^. In both cases, the reaction yields were inferior to the Schreiner’s thiourea, which provides another evidence that while Brønsted acidity might play a role in the mechanism, solely drawing upon Brønsted acidic explanations might be insufficient to describe the intricate synergism between hydrogen bonding and Brønsted acid mechanism we propose in this methodology^59^.

An additional control experiment was also conducted by simply first adding catalyst **A** to **19a** without addition of acceptor **21a** in an NMR tube, we noticed that after 8 h at RT, **27b** was formed with 49% NMR yield (Fig. 2d). No **27a** could be detected without addition of acceptor **21a**, further proving the cruciality of the acceptor for the major pathway to occur (Fig. 2c). The sequential addition of acceptor **21a** subsequently gave **20a** in 92% NMR yield, confirming that while product formation in our protocol through **27b** via a step-wise reaction is the minor route, it is also viable en route to **20a**.

### One-pot multicatalytic diversification

With a facile and effective furanosylation protocol in hand, our protocol can be extended into a tractable multicatalysis concept for diversification (Table 5)^60^, which allows rapid generation of diversified synthetic carbohydrates with varying sugar donors, acceptors, and linker length.

**Table 5:** Scope of one-pot multicatalytic diversification

Reaction conditions: **19** (0.2 mmol, 1 equiv.), **21** (0.4 mmol, 2 equiv.), and 0.2 mol% catalyst **A** in PhF (1.2 mL), 50 °C, 16 h, then solvent removal, azide or alkyne (2 equiv.), Cp*RuCl(PPh_3_)_2_ (5 mol%) in PhCH_3_ (2 mL), 80 °C, 4 h or CuSO_4_•5H_2_O/sodium ascorbate (10 mol%), CH_2_Cl_2_/H_2_O (1:1 v/v, 2 mL), RT, 6 h; α/β ratio determined by ^1^H NMR on the crude reaction mixture

We sought to introduce stereochemical and morphological diversity in our synthesized glycosides by tapping upon various carbohydrate donors present in the chiral pool, such as (*L*,*D*)-Xyl*f* and (*D*)-Ara*f* derived donors. By coupling the strained donor unit **19** with an *O*-acceptor bearing alkyne or azide alcohols **21** with varying carbon chain (*n* = 0, 1, 2), a pool of *O*-glycosides functionalized with a CLICK handle (alkyne or azide) with multiple linker length can be generated (Table 5). Without isolating the *O*-glycoside in the linker pool, our developed furanosylation protocol is compatible with a one-pot second catalytic step where two diversification parameters can be permutated through the sequential CLICK reaction. Firstly, acceptors bearing an azido or alkyne functional group provides a direct mean to incorporate various moieties of biological interest which include either carbohydrates or biologically active motifs such as isatins and coumarins. Secondly, by orthogonally selecting a Cu(II) catalyzed CLICK or a Ru(II) catalyzed CLICK, differential regioselectivities was exploited to generate either a linear-shaped disaccharide or a bent disaccharide (Table 5)^61,62^, paving the way to access oligosaccharides with different molecular morphologies. A representative scope **25–26** of accessible disaccharides by diastereomeric (sugar donor) or regioisomeric (CLICK) permutations via this multicatalytic strategy is demonstrated in Table 5. An analogous strategy was also demonstrated by azido containing acceptors, which allowed us to access analogs containing a reversed triazole and introducing biological warheads, such as isatin **25d** or coumarin **25e**. Additionally, CLICK functionalized triazole derivatives of carbohydrates are also known to be potent inhibitors of α-glucosidases, further augmenting the biological potential of the multicatalytically generated compounds^63^.

### Gram scale synthesis and further derivatizations

**Fig. 3:**
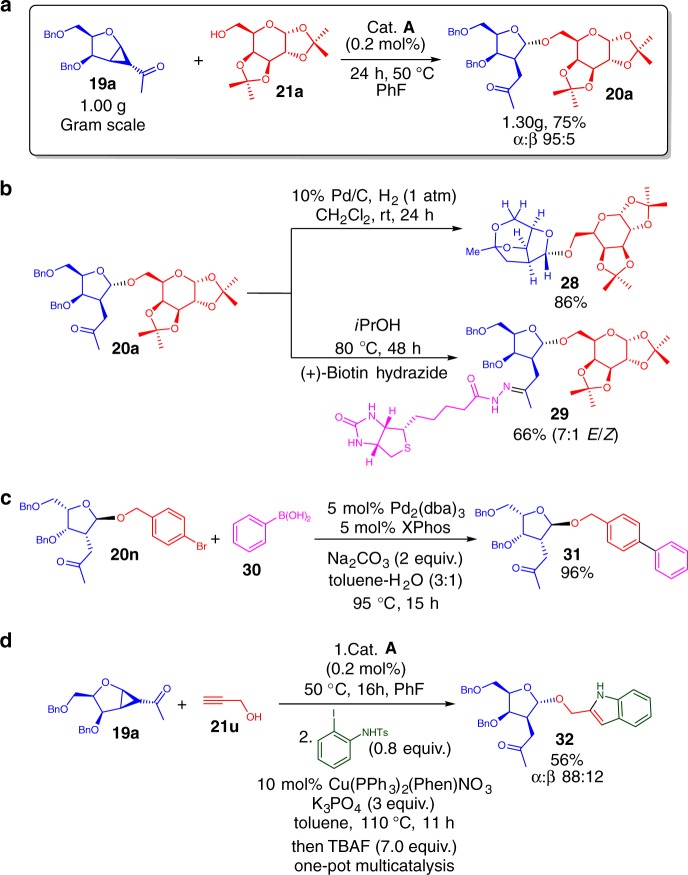
Further derivatizations. **a** Gram scale reaction. **b** Hemiketalization/ketalization cascade and biotinylated hydrazone formation. **c** Suzuki coupling to access **31**. **d** One-pot multicatalytic strain-release glycosylation/[3 + 2]-cycloaddition sequence

To demonstrate the scalability of our protocol, we performed a gram scale strain-release furanosylation at a reduced loading of 0.05 mol% (Fig. 3a). To our delight, the upscaled reaction proceeded smoothly to yield 75% of **20a** with slight increase in anomeric selectivity (α:β 95:5). A further attempted hydrogenolysis of the benzyl protecting groups gave a surprising result, the deprotected hydroxyl groups underwent a hemiketalization/ketalization cascade (Fig. 3b and see Supplementary Figure 5) to form an architecturally complex caged ketal **28** with 86% yield. The availability of the functional C2-ketone was advantageous for the attachment of a biotin label via hydrazone formation to yield 66% of **29** (Fig. 3b), a known useful strategy that has been employed in chemoenzymatic labeling approaches in pyranose based probes^64,65^. By exploiting the C-Br bond in glycoside derivatives such as **20n**, we are able to employ facile Pd-catalyzed Suzuki coupling to form **31** quantitatively (Fig. 3c). Furthermore, a facile one-pot multicatalytic synthesis was achieved by combining our strain-release glycosylation with a Cu-catalyzed non-CLICK [3 + 2]-cycloaddition of alkynes with *o*-iodotosylanilines to generate a C2- indole glycoside **32** (Fig. 3d) which opens up a route towards related indolyl-glycosides with anti-cancer activities^66,67^.

## Discussion

We report a highly efficient extremely low loading thiourea catalyzed stereoselective strain-release furanosylation strategy, which accommodates a wide variety of *O*, *N*, *S*, and *C*-acceptors. We also demonstrated that this protocol is also extendable towards *O* and *N*-strain-release pyranosylations. In-situ NMR monitoring and in-situ characterization of non-isolable transient intermediates also provided deeper mechanistic insights into the intricacies of hydrogen bonding and proton shuttling of thiourea catalyzed strain-release glycosylations. Preliminary initial rate kinetics study provided insightful understanding of the possible termolecular nature of the strain-release furanosylation in the rate determining step. A one-pot multicatalytic diversification concept is also introduced, which merges thiourea catalysis and CLICK chemistry to expedite the access of diverse synthetic furanosides with varying linker length, molecular shape, and biological warheads. We envision that our unique demonstration of a multicatalytic diversity concept paves the way as a general diversification strategy in other catalytic glycosylation protocols. Further investigation into the biological activities of these compounds are currently underway.

## Methods

### General techniques

Unless otherwise stated, all reactions were set up under inert atmosphere (argon) utilizing glassware that were oven dried and cooled under argon purging. Silica Gel Flash Column Chromatography was performed on deactivated Silica gel Merck 60 (particle size 40–63 μm) (Triethylamine (5% v/v) was used as the deactivating reagent). Starting materials were purchased directly from commercial suppliers (Sigma Aldrich, Acros, Alfa Aesar, VWR) and used without further purifications unless otherwise stated. All solvents were dried according to standard procedures or brought from commercial suppliers. Reaction solvent (Fluorobenzene) was stored over activated 3Ǻ molecule sieves. Reactions were monitored using thin-layer chromatography (TLC) on Merck silica gel aluminum plates with F254 indicator. Visualization of the developed plates was performed under UV light (254 nm) or KMnO4 stain or H_2_SO_4_-EtOH (10% H_2_SO_4_ v/v). Dry loading was performed on Silica gel 9 due to observed product decomposition on normal silica gel.

NMR characterization data (^1^H NMR, ^13^C NMR and 2D spectra) were collected at 300 K on a Bruker DRX400 (400 MHz), Bruker DRX500 (500 MHz), INOVA500 (500 MHz), and Bruker DRX700 (700 MHz) using acetone-d_6_, CD_2_Cl_2_ or CDCl_3_ as solvent. Data for ^1^H NMR are reported as follows: chemical shift (*δ* ppm), multiplicity (*s* = singlet, *d* = doublet, *t* = triplet, *q* = quartet, *m* = multiplet, br = broad), coupling constant (Hz), integration with the solvent resonance as internal standard (acetone-d_6_: *δ* = 2.05 ppm for ^1^H, *δ* = 29.92 ppm for ^13^C; CD_2_Cl_2_: *δ* = 5.32 ppm for ^1^H, *δ* = 54.00 ppm for ^13^C; CDCl_3_: *δ* = 7.26 ppm for ^1^H, *δ* = 77.16 ppm for ^13^C).

High resolution mass spectra were recorded on a LTQ Orbitrap mass spectrometer coupled to an Accela HPLC-System (HPLC column: Hypersyl GOLD, 50 mm × 1 mm, particle size 1.9 μm, ionization method: electron spray ionization). Optical rotations were measured in a Schmidt + Haensch Polartronic HH8 polarimeter equipped with a sodium lamp source (589 nm), and are reported as follows: [α]_D_^T °C^ (*c* *=* g/100 mL, solvent). Melting point ranges were taken from solids which were obtained from the solvents as indicated. They were determined on a BÜCHI Melting Point B-540 Apparatus. The microwave reaction was conducted on the Discover SP-Microwave Synthesizer and 10 mL tube with a proper cap was used.

The ratio of anomers was determined by ^1^H-NMR and HSQC analysis of the crude reaction mixture via integration of characteristic signals of the anomeric proton in the ^1^H NMR spectra. Chemical yields refer to isolated substances after flash column chromatography, combined yield of both anomers reported. NMR yields were determined using dibromomethane or 1,3,5-trimethoxybenzene as internal standard.

### General procedure for thiourea catalyzed furanosylation

An oven dried tube with a stirrer bar was charged with strained cyclopropanated furanoside **19a**, ***ent*-19a**, or **19b** (0.2 mmol, 1.0 equiv.), and glycosyl acceptor **21** (0.4 mmol, 2.0 equiv.). Then the tube was purged with argon and sealed with a rubber stopper. After that, anhydrous fluorobenzene (1.1 mL) and a solution of catalyst **A** (100 µL, 4 mM, 0.002 equiv., freshly prepared) was added. The tube was further sealed with parafilm and immersed in a preheated 50 °C oil bath for 16 h. Upon completion of the reaction, the reaction mixture was subsequently dry loaded onto silica 9 and subjected to flash column chromatography with deactivated silica gel for purification to yield **20**.

### General procedure for thiourea catalyzed pyranosylation

An oven dried tube with a stirrer bar was charged with strained cyclopropane pyranoside **22** (0.1 mmol, 1 equiv.), glycosyl acceptor **21** (0.2 mmol, 2 equiv.), catalyst **A** (1 mol%). Then the tube was purged with argon. Subsequently, anhydrous fluorobenzene (1 mL) was added to the tube under argon, subsequently the tube was sealed with parafilm and stirred at RT for 12 h. Upon completion of the reaction, the reaction mixture was subsequently dry loaded onto silica 9 and subjected to flash column chromatography with deactivated silica gel for purification.

### General procedure for multicatalytic diversification

An oven dried tube with a stirrer bar was charged with strained cyclopropanated furanoside **19a**, **ent-19a**, **19b** (70.5 mg, 0.2 mmol, 1.0 equiv.), and glycosyl acceptor (0.4 mmol, 2.0 equiv.). Then the tube was purged with argon and sealed with a rubber stopper. After that, anhydrous fluorobenzene (1.1 mL) and a solution of catalyst A (100 μL, 4 mM) was added. The tube was further sealed with parafilm and immersed in a preheated 50 °C oil bath for 16 h. Upon completion of the reaction, the solvent was removed under reduced pressure to give a residue. Then Cp*RuCl(PPh_3_)_2_ (5 mol%) was added to the residue. The tube was purged with argon and added with azide (0.4 mmol, 2.0 equiv.) toluene (2.0 mL) solution, and sealed and heated at 80 °C for 4 h. The mixture was absorbed onto silica 9 and purified by silica gel column chromatography (dry loading).

## Electronic supplementary material

Supplementary Information

## Data Availability

The authors declare that the data supporting the findings of this study are available within the article and its Supplementary Information Files. Additional data are available from the corresponding author upon reasonable request.
