# Supplementary material for: An ultra-low thiourea catalyzed strain-release glycosylation and a multicatalytic diversification strategy
Source: Nat Commun. 2018 Oct 3;9:4057. doi: 10.1038/s41467-018-06329-4 (PMC6170412; doi:10.1038/s41467-018-06329-4)
Supplement: Supplementary file 1 — Supplementary Information [file 41467_2018_6329_MOESM1_ESM.pdf]

Supplementary Information

An Ultra-Low Thiourea Catalyzed Strain-Release Glycosylation and a Multicatalytic Diversification Strategy

Xu & Loh

Supplementary Tables:

Supplementary Table 1: Optimization for Furanosylation

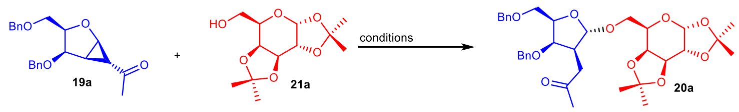

Entry	catalyst	solvent	temp/°C	time/h	yield% (α:β)[b]
1	5 mol% **A**	PhF	RT	6	77% (93:7)
2	5 mol% **B**	PhF	RT	6	< 5%
3	5 mol% **C**	PhF	RT	6	< 5%
4	5 mol% **D**	PhF	RT	6	5%[c]
5	5 mol% **E**	PhF	RT	6	< 5%
6	5 mol% **A**	CH_2Cl_2	RT	6	63% (91:9)
7	5 mol% **A**	CH_3CN	RT	6	16% (62:38)
8	5 mol% **A**	Acetone	RT	6	42% (84:16)
9	5 mol% **A**	THF	RT	6	43% (83:17)
10	5 mol% **A**	Dioxane	RT	6	55% (83:17)
11	5 mol% **A**	Et_2O	RT	6	75% (92:8)
12	5 mol% **A**	Toluene	RT	6	66% (89:11)
13	5 mol% **A**	PhF	0	6	< 5%
14	5 mol% **A**	PhF	50	6	99% (93:7)
15	5 mol% **A**	PhF	70	6	97% (94:6)
16	1 mol% **A**	PhF	50	6	>95% (93:7)
17	0.2 mol% **A**	PhF	50	16	>95% (93:7)
18	none	PhF	50	16	< 5%
19	0.4 mol% **F**	PhF	50	16	< 5%
20	0.2 mol% **D**	PhF	50	16	86% (97:3)
21[c]	0.2 mol% **A**	PhF	50	16	< 5%

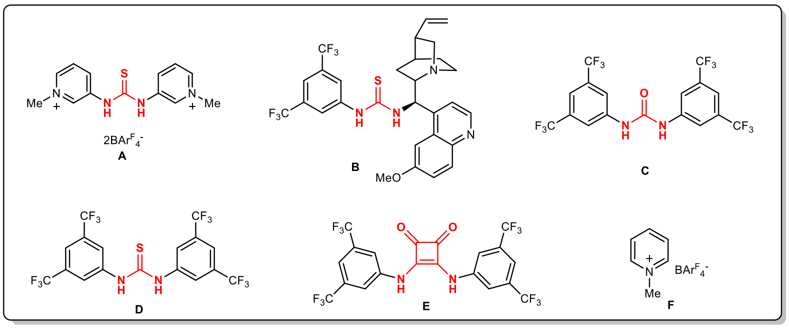

[a]Reaction conditions: Furanosyl donor **19a** (0.1 mmol. 1 equiv.), acceptor **21a** (0.2 mmol, 2 equiv.), catalyst in PhF (0.6 mL), temperature, 6-16 h, Ar; [b]α/β ratio determined by ^1H NMR and HSQC on the crude reaction mixture with CH_2Br_2 as an internal standard. [c]K_2CO_3 (2.0 equiv.) was added.

Supplementary Table 2: Optimization for Pyranosylation

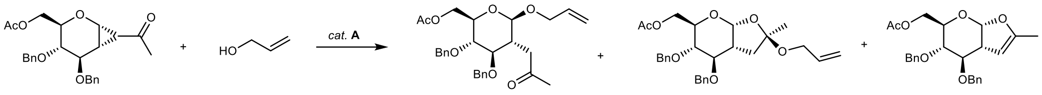

Entry	Cat. **A** loading	Solvent	Temp. (°C)	Time (h)	Results		
					**P1**	**P2**	**P3**
1	1%	$PhCH_3$	70	2	60%, β/α > 20:1	24%	12%
2	1%	$PhCH_3$	60	2	65%, β/α > 20:1	21%	11%
3	1%	$PhCH_3$	80	2	36%, β/α > 20:1	31%	35%
4	1%	$PhCH_3$	50	2	48%, β/α > 20:1	6%	36%
5	1%	PhF	50	2	70%, β/α > 20:1	15%	15%
6[b]	1%	PhF	RT	2	40%, β/α > 20:1	<5%	<5%
7[c]	1%	PhF	RT	6	84%, β/α > 20:1	<5%	<5%
8[c]	0.5%	PhF	RT	6	12%, β/α > 20:1	<5%	<5%
9 [c]	1%	PhF	RT	12	>95% (79%[c]) β/α > 20:1	<5%	<5%
10	----	PhF	RT	12	0	0	0

[a]Reaction conditions: pyranosyl donor **22a** (0.05 mmol. 1 equiv.), allyl alcohol (0.1 mmol, 2 equiv.), catalyst in PhF (1 mL), temperature, 2-12 h, Ar; α/β ratio and yield was determined by ^1H NMR on the crude reaction mixture with CH_2Br_2 as an internal standard. [b]*ca.*50% starting material was remaining. [c]0.1 M donor.

Supplementary Table 3: Control Experiments and Counteranion Effects

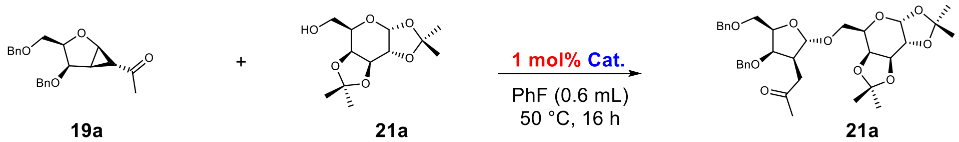

19a + 21a → 21a

Entry	Catalyst	Result[a]	Comments	Entry	Catalyst	Result[a]	Comments
1	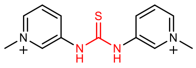 2 BArF⁻ **A**	>99% α/β 93:7		6	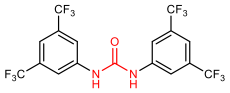 **C**	< 5%	
2	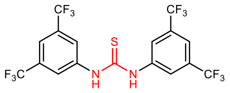 **D**	90% α/β 97:3	pKa = 8.4[8] (in DMSO)	7	$Et_3N·HCl$	39% α/β >95:5	pKa = 9.0[12] (in DMSO)
3	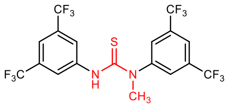 **G**	78% α/β 96:4		8	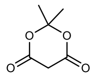 **I**	< 5%	pKa = 7.3[14] (in DMSO)
4	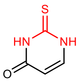 **H**	83% α/β >95:5	Uracil pKa: 14.1[9] (in DMSO) >25 kJ mol-1 more acidic when substituted by thiocarbonyl[10] 2-thiouracil pKa < 8.4	9	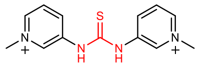 2 I⁻ **J**	< 5%	5 mol% **J** was used; **J** is not soluble in fluorobenzene
5	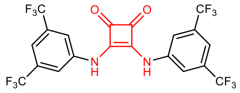 **E**	< 5%	pKa = 8.3[11] (in DMSO)	10	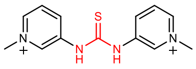 2 OTf⁻ **K**	90% α/β 95:5	

Supplementary Table 4: *In situ* NMR reaction monitoring at RT for furanosylation with **19a**

Time /min	Concentration/M				Time /min	Concentration/M			
	**[27a]**	**[27b]**	**[20a]**	**[19a]**		**[27a]**	**[27b]**	**[20a]**	**[19a]**
4.5	0.04	0.012	0.102	0.008	64.5	0	0.002	0.166	0
6.0	0.018	0.012	0.13	0	72.0	0	0.002	0.166	0
7.5	0.006	0.012	0.144	0	79.5	0	0	0.17	0
9.0	0.002	0.012	0.15	0	87.0	0	0	0.172	0
10.5	0	0.012	0.152	0	945	0	0	0.174	0
12.0	0	0.01	0.15	0	109.5	0	0	0176	0
13.5	0	0.01	0.152	0	124.5	0	0	0.18	0
15.0	0	0.01	0.148	0	130.5	0	0	0.178	0
16.5	0	0.01	0.154	0	150.5	0	0	0.178	0
18.0	0	0.01	0.154	0	170.5	0	0	0.174	0
22.5	0	0.008	0.154	0	190.5	0	0	0.182	0
27.0	0	0.008	0.15	0	210.5	0	0	0.18	0
31.5	0	0.008	0.154	0	230.5	0	0	0.178	0
36.0	0	0.006	0.156	0	250.5	0	0	0.18	0
40.5	0	0.006	0.158	0	270.5	0	0	0182	0
45.0	0	0.004	0.156	0	290.5	0	0	0.18	0
49.5	0	0.004	0.162	0	290.5	0	0	0.178	0
57.0	0	0.002	0.164	0					

Supplementary Table 5: *In situ* NMR reaction monitoring at 50 ℃ for furanosylation with **19a**

Time /min	Concentration/M				Time /min	Concentration/M			
	**[27a]**	**[27b]**	**[20a]**	**[19a]**		**[27a]**	**[27b]**	**[20a]**	**[19a]**
5.5	0	0.01	0.154	0	42.5	0	0	0.182	0
6.5	0	0.008	0.158	0	47.5	0	0	0.186	0
7.5	0	0.006	0.164	0	52.5	0	0	0.184	0
8.5	0	0.006	0.164	0	62.5	0	0	0.178	0
9.5	0	0.004	0.17	0	64.5	0	0	0.182	0
10.5	0	0.002	0.17	0	79.5	0	0	0.182	0
11.5	0	0.002	0.176	0	94.5	0	0	0.182	0
12.5	0	0	0.176	0	109.5	0	0	0.184	0
13.5	0	0	0.178	0	124.5	0	0	0.184	0
14.5	0	0	0.18	0	139.5	0	0	0.188	0
16.5	0	0	0.182	0	169.5	0	0	0.188	0
18.5	0	0	0.182	0	199.5	0	0	0.188	0
20.5	0	0	0.182	0	229.5	0	0	0.188	0
22.5	0	0	0.184	0	259.5	0	0	0.186	0
24.5	0	0	0.184	0	289.5	0	0	0.186	0
28.5	0	0	0.18	0	319.5	0	0	0.186	0
32.5	0	0	0.182	0	349.5	0	0	0.188	0
37.5	0	0	0.182	0					

Supplementary Table 6: Experiments information for initial rate kinetics

Reagents	Donor **19a** /M				Acceptor **21a**/M				cat. **A**/mol%		
	0.3	0.2	0.13	0.1	0.4	0.2	0.1	0.05	0.2	0.08	0.02
Donor **19a**/mg	52.8	35.2	23.4	17.6	35.2	35.2	35.2	35.2	35.2	35.2	35.2
Solution **A**/µL	10	10	10	10	10	10	10	10	10	4	1
Solution **B**/µL	200	200	200	200	200	100	50	25	200	200	200
Dibromomethane/µL	10.5	10.5	10.5	10.5	10.5	10.5	10.5	10.5	10.5	10.5	10.5
d^5-PhF/µL	280	280	280	280	280	380	430	455	280	286	289

Supplementary Table 7: Fitting and Calculation for initial rate kinetics

Object	Conc.	Fitting Equation	Derivative Equation	Slope (x=0)
Donor **19a**	0.30M	$y = 7.3067\times10^{-15}x^5 - 1.9672\times10^{-11}x^4 + 2.0302\times10^{-8}x^3 - 1.0004\times10^{-5}x^2 + 2.3979\times10^{-3}x - 3.0034\times10^{-6}$ $R^2 = 0.9992$	$y = 3.6533\times10^{-14}x^4 - 7.8688\times10^{-11}x^3 + 6.0906\times10^{-8}x^2 - 2.0008\times10^{-5}x + 2.3979\times10^{-3}$	2.3979×10^{-3}
	0.20M	$y = -2.4595\times10^{-12}x^4 + 5.1155\times10^{-9}x^3 - 3.8920\times10^{-6}x^2 + 1.3177\times10^{-3}x + 3.1386\times10^{-4}$ $R^2 = 0.9976$	$y = -9.8380\times10^{-12}x^3 + 1.5346\times10^{-8}x^2 - 7.7840\times10^{-6}x + 1.3177\times10^{-3}$	1.3177×10^{-3}
	0.13M	$y = -1.1313\times10^{-12}x^4 + 2.5661\times10^{-9}x^3 - 2.1522\times10^{-6}x^2 + 8.1146\times10^{-4}x + 9.5924\times10^{-5}$ $R^2 = 0.9966$	$y = -4.5252\times10^{-12}x^2 + 7.6983\times10^{-8}x^2 - 4.3044\times10^{-6}x + 8.1146\times10^{-4}$	8.1146×10^{-4}
	0.10M	$y = -1.5877\times10^{-12}x^4 + 3.0109\times10^{-9}x^3 - 2.0903\times10^{-6}x^2 + 6.5116\times10^{-4}x + 5.2801\times10^{-5}$ $R^2 = 0.9991$	$y = -6.3508\times10^{-12}x^2 + 9.0327\times10^{-8}x^2 - 4.1806\times10^{-6}x + 6.5116\times10^{-4}$	6.5116×10^{-4}
Acceptor **21a**	0.40M	$y = -2.4595\times10^{-12}x^4 + 5.1155\times10^{-9}x^3 - 3.8920\times10^{-6}x^2 + 1.3177\times10^{-3}x + 3.1386\times10^{-4}$ $R^2 = 0.9976$	$y = -9.8380\times10^{-12}x^3 + 1.5346\times10^{-8}x^2 - 7.7840\times10^{-6}x + 1.3177\times10^{-3}$	1.3177×10^{-3}
	0.20M	$y = -2.2743\times10^{-18}x^6 + 1.0888\times10^{-14}x^5 - 1.8262\times10^{-11}x^4 + 1.4550\times10^{-8}x^3 - 5.9522\times10^{-6}x^2 + 1.2734\times10^{-3}x + 6.1997\times10^{-8}$ $R^2 = 0.9991$	$y = 1.3648\times10^{-17}x^5 + 5.4440\times10^{-14}x^4 - 7.3048\times10^{-10}x^3 + 4.3650\times10^{-8}x^2 - 1.1904\times10^{-5}x + 1.2734\times10^{-3}$	1.2743×10^{-3}
	0.10M	$y = 4.9015\times10^{-16}x^5 + 1.5565\times10^{-12}x^4 + 1.8288\times10^{-9}x^3 - 1.0703\times10^{-6}x^2 + 3.6819\times10^{-4}x - 5.6170\times10^{-6}$ $R^2 = 0.9997$	$y = 2.4507\times10^{-15}x^4 + 6.2260\times10^{-12}x^3 + 5.4864\times10^{-8}x^2 - 2.1406\times10^{-6}x + 3.6819\times10^{-4}$	3.6819×10^{-4}
	0.05M	$y = 2.4962\times10^{-14}x^4 + 1.0765\times10^{-10}x^3 - 2.3128\times10^{-7}x^2 + 1.6346\times10^{-4}x - 1.4873\times10^{-5}$ $R^2 = 0.9996$	$y = 9.9848\times10^{-14}x^3 + 3.2295\times10^{-8}x^2 - 4.6256\times10^{-6}x + 1.6346\times10^{-4}$	1.6346×10^{-4}
Catalyst **A**	0.20%	$y = 8.0078\times10^{-17}x^6 - 1.4264\times10^{-13}x^5 + 8.7211\times10^{-11}x^4 - 1.6842\times10^{-8}x^3 - 2.9387\times10^{-6}x^2 + 1.5336\times10^{-3}x + 5.4830\times10^{-9}$ $R^2 = 0.9999$	$y = 4.8047\times10^{-16}x^5 + 7.1320\times10^{-13}x^4 + 3.4884\times10^{-10}x^3 – 5.0526\times10^{-8}x^2 - 5.8774\times10^{-3}x + 1.5336\times10^{-3}$	1.5336×10^{-3}
	0.08%	$y = -5.5382\times10^{-12}x^4 + 9.3436\times10^{-9}x^3 - 5.6850\times10^{-6}x^2 + 1.4961\times10^{-3}x + 1.2504\times10^{-4}$ $R^2 = 0.9997$	$y = -1.6275\times10^{-11}x^3 + 2.8031\times10^{-8}x^2 - 1.1370\times10^{-5}x + 1.4961\times10^{-3}$	1.4961×10^{-3}
	0.02%	$y = 1.4445\times10^{-9}x^3 – 2.0155\times10^{-6}x^2 + 9.5625\times10^{-4}x – 3.5539\times10^{-3}$ $R^2 = 0.9798$	$y = 4.3335\times10^{-9}x^2 – 4.0310\times10^{-6}x + 9.5625\times10^{-4}$	9.5625×10^{-4}

Supplementary Table 8: Calculation for reaction order

Data for Donor **19a**		Data for Acceptor **21a**		Data for cat. **A**	
ln(rate)	ln[**19a**]	ln(rate)	ln[**21a**]	ln(rate)	ln[cat. **A**]
-6.03312	-1.20397	-6.63164	-0.91629	-6.47988	-8.51719
-6.63164	-1.60944	-6.66638	-1.60944	-6.50496	-9.43348
-7.11724	-2.04022	-7.90743	-2.30259	-6.95275	-10.81978
-7.33700	-2.30259	-8.72176	-2.99573		
ln(rate) = 1.19×ln[**19a**] - 4.65		ln(rate) = 1.08×ln[**21a**] – 5.36		ln(rate) = 0.21×ln[cat. **A**] – 4.58	
Order *x* = 1.19		Order *x* = 1.08		Order *x* = 0.21	

Supplementary Figures:

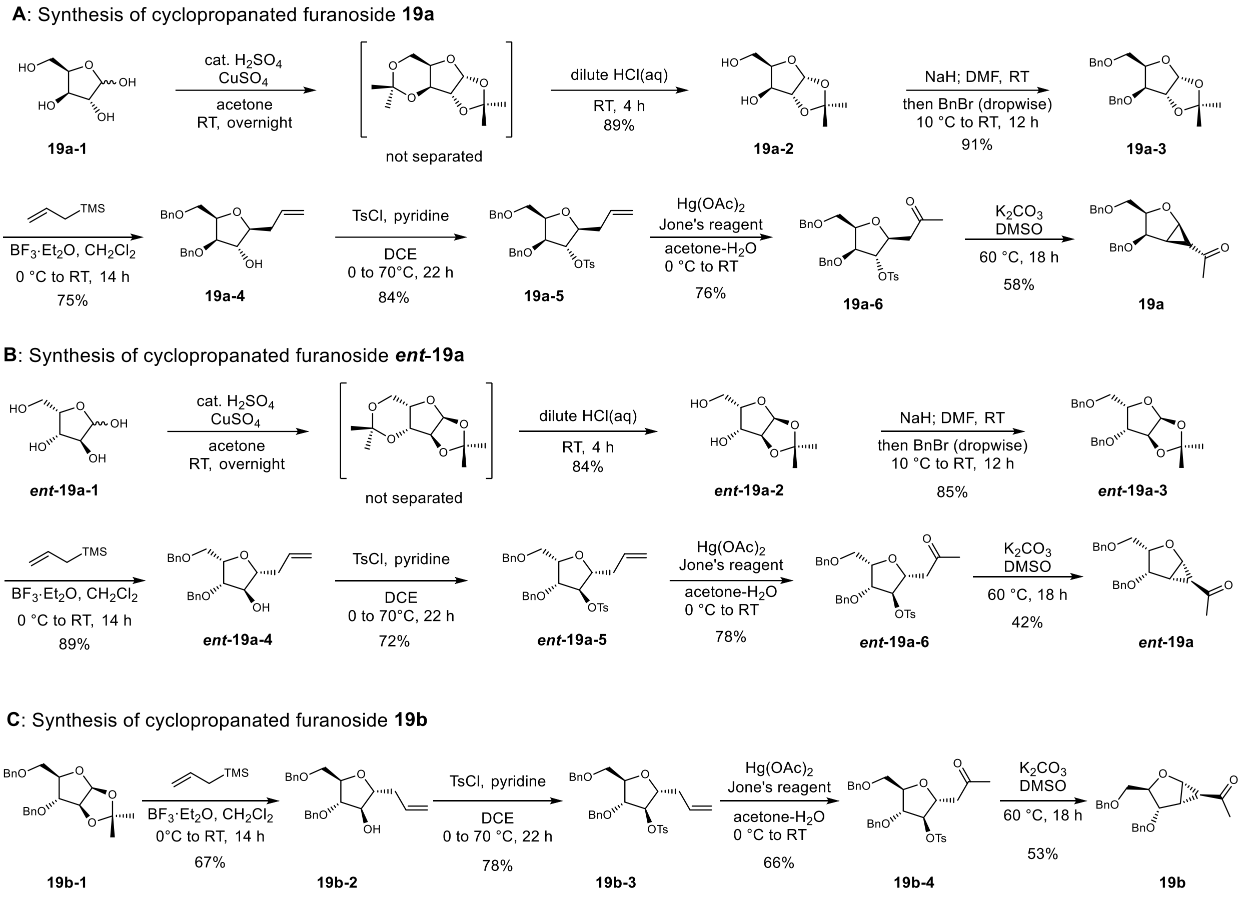

Supplementary Figure 1: General methods for synthesis of cyclopropanated furanosides. (**A**) Synthesis of cyclopropanated furanoside **19a**. (**B**) Synthesis of cyclopropanated furanoside ***ent*-19a**. (**C**) Synthesis of cyclopropanated furanoside **19b**.

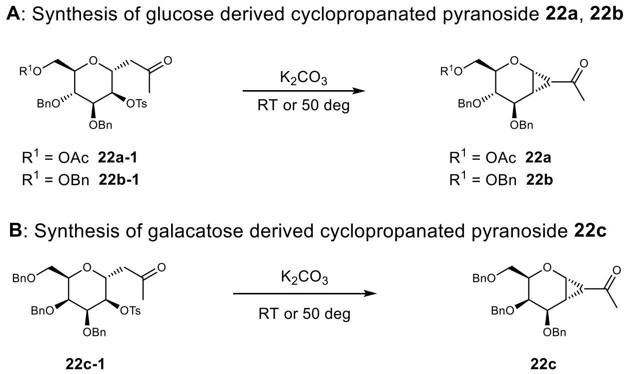

Supplementary Figure 2. General methods for synthesis of cyclopropanated furanosides. (**A**) Synthesis of glucose derived cyclopropanated pyranoside **22a**, **22b**. (**B**) Synthesis of galactose derived cyclopropanated pyranoside **22c**.

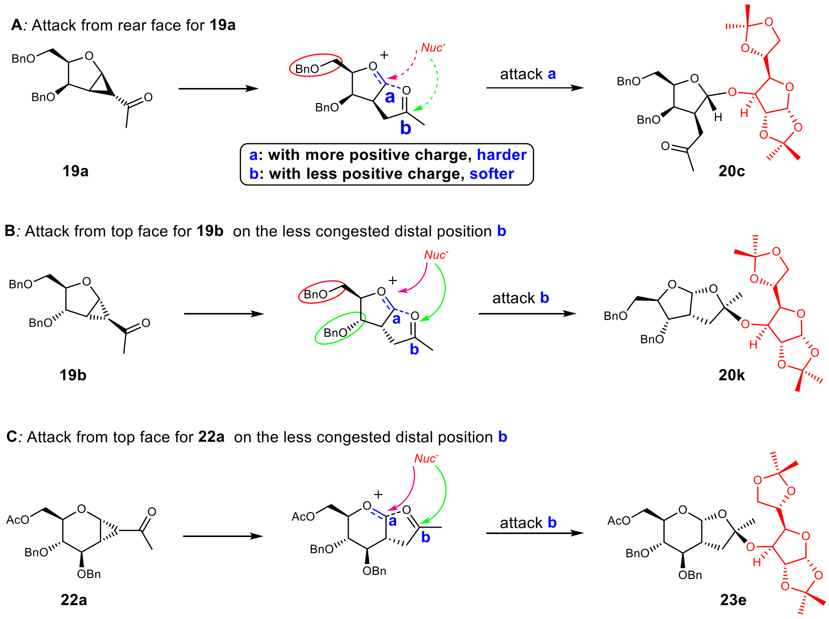

Supplementary Figure 3. Plausible mechanism for the formation of bicyclic **20k** and **23e**. (**A**) Attack from rear face for **19a**. (**B**) Attack from top face for **19b** on the less congested distal position b. (**C**) Attack from top face for **22a** on the less congested distal position b.

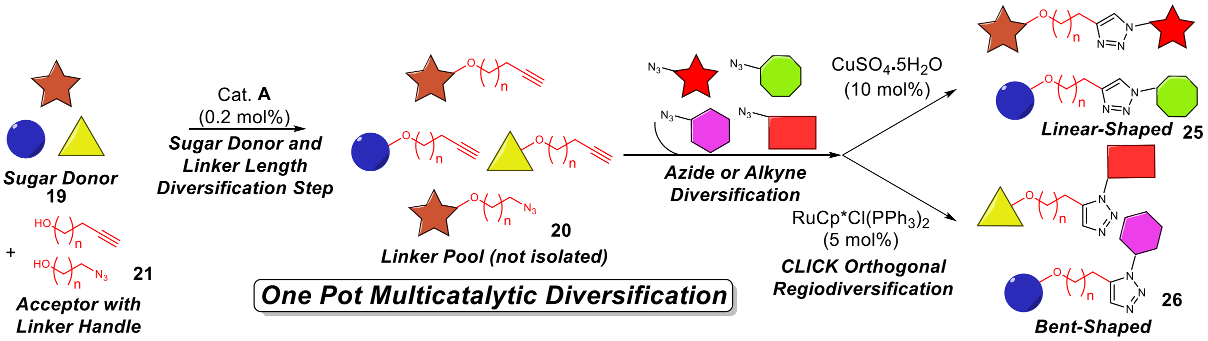

Supplementary Figure 4. General representation for the multicatalytic diversification

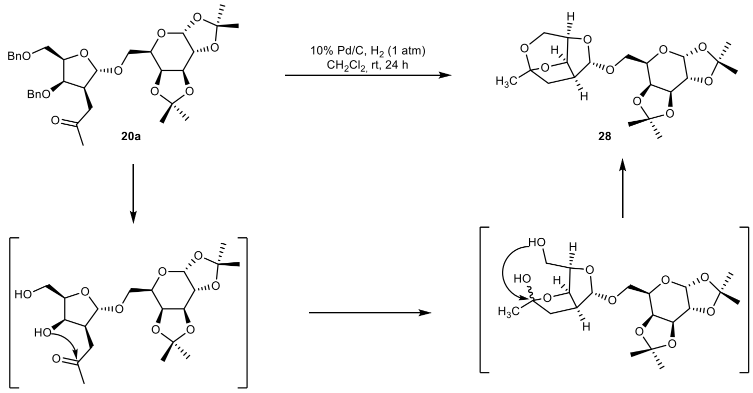

Supplementary Figure 5. Hemiketalization/ketalization cascade to form **28**

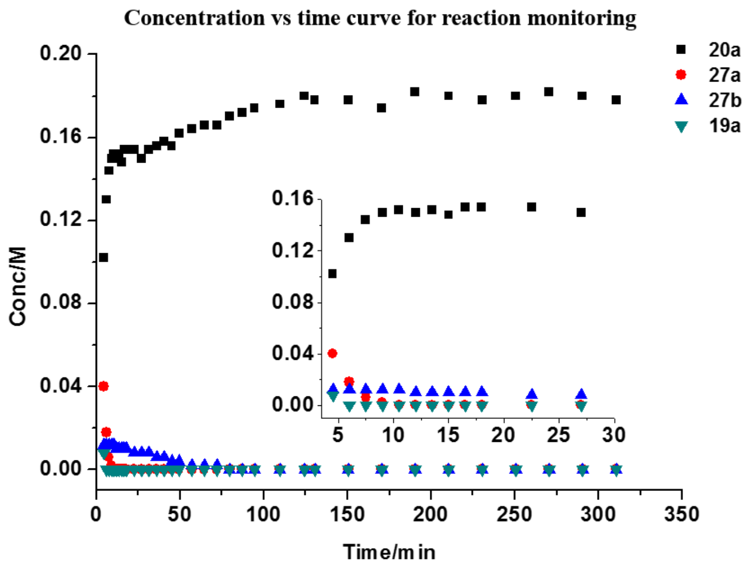

Supplementary Figure 6. Concentration vs time graph for reaction monitoring at RT

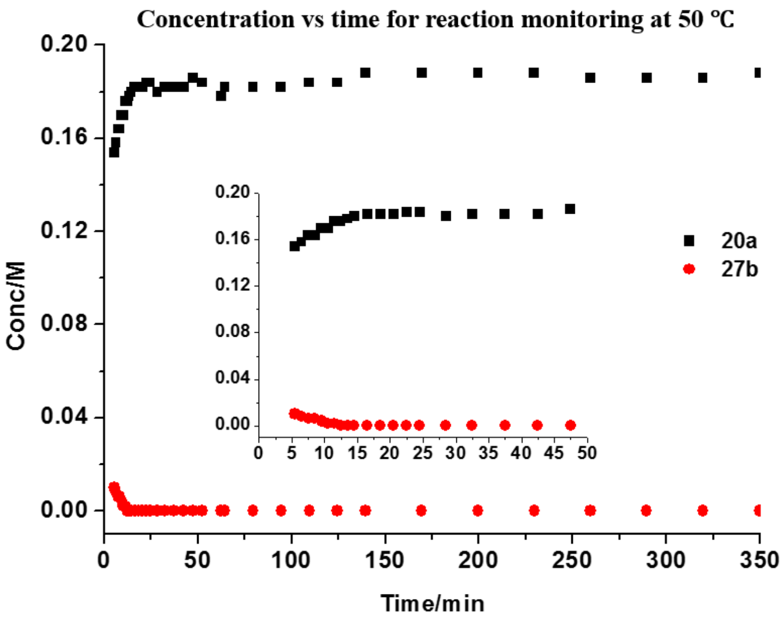

Supplementary Figure 7. Concentration vs time graph for reaction monitoring at 50°C

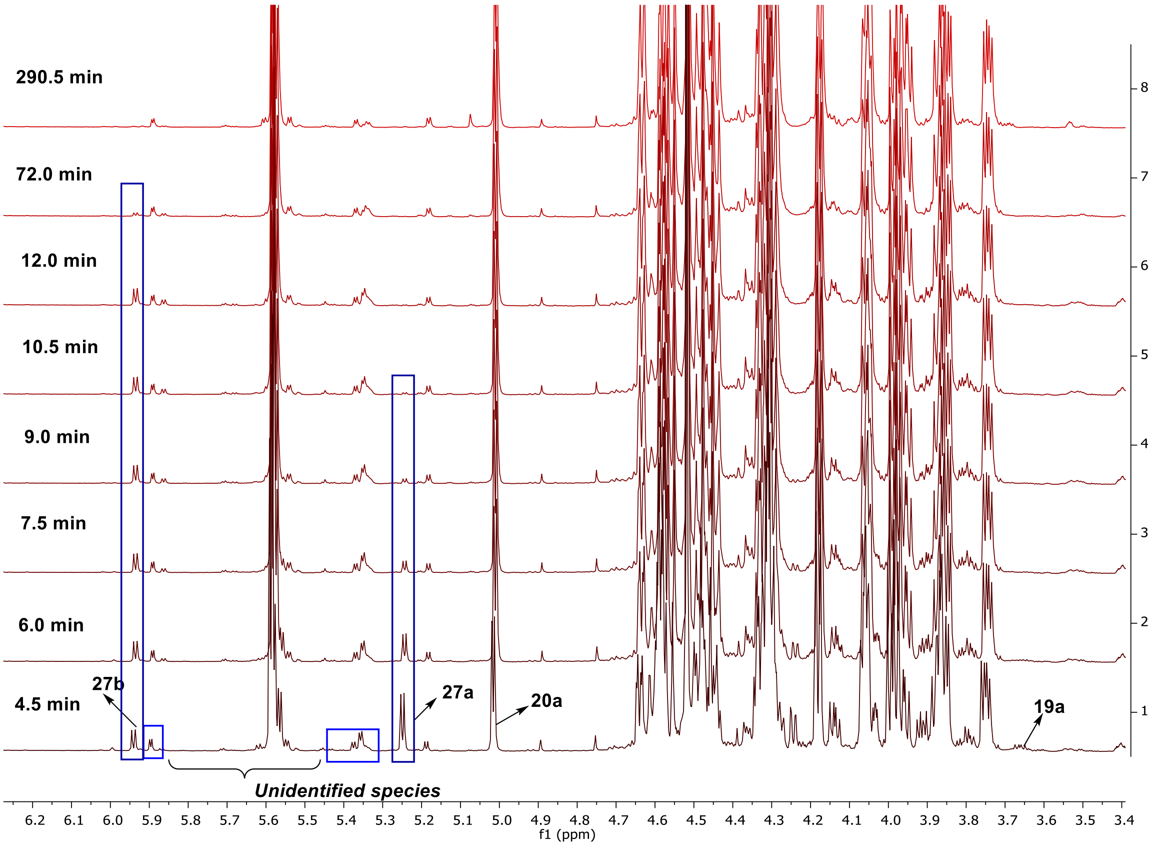

Supplementary Figure 8. Stacked ^{1}H spectra plot for reaction monitoring at RT

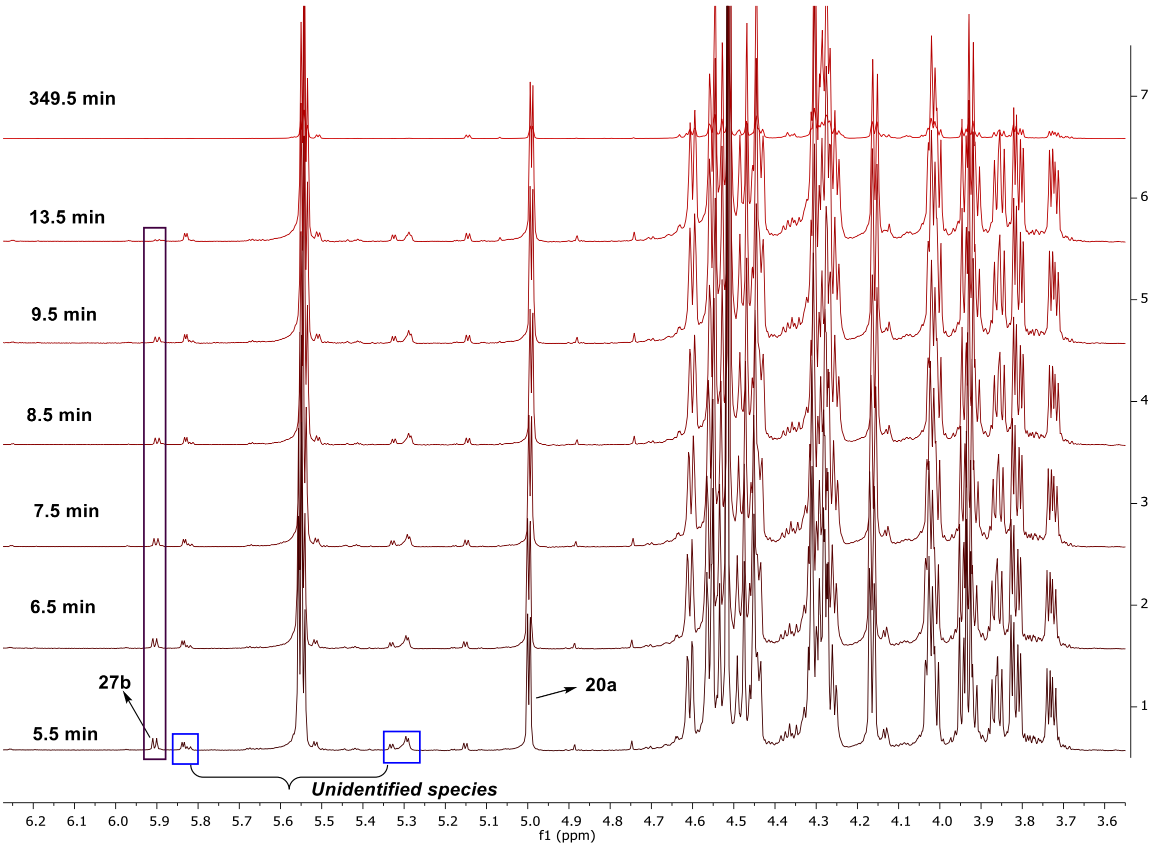

Supplementary Figure 9. Stacked ^{1}H spectra plot for reaction monitoring at 50℃

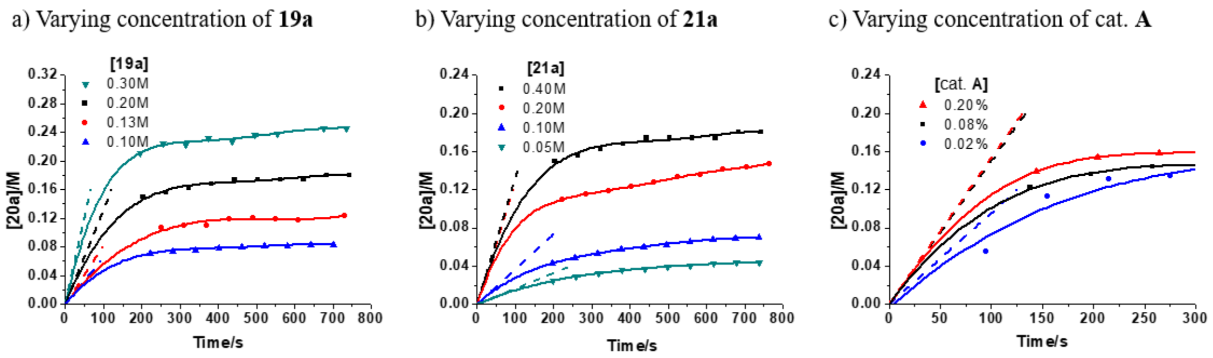

Supplementary Figure 10. Graphs for initial rate kinetics

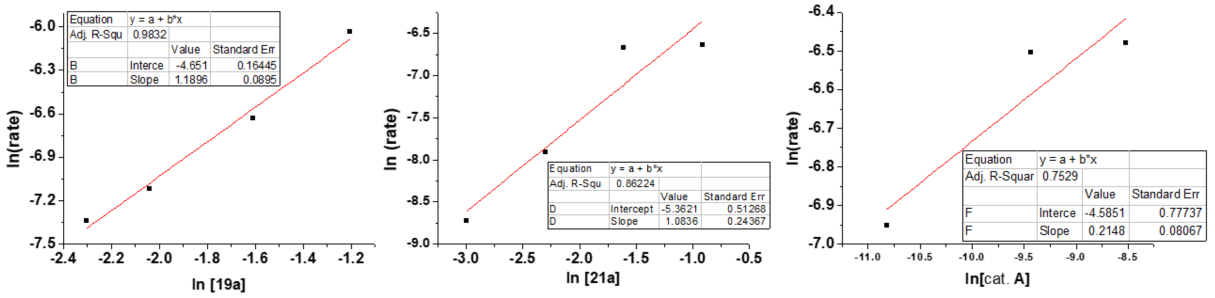

Supplementary Figure 11. Graphs for calculation for reaction order

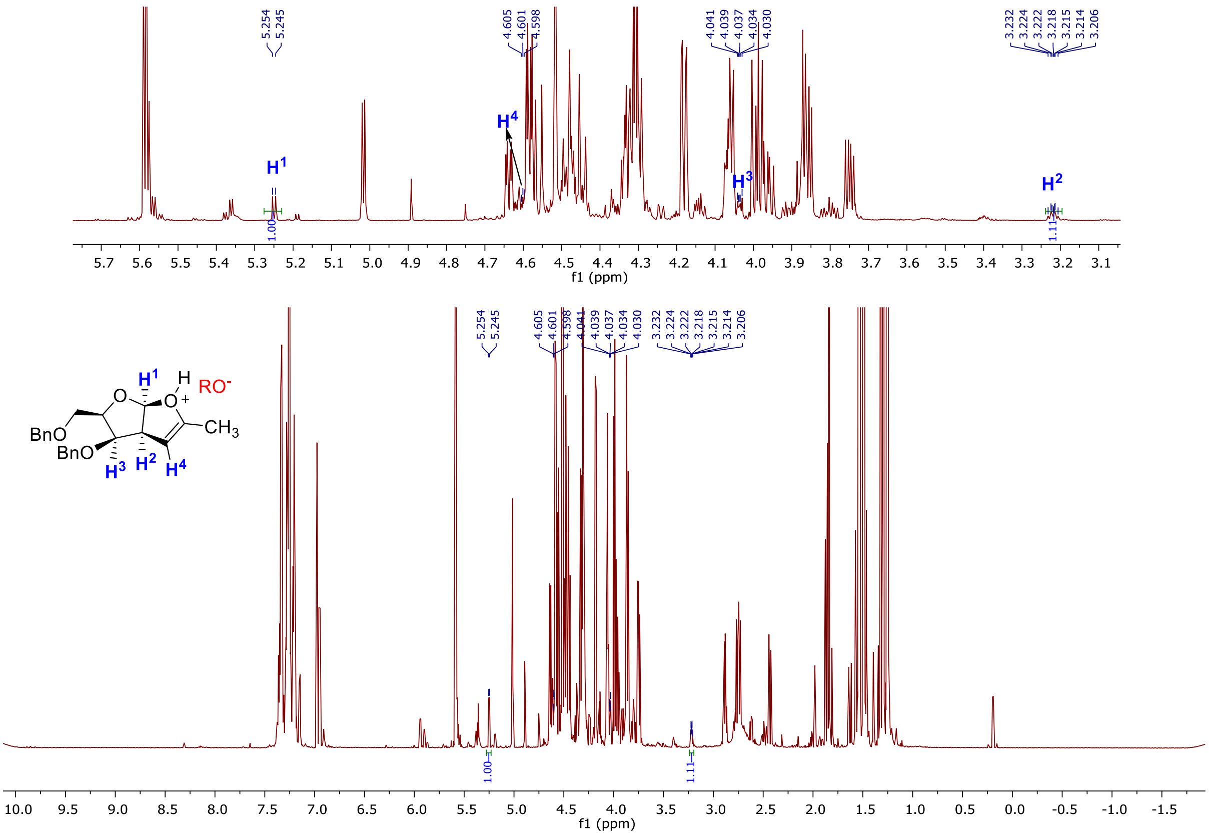

Supplementary Figure 12. *In situ* ^{1}H spectra for intermediate **27a**

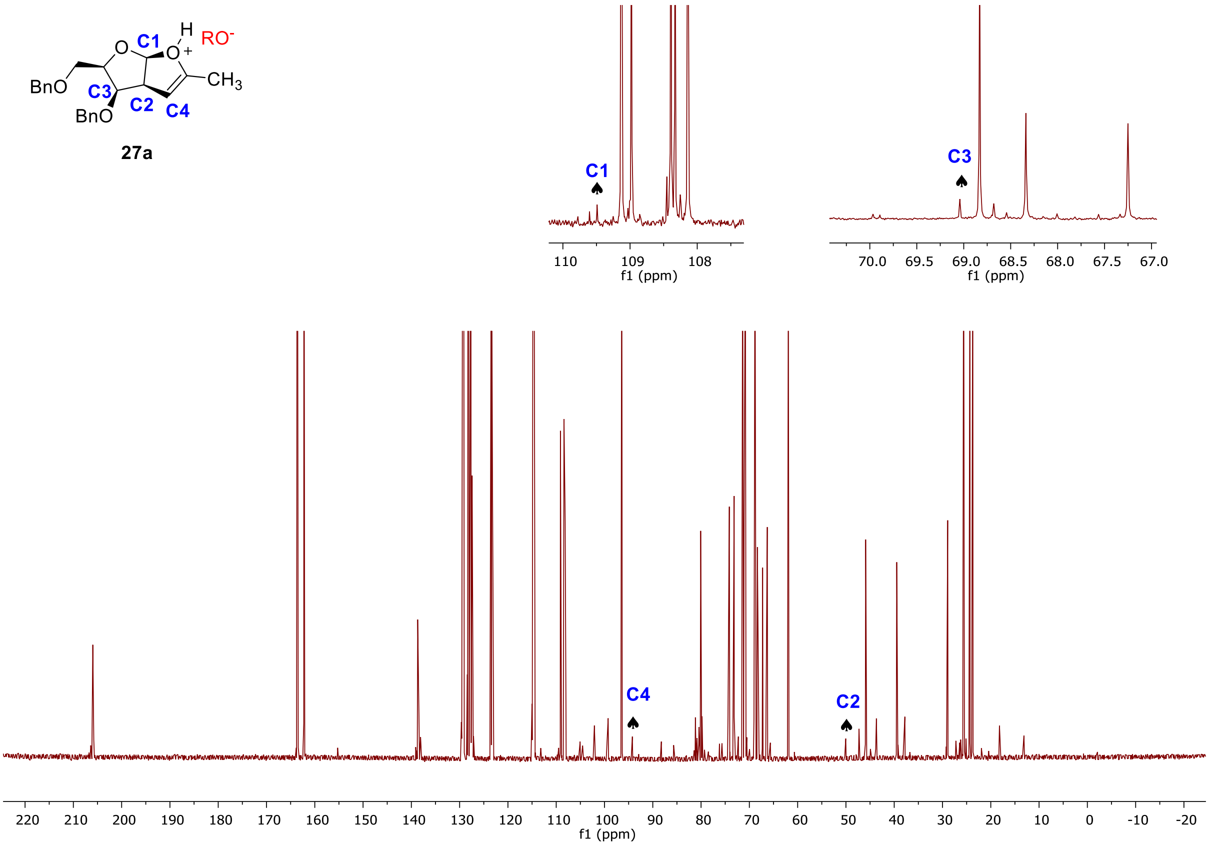

Supplementary Figure 13. *In situ* ^{13}C spectra for intermediate **27a**

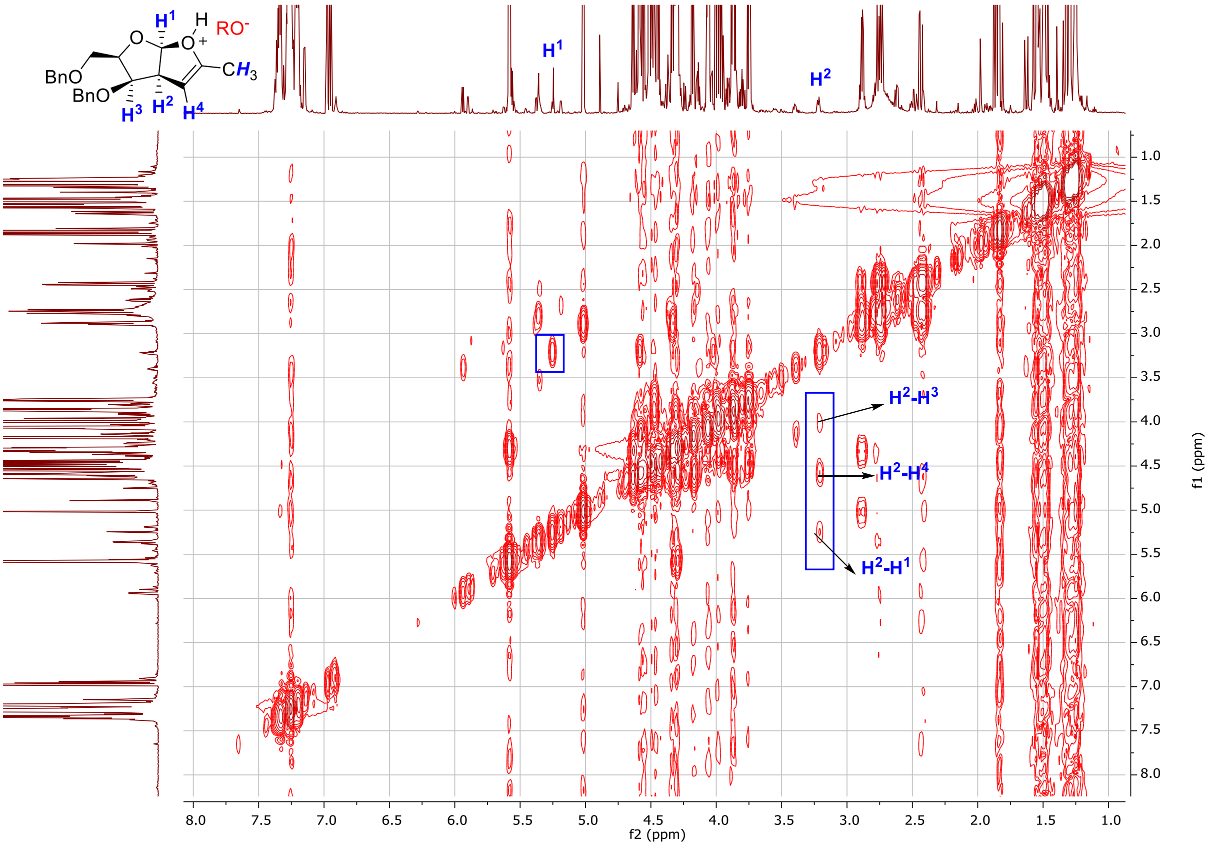

Supplementary Figure 14. *In situ* COSY spectra for intermediate **27a**

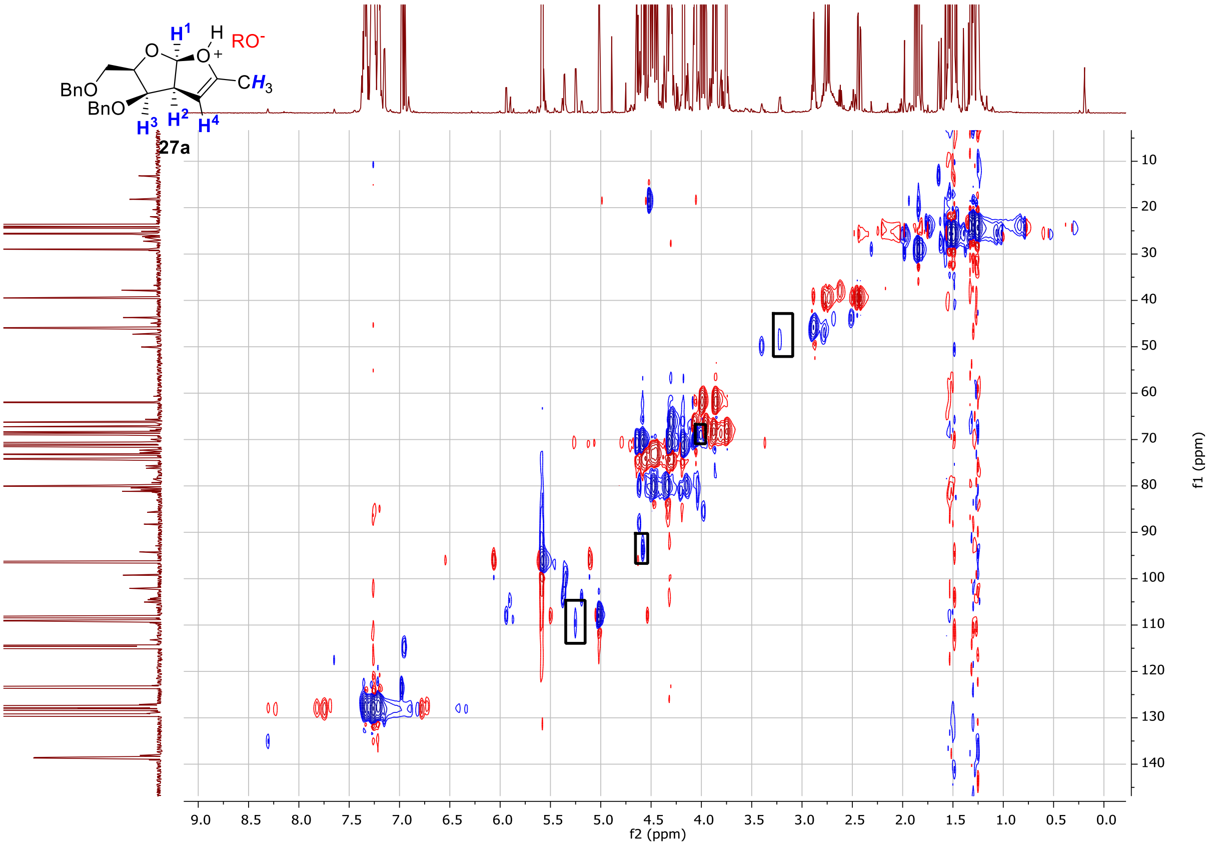

Supplementary Figure 15. *In situ* HSQC spectra for intermediate **27a**

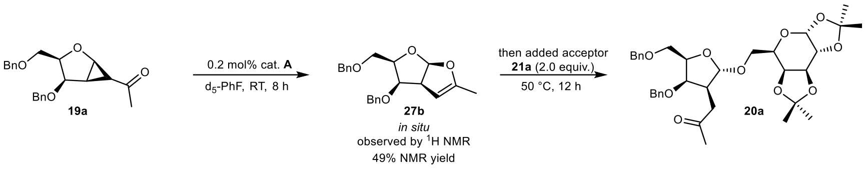

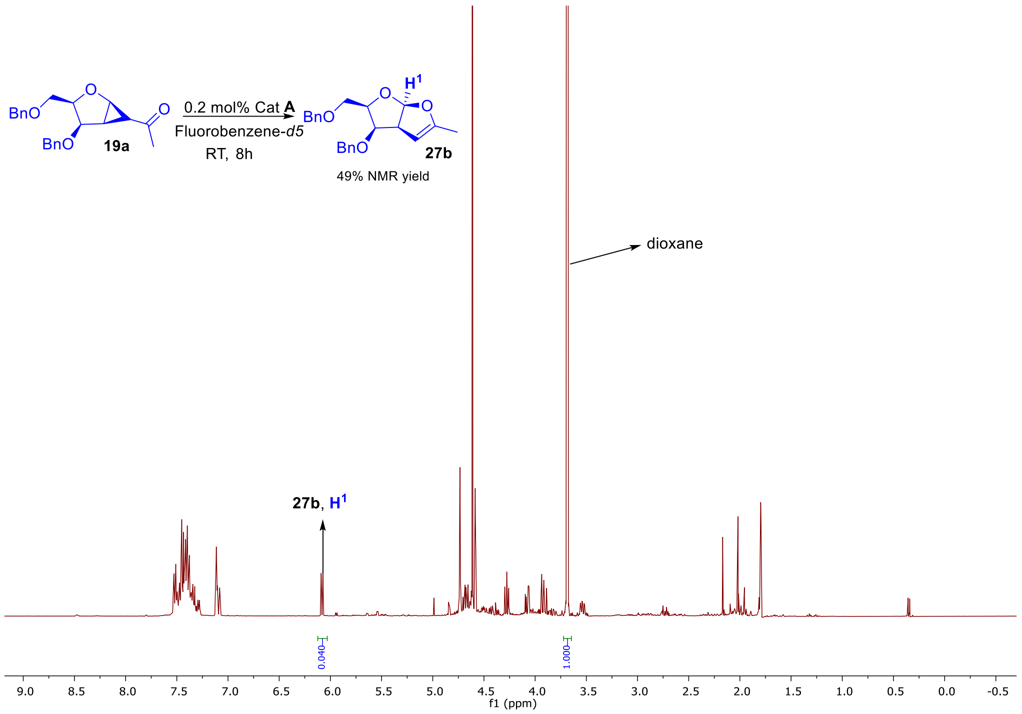

Spectrum of the final crude solution after heating for 12 h

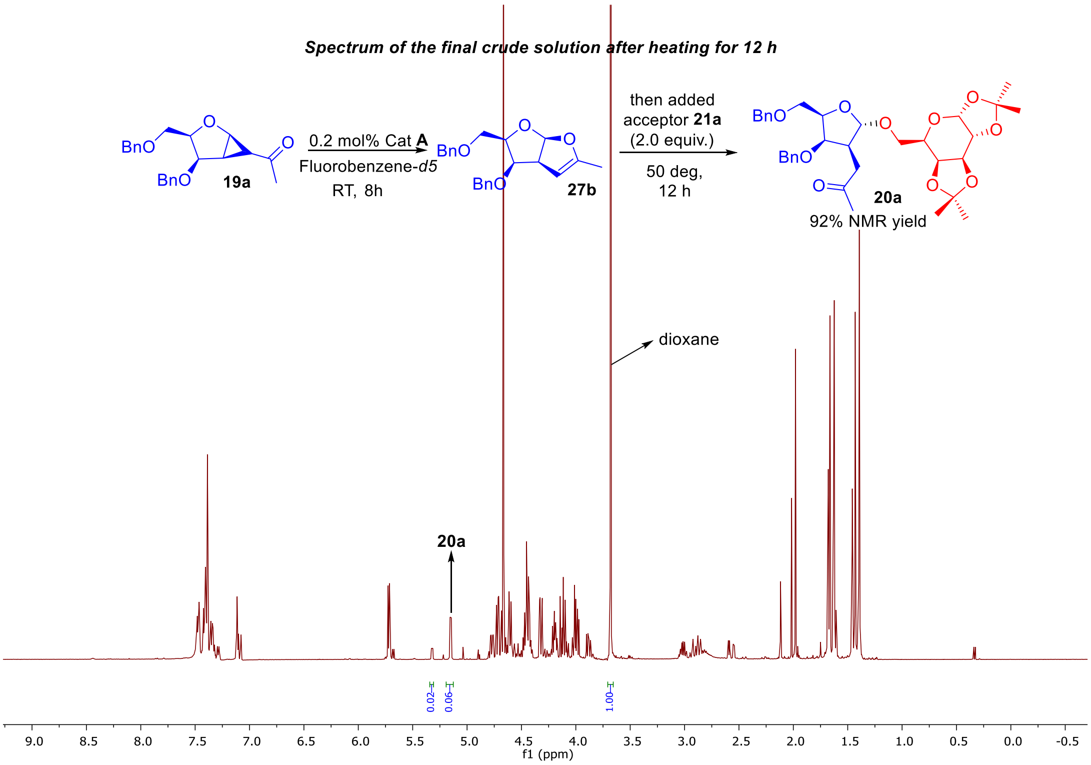

Supplementary Figure 16. Sequential addition control experiment

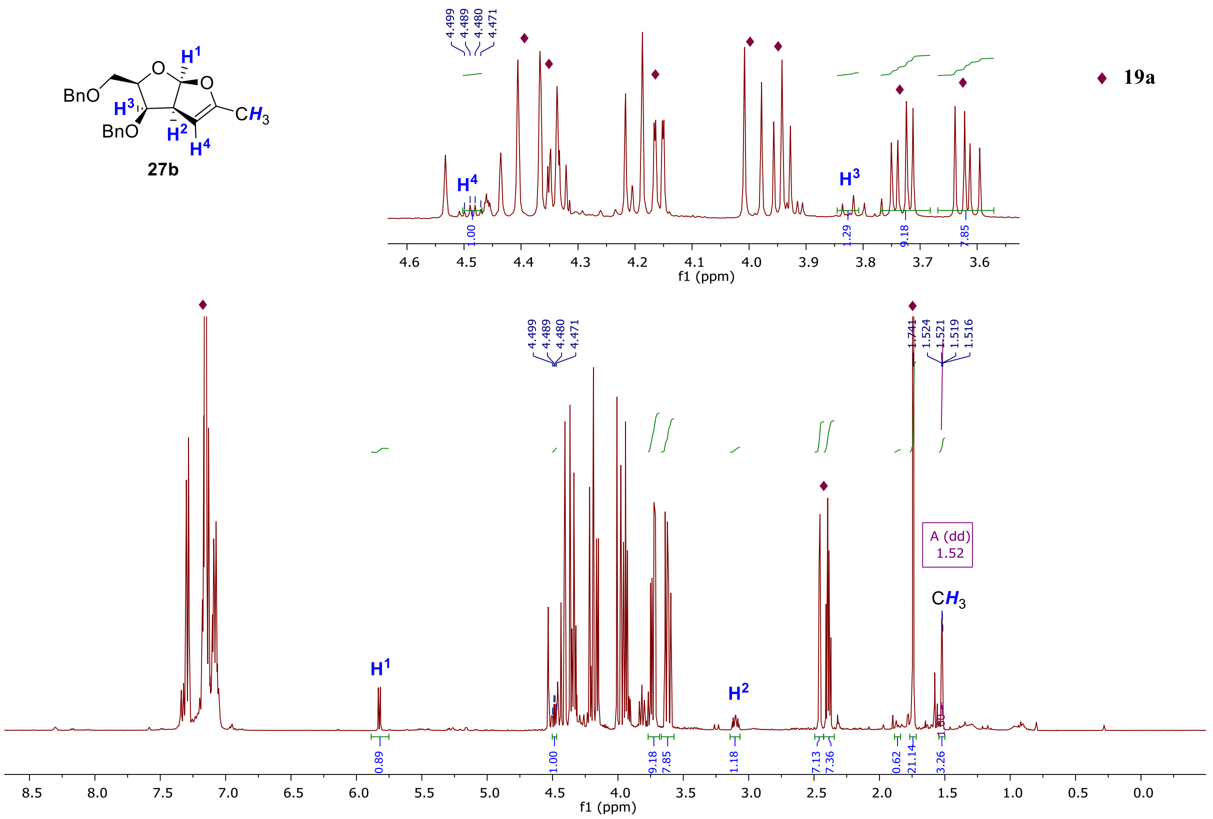

Supplementary Figure 17. *In situ* ^{1}H spectra for intermediate **27b**

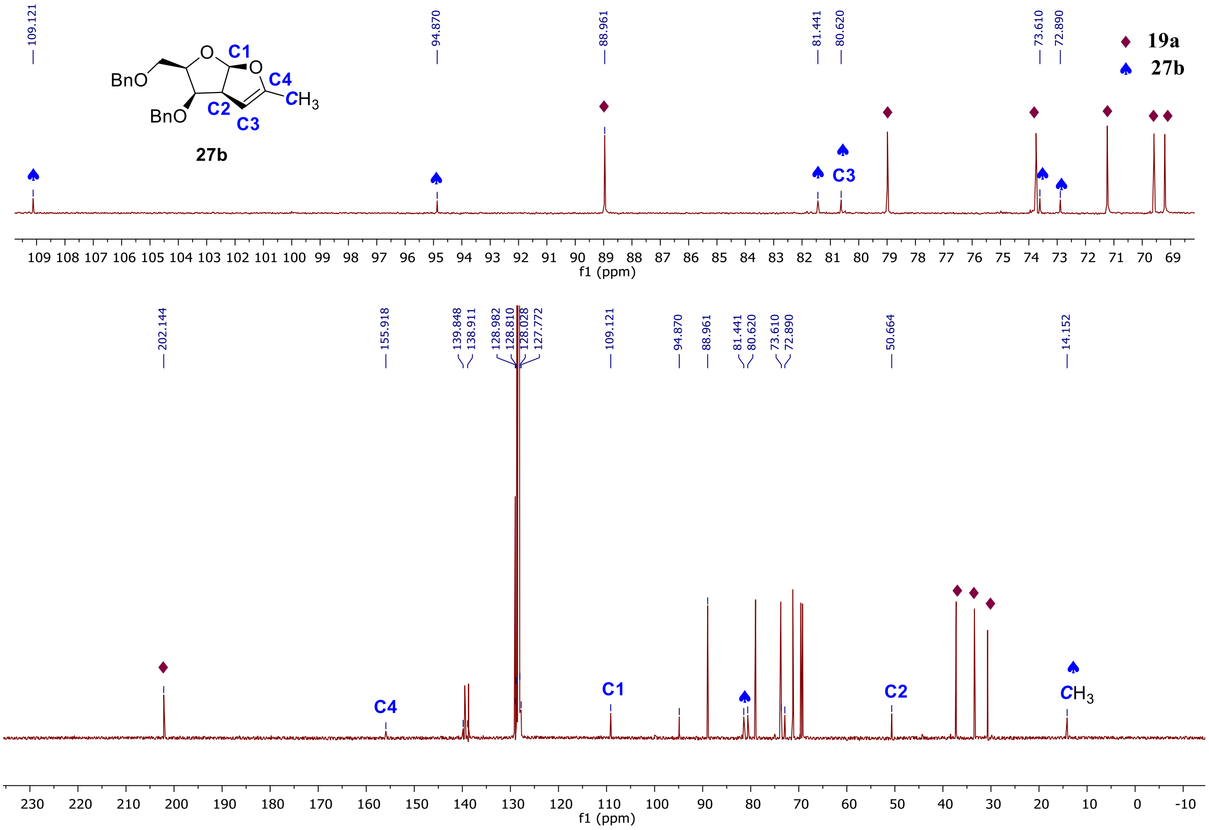

Supplementary Figure 18. *In situ* ^{13}C spectra for intermediate **27b**

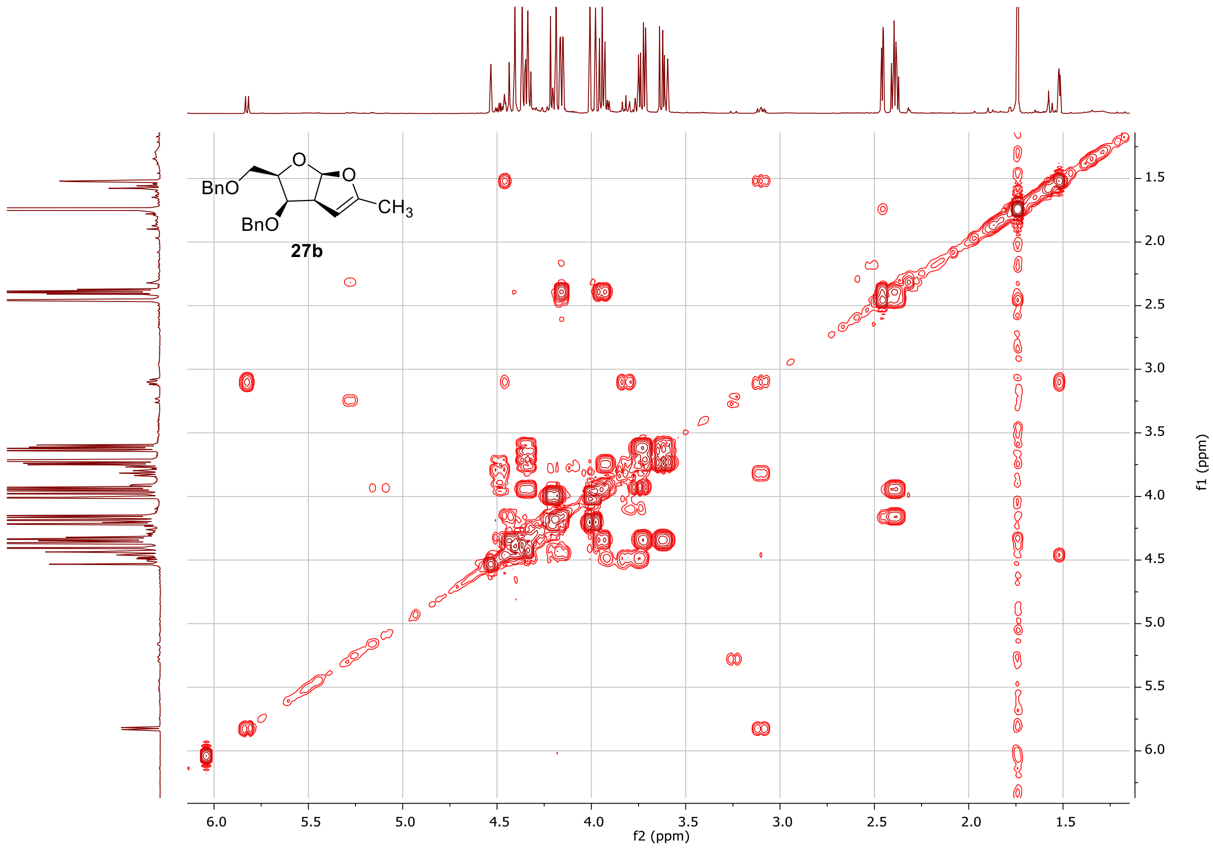

Supplementary Figure 19. *In situ* COSY spectra for intermediate **27b**

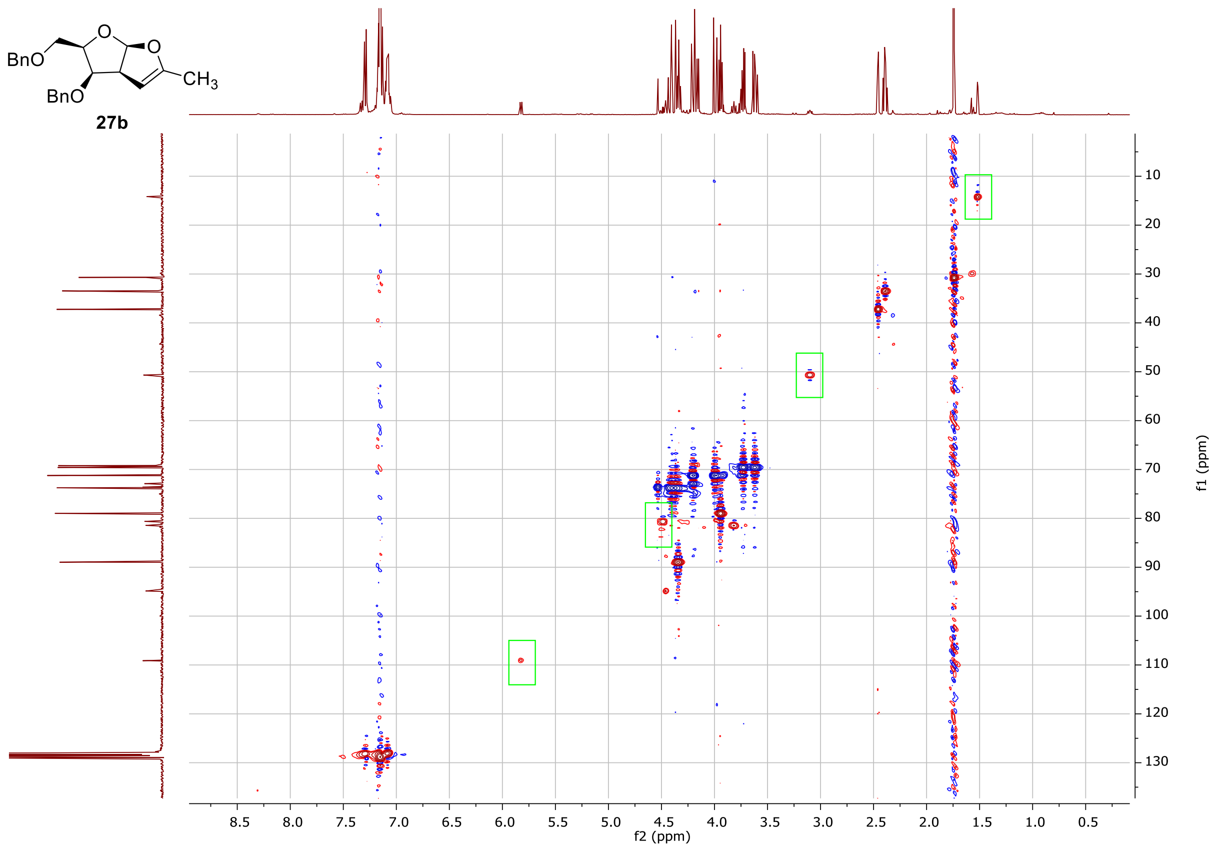

Supplementary Figure 20. *In situ* HSQC spectra for intermediate **27b**

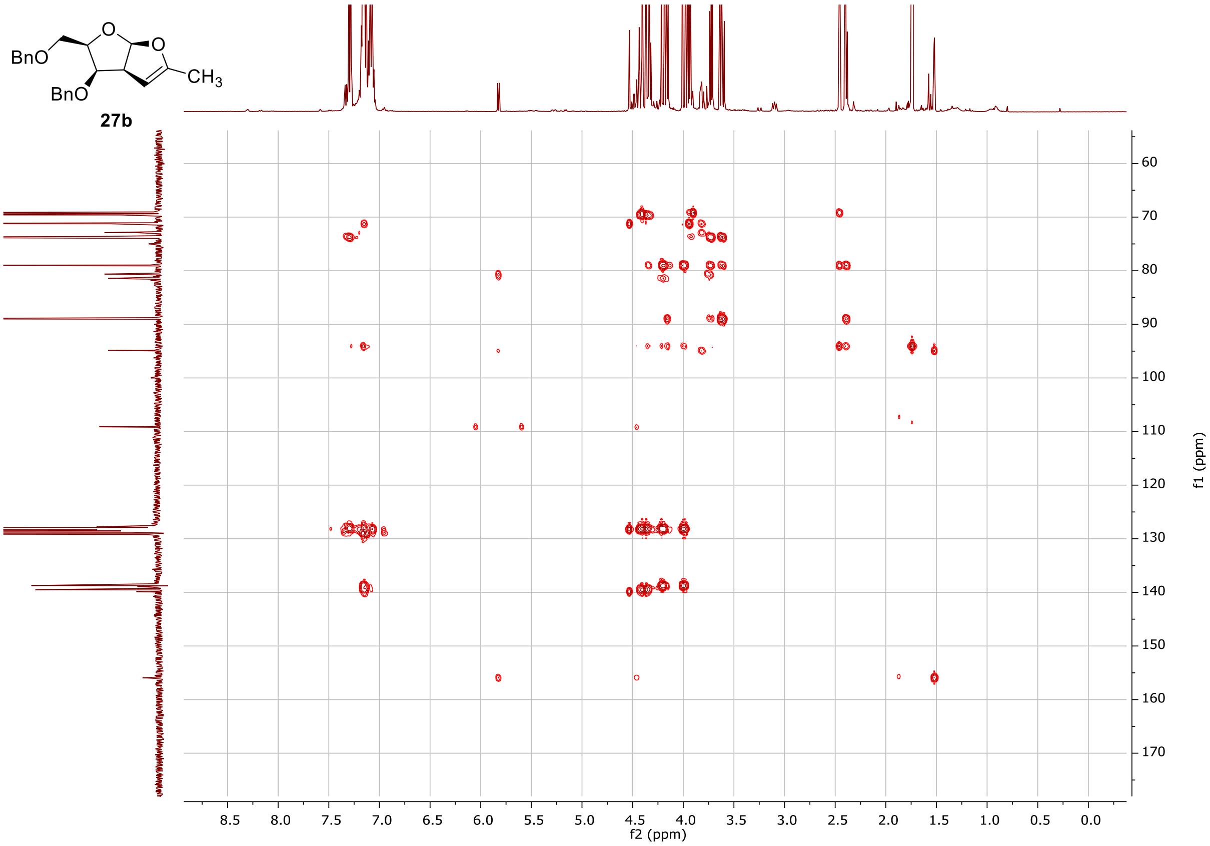

Supplementary Figure 21. *In situ* HMBC spectra for intermediate **27b**

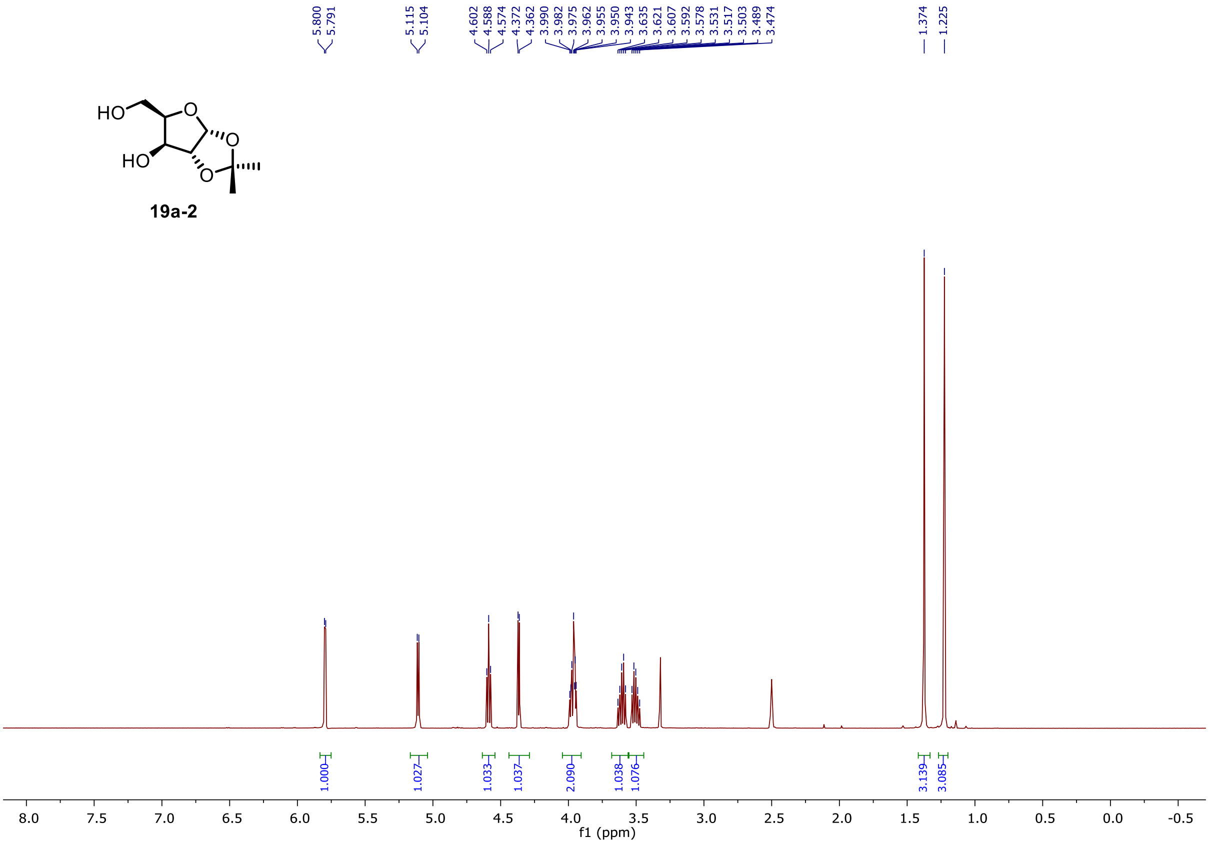

Supplementary Figure 22. ^{1}H spectra for **19a-2**

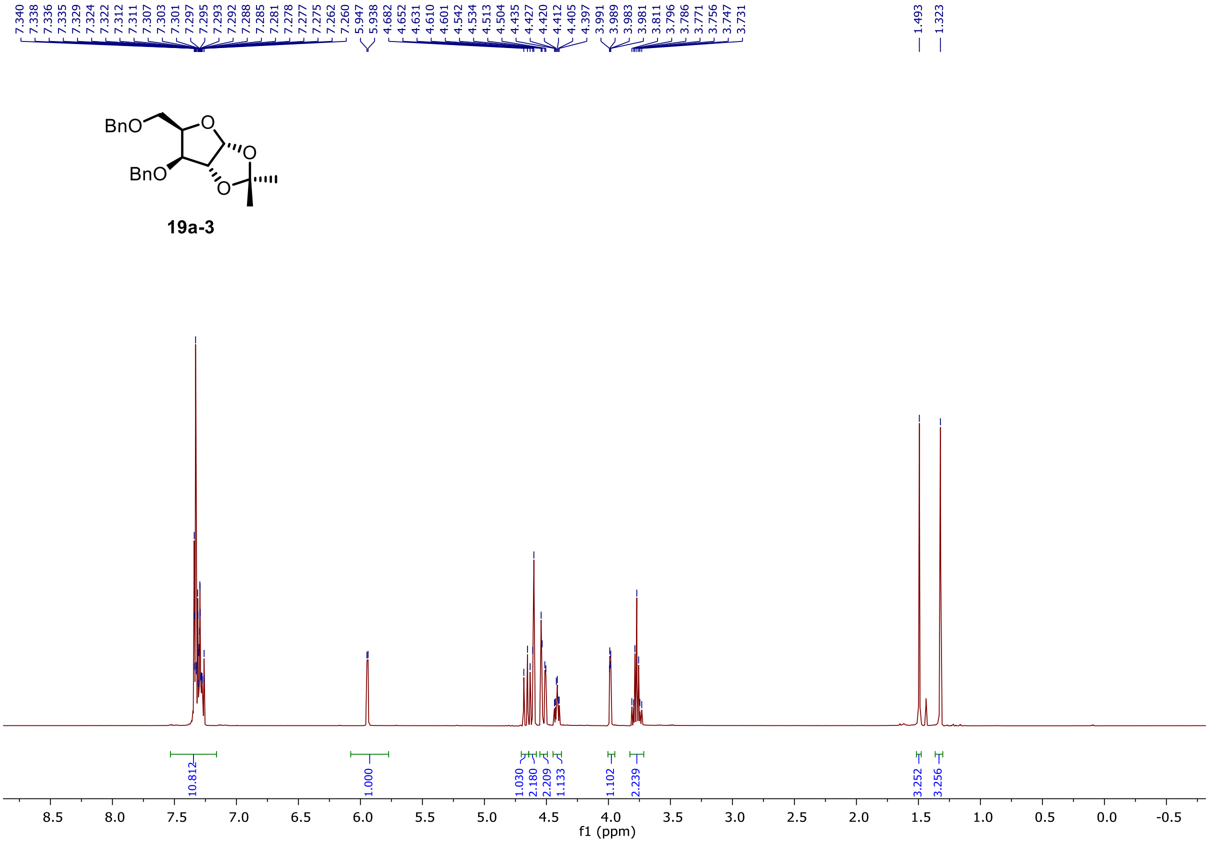

Supplementary Figure 23. ^{1}H spectra for **19a-3**

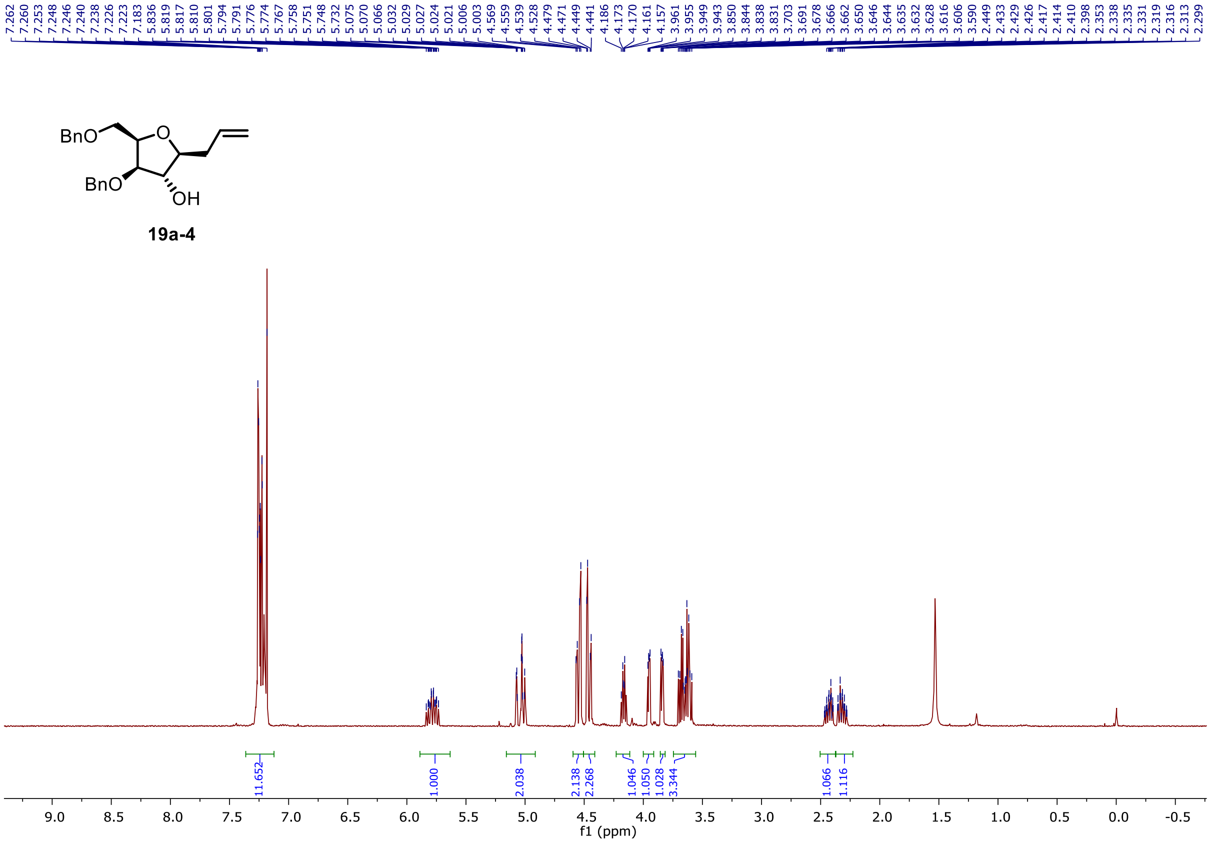

Supplementary Figure 24. ^{1}H spectra for **19a-4**

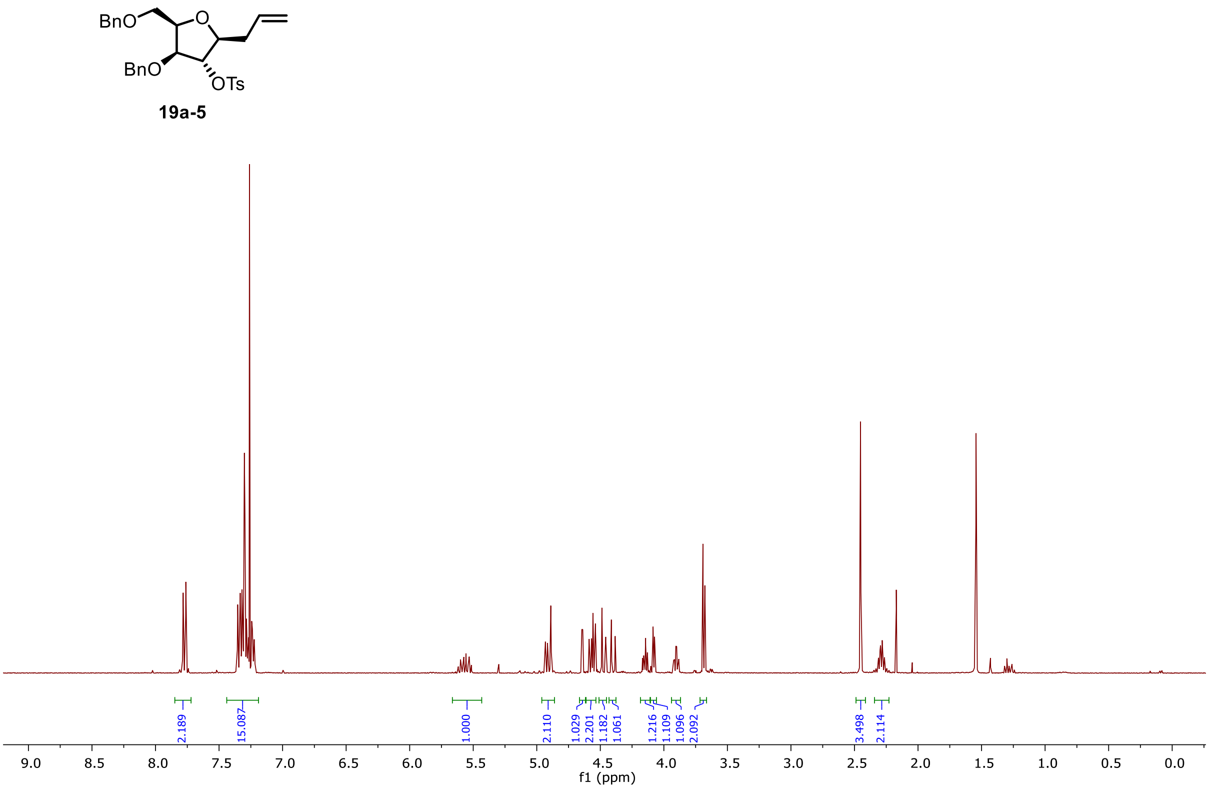

Supplementary Figure 25. ^{1}H spectra for **19a-5**

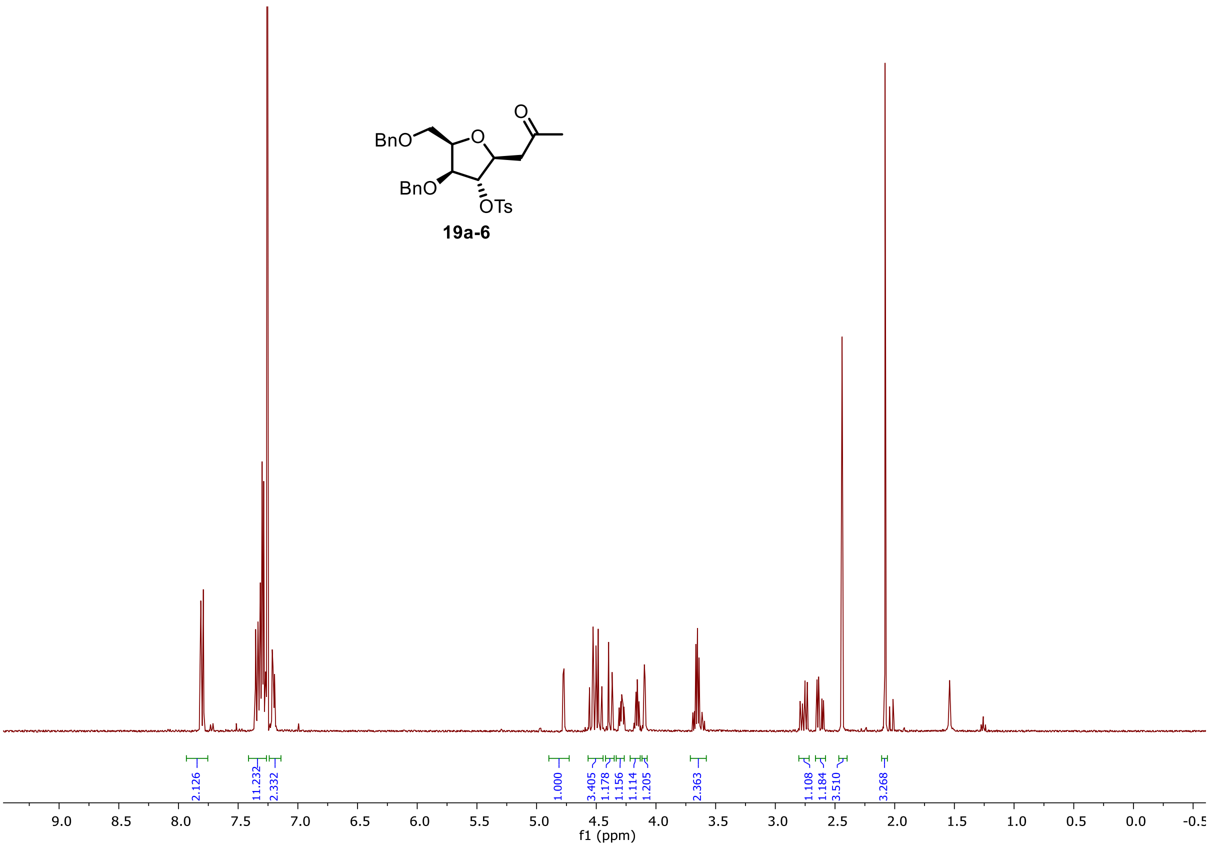

Supplementary Figure 26. ^{1}H spectra for **19a-6**

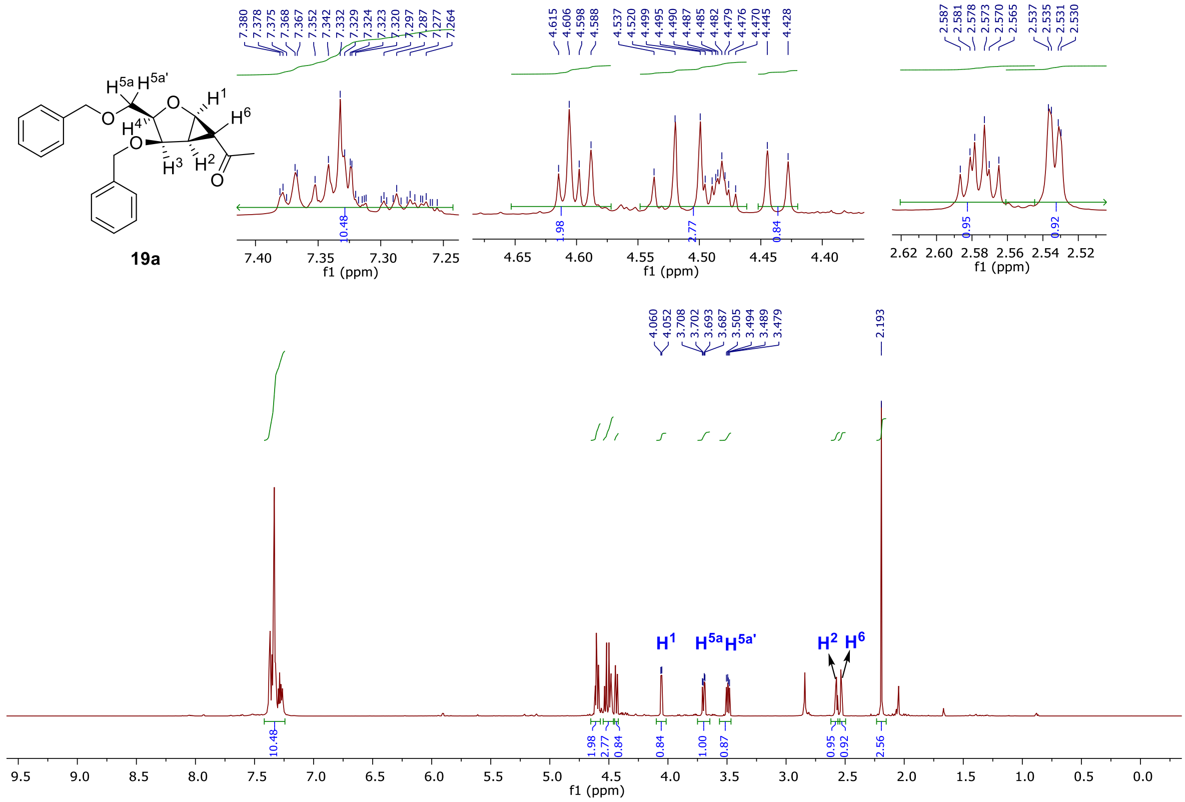

Supplementary Figure 27. ^{1}H spectra for **19a**

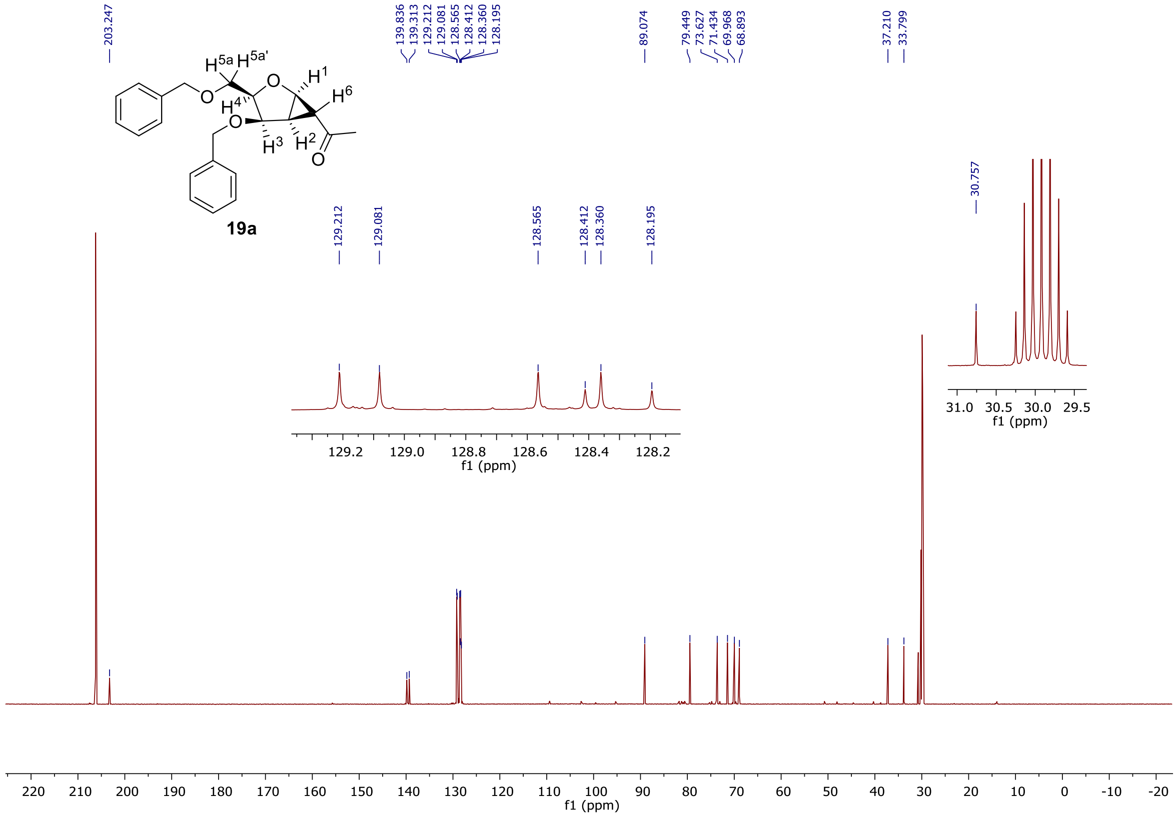

Supplementary Figure 28. ^{13}C spectra for **19a**

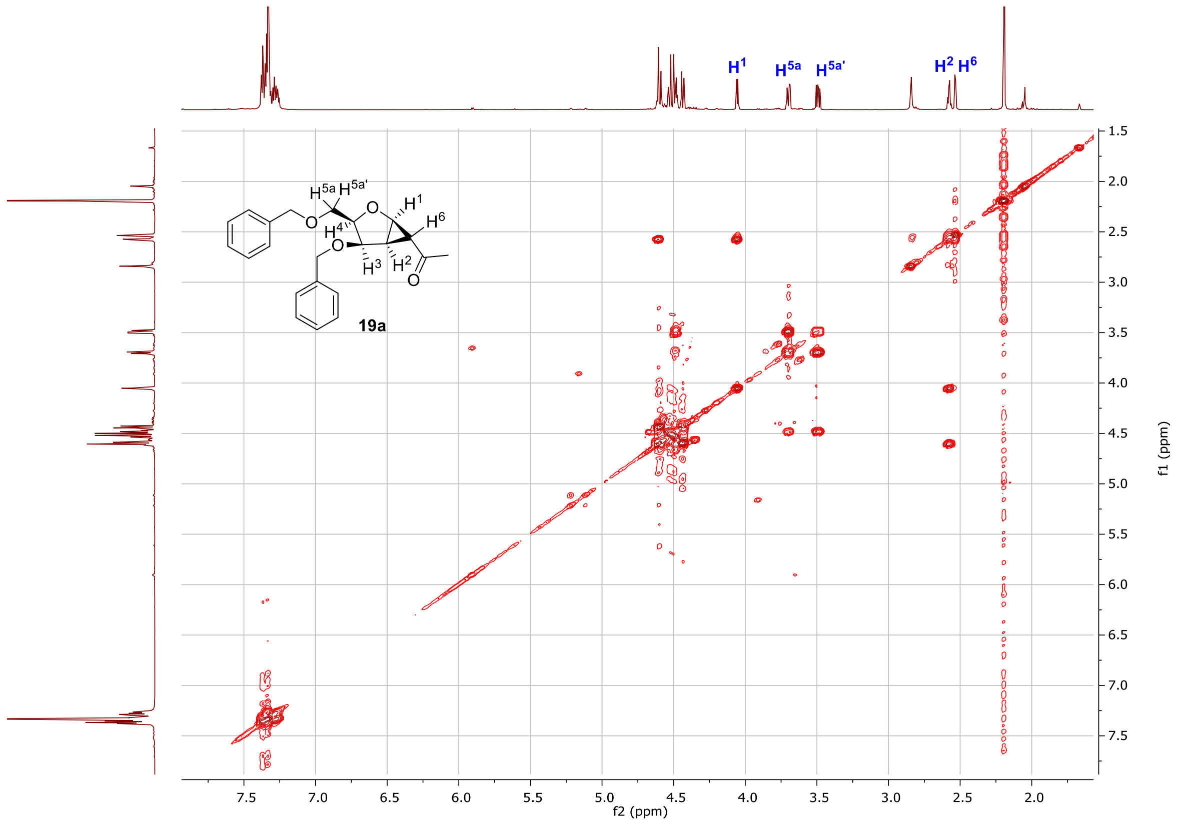

Supplementary Figure 29. COSY spectra for **19a**

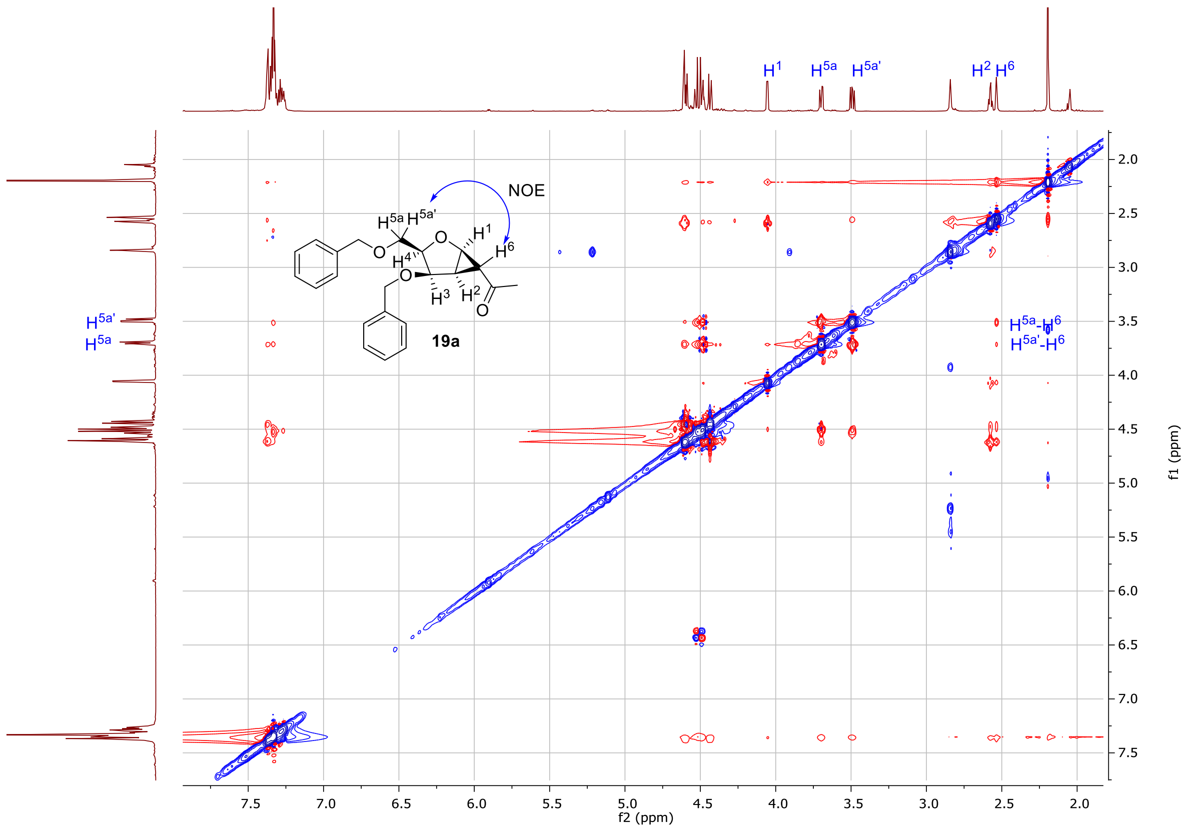

Supplementary Figure 30. NOESY spectra for **19a**

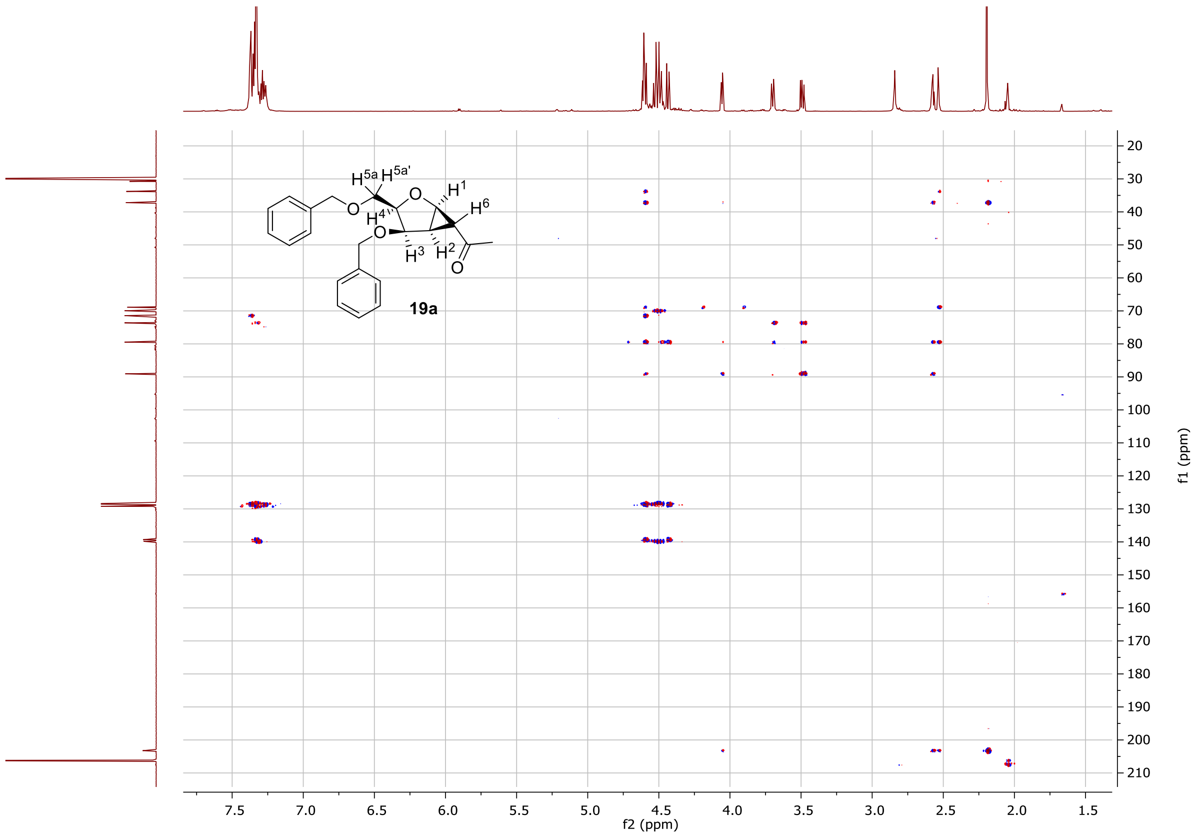

Supplementary Figure 31. HMBC spectra for **19a**

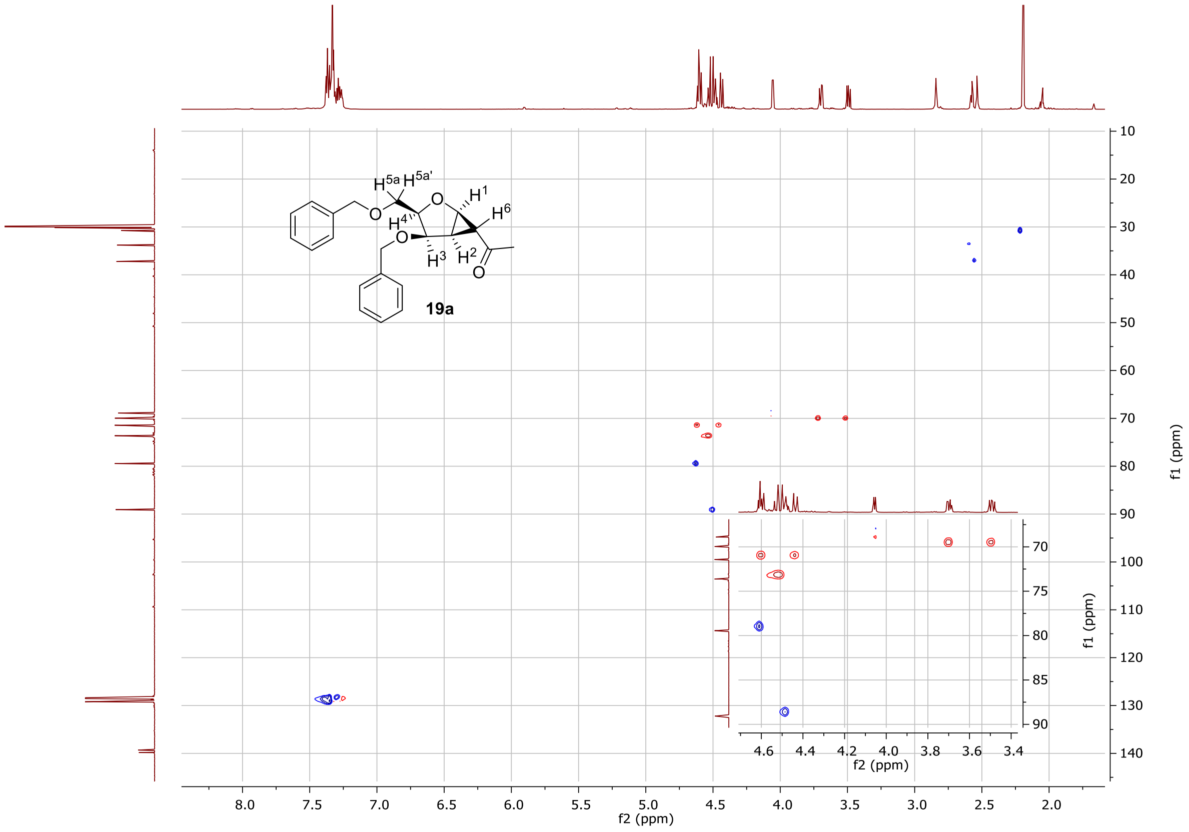

Supplementary Figure 32. HSQC spectra for **19a**

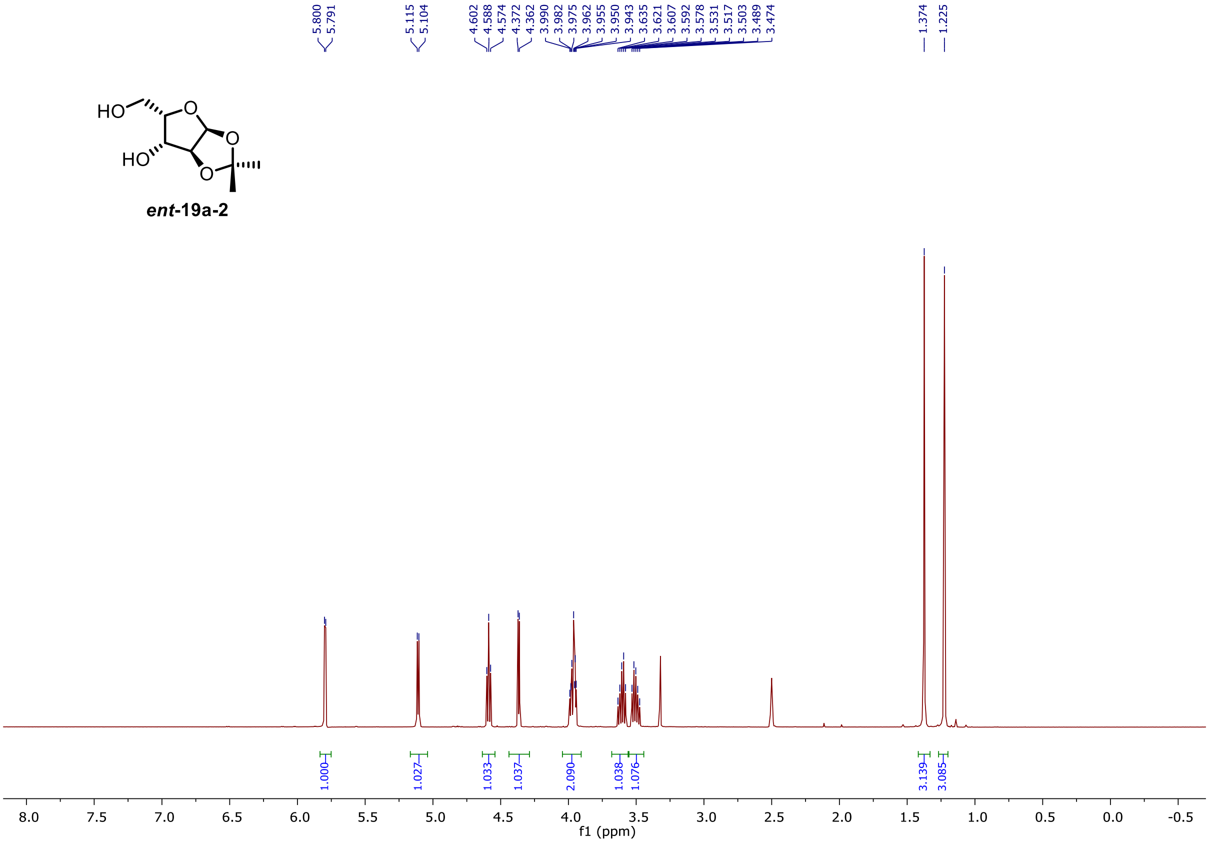

Supplementary Figure 33. ^{1}H spectra for ***ent*-19a-2**

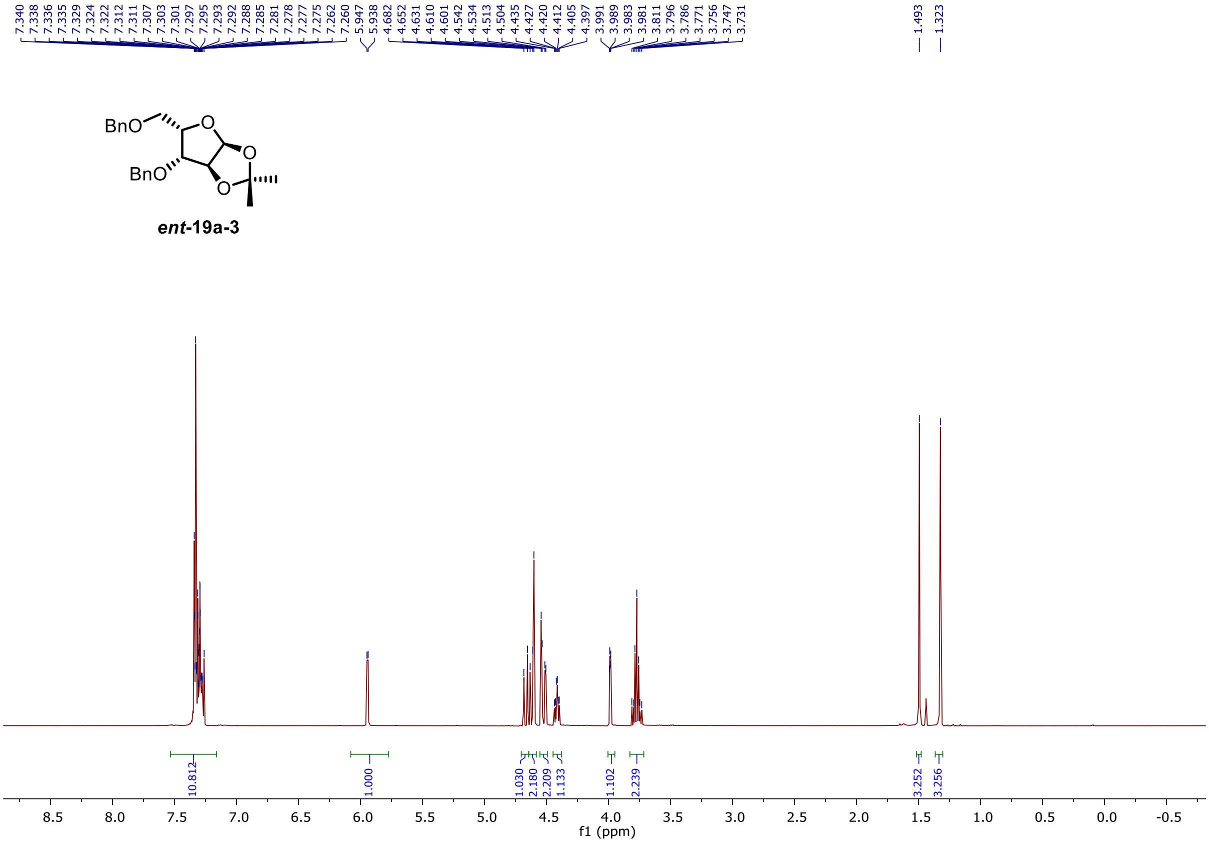

Supplementary Figure 34. ^{1}H spectra for ***ent*-19a-3**

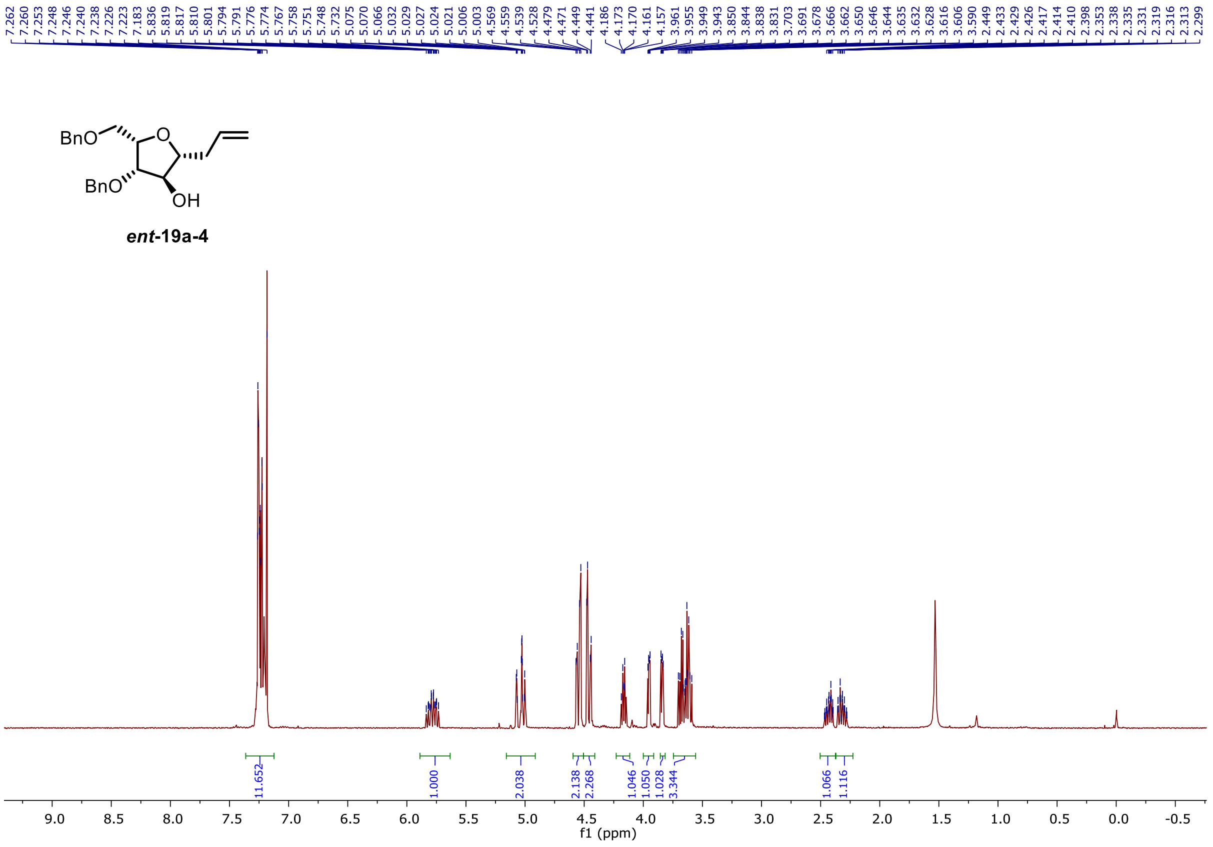

Supplementary Figure 35. ^{1}H spectra for ***ent*-19a-4**

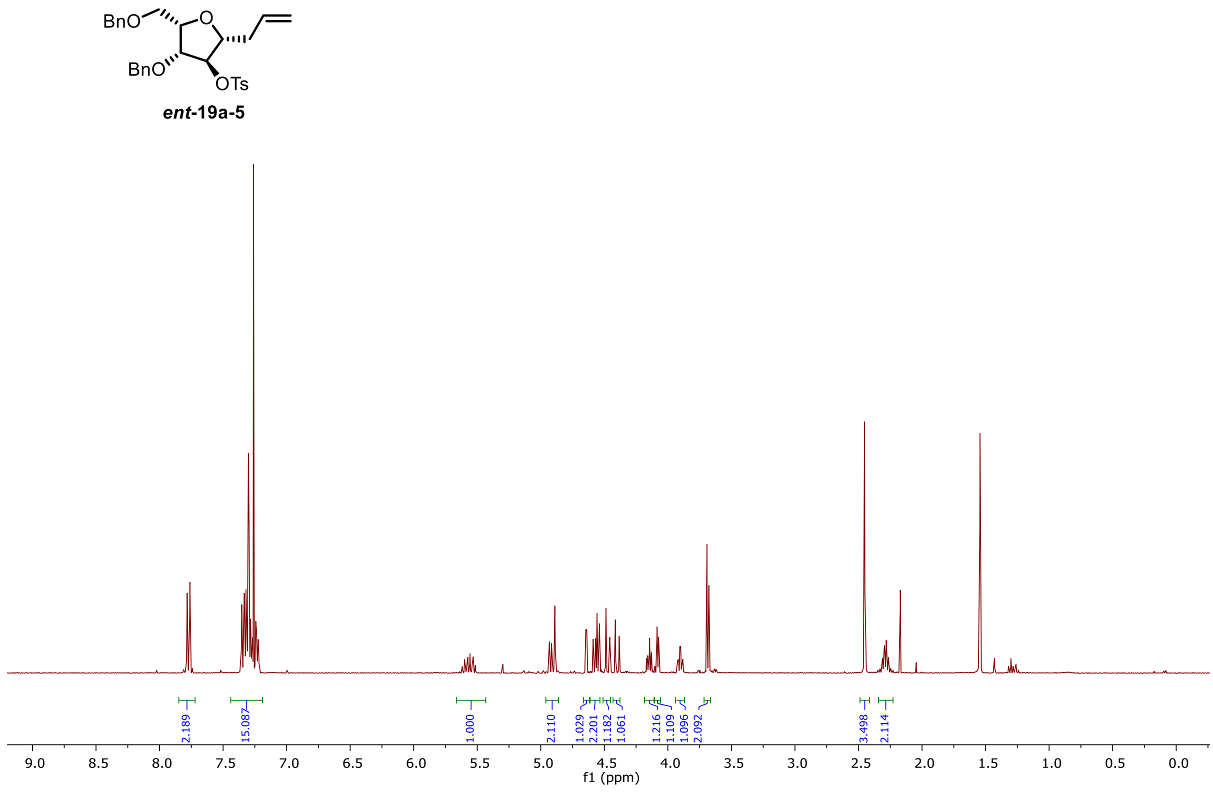

Supplementary Figure 36. ^{1}H spectra for ***ent*-19a-5**

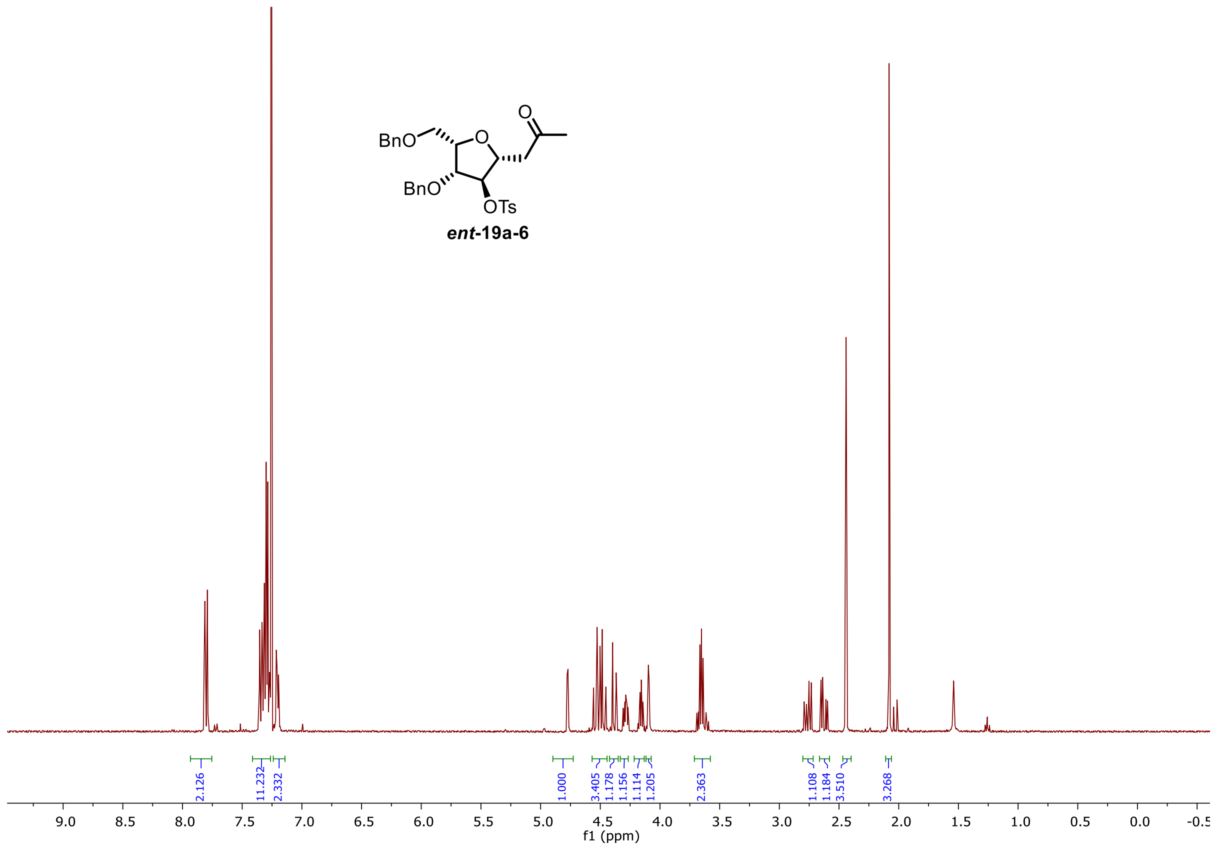

Supplementary Figure 37. ^{1}H spectra for ***ent*-19a-6**

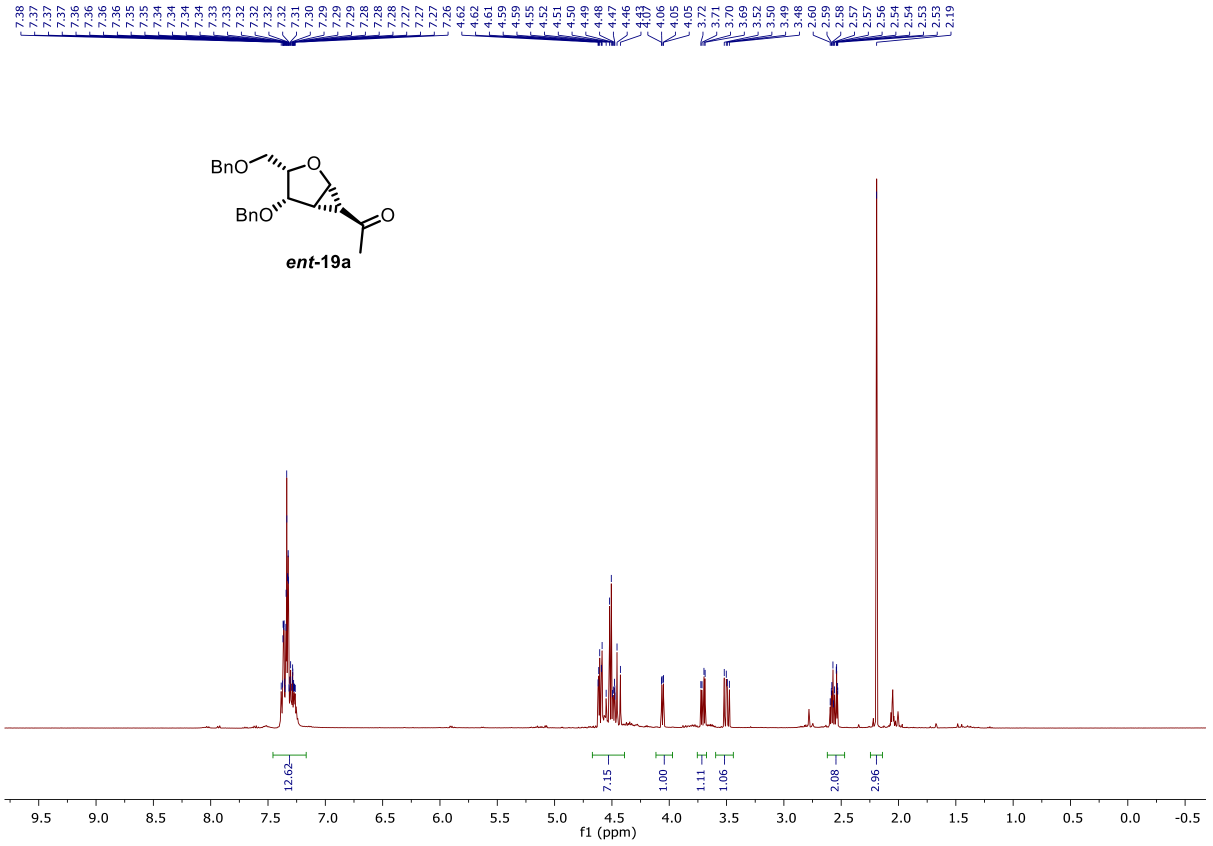

Supplementary Figure 38. ^{1}H spectra for ***ent*-19a**

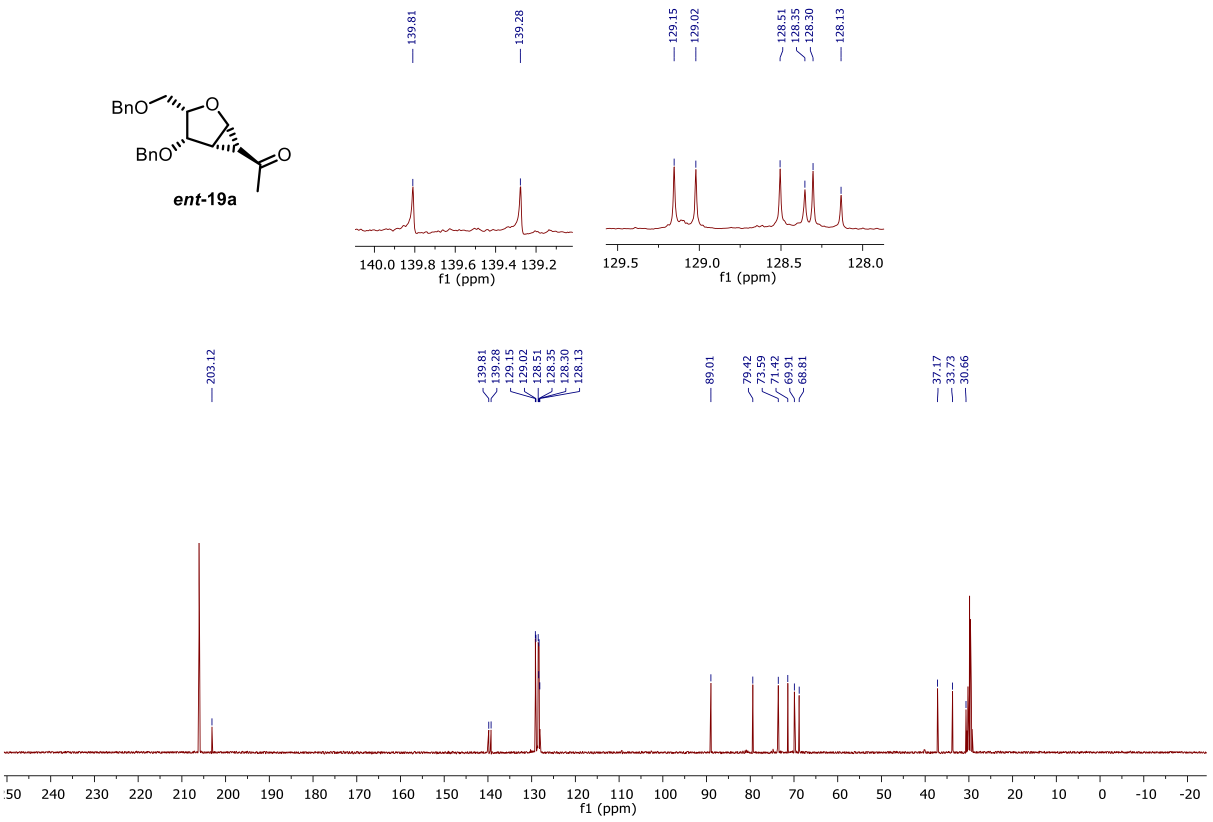

Supplementary Figure 39. ^{13}C spectra for ***ent*-19a**

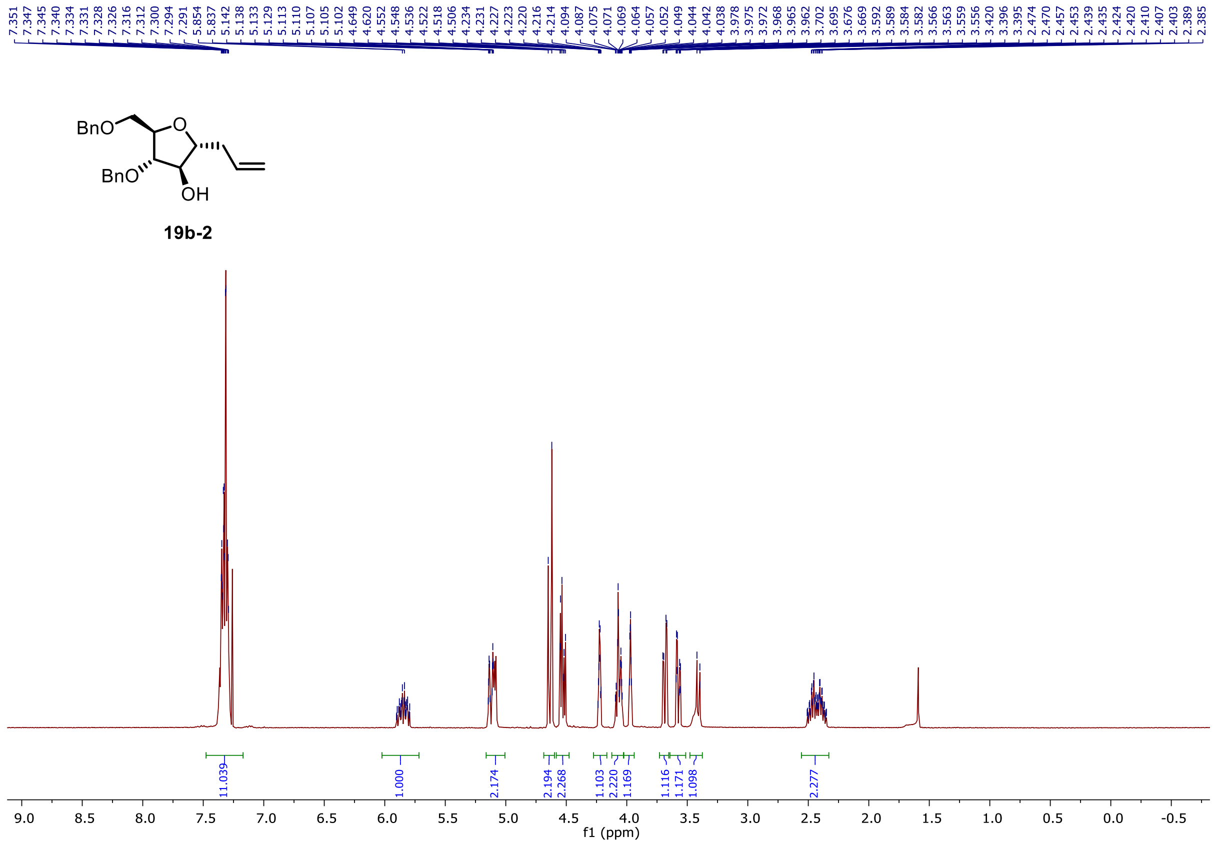

Supplementary Figure 40. ^{1}H spectra for **19b-2**

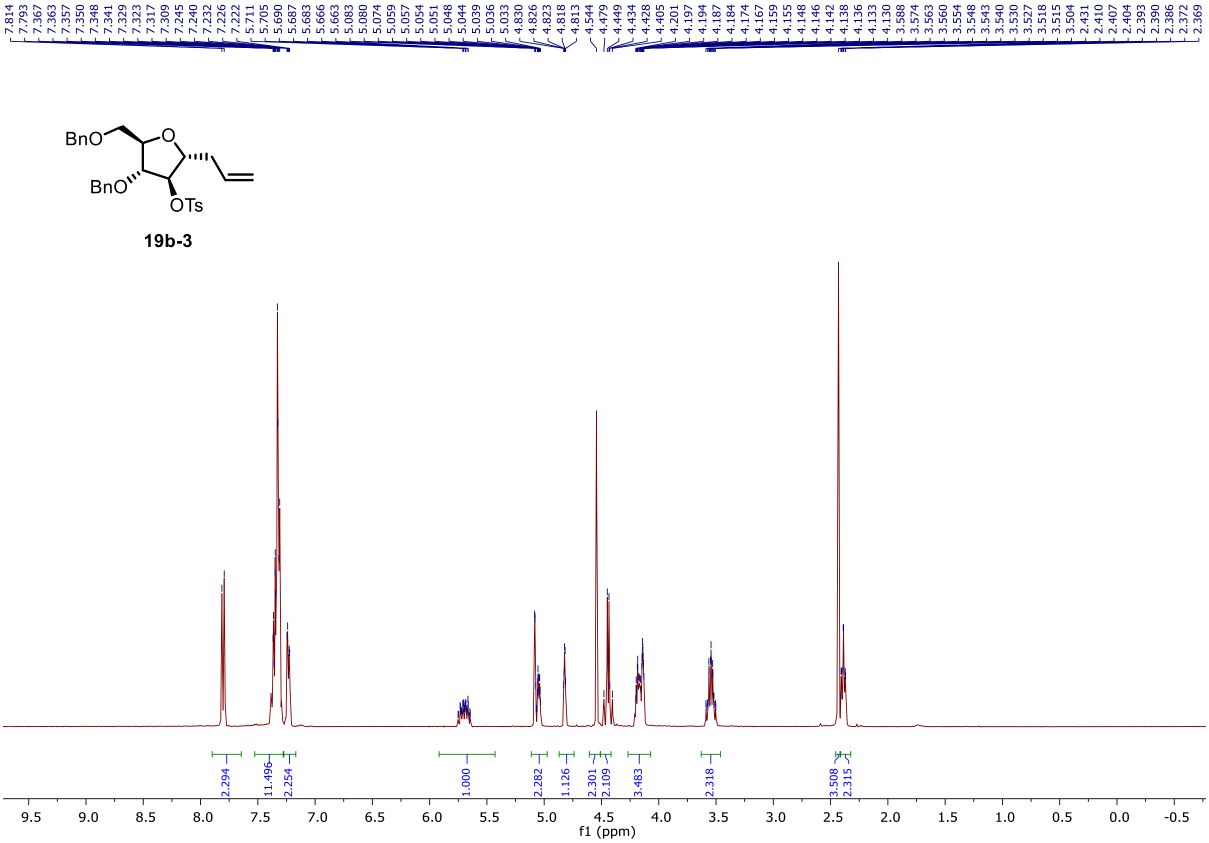

Supplementary Figure 41. ^{1}H spectra for **19b-3**

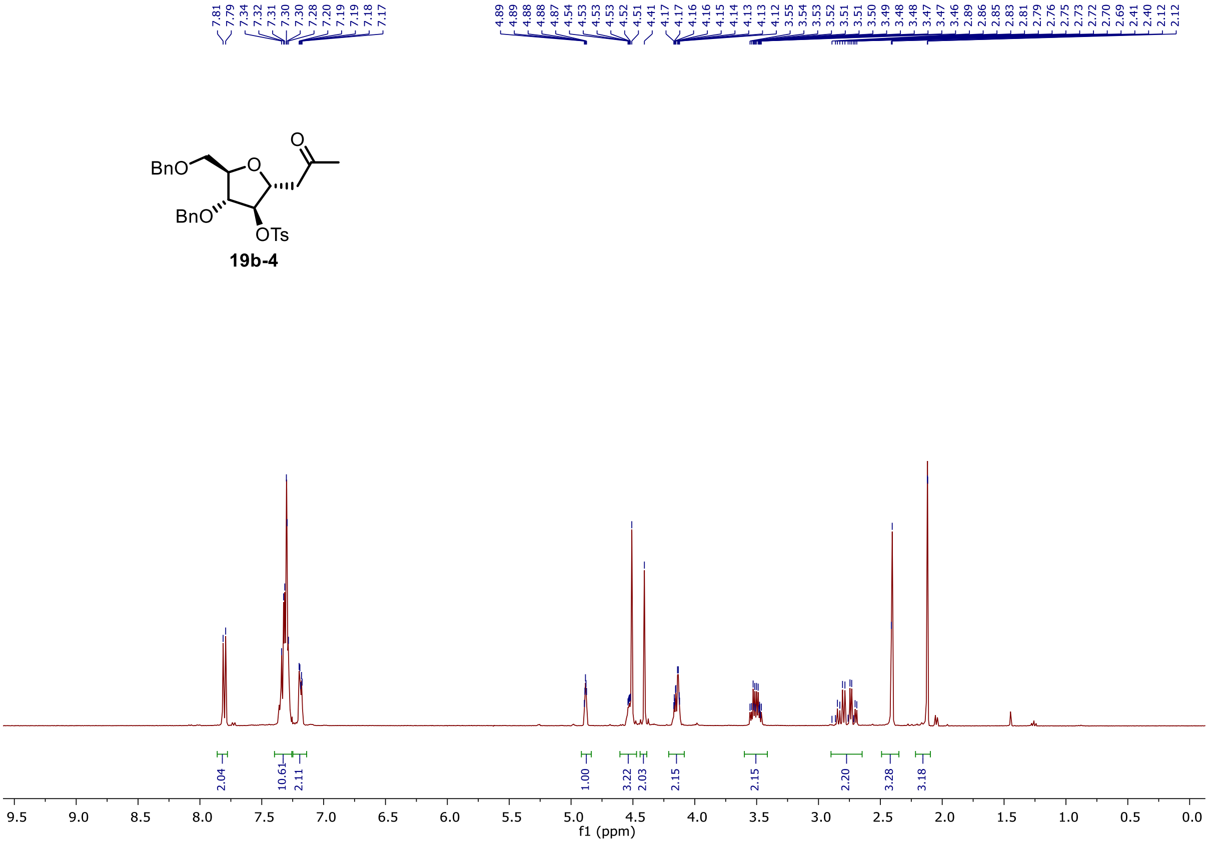

Supplementary Figure 42. ^{1}H spectra for **19b-4**

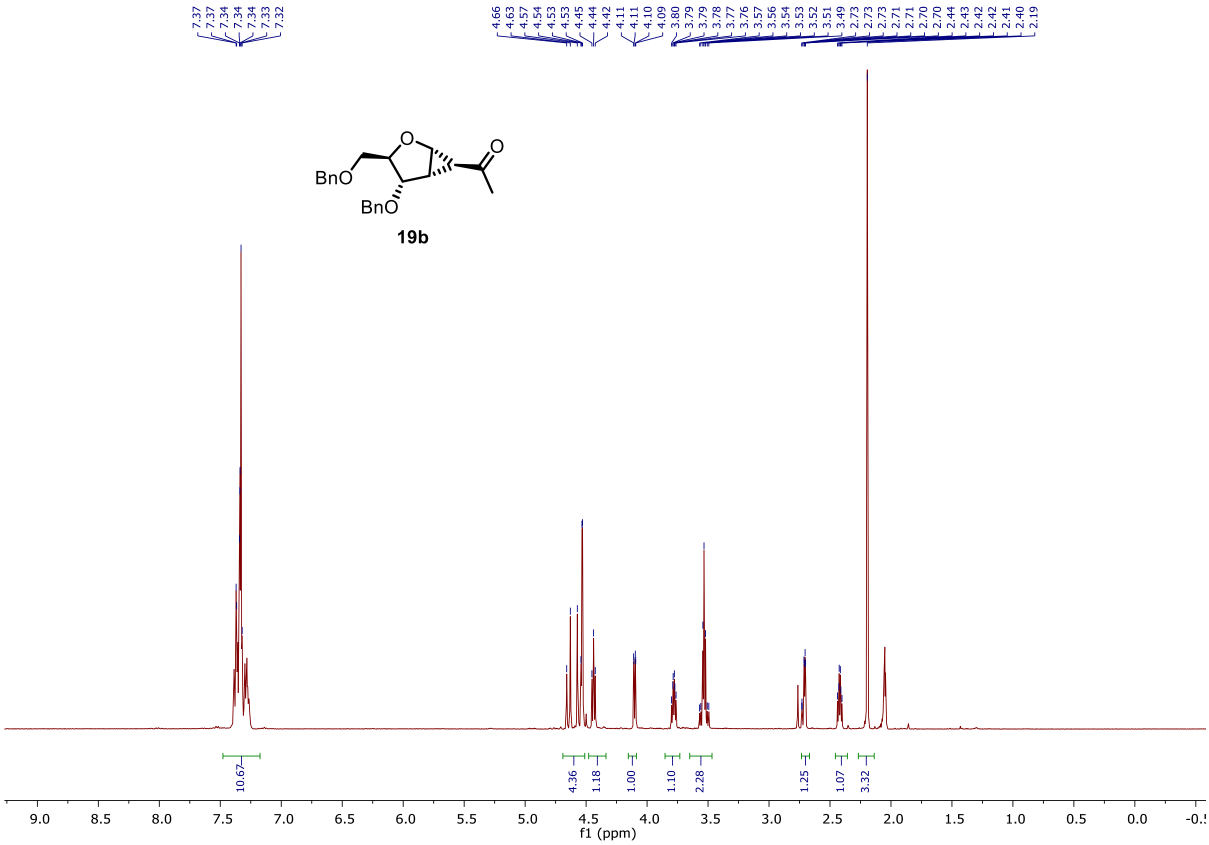

Supplementary Figure 43. ^{1}H spectra for **19b**

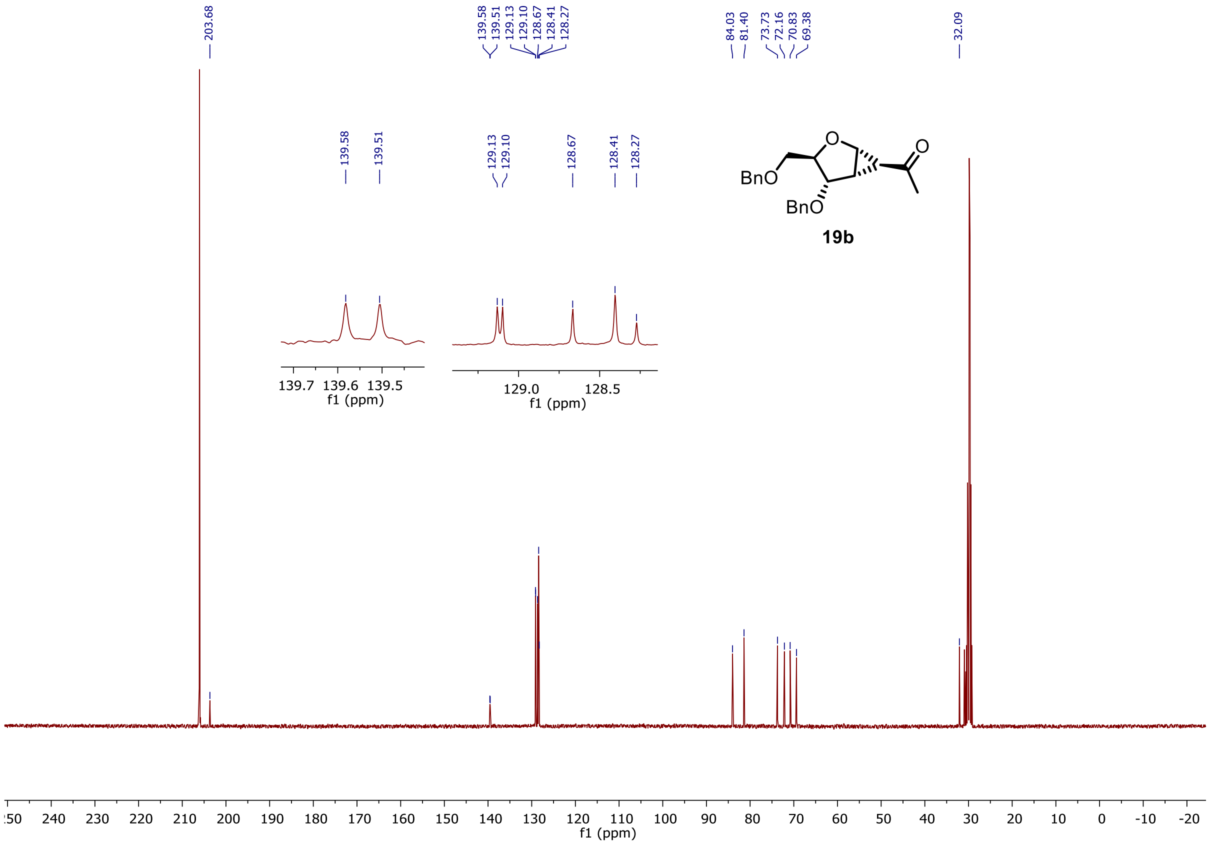

Supplementary Figure 44. ^{13}C spectra for **19b**

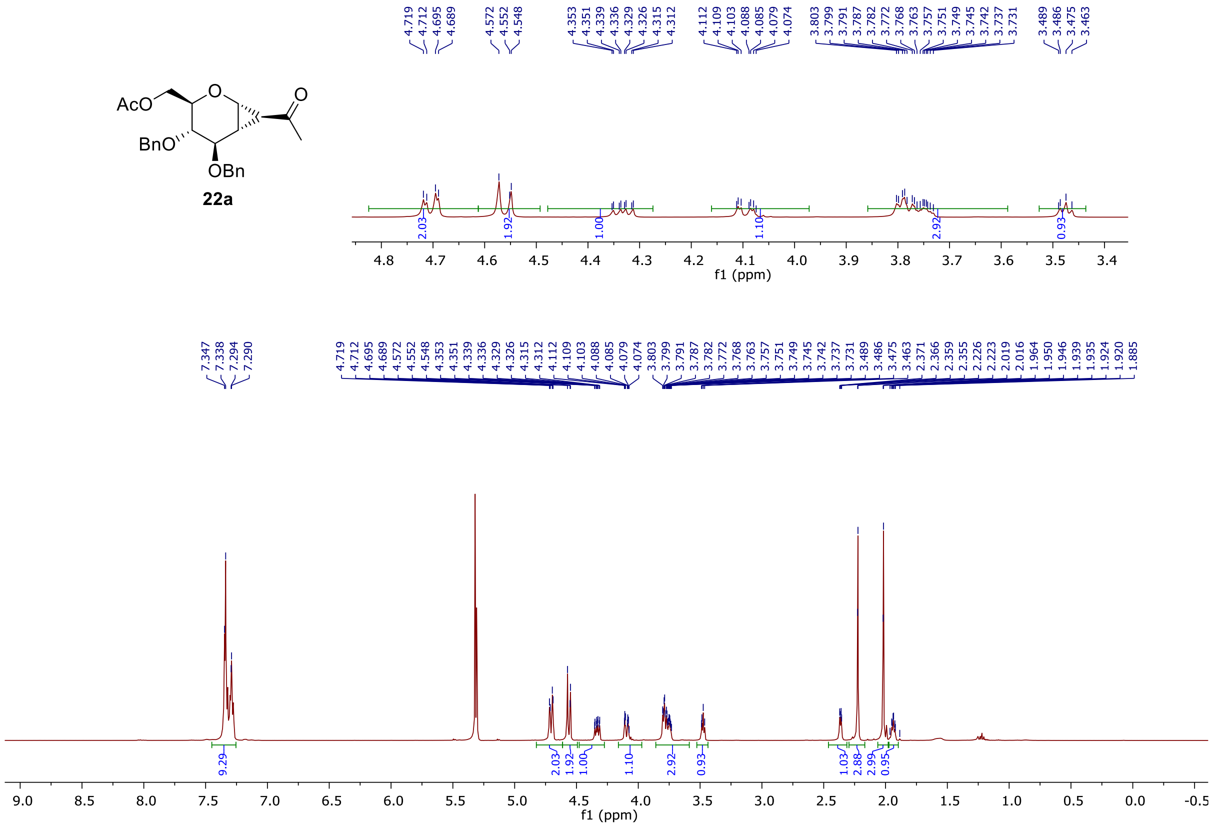

Supplementary Figure 45. ^{1}H spectra for **22a**

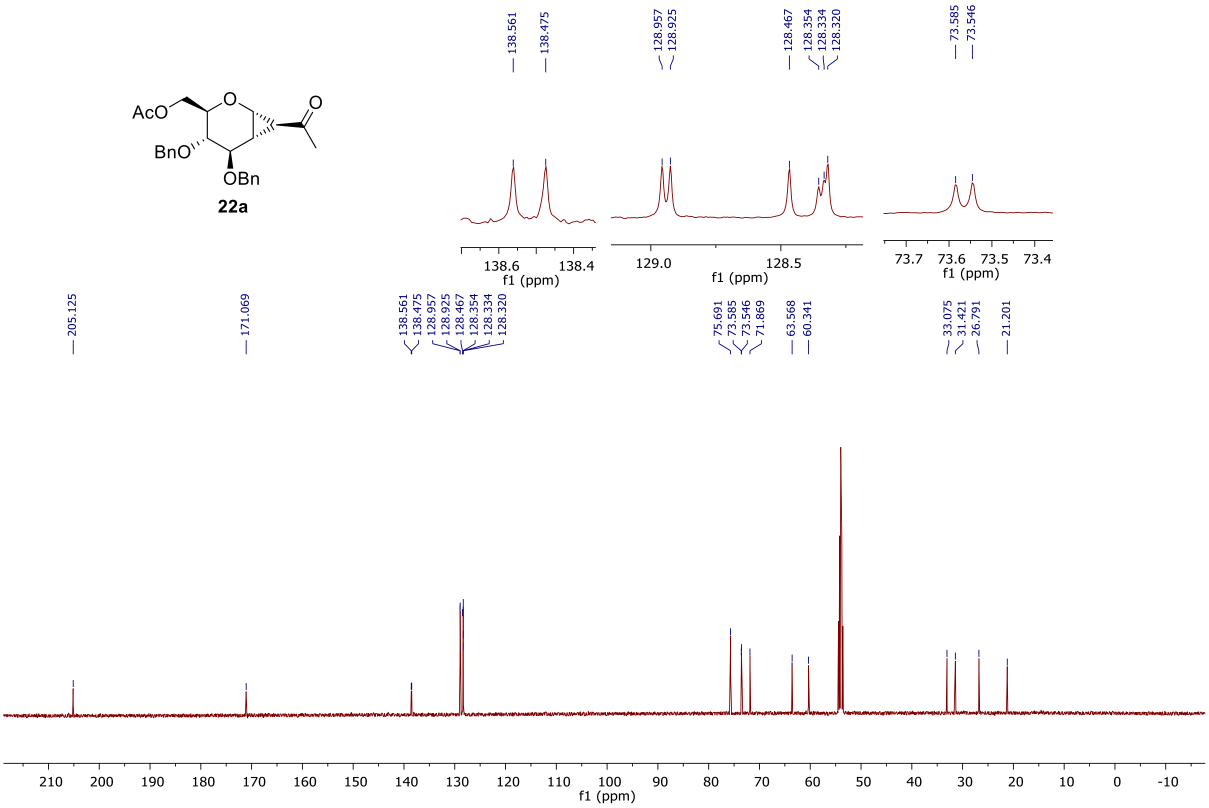

Supplementary Figure 46. ^{13}C spectra for **22a**

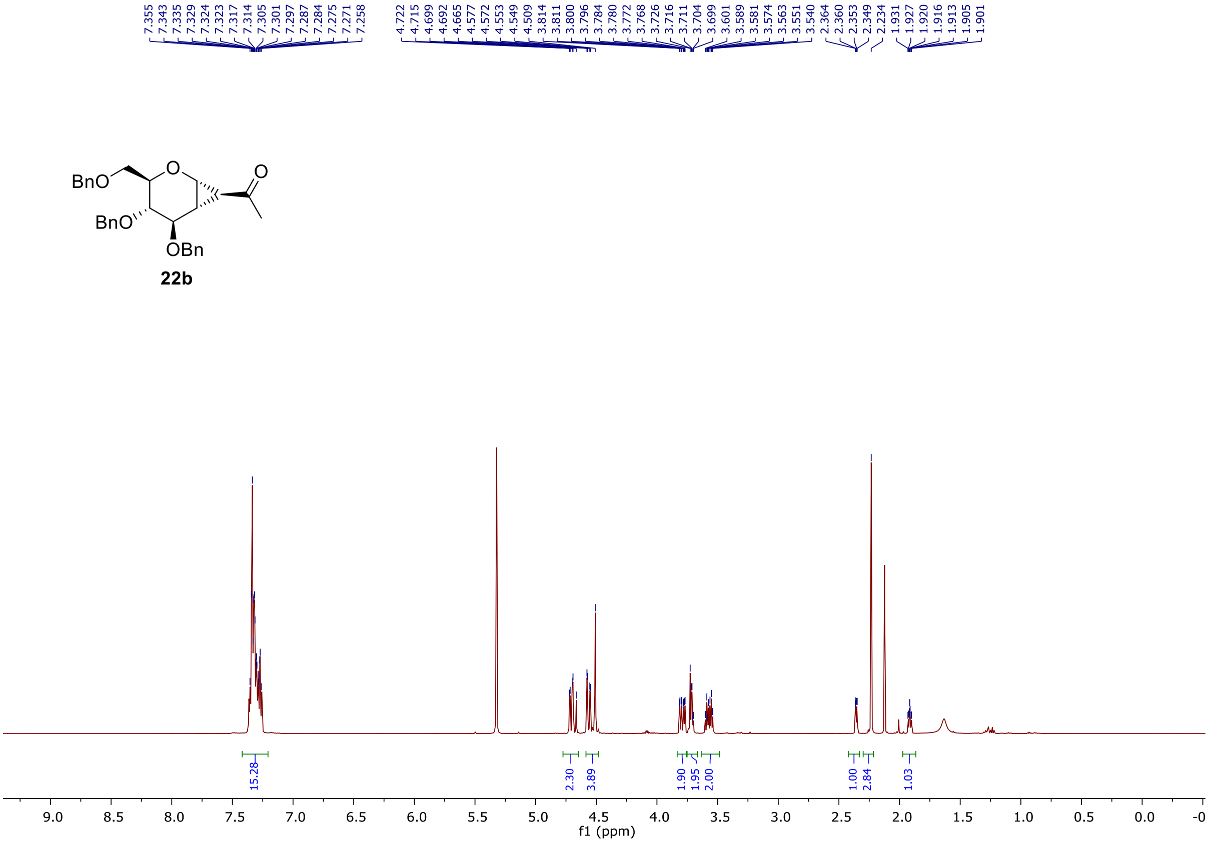

Supplementary Figure 47. ^{1}H spectra for **22b**

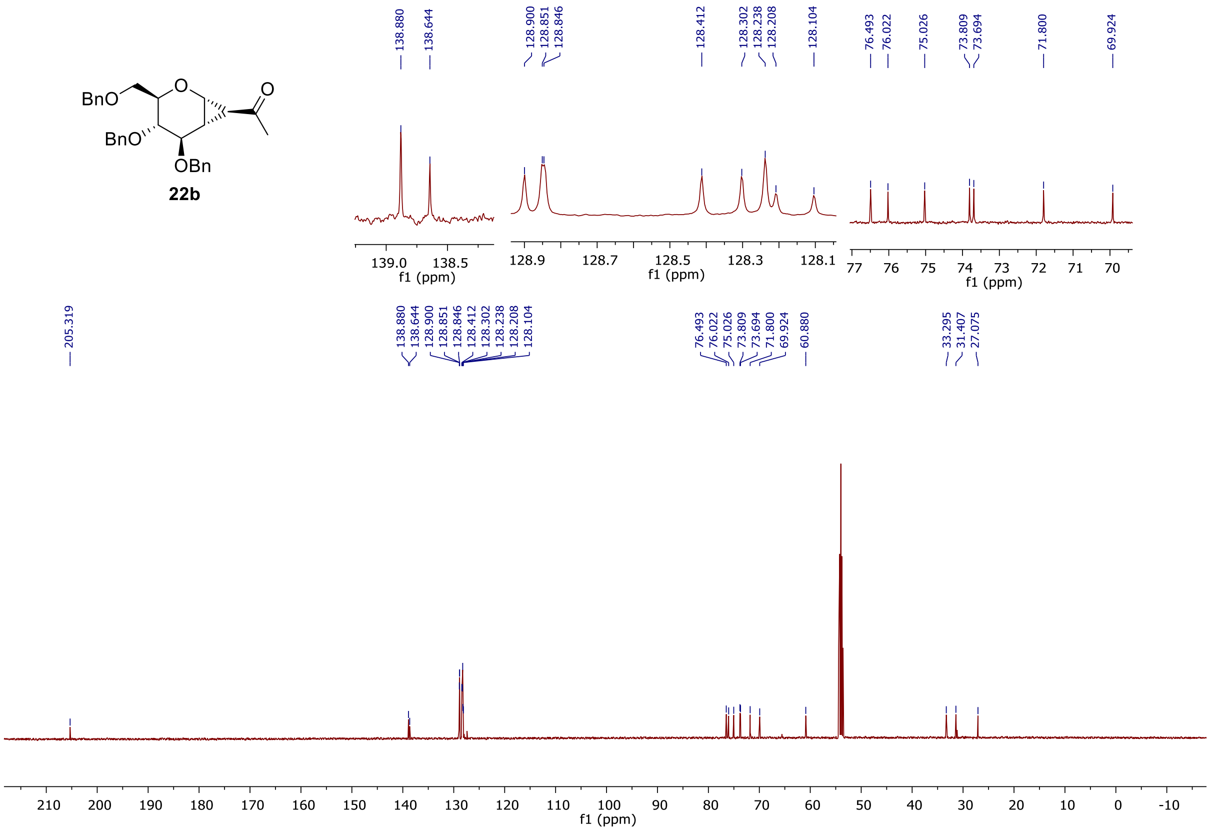

Supplementary Figure 48. ^{13}C spectra for **22b**

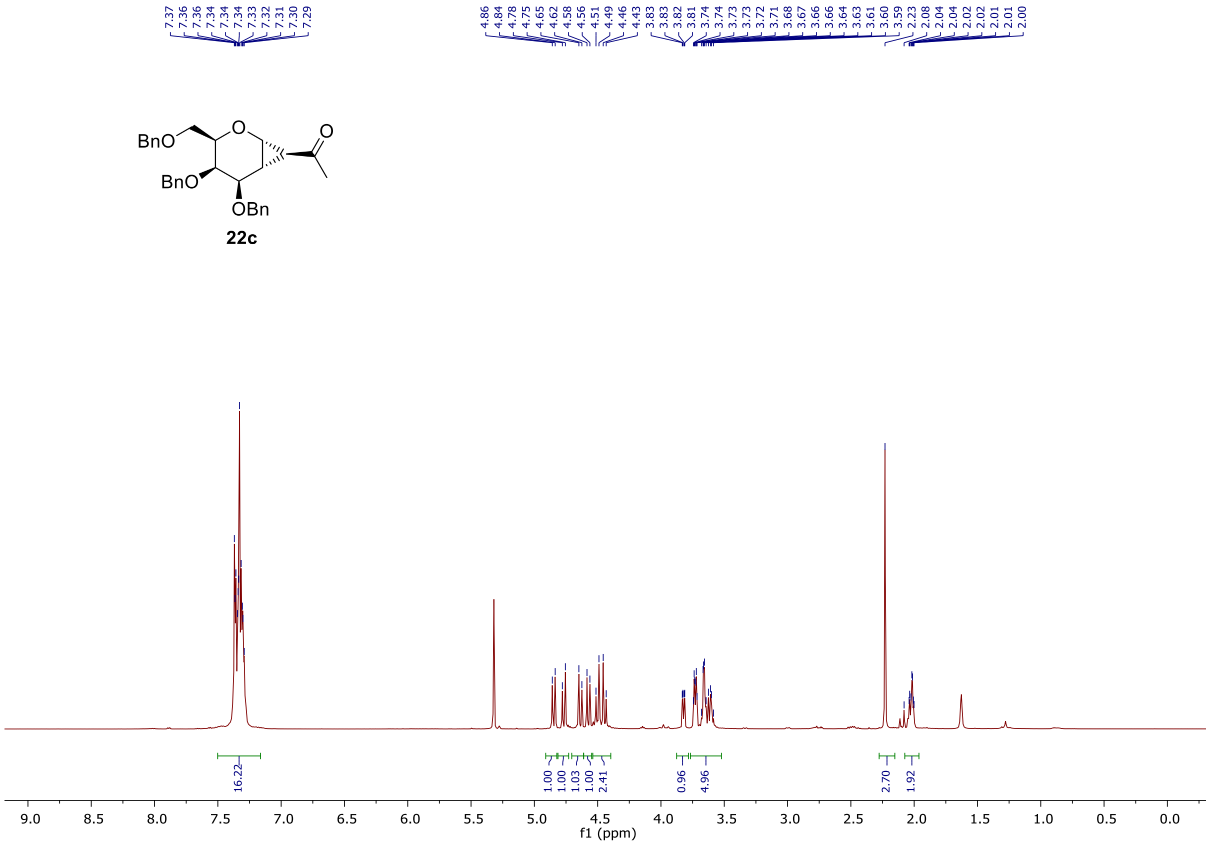

Supplementary Figure 49. ^{1}H spectra for **22c**

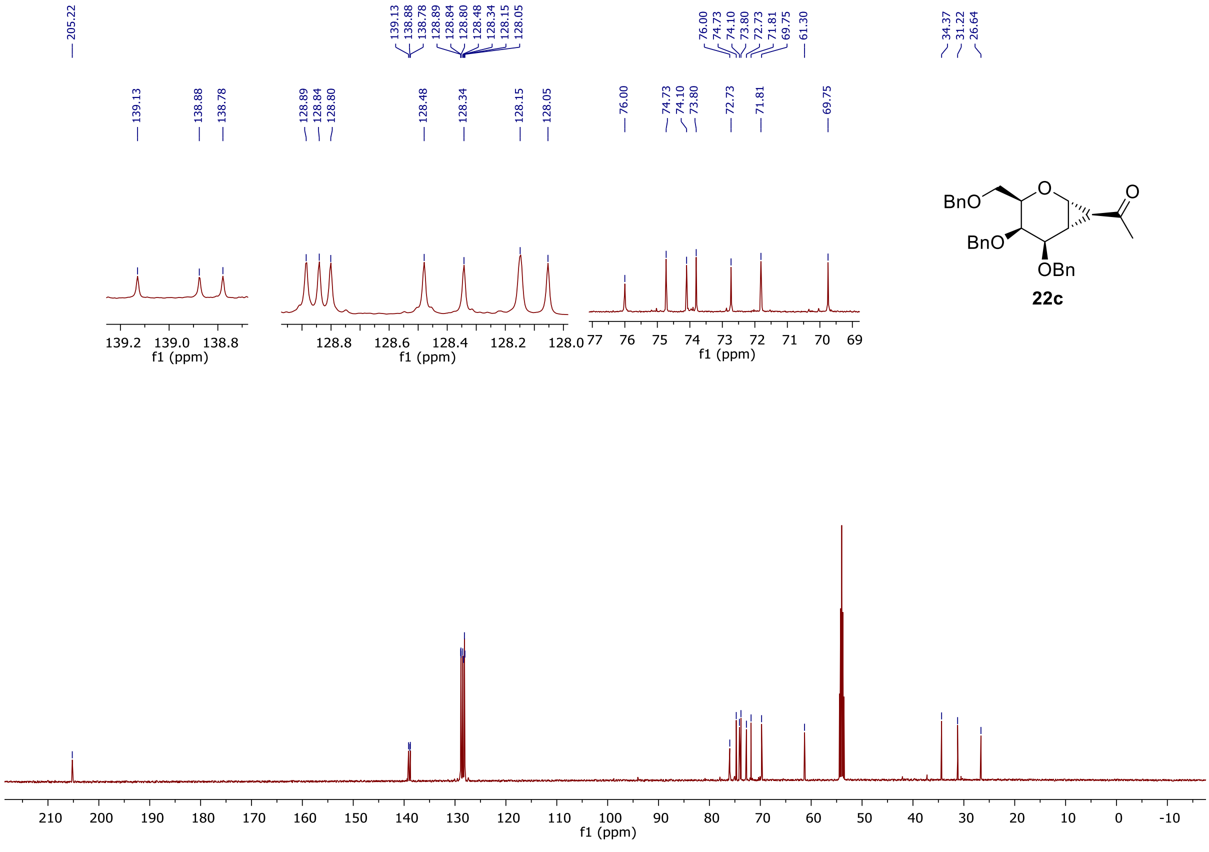

Supplementary Figure 50. ^{13}C spectra for **22c**

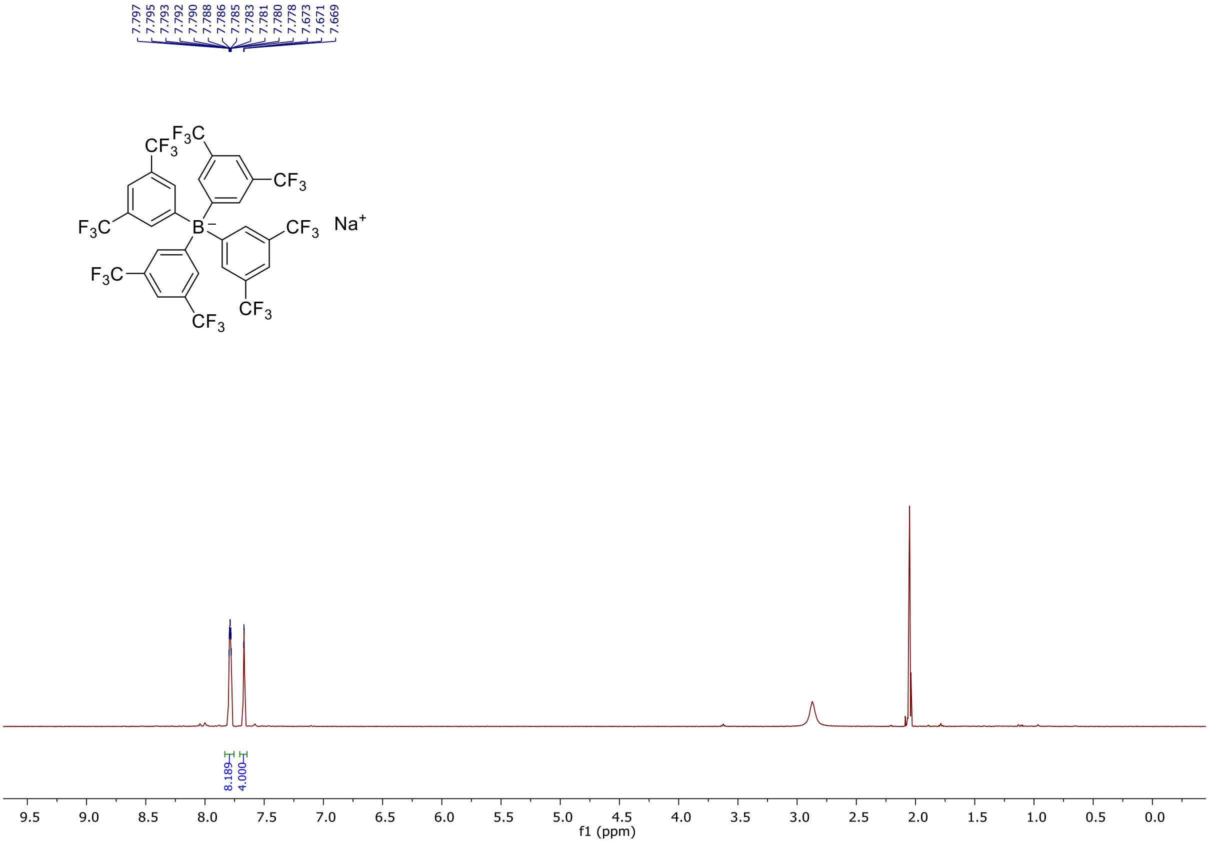

Supplementary Figure 51. ^{1}H spectra for **NaBArF**

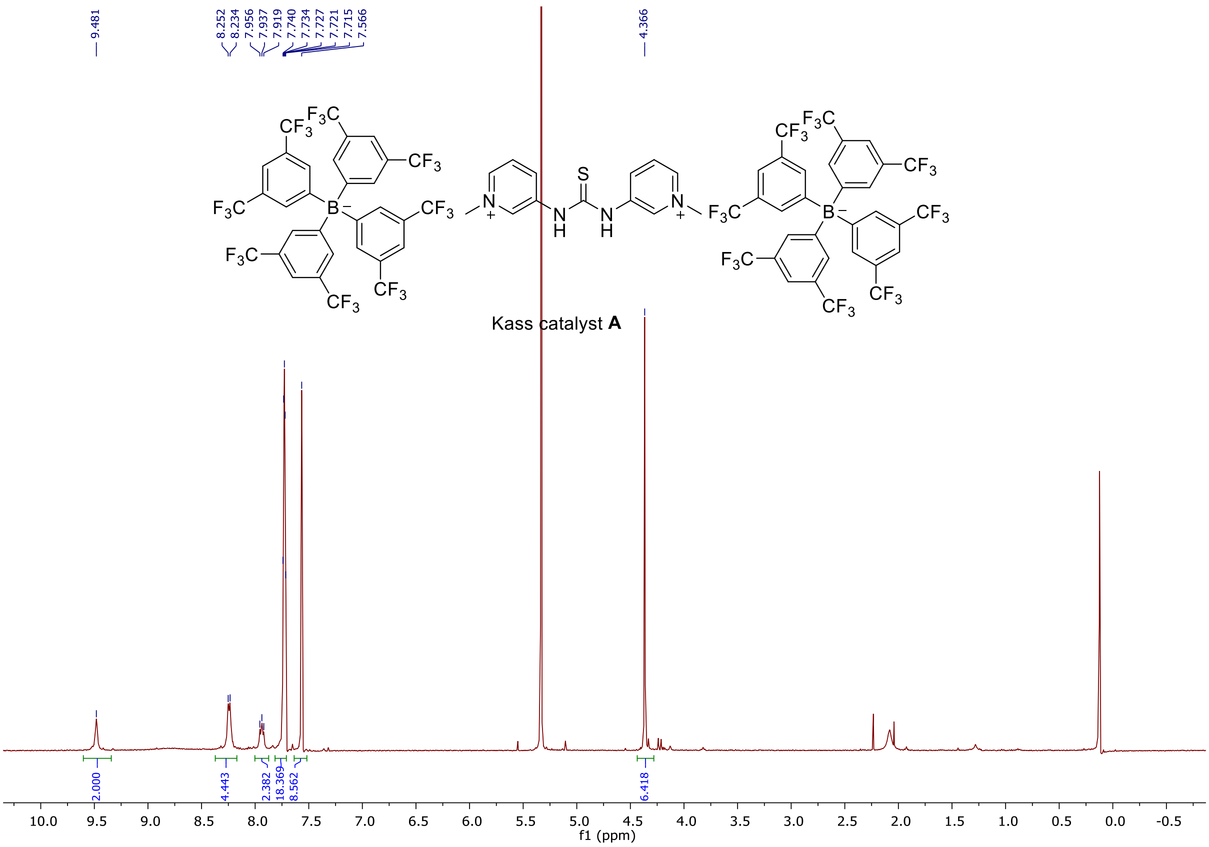

Supplementary Figure 52. ^{1}H spectra for Kass catalyst **A**

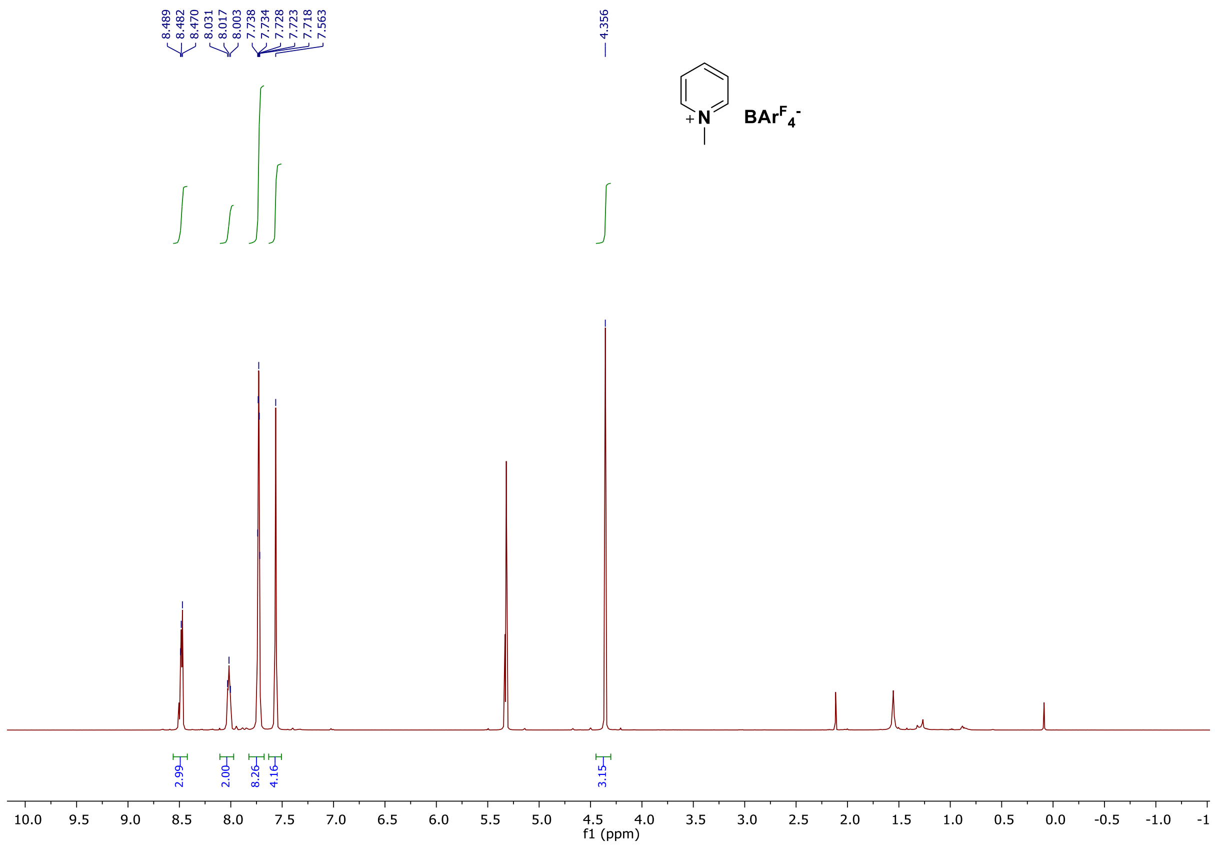

Supplementary Figure 53. ^{1}H spectra for compound **F**

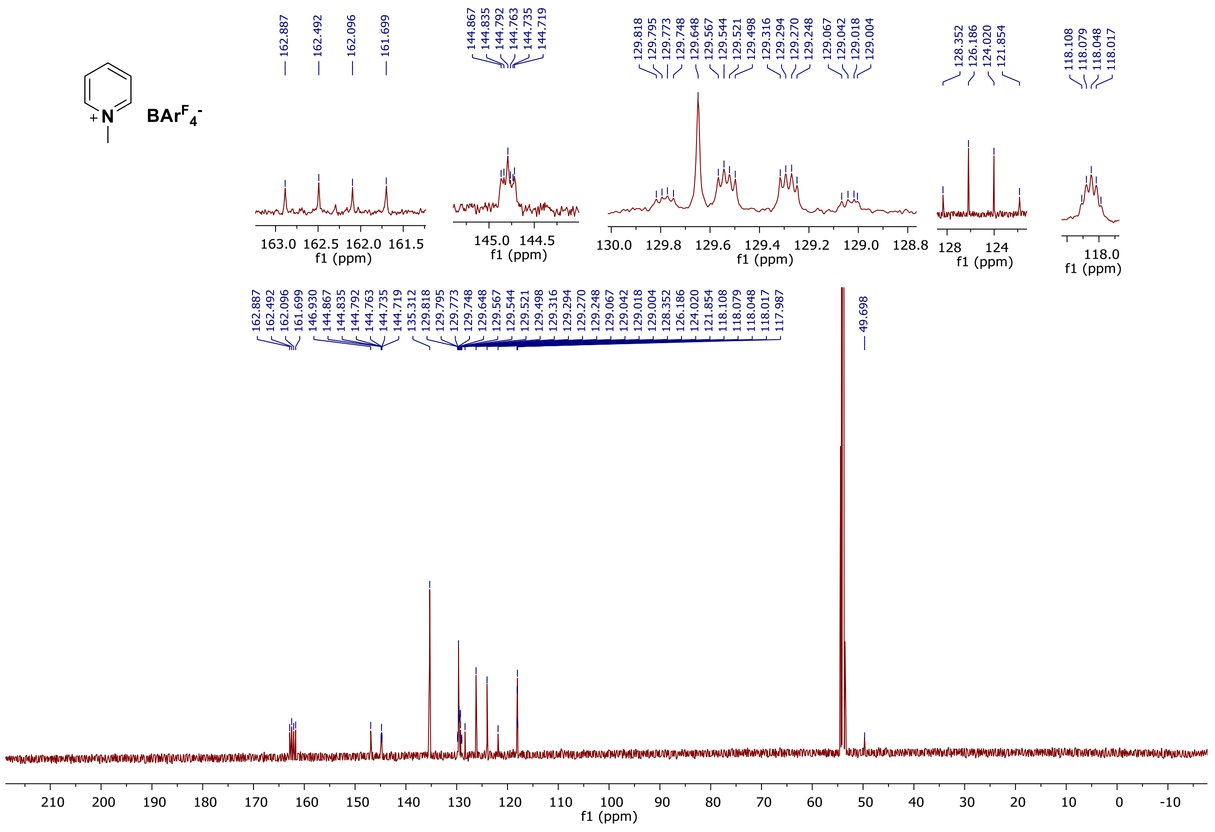

Supplementary Figure 54. ^{13}C spectra for compound **F**

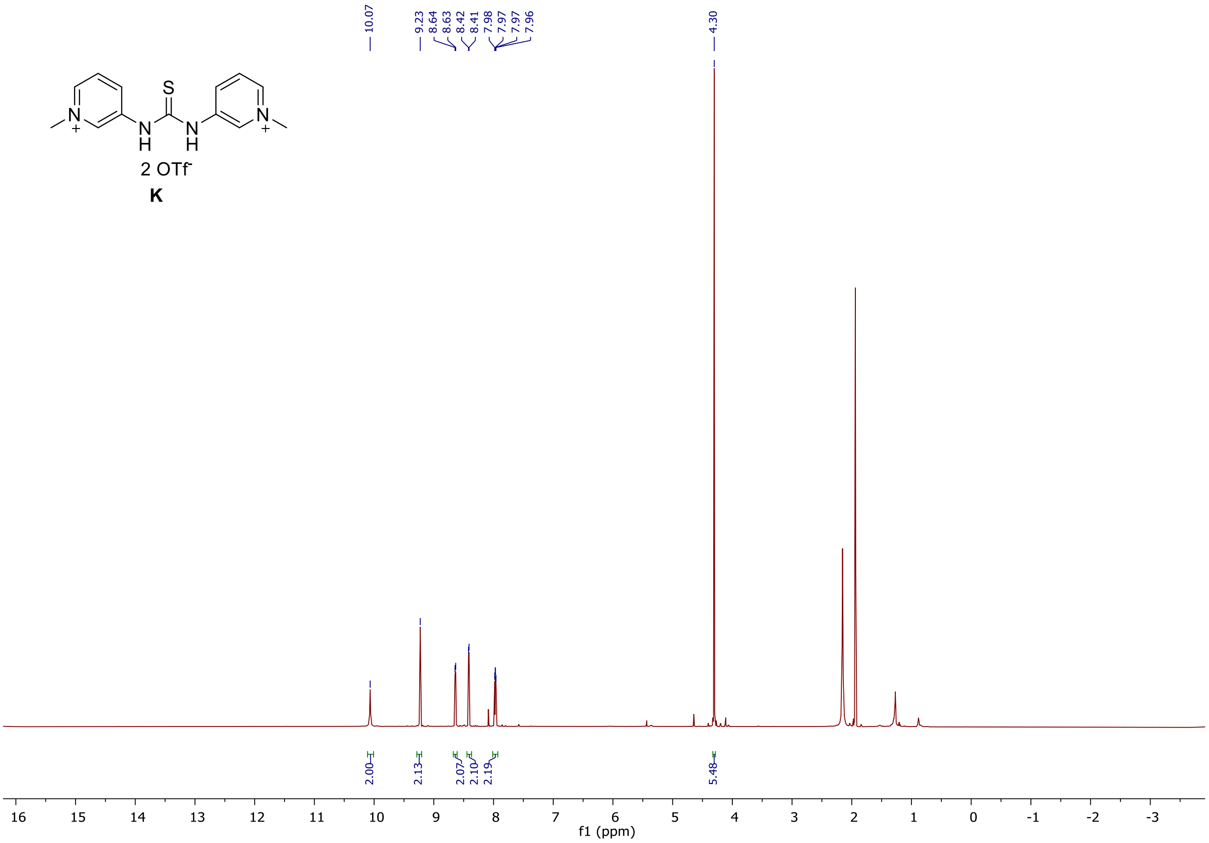

Supplementary Figure 55. ^{1}H spectra for compound **K**

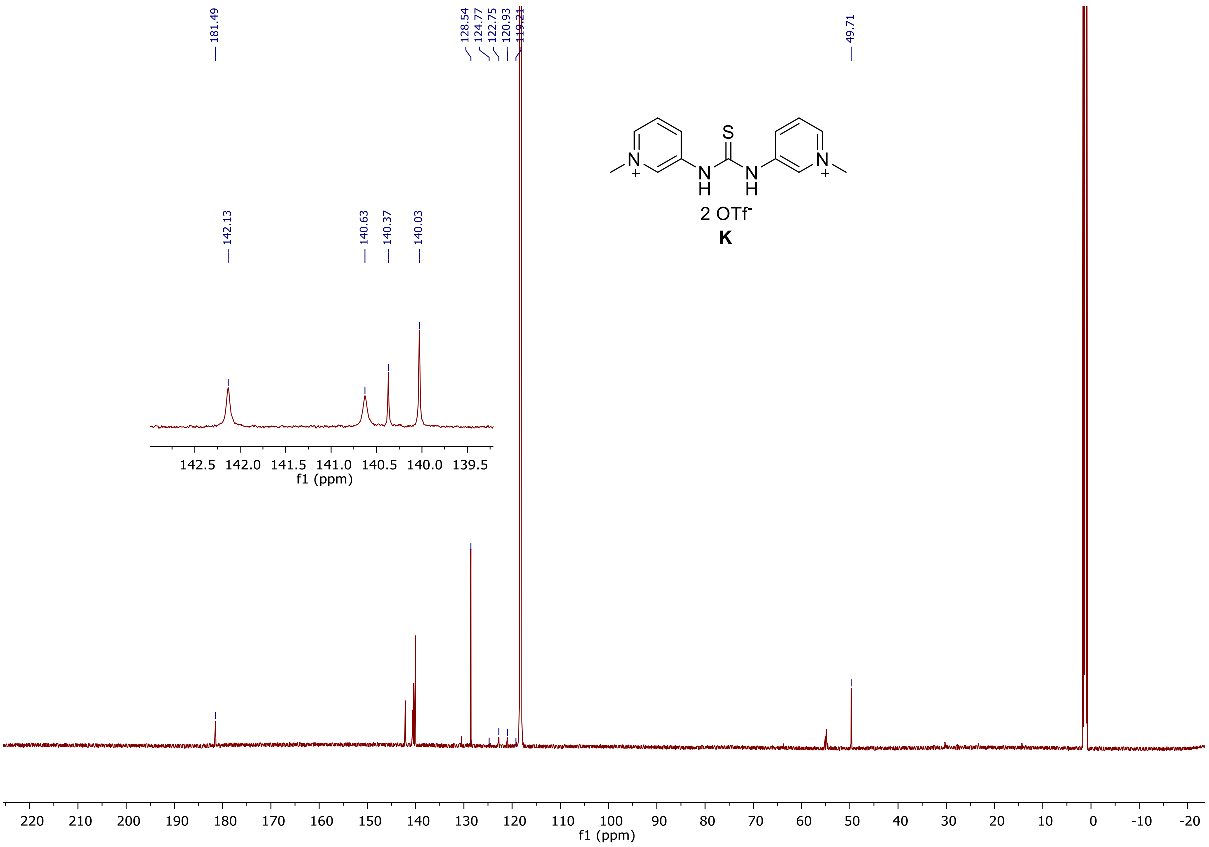

Supplementary Figure 56. ^{13}C spectra for compound **K**

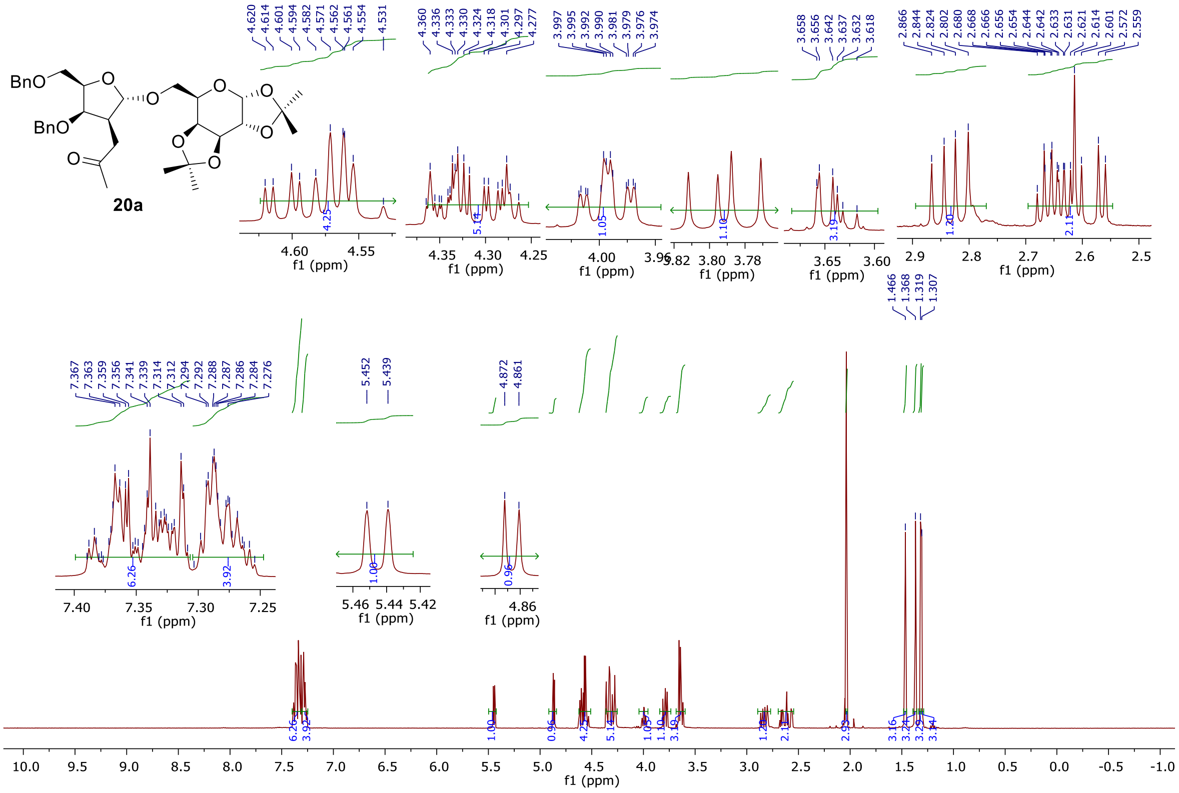

Supplementary Figure 57. ^{1}H spectra for **20a**

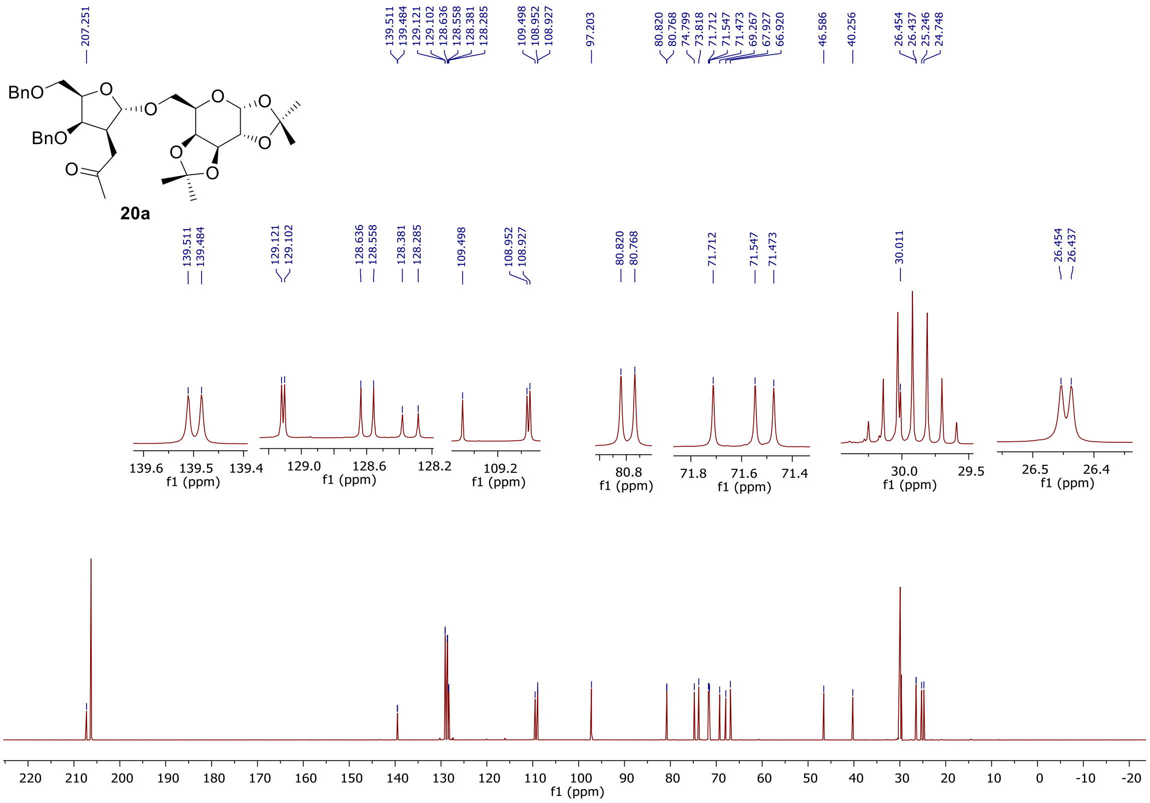

Supplementary Figure 58. ^{13}C spectra for **20a**

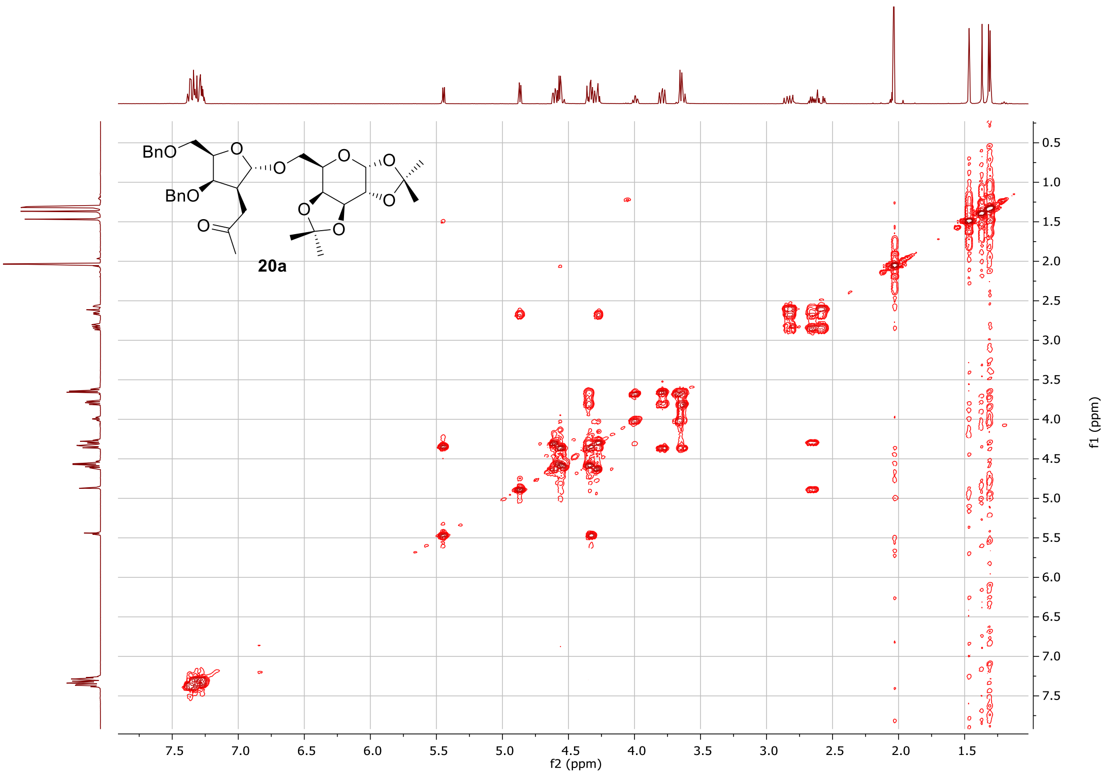

Supplementary Figure 59. COSY spectra for **20a**

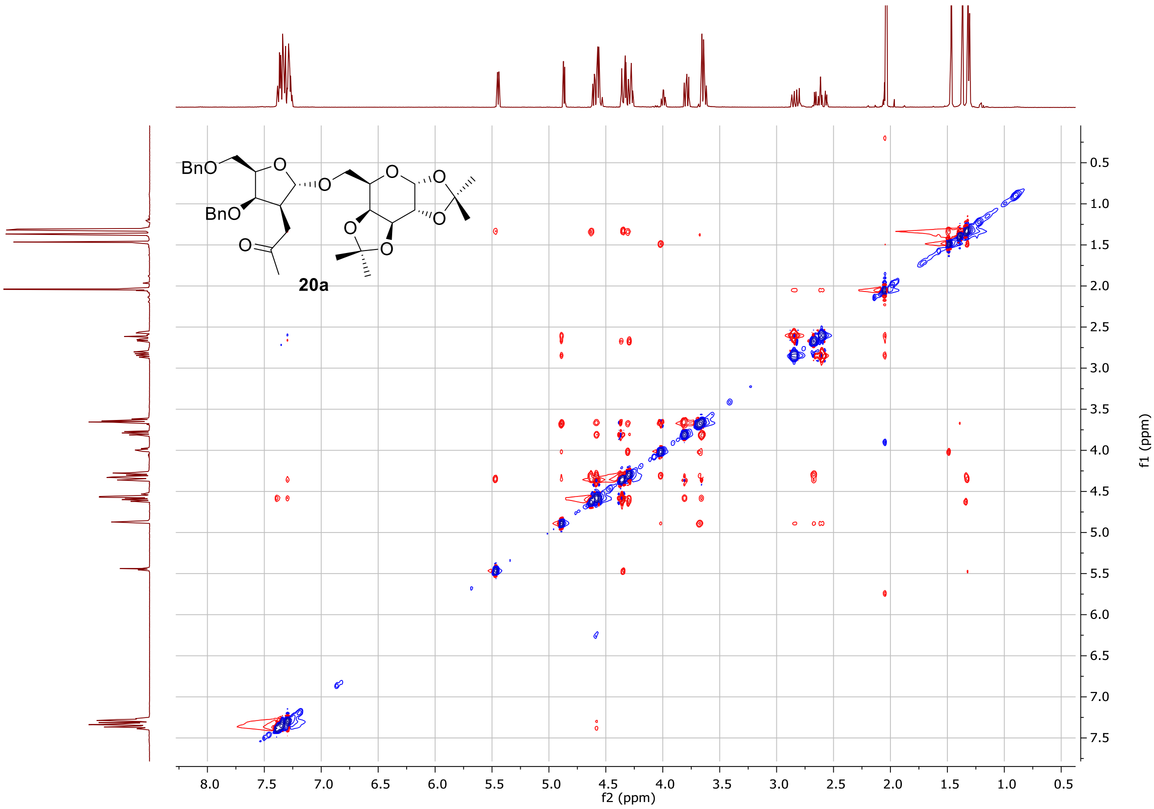

Supplementary Figure 60. NOESY spectra for **20a**

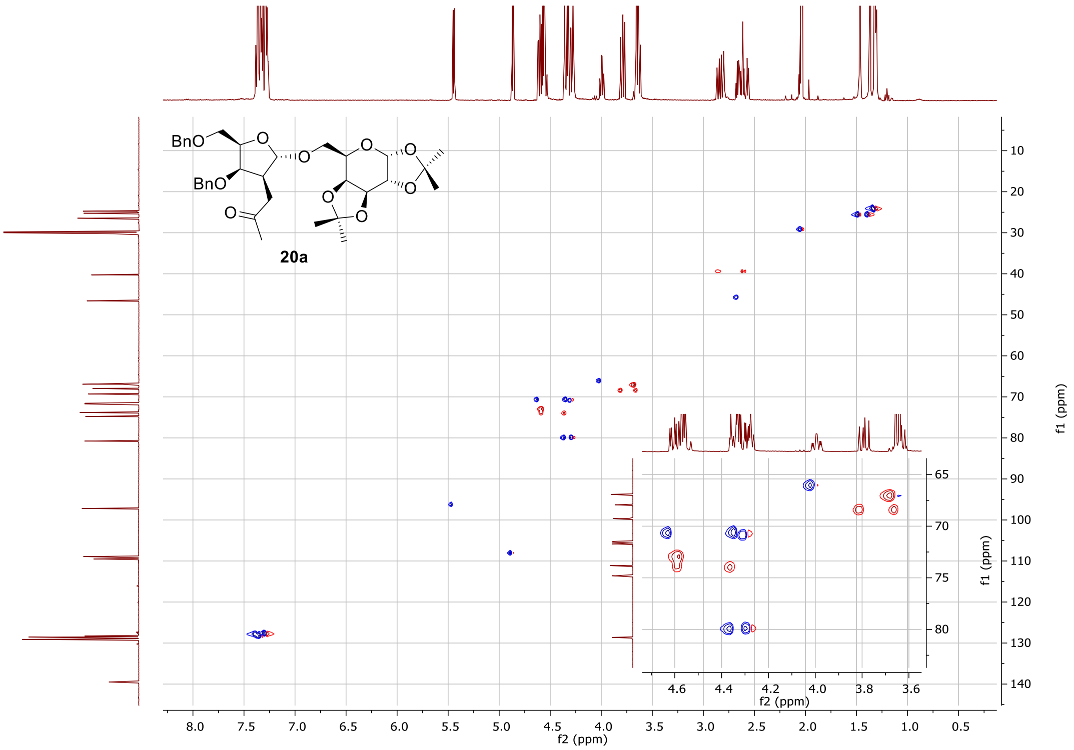

Supplementary Figure 61. HSQC spectra for **20a**

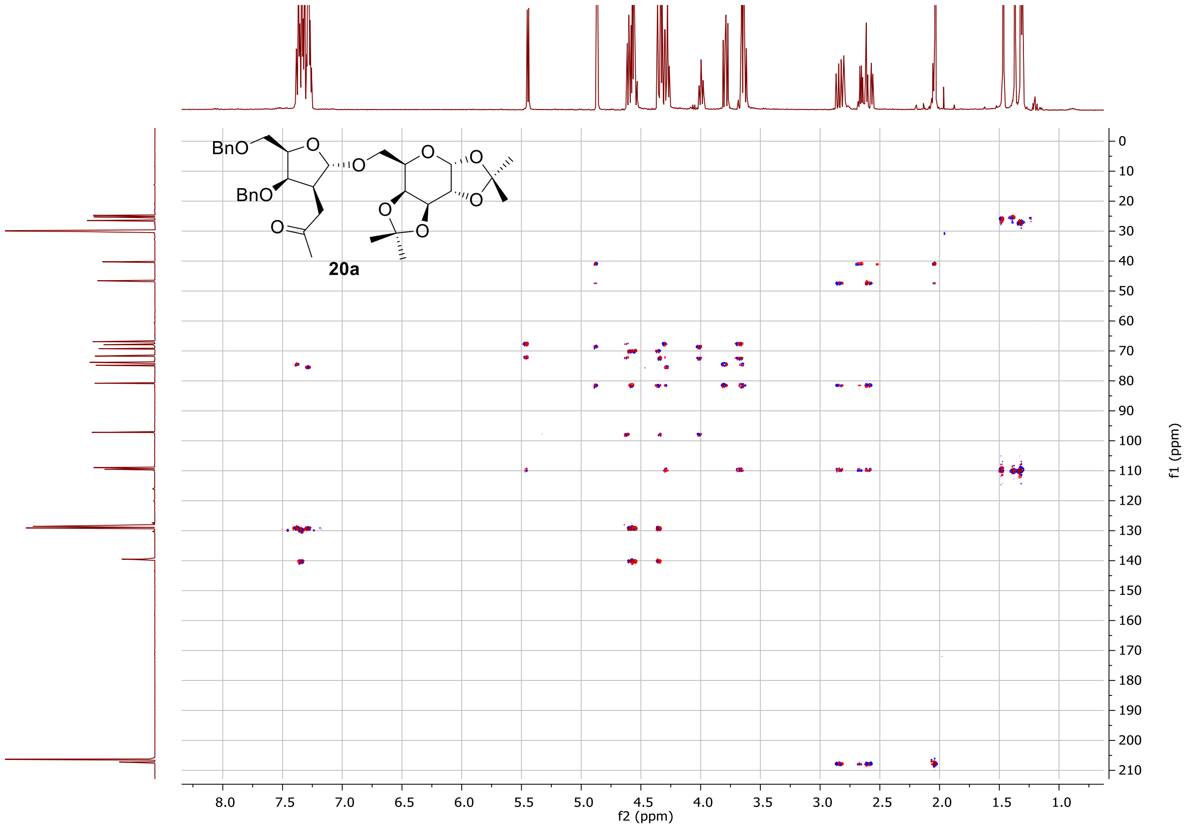

Supplementary Figure 62. HMBC spectra for **20a**

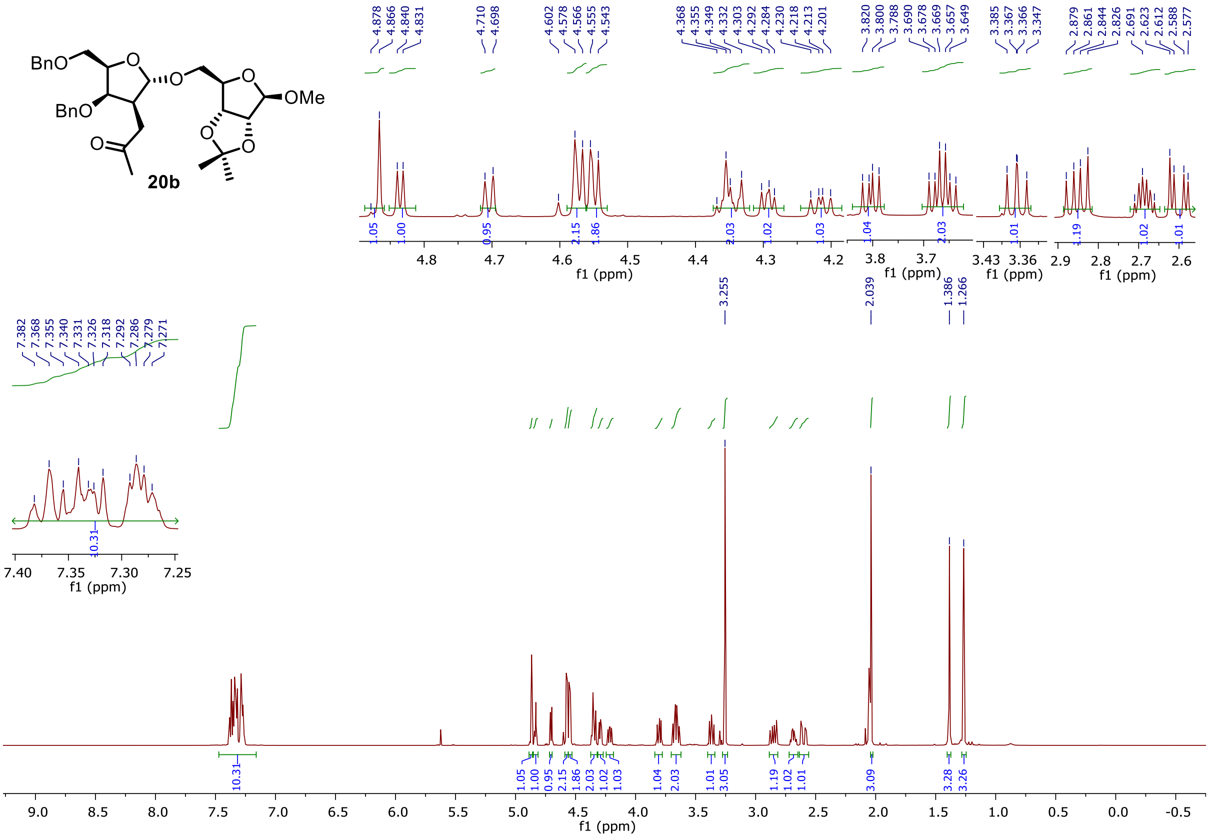

Supplementary Figure 63. ^{1}H spectra for **20b**

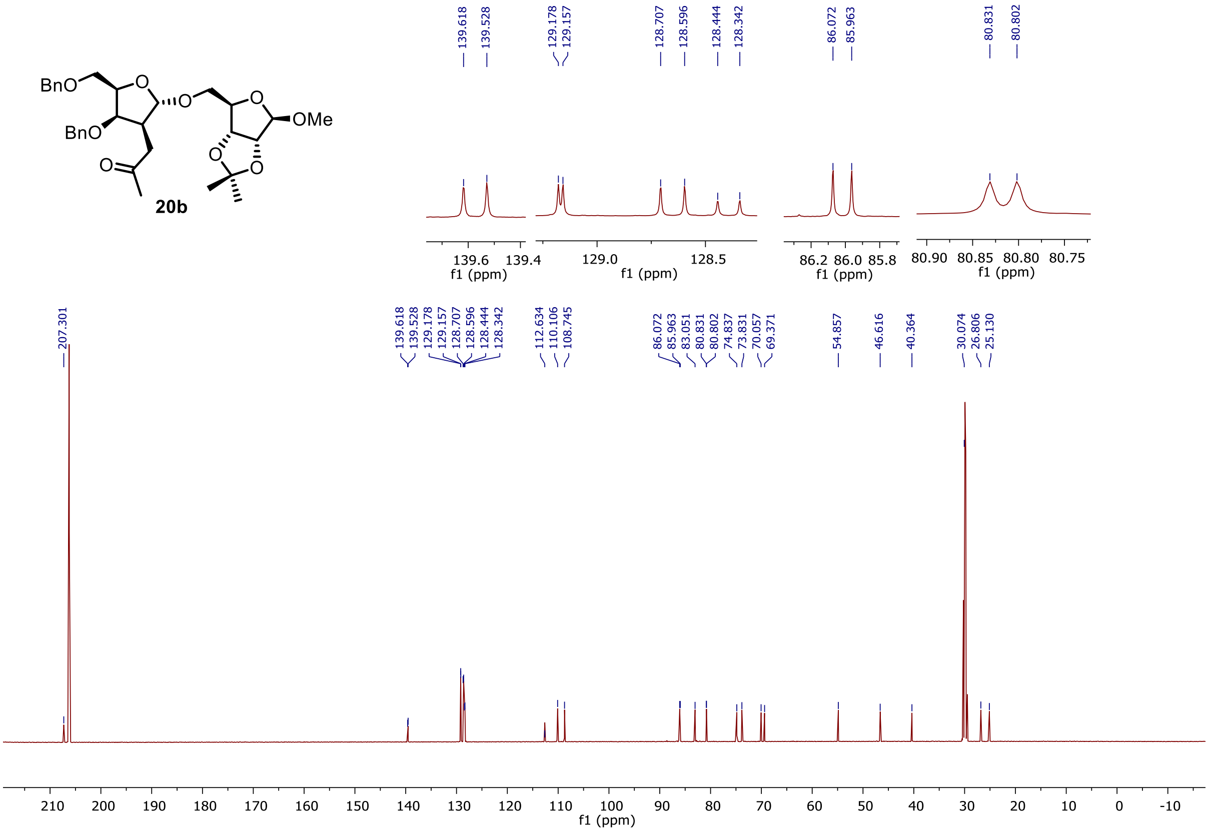

Supplementary Figure 64. ^{13}C spectra for **20b**

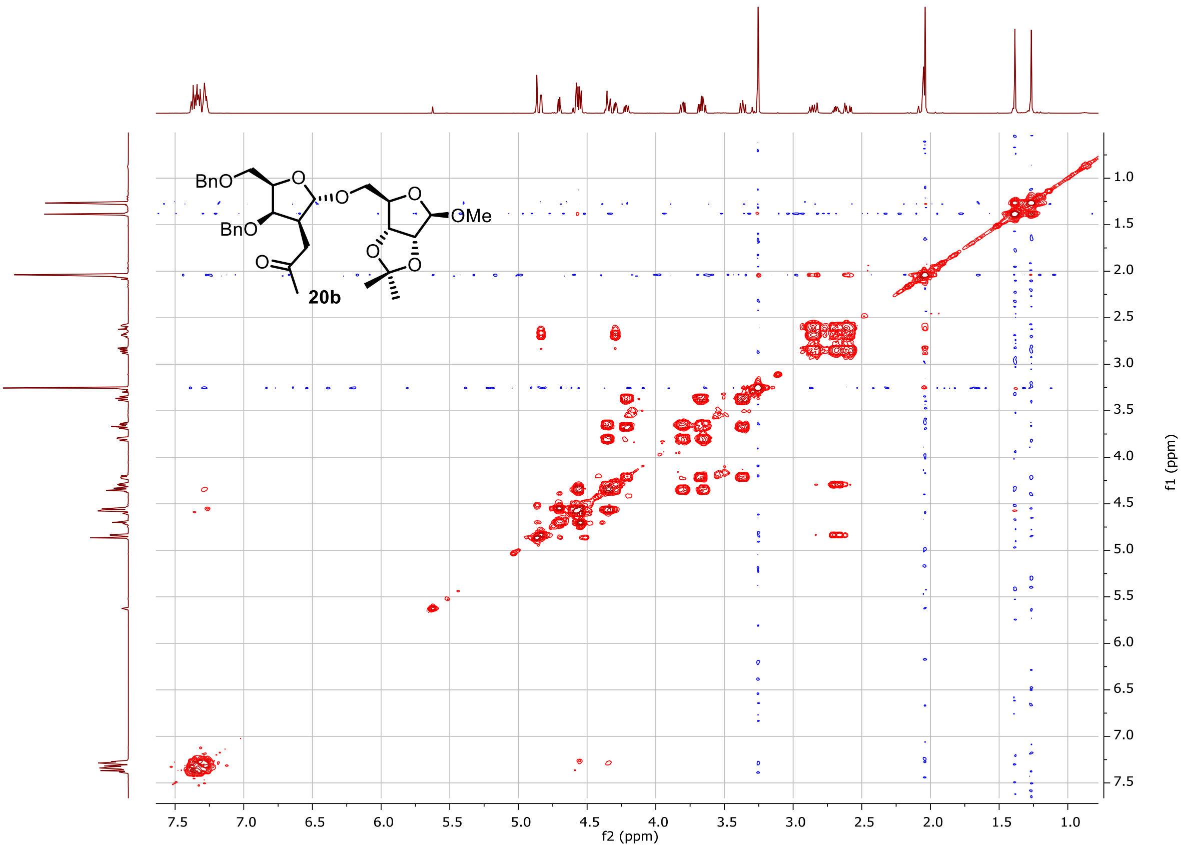

Supplementary Figure 65. COSY spectra for **20b**

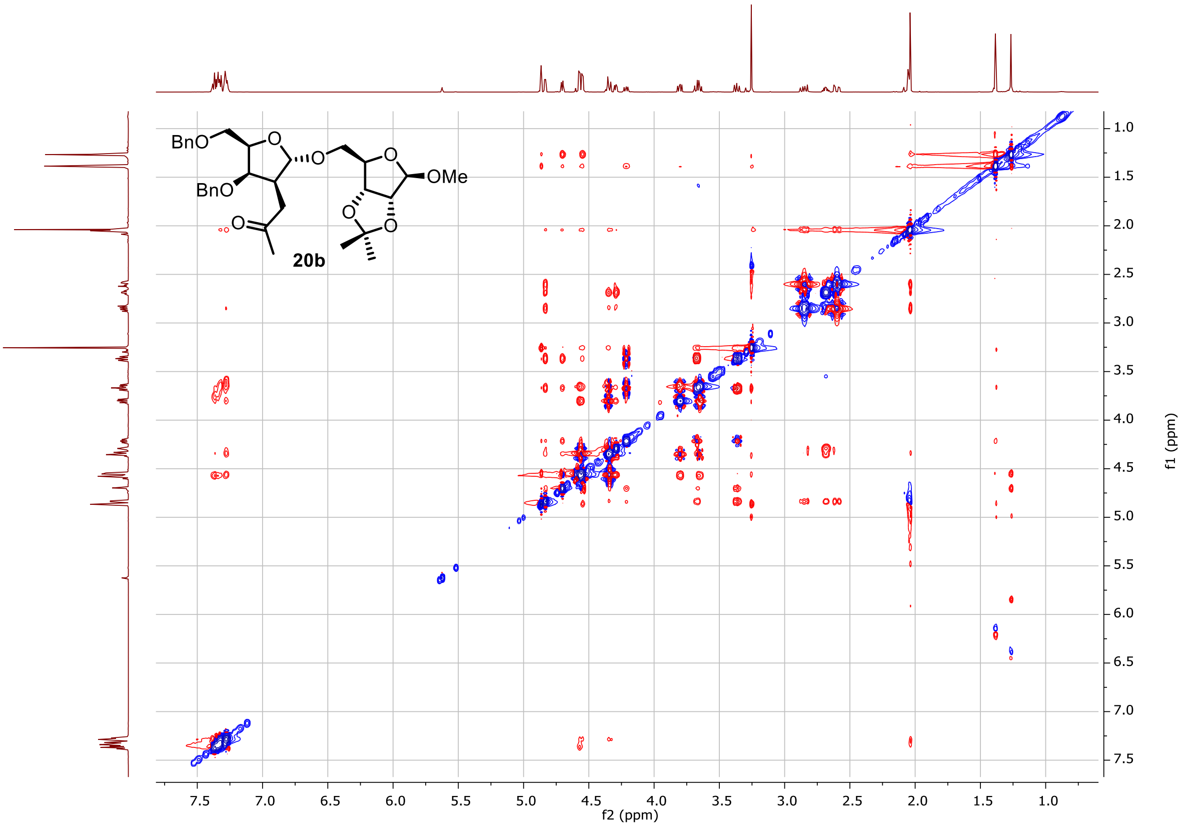

Supplementary Figure 66. NOESY spectra for **20b**

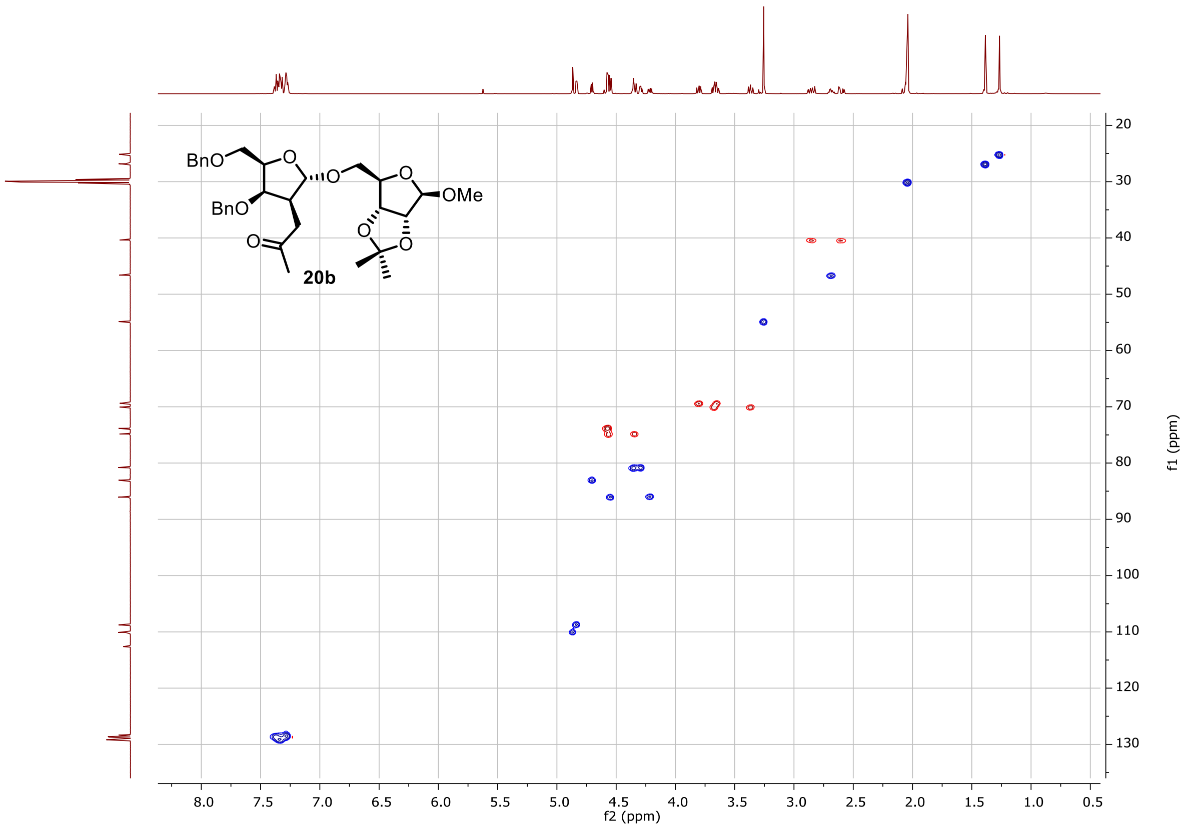

Supplementary Figure 67. HSQC spectra for **20b**

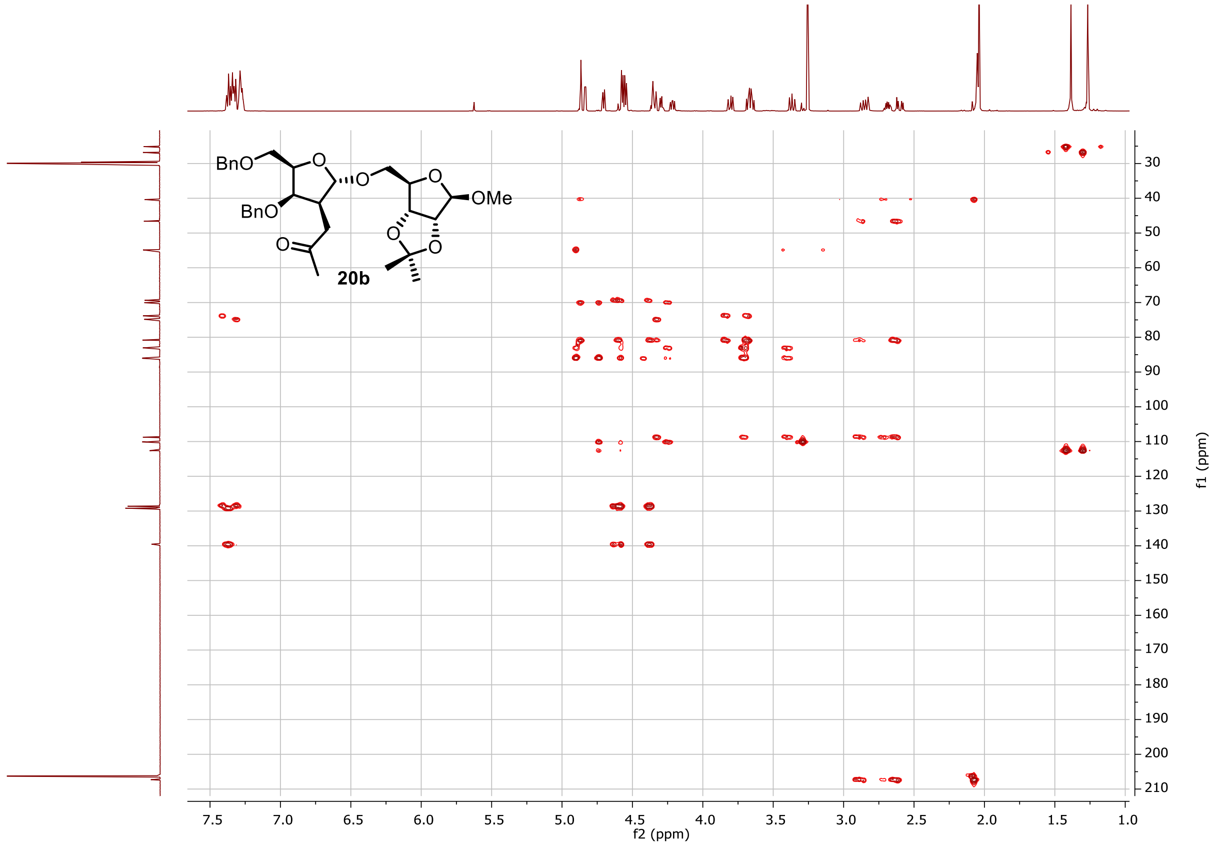

Supplementary Figure 68. HMBC spectra for **20b**

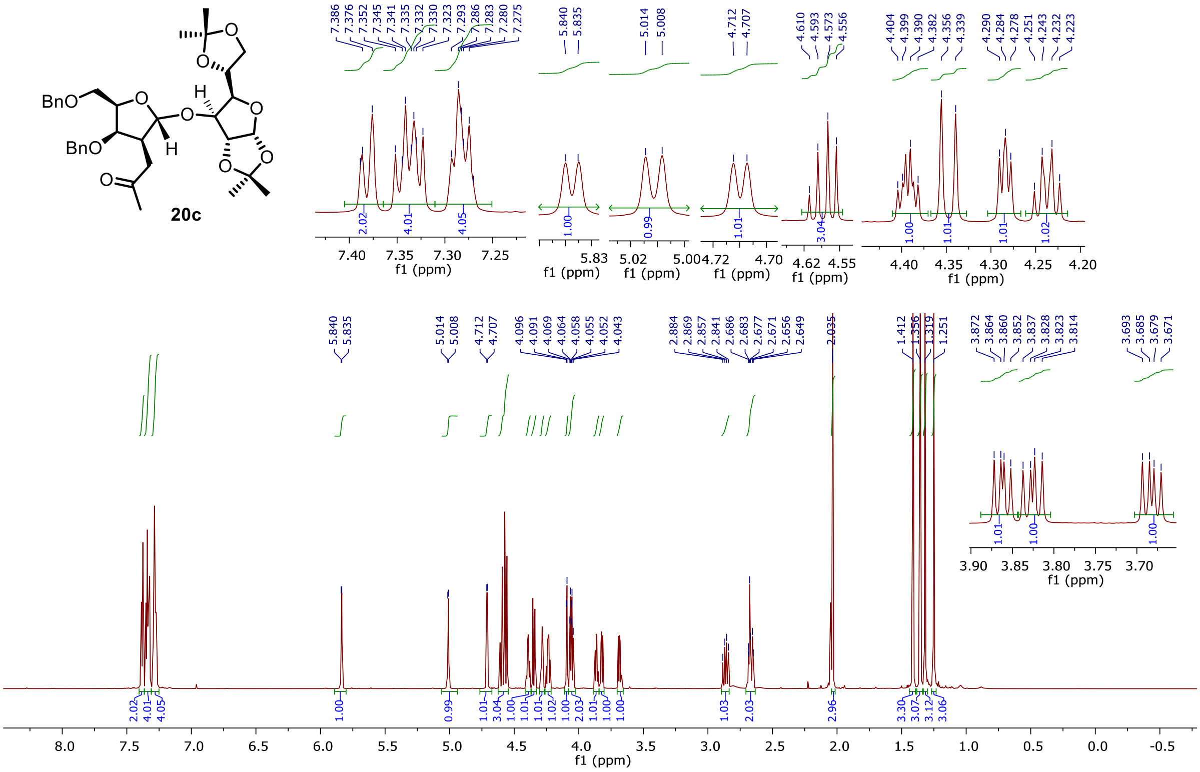

Supplementary Figure 69. ^{1}H spectra for **20c**

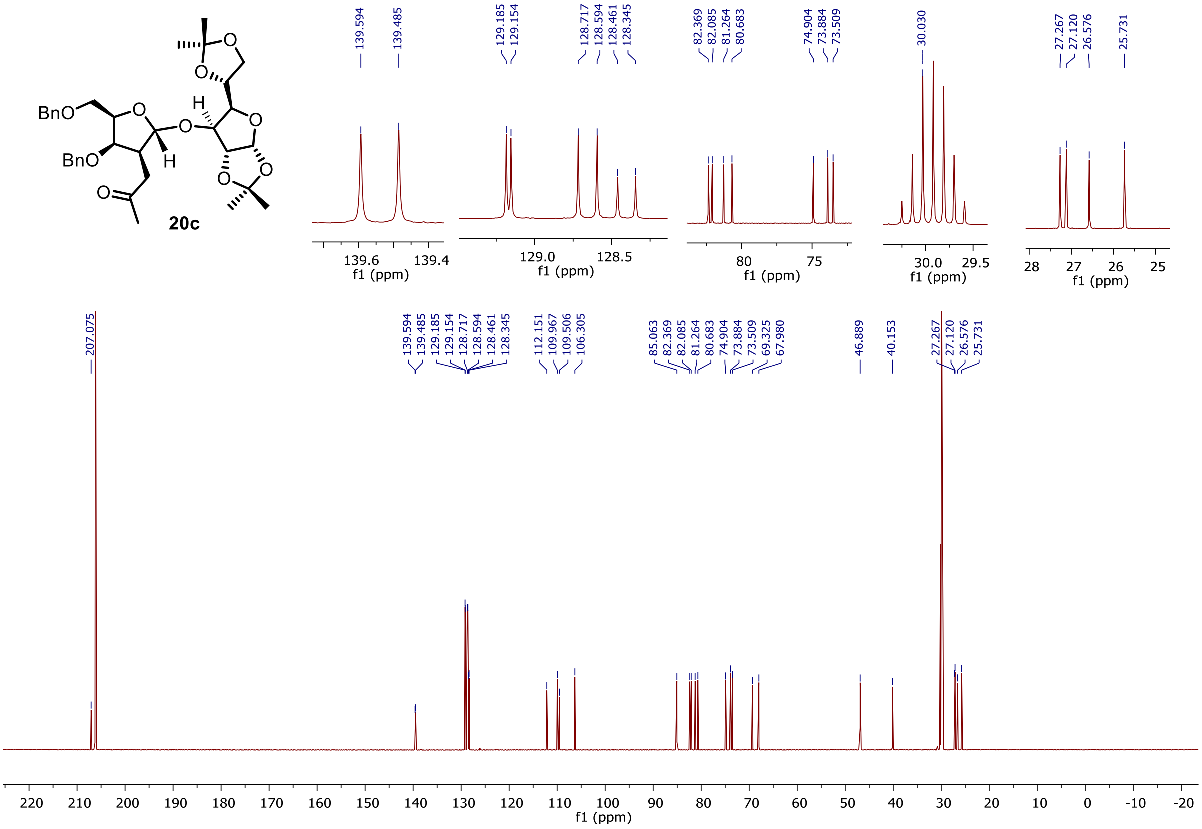

Supplementary Figure 70. ^{13}C spectra for **20c**

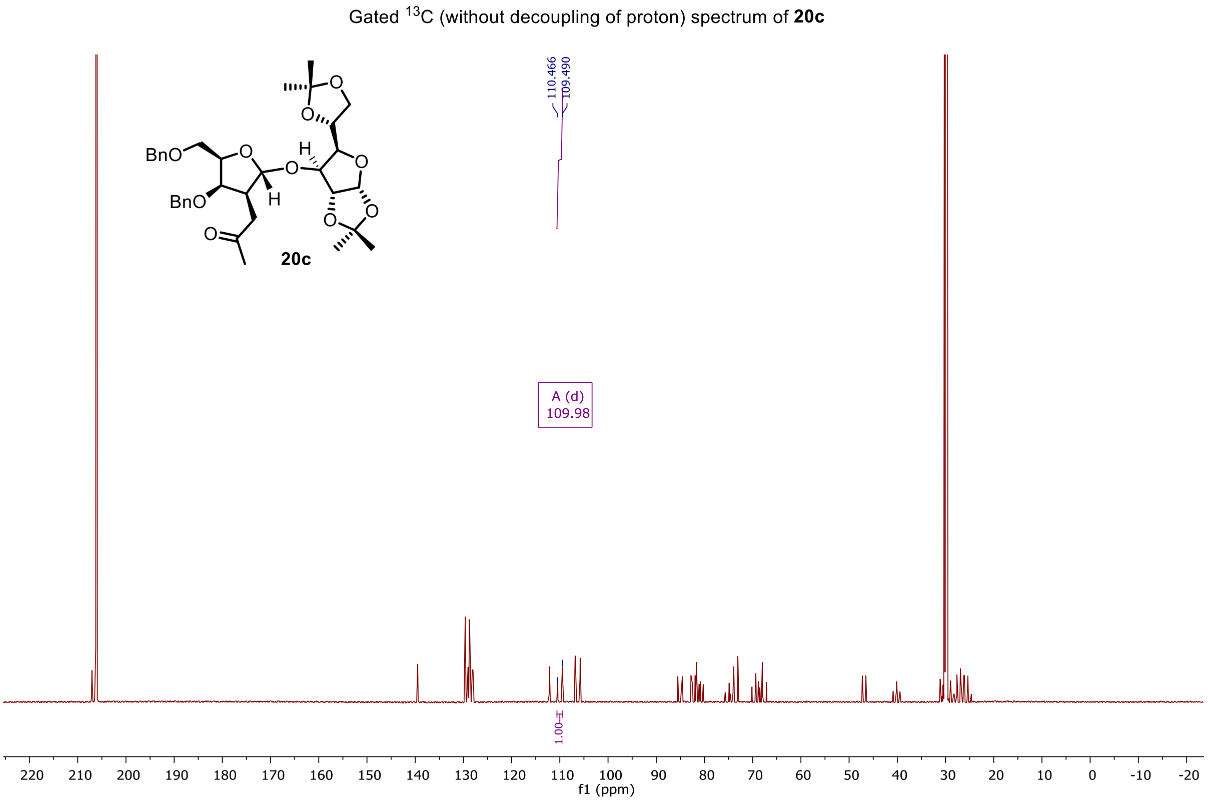

Supplementary Figure 71. Gated ^{13}C (with coupling of proton) spectra for **20c**

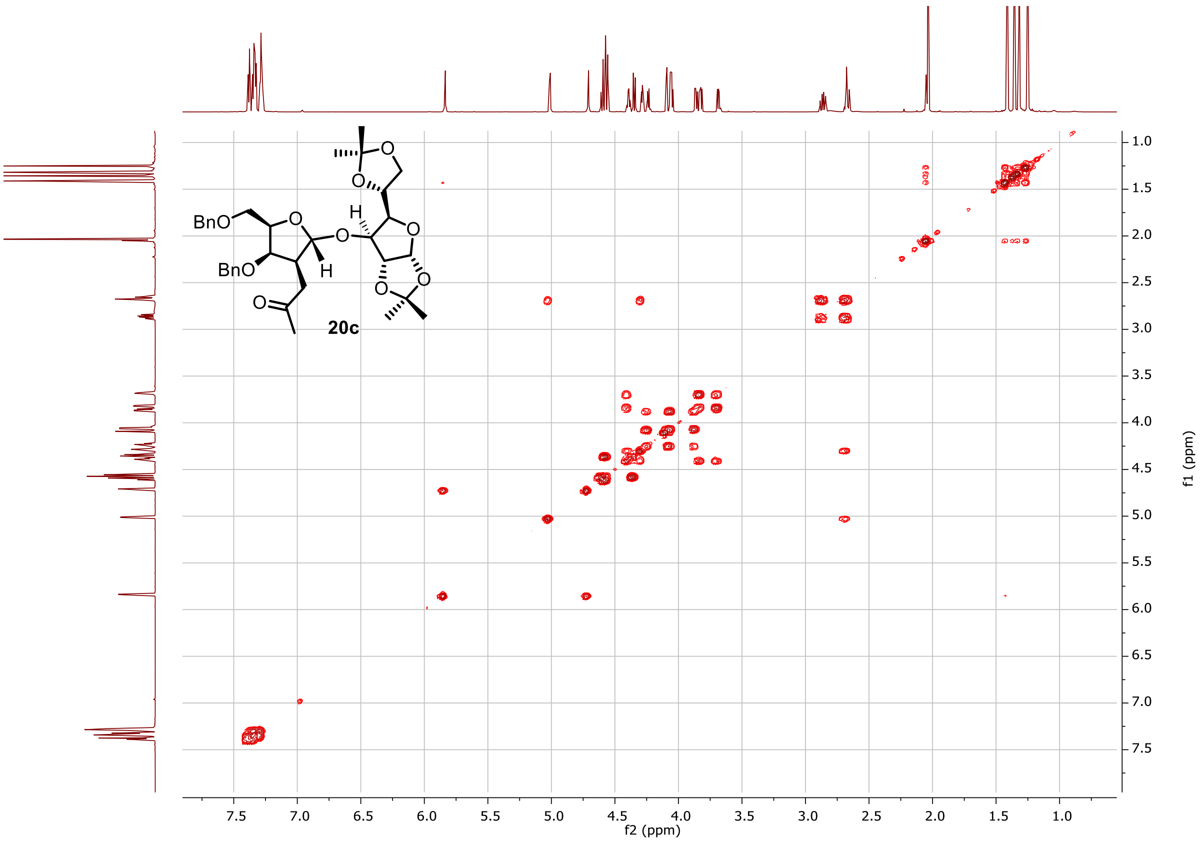

Supplementary Figure 72. COSY spectra for **20c**

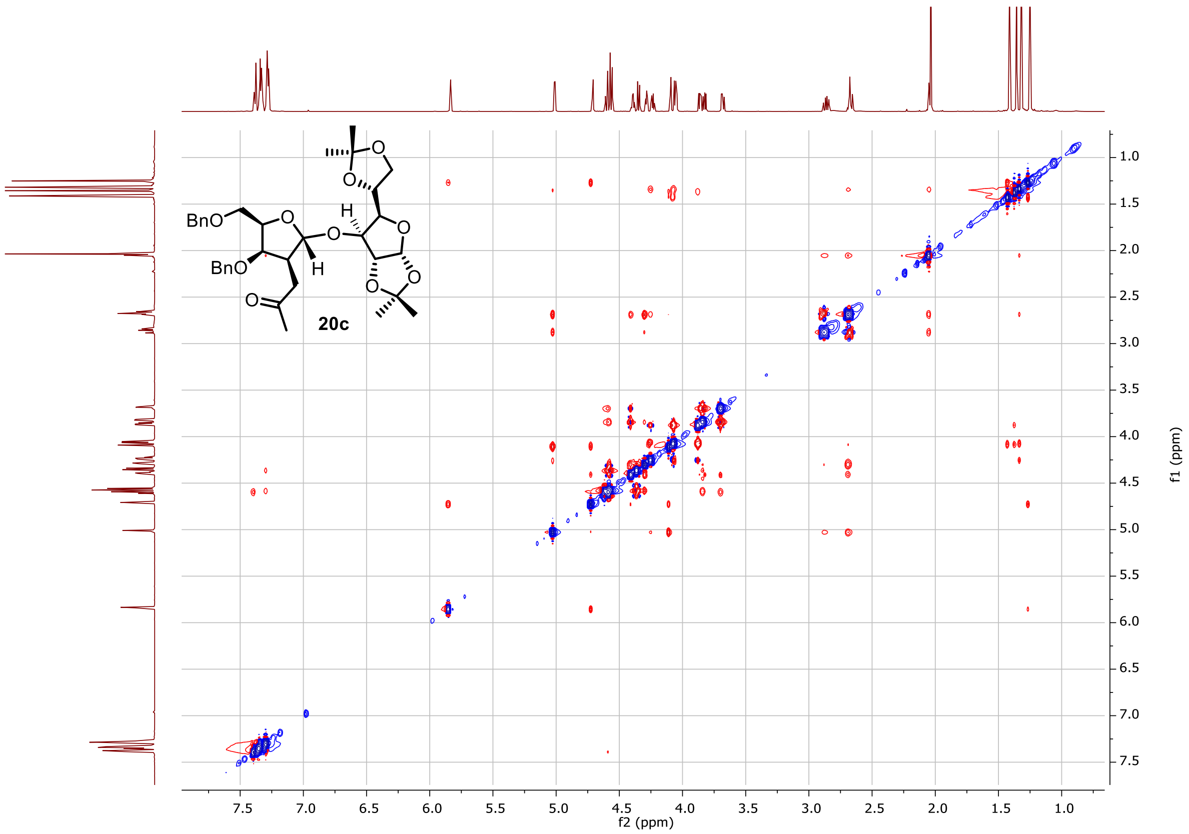

Supplementary Figure 73. NOESY spectra for **20c**

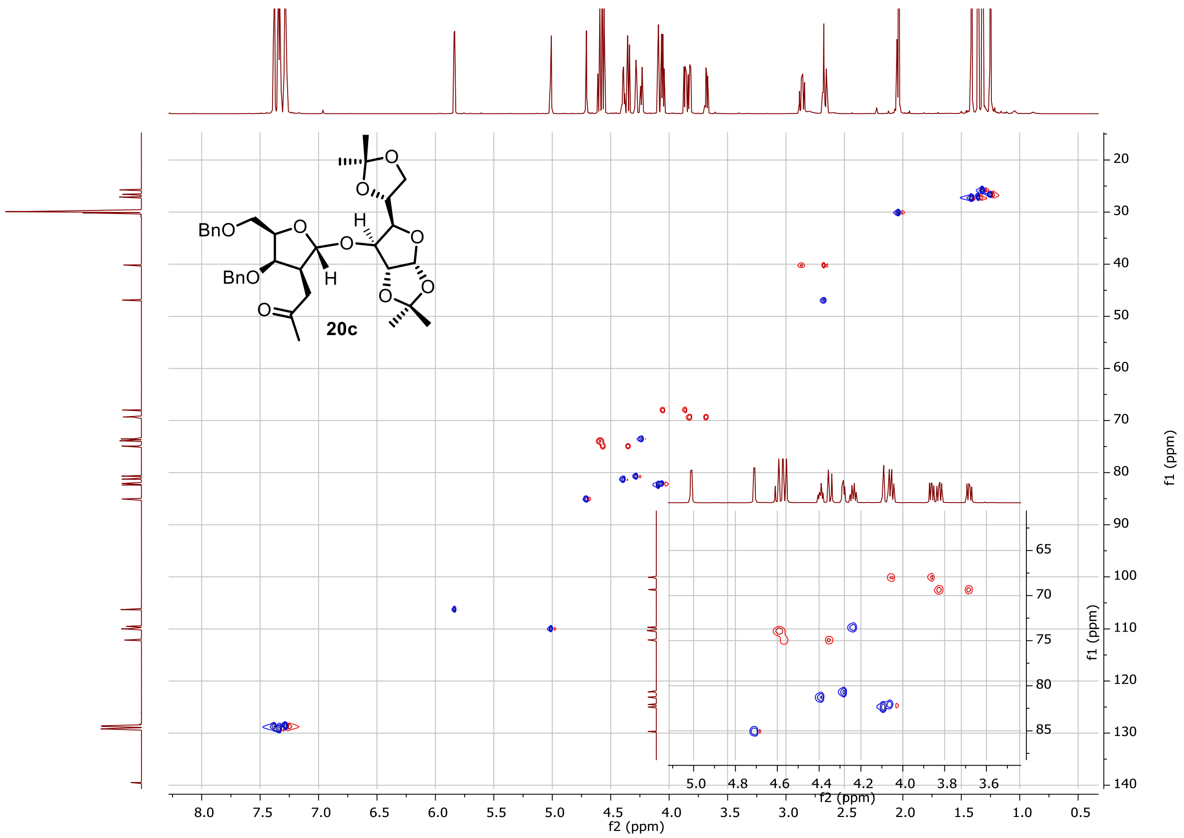

Supplementary Figure 74. HSQC spectra for **20c**

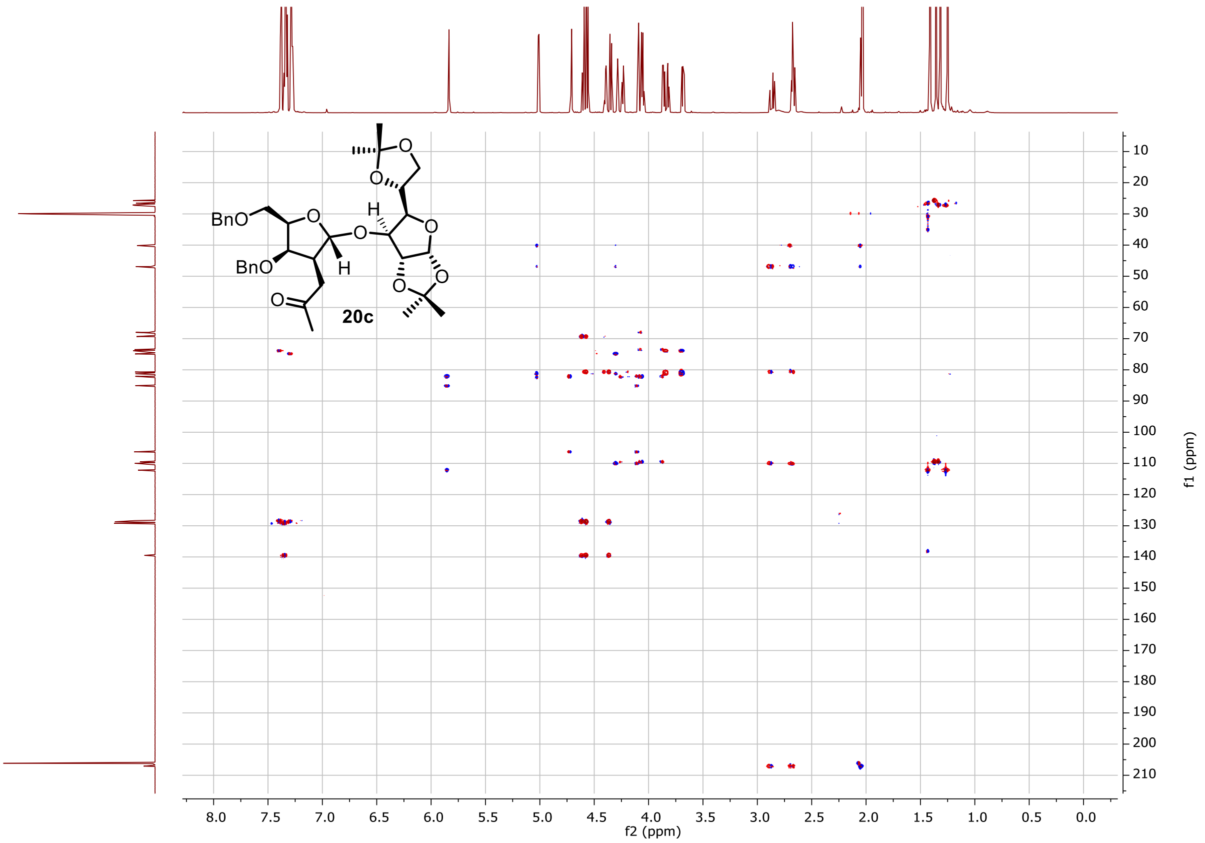

Supplementary Figure 75. HMBC spectra for **20c**

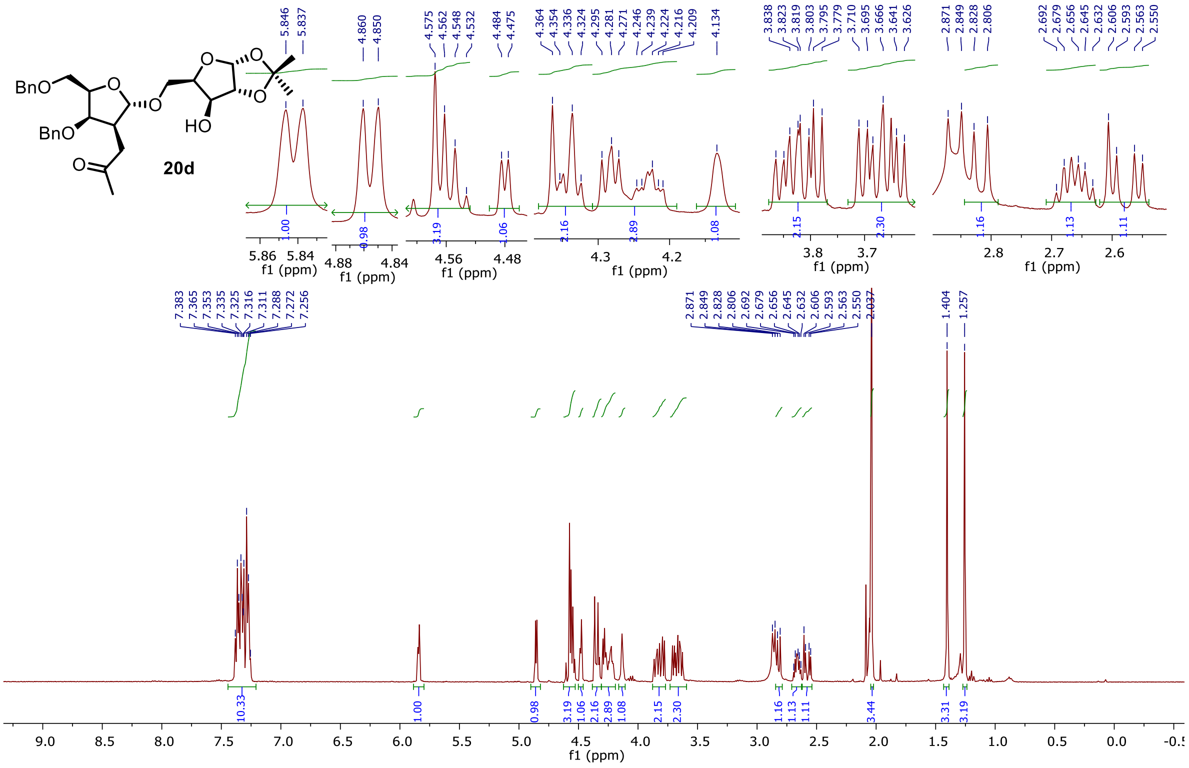

Supplementary Figure 76. ^{1}H spectra for **20d**

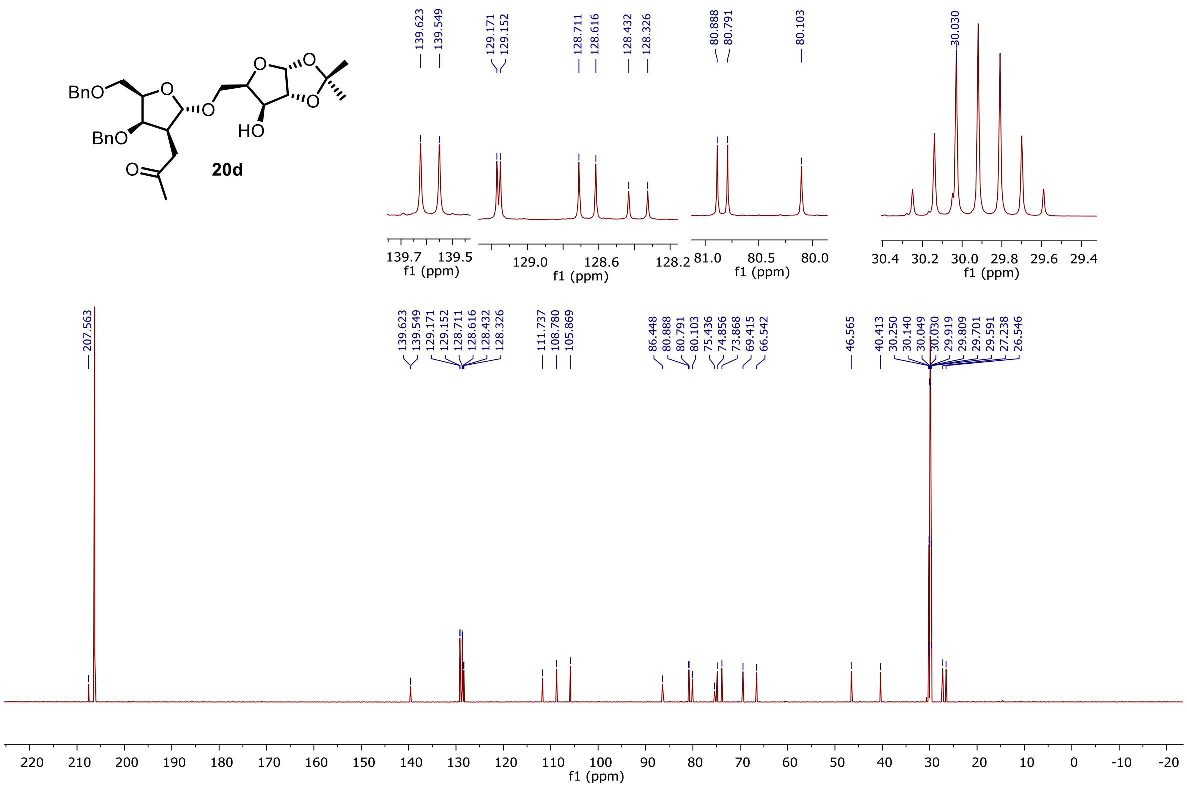

Supplementary Figure 77. ^{13}C spectra for **20d**

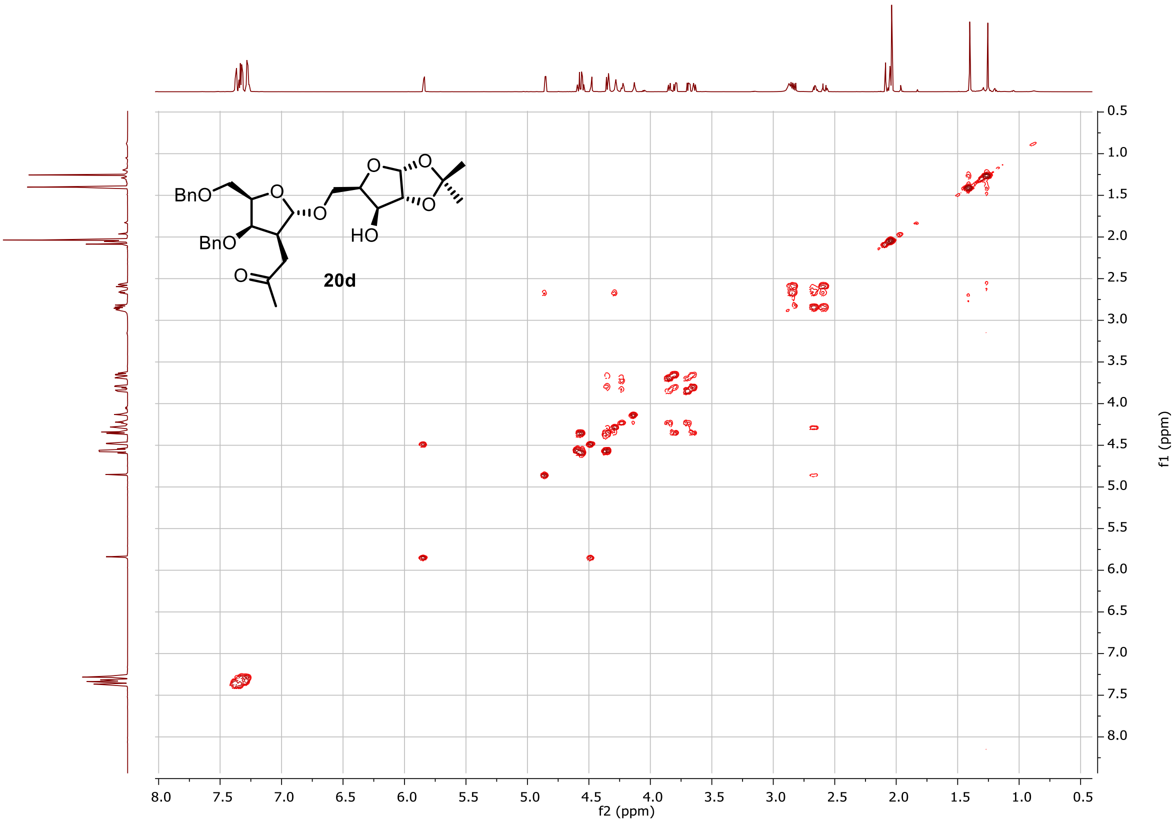

Supplementary Figure 78. COSY spectra for **20d**

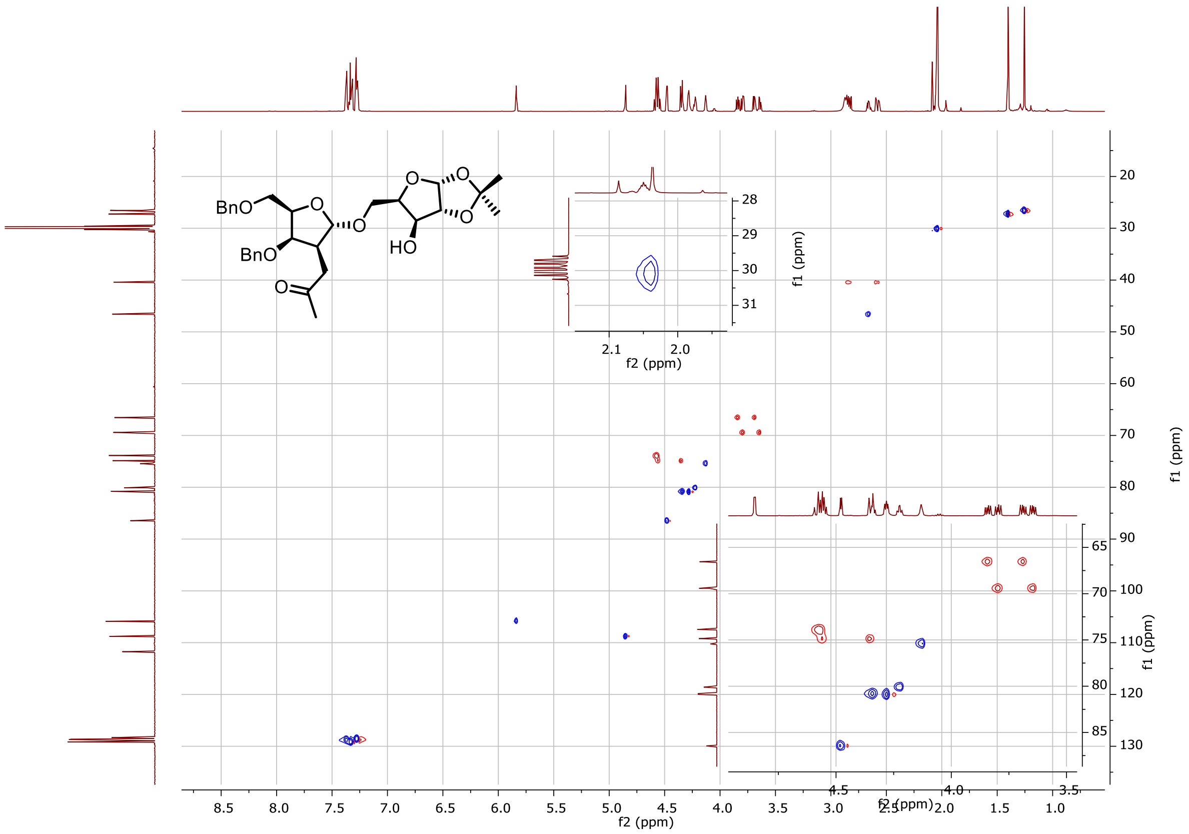

Supplementary Figure 79. HSQC spectra for **20d**

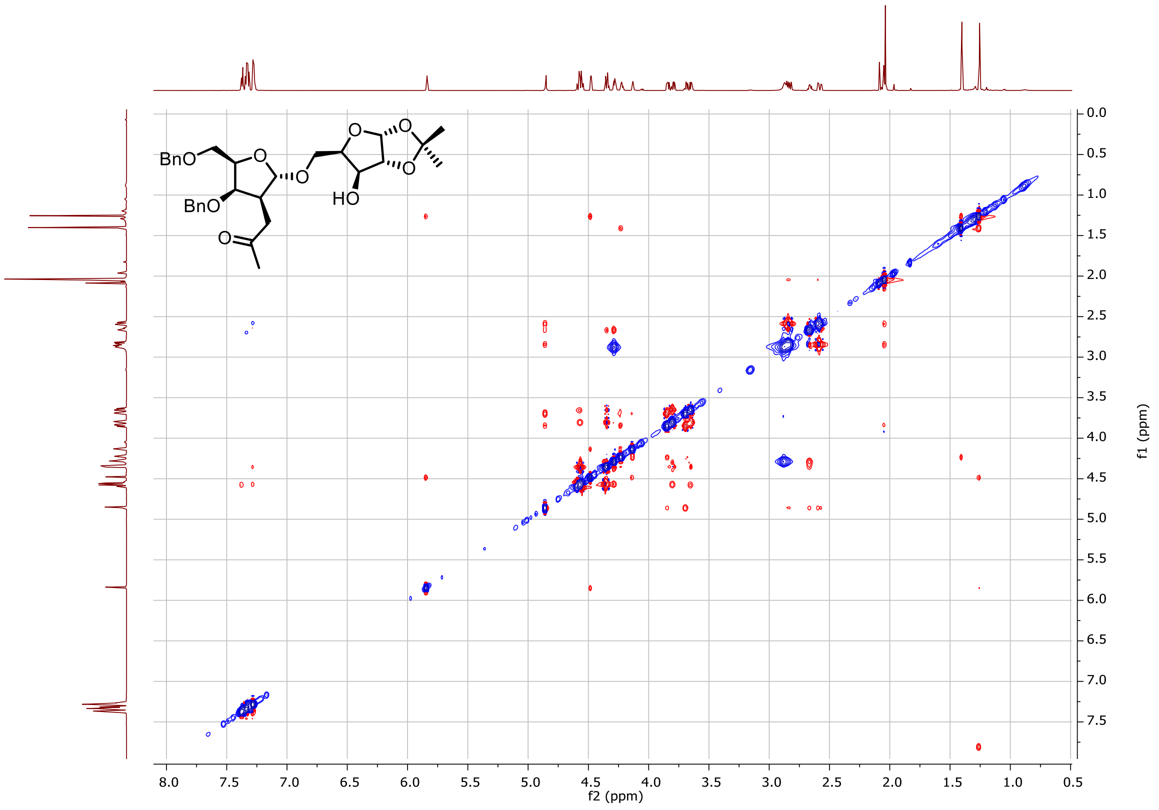

Supplementary Figure 80. NOESY spectra for **20d**

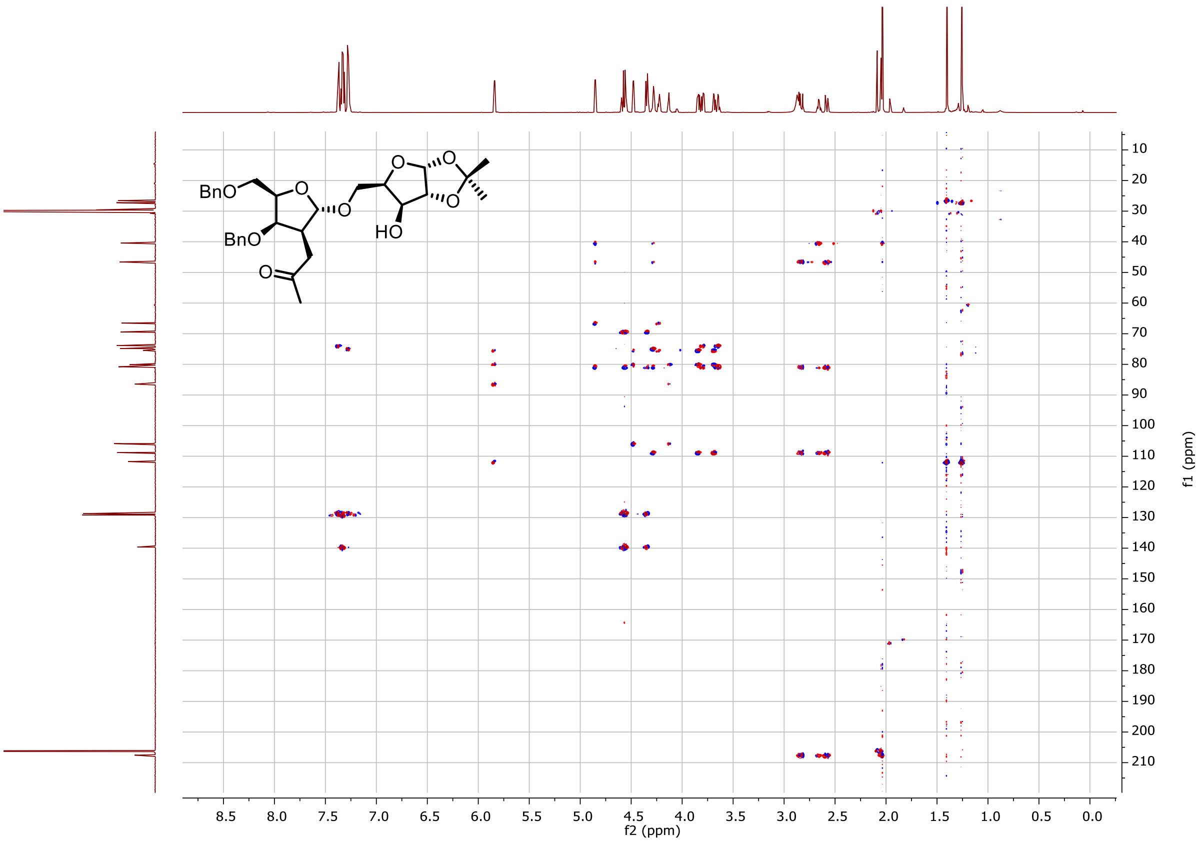

Supplementary Figure 81. HMBC spectra for **20d**

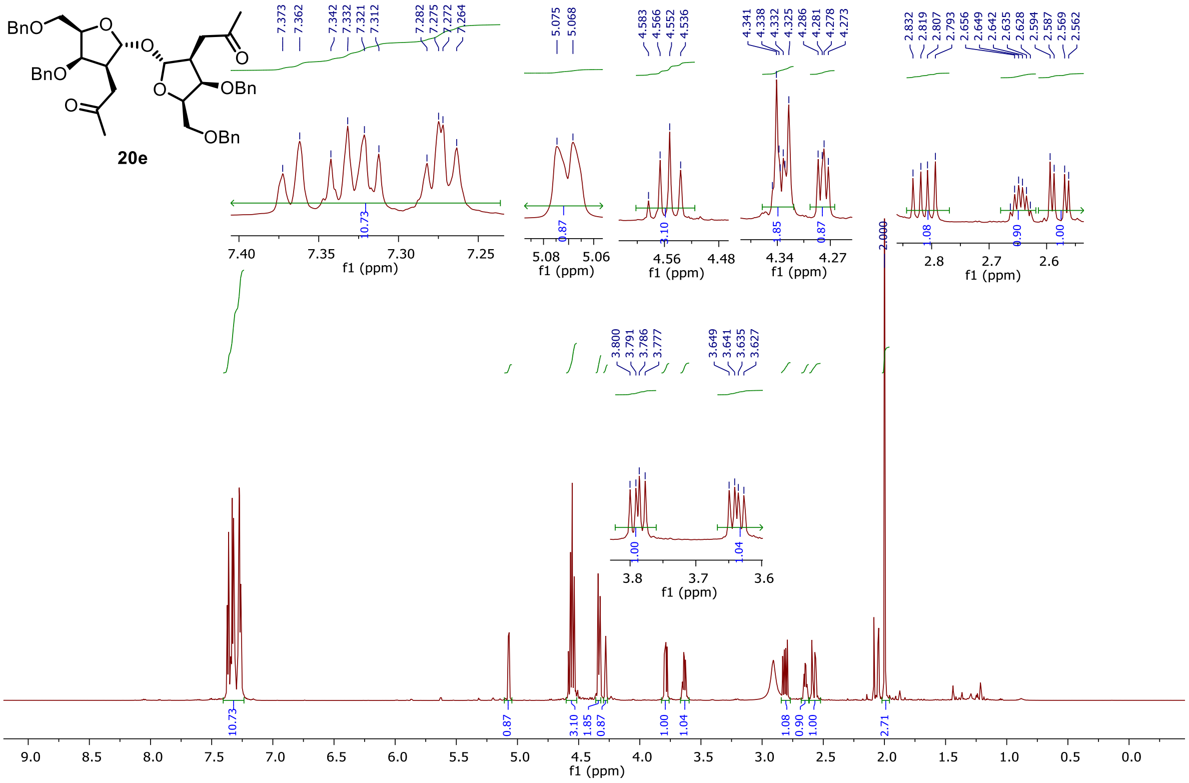

Supplementary Figure 82. ¹H spectra for **20e**

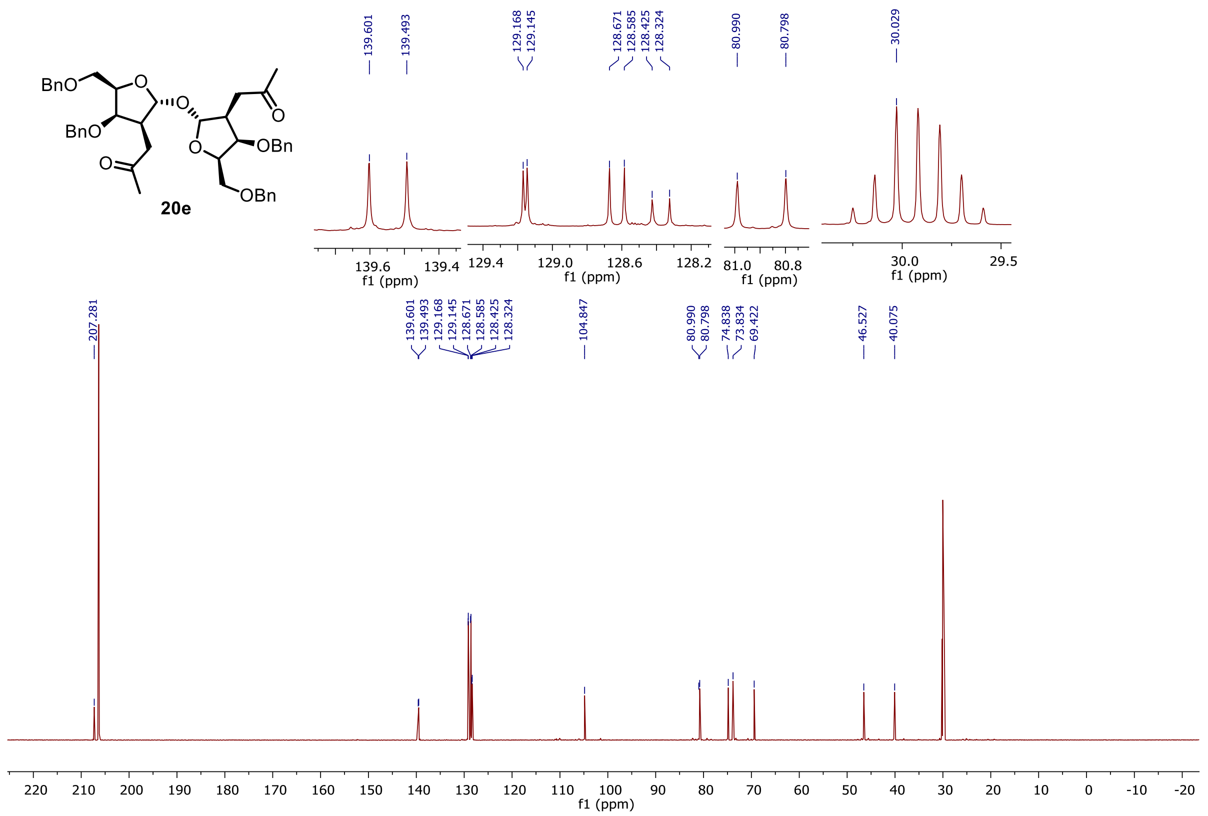

Supplementary Figure 83. ^{13}C spectra for **20e**

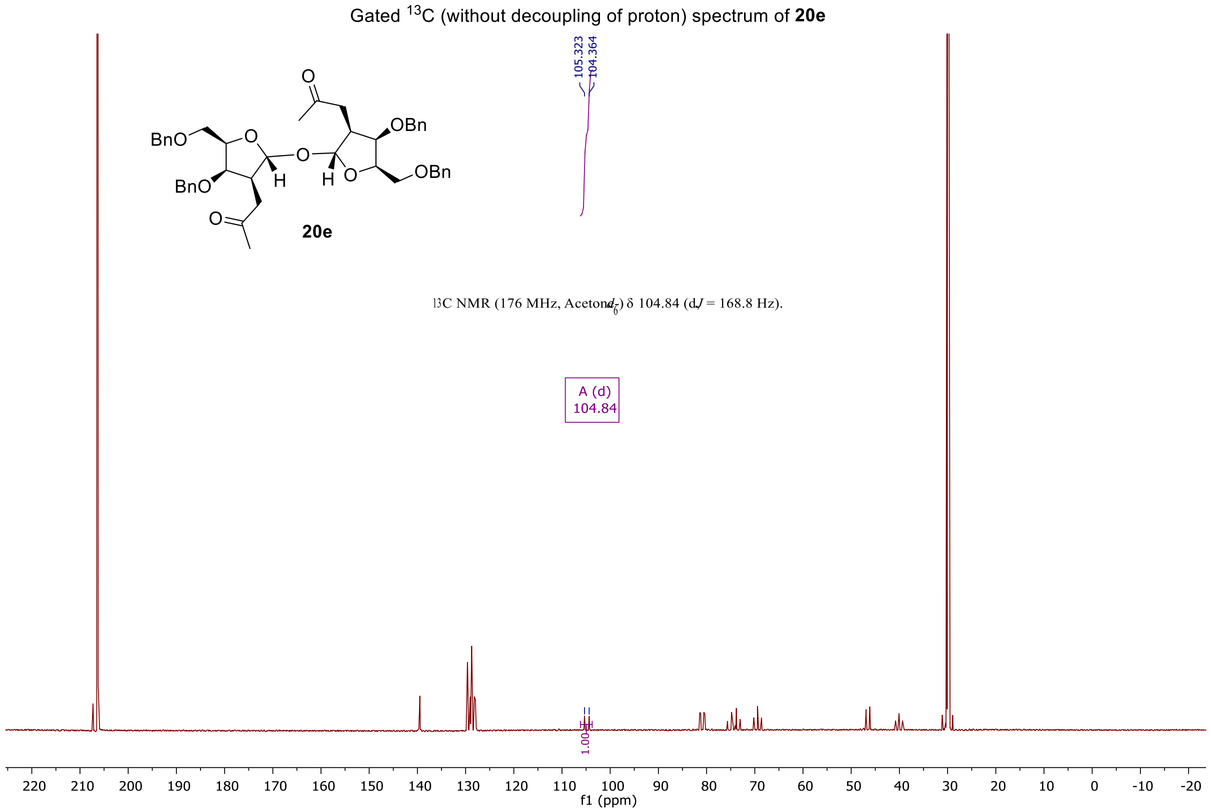

Supplementary Figure 84. Gated ^{13}C (with coupling of proton) spectra for **20e**

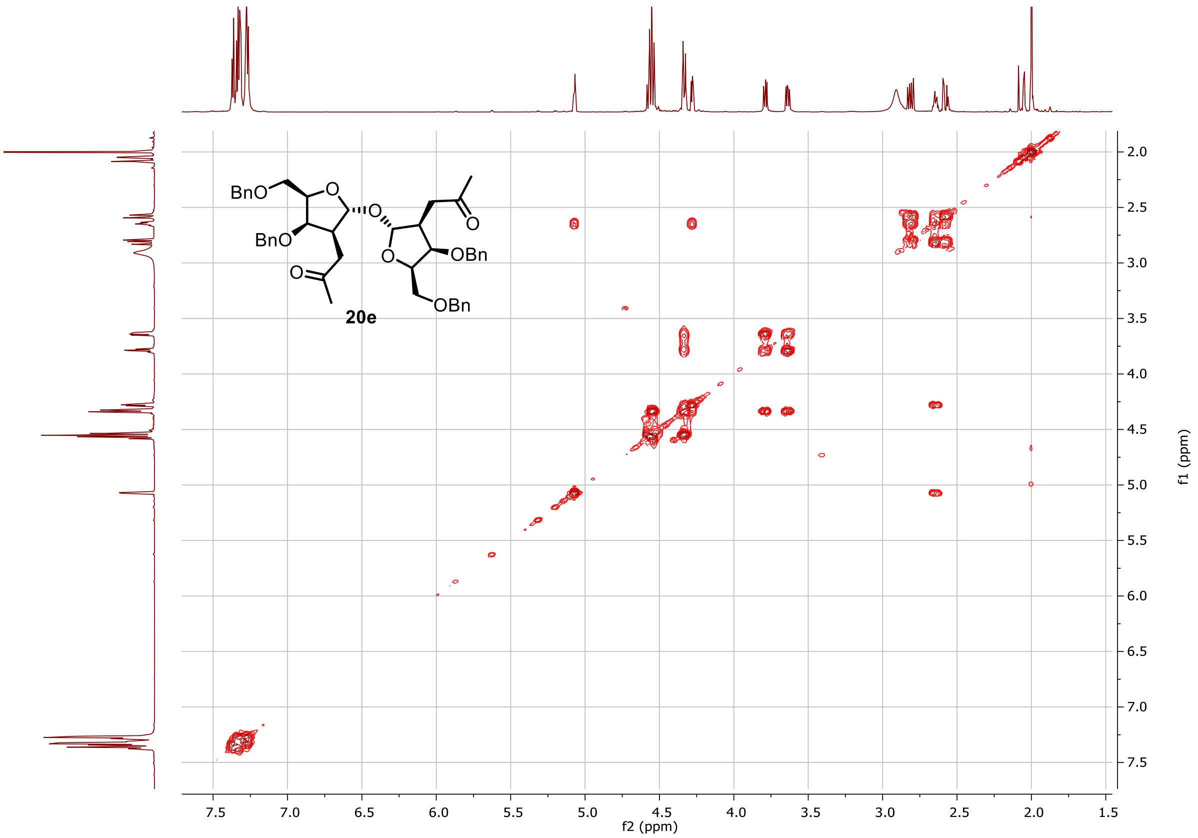

Supplementary Figure 85. COSY spectra for **20e**

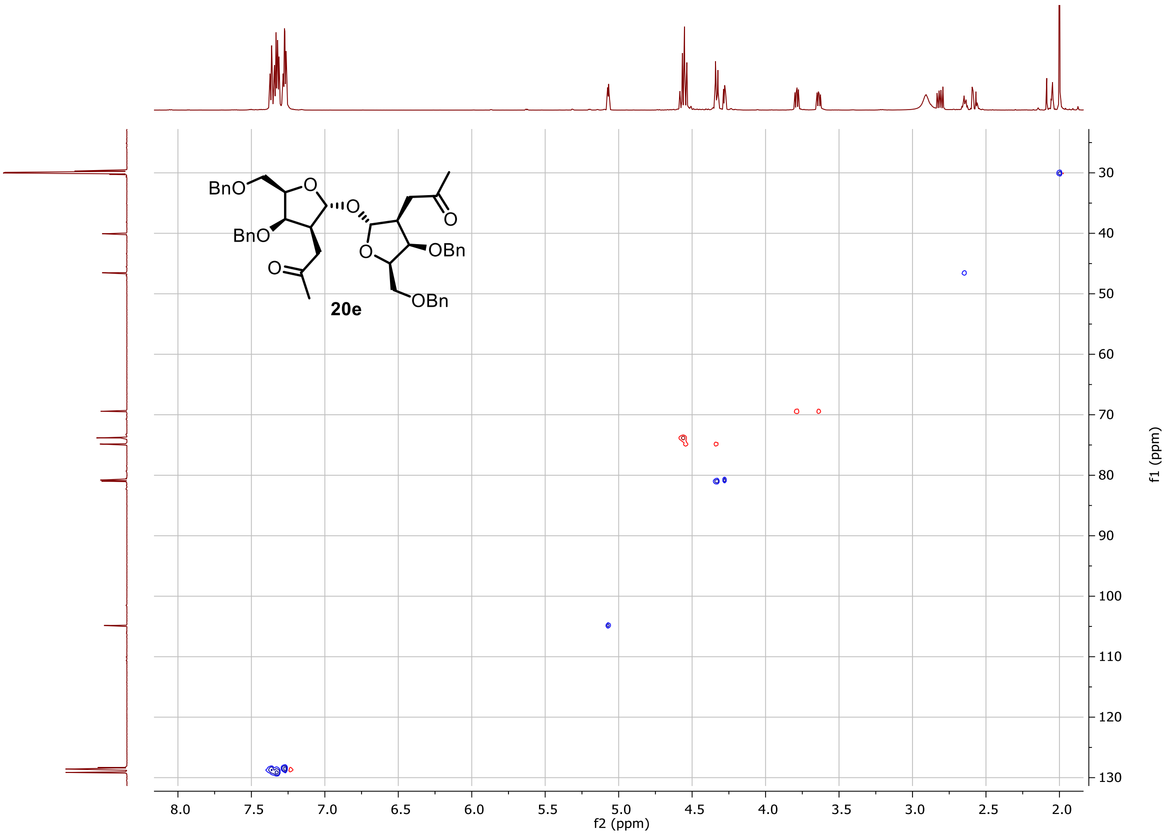

Supplementary Figure 86. HSQC spectra for **20e**

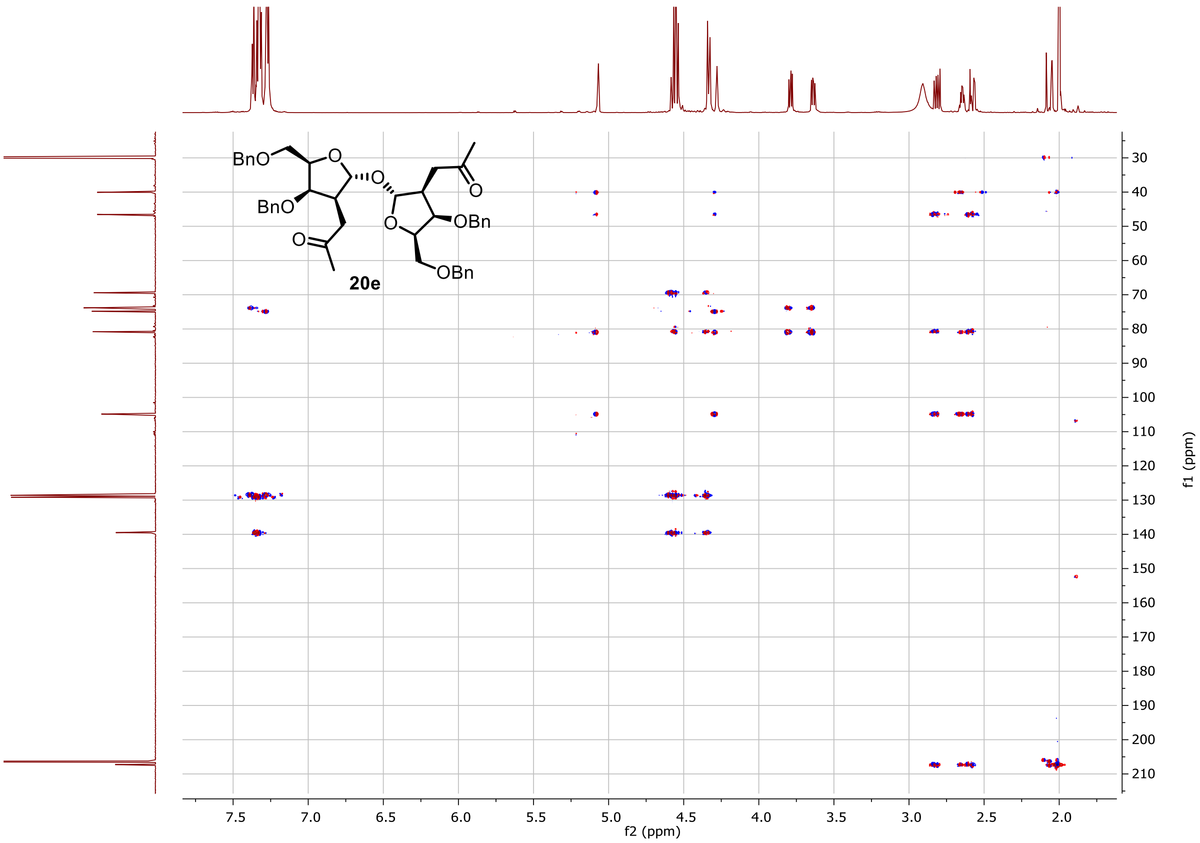

Supplementary Figure 87. HMBC spectra for **20e**

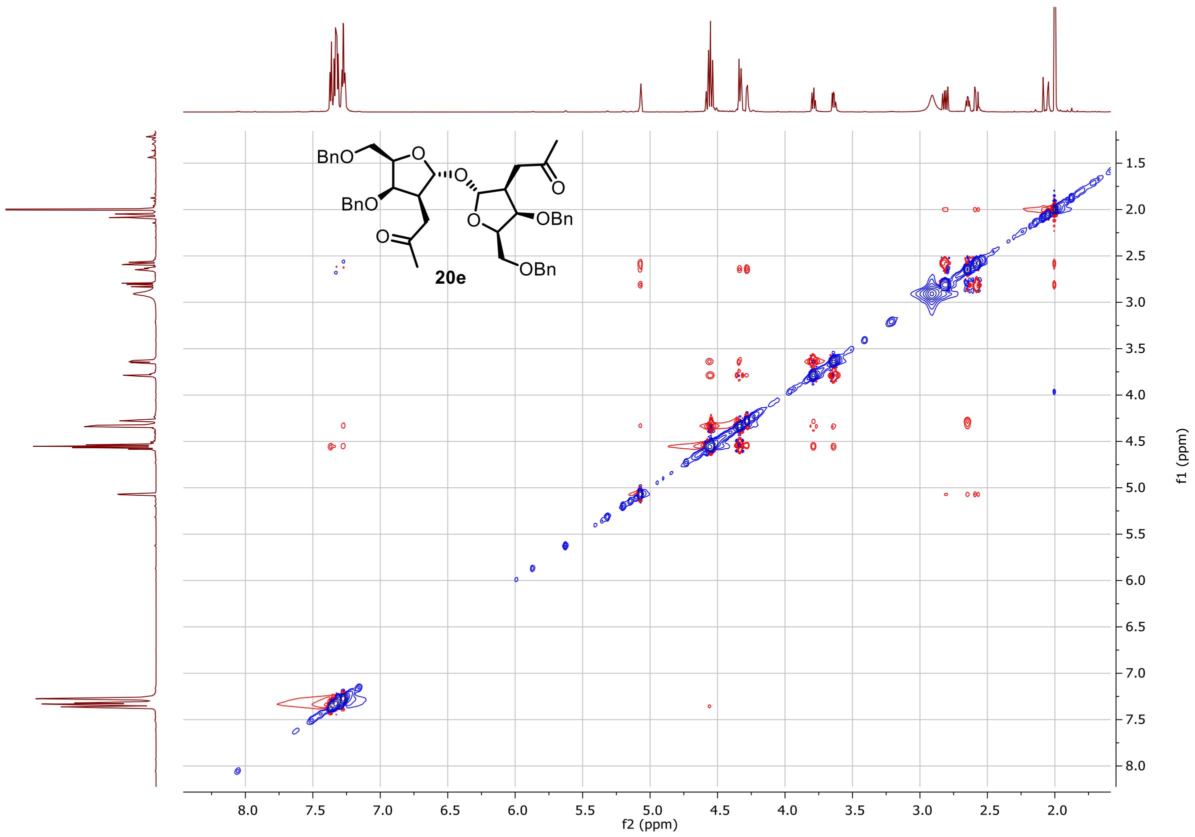

Supplementary Figure 88. NOESY spectra for **20e**

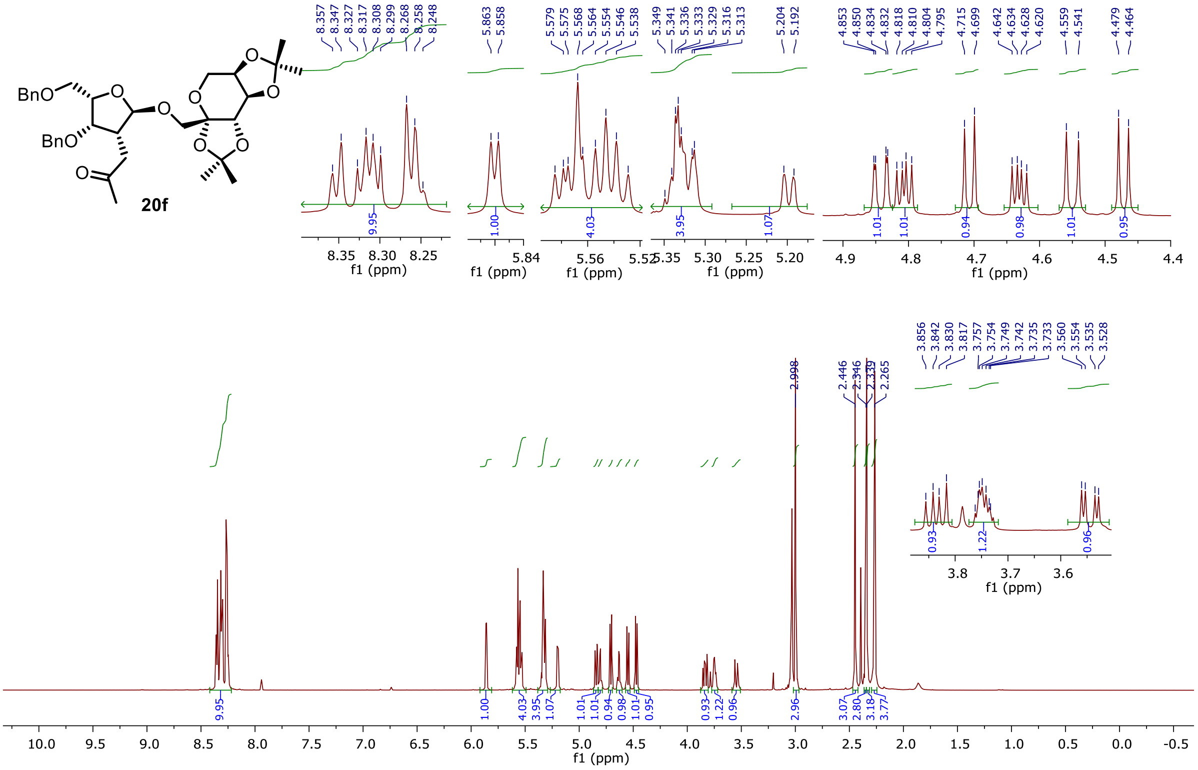

Supplementary Figure 89. ^{1}H spectra for **20f**

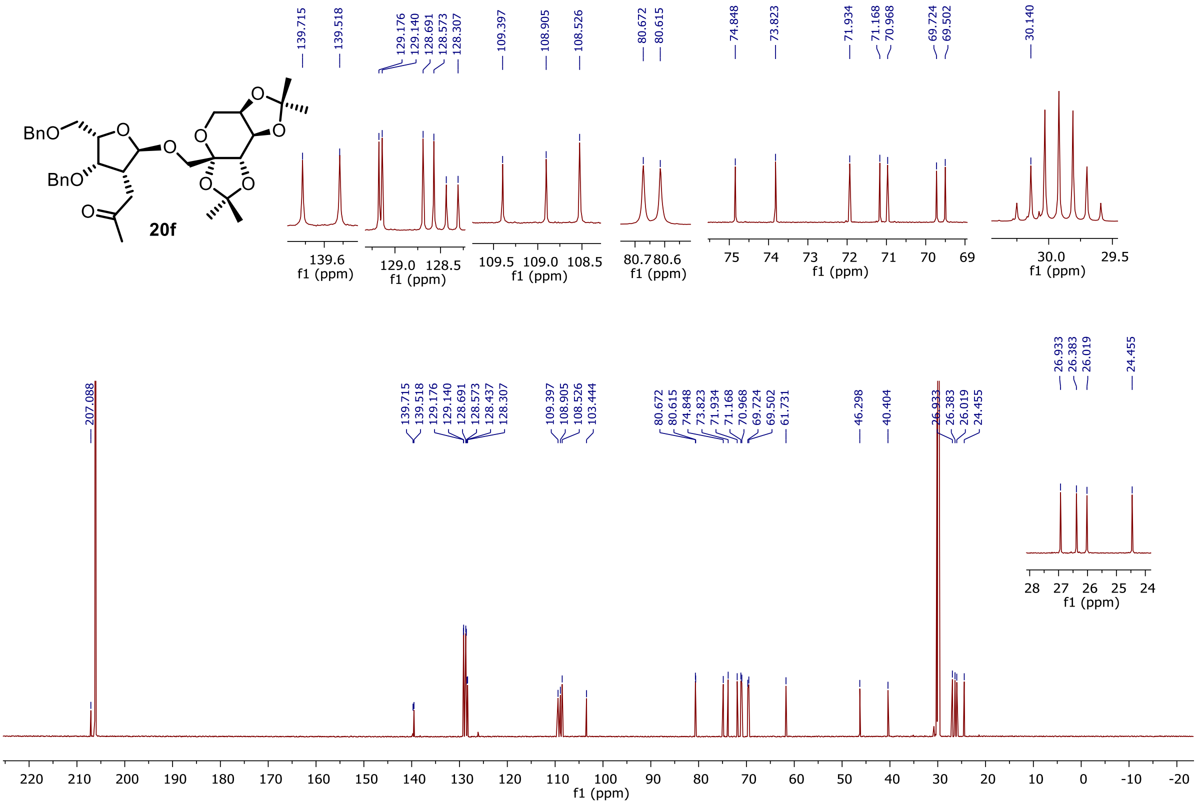

Supplementary Figure 90. ^{13}C spectra for **20f**

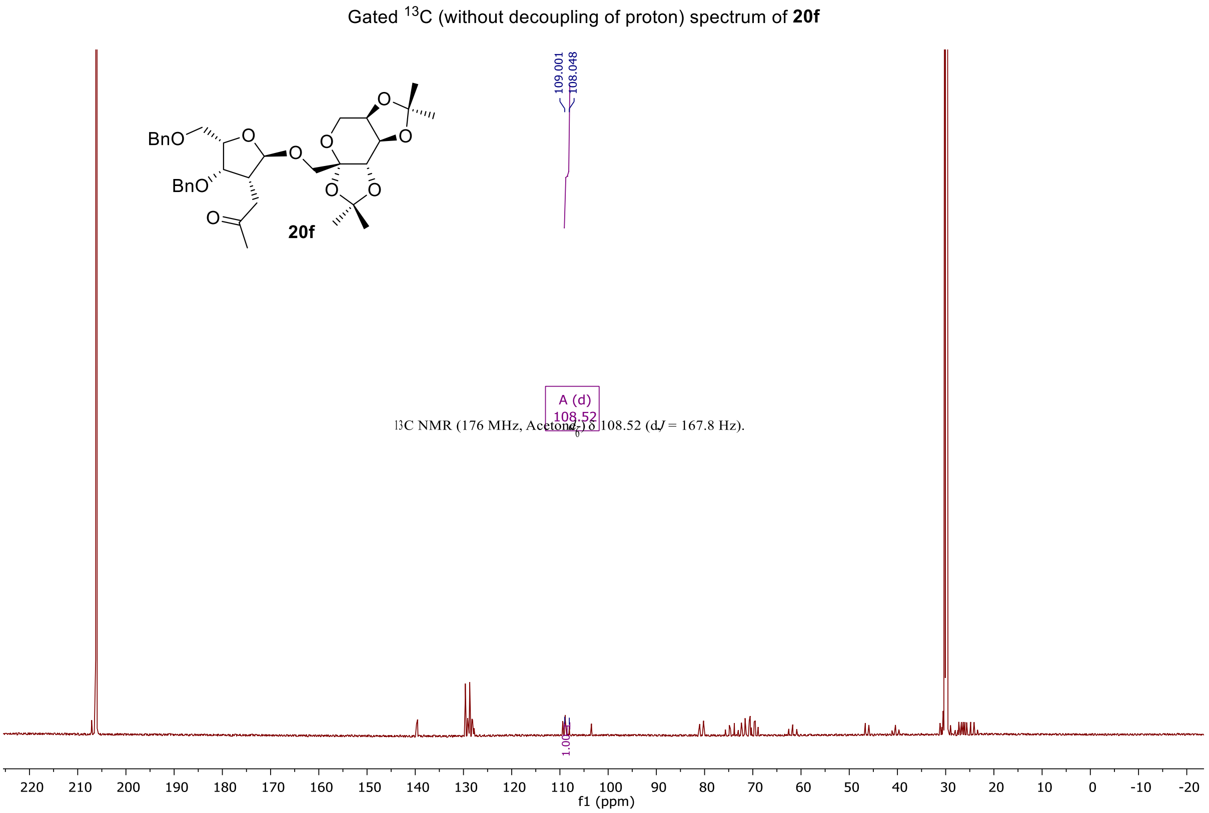

Supplementary Figure 91. Gated ^{13}C (with coupling of proton) spectra for **20f**

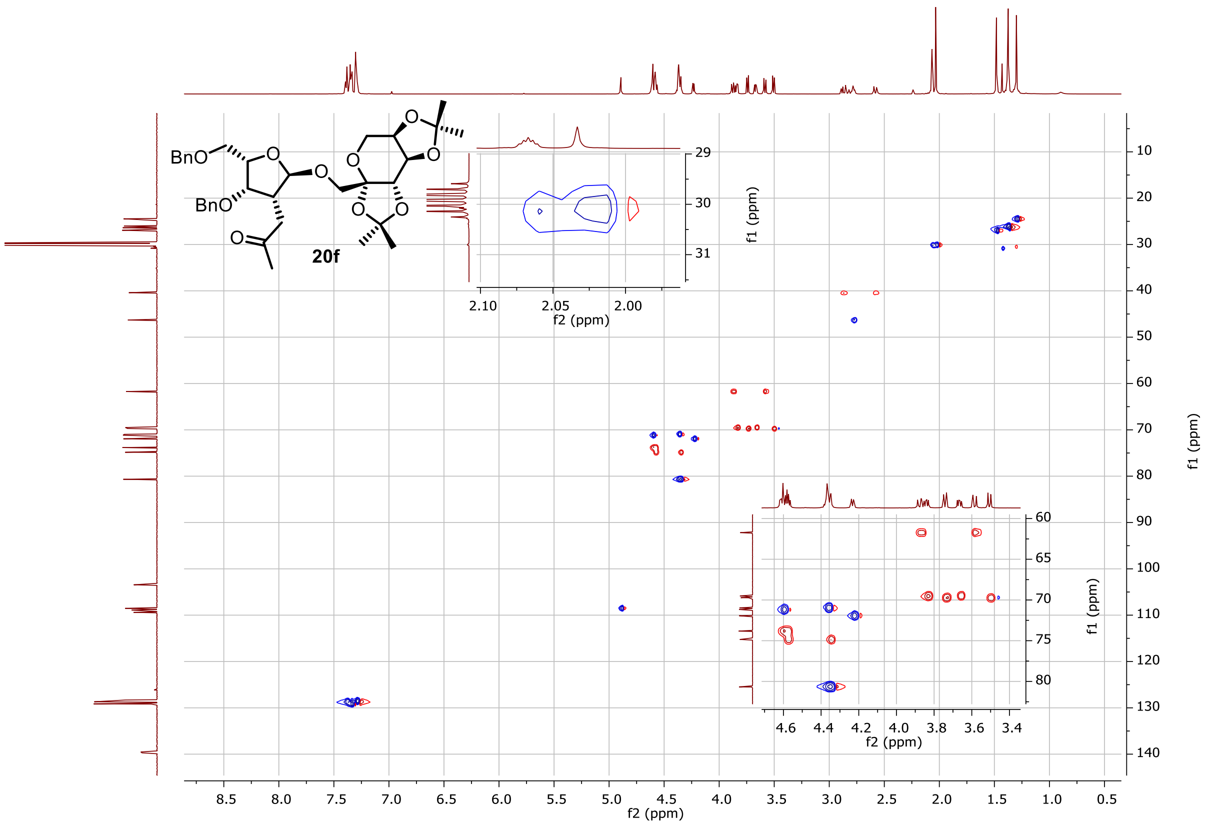

Supplementary Figure 92. HSQC spectra for **20f**

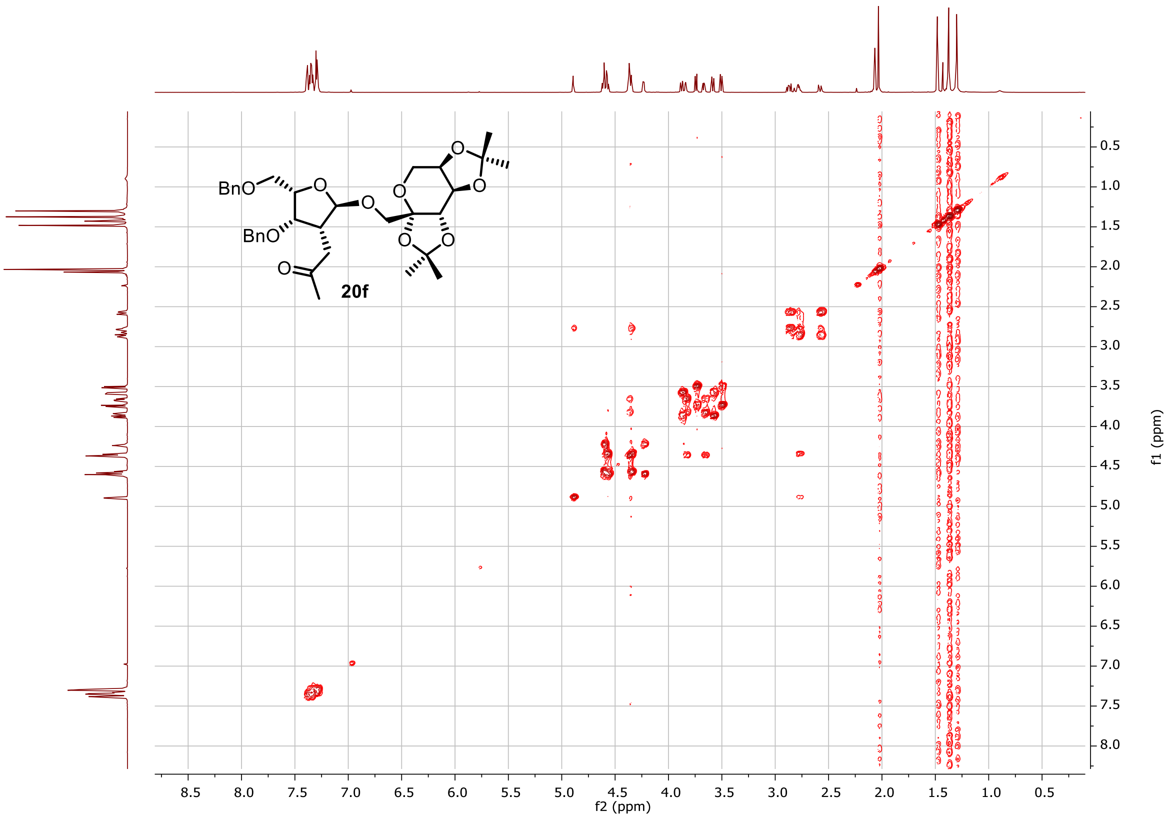

Supplementary Figure 93. COSY spectra for **20f**

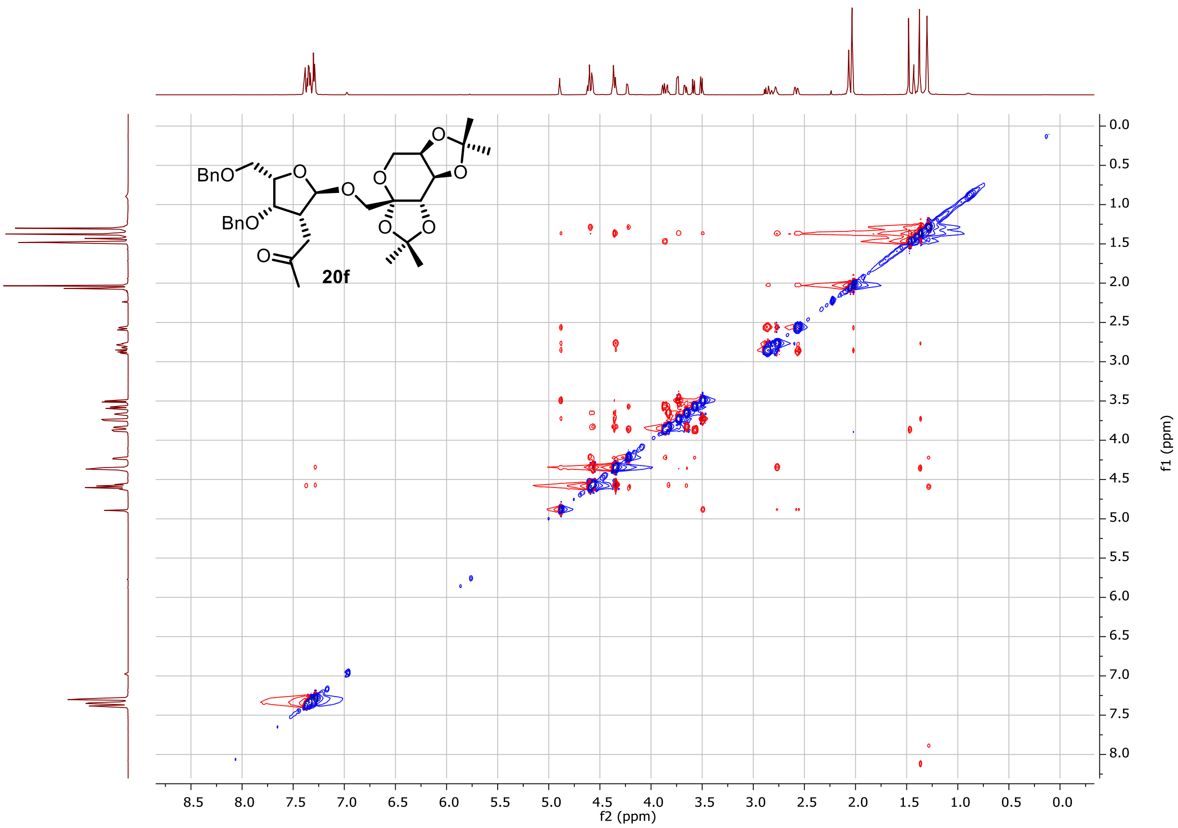

Supplementary Figure 94. NOESY spectra for **20f**

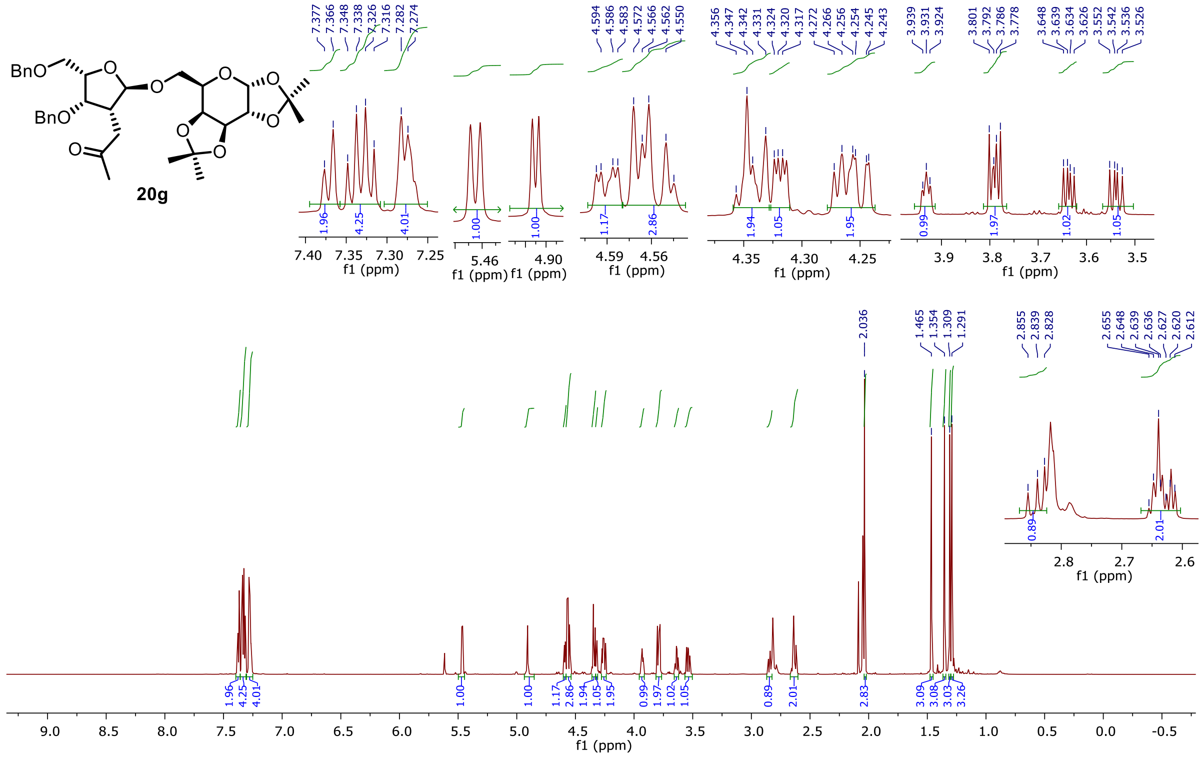

Supplementary Figure 95. ^{1}H spectra for **20g**

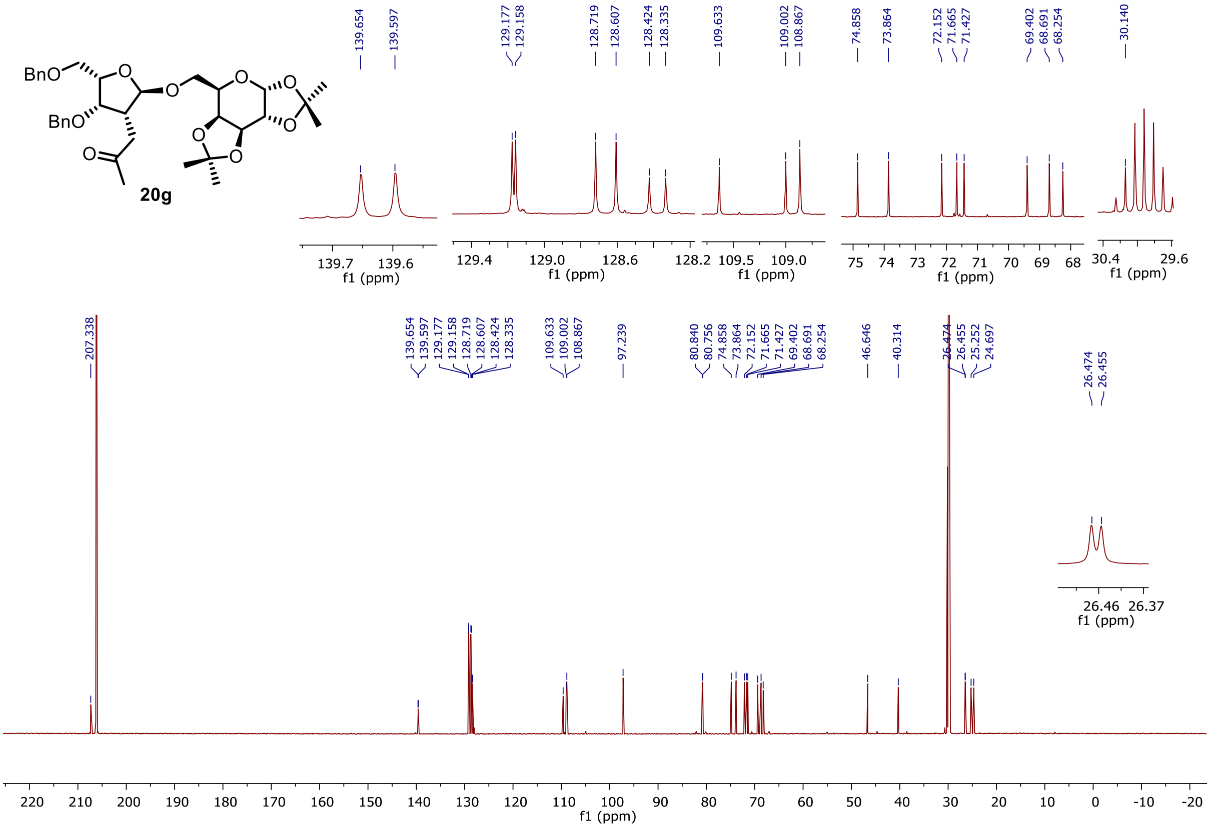

Supplementary Figure 96. ^{13}C spectra for **20g**

Gated ^{13}C (without decoupling of proton) spectrum of **20g**

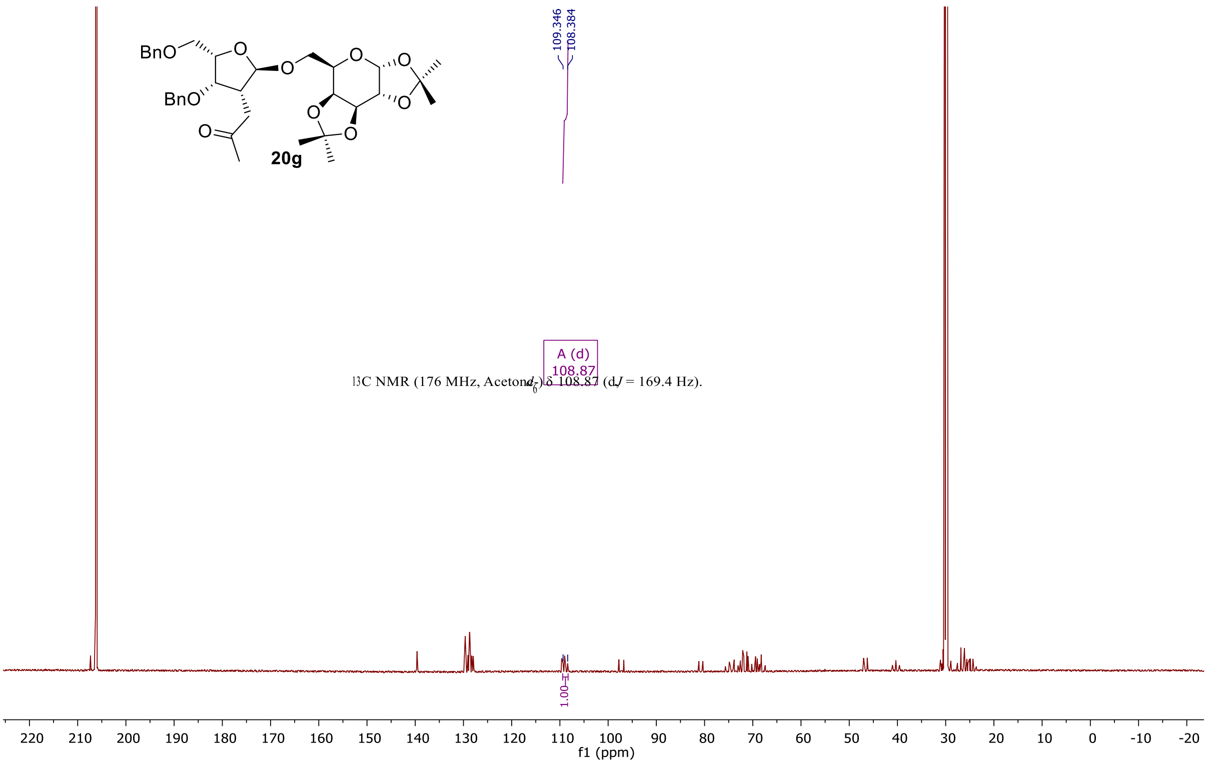

Supplementary Figure 97. Gated ^{13}C (with coupling of proton) spectra for **20g**

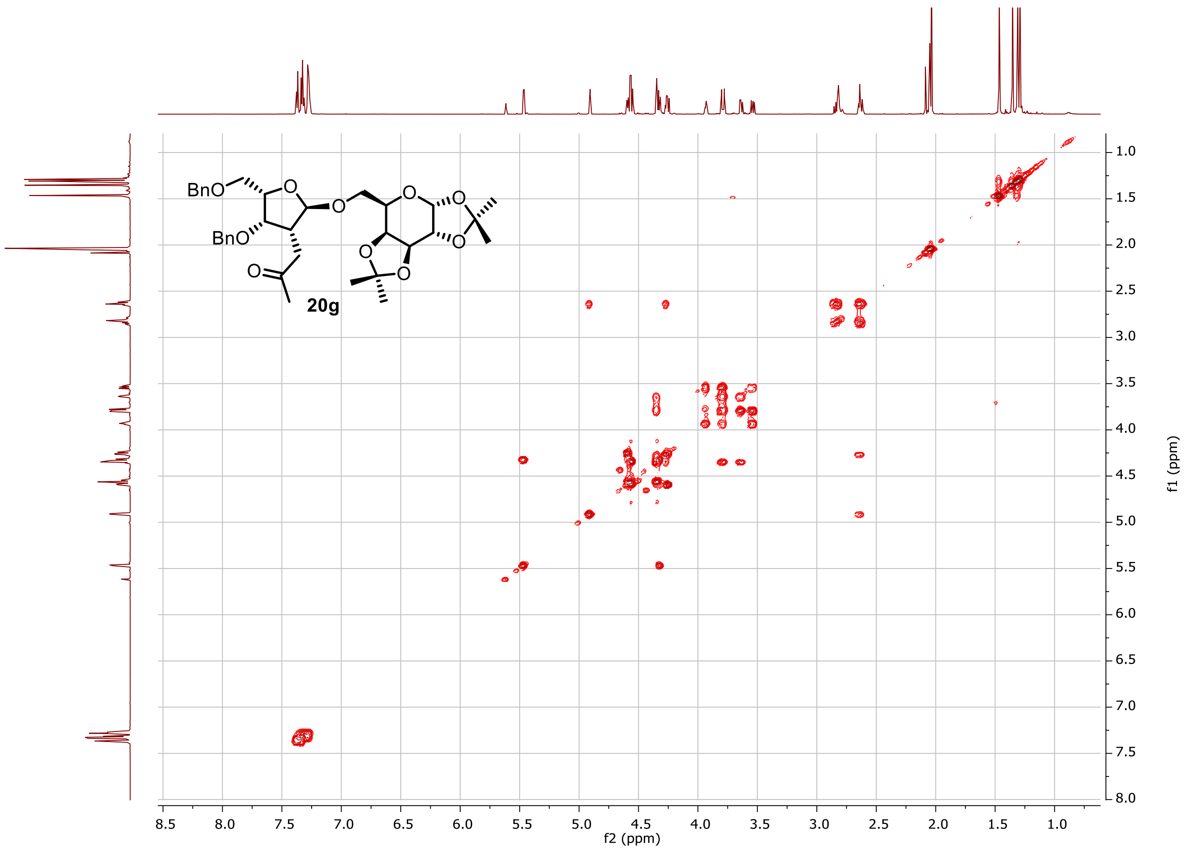

Supplementary Figure 98. COSY spectra for **20g**

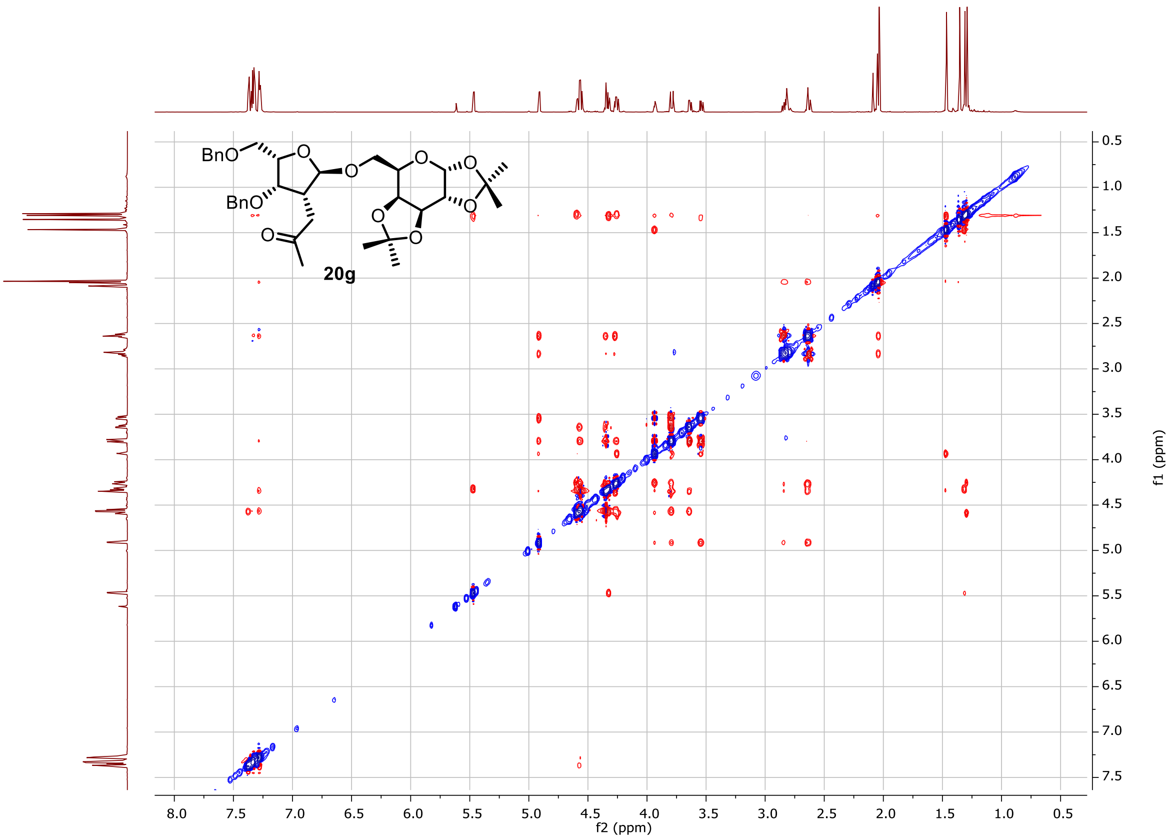

Supplementary Figure 99. NOESY spectra for **20g**

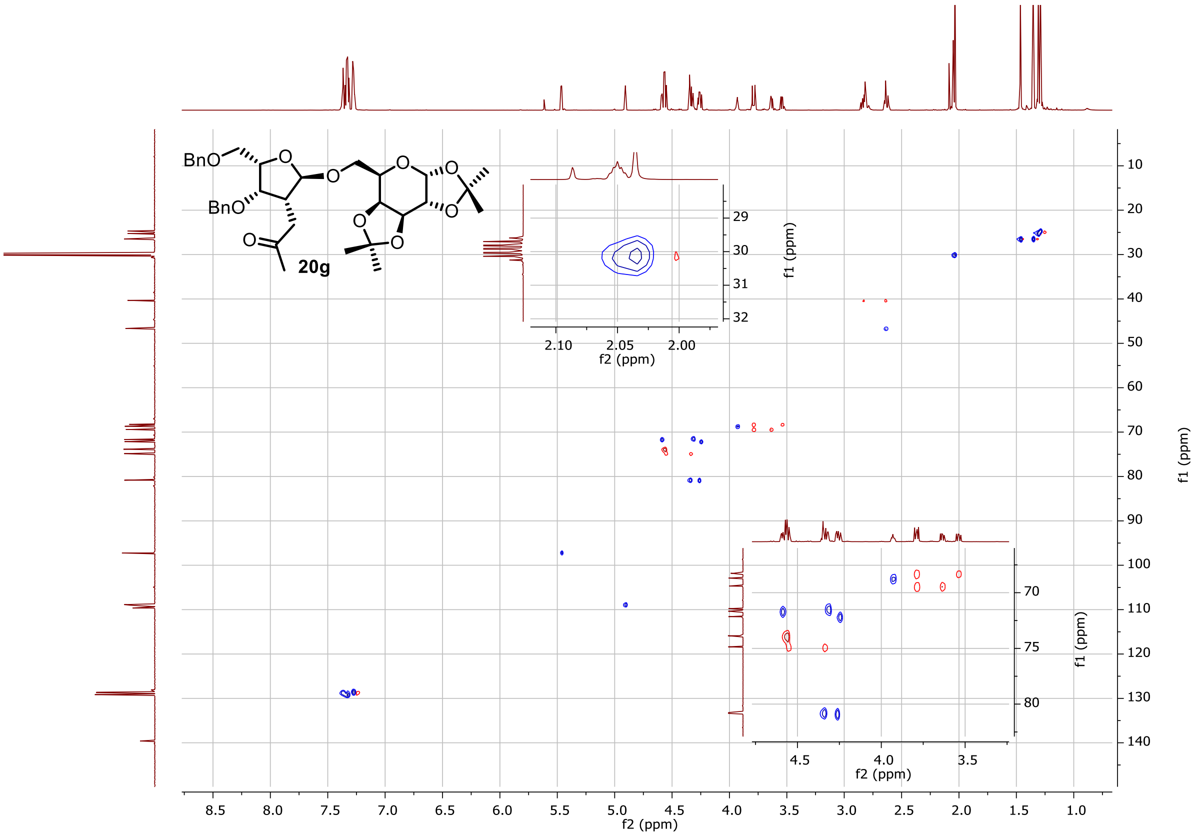

Supplementary Figure 100. HSQC spectra for **20g**

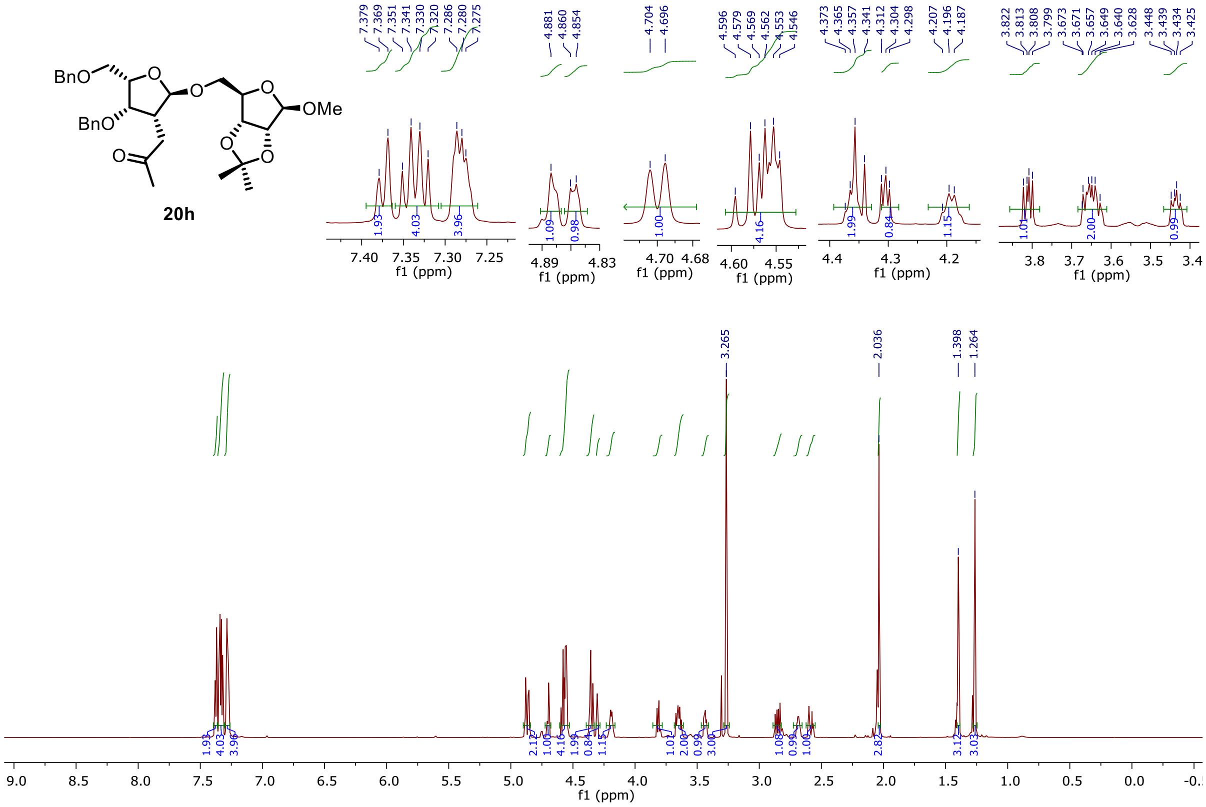

Supplementary Figure 101. ^{1}H spectra for **20h**

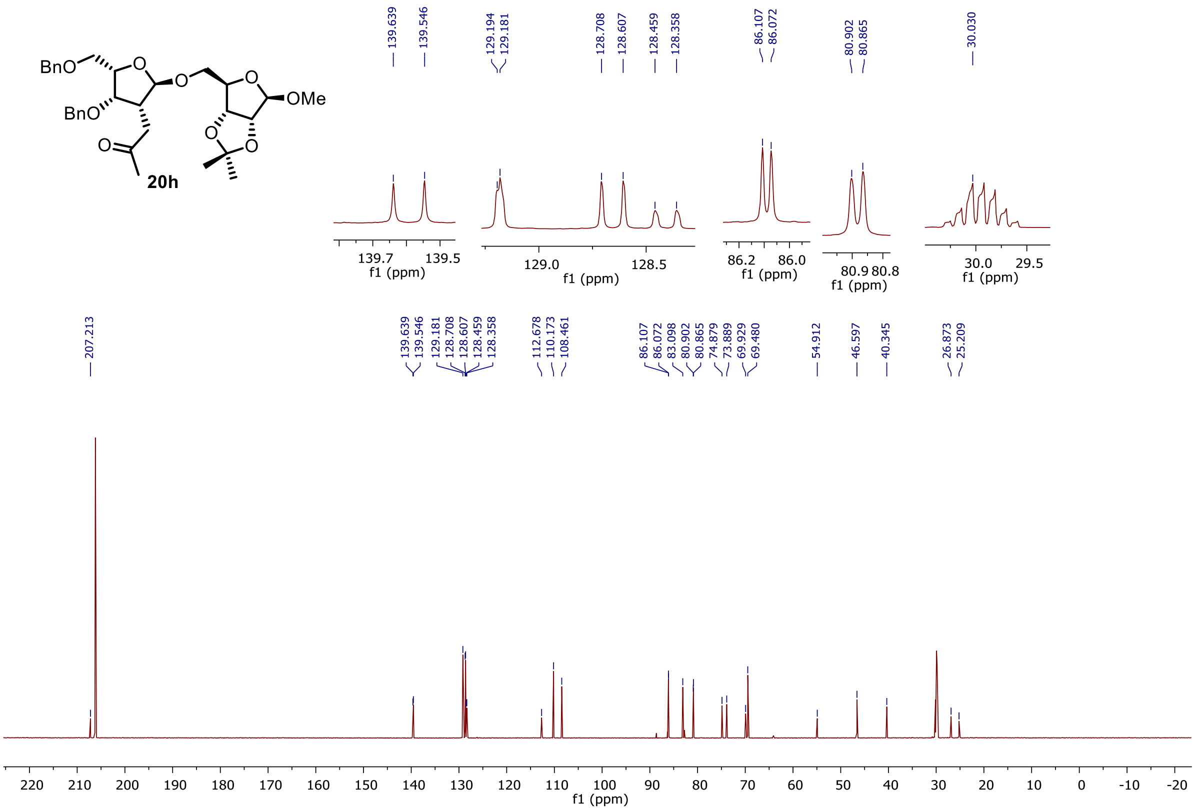

Supplementary Figure 102. ^{13}C spectra for **20h**

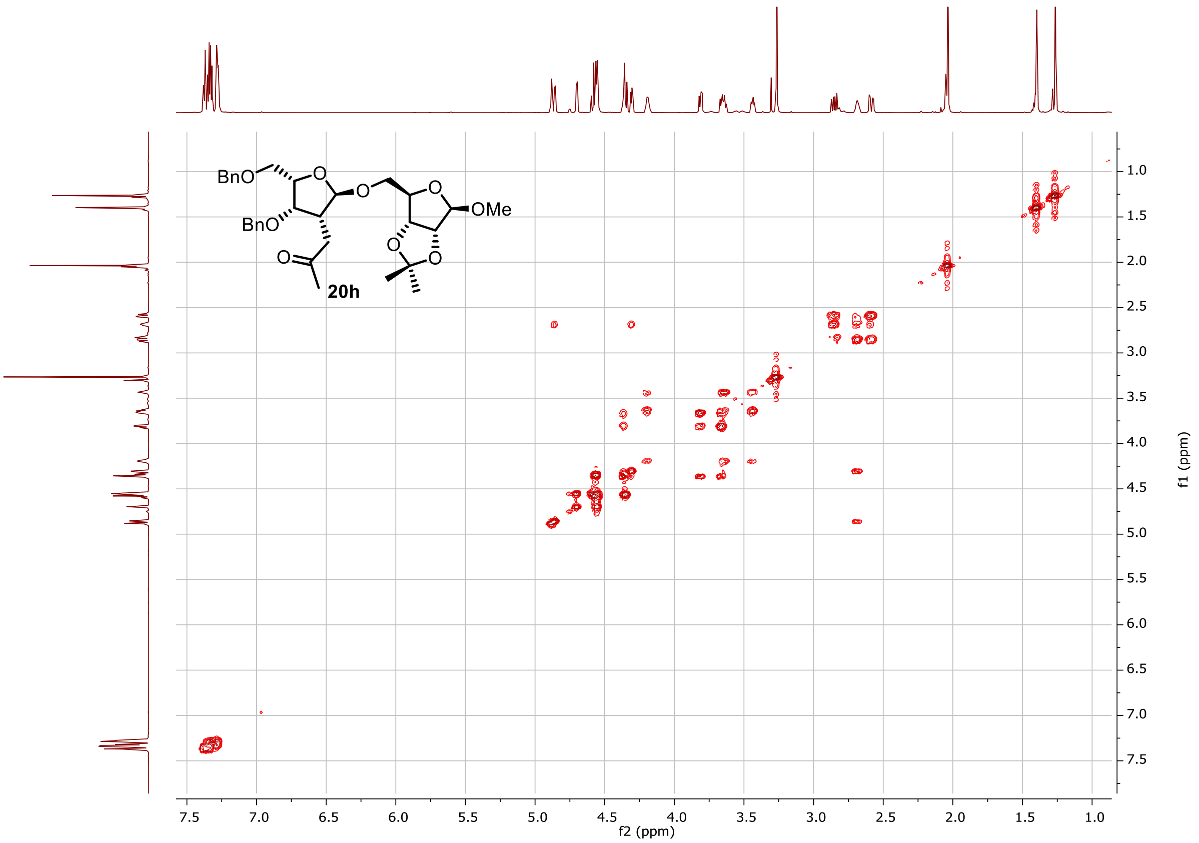

Supplementary Figure 103. COSY spectra for **20h**

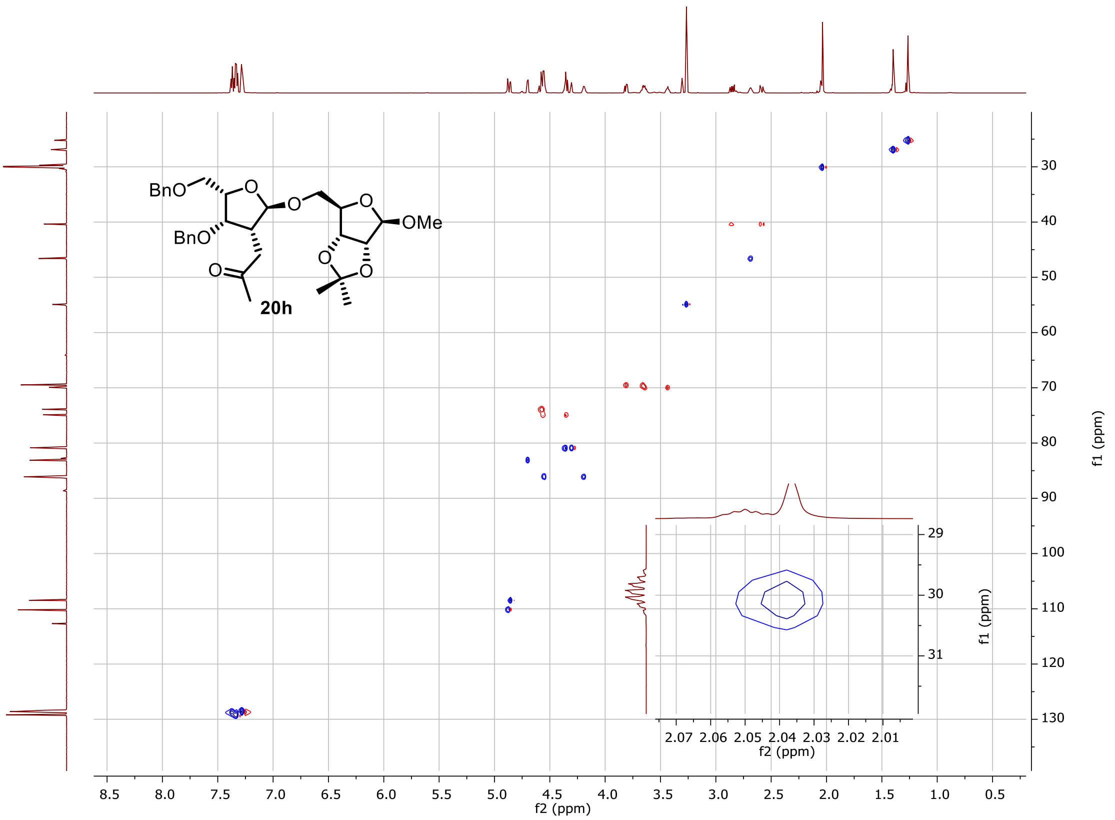

Supplementary Figure 104. HSQC spectra for **20h**

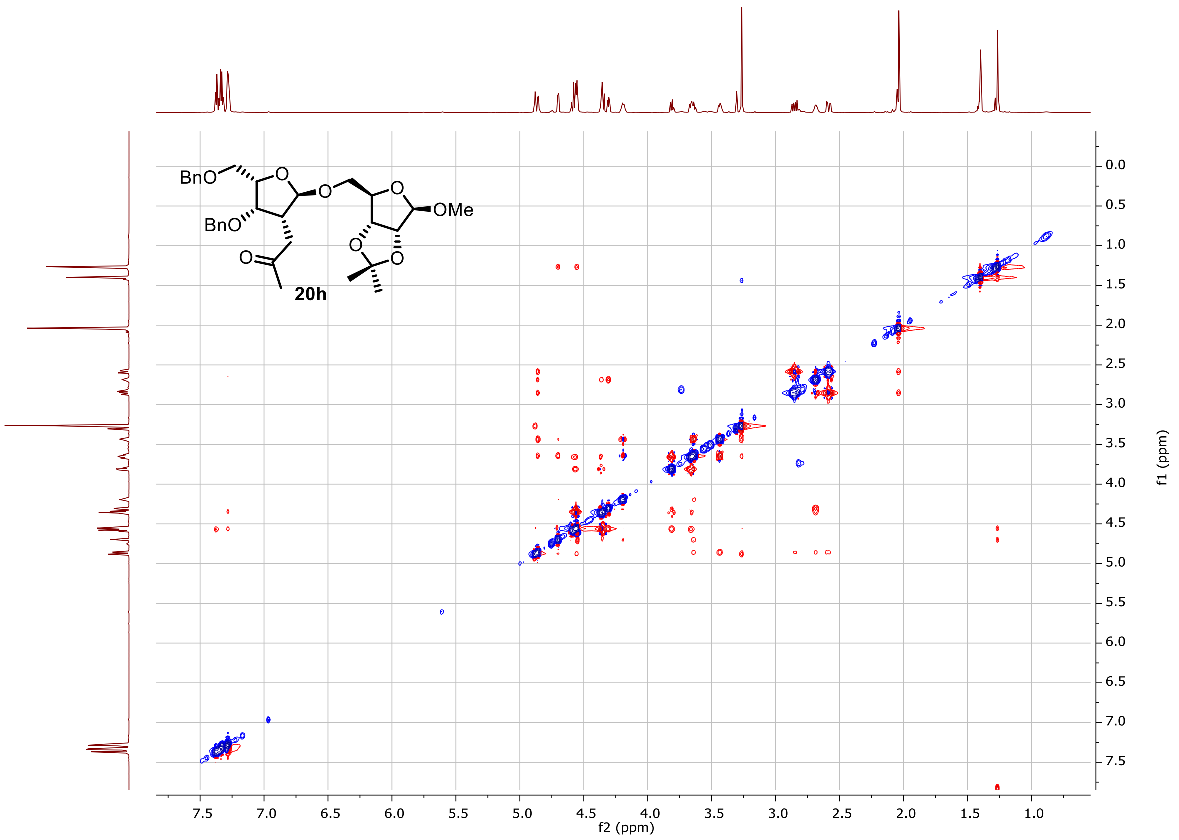

Supplementary Figure 105. NOESY spectra for **20h**

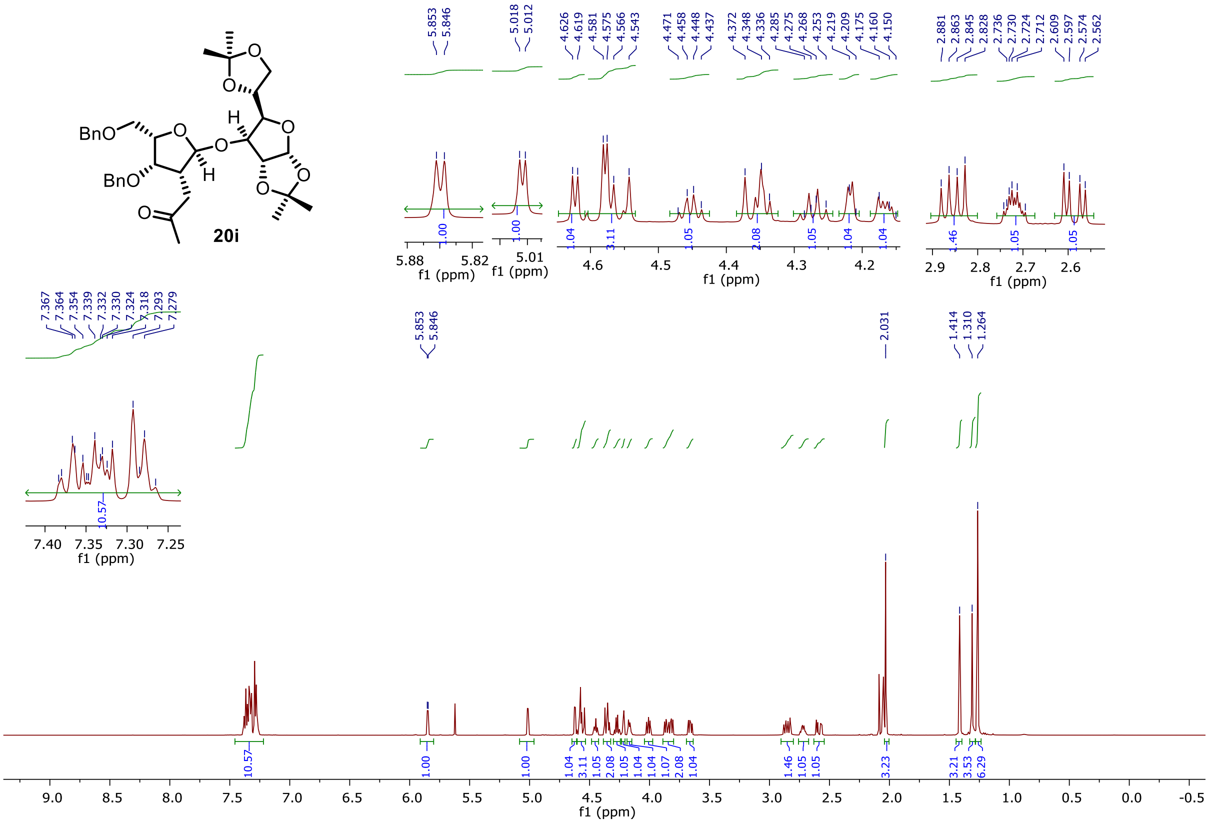

Supplementary Figure 106. ^{1}H spectra for **20i**

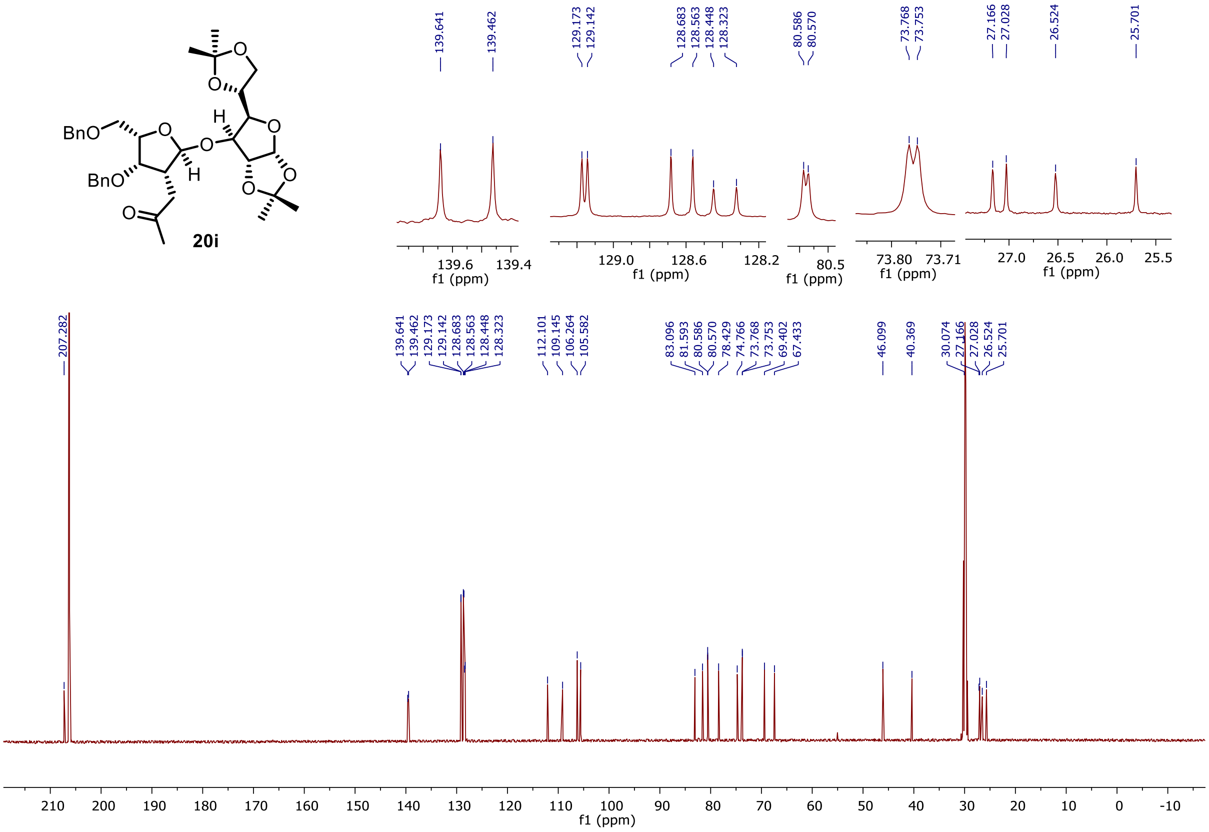

Supplementary Figure 107. ^{13}C spectra for **20i**

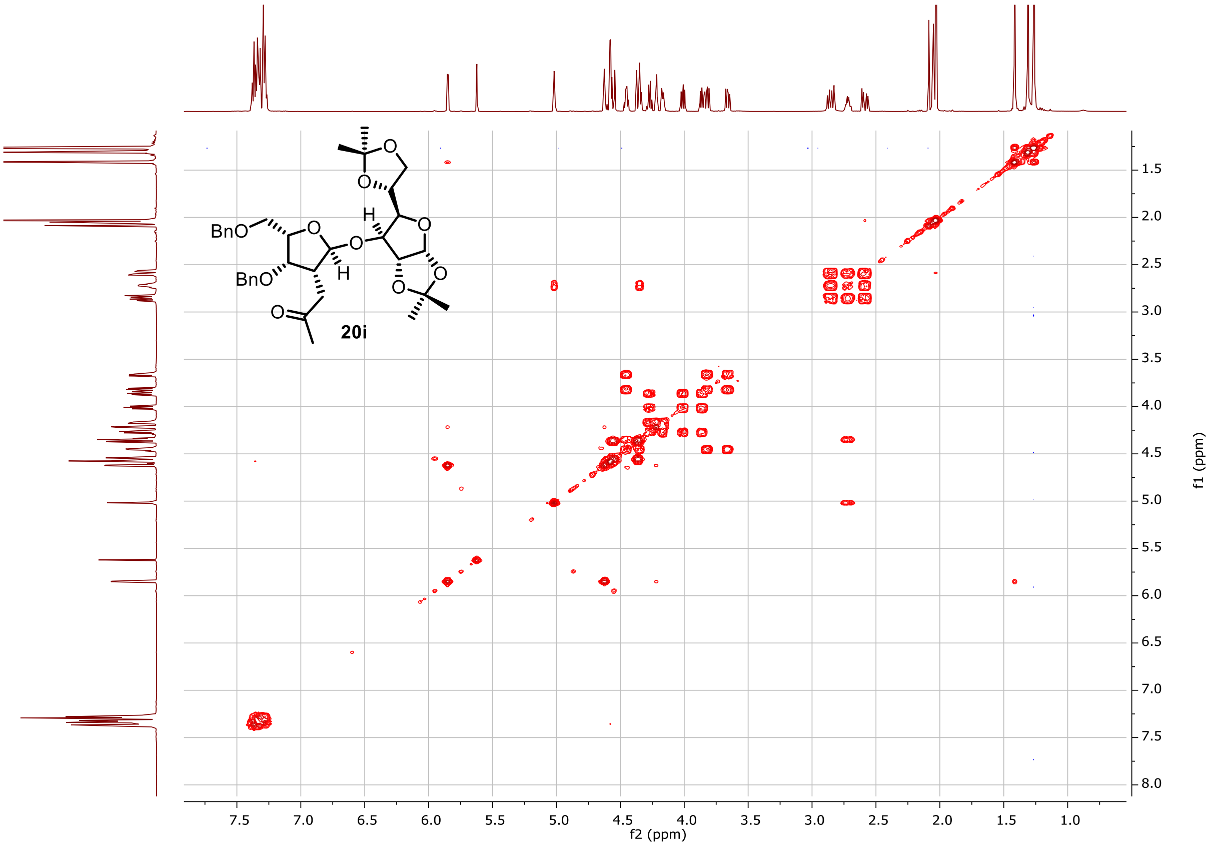

Supplementary Figure 108. COSY spectra for **20i**

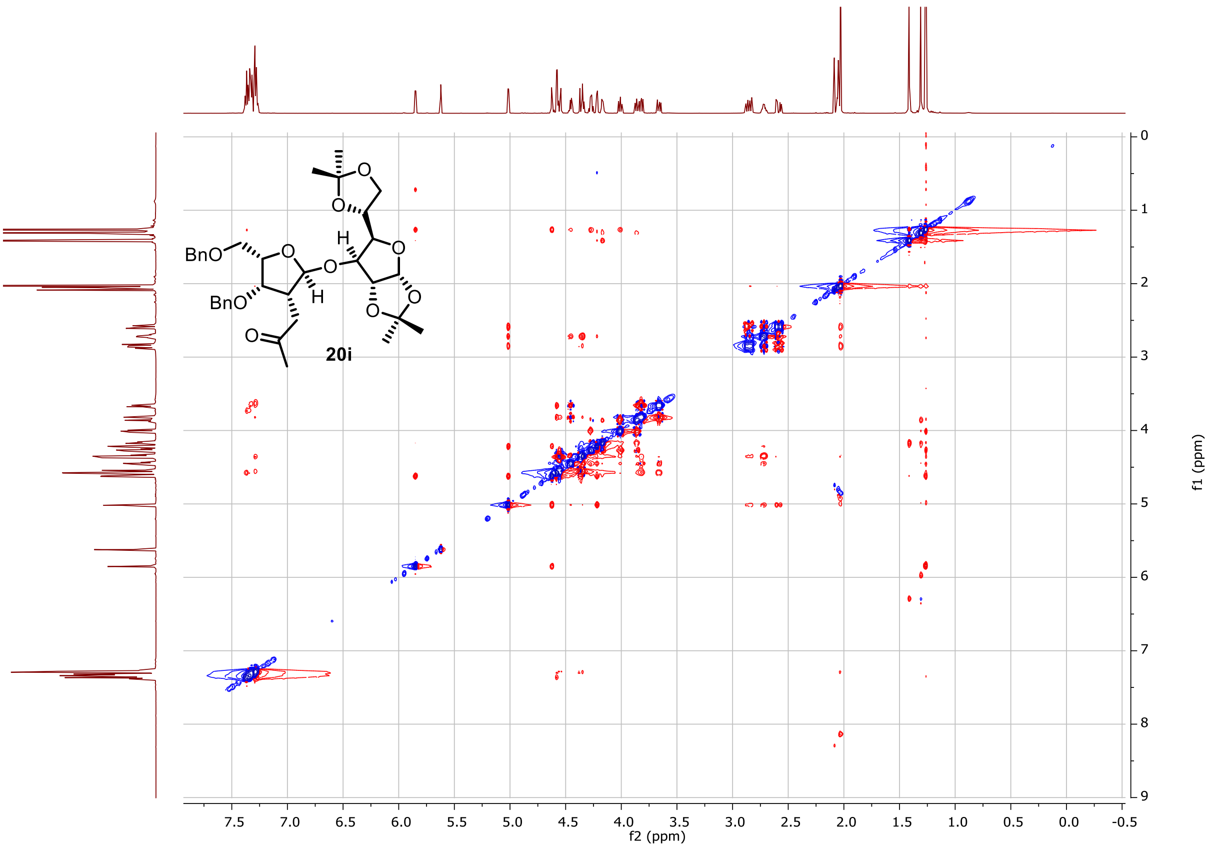

Supplementary Figure 109. NOESY spectra for **20i**

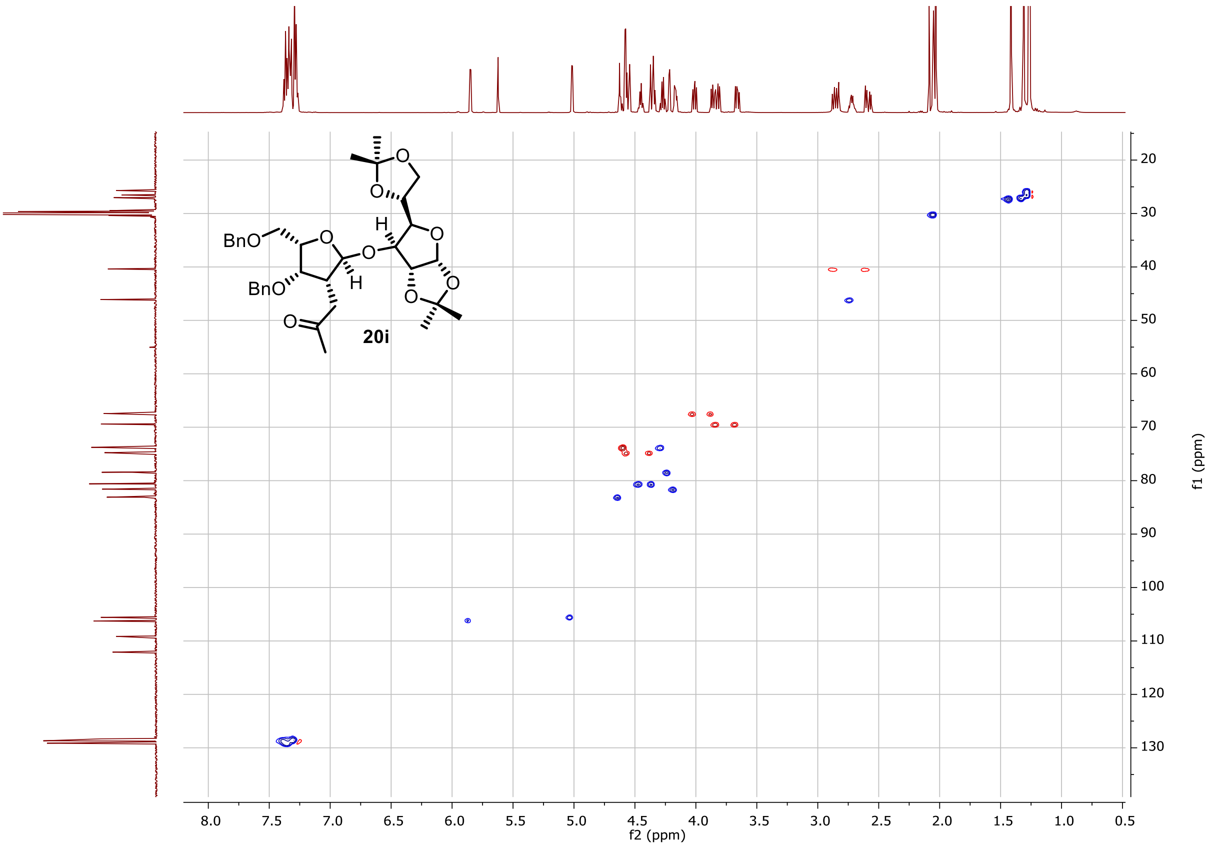

Supplementary Figure 110. HSQC spectra for **20i**

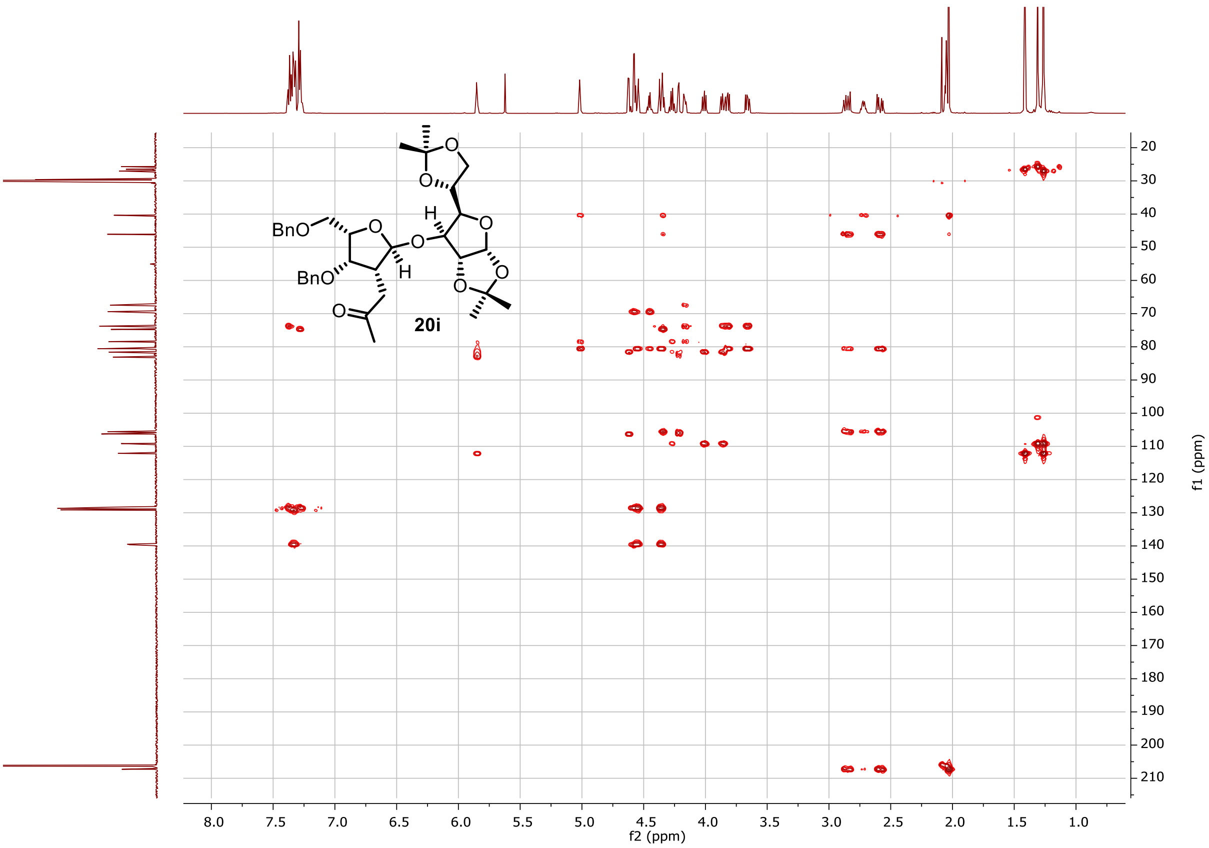

Supplementary Figure 111. HMBC spectra for **20i**

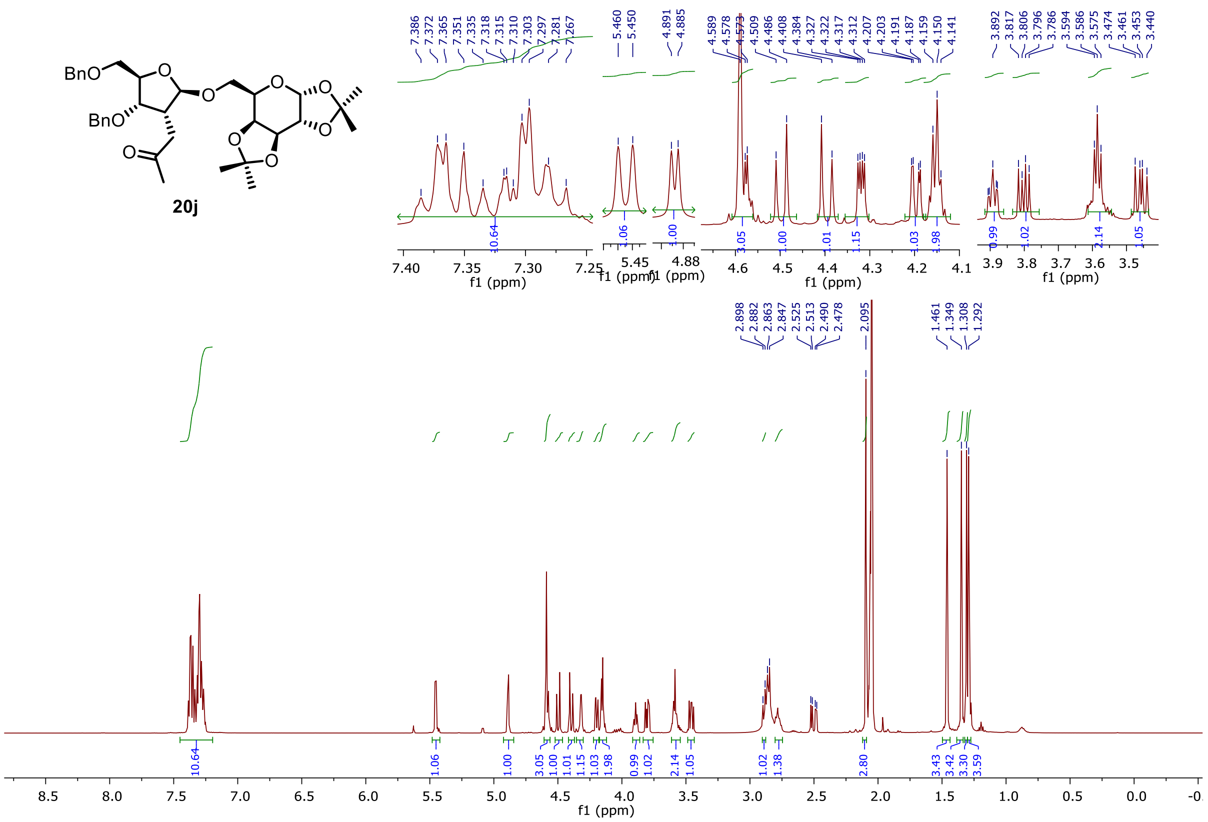

Supplementary Figure 112. ^{1}H spectra for **20j**

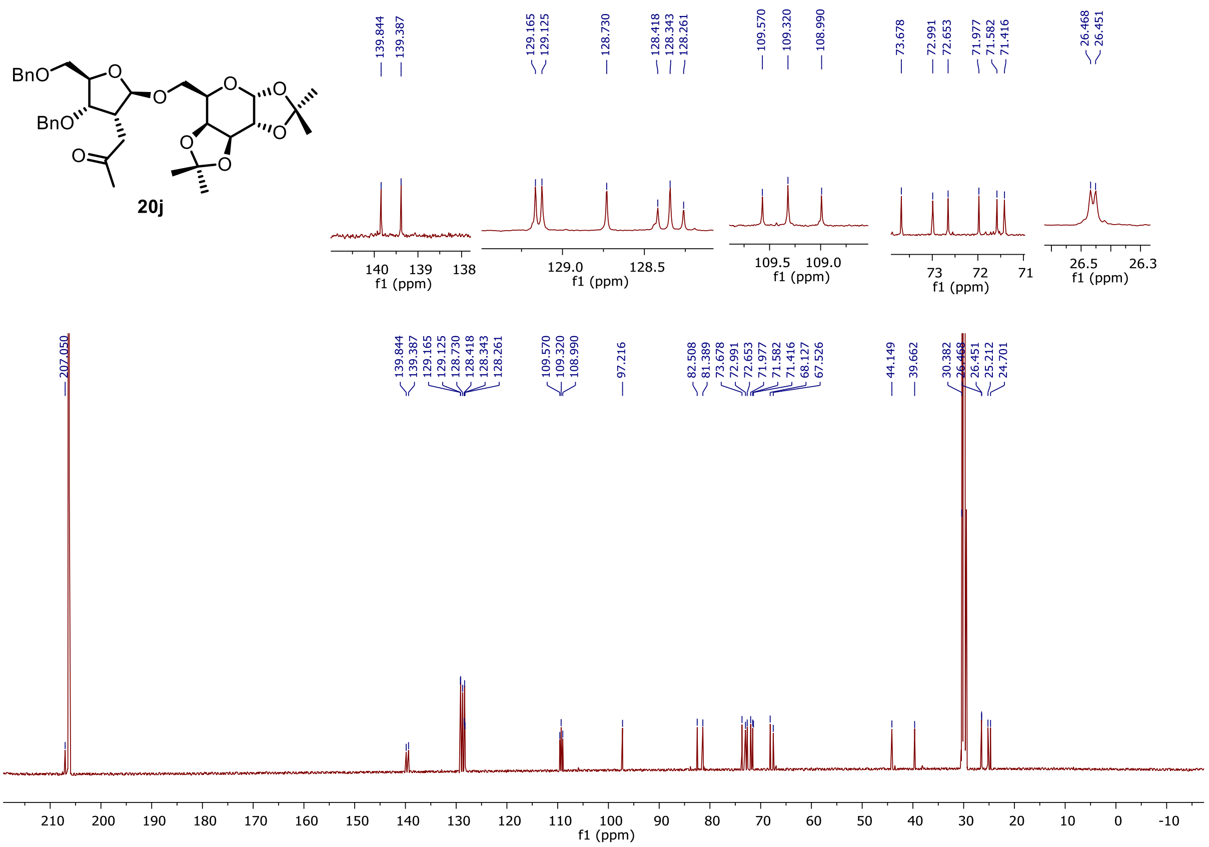

Supplementary Figure 113. ^{13}C spectra for **20j**

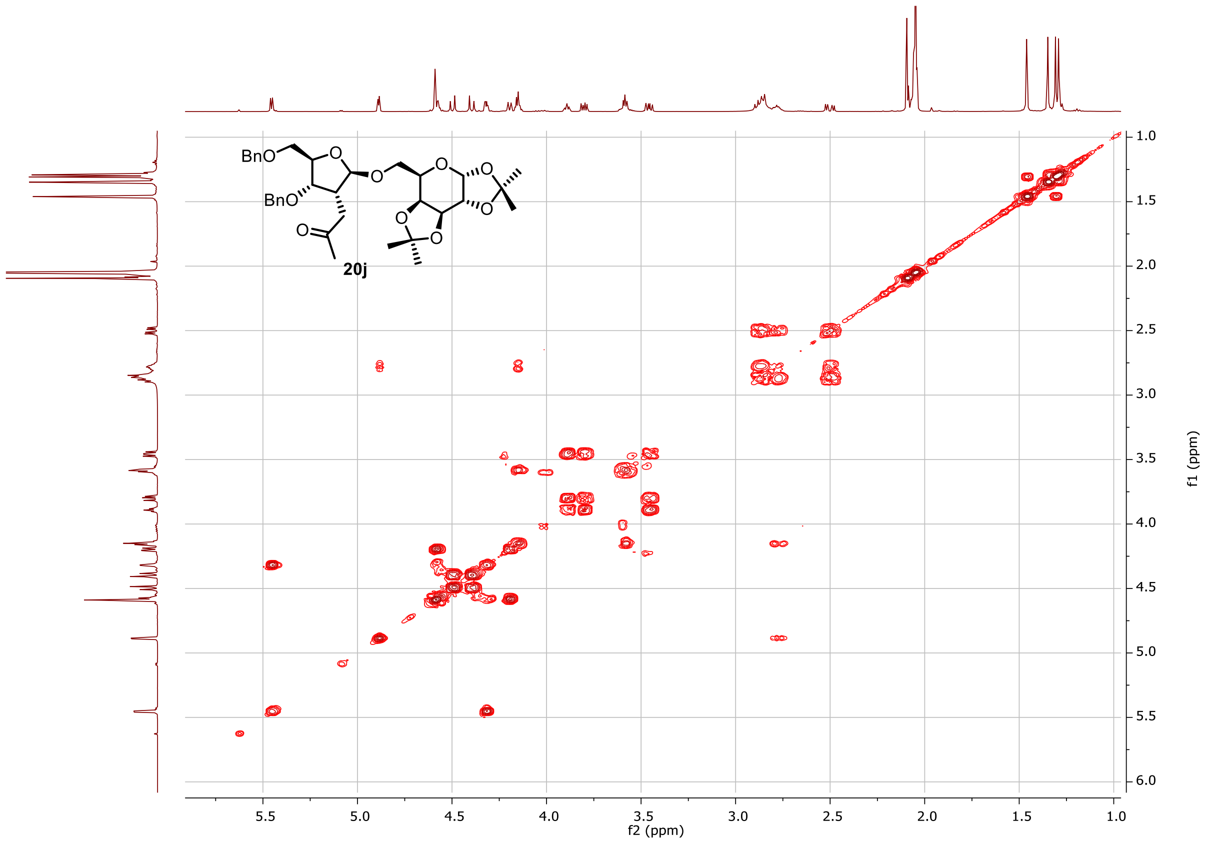

Supplementary Figure 114. COSY spectra for **20j**

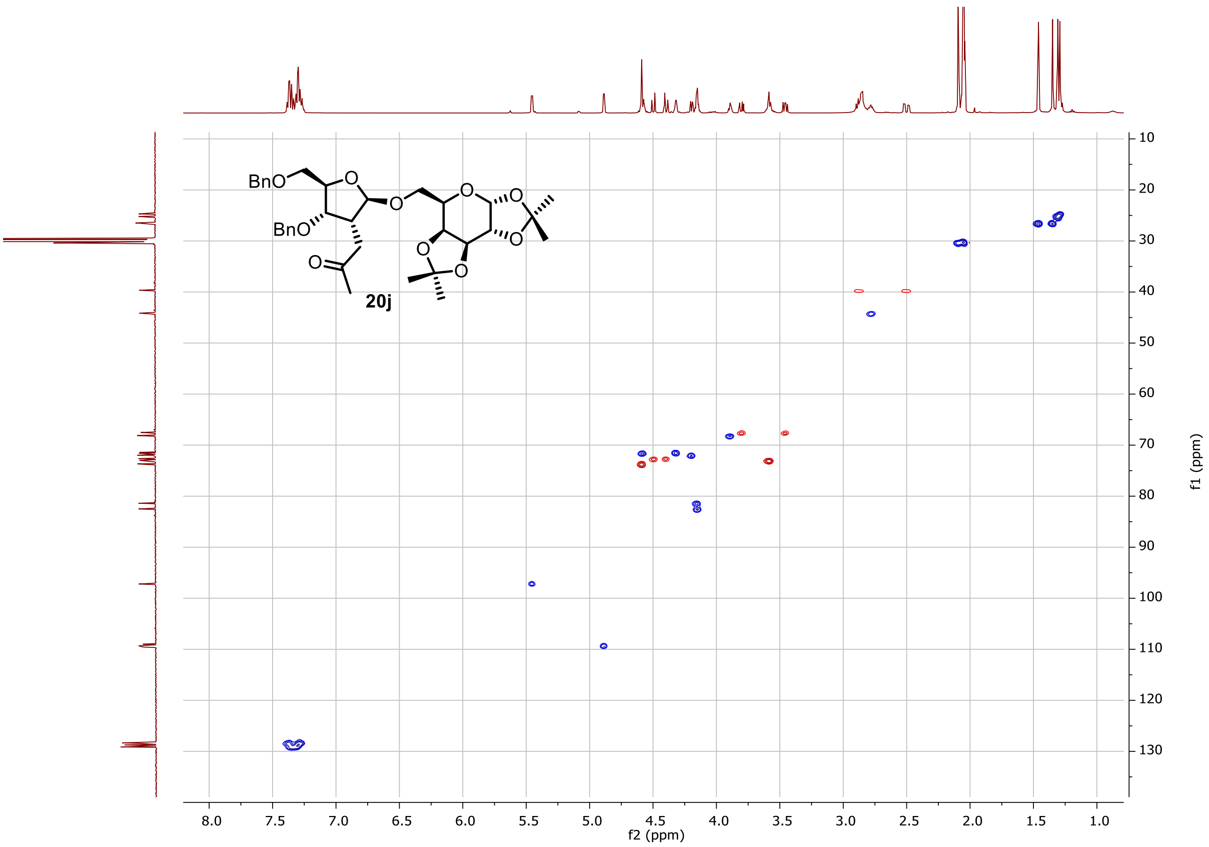

Supplementary Figure 115. HSQC spectra for **20j**

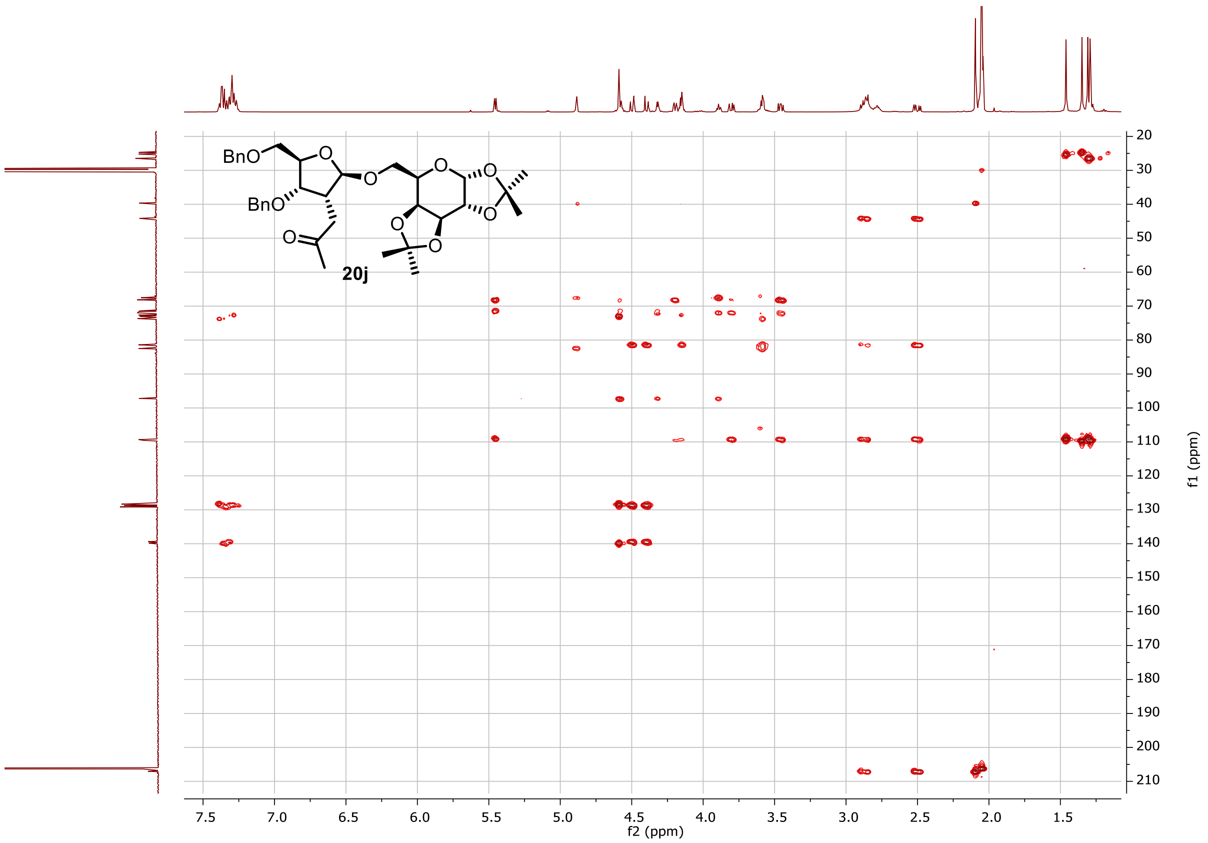

Supplementary Figure 116. HMBC spectra for **20j**

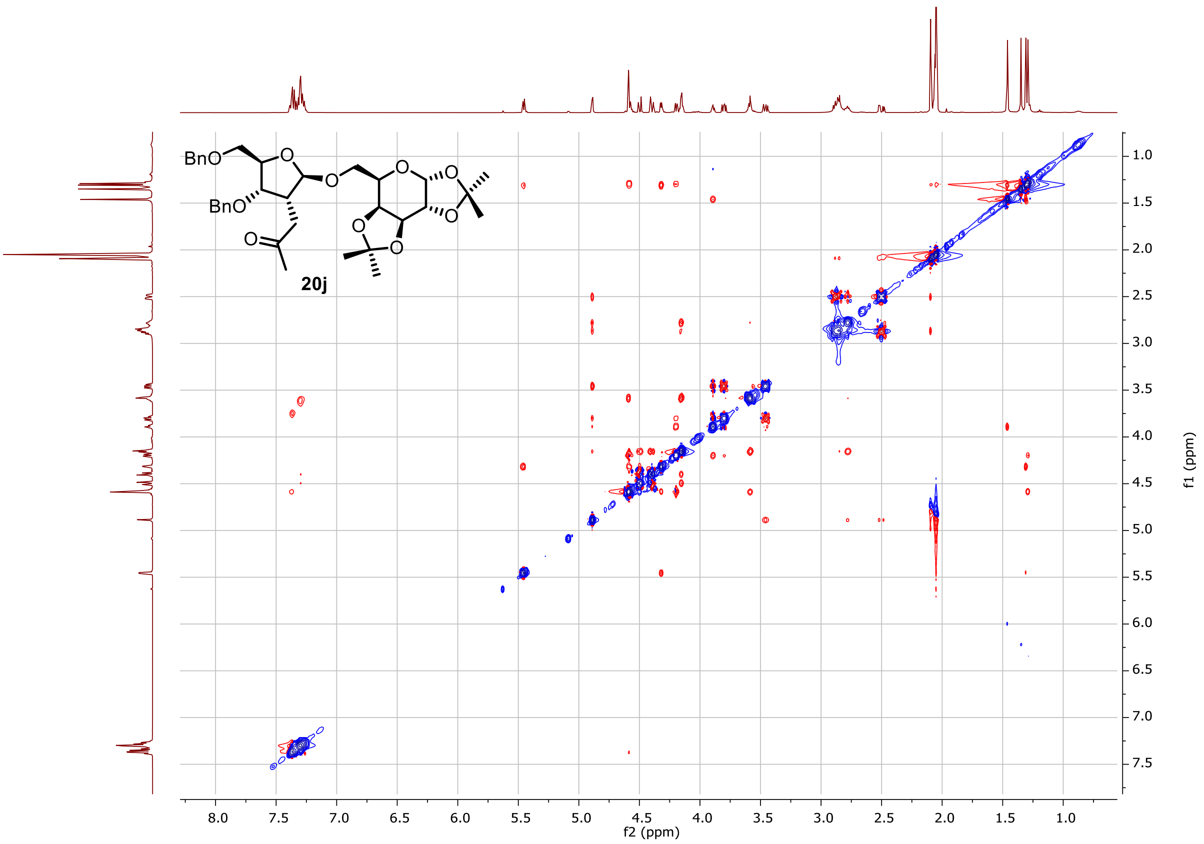

Supplementary Figure 117. NOESY spectra for **20j**

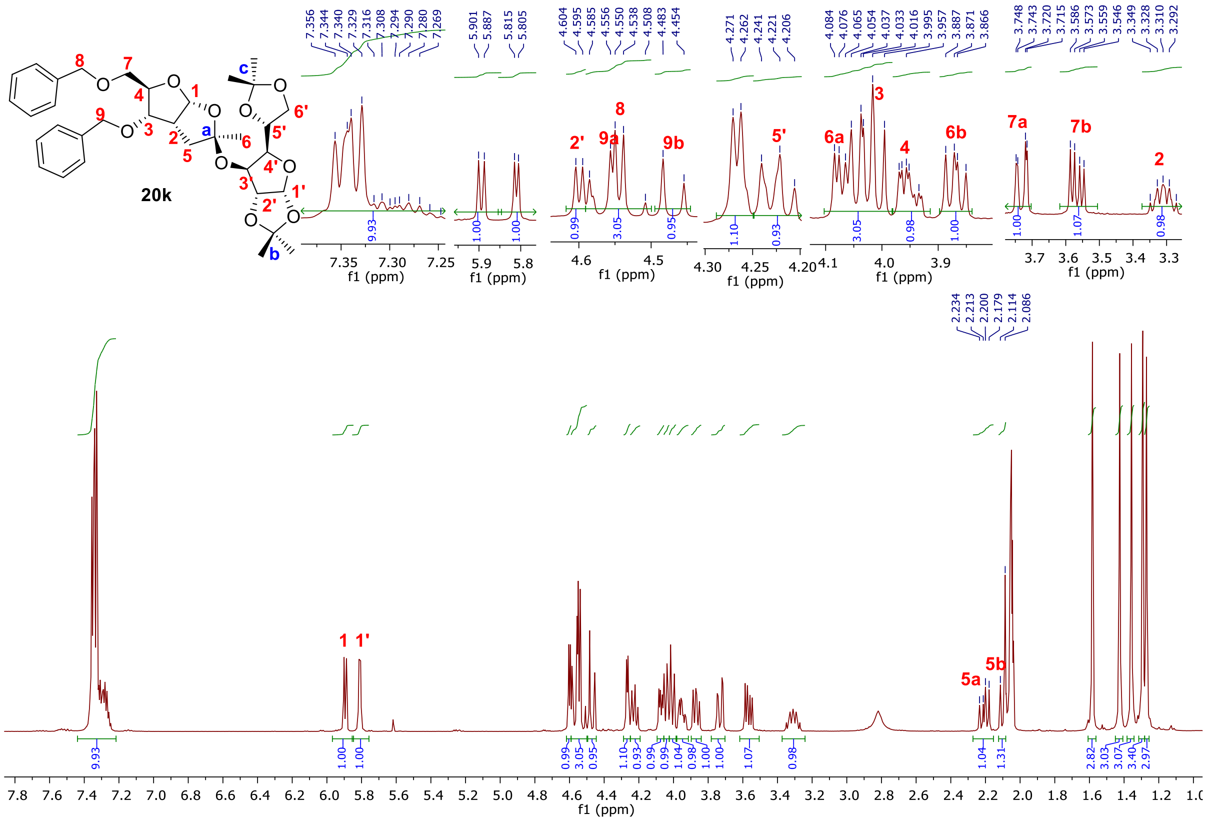

Supplementary Figure 118. ^{1}H spectra for **20k**

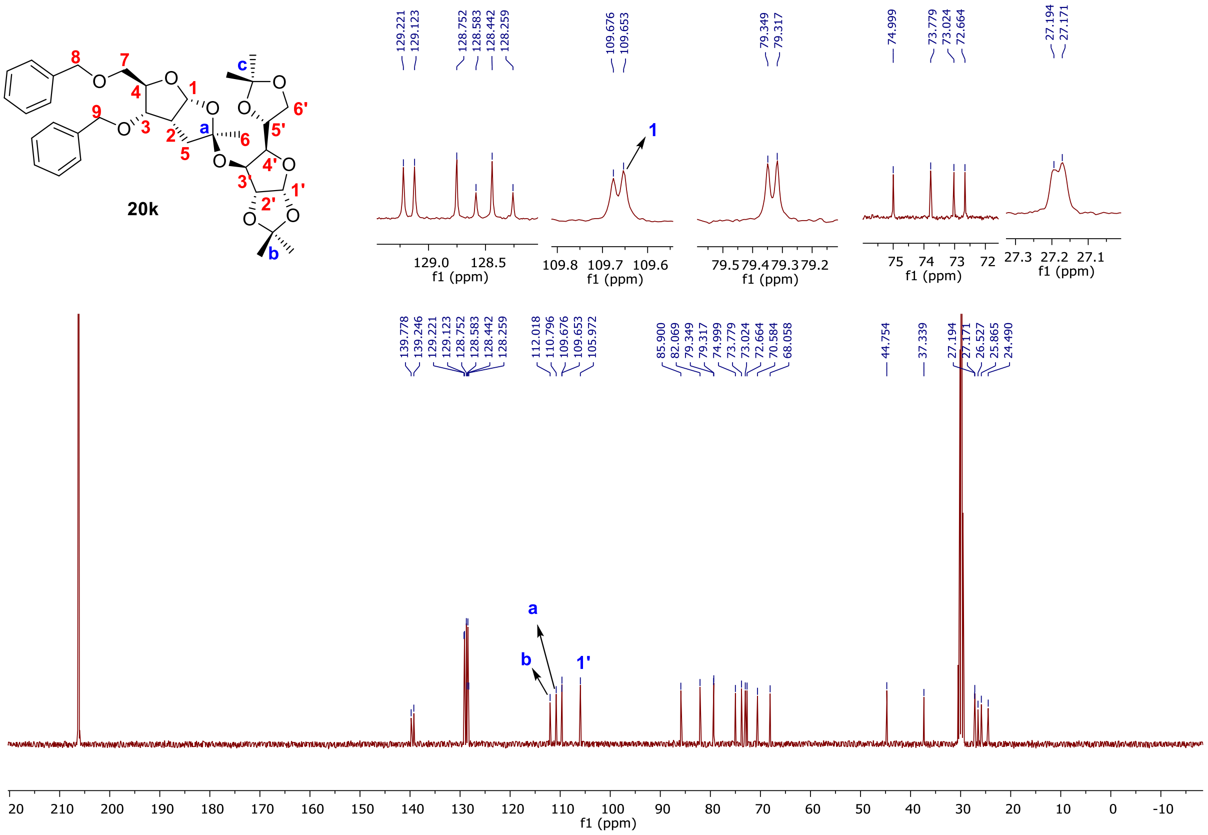

Supplementary Figure 119. ^{13}C spectra for **20k**

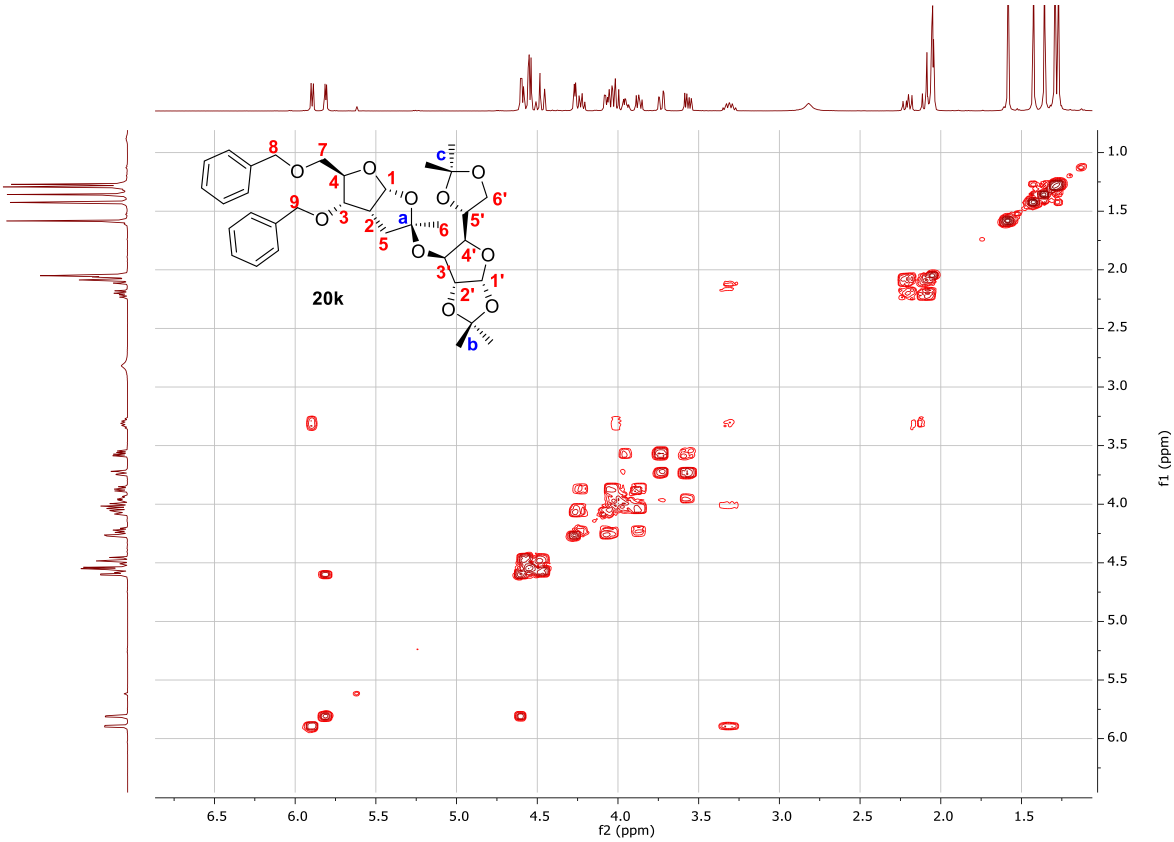

Supplementary Figure 120. COSY spectra for **20k**

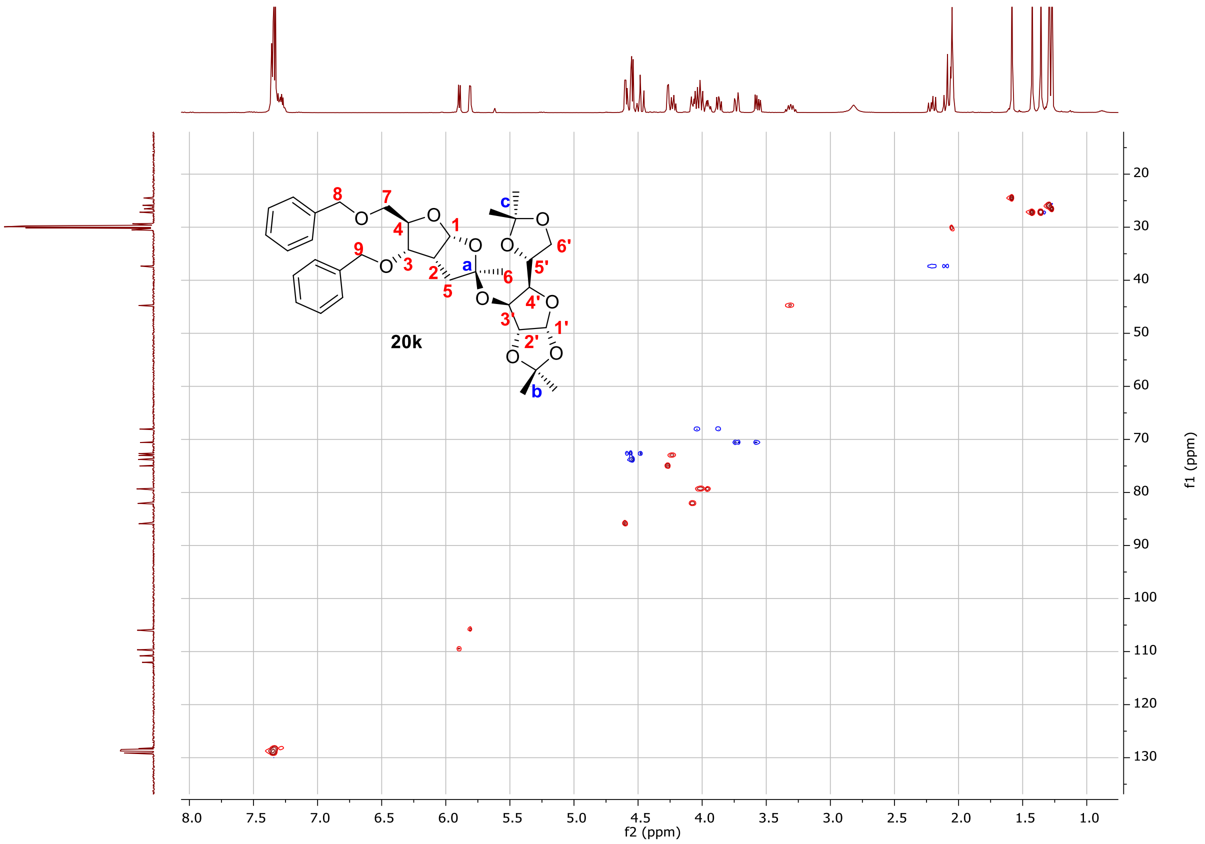

Supplementary Figure 121. HSQC spectra for **20k**

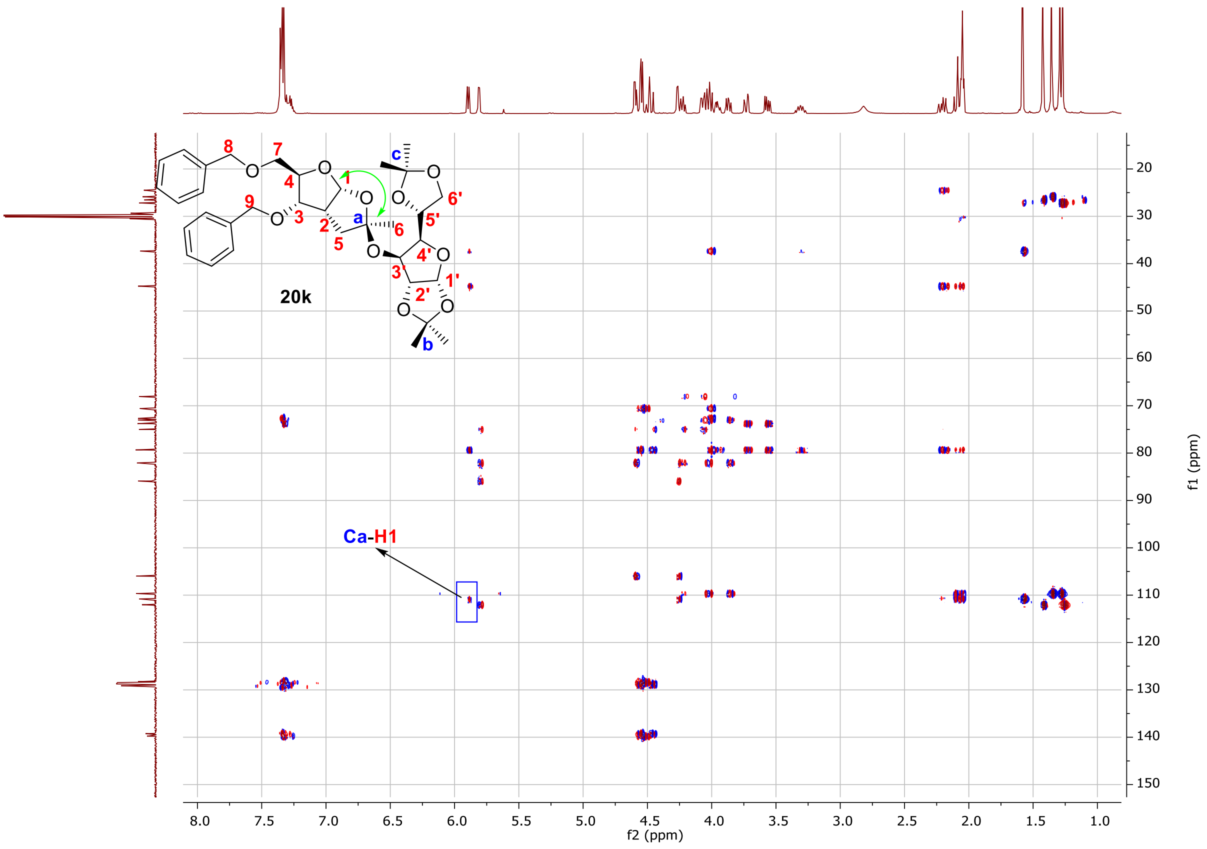

Supplementary Figure 122. HMBC spectra for **20k**

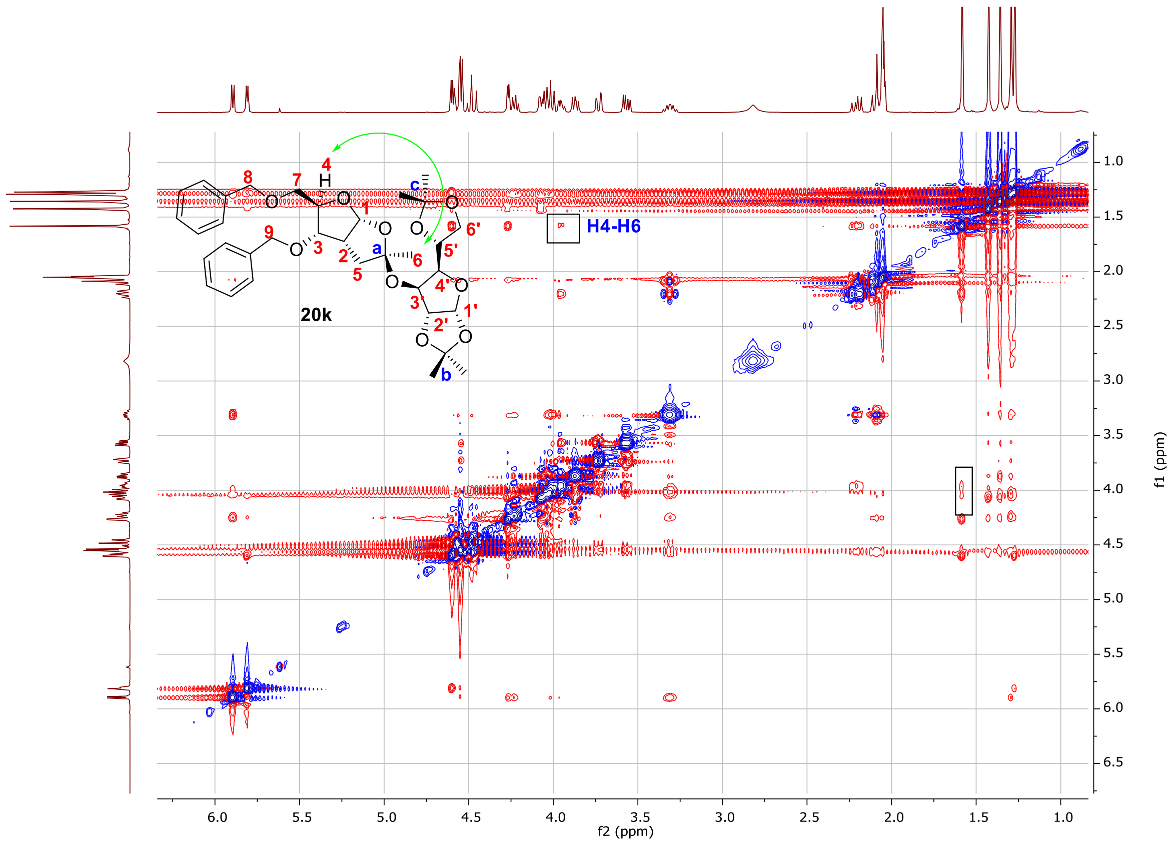

Supplementary Figure 123. NOESY spectra for **20k**

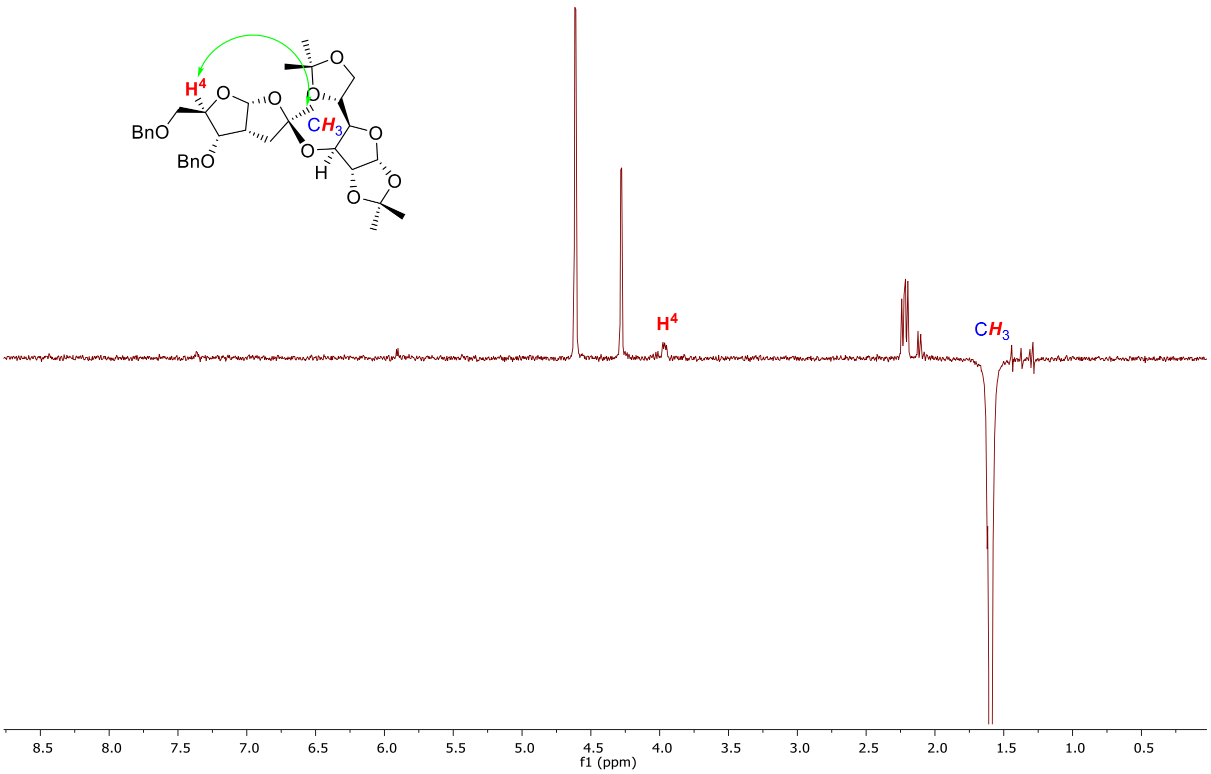

Supplementary Figure 124. 1D NOE spectra for **20k**

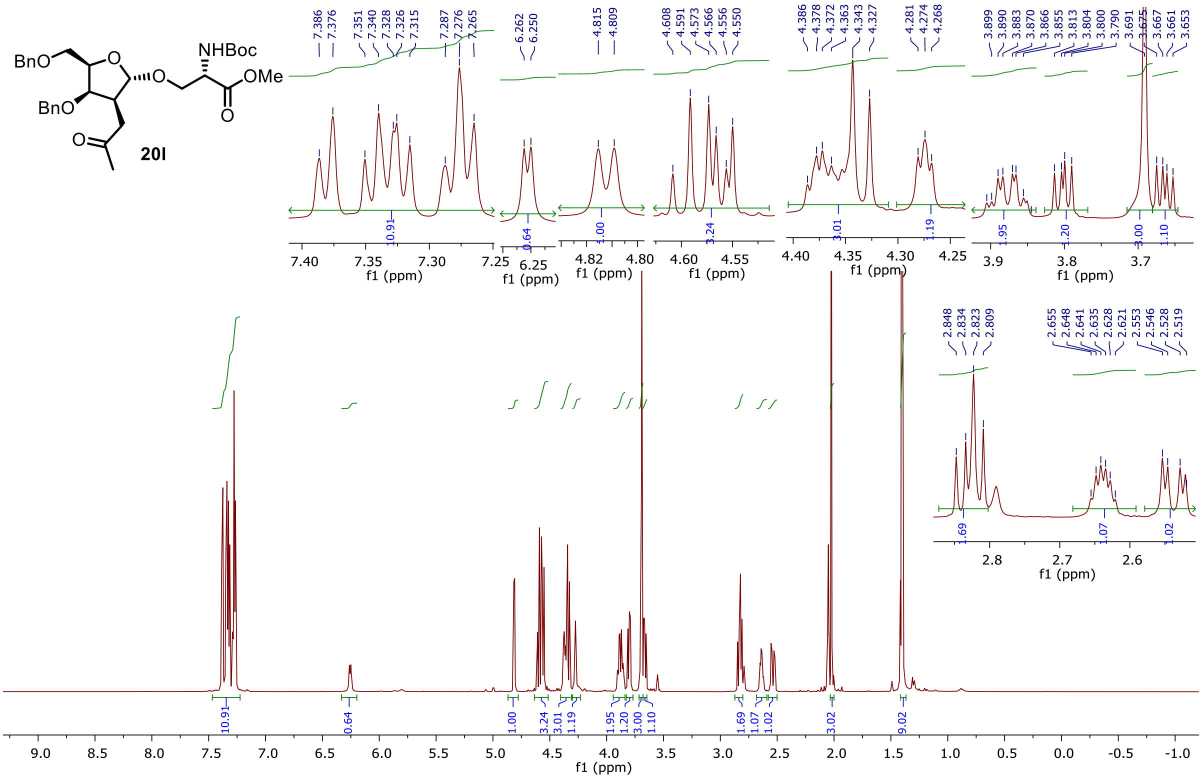

Supplementary Figure 125. ^{1}H spectra for **20l**

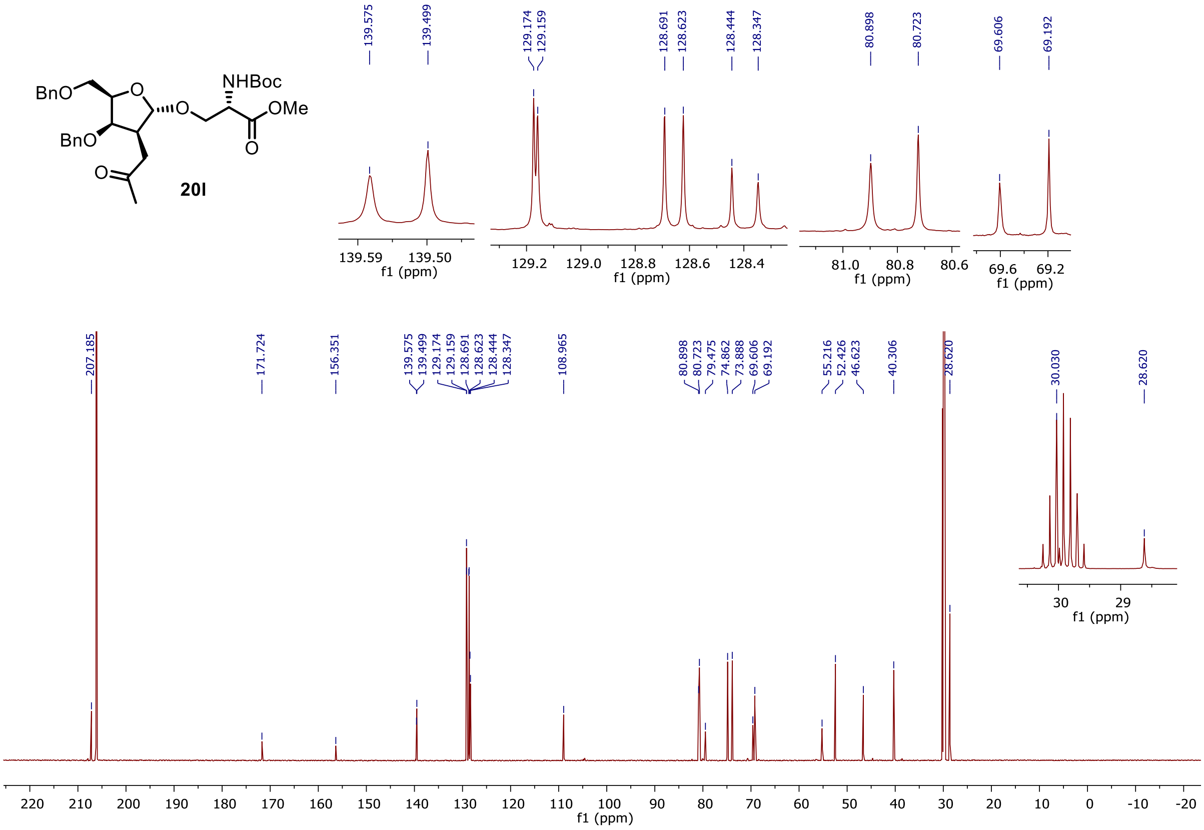

Supplementary Figure 126. ^{13}C spectra for **20l**

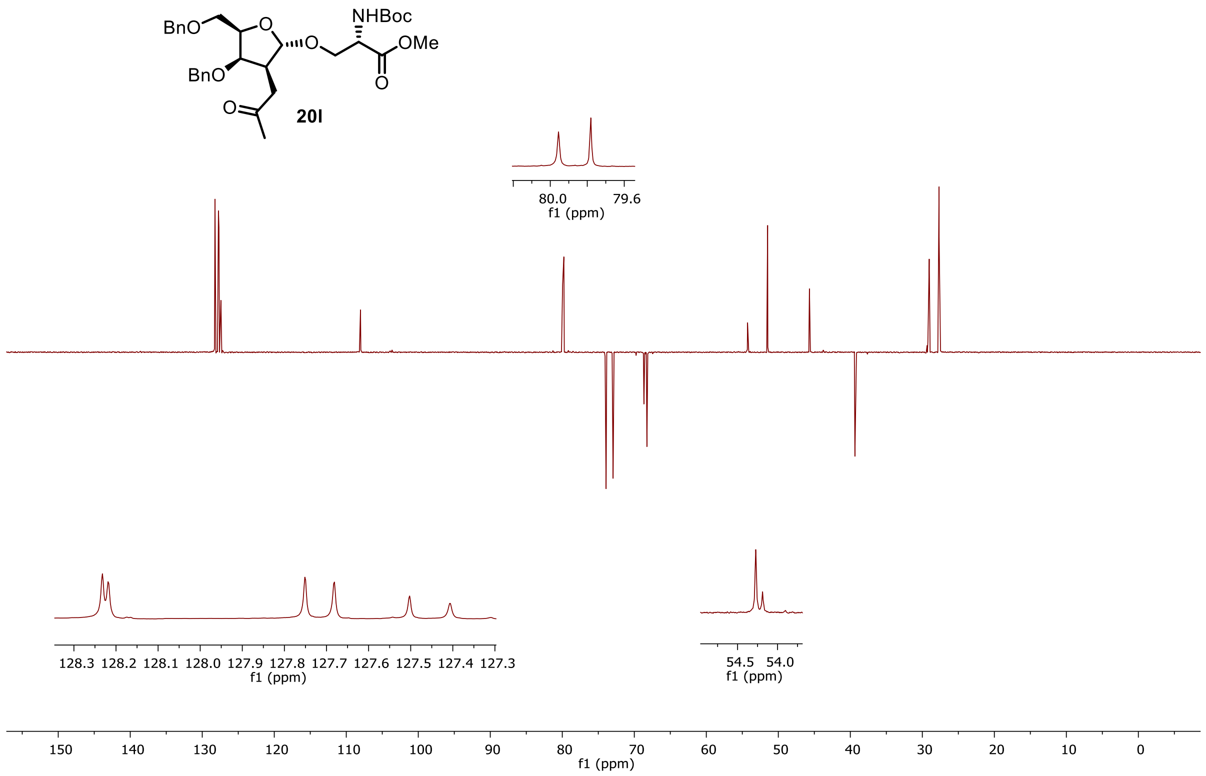

Supplementary Figure 127. DEPT spectra for **20l**

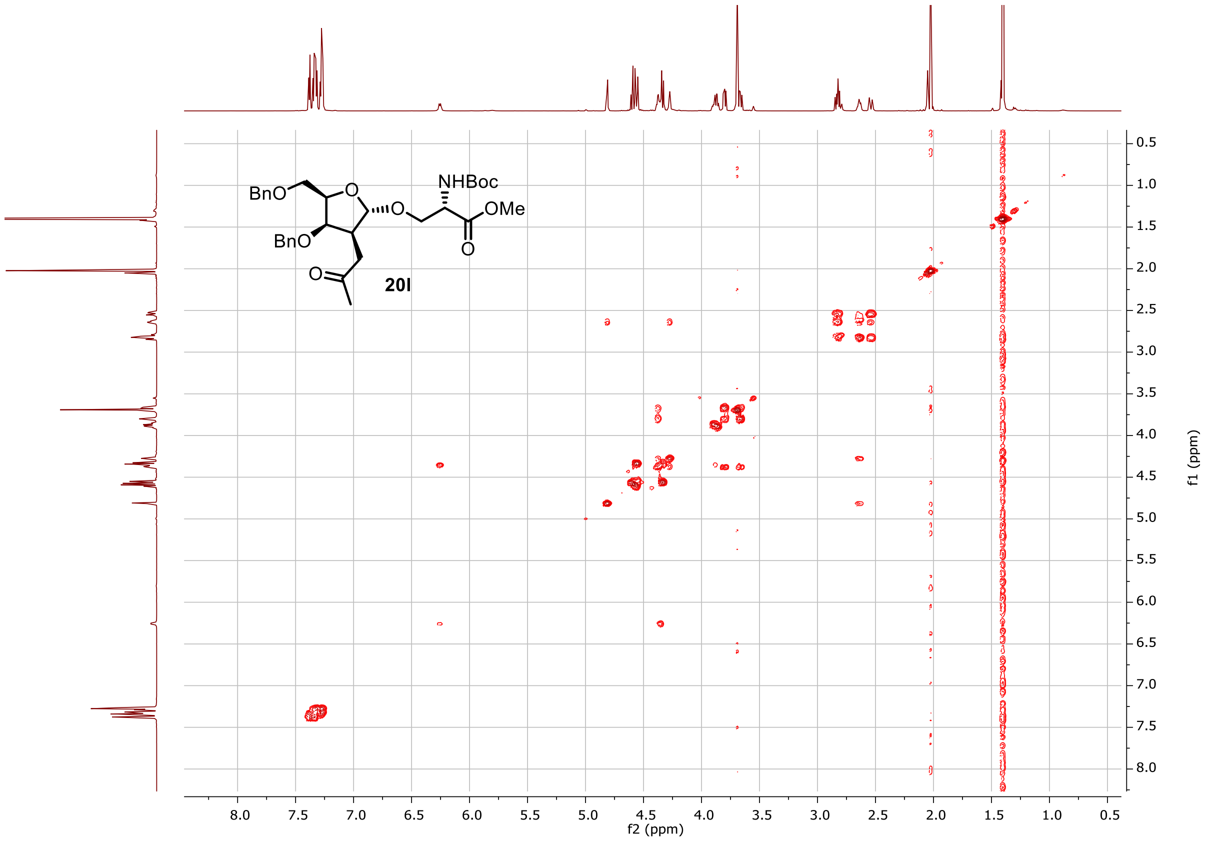

Supplementary Figure 128. COSY spectra for **20l**

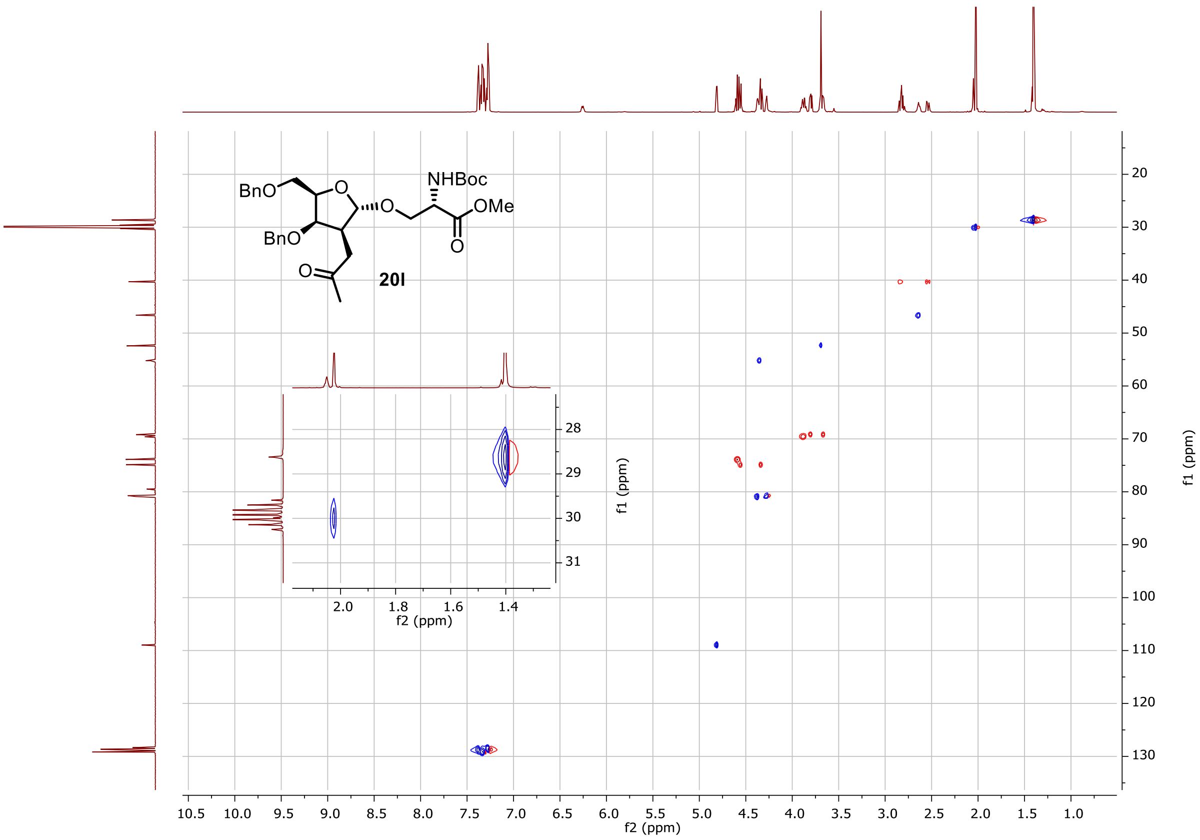

Supplementary Figure 129. HSQC spectra for **20l**

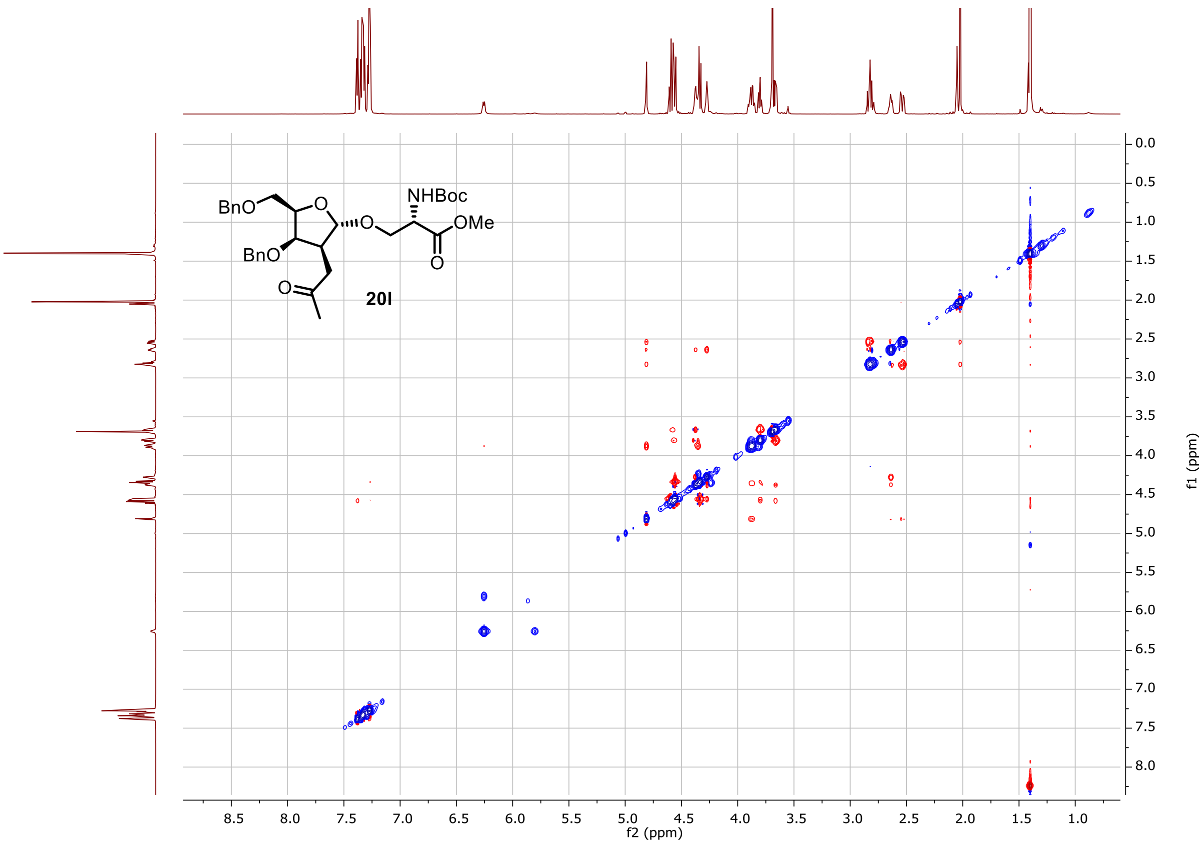

Supplementary Figure 130. NOESY spectra for **20l**

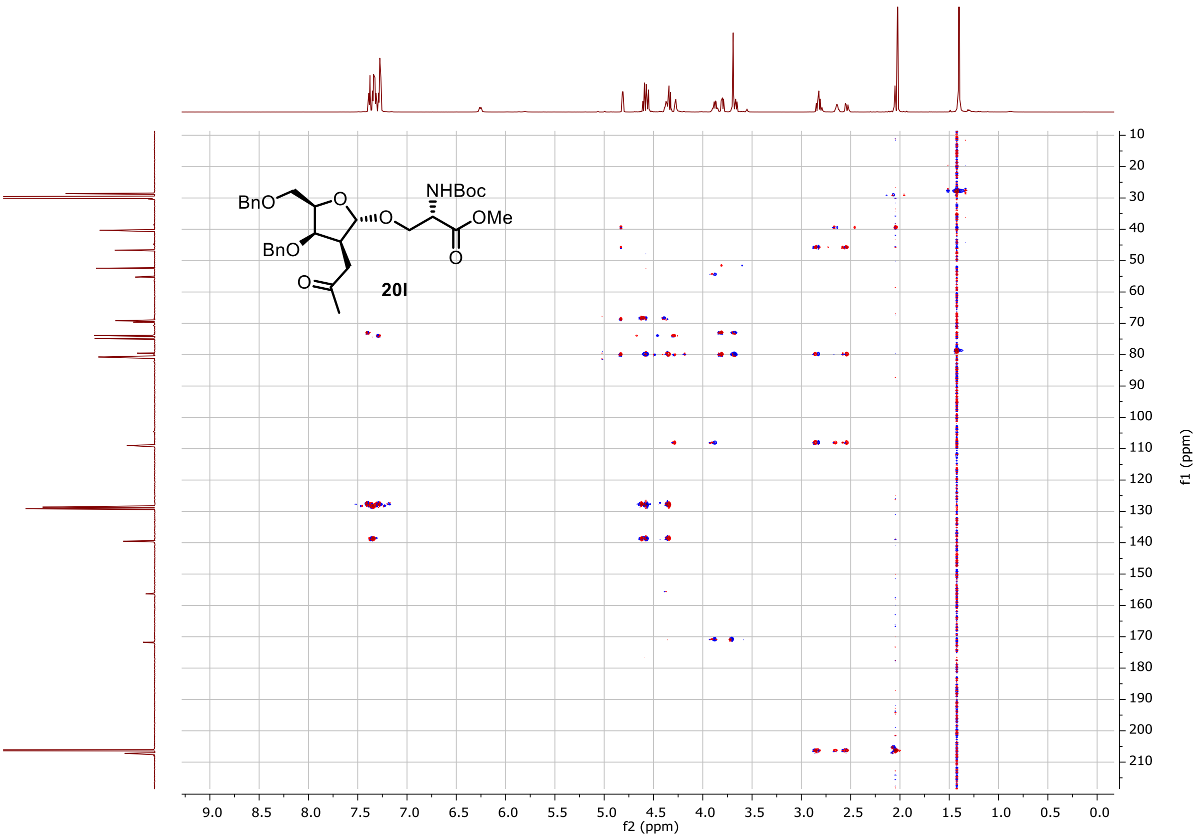

Supplementary Figure 131. HMBC spectra for **20l**

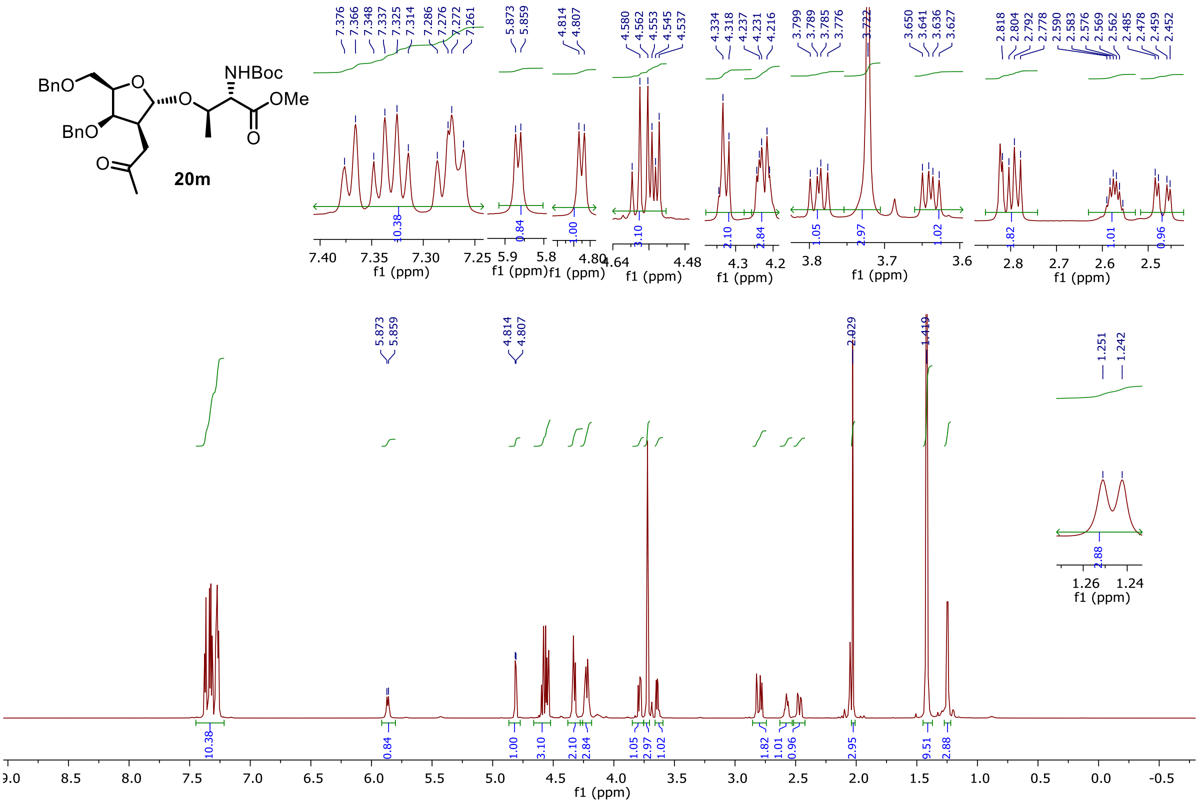

Supplementary Figure 132. ^{1}H spectra for **20m**

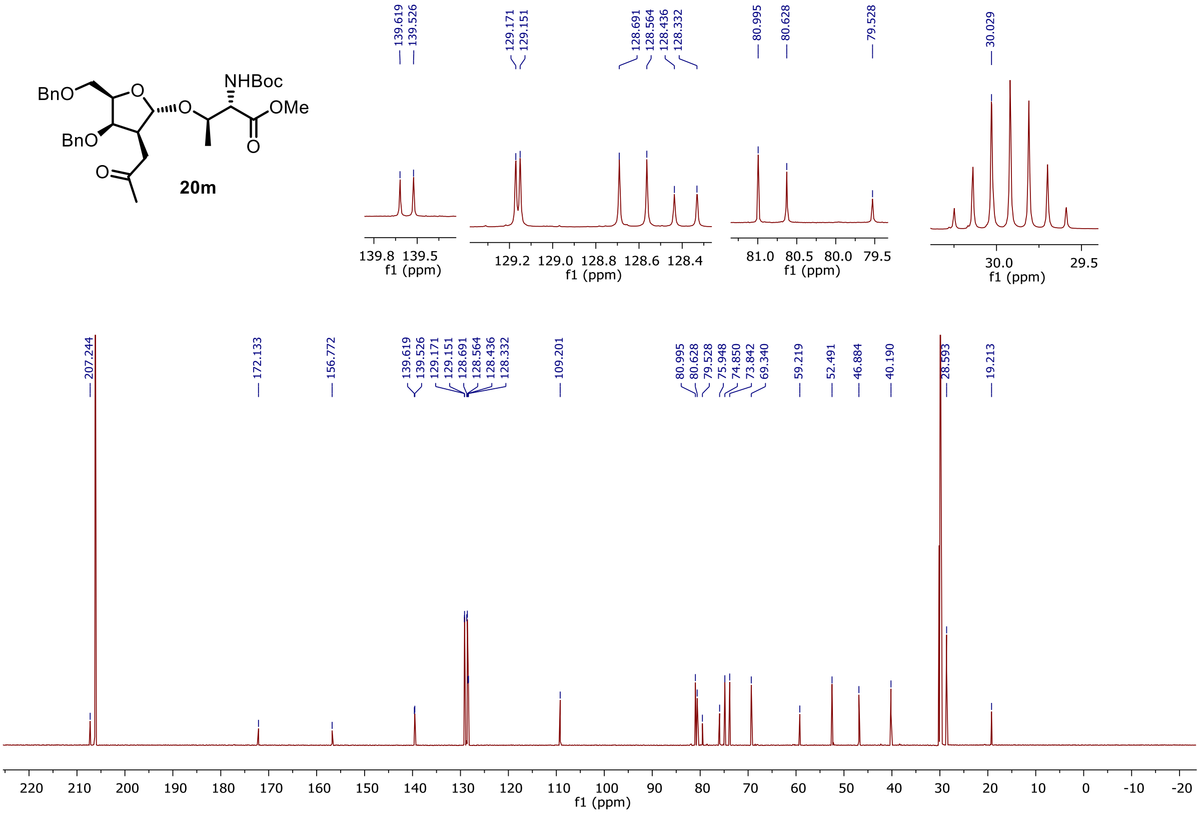

Supplementary Figure 133. ^{13}C spectra for **20m**

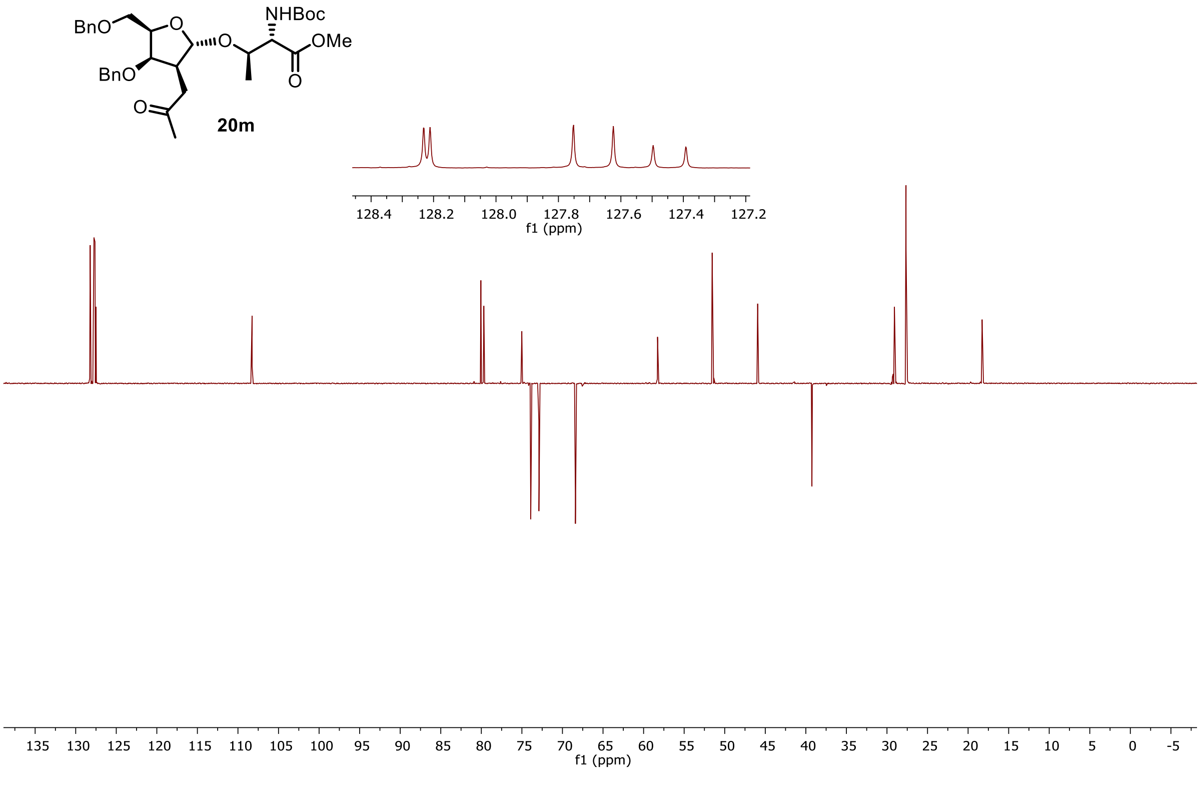

Supplementary Figure 134. DEPT spectra for **20m**

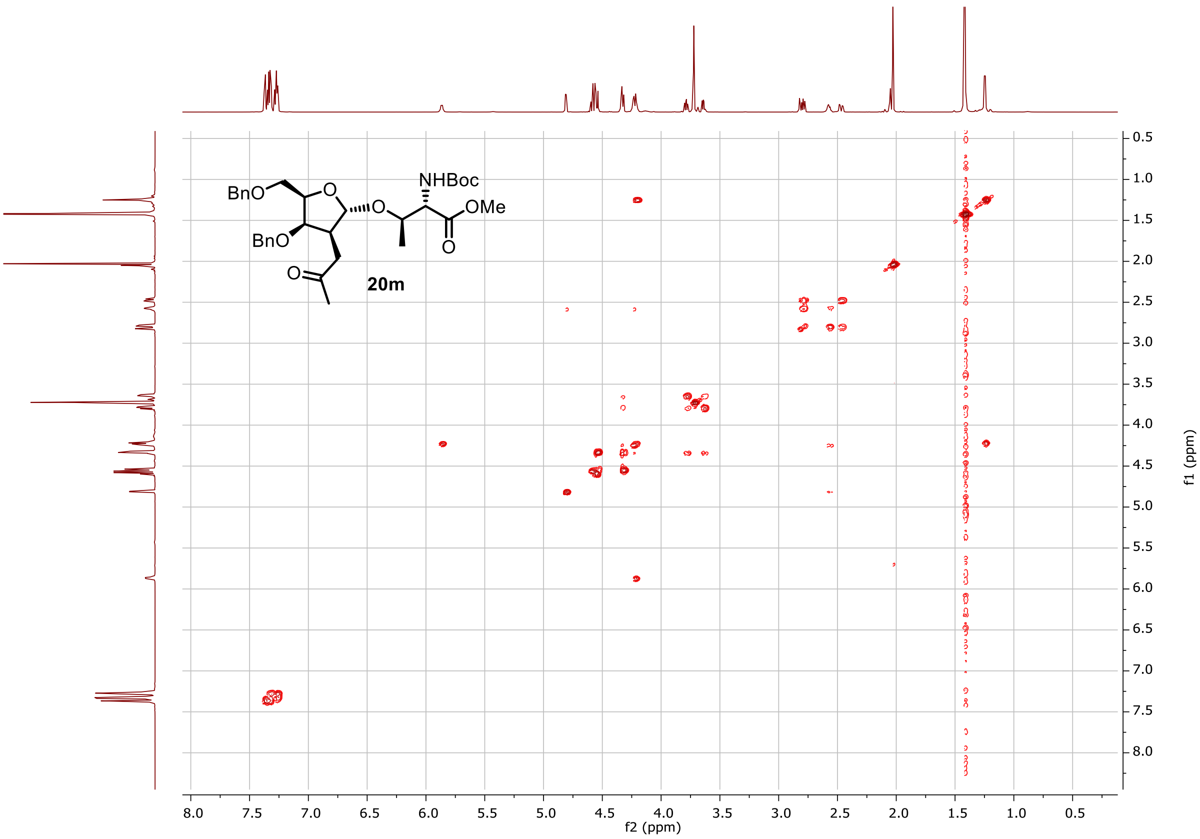

Supplementary Figure 135. COSY spectra for **20m**

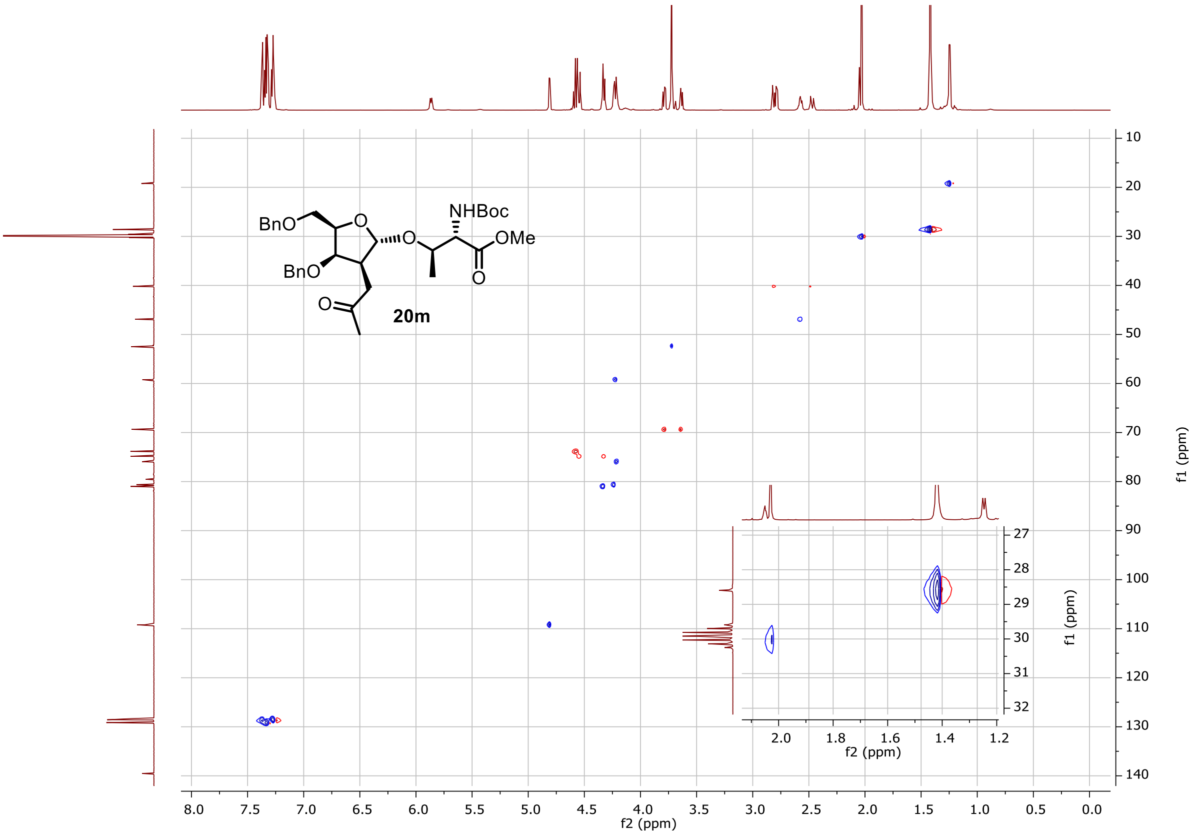

Supplementary Figure 136. HSQC spectra for **20m**

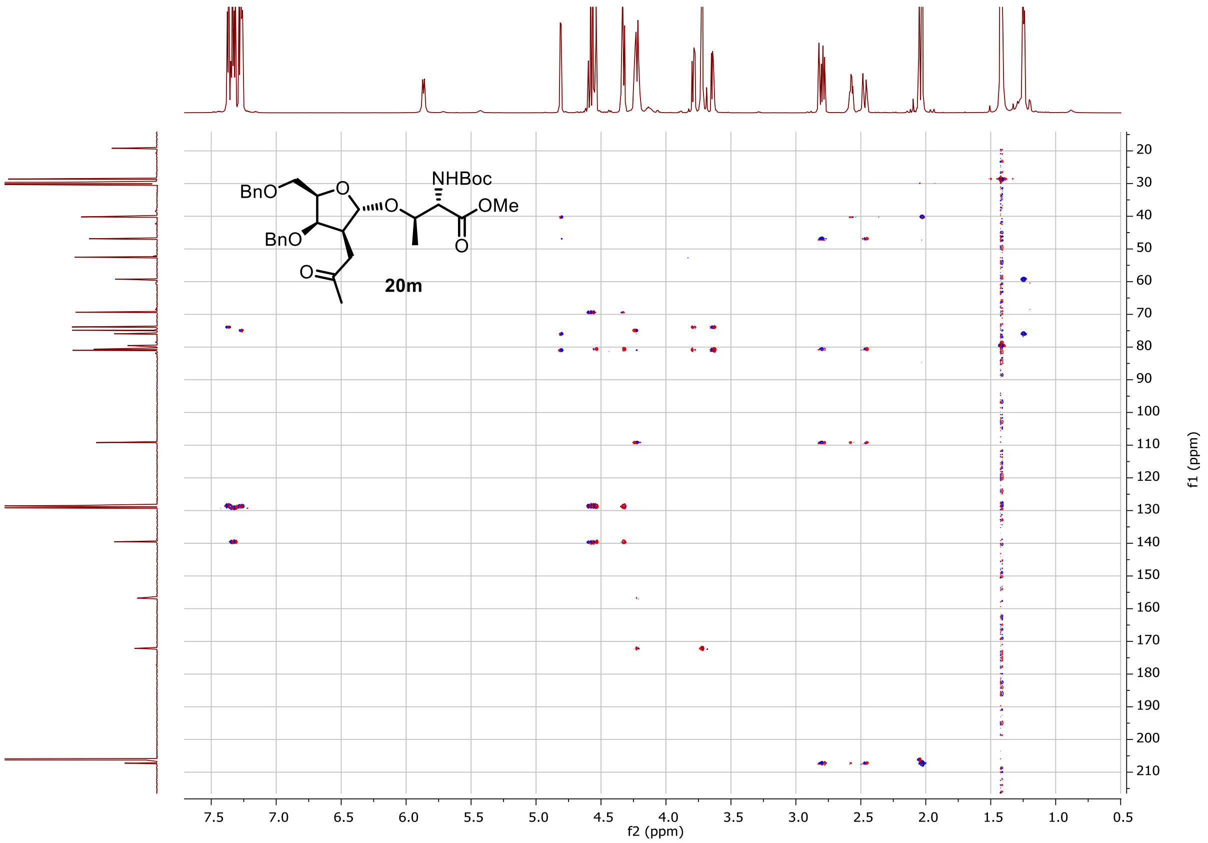

Supplementary Figure 137. HMBC spectra for **20m**

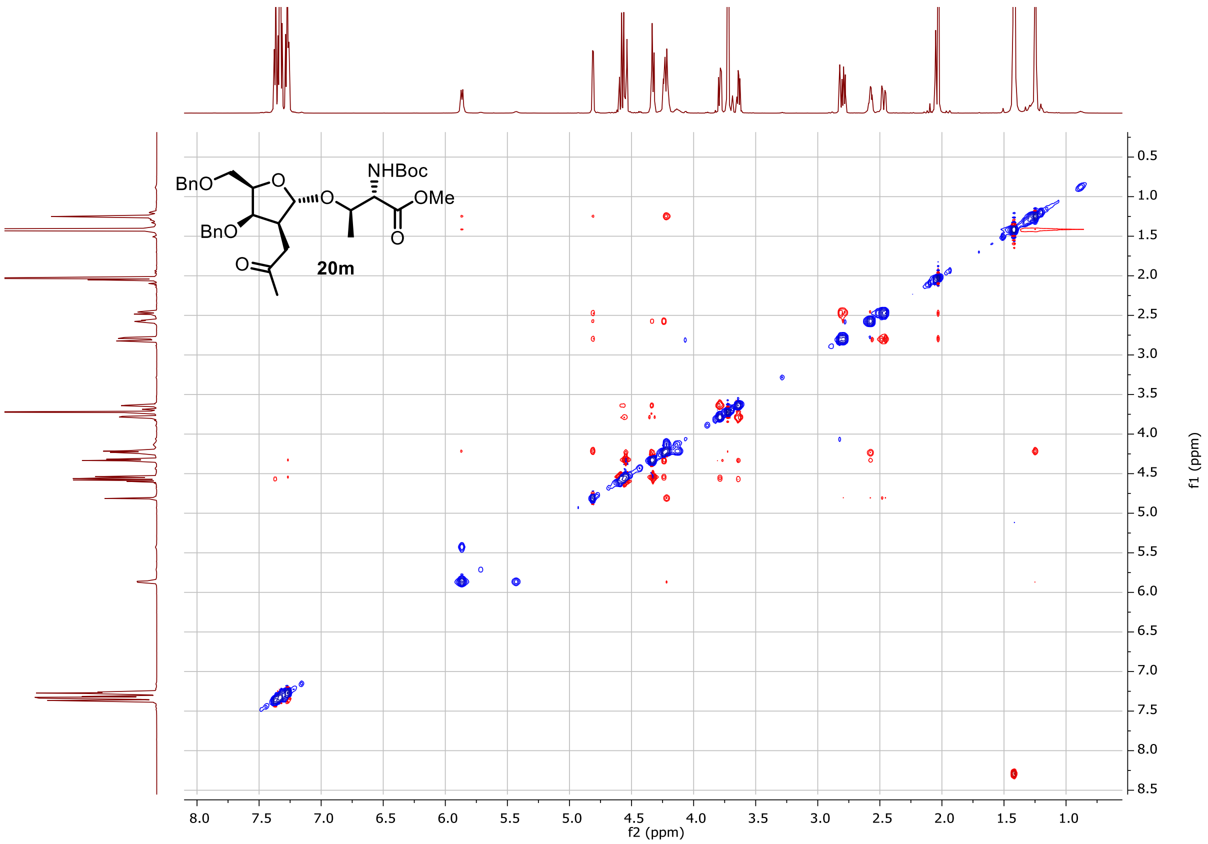

Supplementary Figure 138. NOESY spectra for **20m**

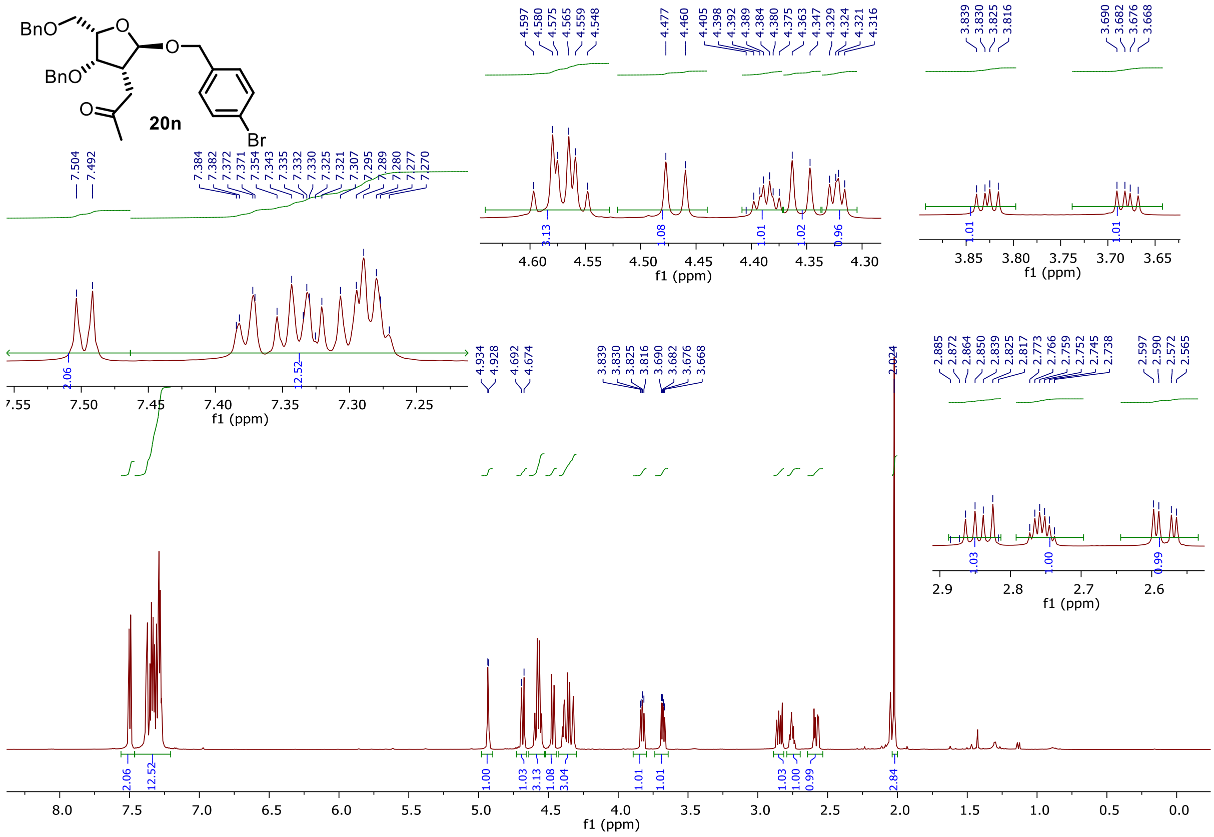

Supplementary Figure 139. ^{1}H spectra for **20n**

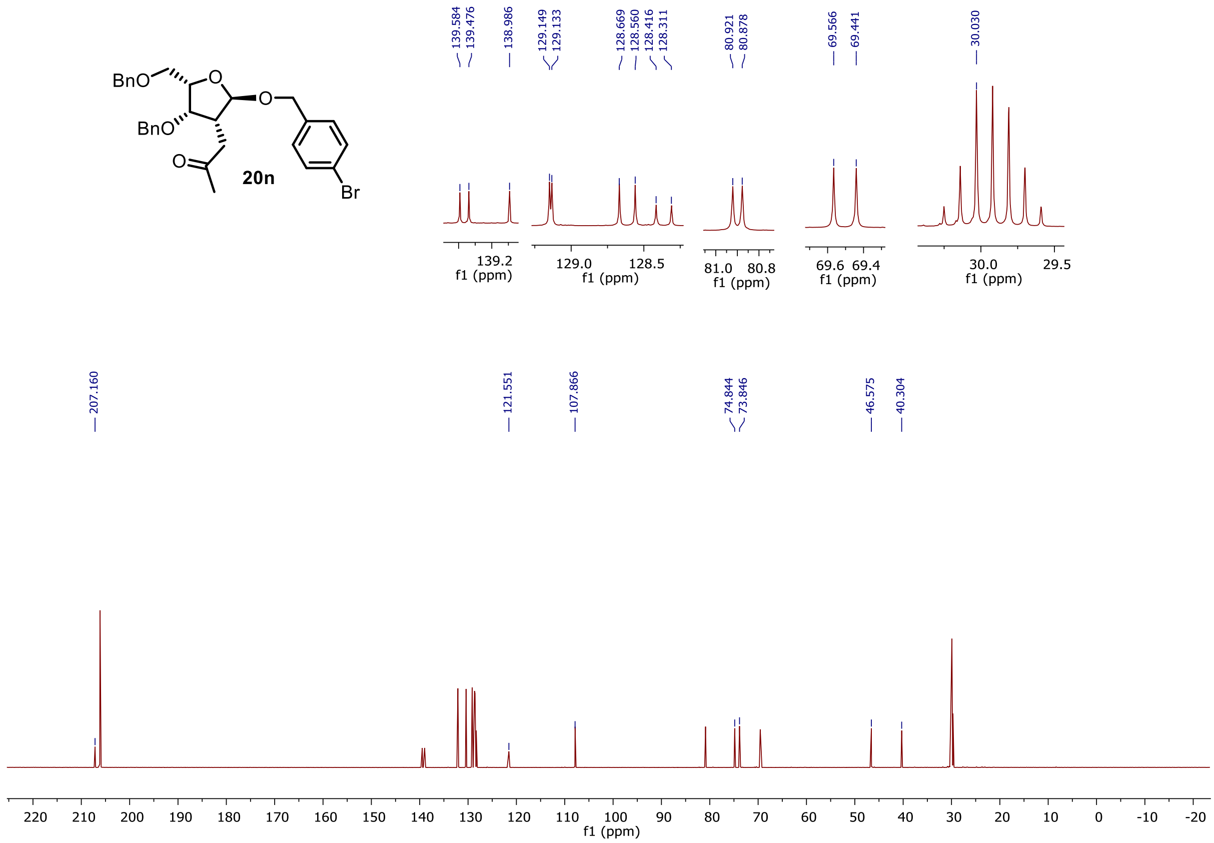

Supplementary Figure 140. ^{13}C spectra for **20n**

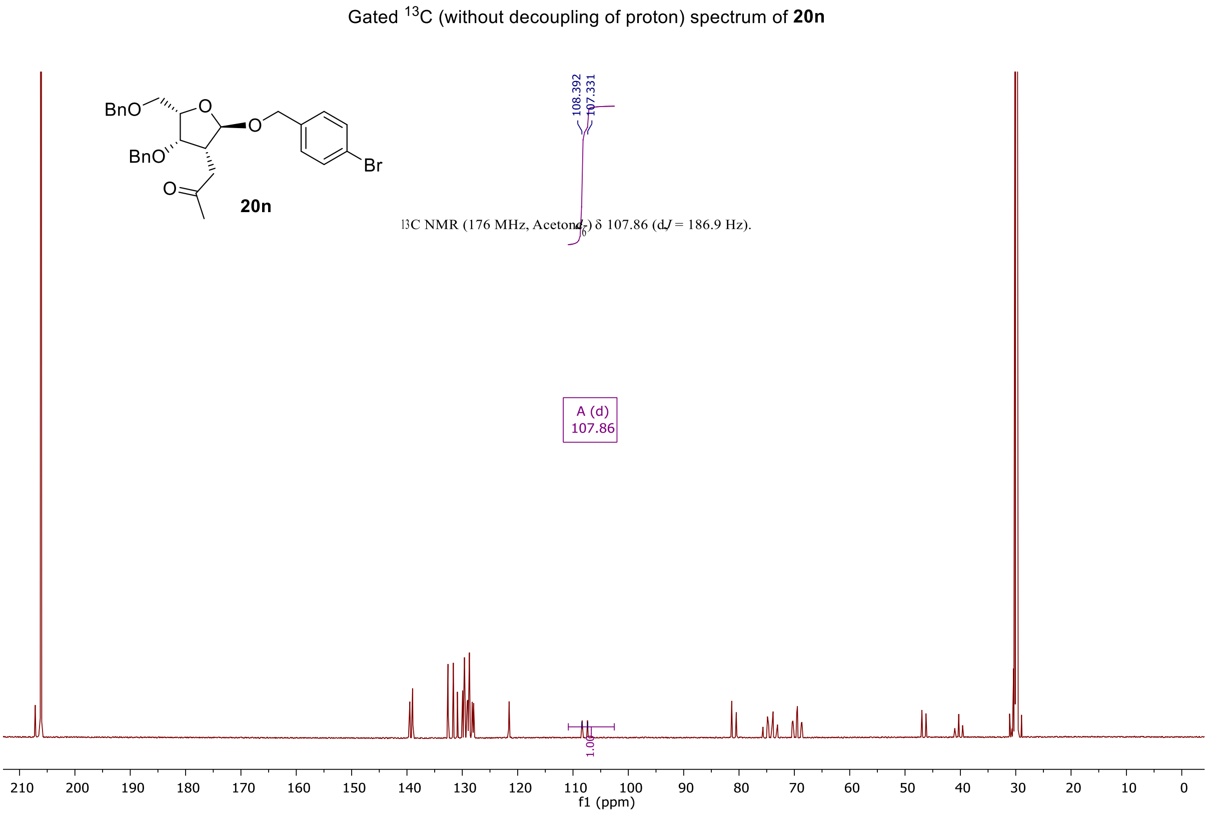

Supplementary Figure 141. Gated ^{13}C (with coupling of proton) spectra for **20n**

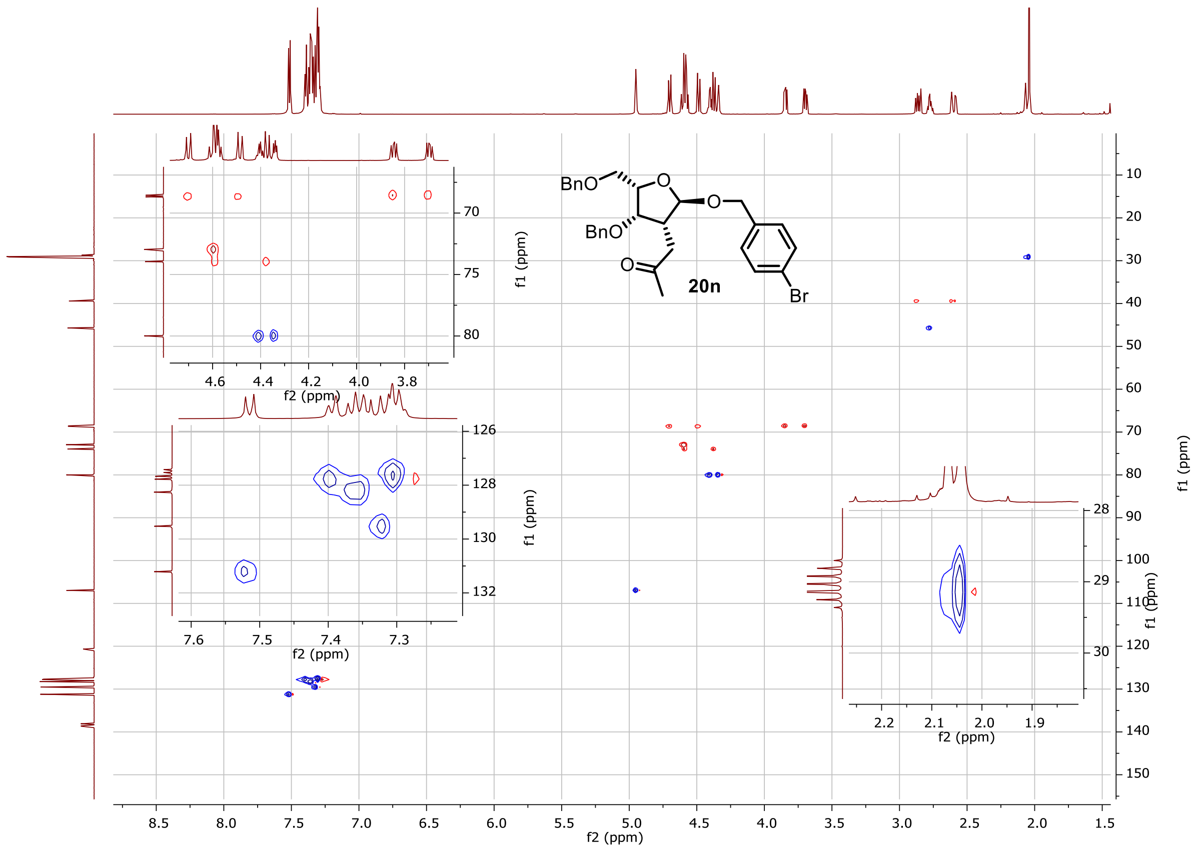

Supplementary Figure 142. HSQC spectra for **20n**

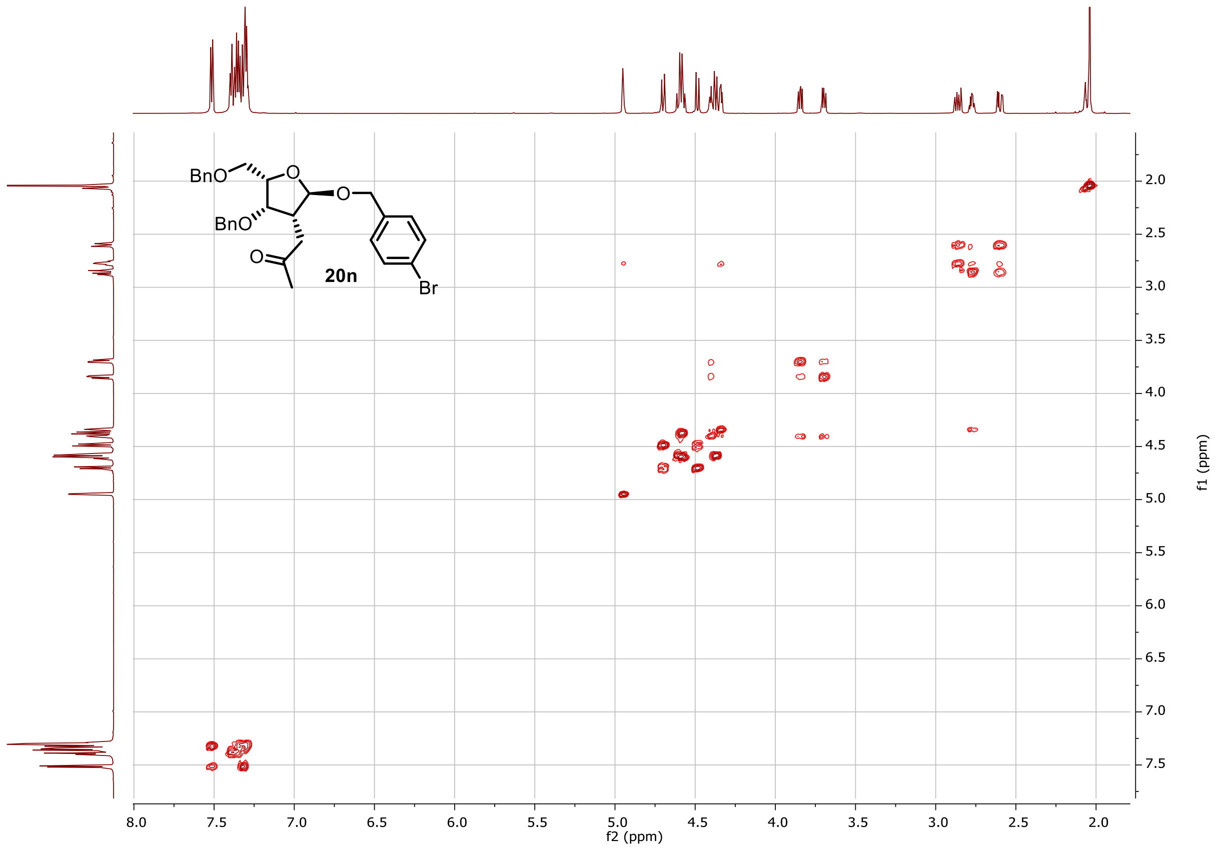

Supplementary Figure 143. COSY spectra for **20n**

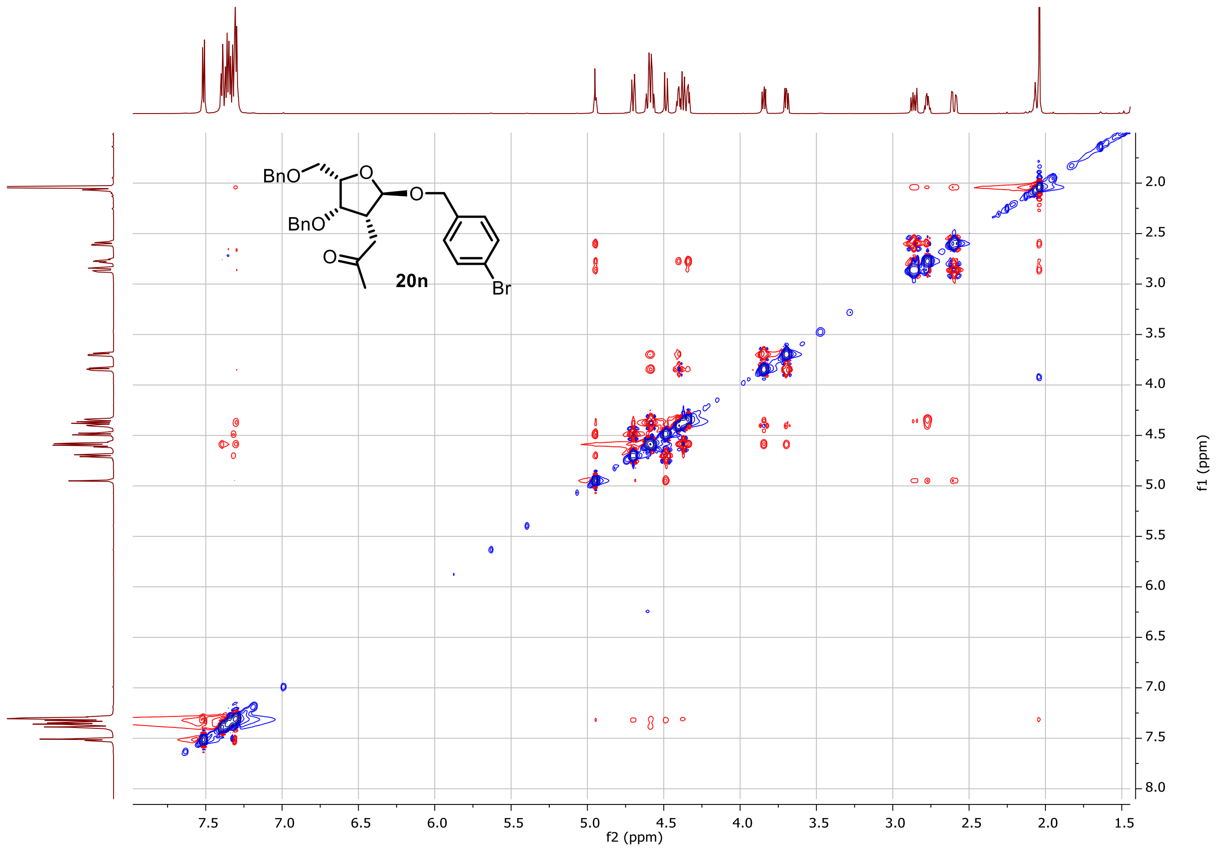

Supplementary Figure 144. NOESY spectra for **20n**

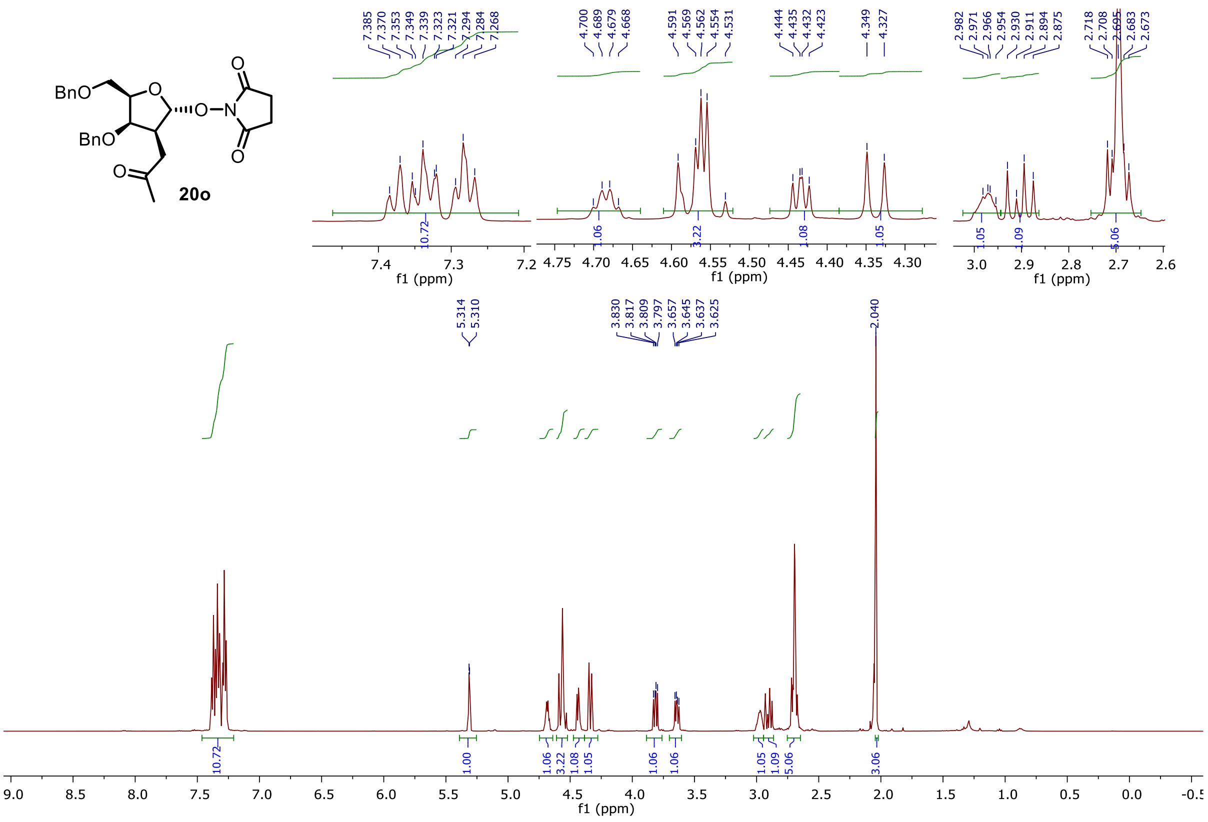

Supplementary Figure 145. ^{1}H spectra for **20o**

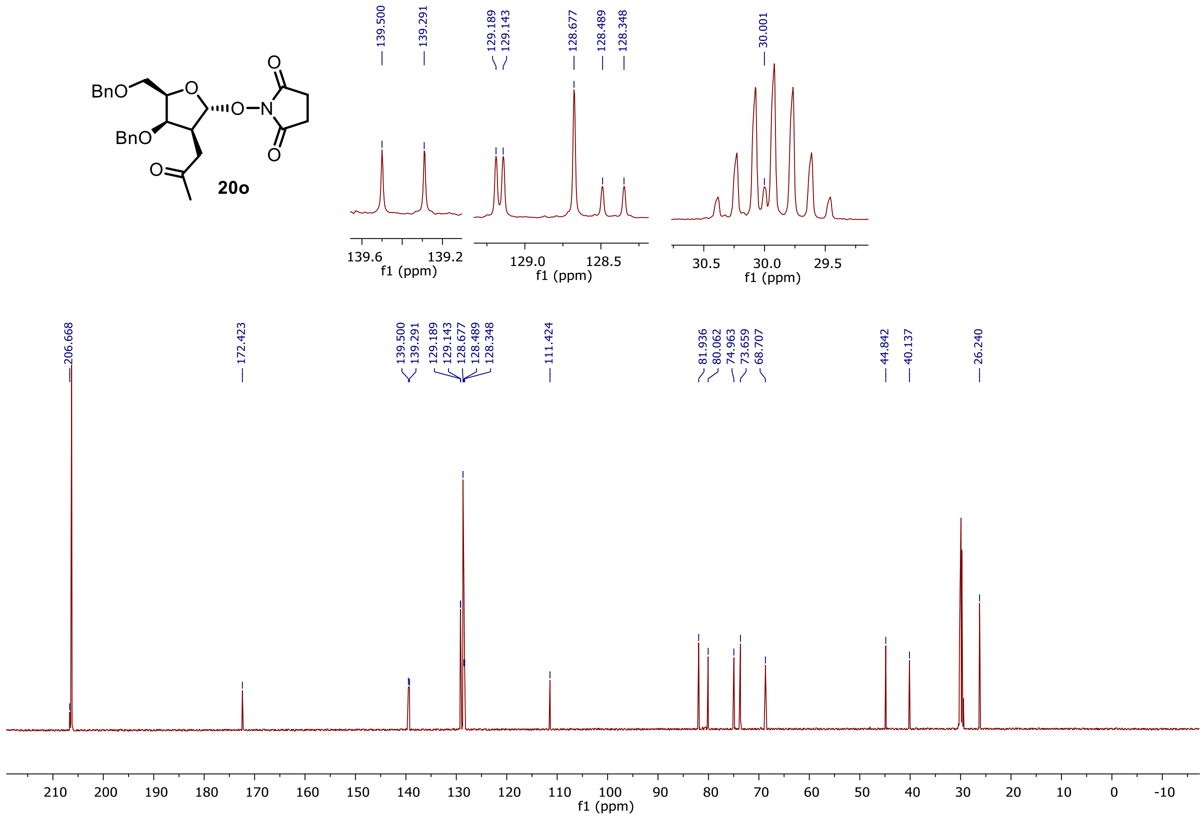

Supplementary Figure 146. ^{13}C spectra for **20o**

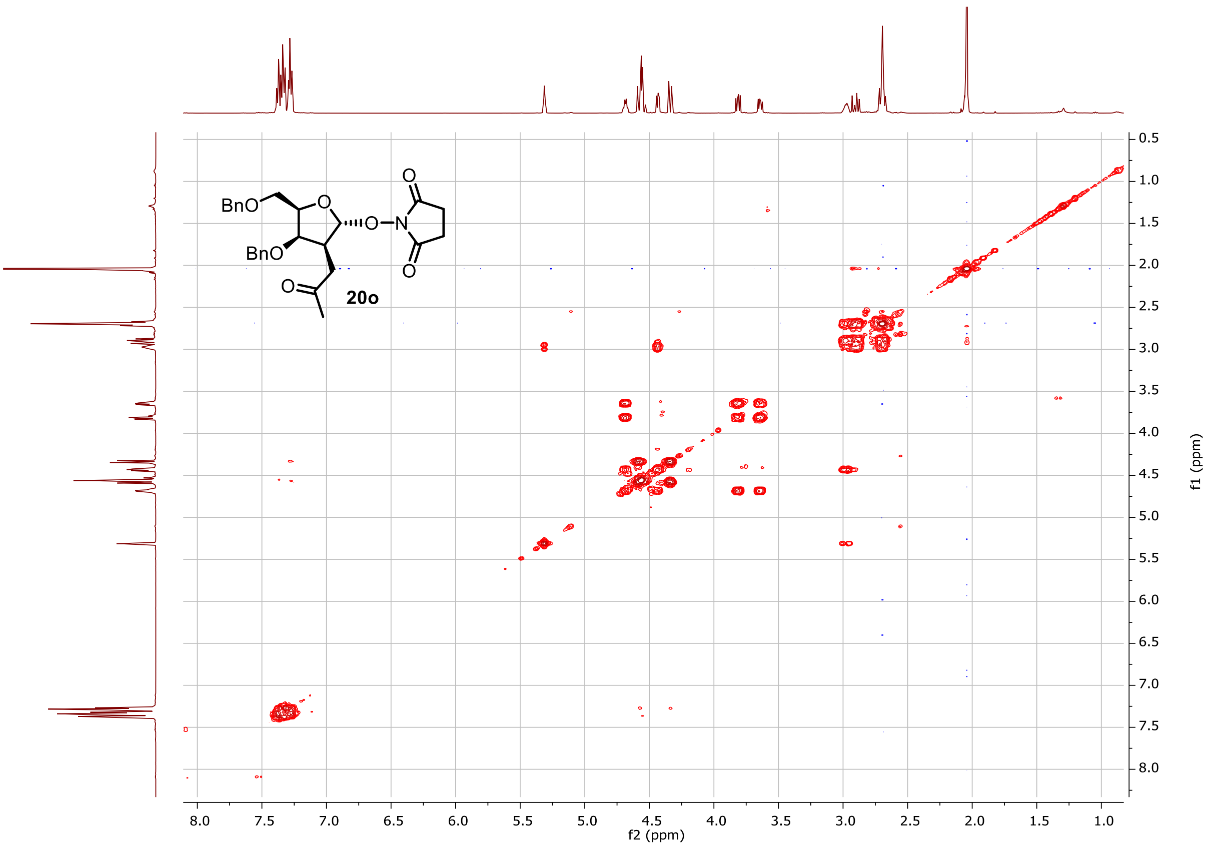

Supplementary Figure 147. COSY spectra for **20o**

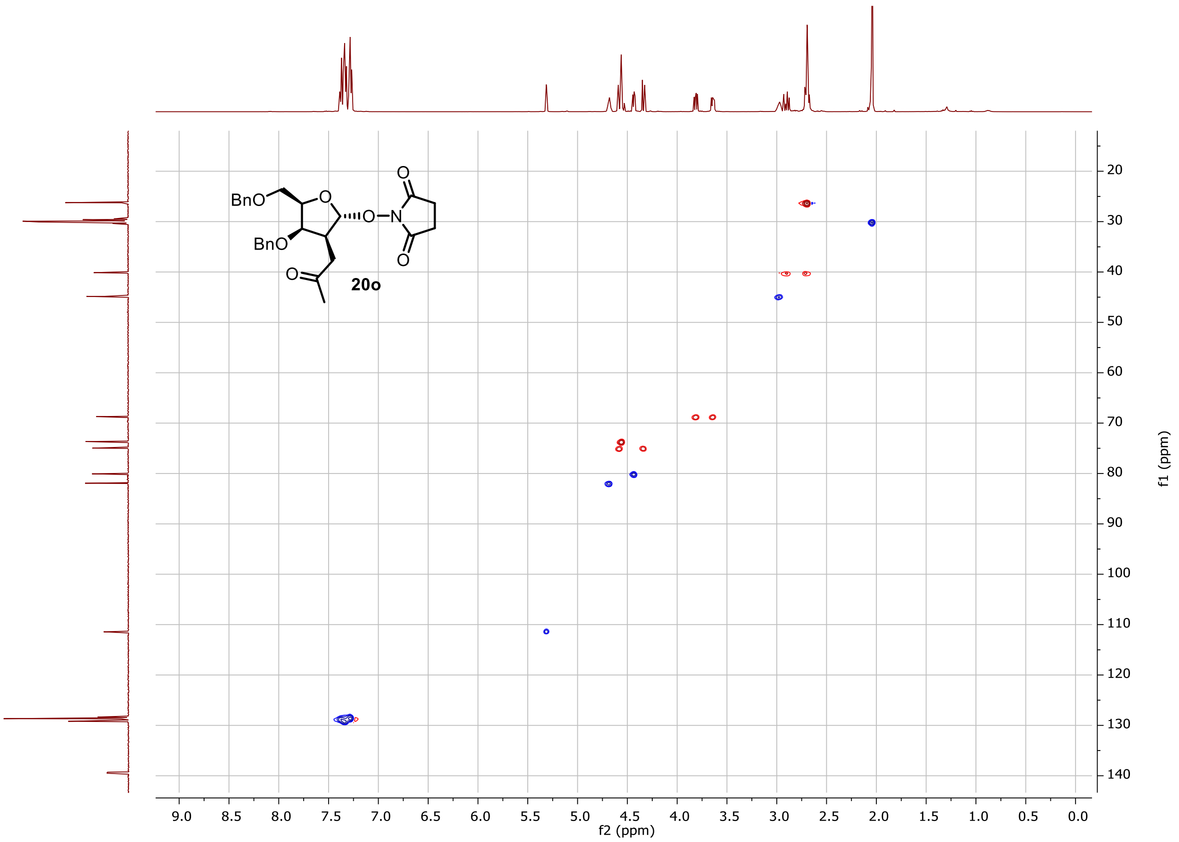

Supplementary Figure 148. HSQC spectra for **20o**

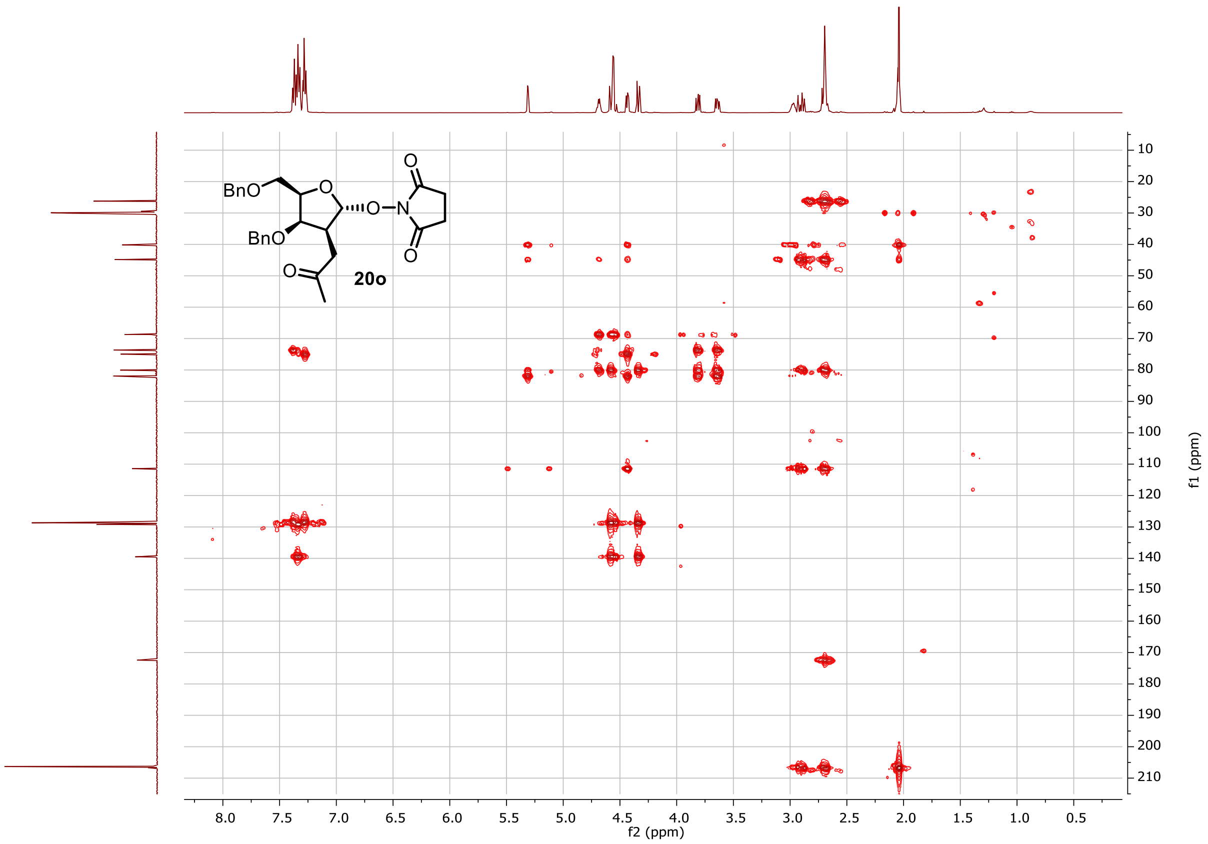

Supplementary Figure 149. HMBC spectra for **20o**

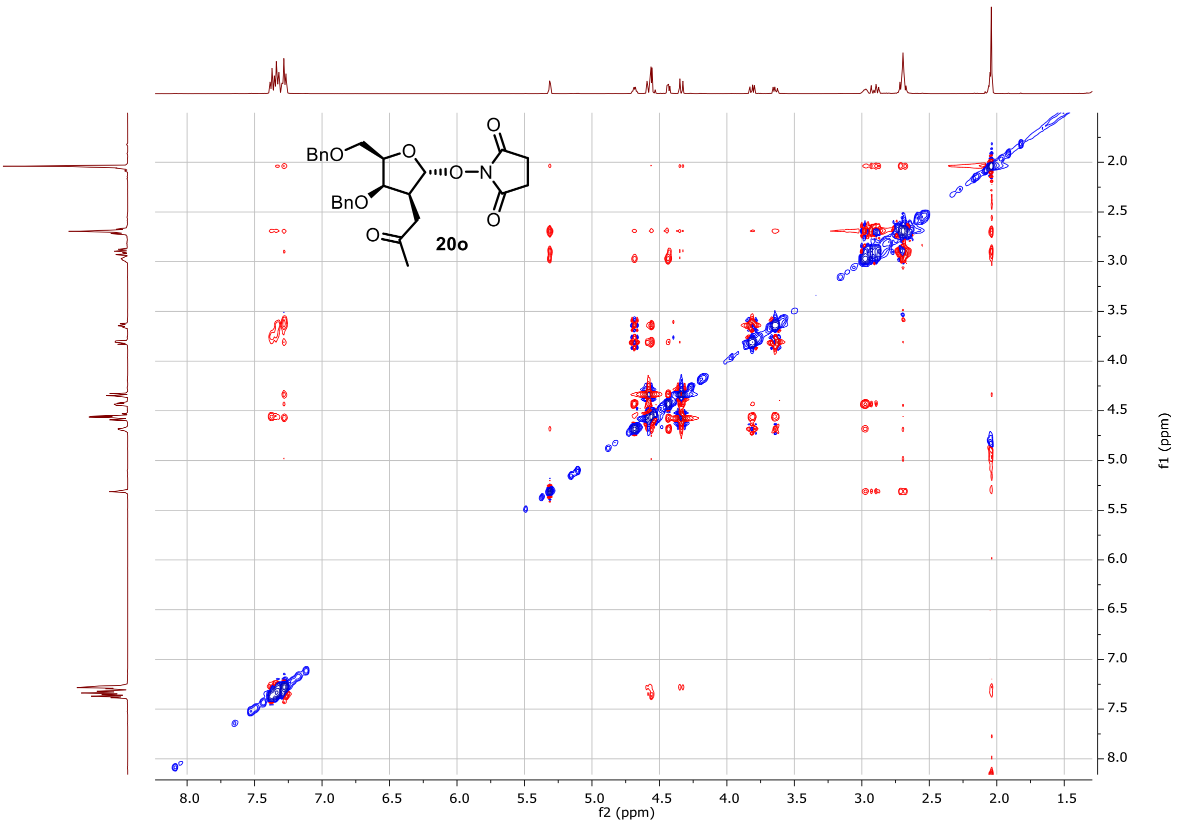

Supplementary Figure 150. NOESY spectra for **20o**

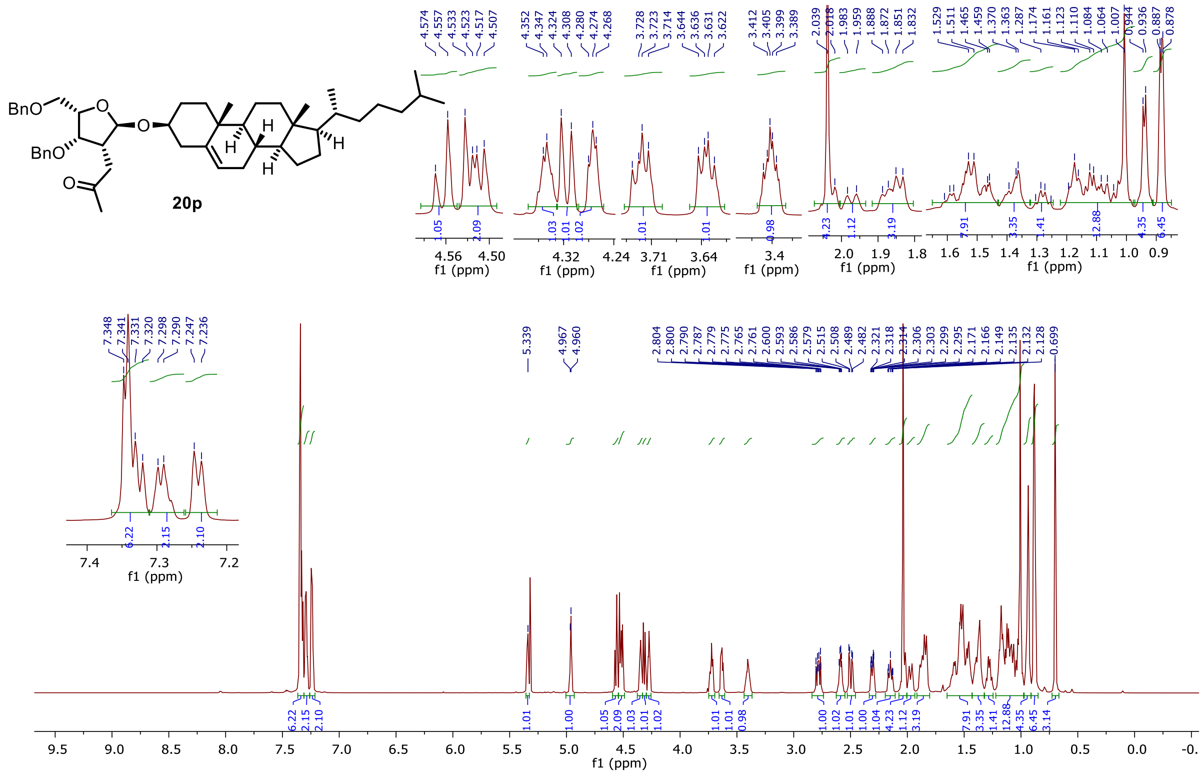

Supplementary Figure 151. ^{1}H spectra for **20p**

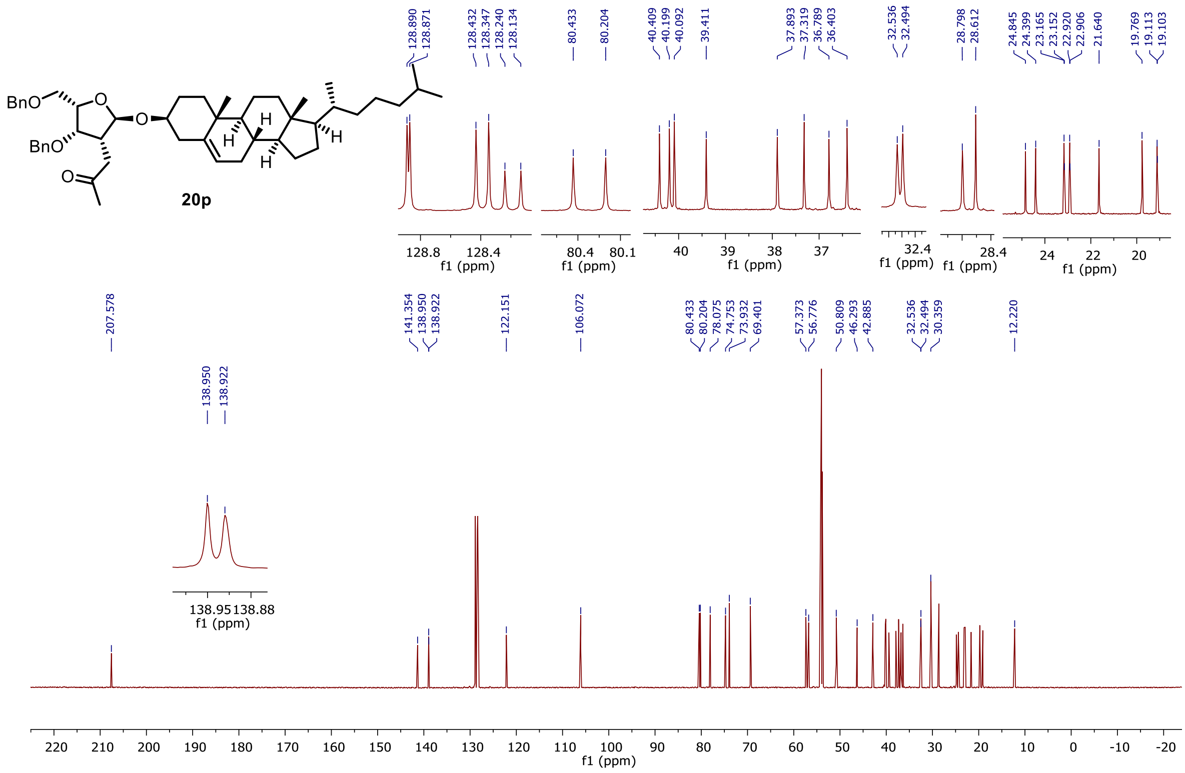

Supplementary Figure 152. ^{13}C spectra for **20p**

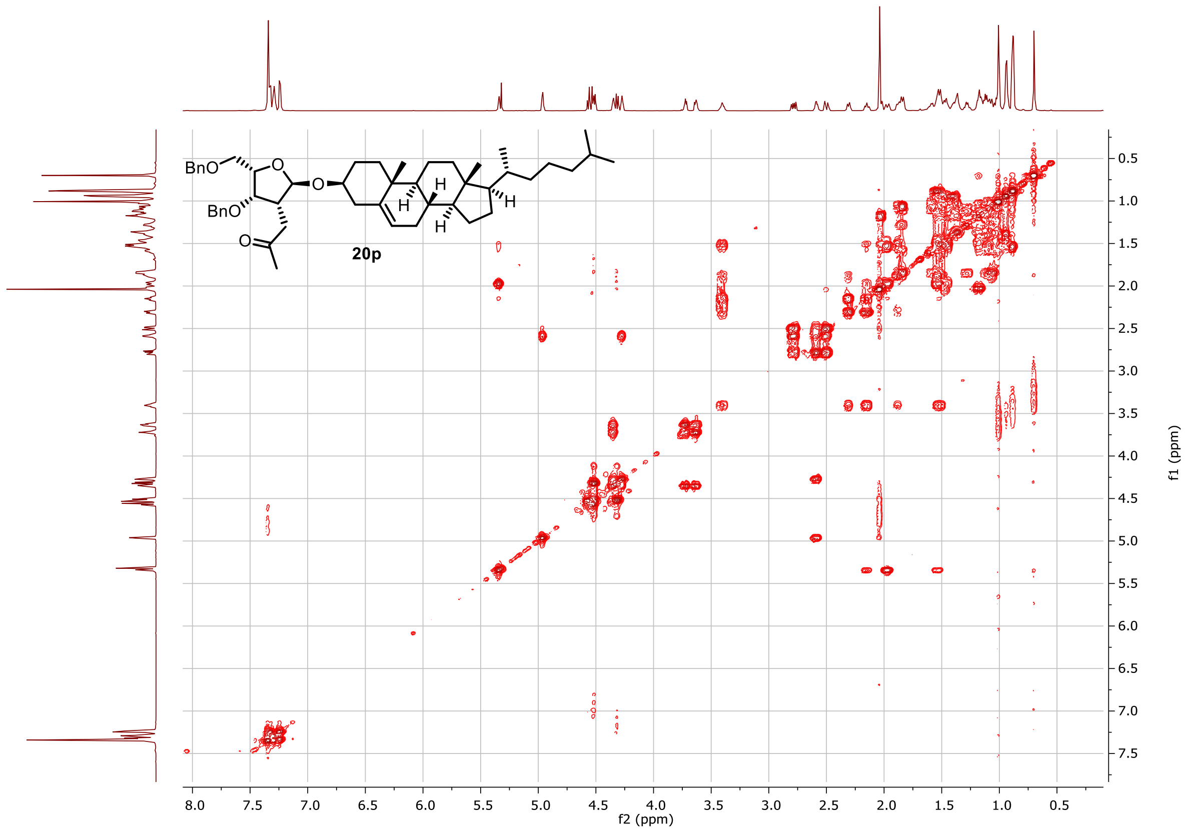

Supplementary Figure 153. COSY spectra for **20p**

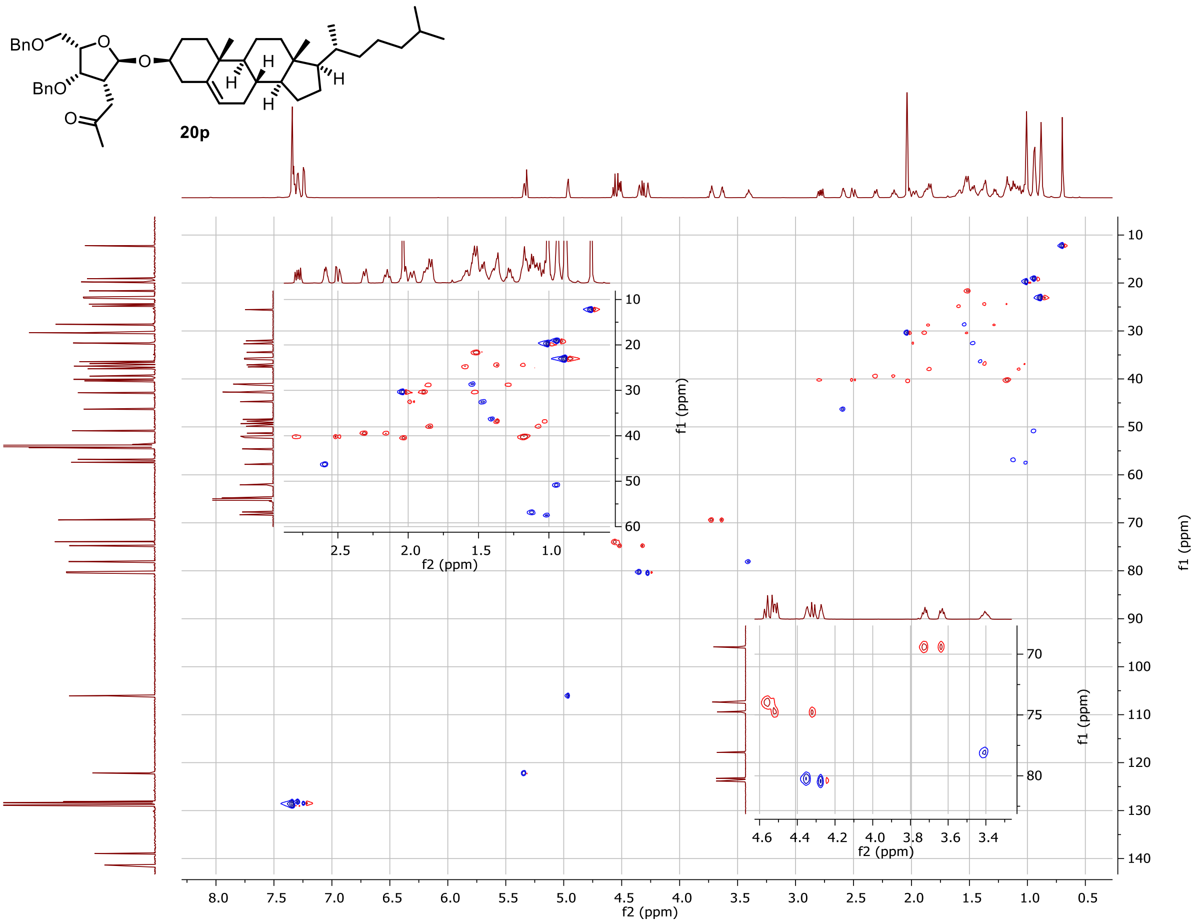

Supplementary Figure 154. HSQC spectra for **20p**

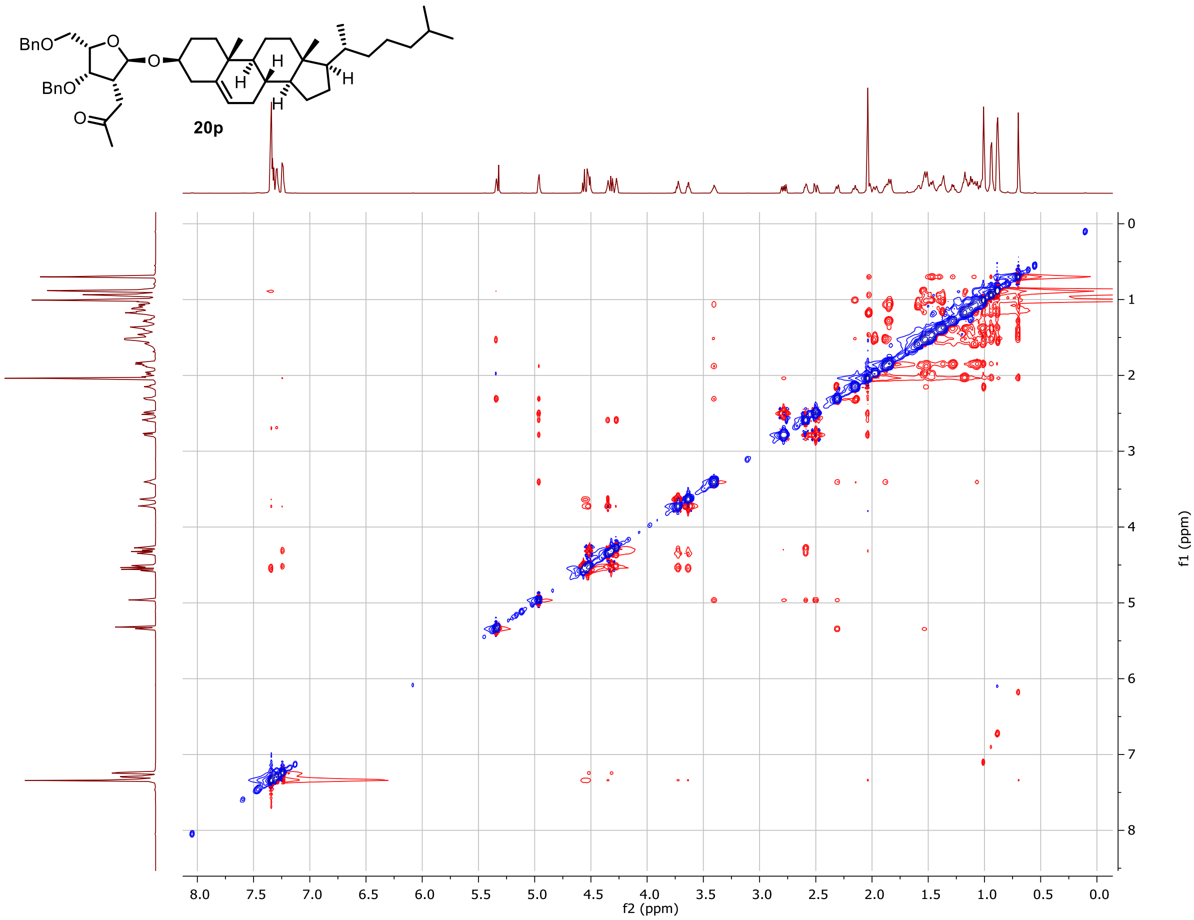

Supplementary Figure 155. NOESY spectra for **20p**

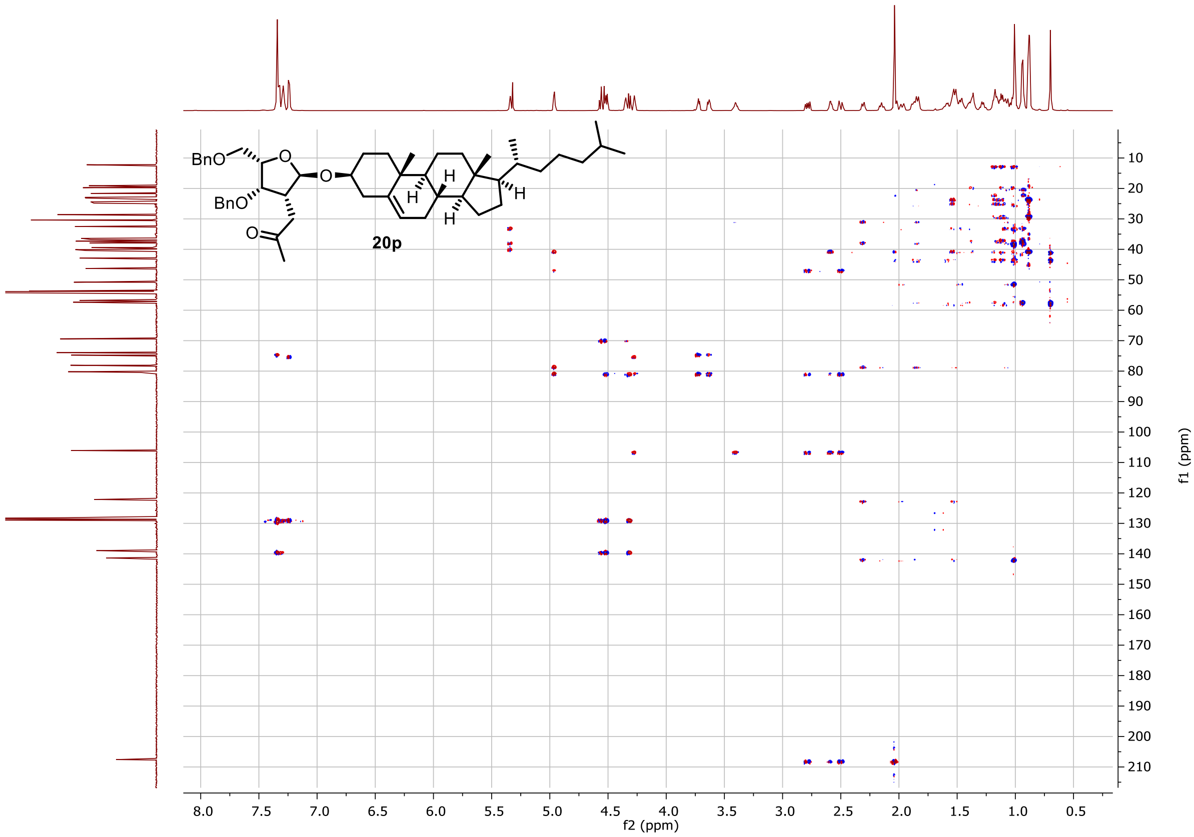

Supplementary Figure 156. HMBC spectra for **20p**

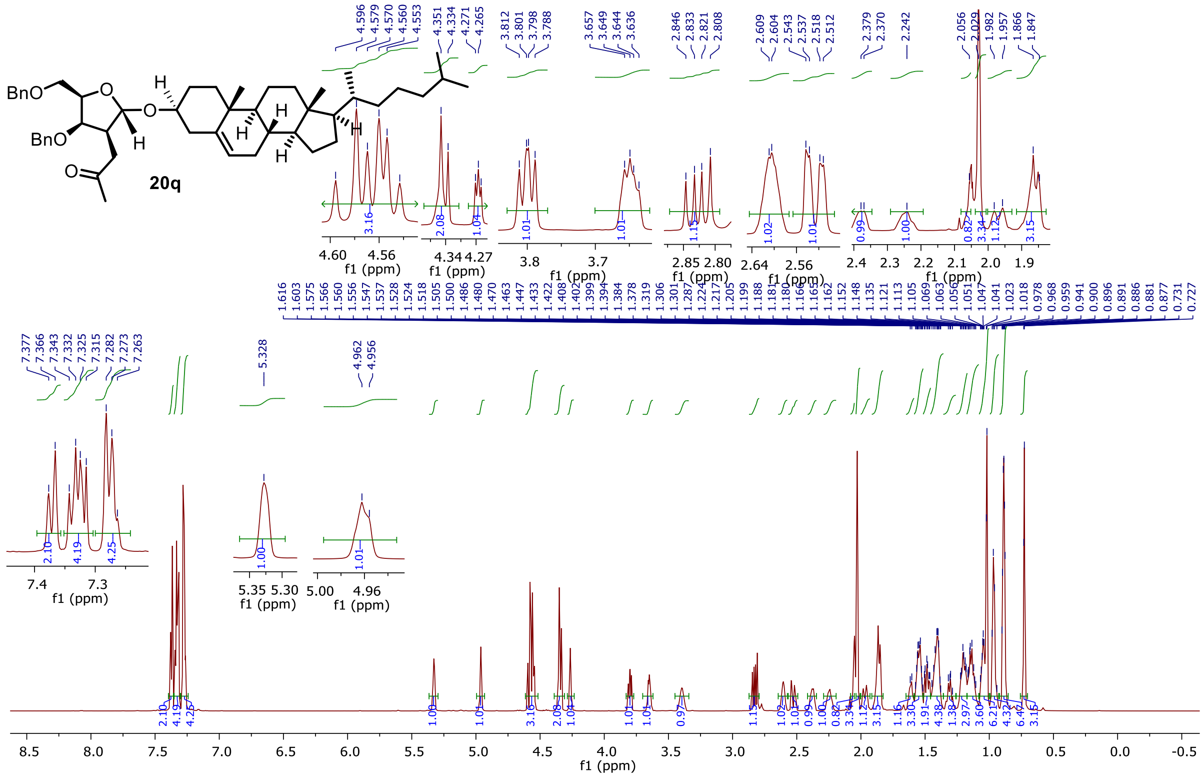

Supplementary Figure 157. ^{1}H spectra for **20q**

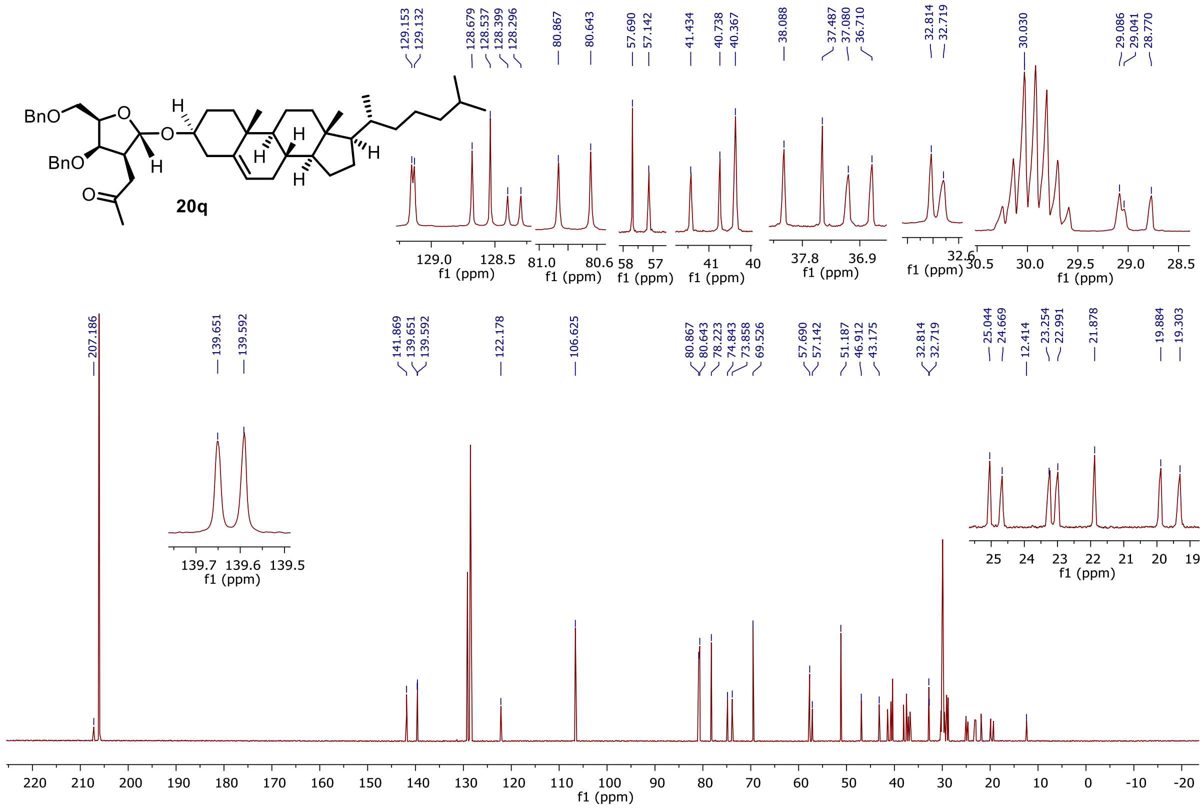

Supplementary Figure 158. ^{13}C spectra for **20q**

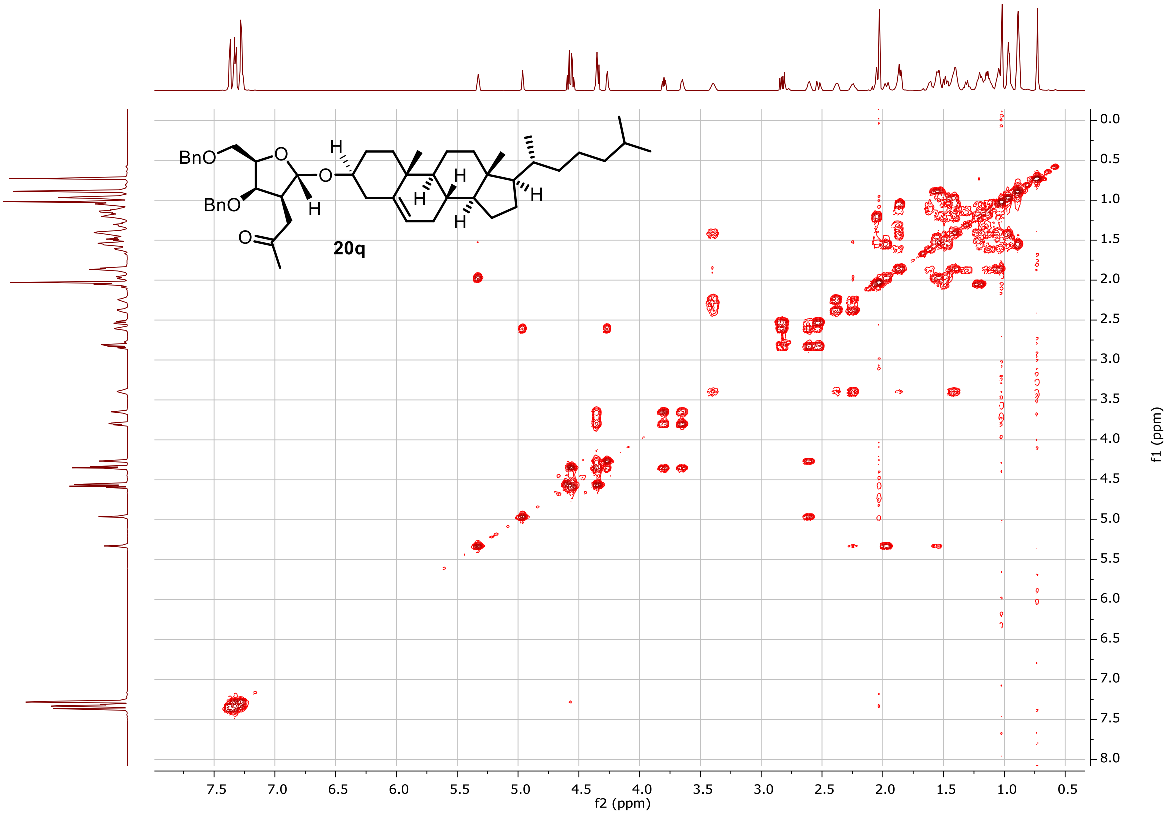

Supplementary Figure 159. COSY spectra for **20q**

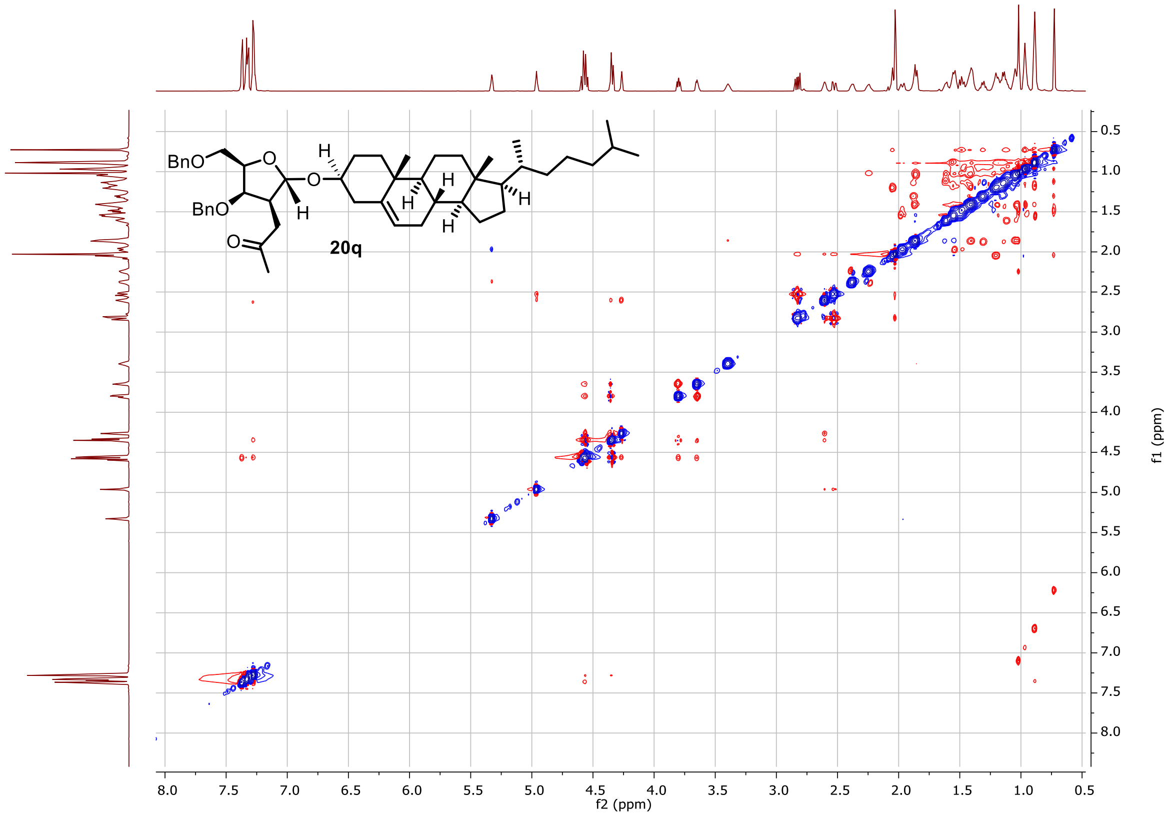

Supplementary Figure 160. NOESY spectra for **20q**

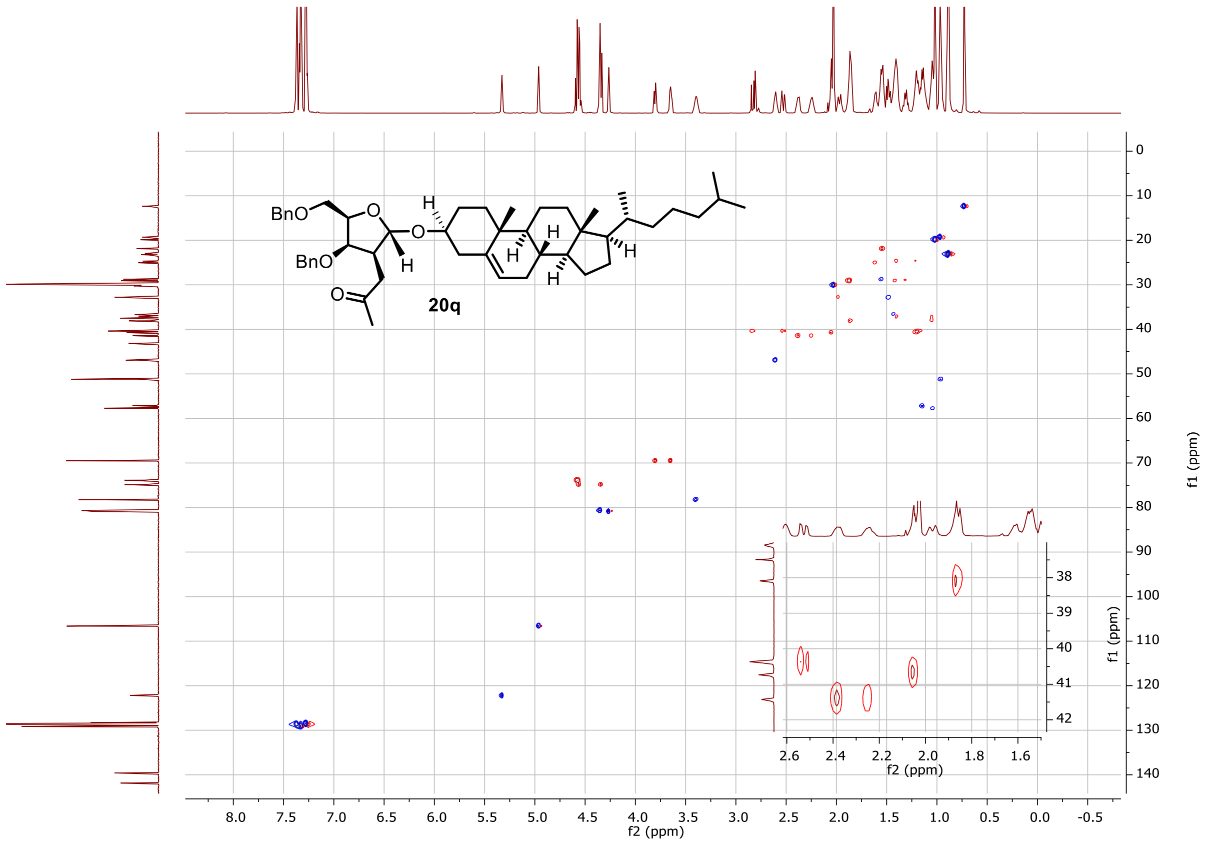

Supplementary Figure 161. HSQC spectra for **20q**

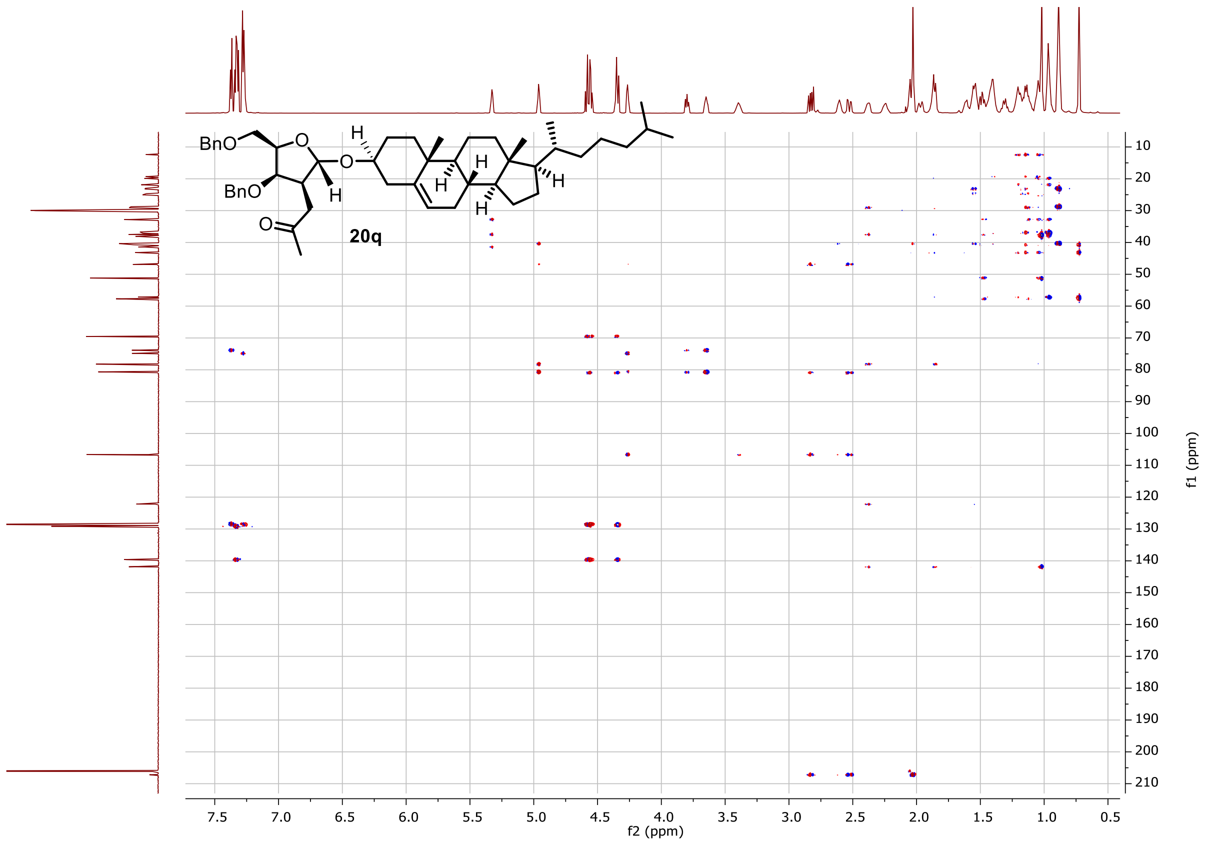

Supplementary Figure 162. HMBC spectra for **20q**

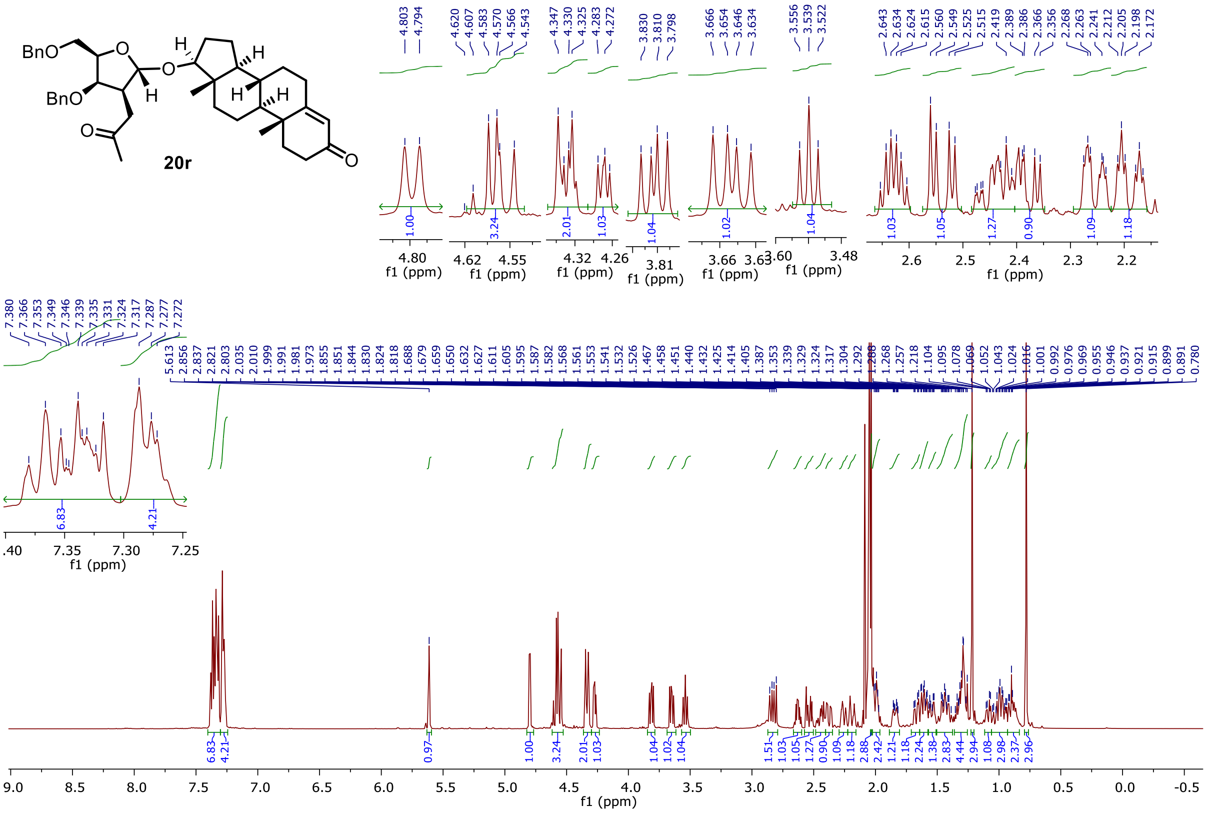

Supplementary Figure 163. ^{1}H spectra for **20r**

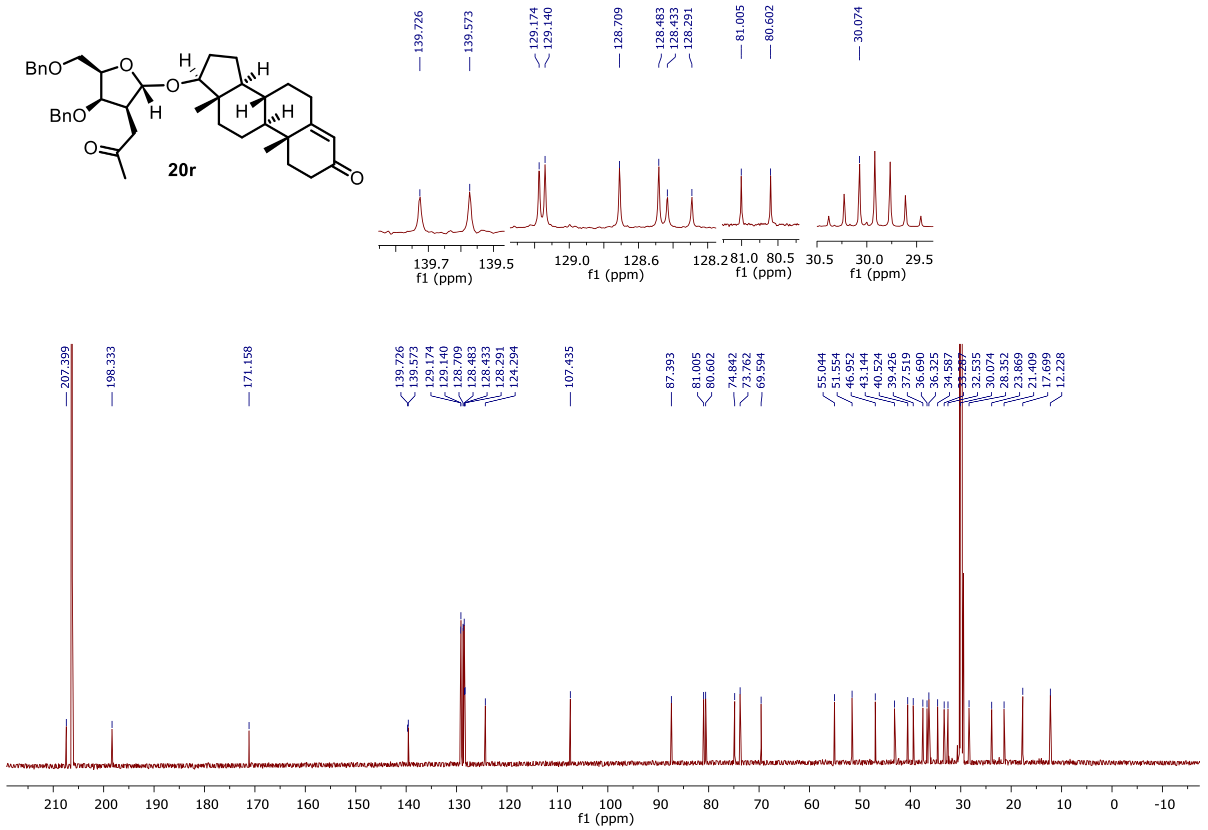

Supplementary Figure 164. ^{13}C spectra for **20r**

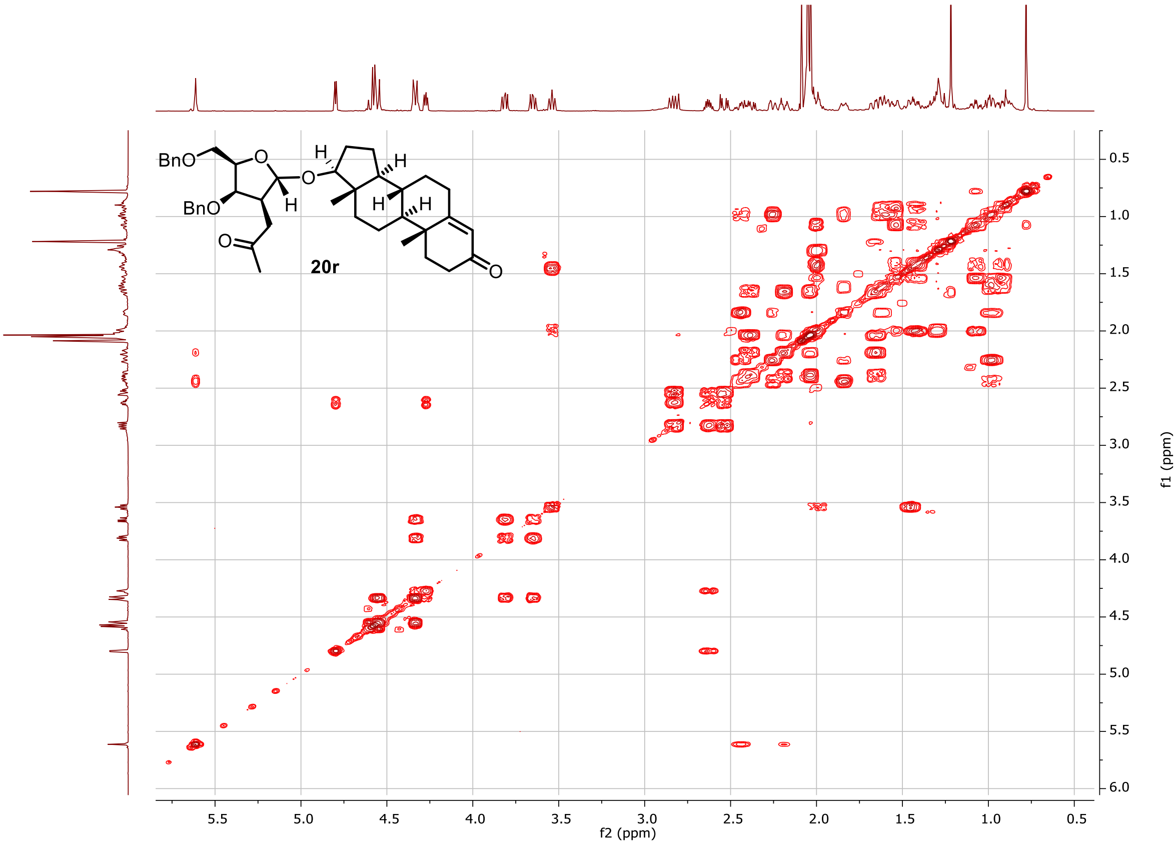

Supplementary Figure 165. COSY spectra for **20r**

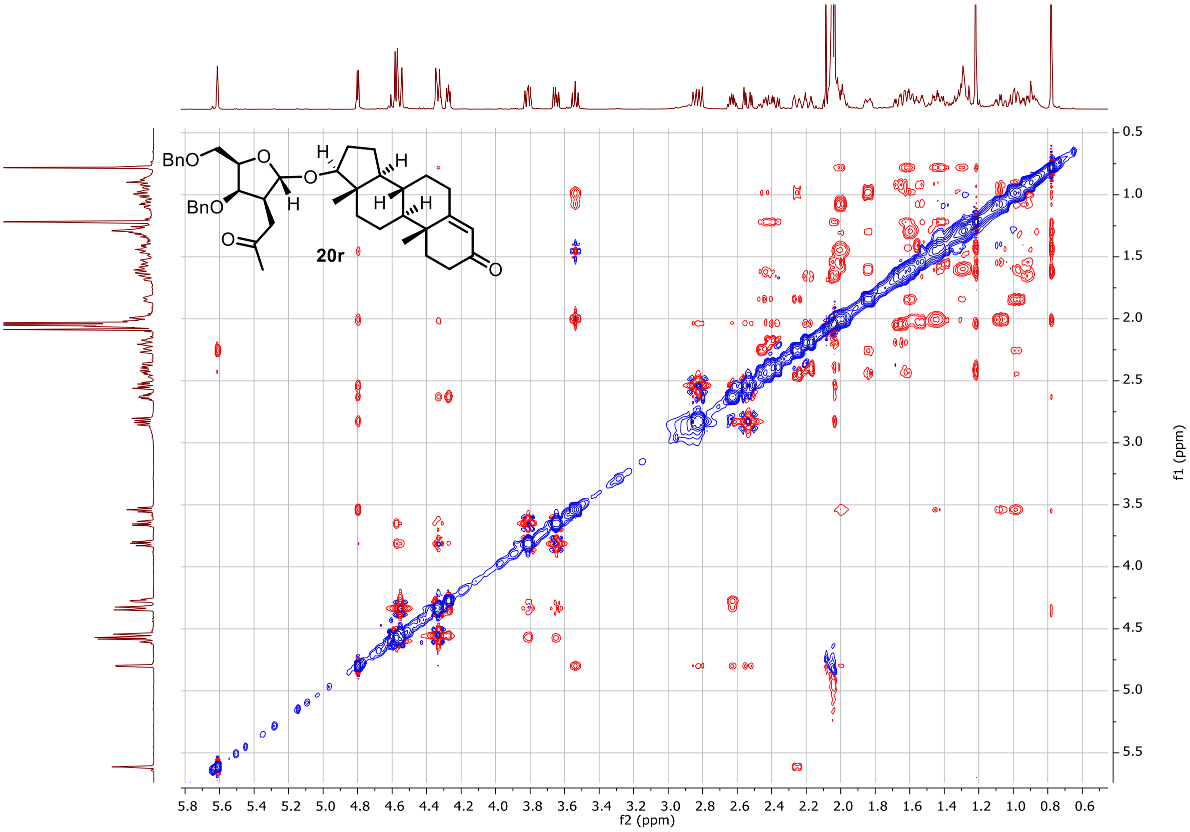

Supplementary Figure 166. NOESY spectra for **20r**

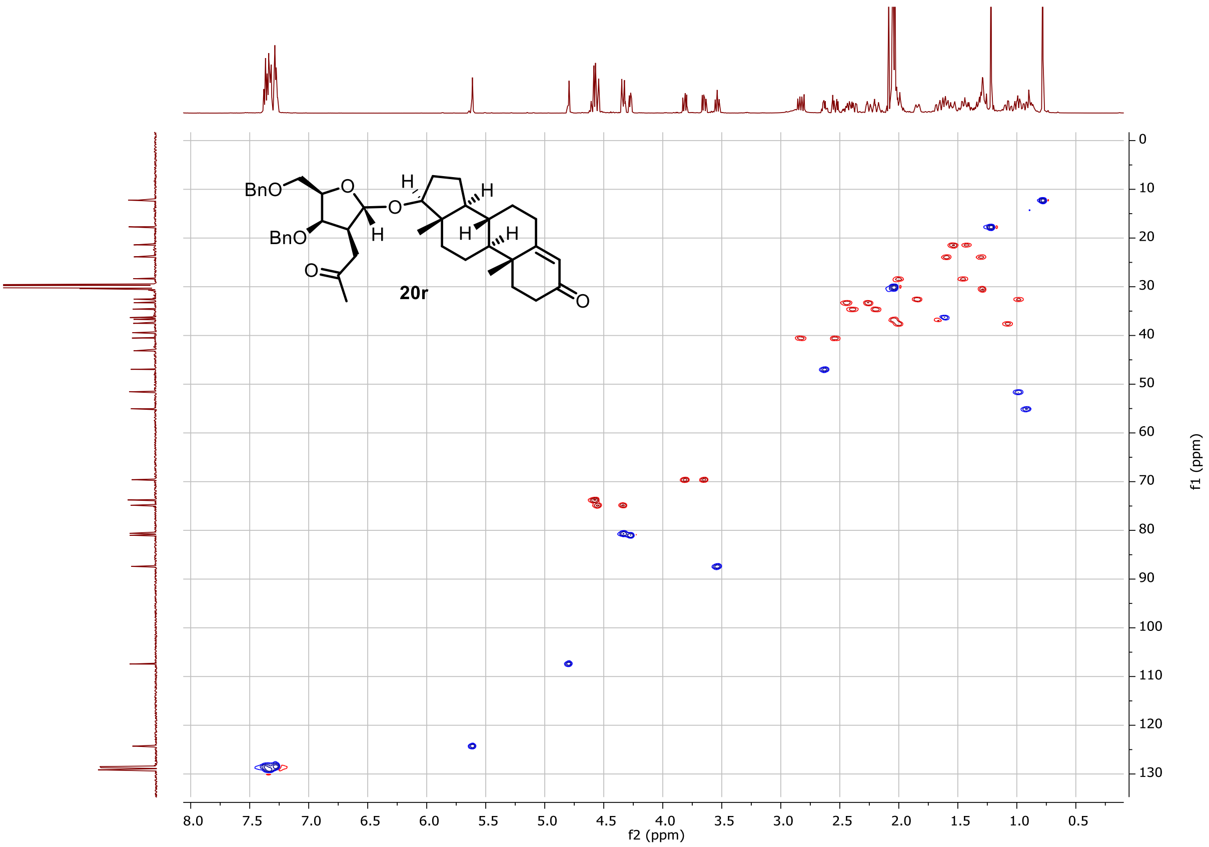

Supplementary Figure 167. HSQC spectra for **20r**

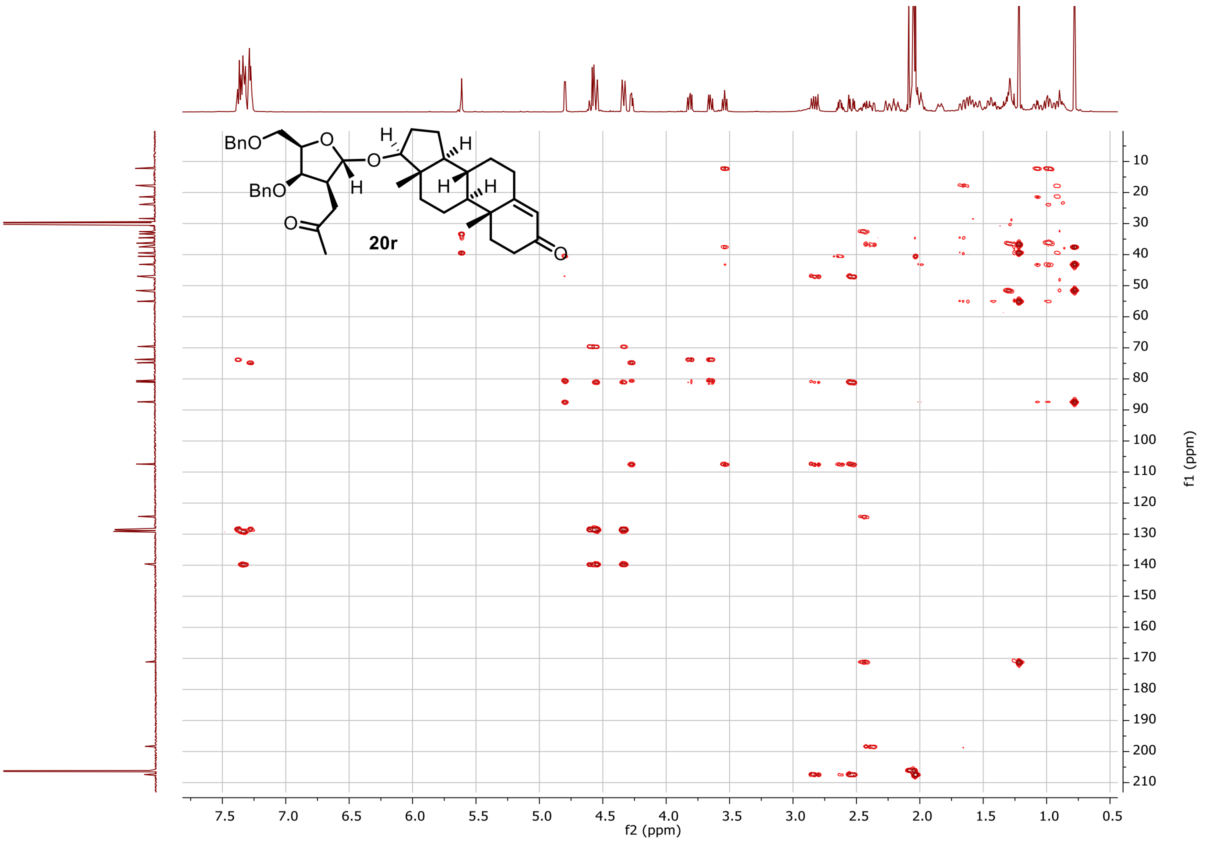

Supplementary Figure 168. HMBC spectra for **20r**

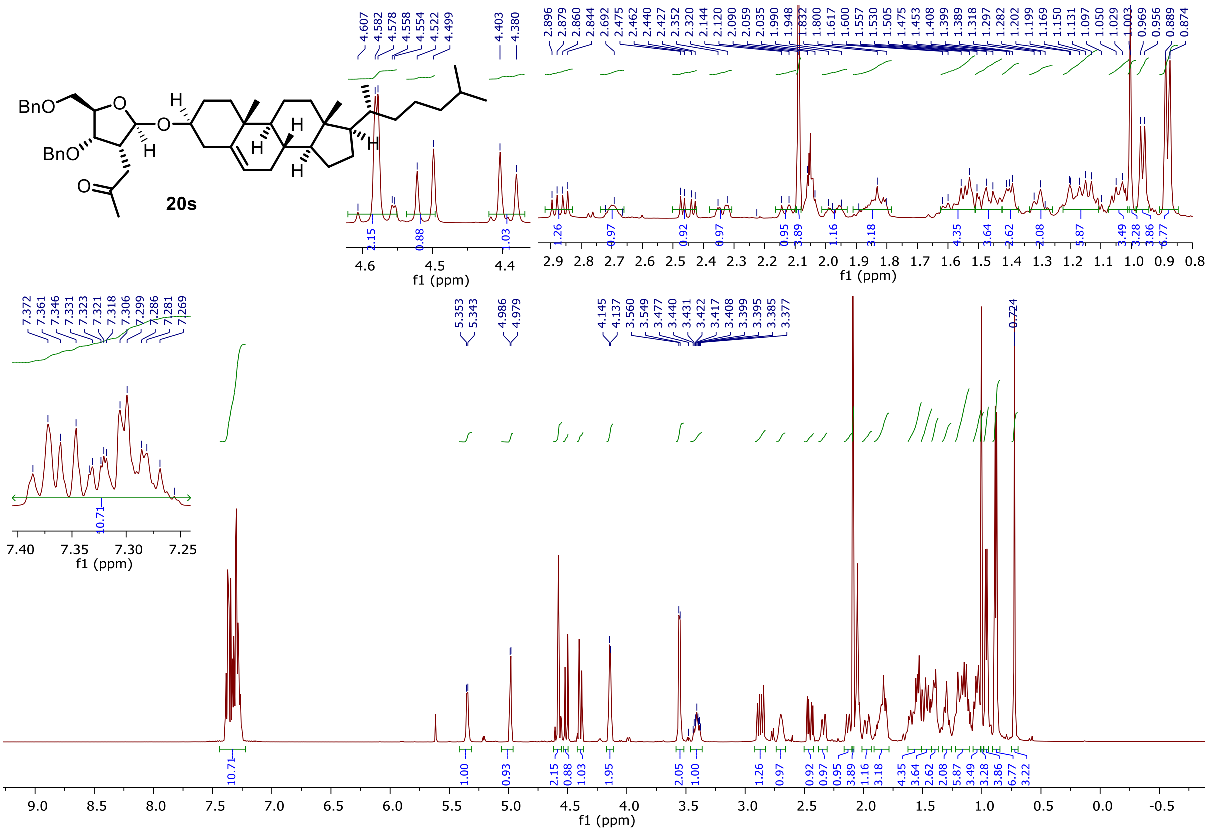

Supplementary Figure 169. ^{1}H spectra for **20s**

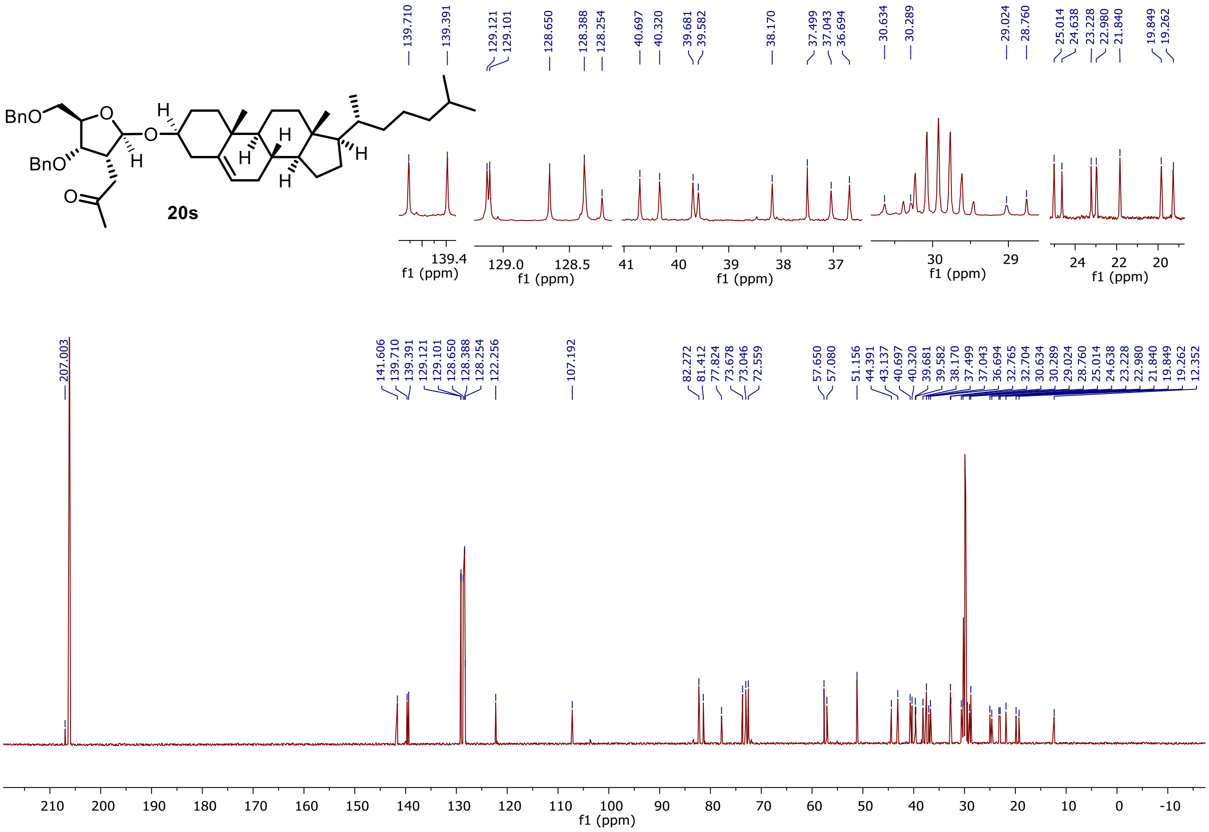

Supplementary Figure 170. ^{13}C spectra for **20s**

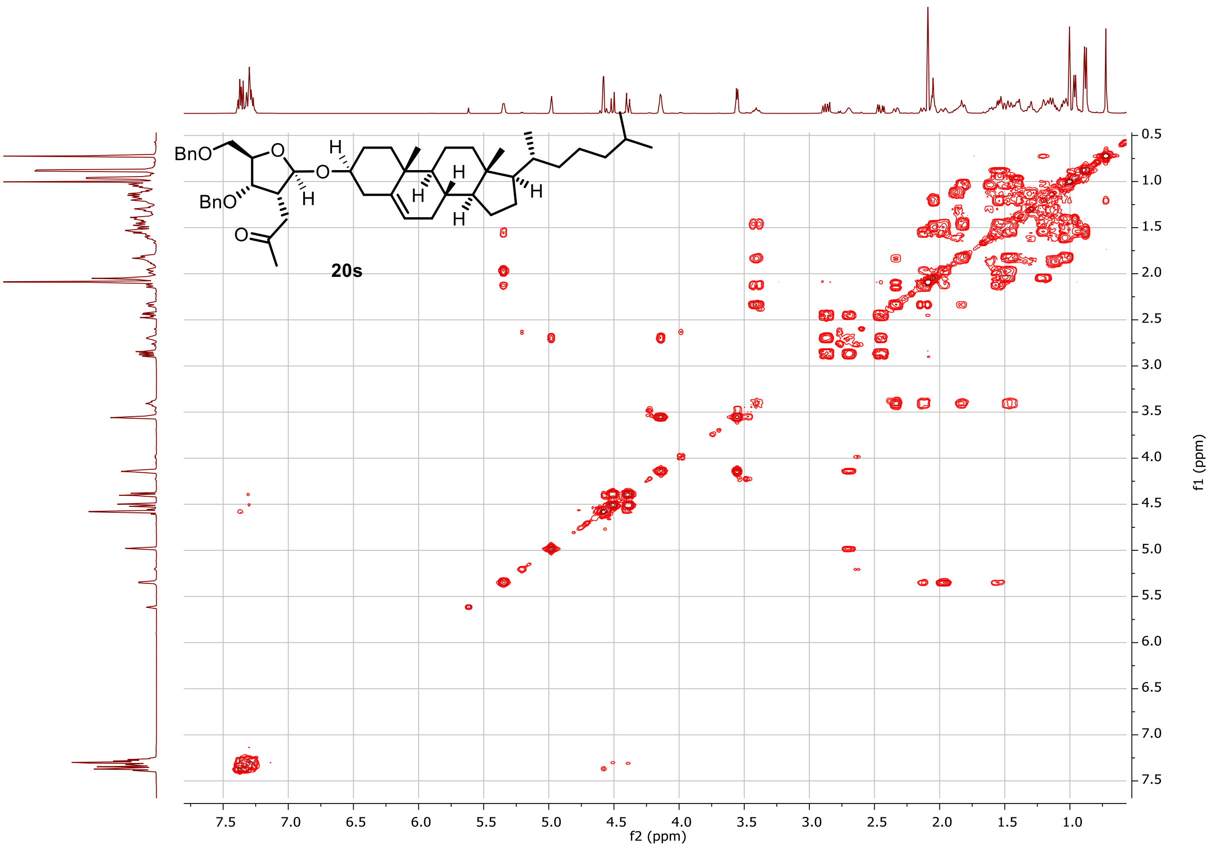

Supplementary Figure 171. COSY spectra for **20s**

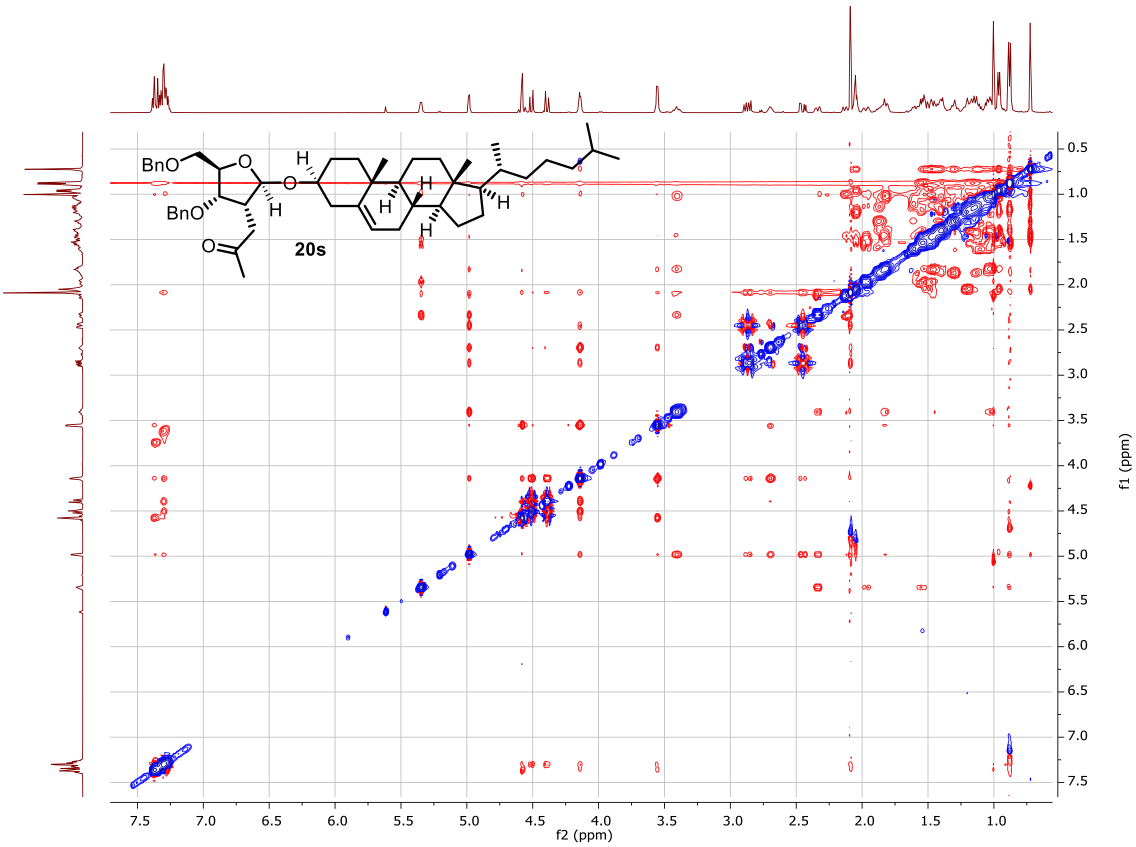

Supplementary Figure 172. NOESY spectra for **20s**

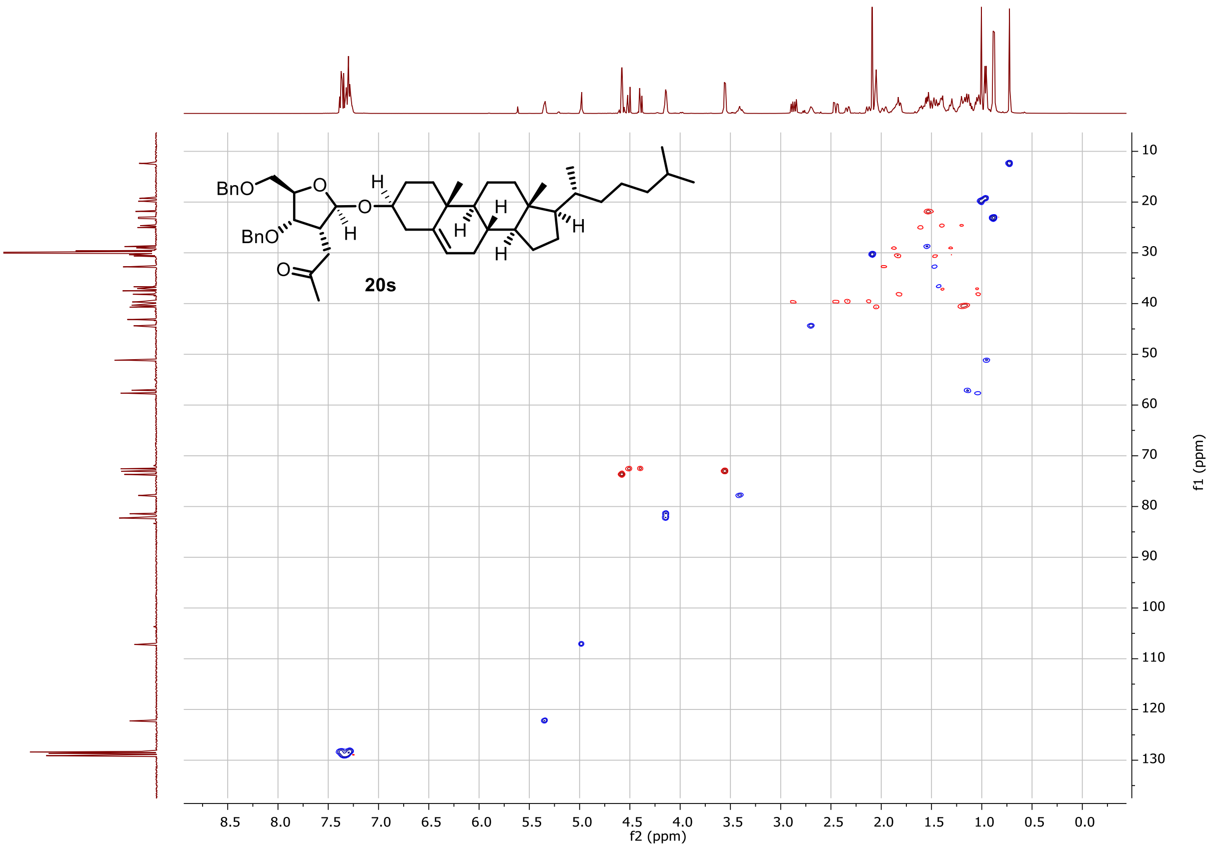

Supplementary Figure 173. HSQC spectra for **20s**

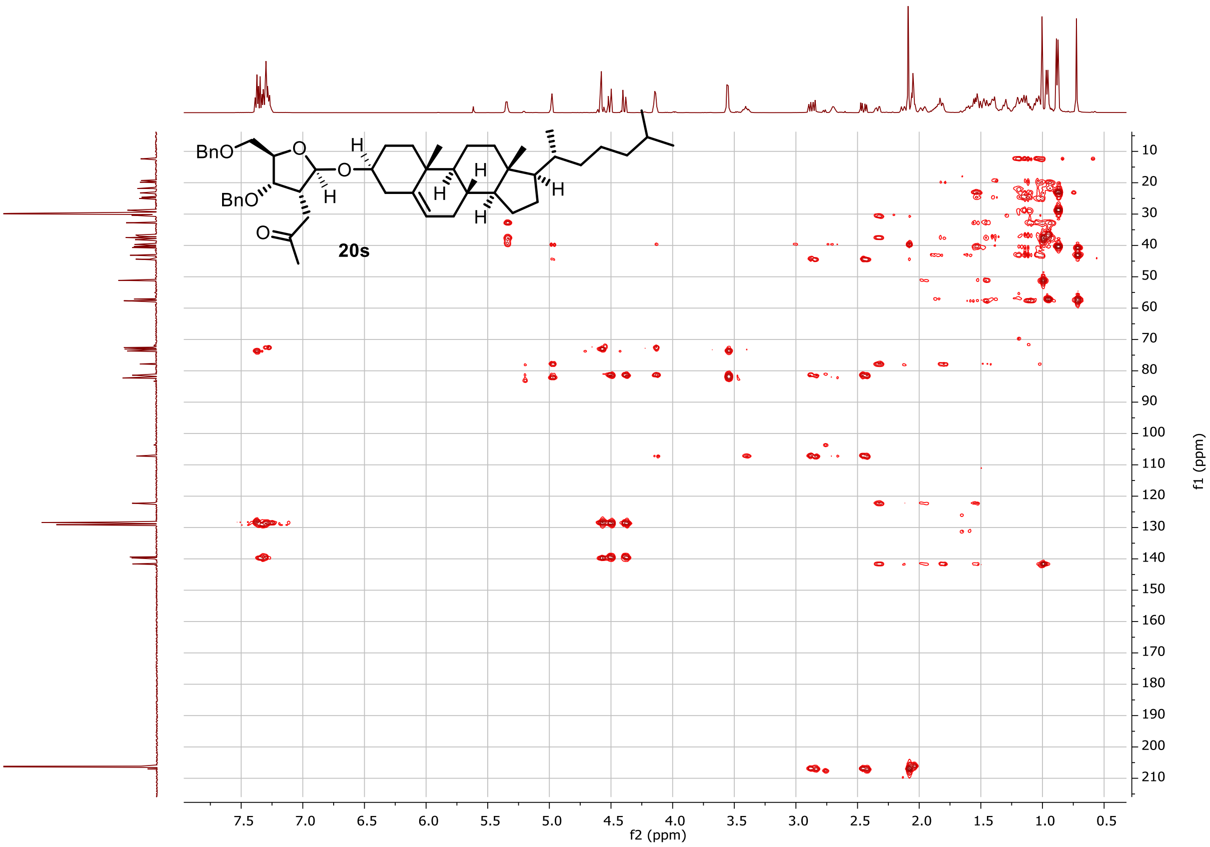

Supplementary Figure 174. HMBC spectra for **20s**

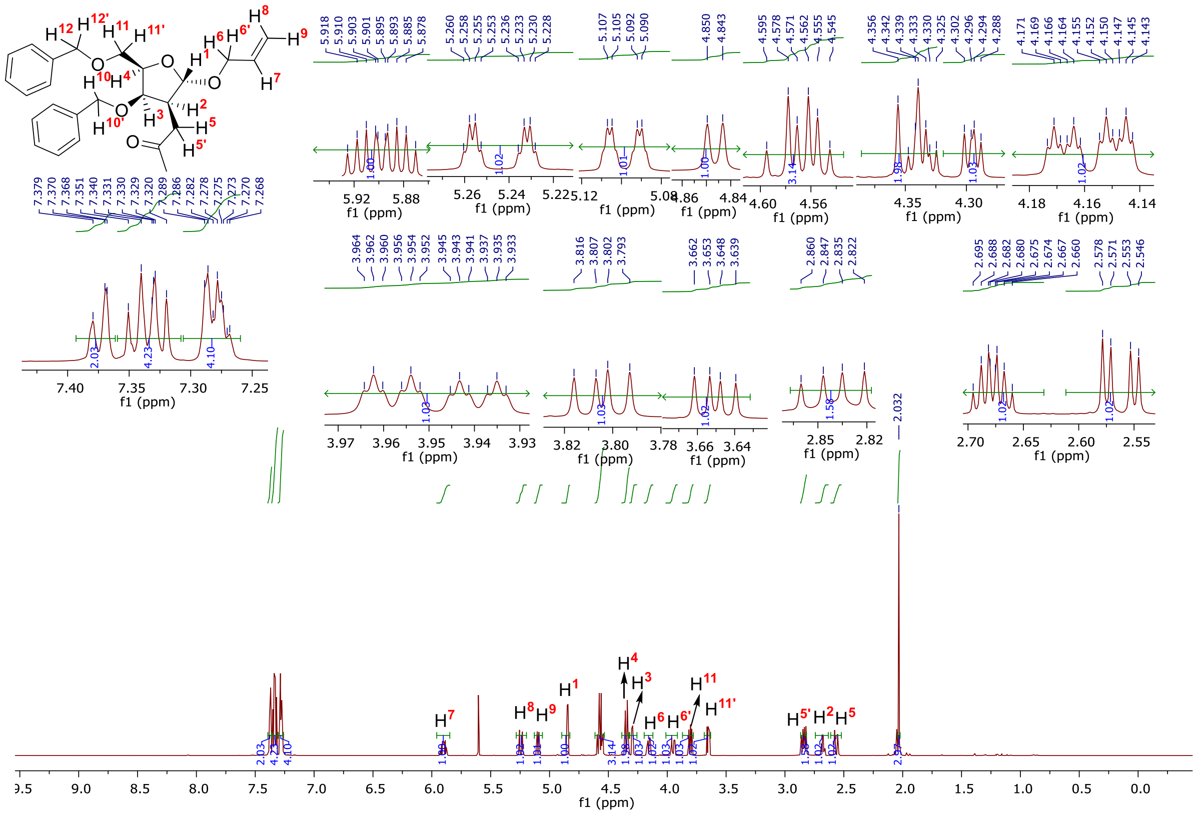

Supplementary Figure 175. ¹H spectra for **20t**

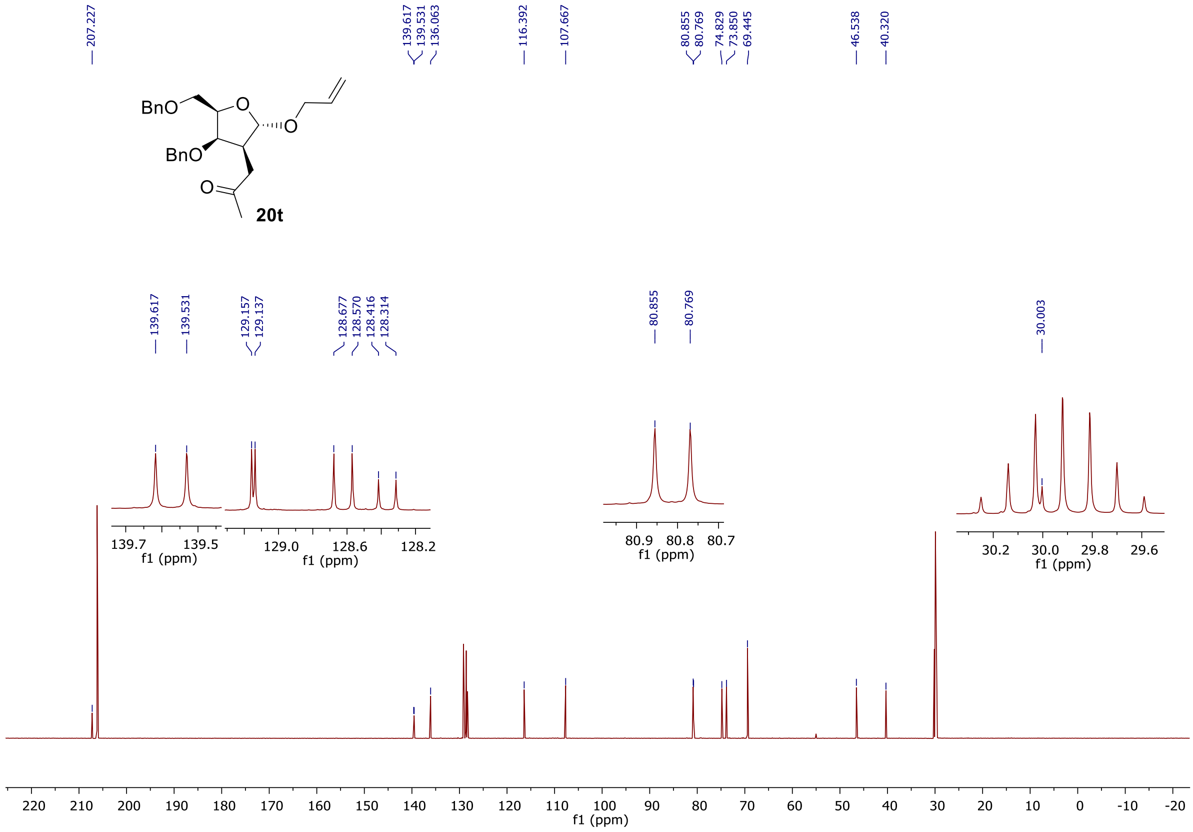

Supplementary Figure 176. ¹³C spectra for **20t**

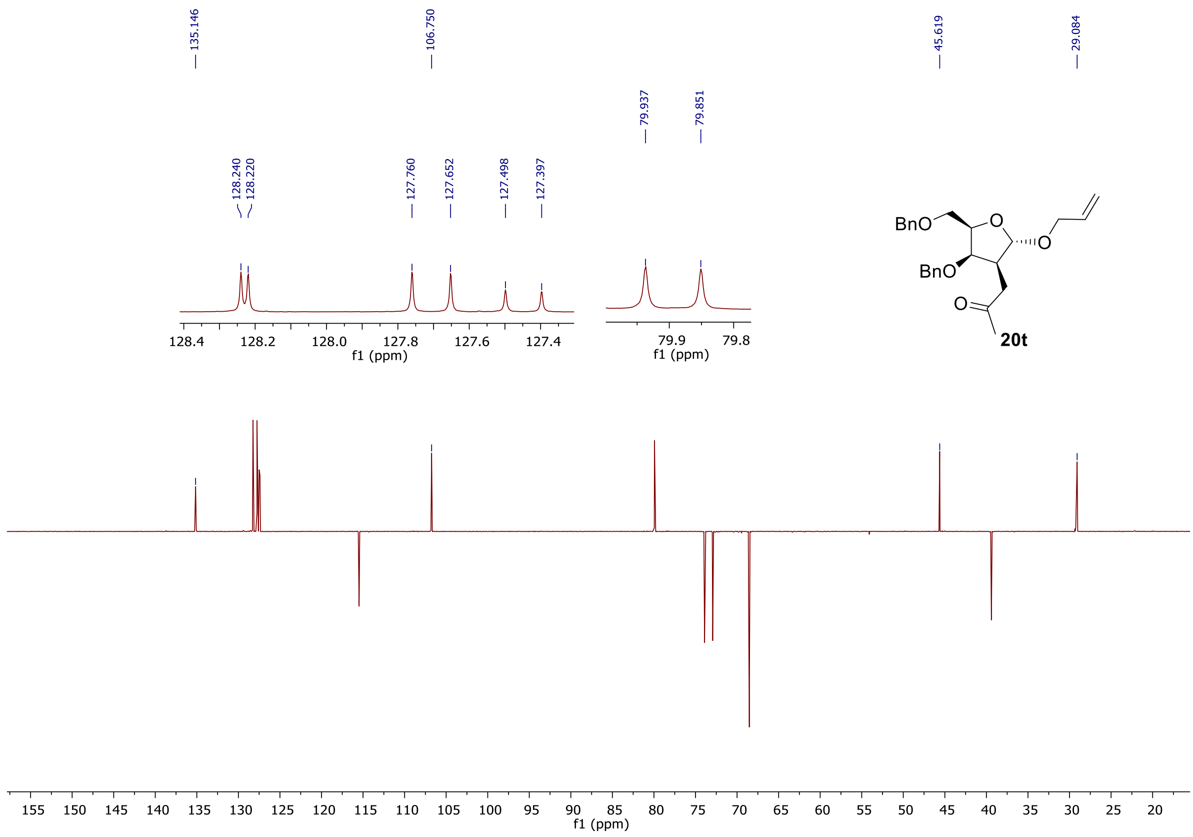

Supplementary Figure 177. DEPT spectra for **20t**

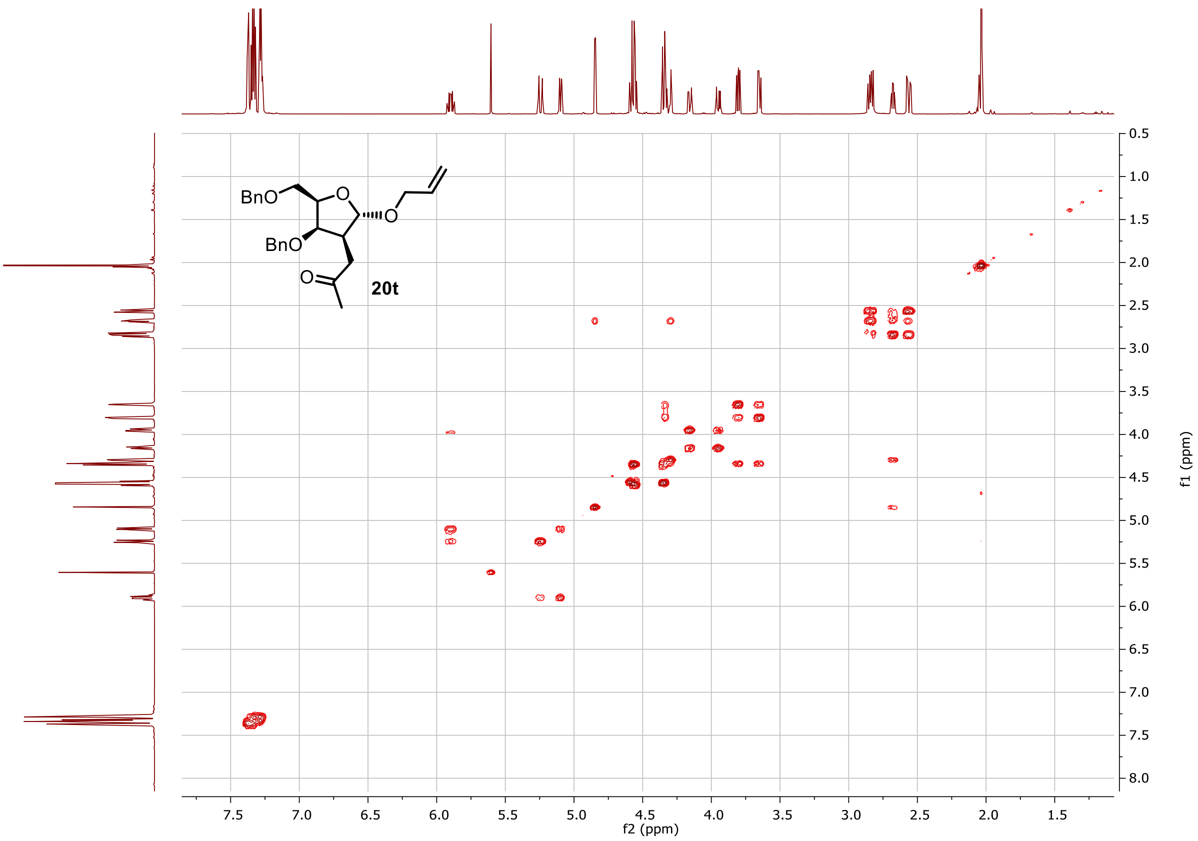

Supplementary Figure 178. COSY spectra for **20t**

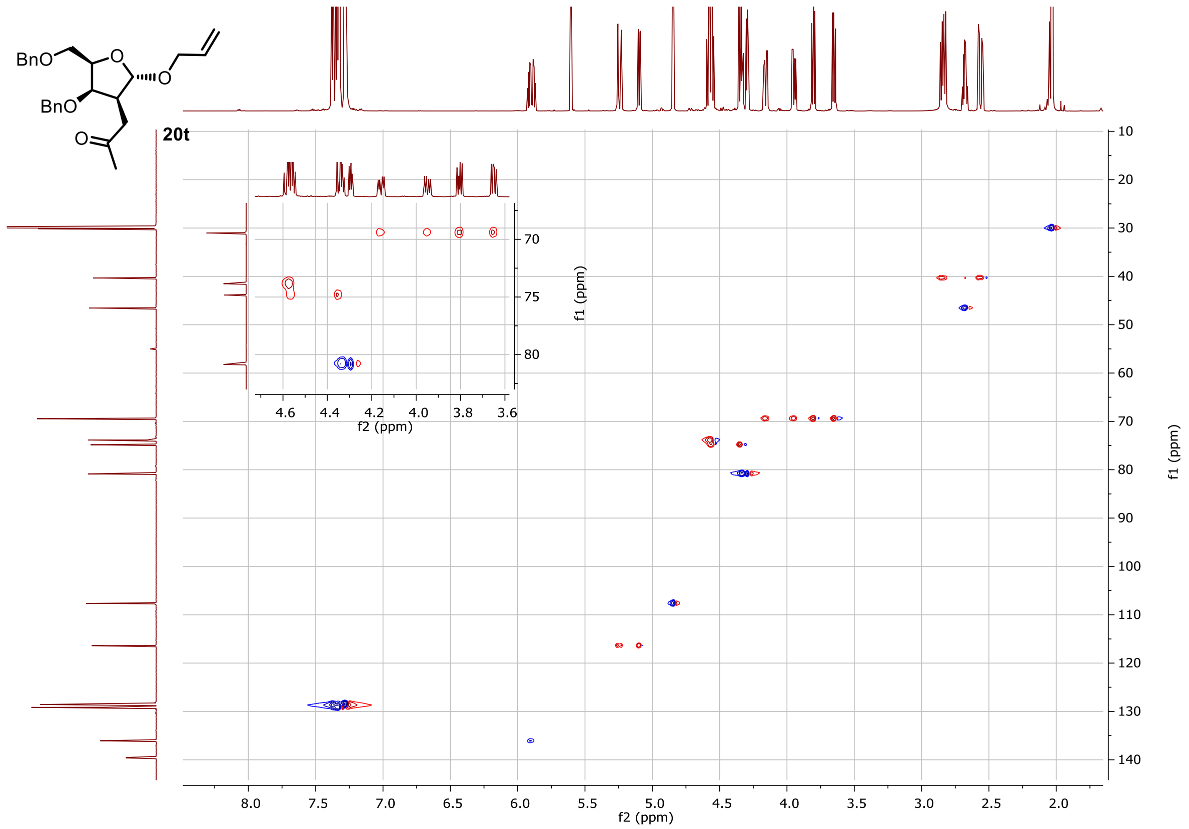

Supplementary Figure 179. HSQC spectra for **20t**

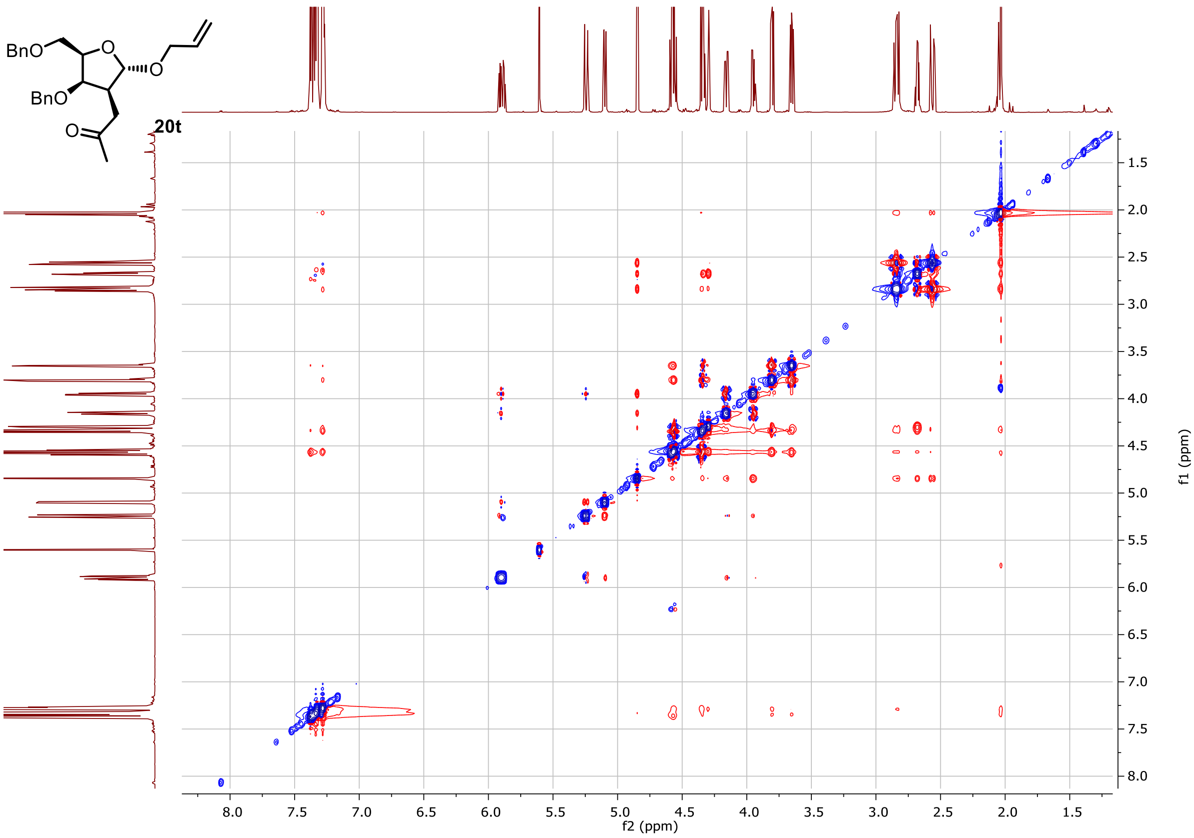

Supplementary Figure 180. NOESY spectra for **20t**

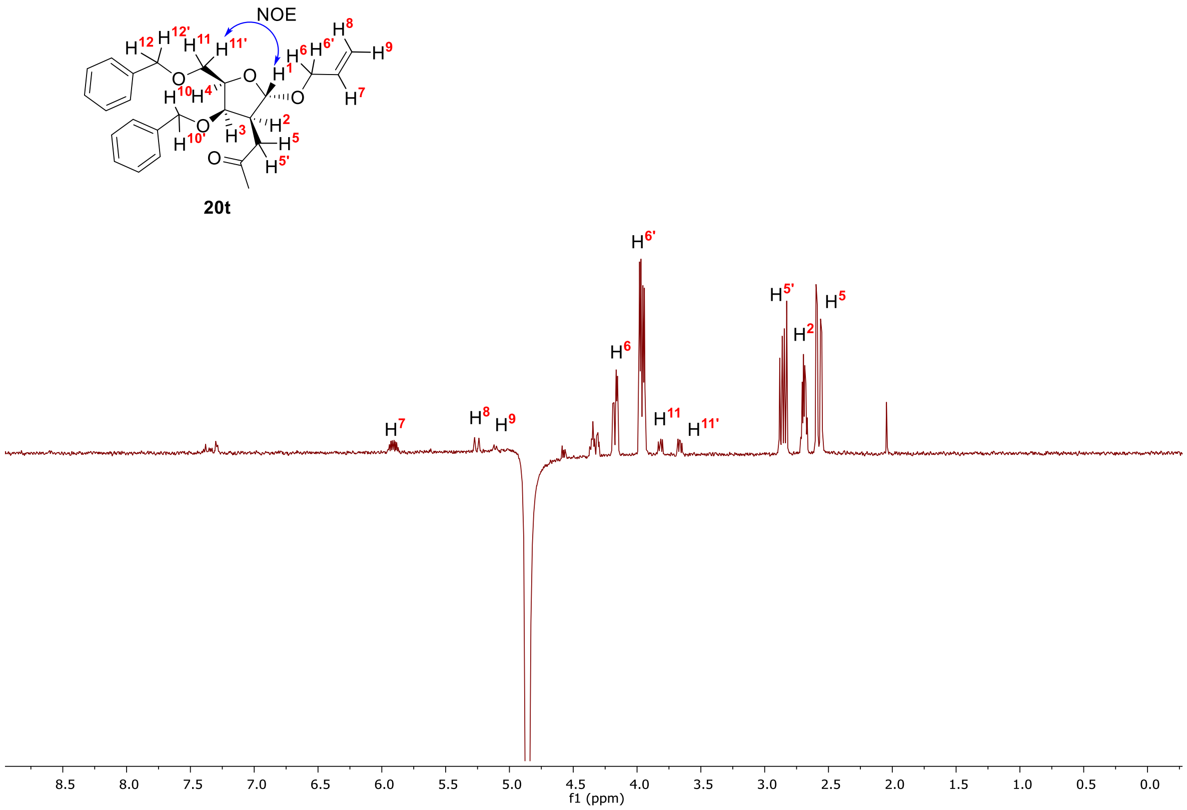

Supplementary Figure 181. 1D NOE spectra for **20t**

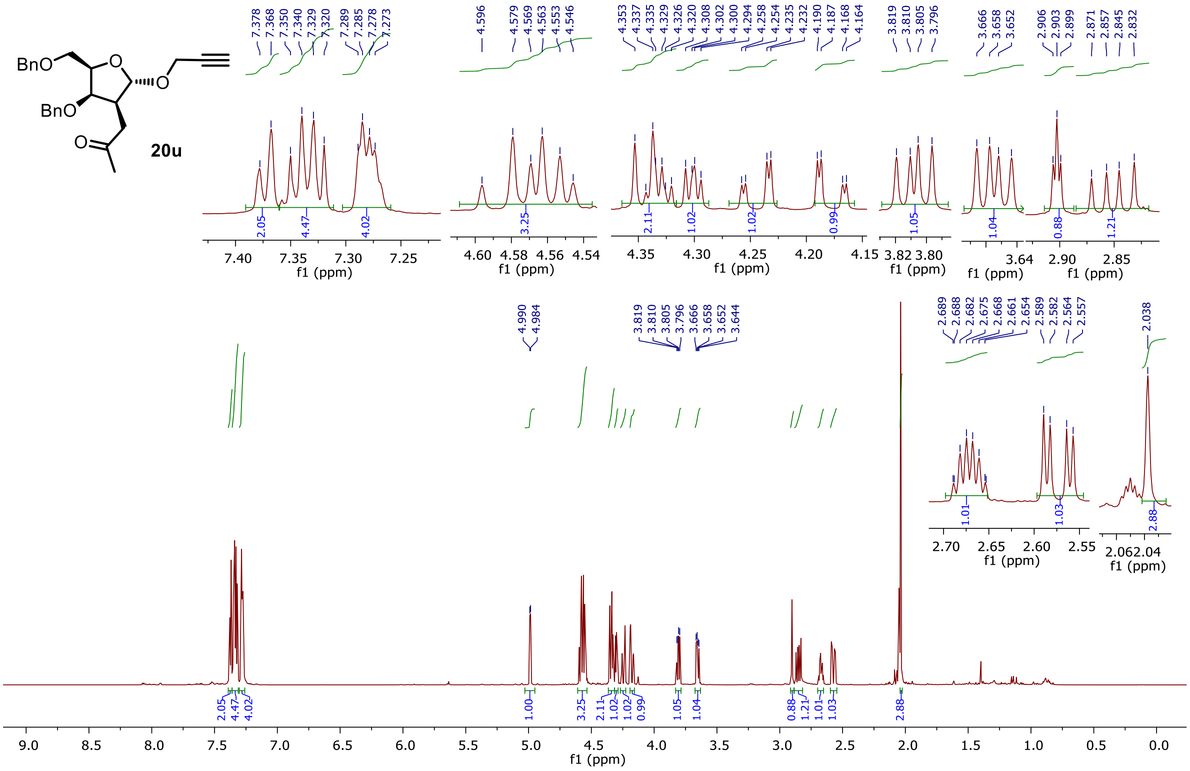

Supplementary Figure 182. ^{1}H spectra for **20u**

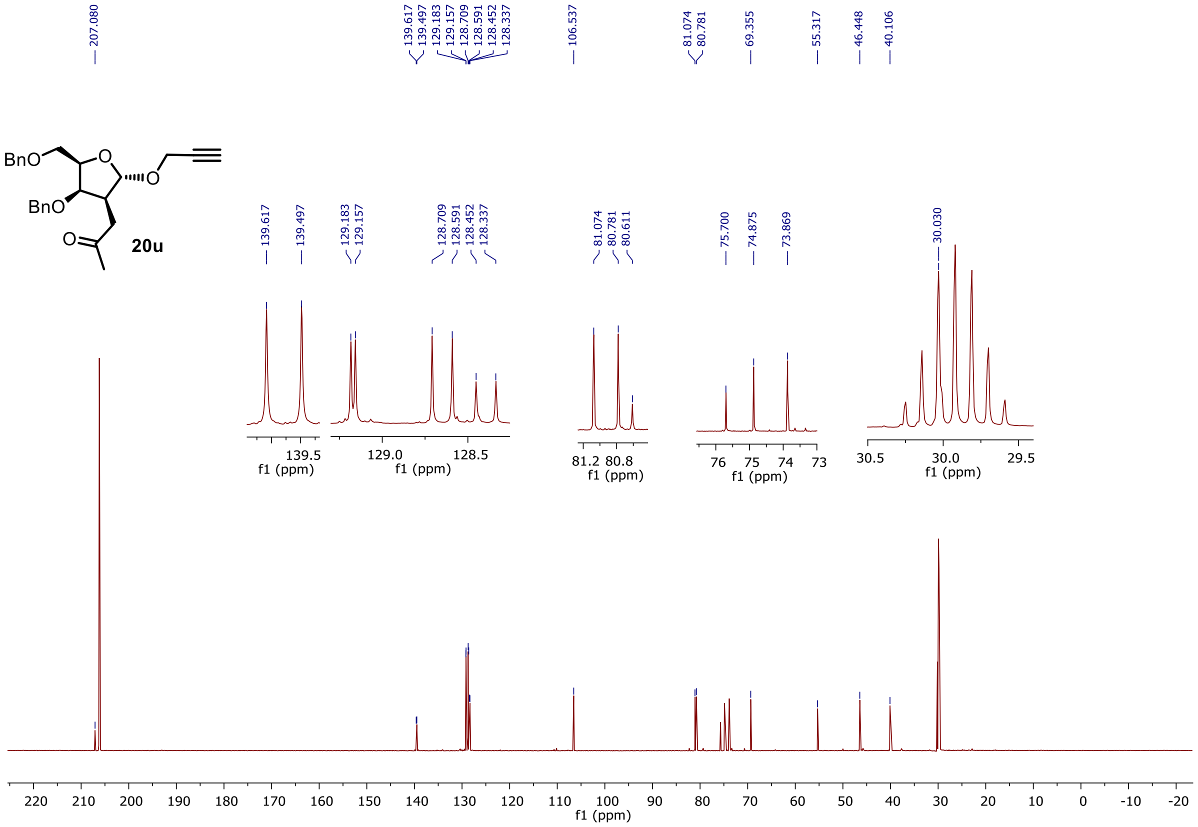

Supplementary Figure 183. ^{13}C spectra for **20u**

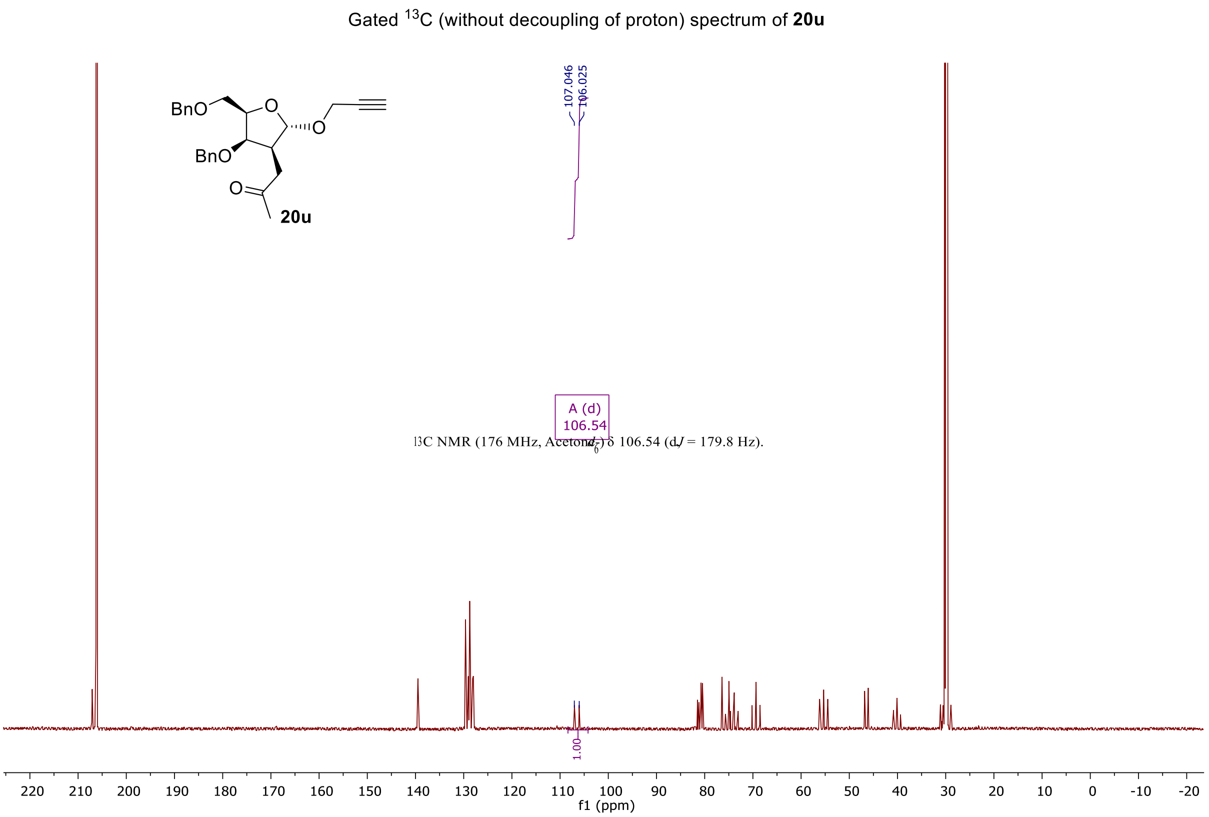

Supplementary Figure 184. Gated ^{13}C (with coupling of proton) spectra for **20u**

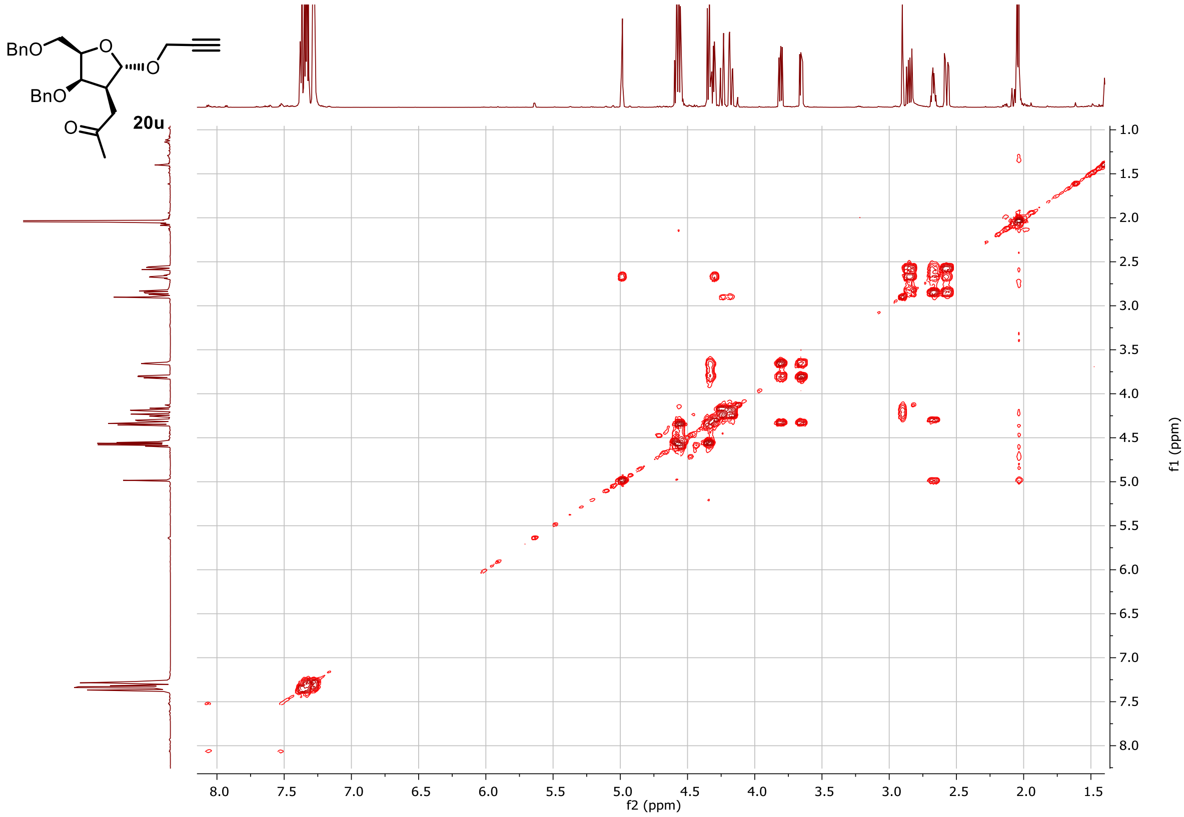

Supplementary Figure 185. COSY spectra for **20u**

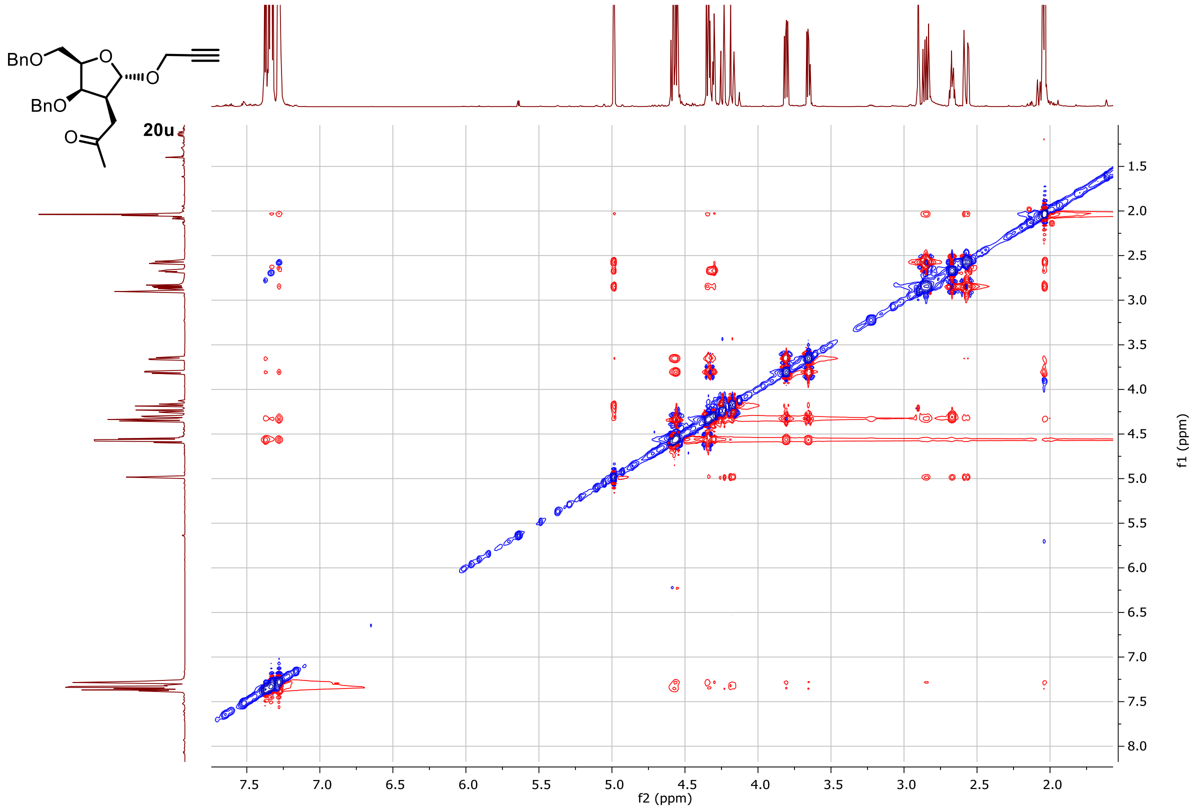

Supplementary Figure 186. NOESY spectra for **20u**

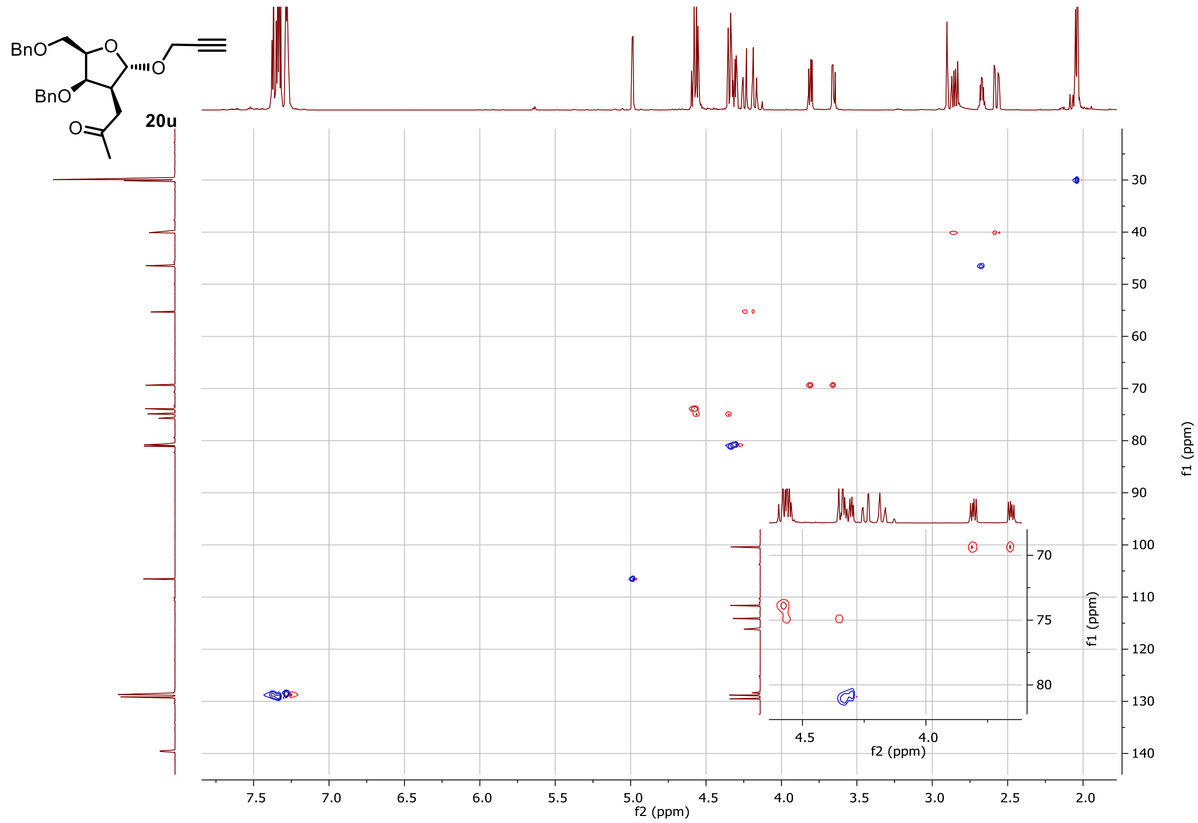

Supplementary Figure 187. HSQC spectra for **20u**

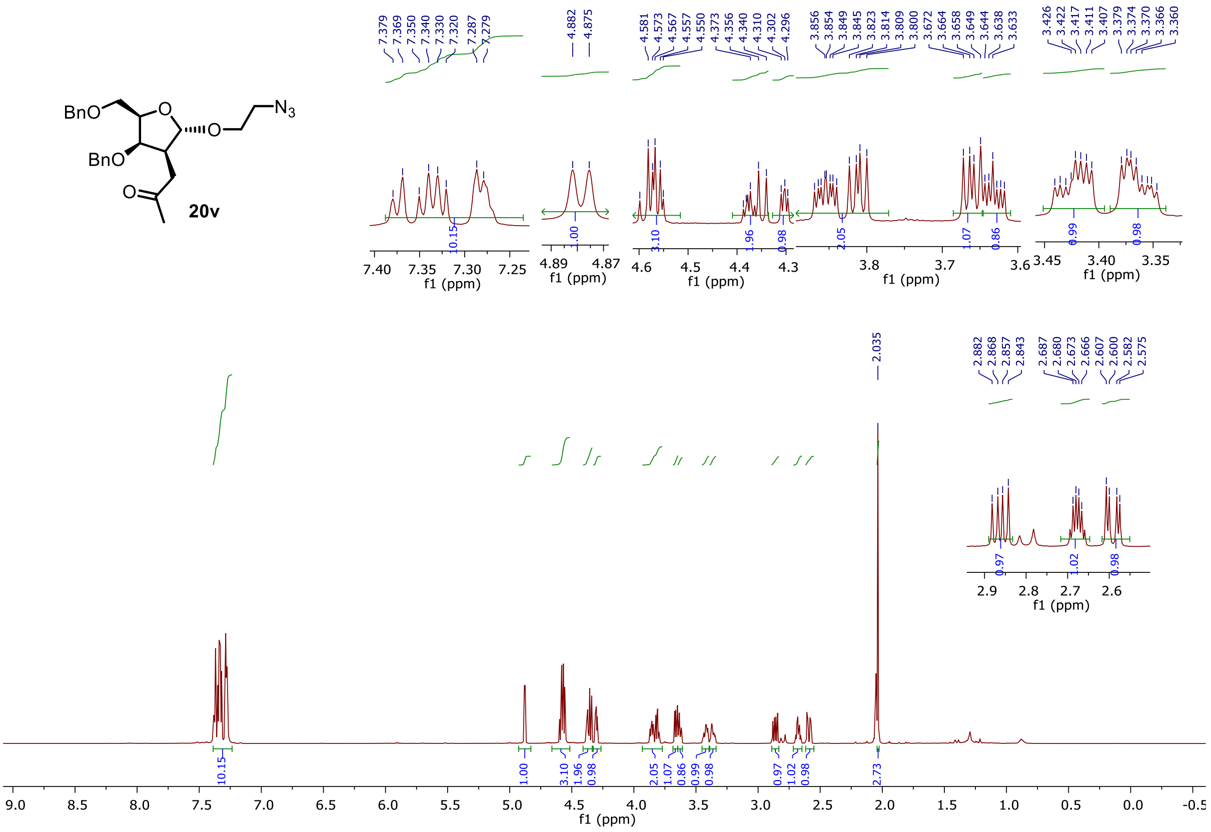

Supplementary Figure 188. ^{1}H spectra for **20v**

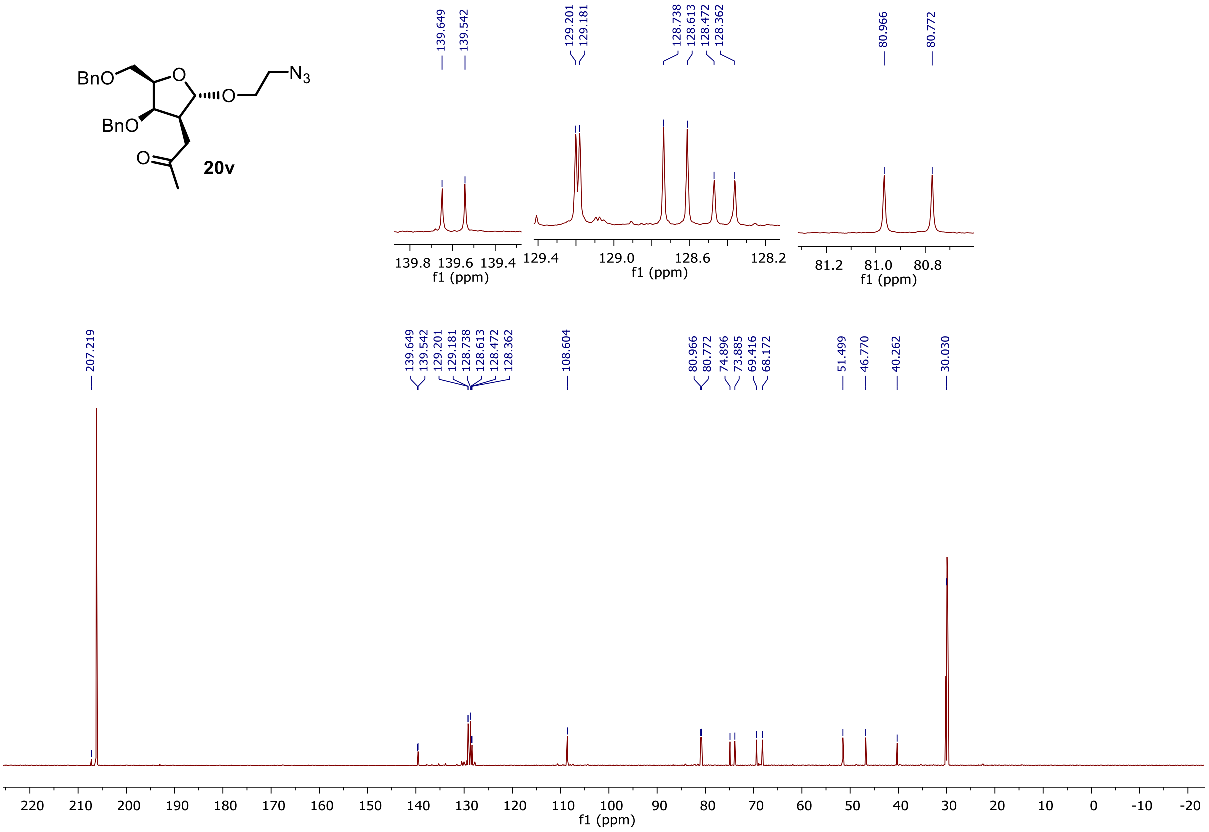

Supplementary Figure 189. ^{13}C spectra for **20v**

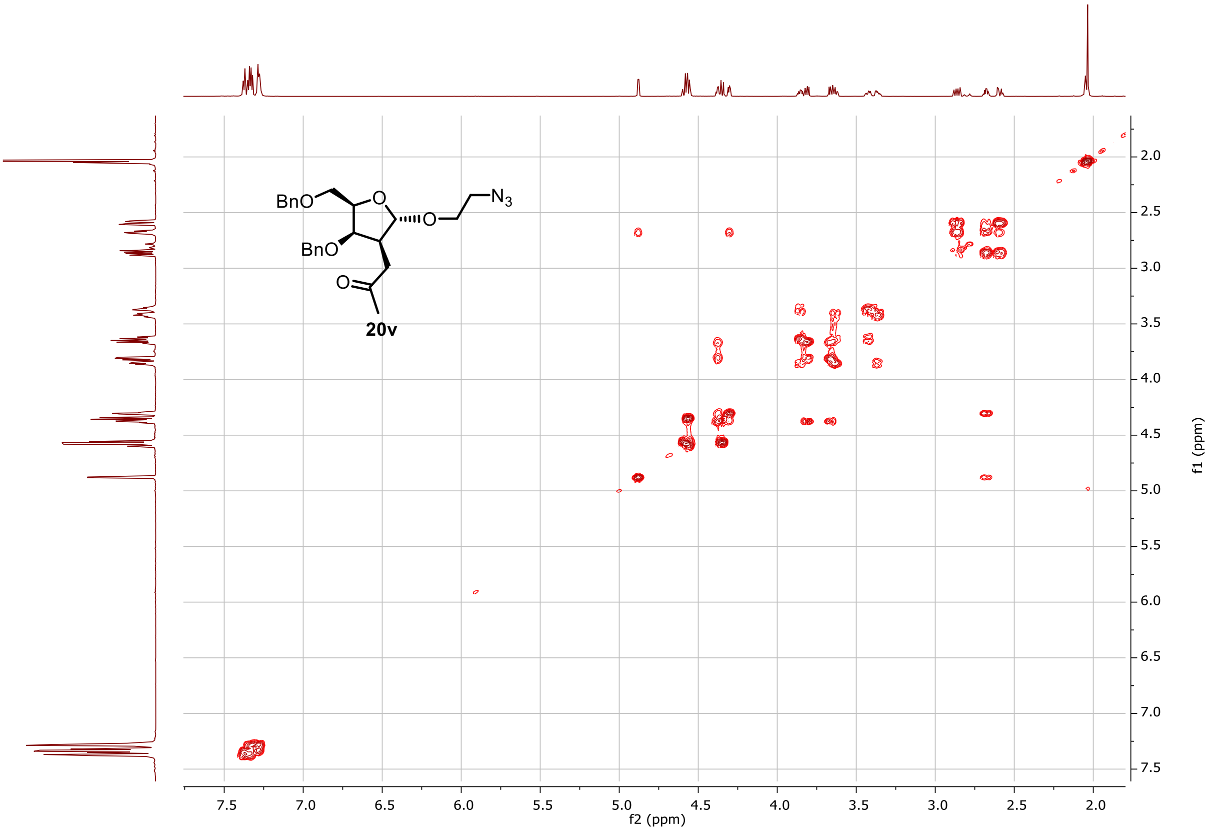

Supplementary Figure 190. COSY spectra for **20v**

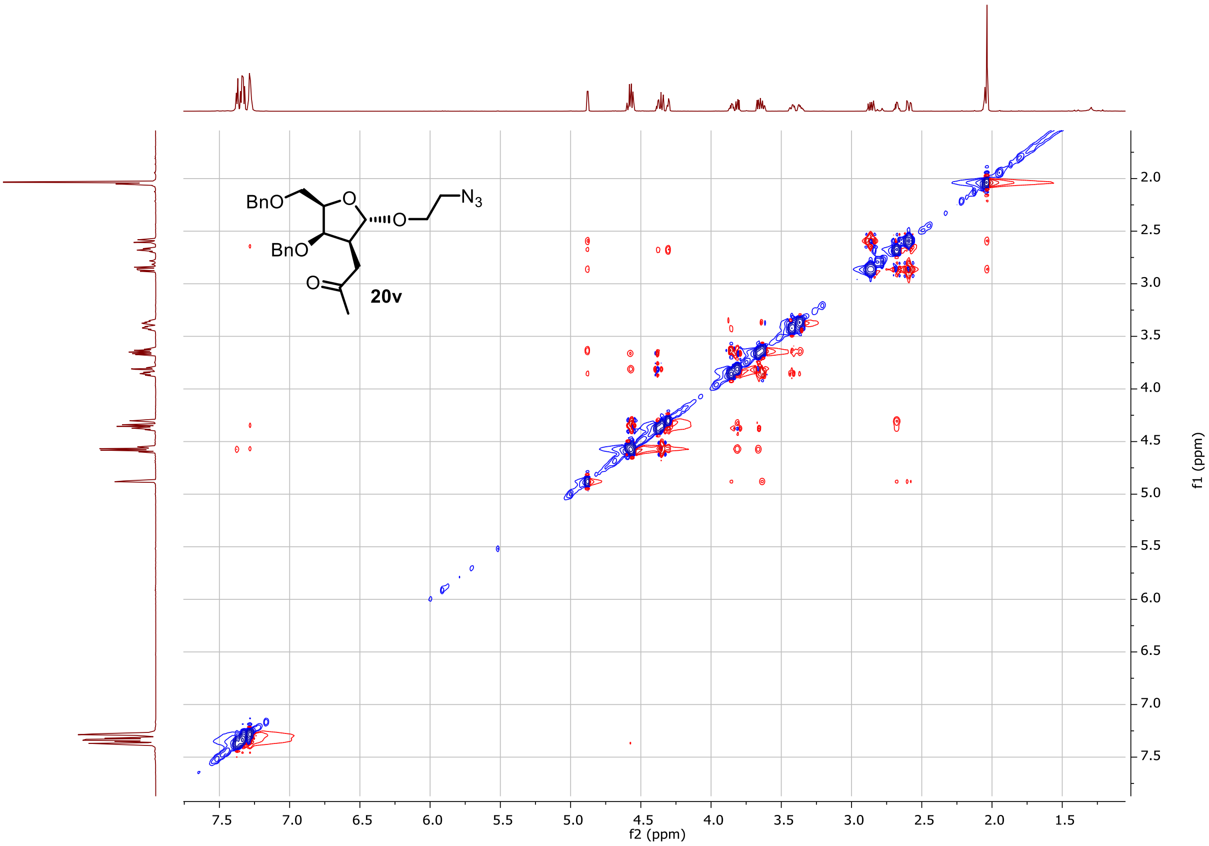

Supplementary Figure 191. NOESY spectra for **20v**

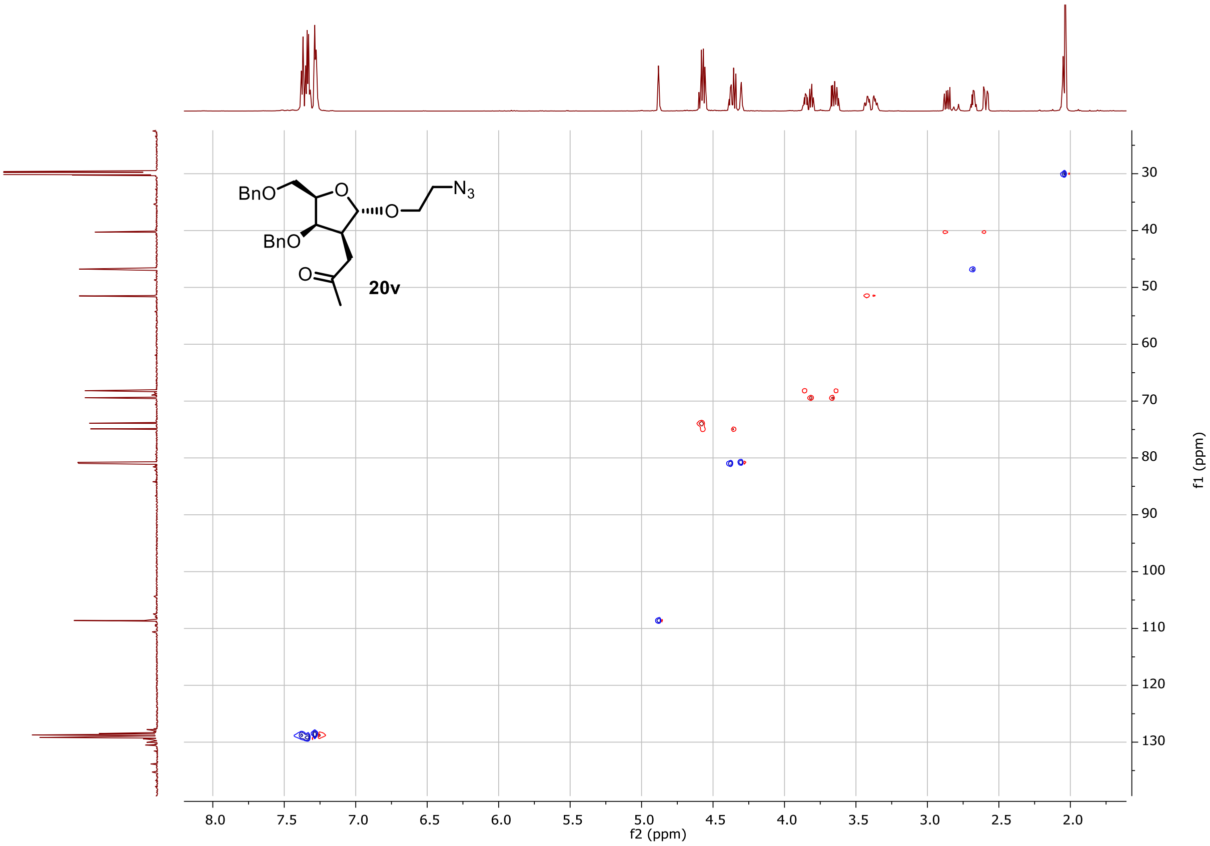

Supplementary Figure 192. HSQC spectra for **20v**

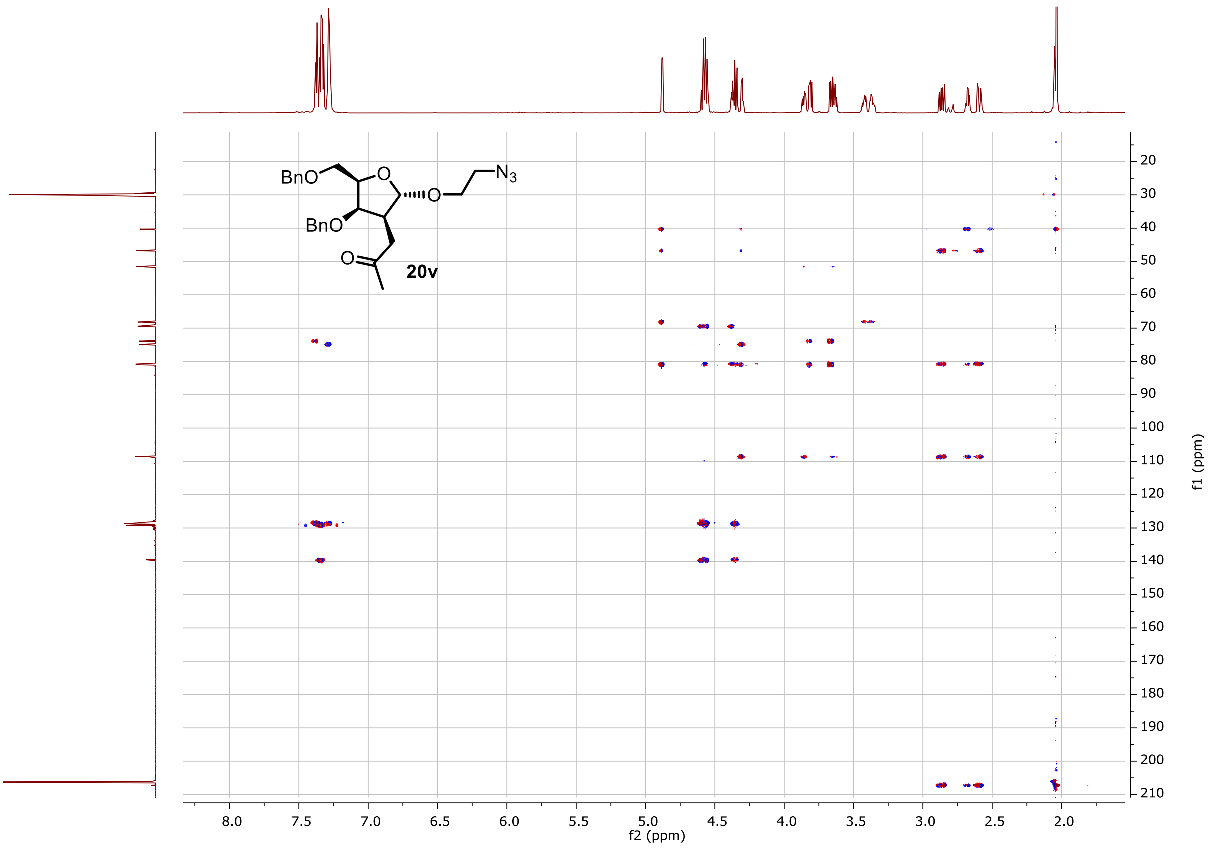

Supplementary Figure 193. HMBC spectra for **20v**

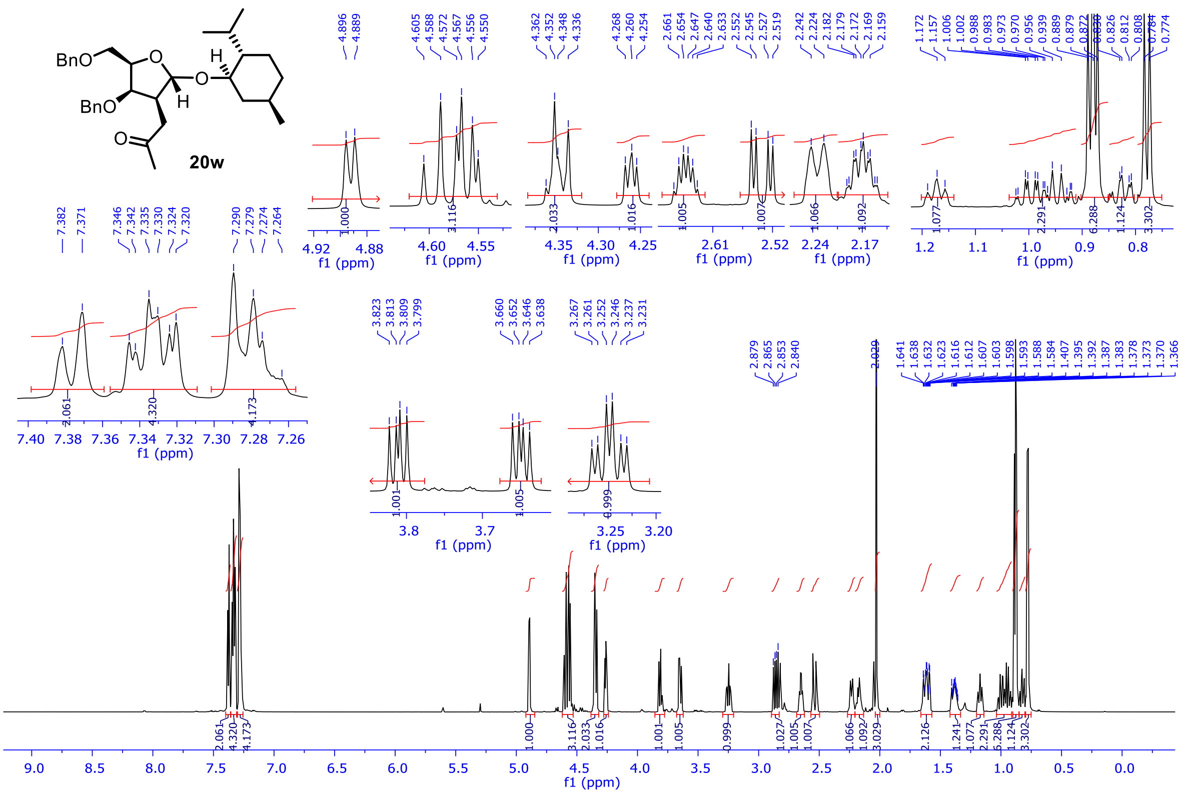

Supplementary Figure 194. ^{1}H spectra for **20w**

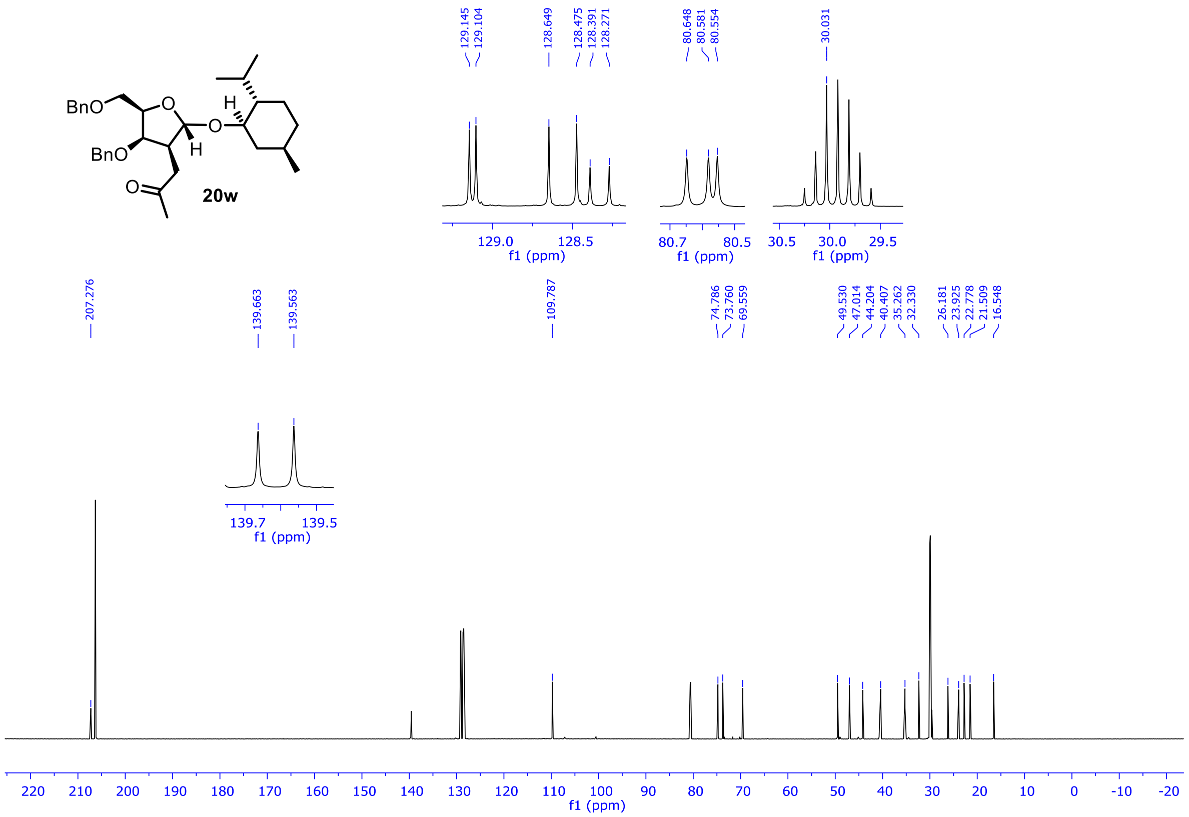

Supplementary Figure 195. ^{13}C spectra for **20w**

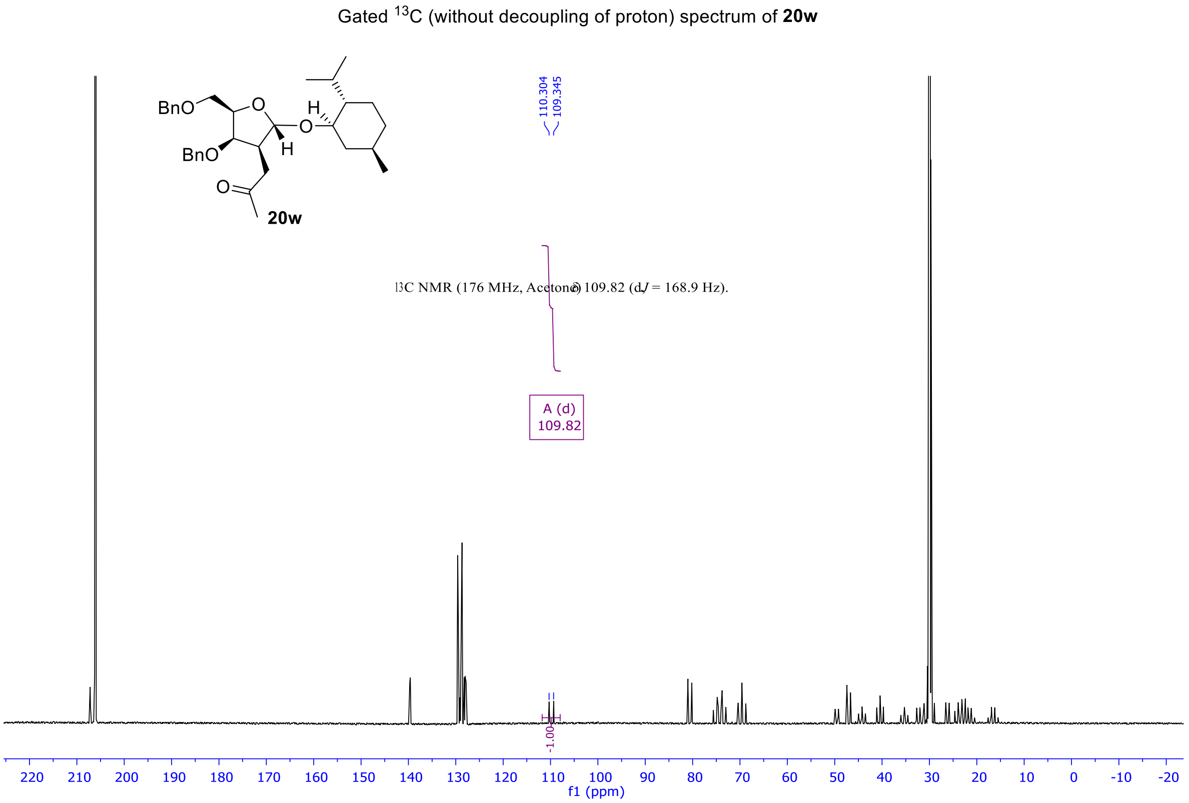

Supplementary Figure 196. Gated ^{13}C (with coupling of proton) spectra for **20w**

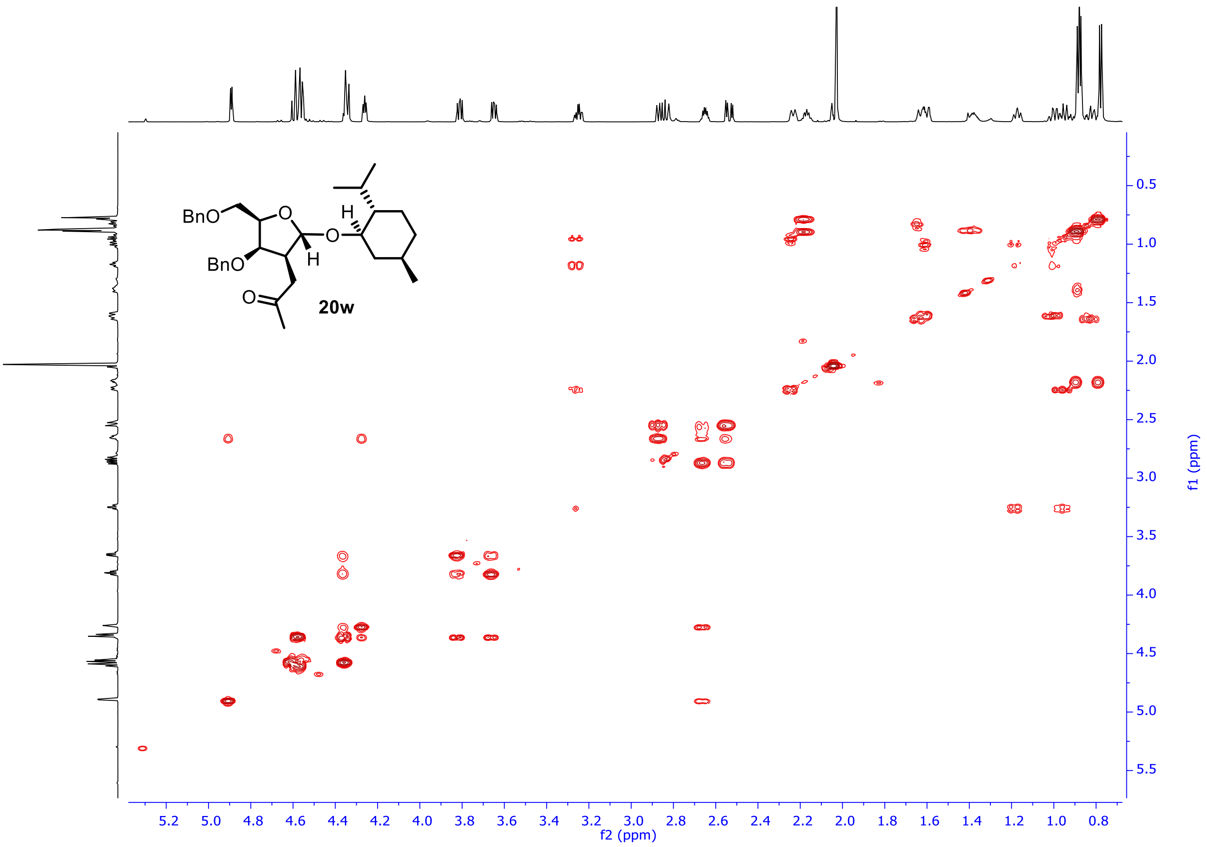

Supplementary Figure 197. COSY spectra for **20w**

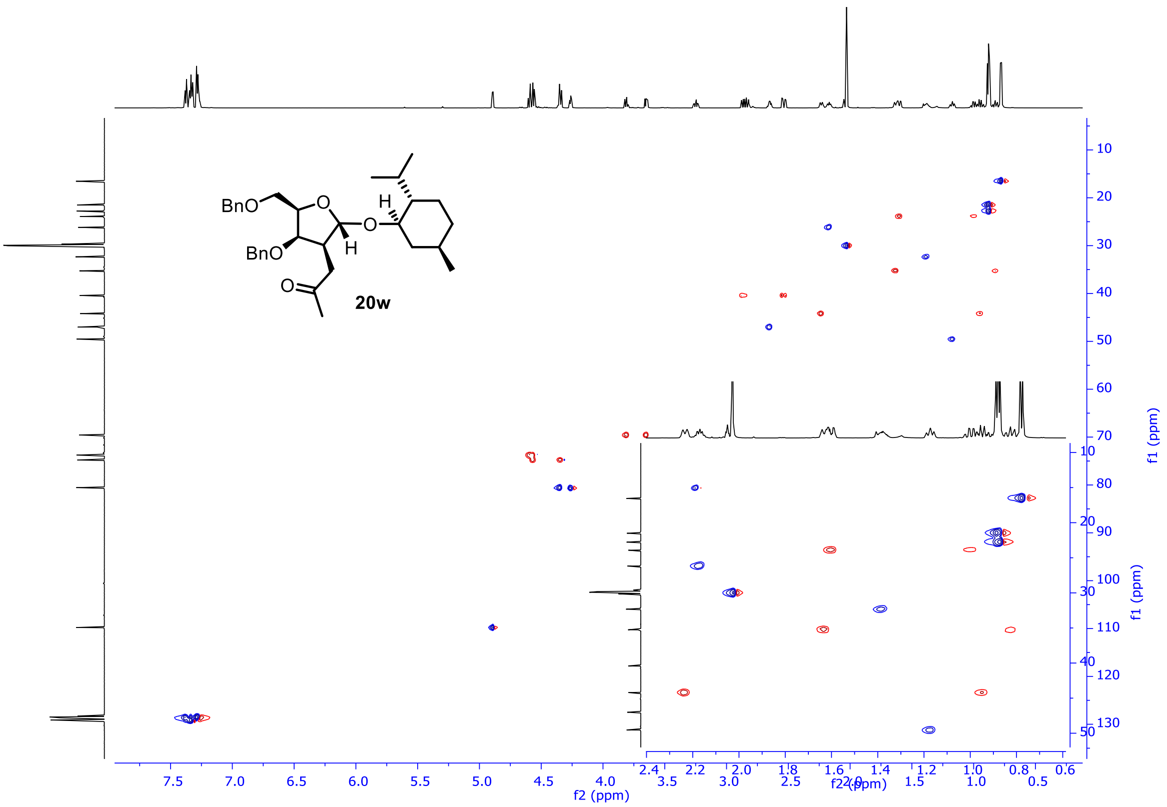

Supplementary Figure 198. HSQC spectra for **20w**

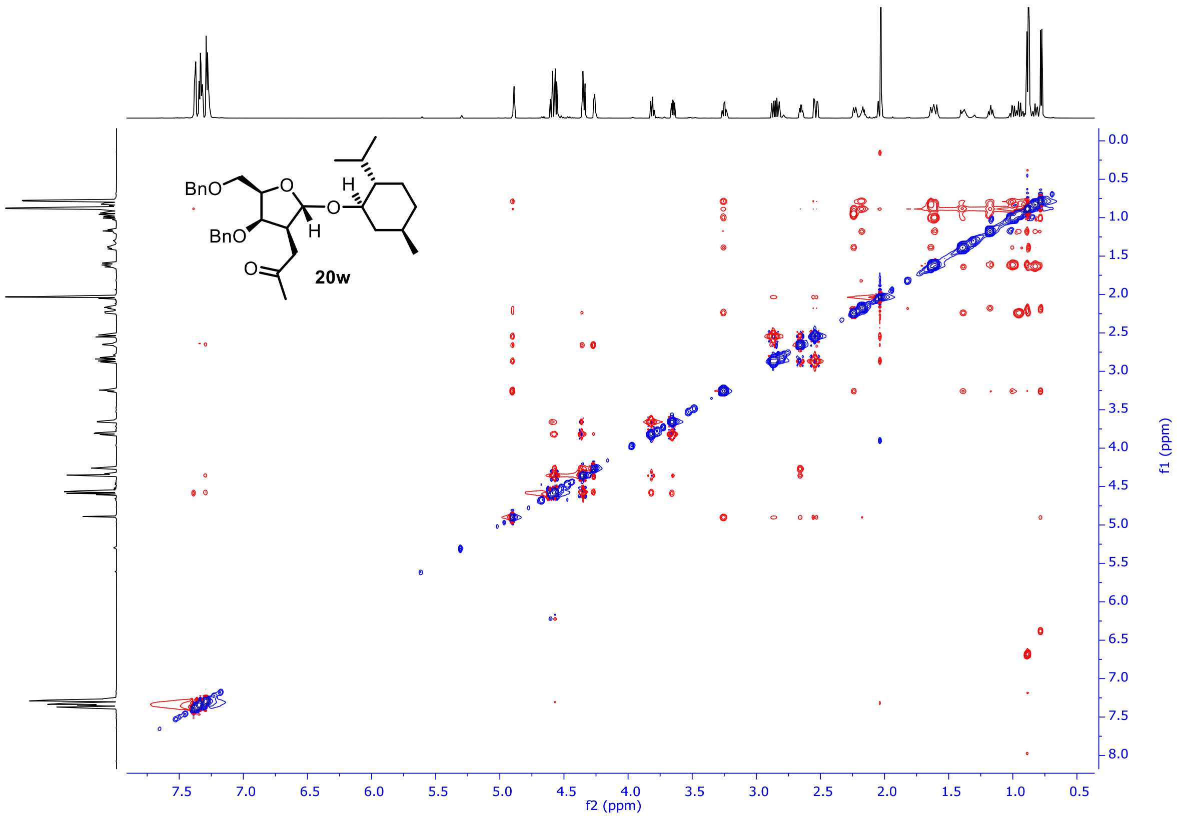

Supplementary Figure 199. NOESY spectra for **20w**

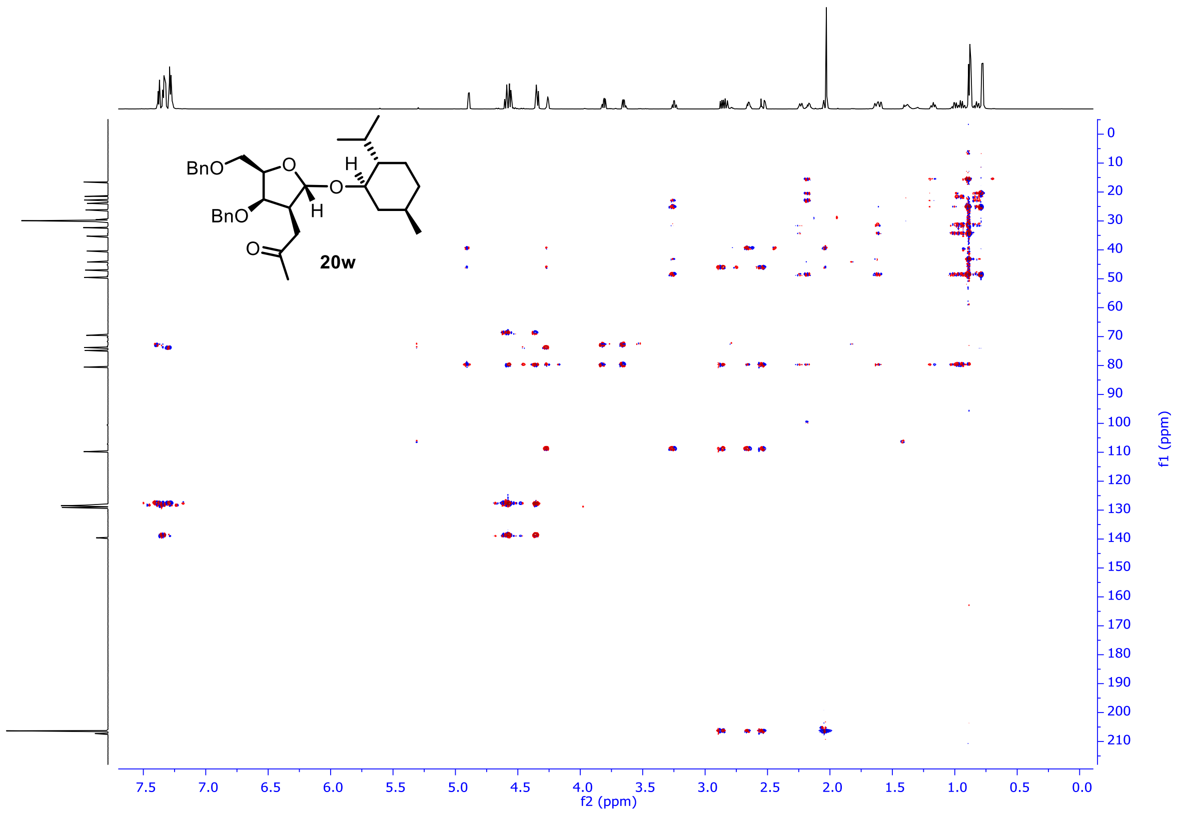

Supplementary Figure 200. HMBC spectra for **20w**

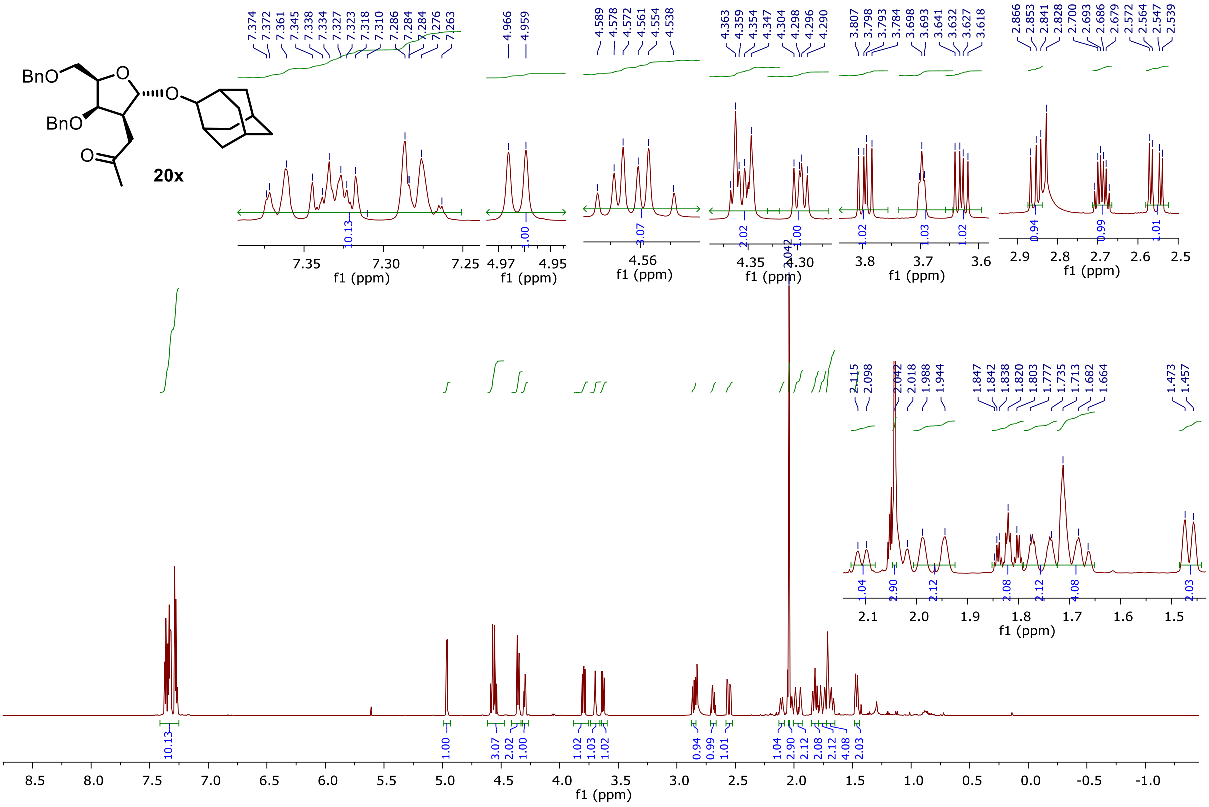

Supplementary Figure 201. ^{1}H spectra for **20x**

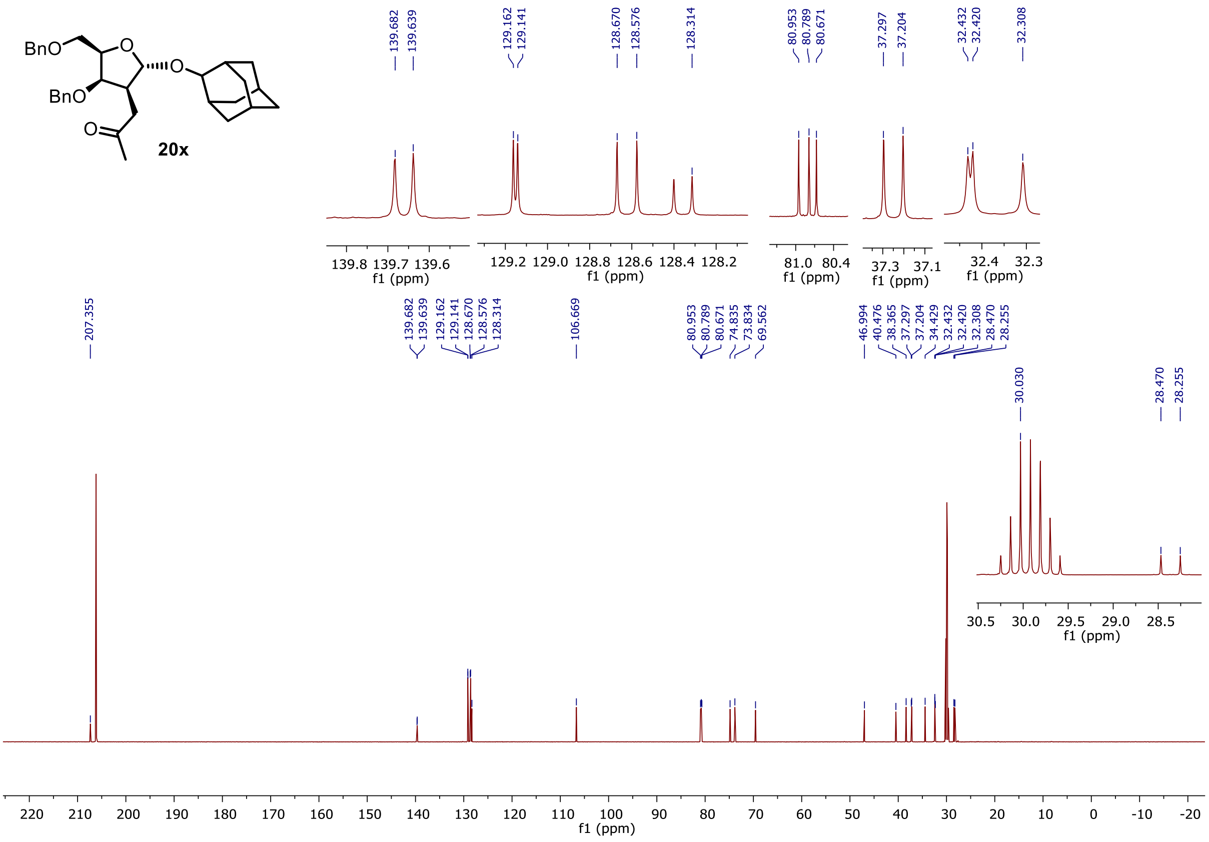

Supplementary Figure 202. ^{13}C spectra for **20x**

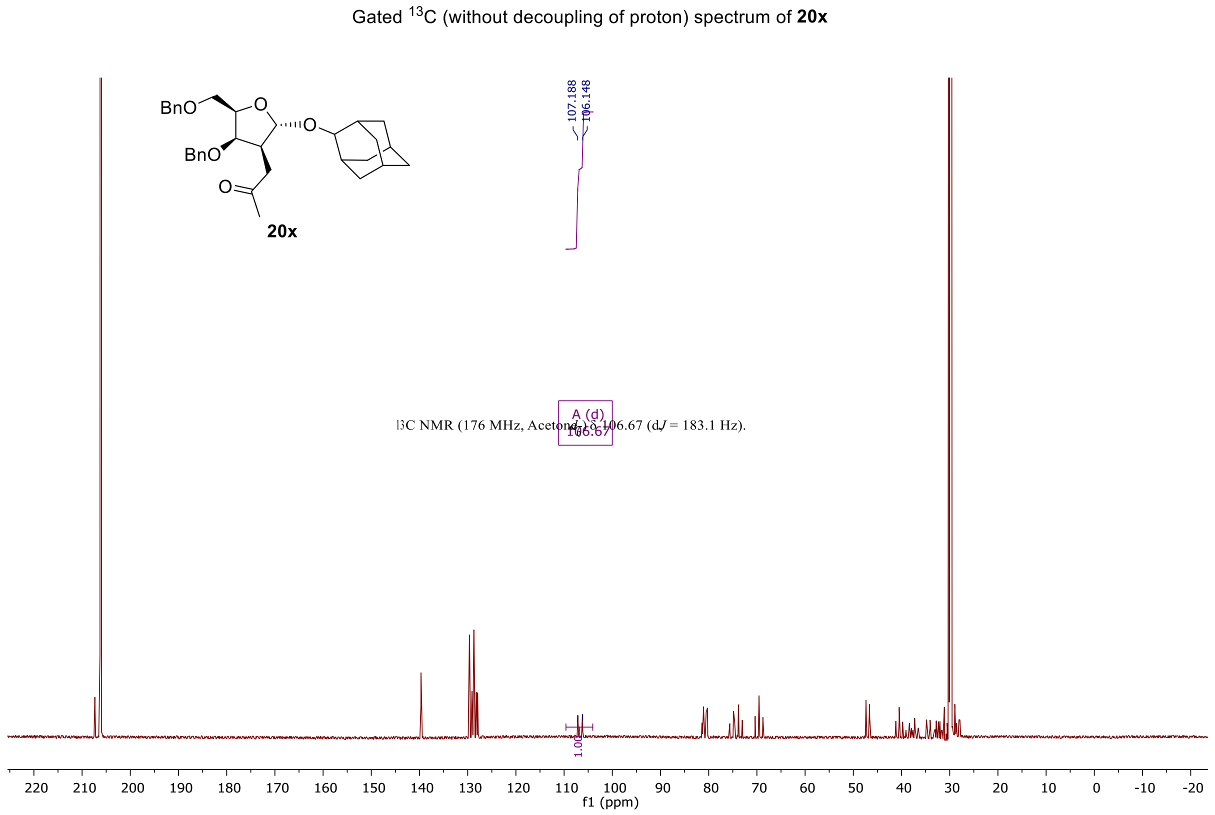

Supplementary Figure 203. Gated ^{13}C (with coupling of proton) spectra for **20x**

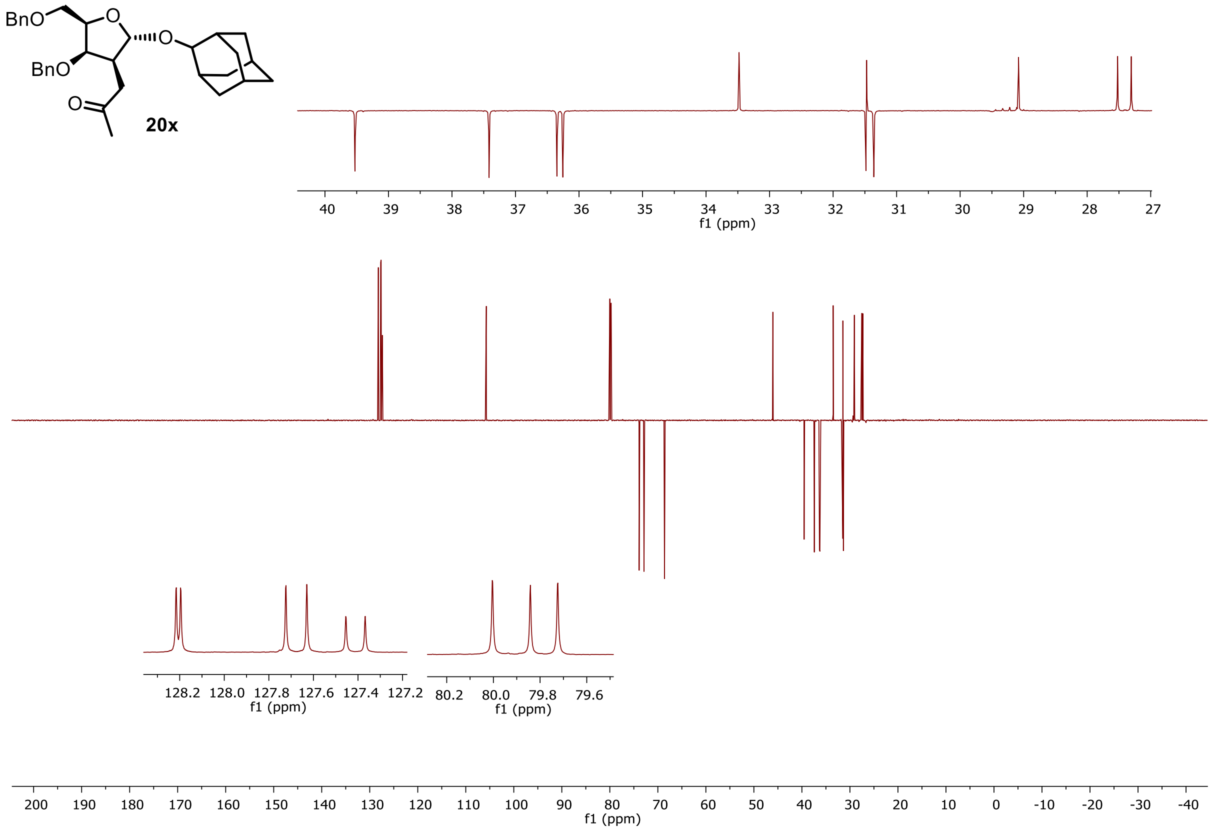

Supplementary Figure 204. DEPT spectra for **20x**

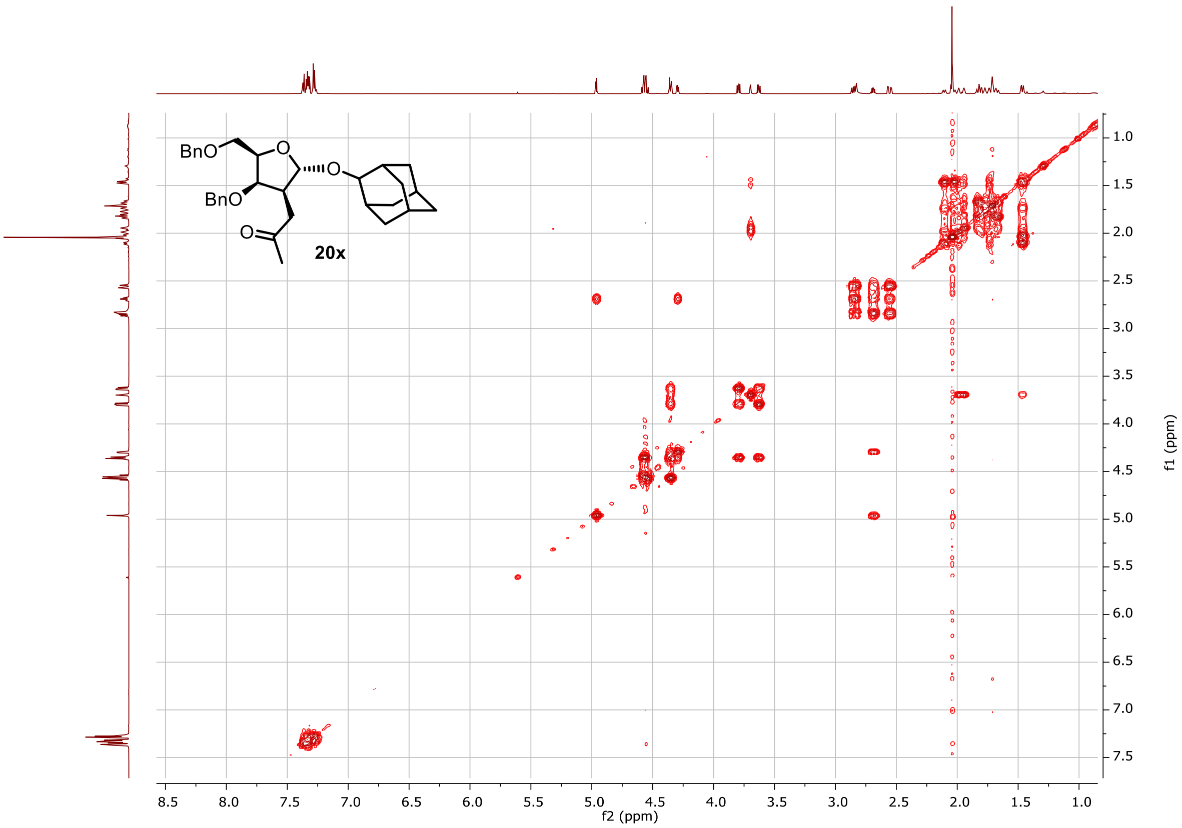

Supplementary Figure 205. COSY spectra for **20x**

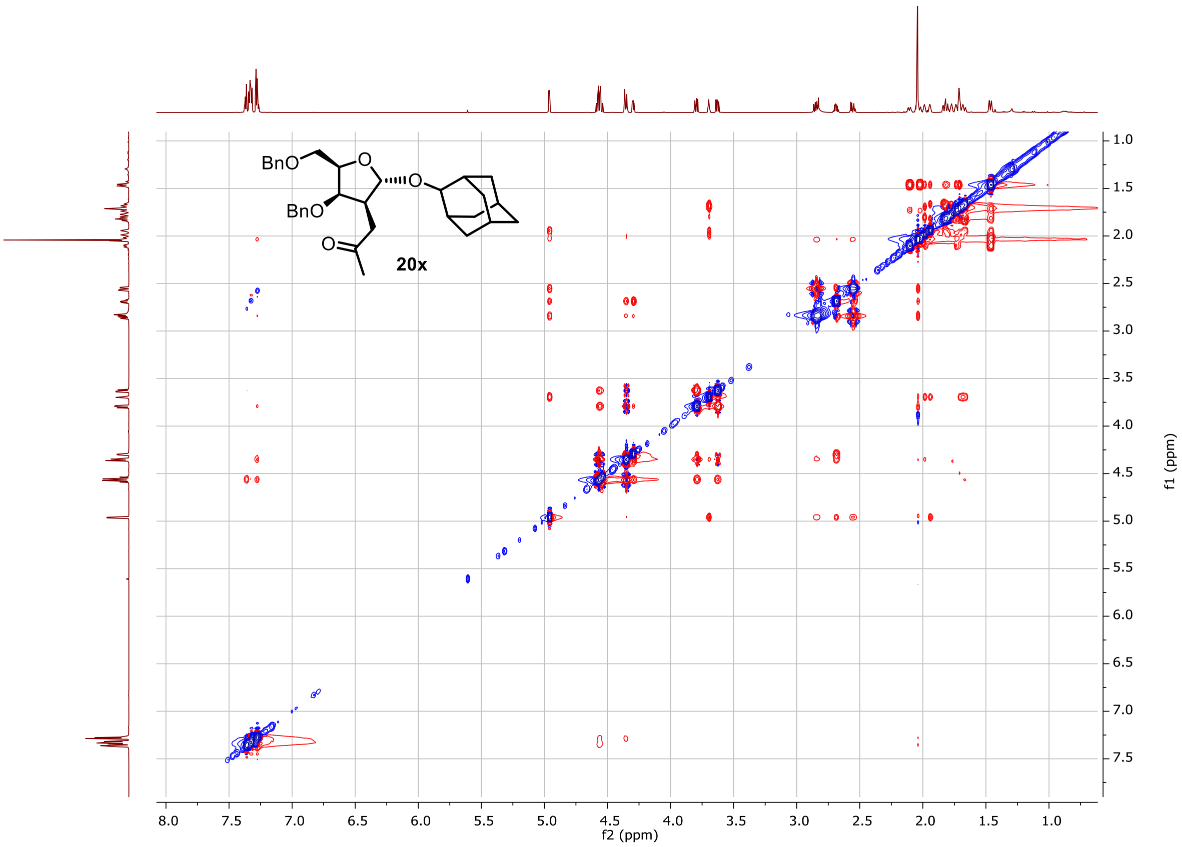

Supplementary Figure 206. NOESY spectra for **20x**

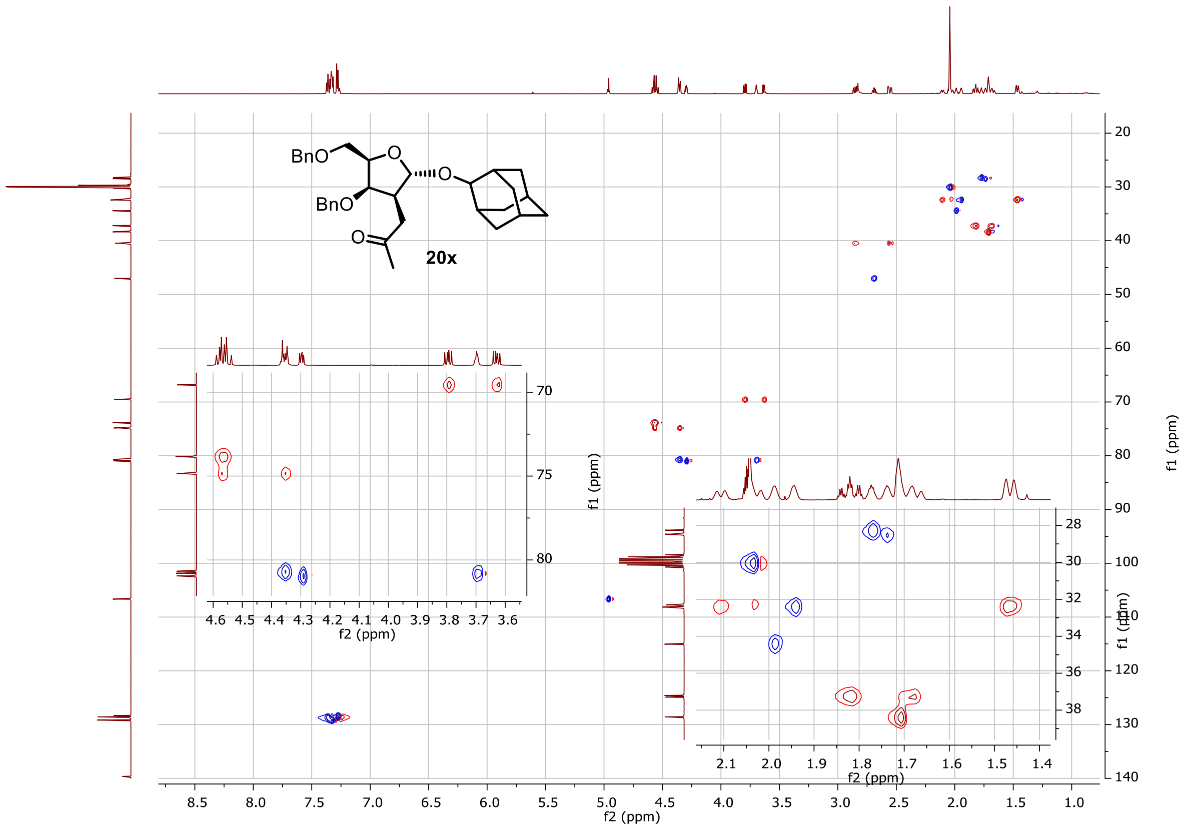

Supplementary Figure 207. HSQC spectra for **20x**

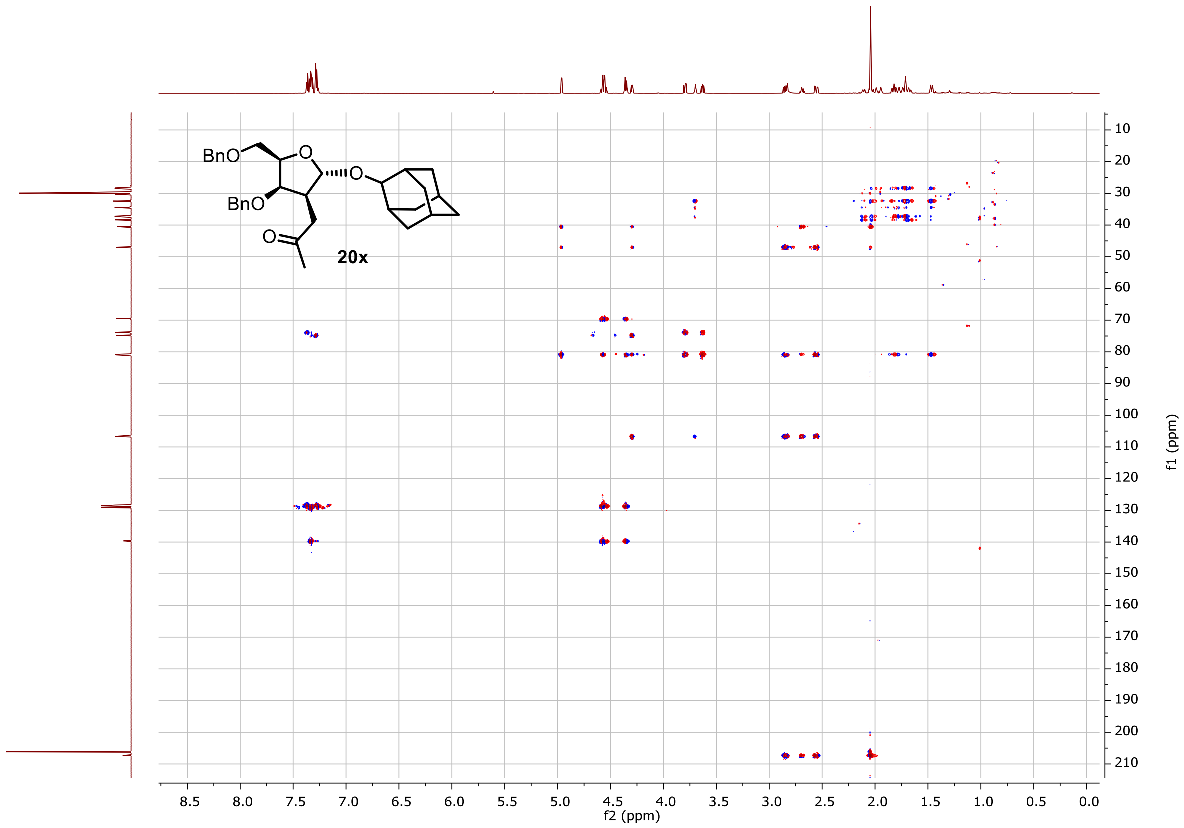

Supplementary Figure 208. HMBC spectra for **20x**

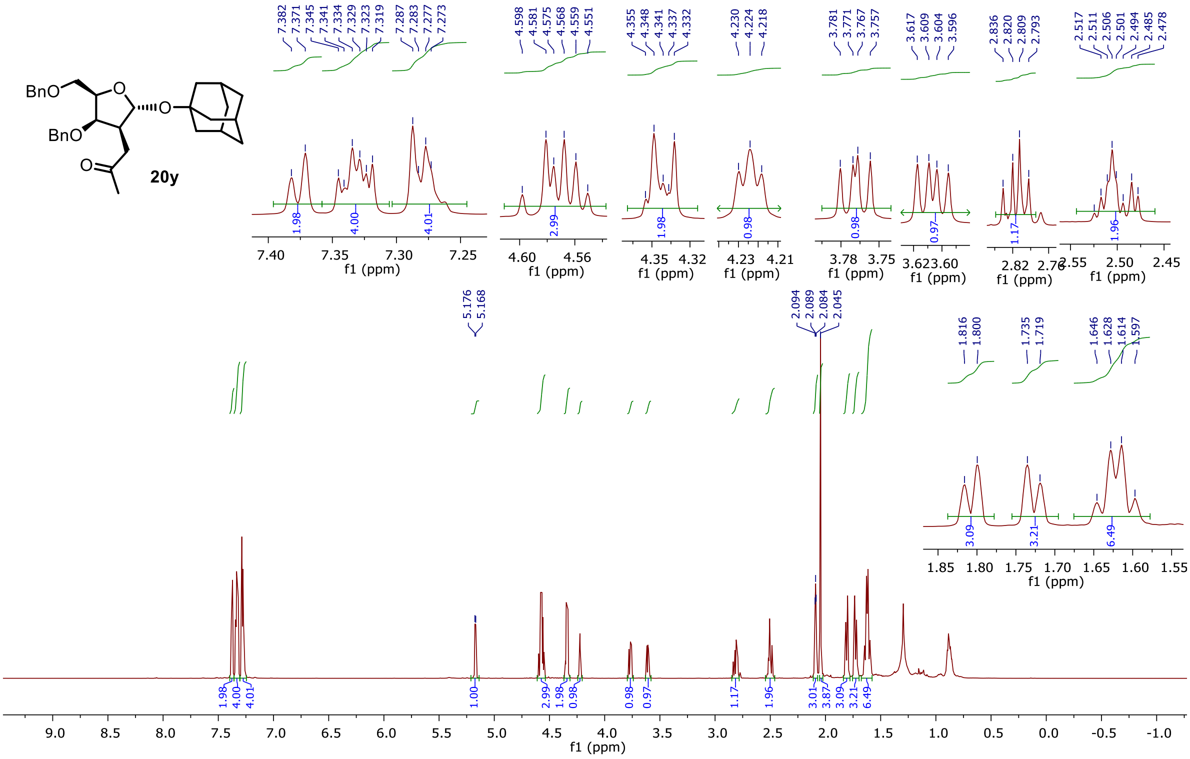

Supplementary Figure 209. ¹H spectra for **20y**

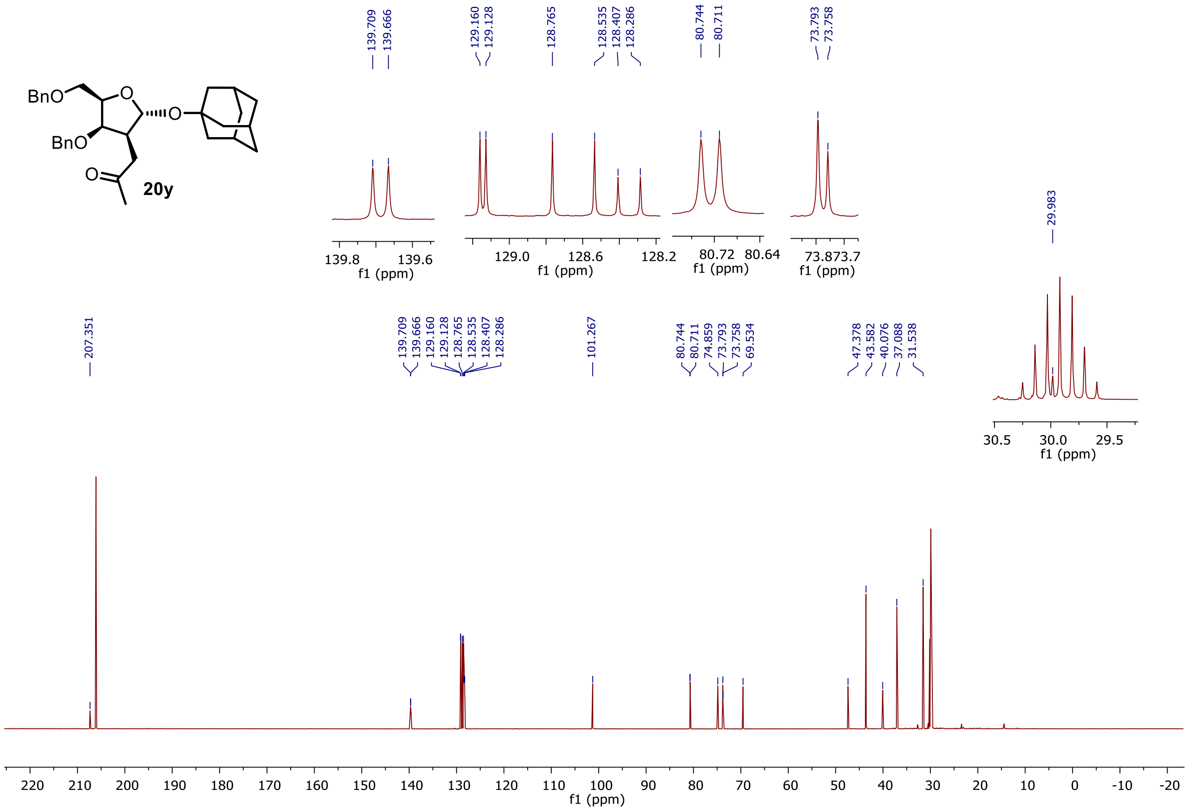

Supplementary Figure 210. ¹³C spectra for **20y**

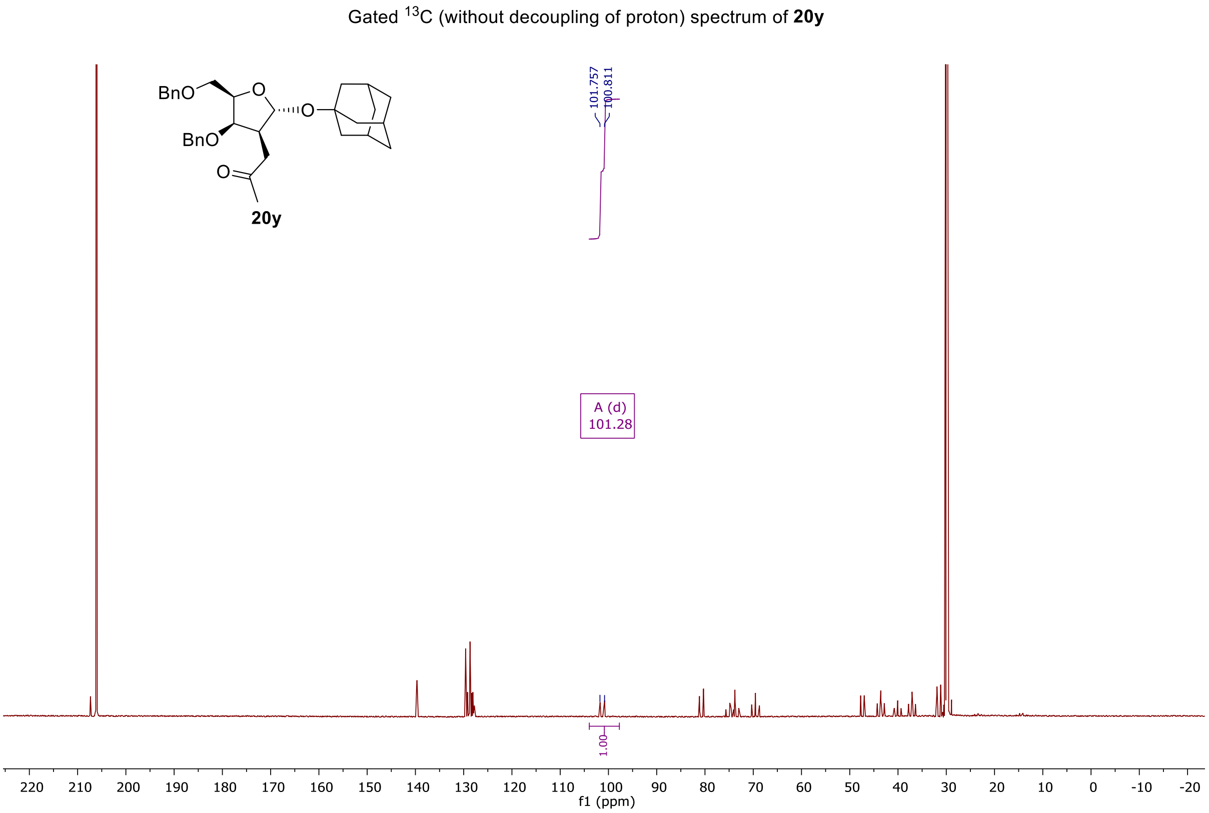

Supplementary Figure 211. Gated ^{13}C (with coupling of proton) spectra for **20y**

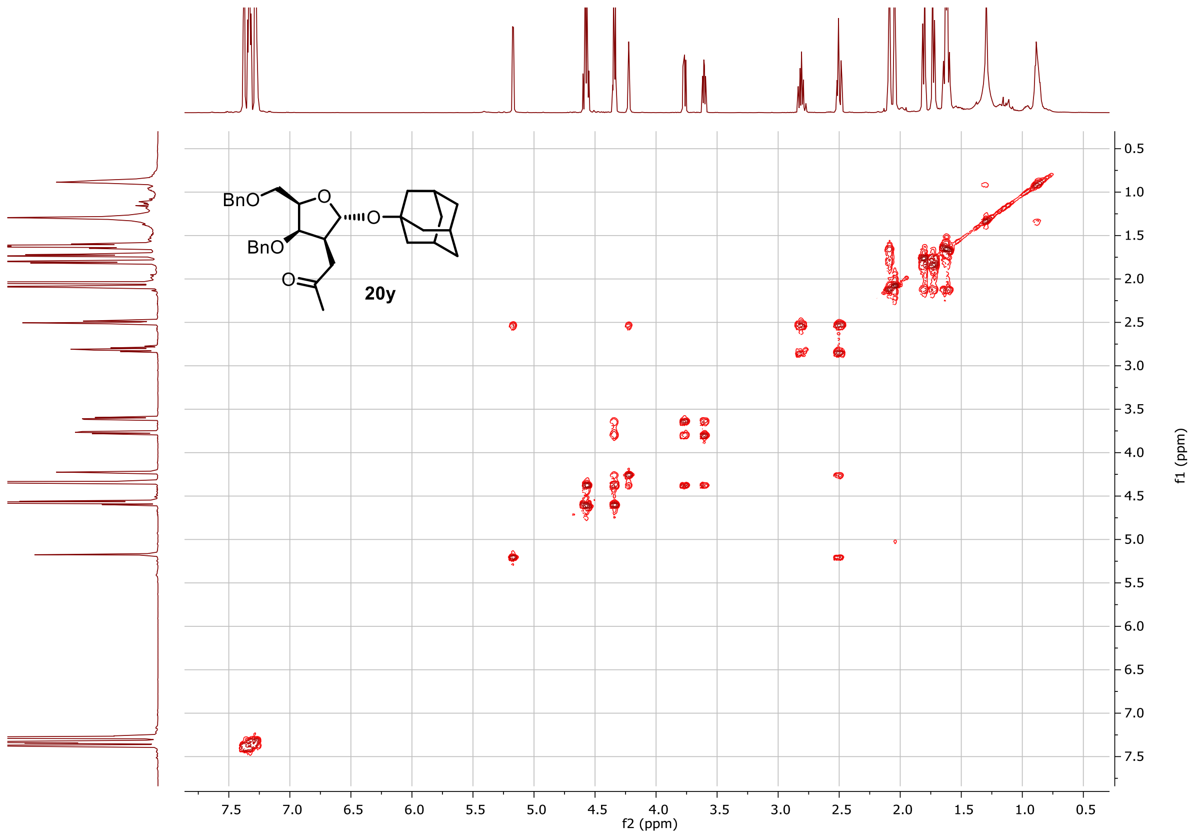

Supplementary Figure 212. COSY spectra for **20y**

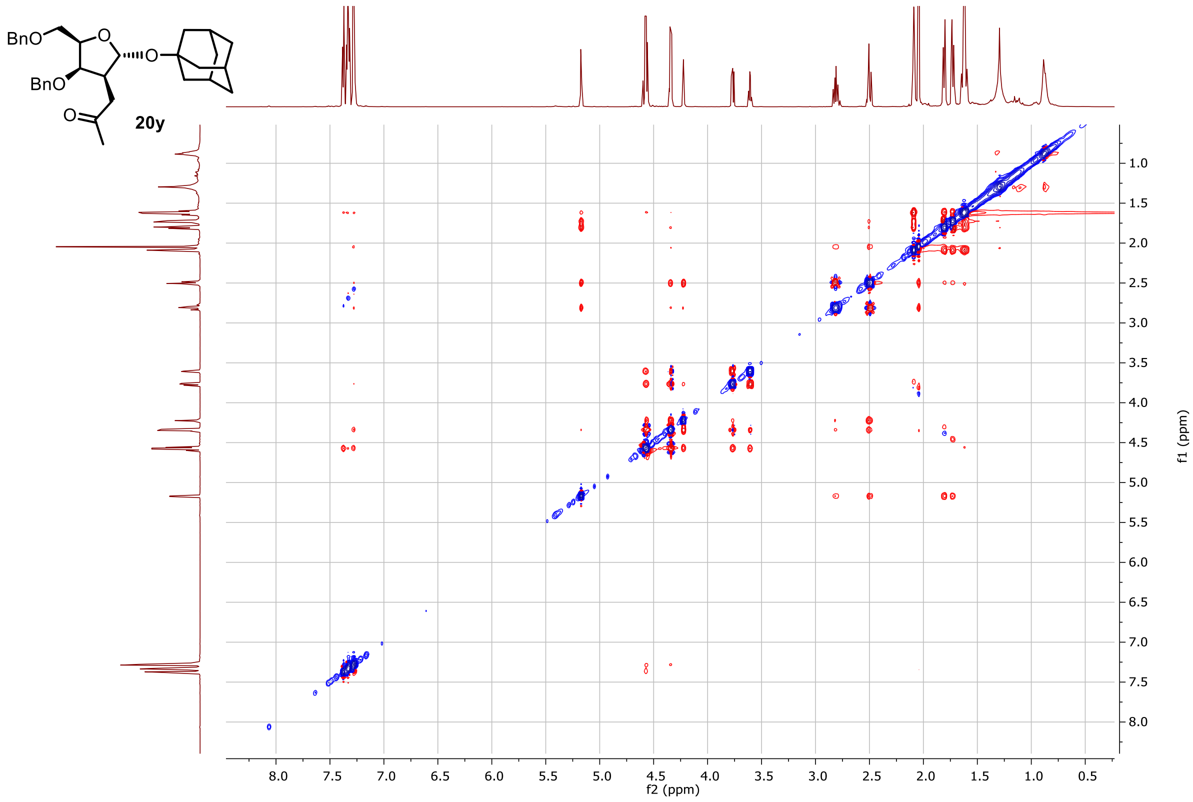

Supplementary Figure 213. NOESY spectra for **20y**

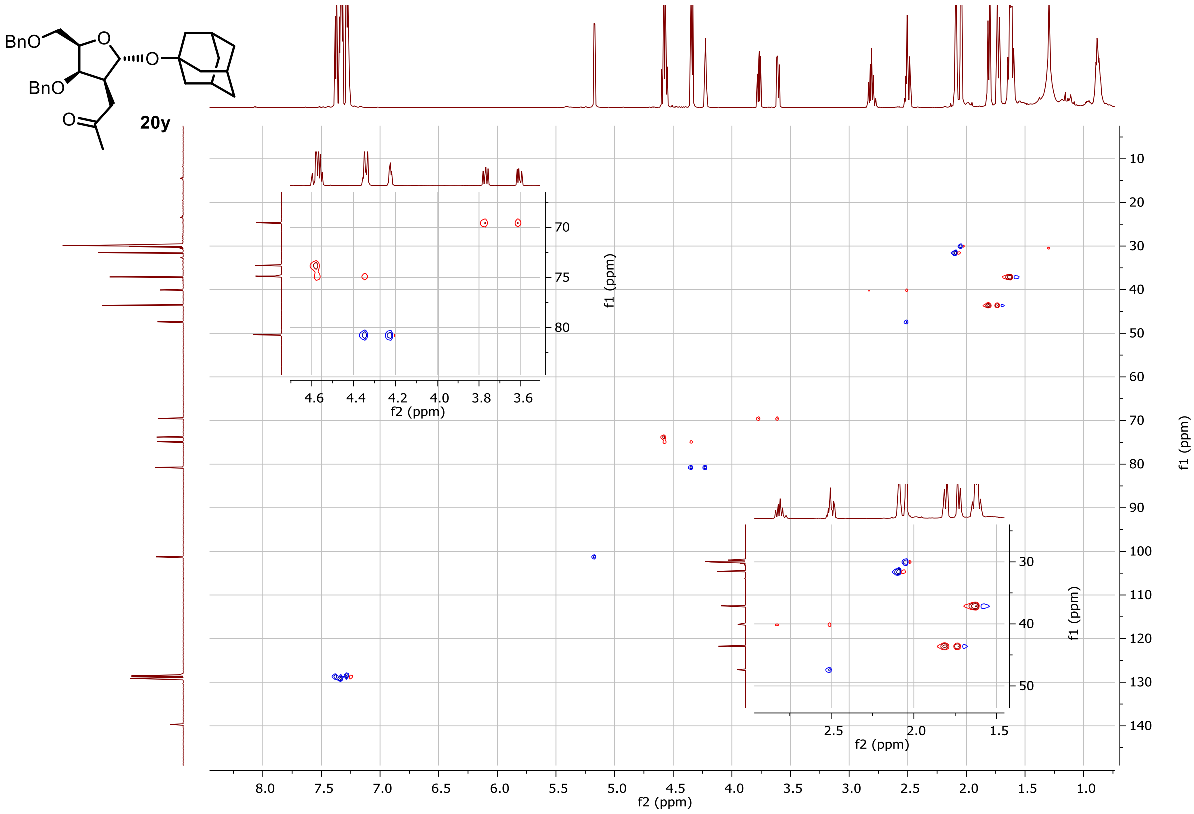

Supplementary Figure 214. HSQC spectra for **20y**

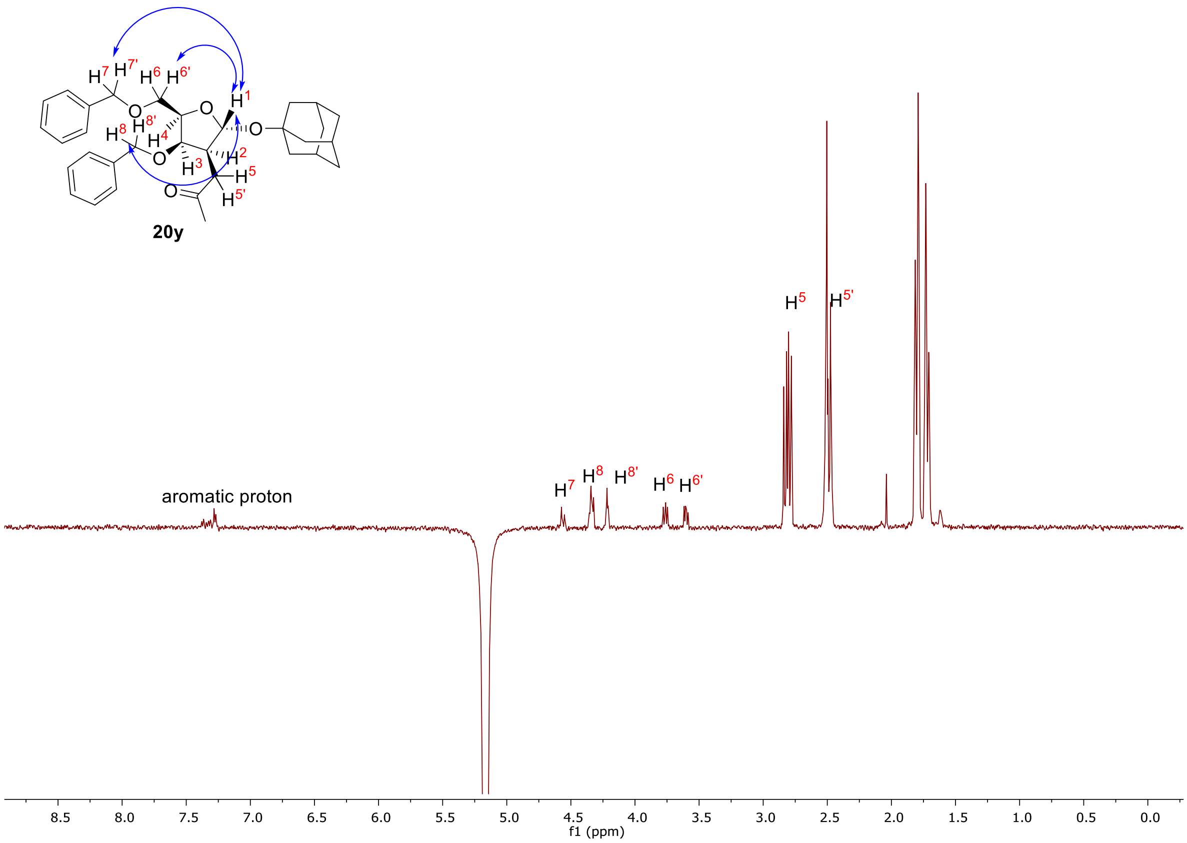

Supplementary Figure 215. 1D NOE spectra for **20y**

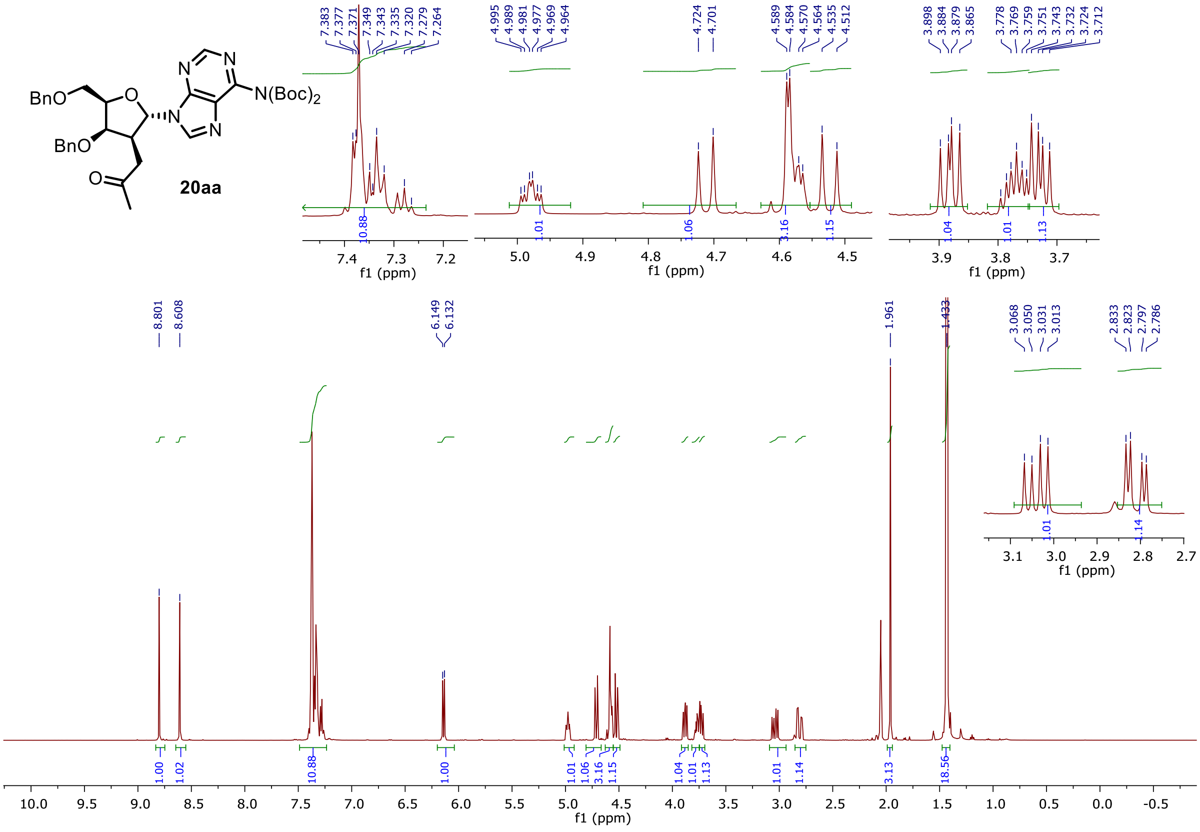

Supplementary Figure 216. ^{1}H spectra for **20aa**

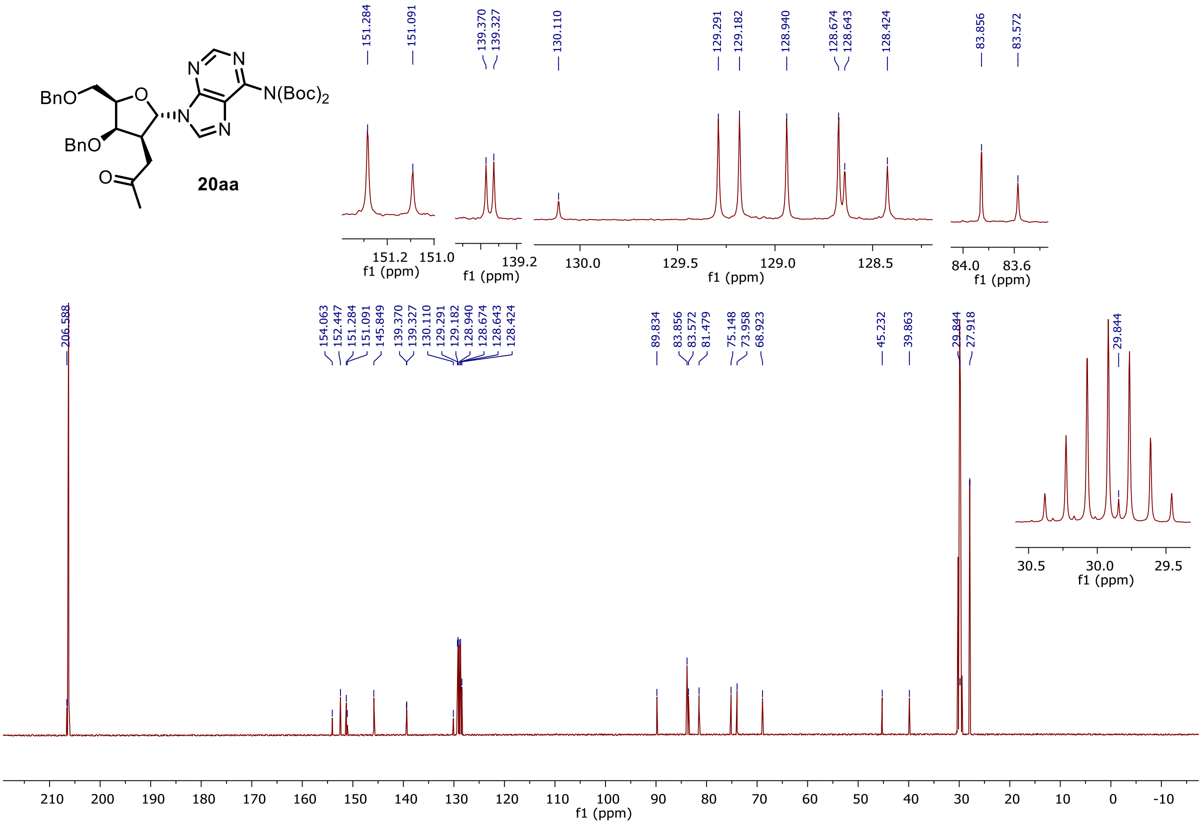

Supplementary Figure 217. ^{13}C spectra for **20aa**

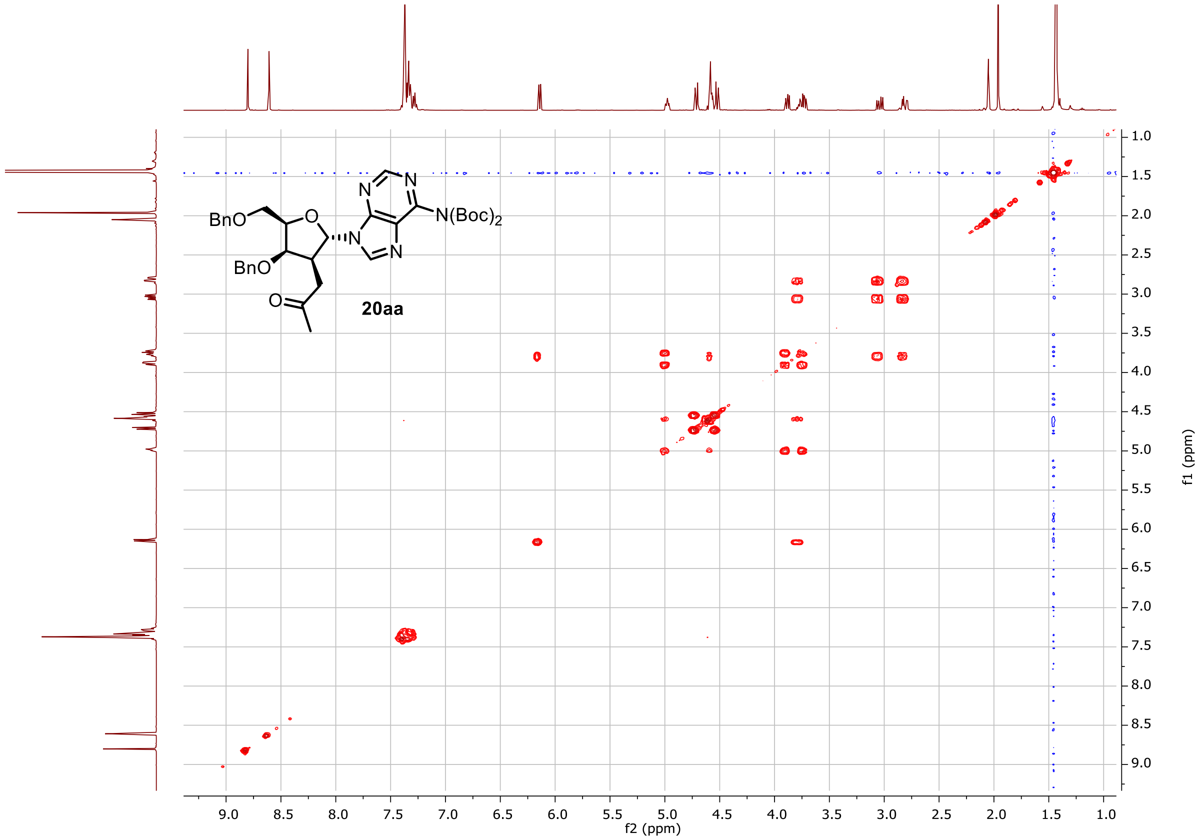

Supplementary Figure 218. COSY spectra for **20aa**

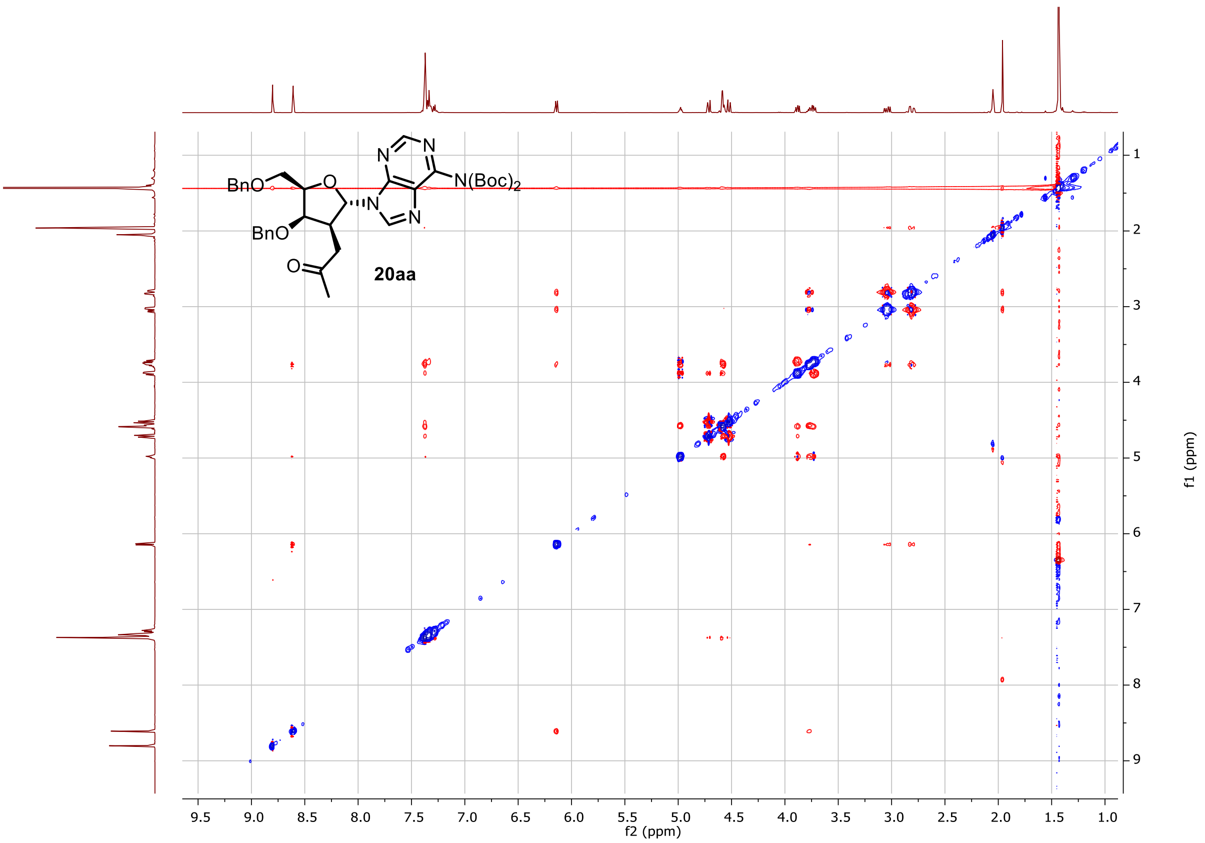

Supplementary Figure 219. NOESY spectra for **20aa**

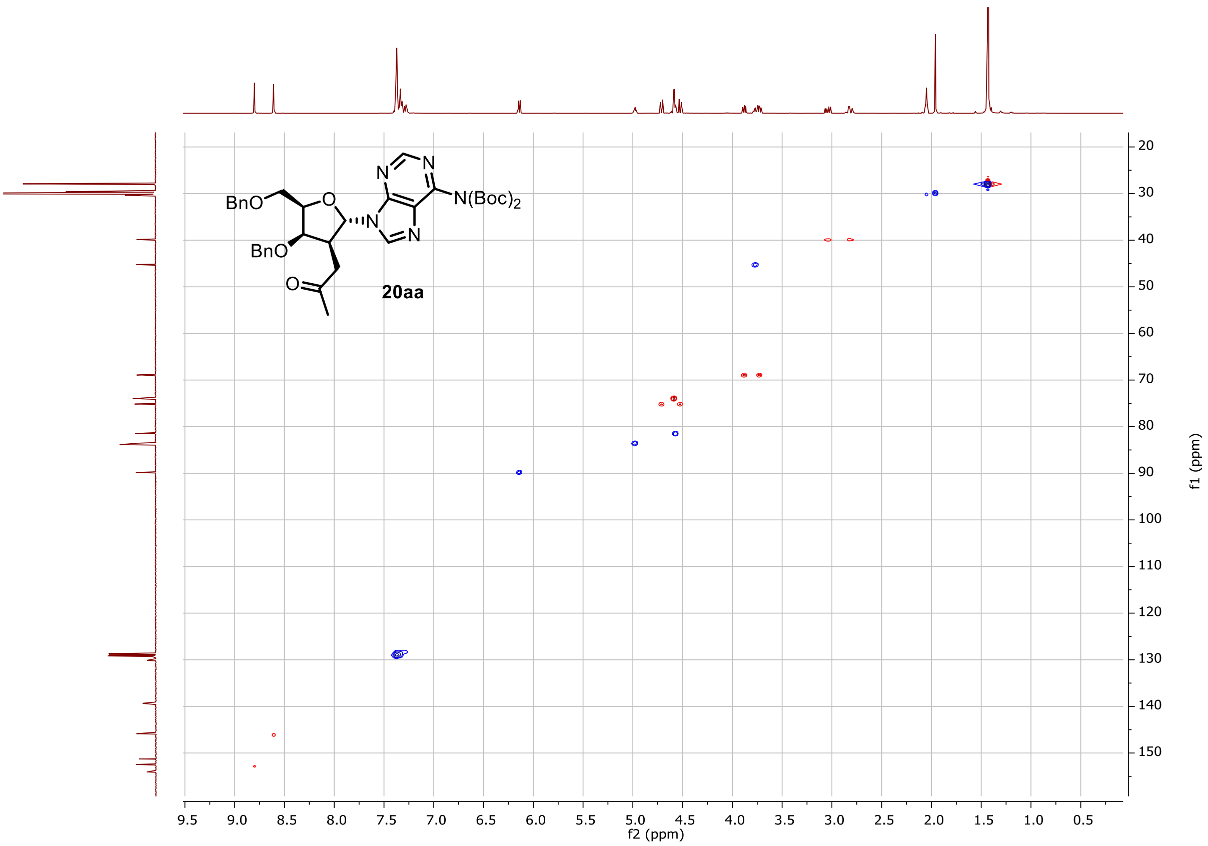

Supplementary Figure 220. HSQC spectra for **20aa**

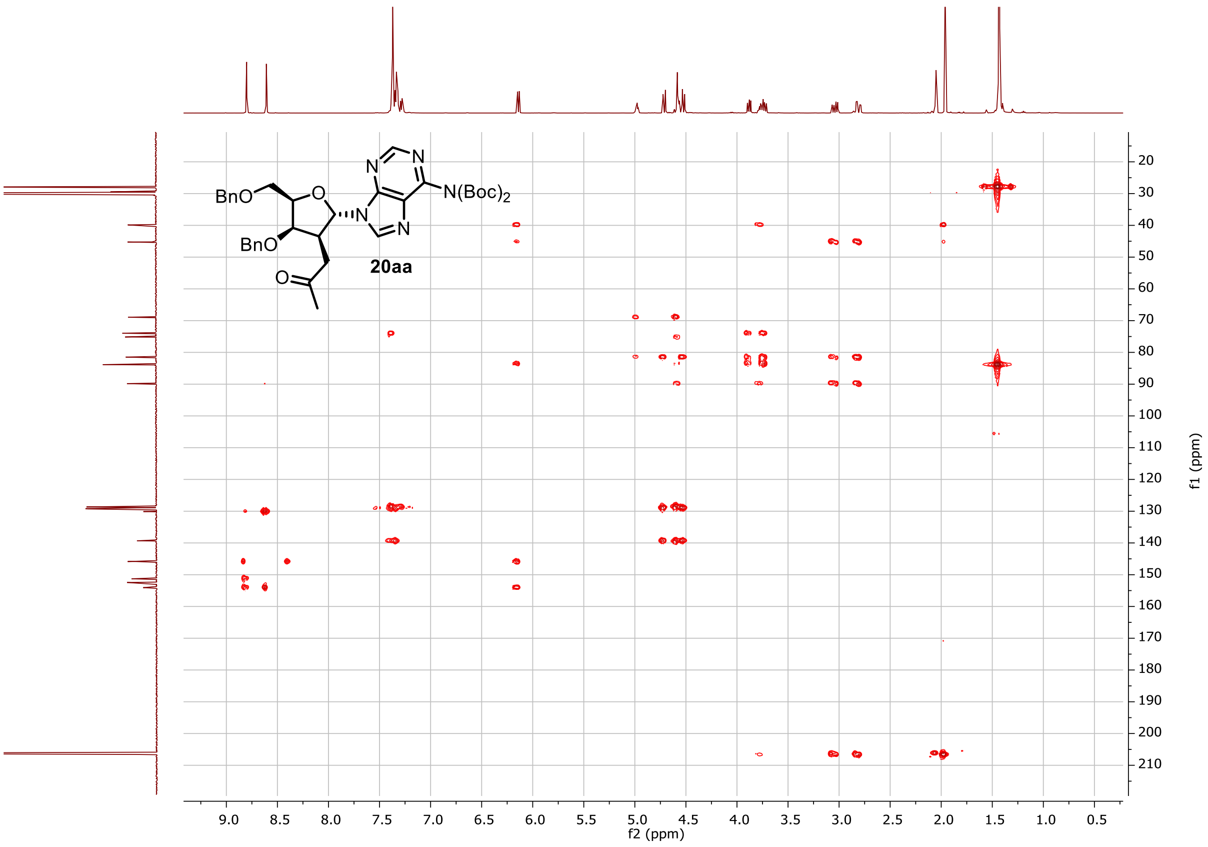

Supplementary Figure 221. HMBC spectra for **20aa**

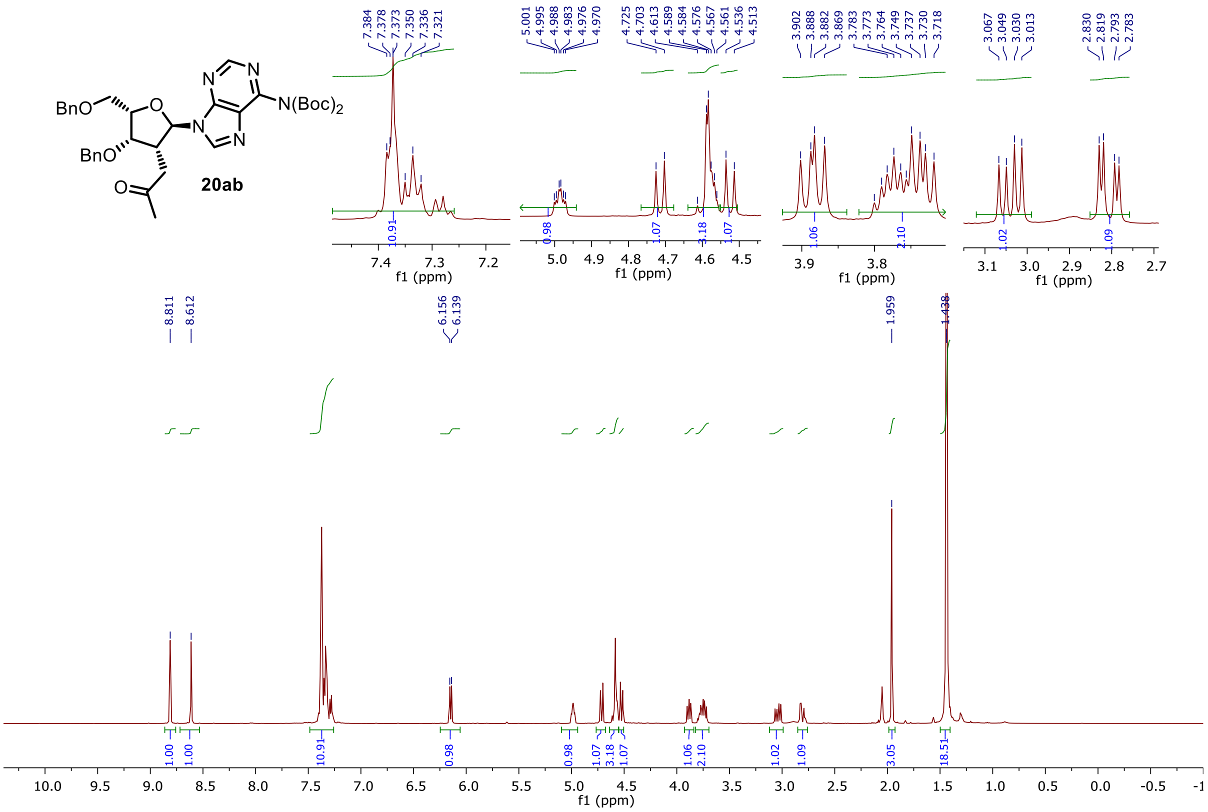

Supplementary Figure 222. ^{1}H spectra for **20ab**

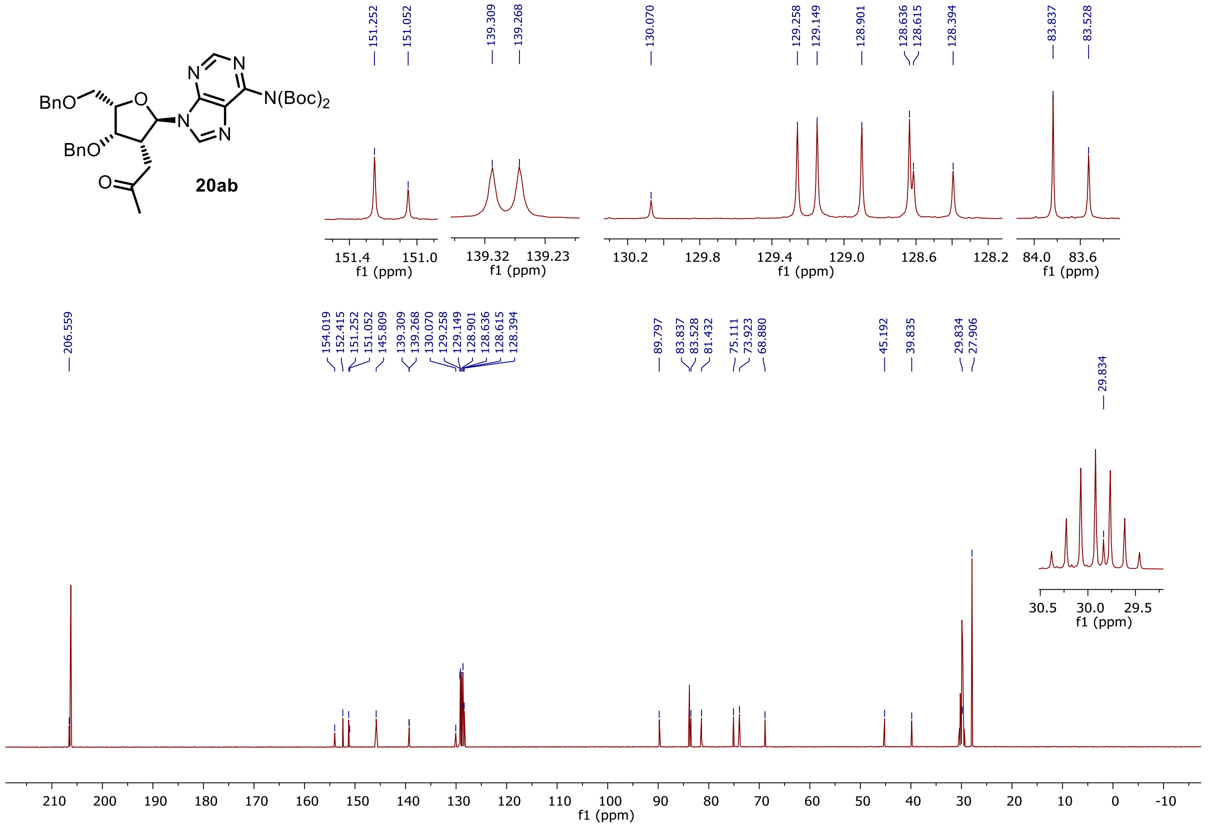

Supplementary Figure 223. ^{13}C spectra for **20ab**

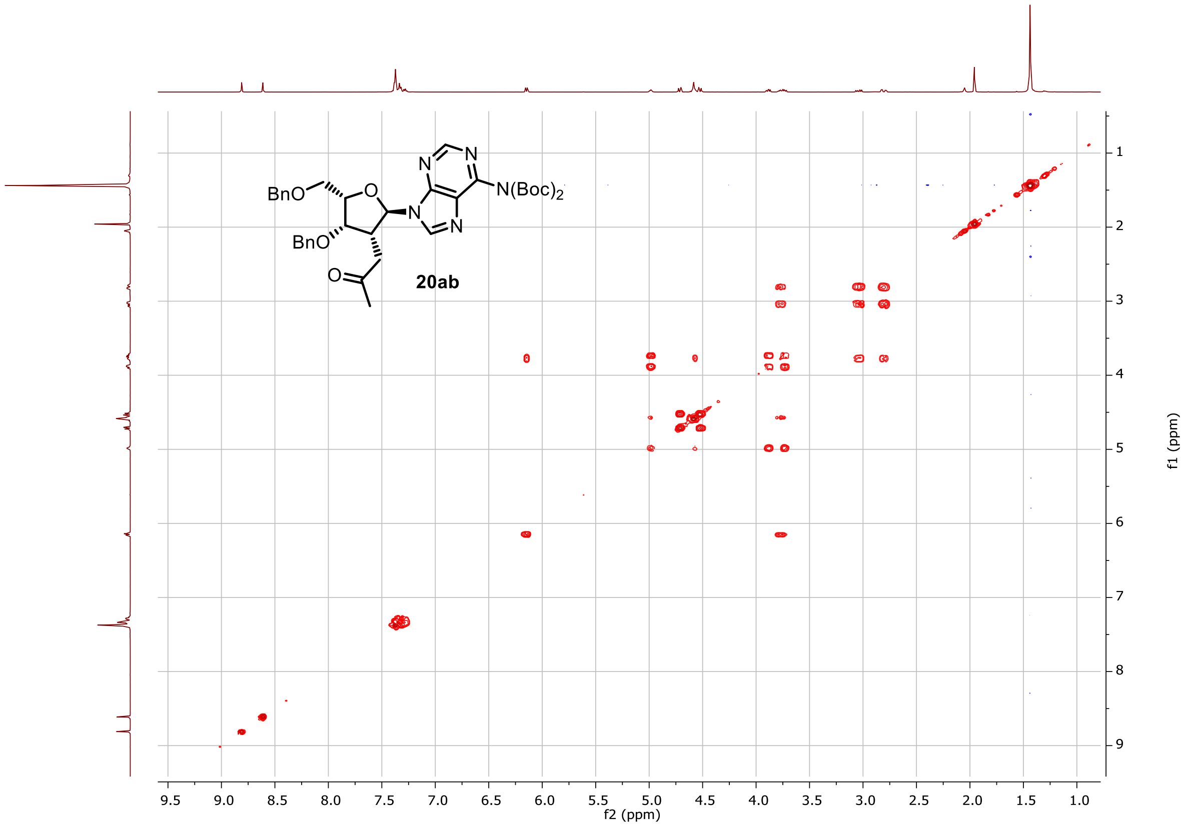

Supplementary Figure 224. COSY spectra for **20ab**

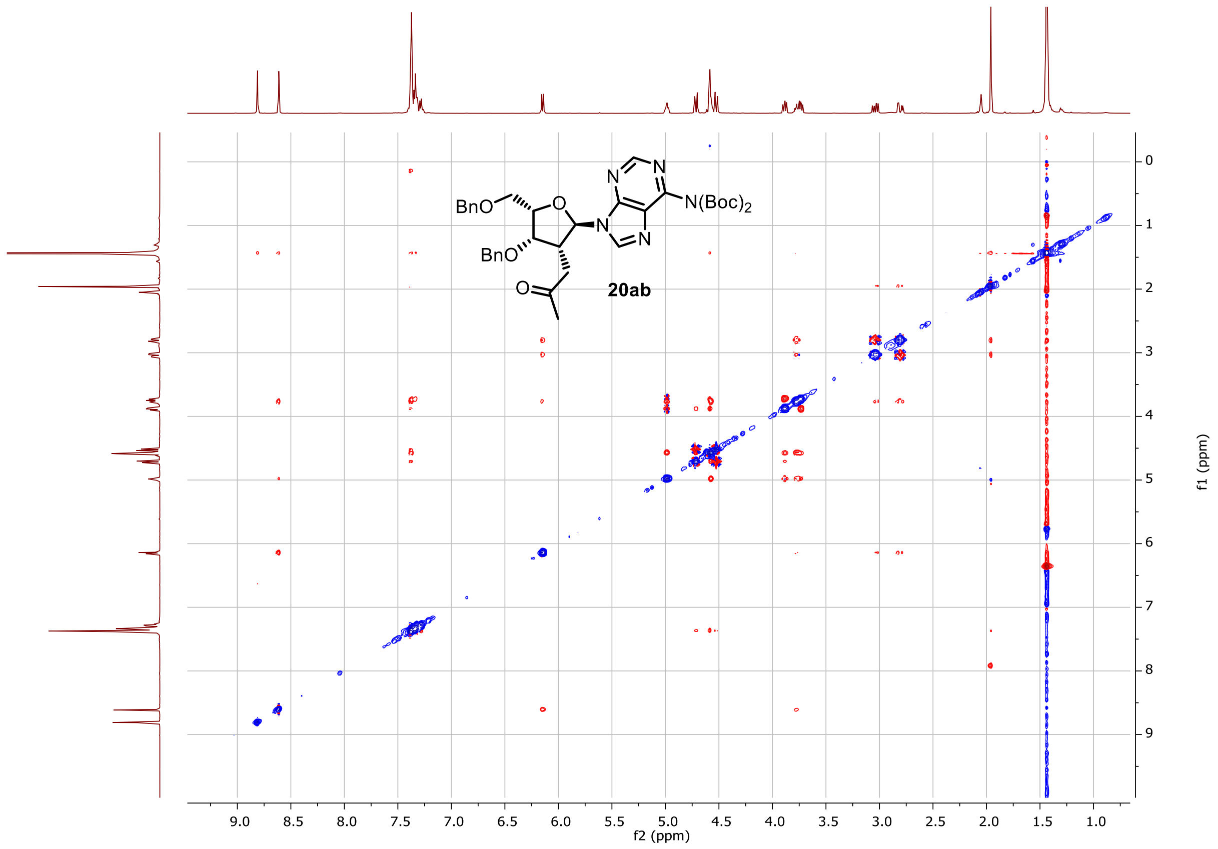

Supplementary Figure 225. NOESY spectra for **20ab**

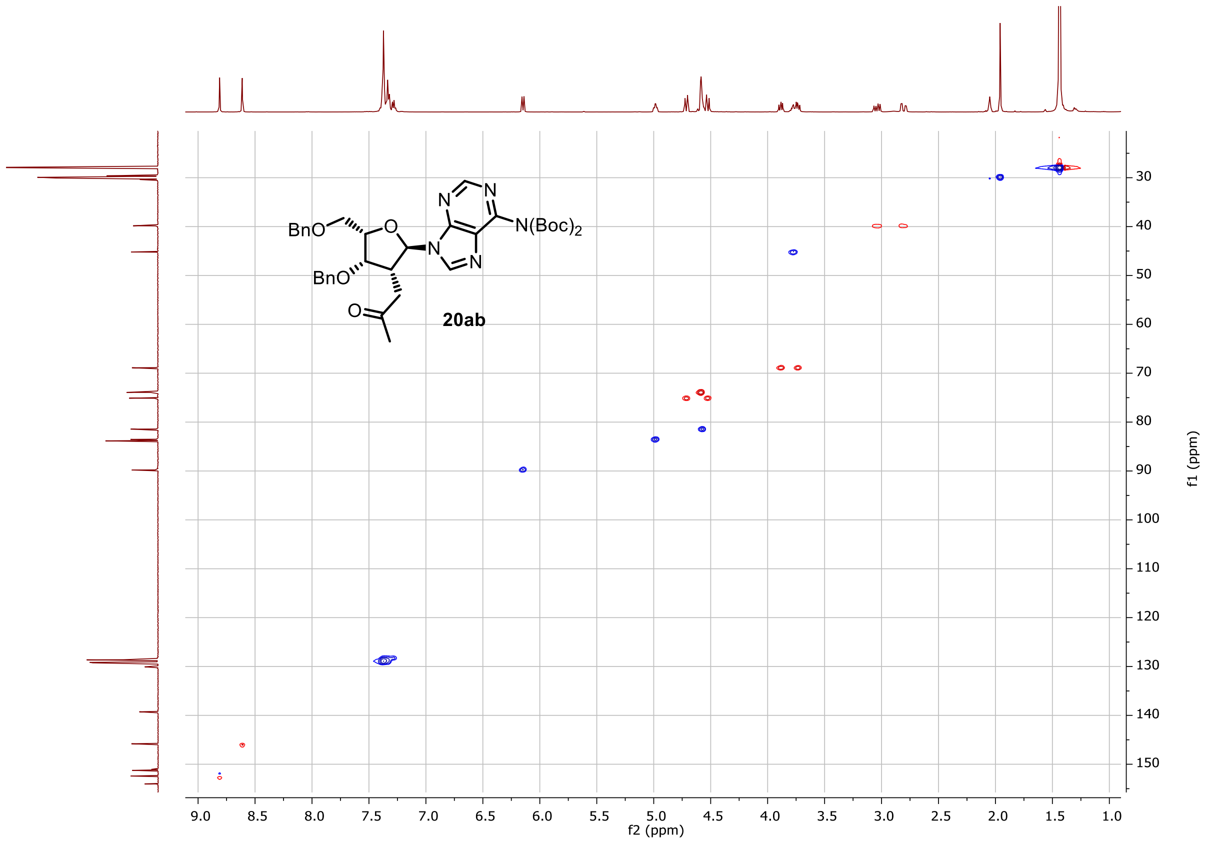

Supplementary Figure 226. HSQC spectra for **20ab**

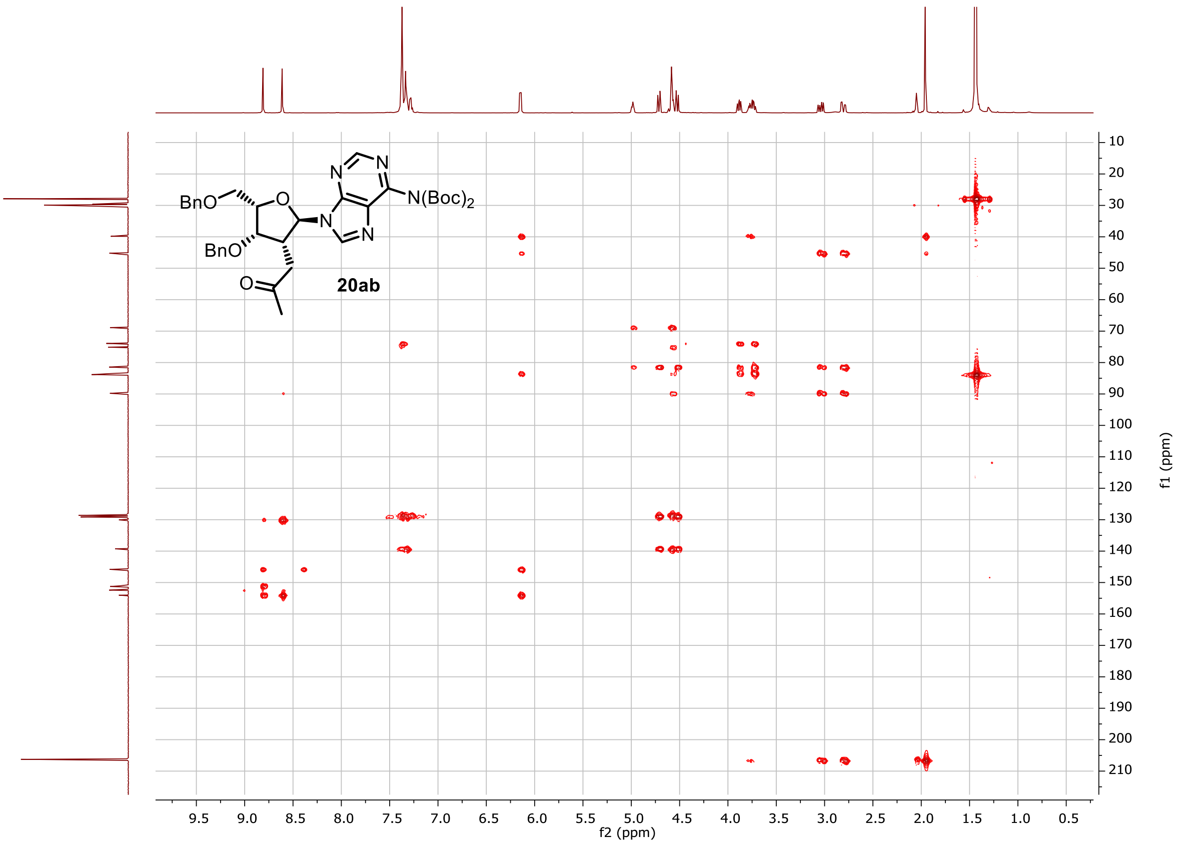

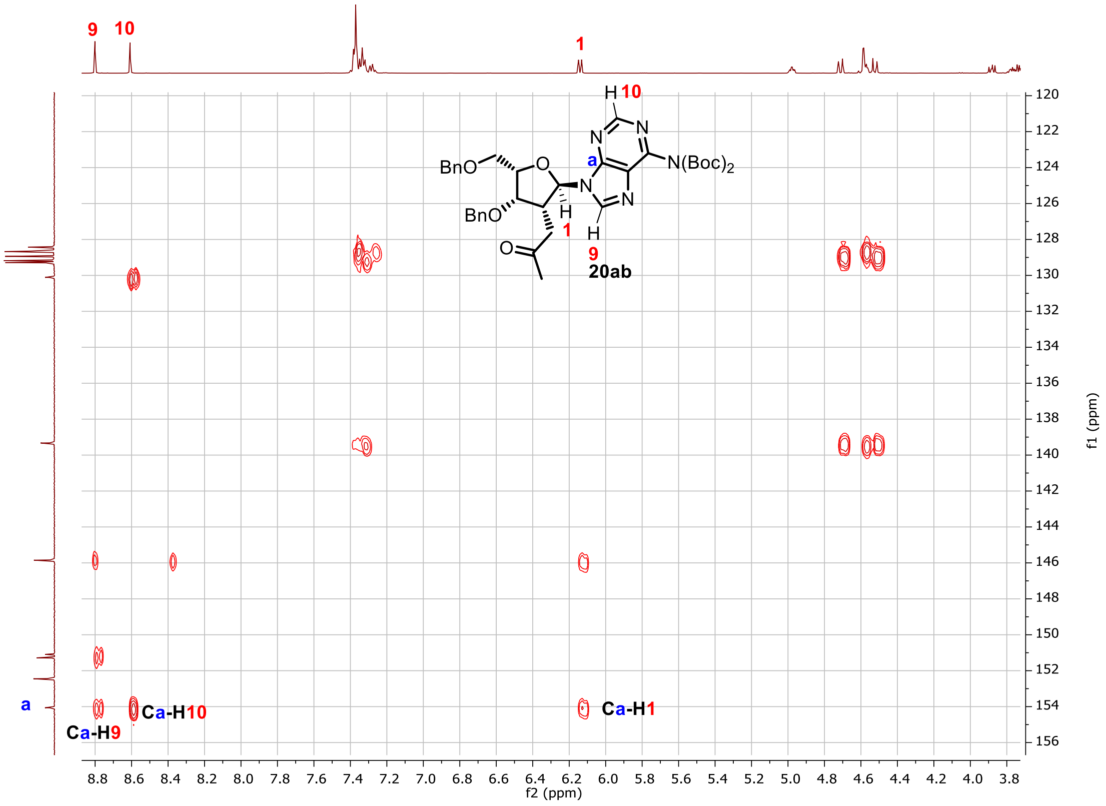

Supplementary Figure 227. HMBC spectra for **20ab**

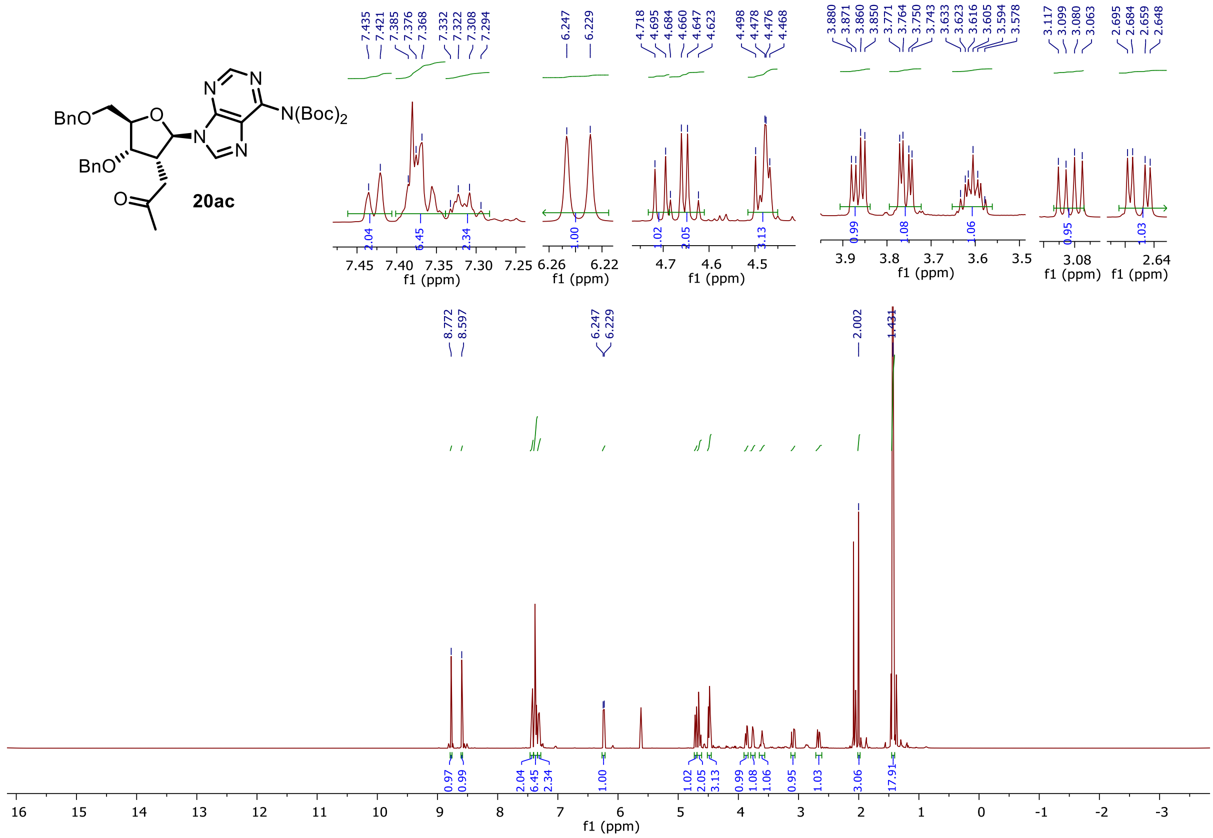

Supplementary Figure 228. ^{1}H spectra for **20ac**

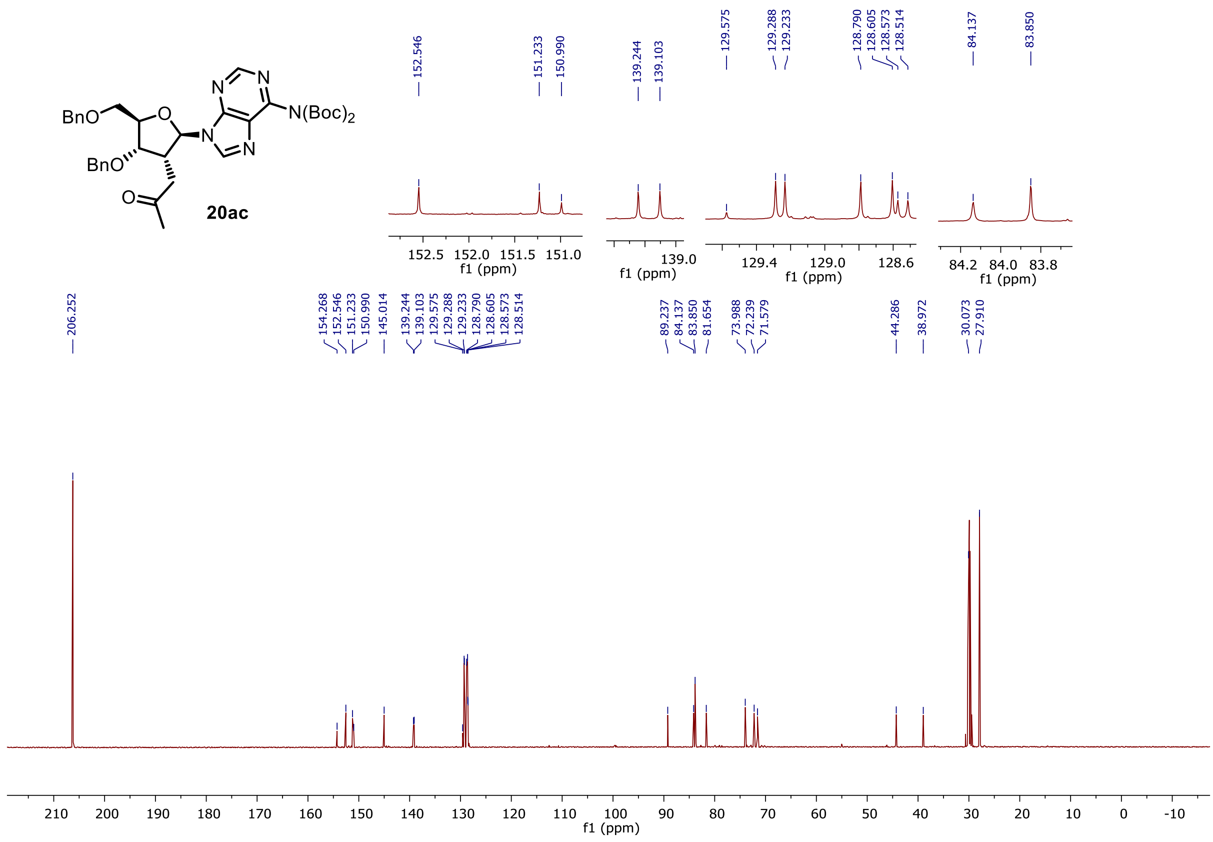

Supplementary Figure 229. ^{13}C spectra for **20ac**

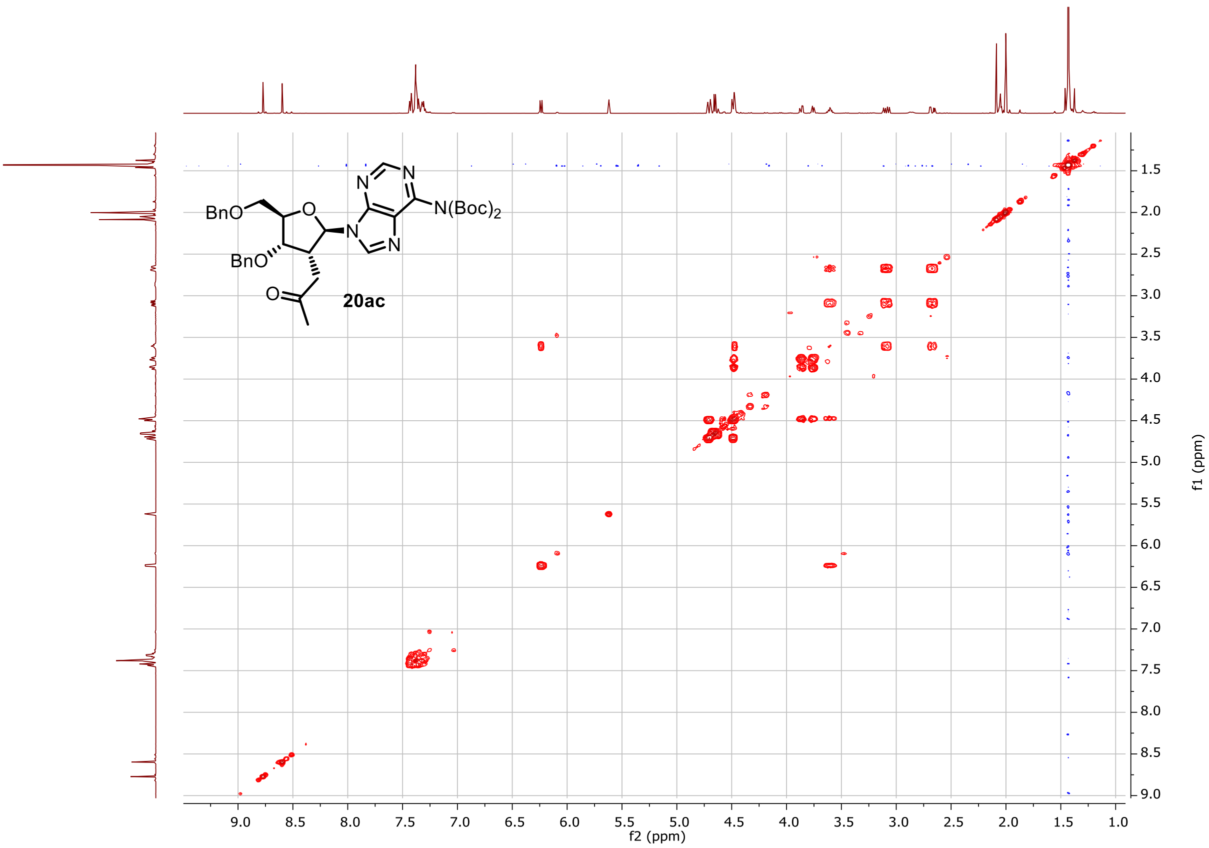

Supplementary Figure 230. COSY spectra for **20ac**

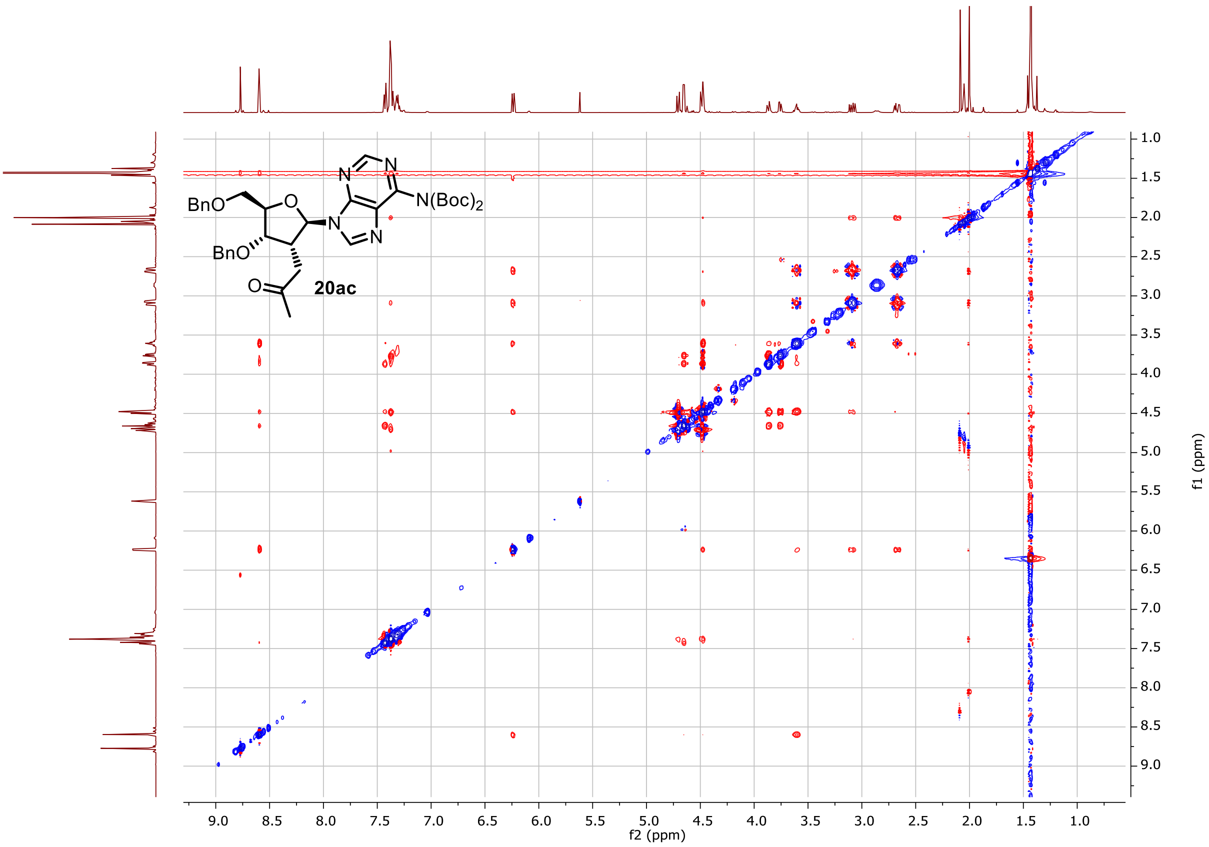

Supplementary Figure 231. NOESY spectra for **20ac**

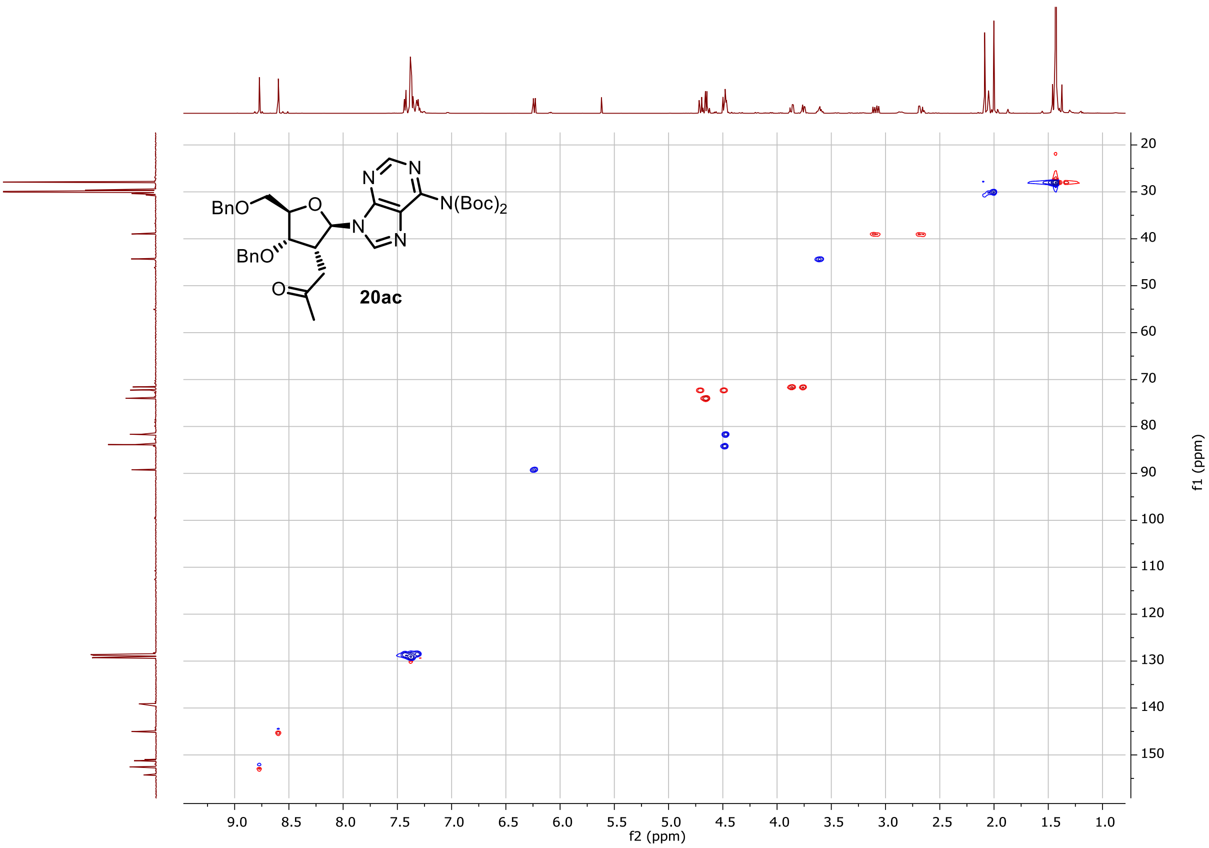

Supplementary Figure 232. HSQC spectra for **20ac**

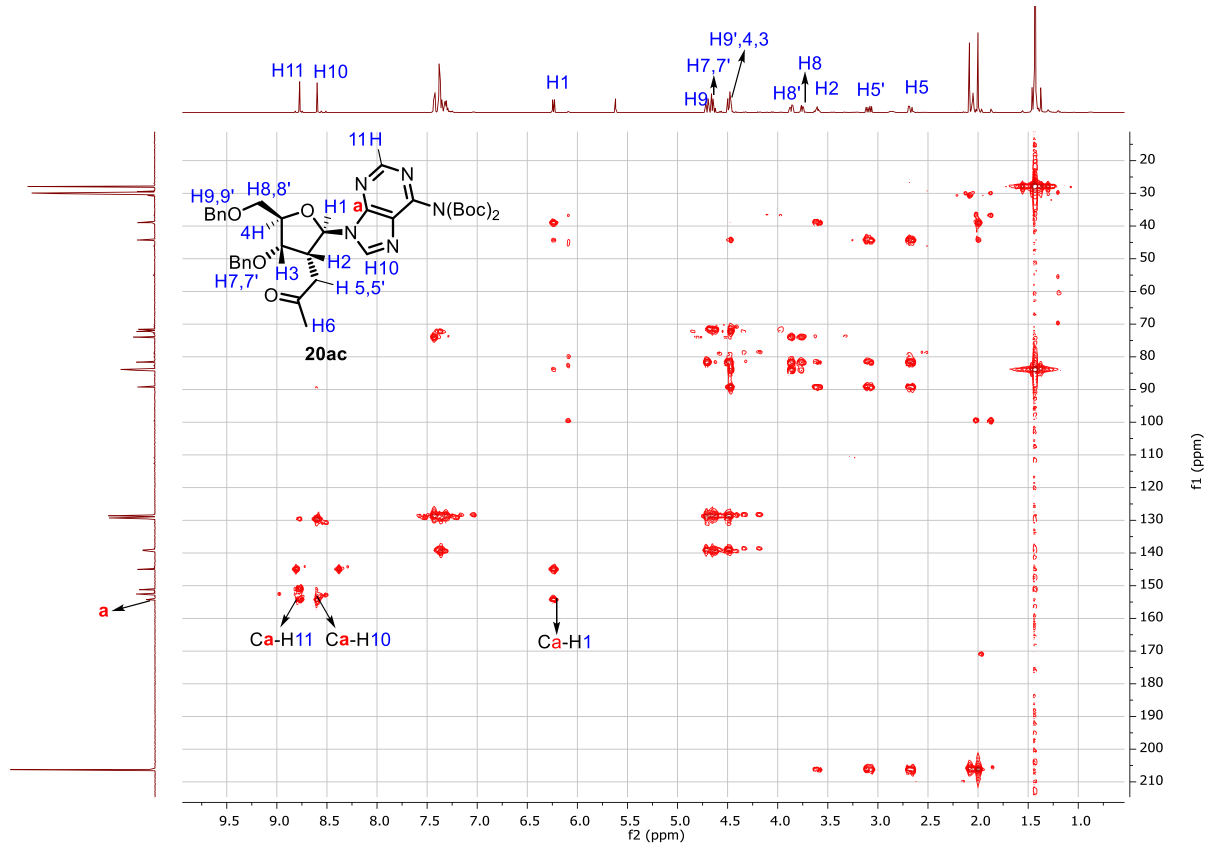

Supplementary Figure 233. HMBC spectra for **20ac**

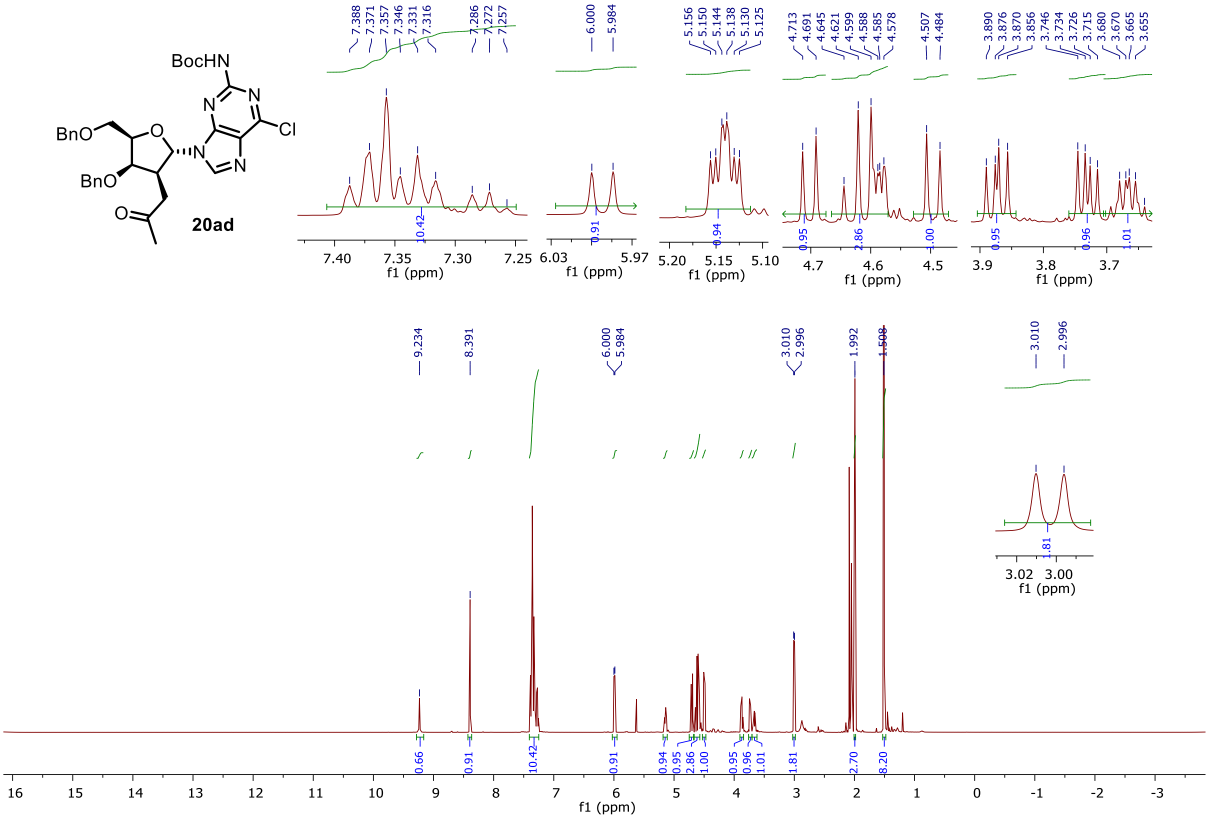

Supplementary Figure 234. ^{1}H spectra for **20ad**

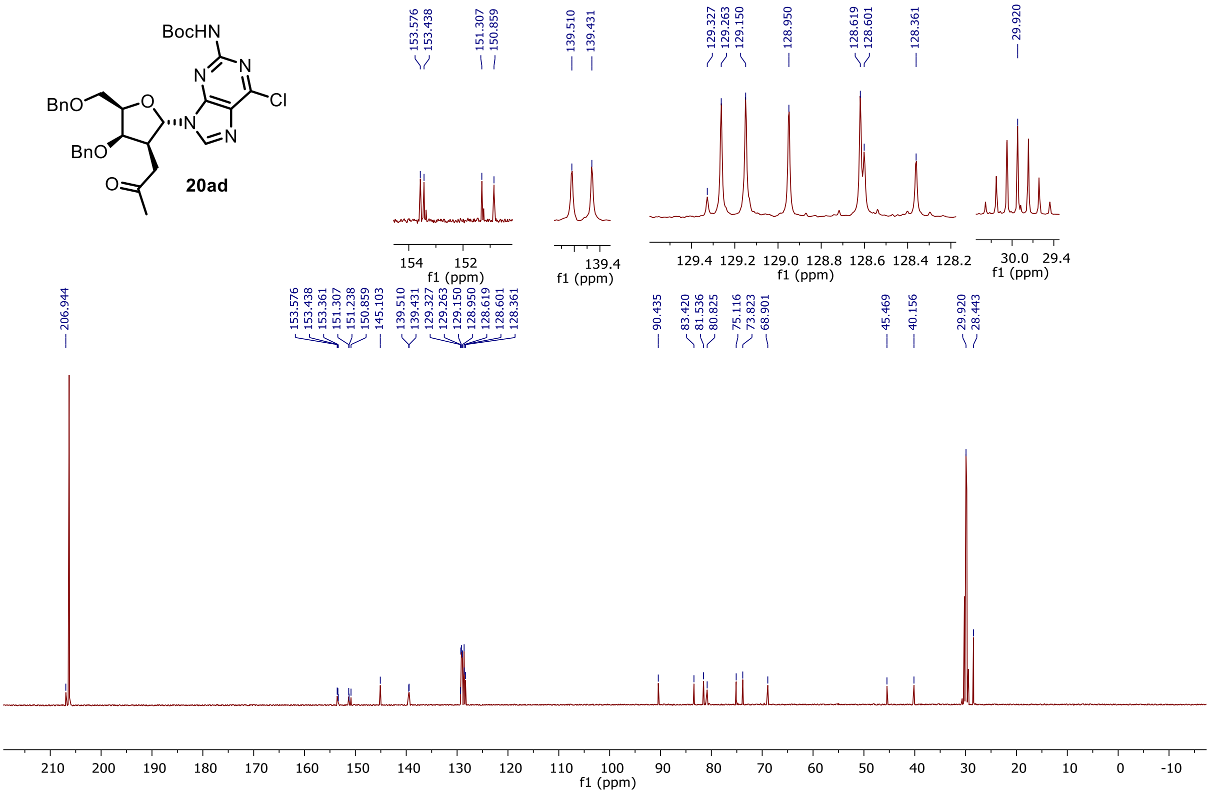

Supplementary Figure 235. ^{13}C spectra for **20ad**

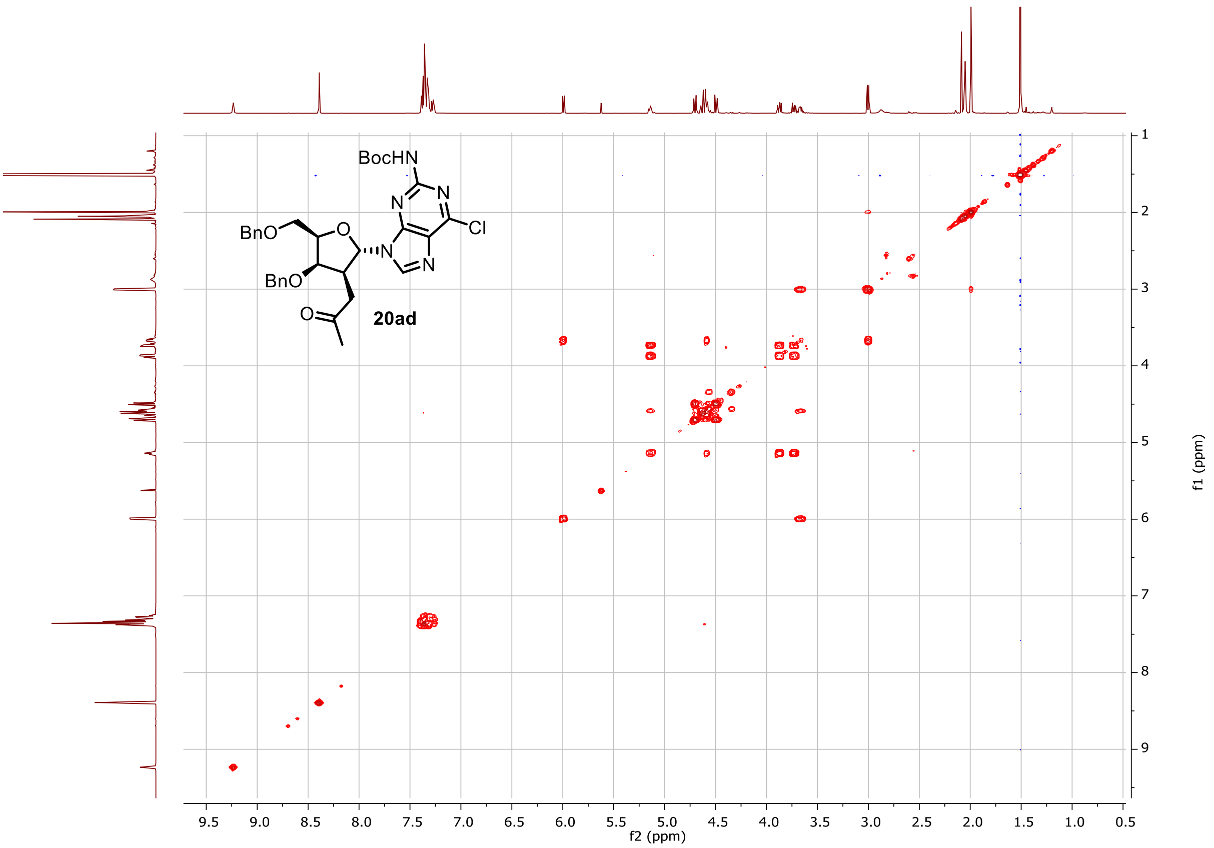

Supplementary Figure 236. COSY spectra for **20ad**

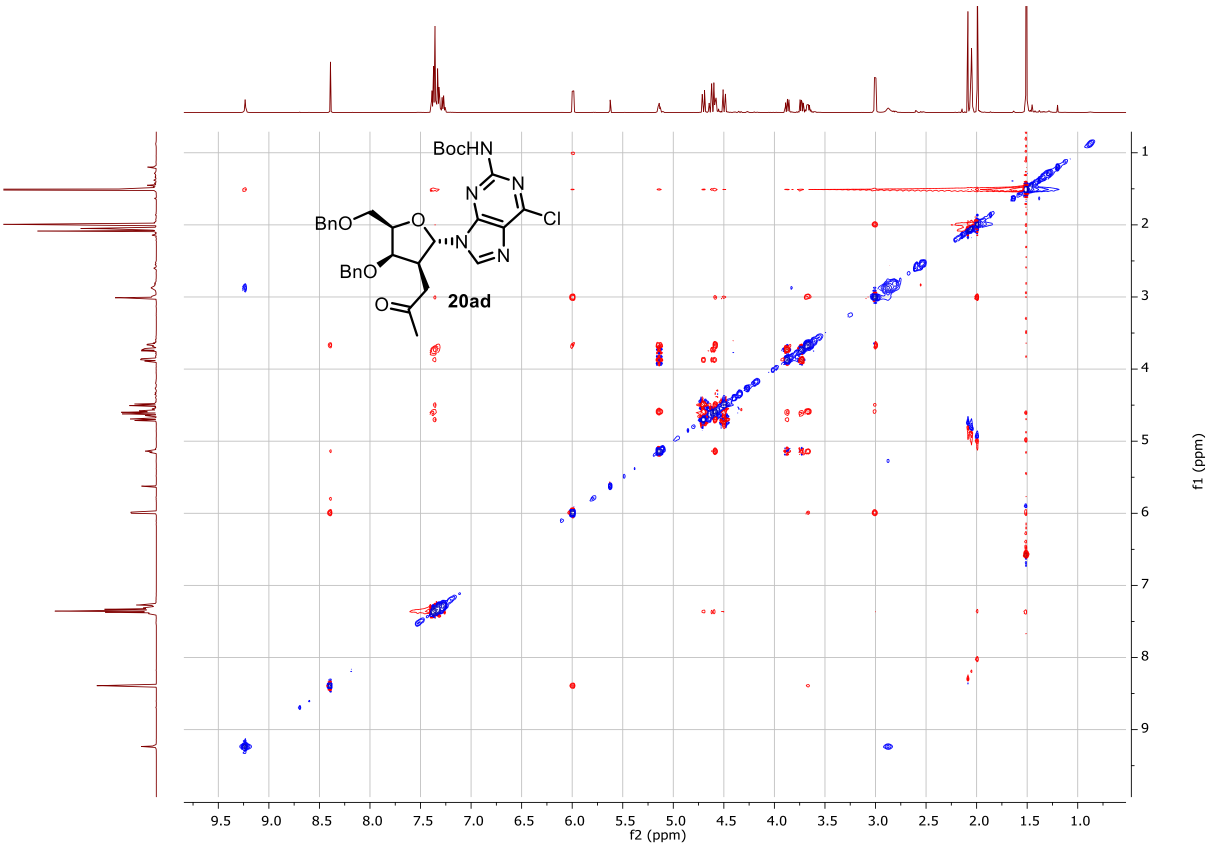

Supplementary Figure 237. NOESY spectra for **20ad**

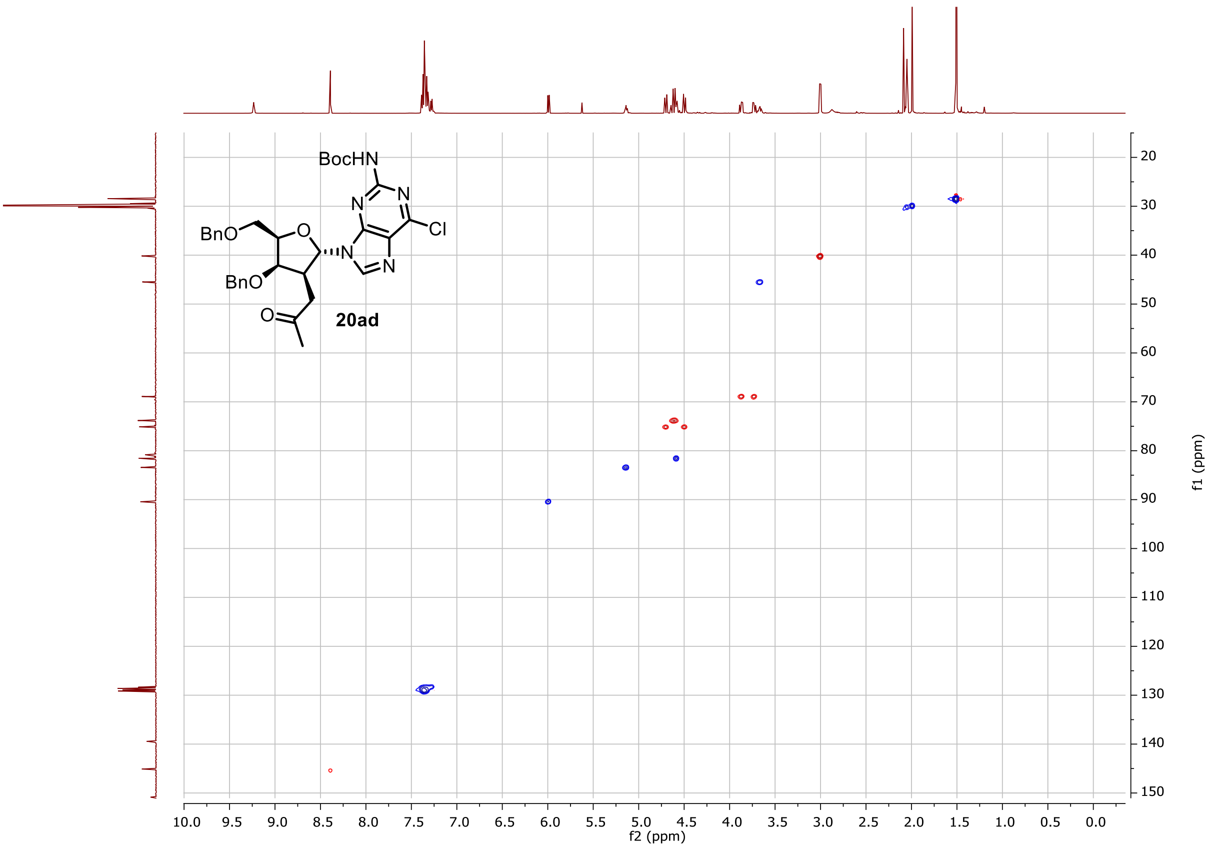

Supplementary Figure 238. HSQC spectra for **20ad**

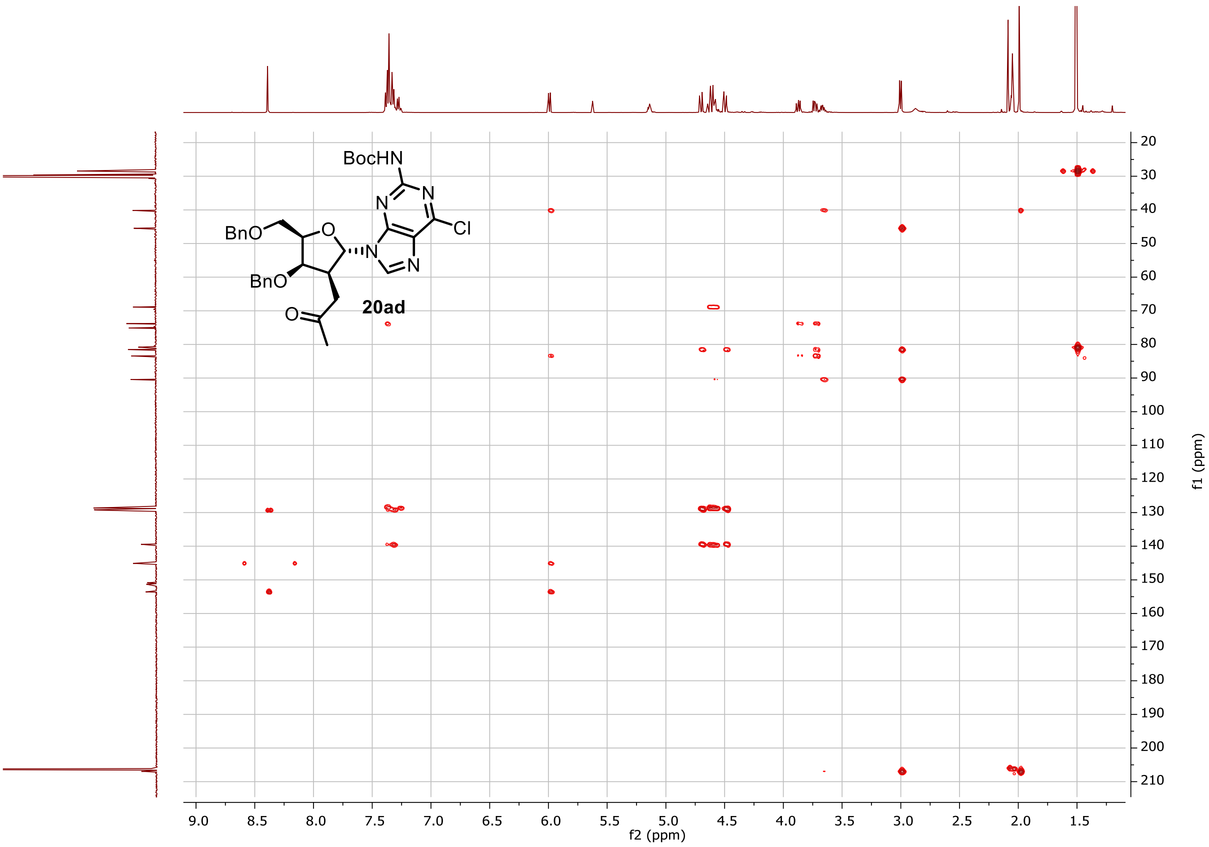

Supplementary Figure 239. HMBC spectra for **20ad**

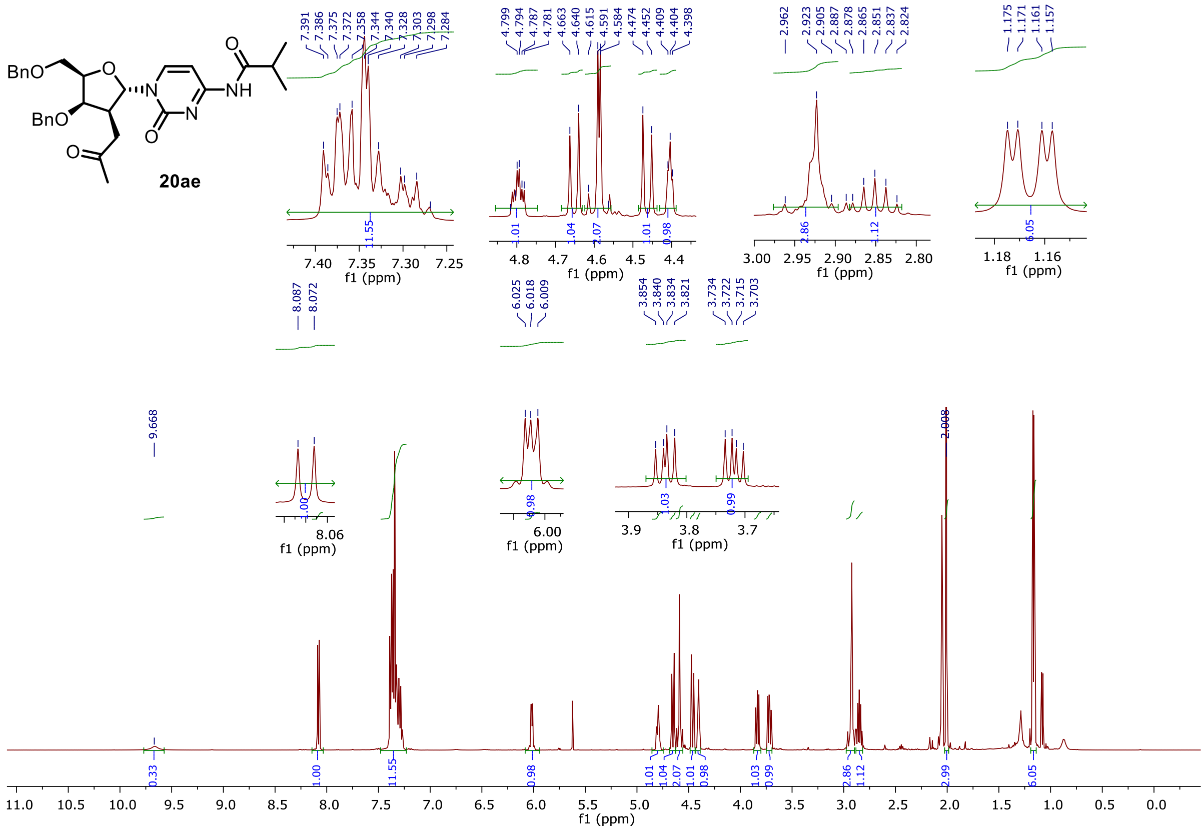

Supplementary Figure 240. ^{1}H spectra for **20ae**

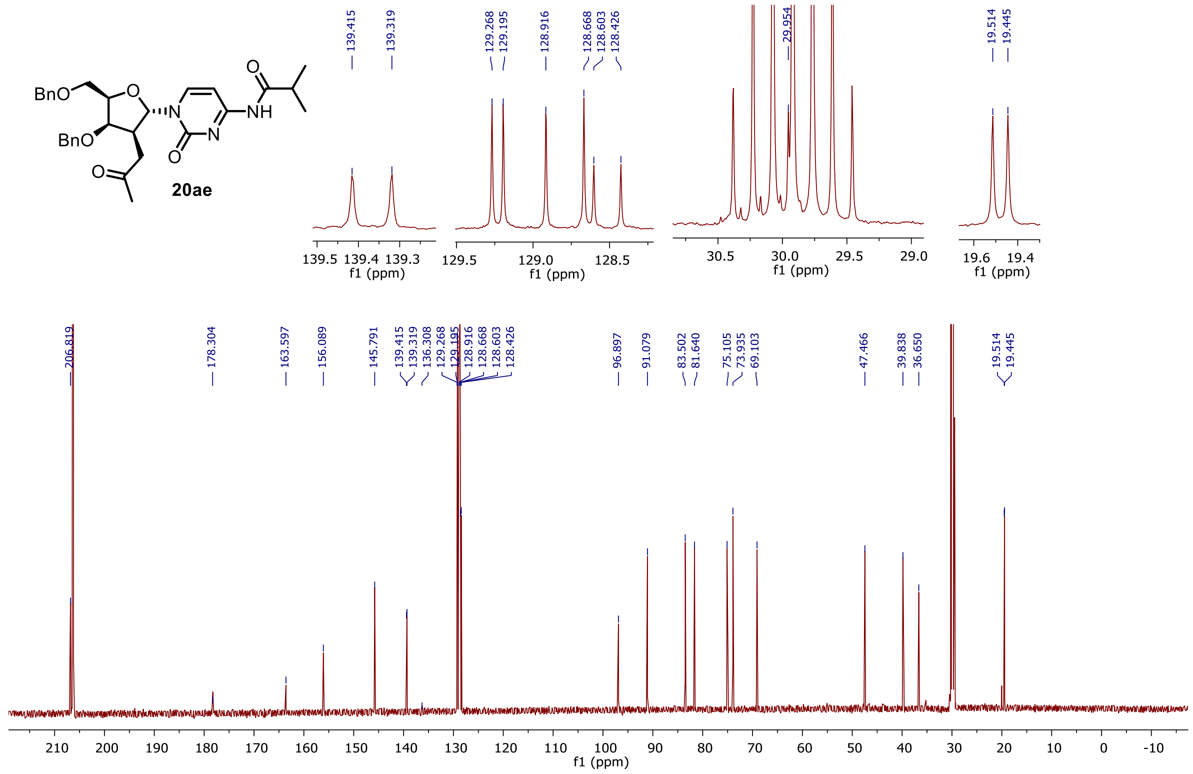

Supplementary Figure 241. ^{13}C spectra for **20ae**

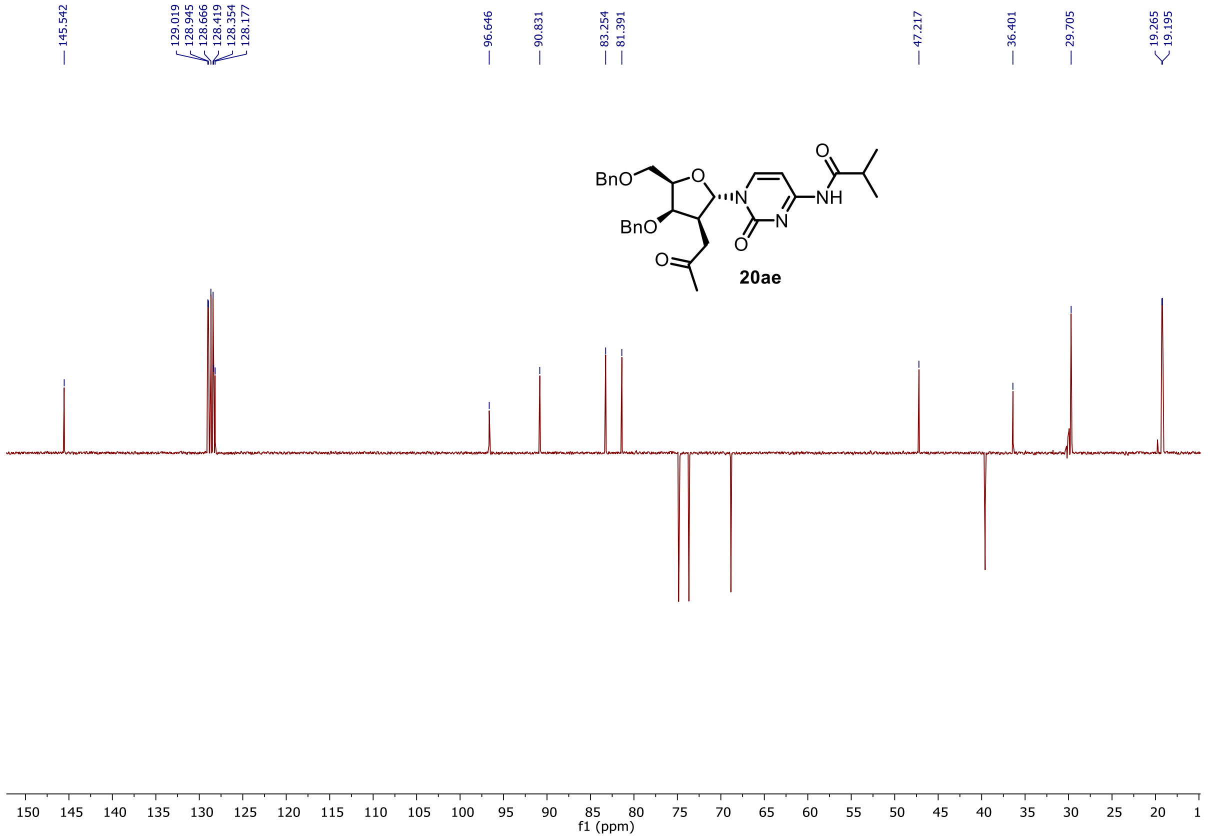

Supplementary Figure 242. DEPT spectra for **20ae**

Gated ^{13}C (without decoupling of proton) spectrum of **20ae**

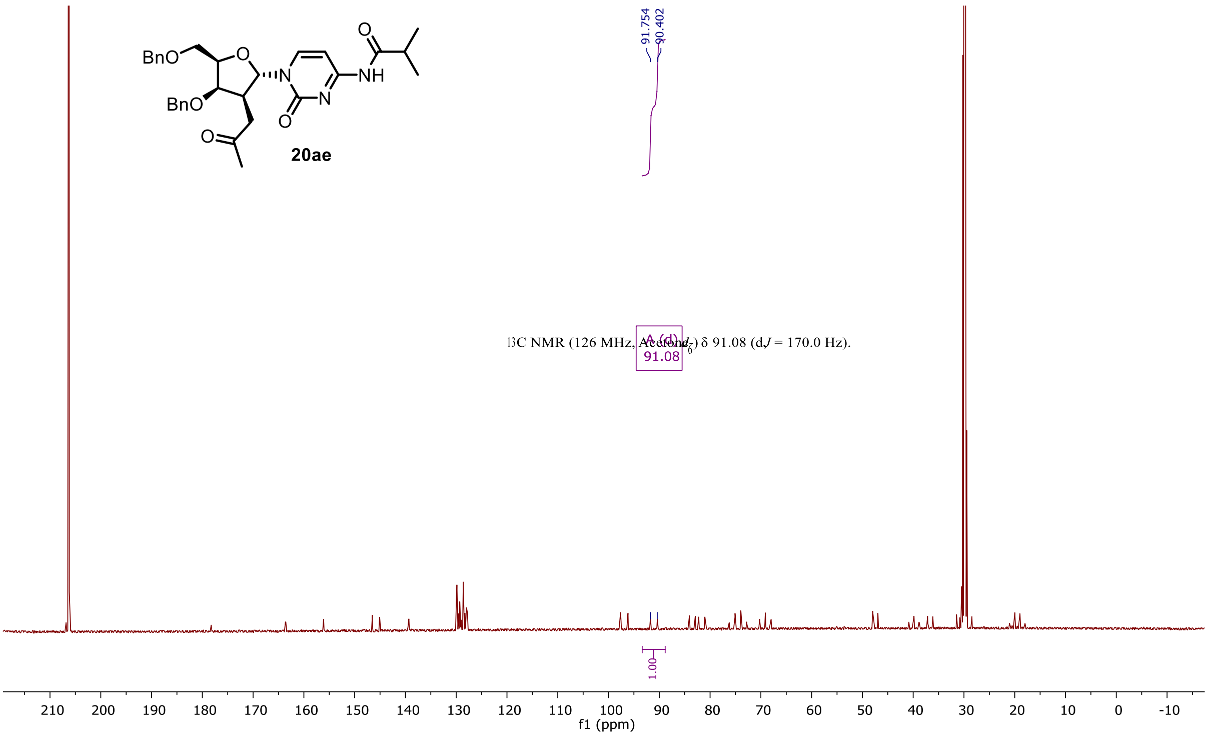

Supplementary Figure 243. Gated ^{13}C (with coupling of proton) spectra for **20ae**

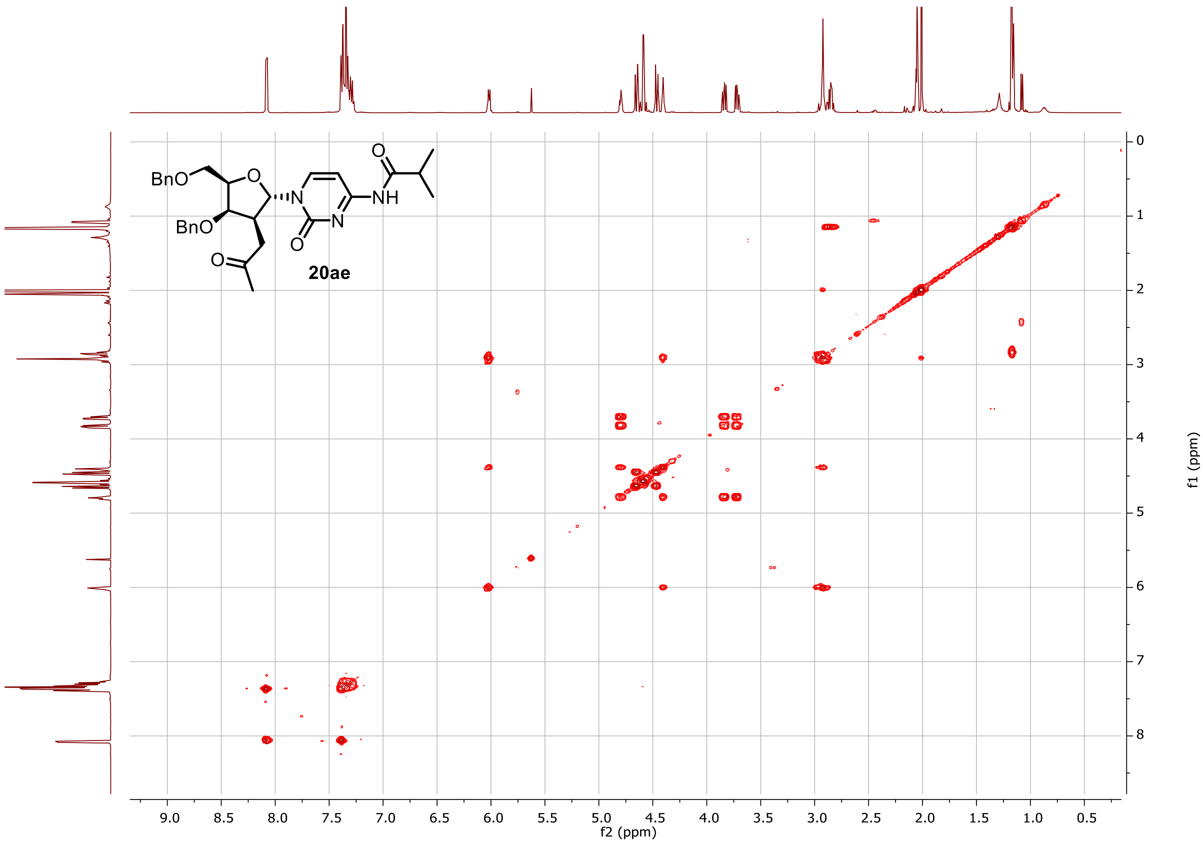

Supplementary Figure 244. COSY spectra for **20ae**

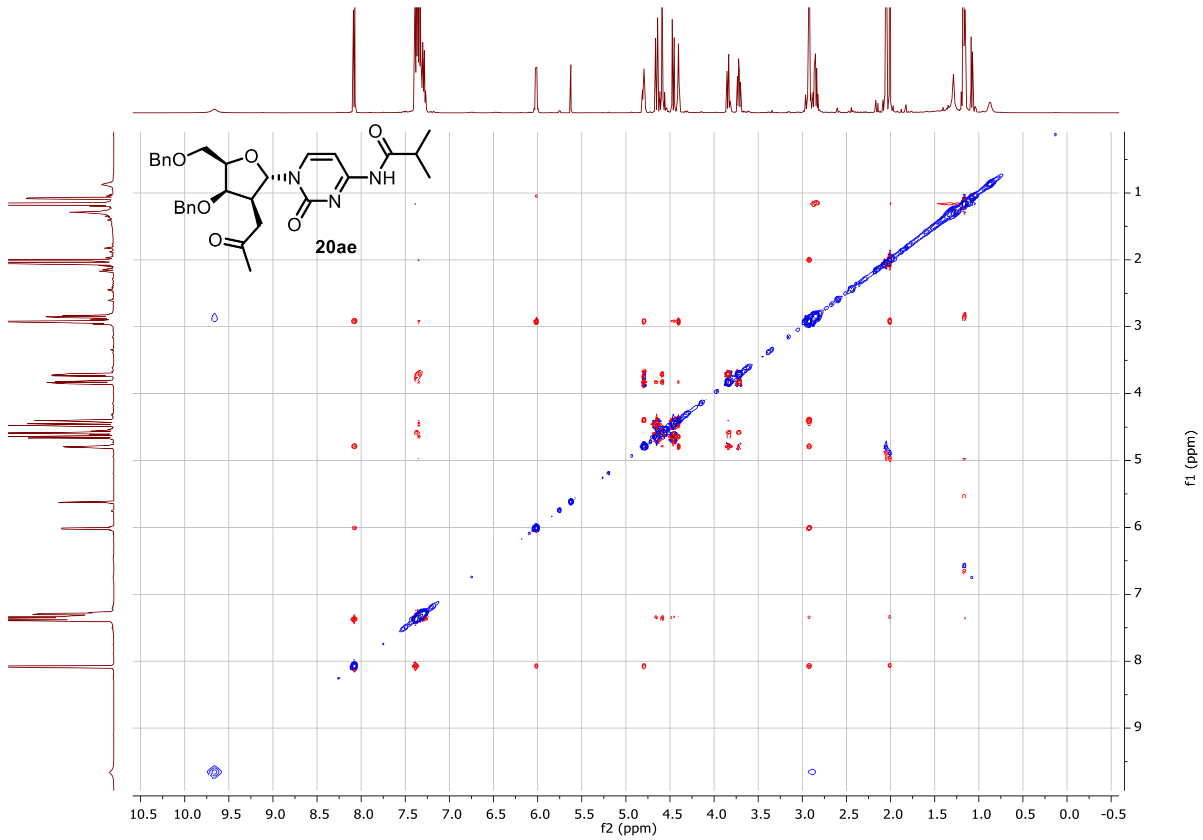

Supplementary Figure 245. NOESY spectra for **20ae**

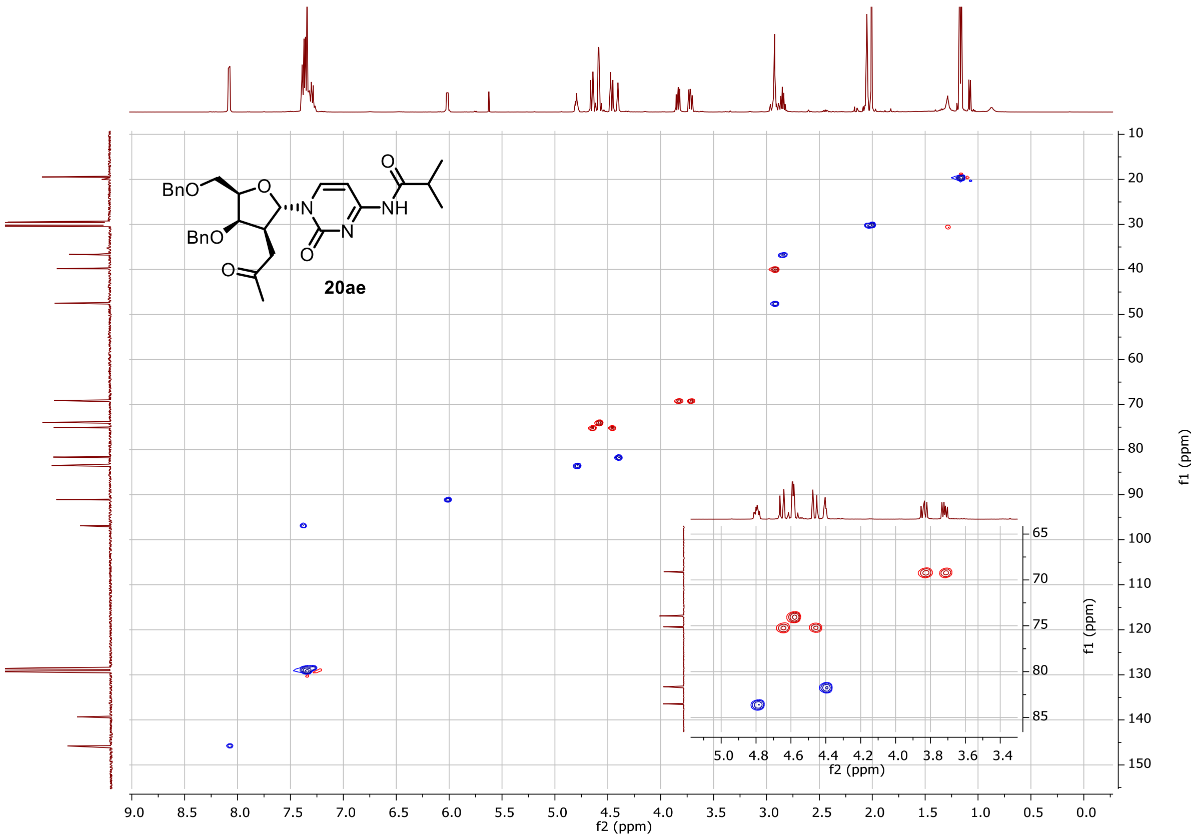

Supplementary Figure 246. HSQC spectra for **20ae**

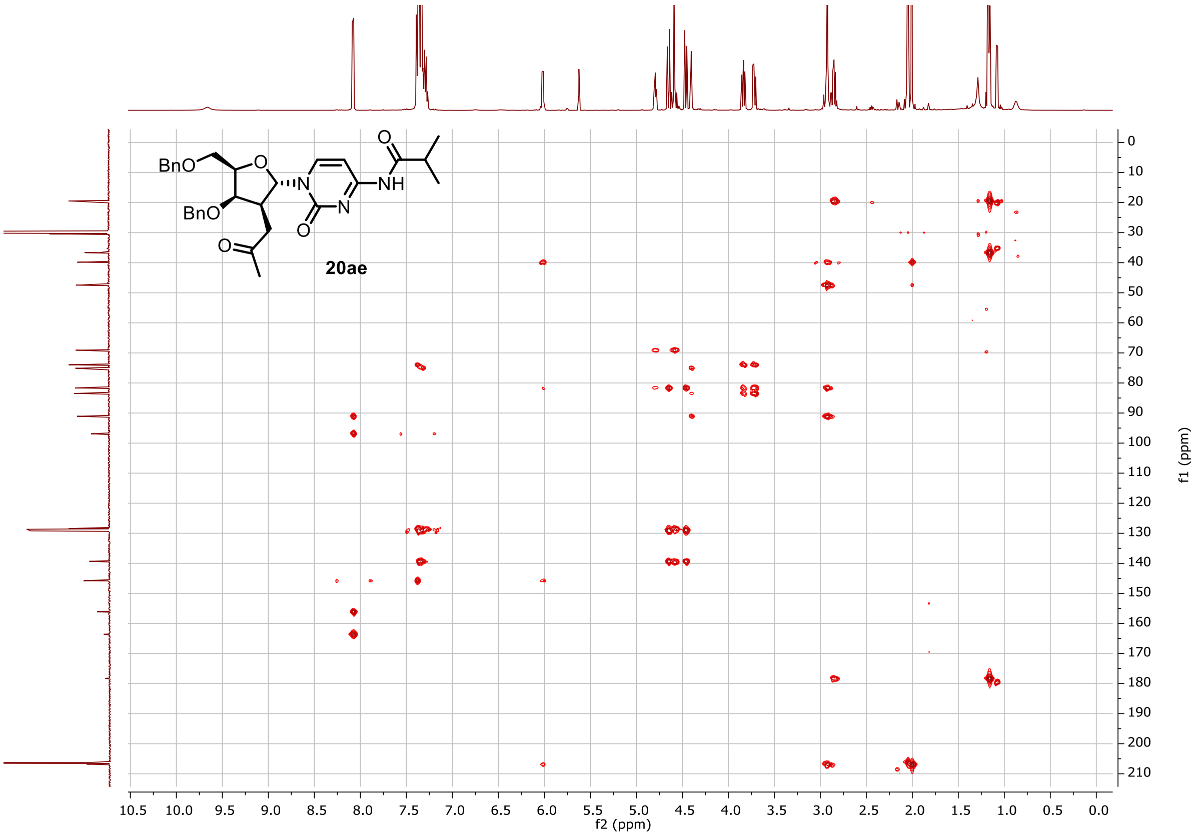

Supplementary Figure 247. HMBC spectra for **20ae**

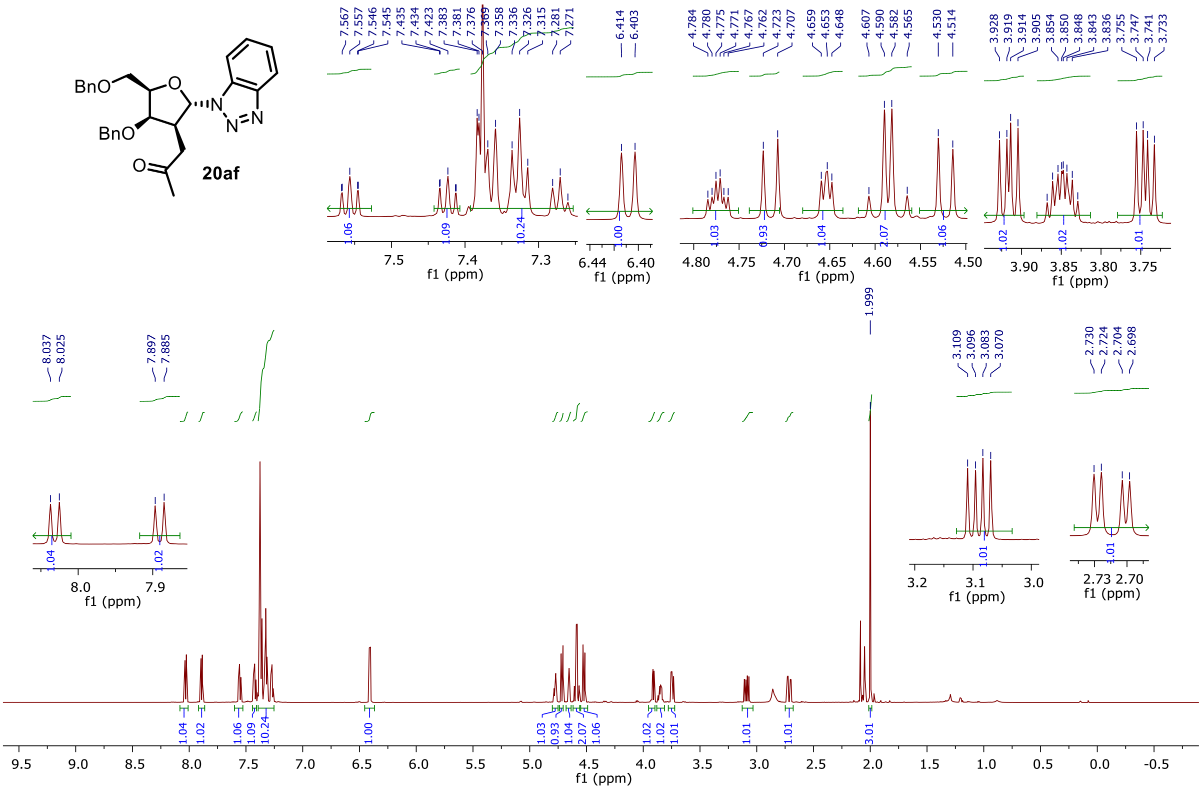

Supplementary Figure 248. ^{1}H spectra for **20af**

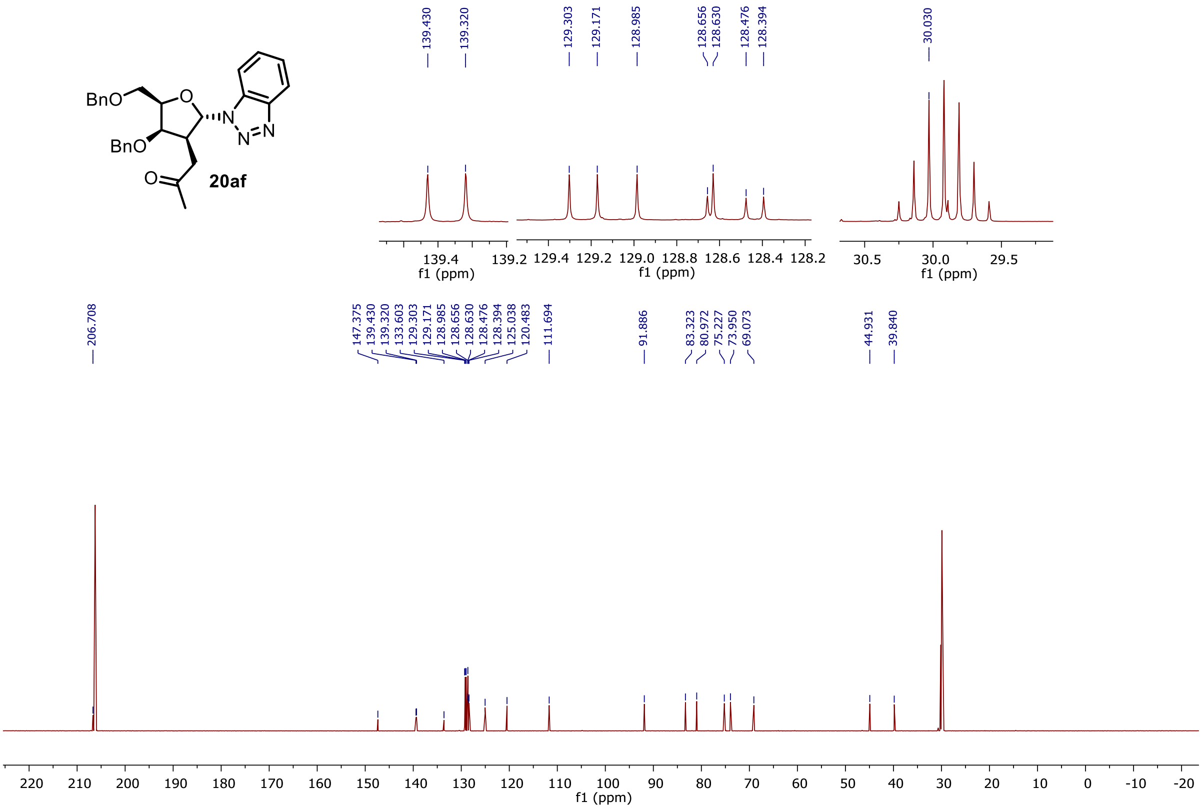

Supplementary Figure 249. ^{13}C spectra for **20af**

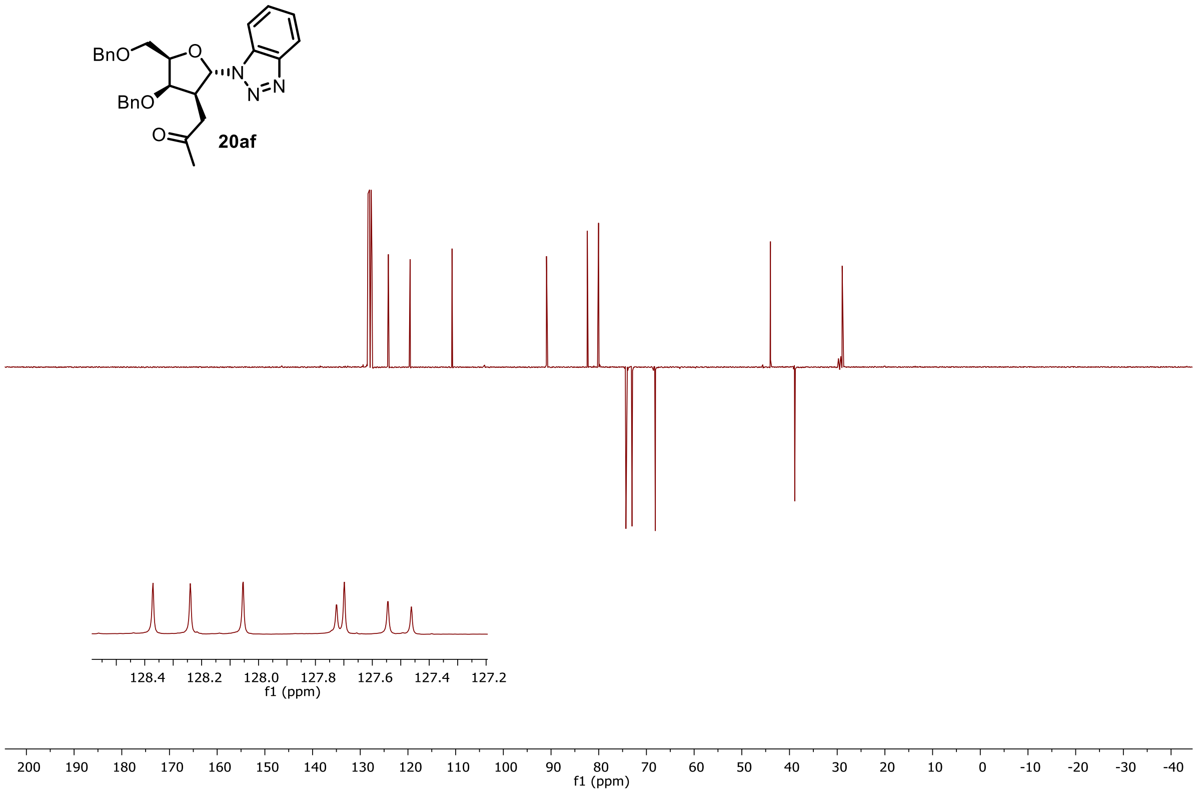

Supplementary Figure 250. DEPT spectra for **20af**

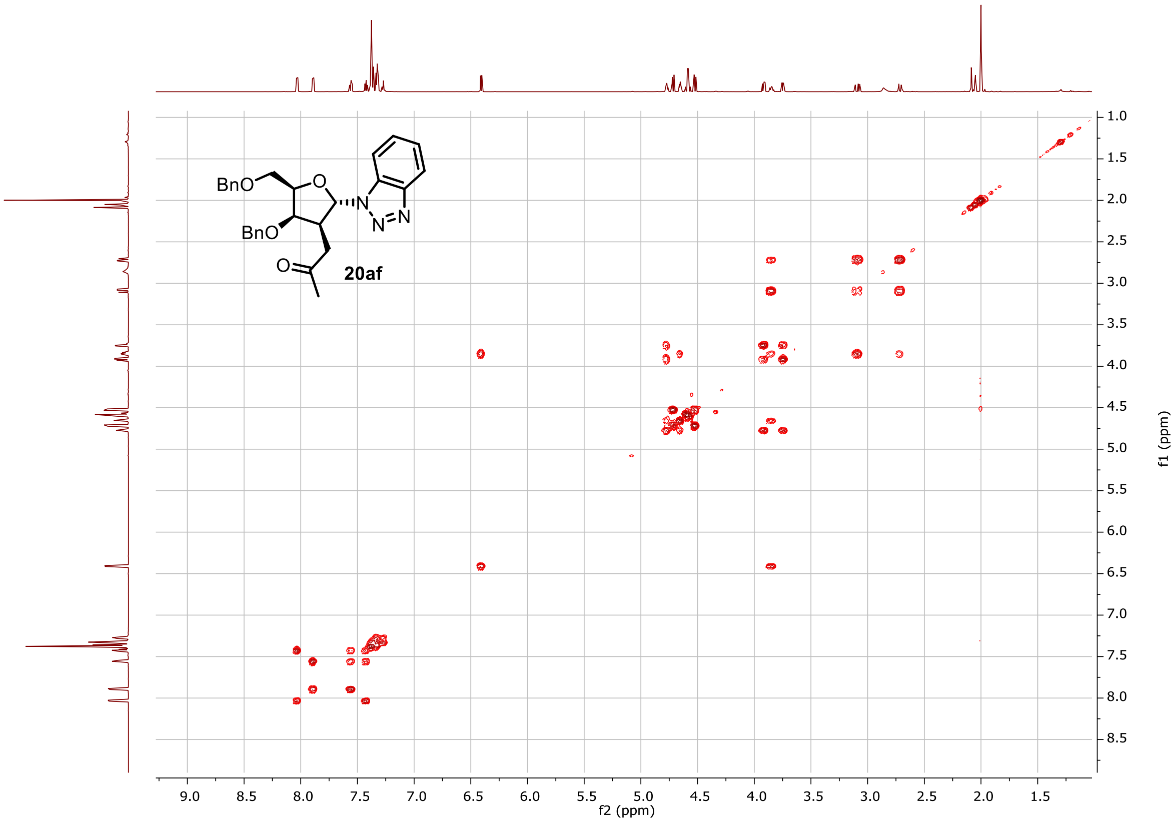

Supplementary Figure 251. COSY spectra for **20af**

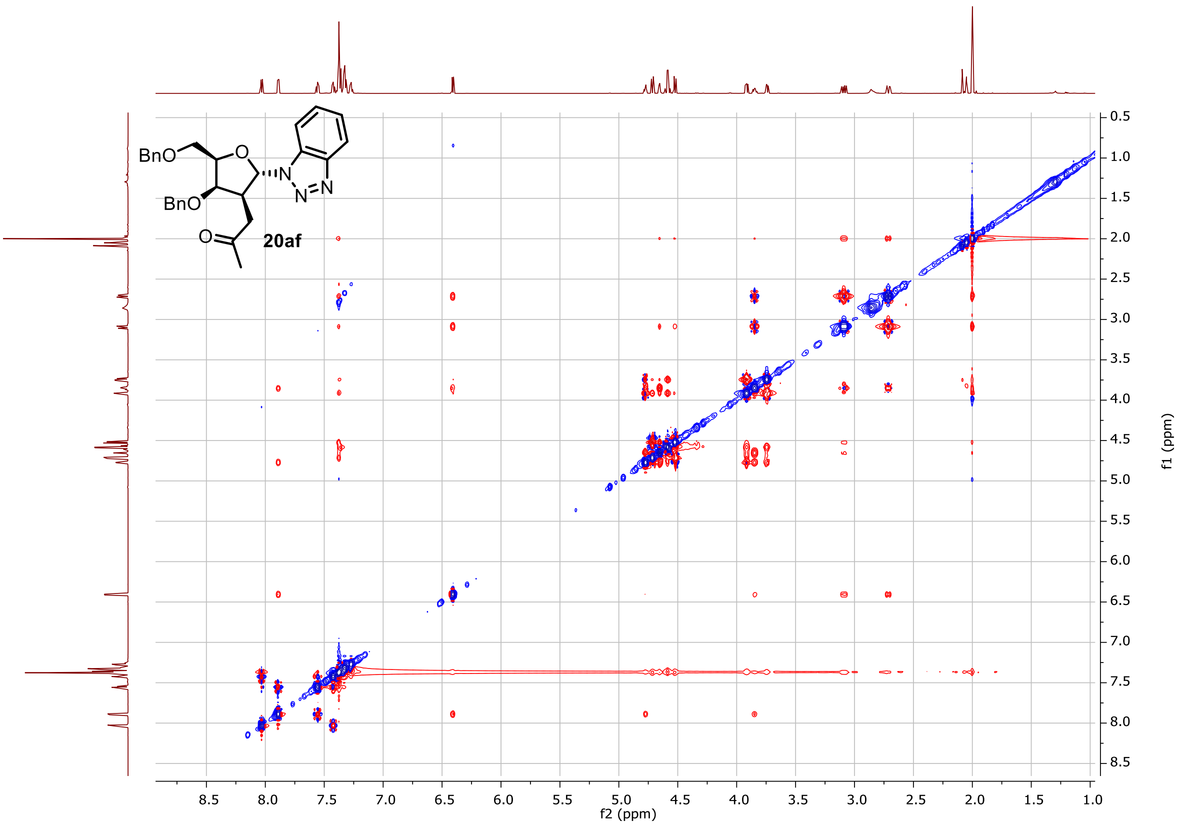

Supplementary Figure 252. NOESY spectra for **20af**

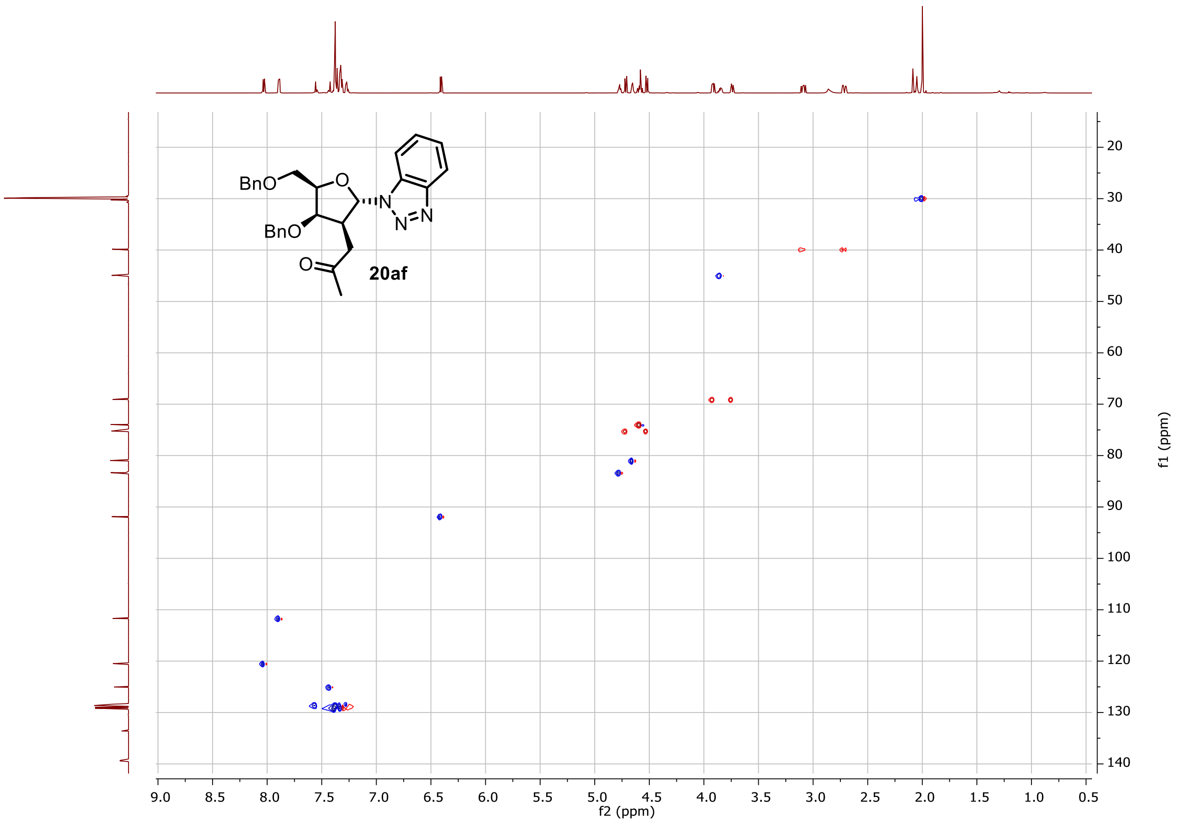

Supplementary Figure 253. HSQC spectra for **20af**

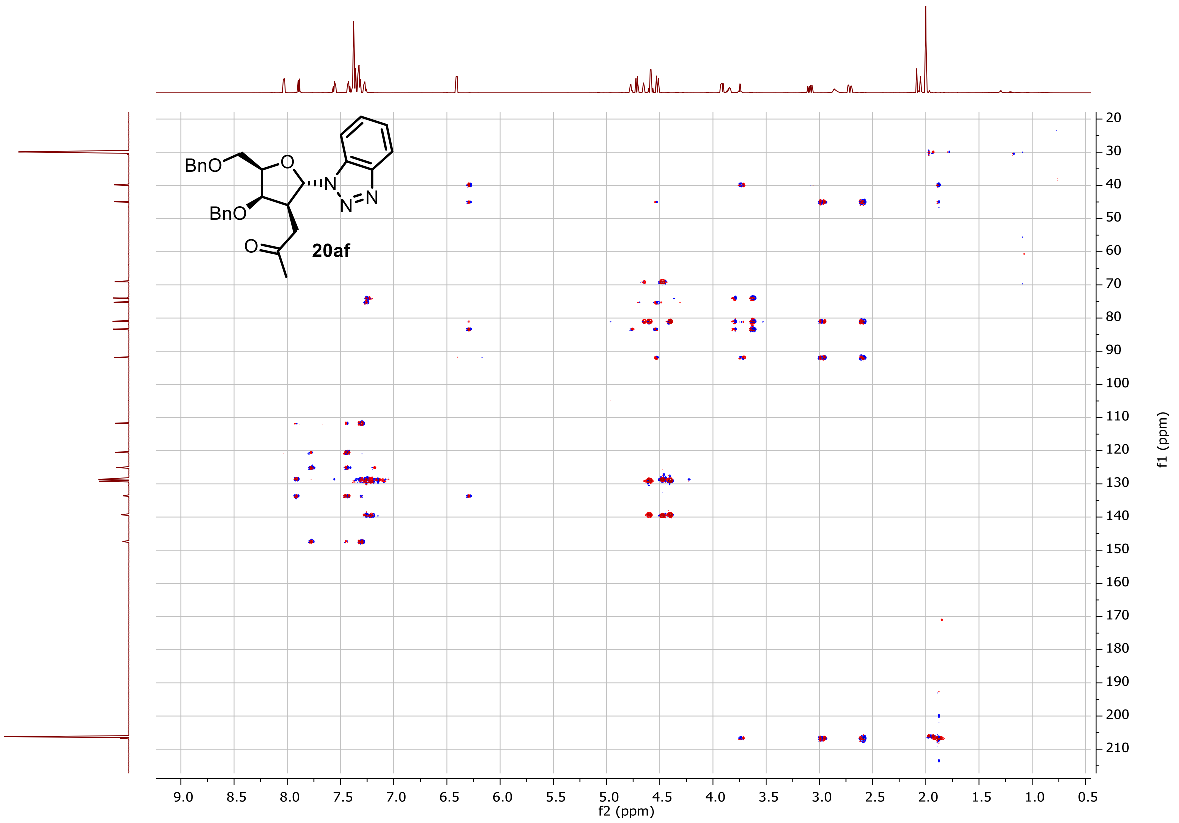

Supplementary Figure 254. HMBC spectra for **20af**

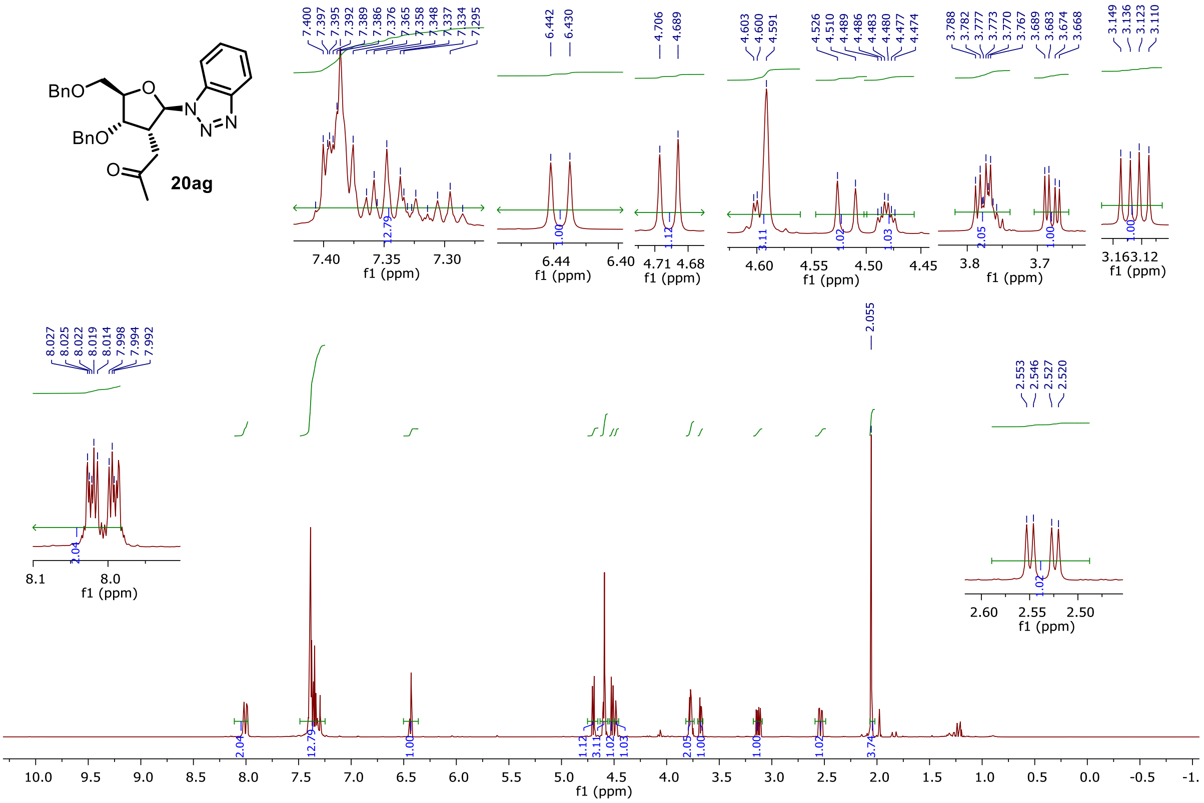

Supplementary Figure 255. ^{1}H spectra for **20ag**

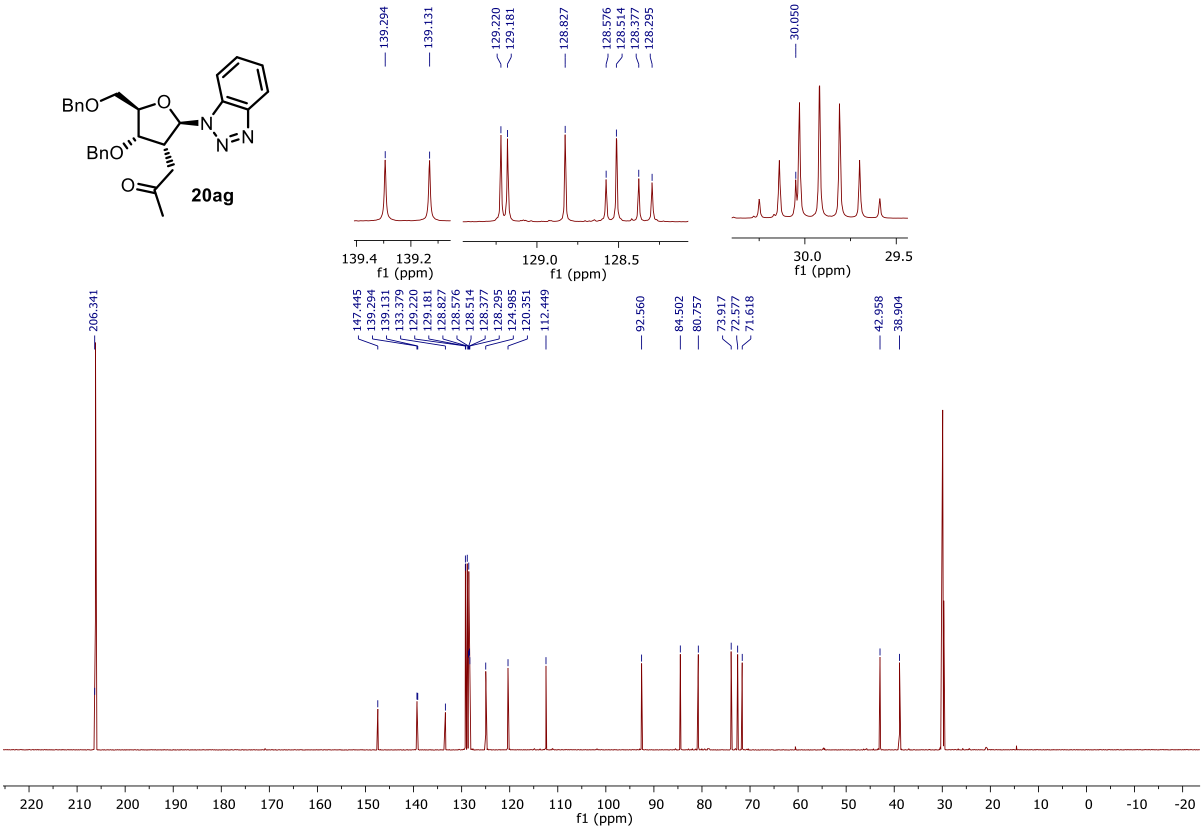

Supplementary Figure 256. ^{13}C spectra for **20ag**

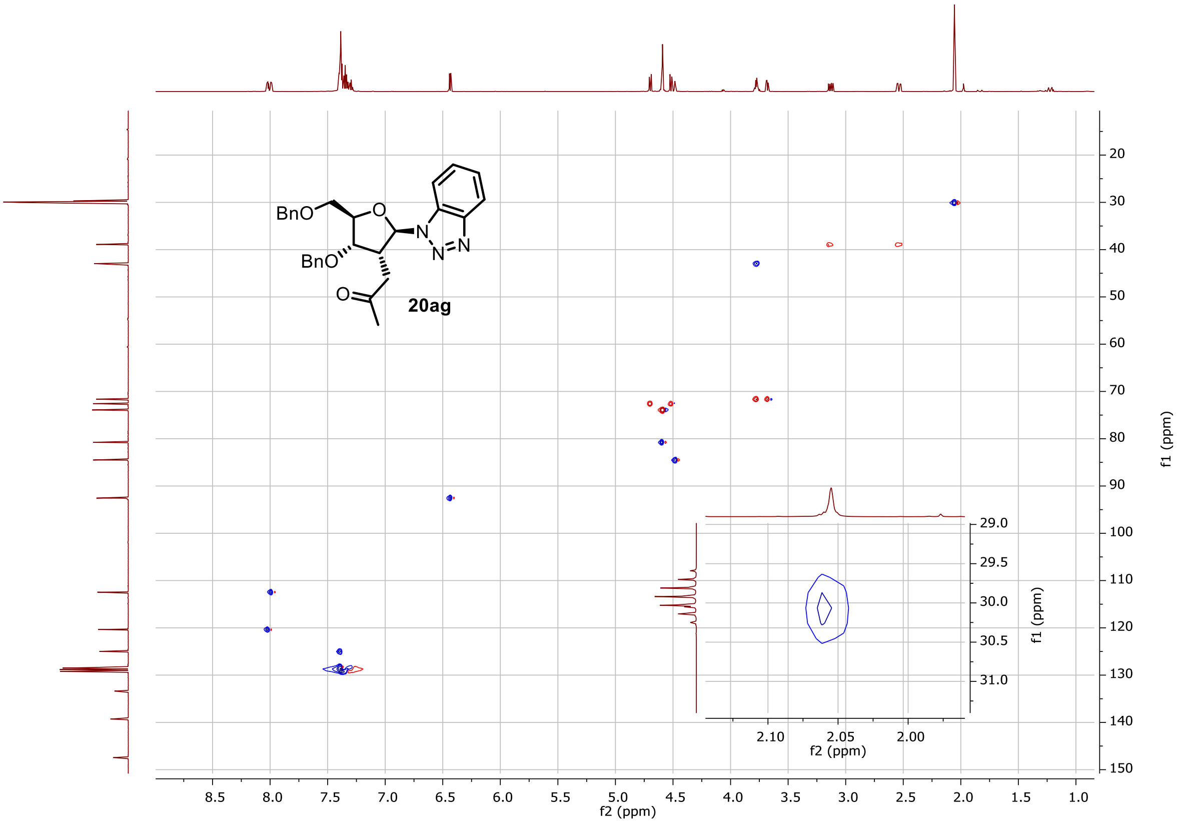

Supplementary Figure 257. HSQC spectra for **20ag**

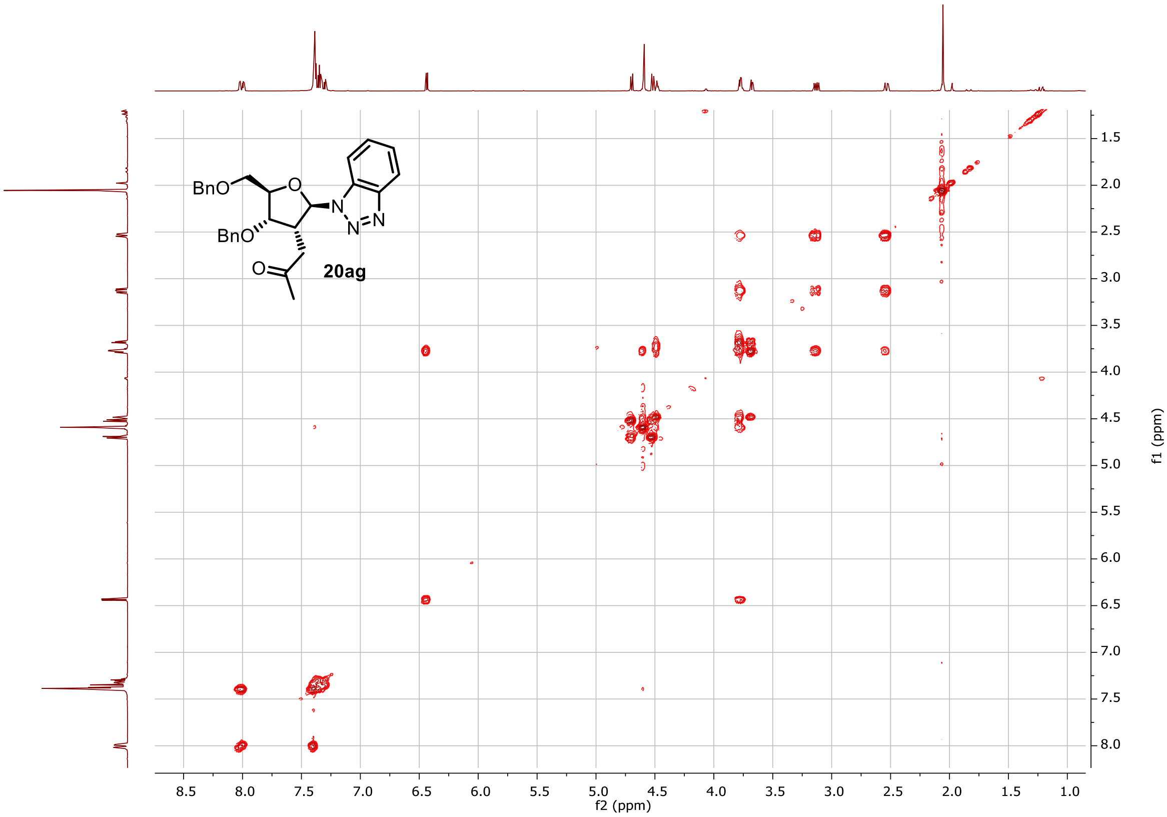

Supplementary Figure 258. COSY spectra for **20ag**

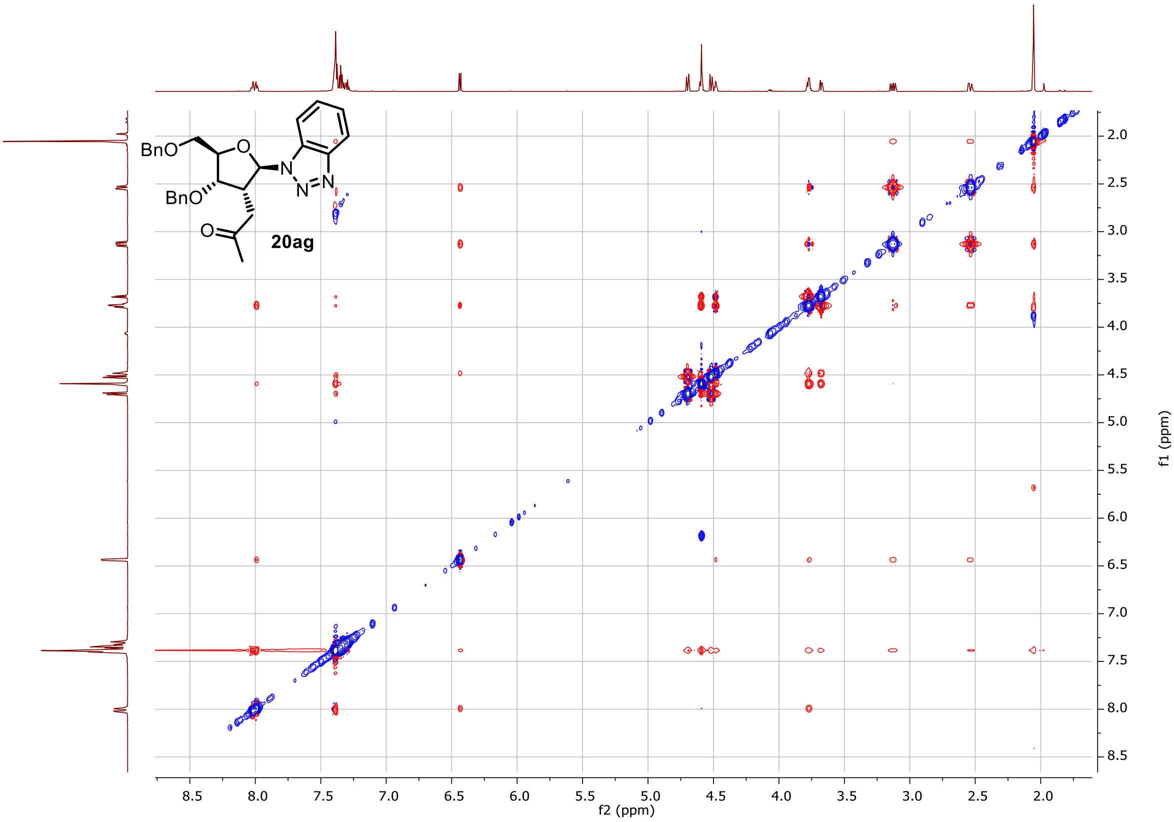

Supplementary Figure 259. NOESY spectra for **20ag**

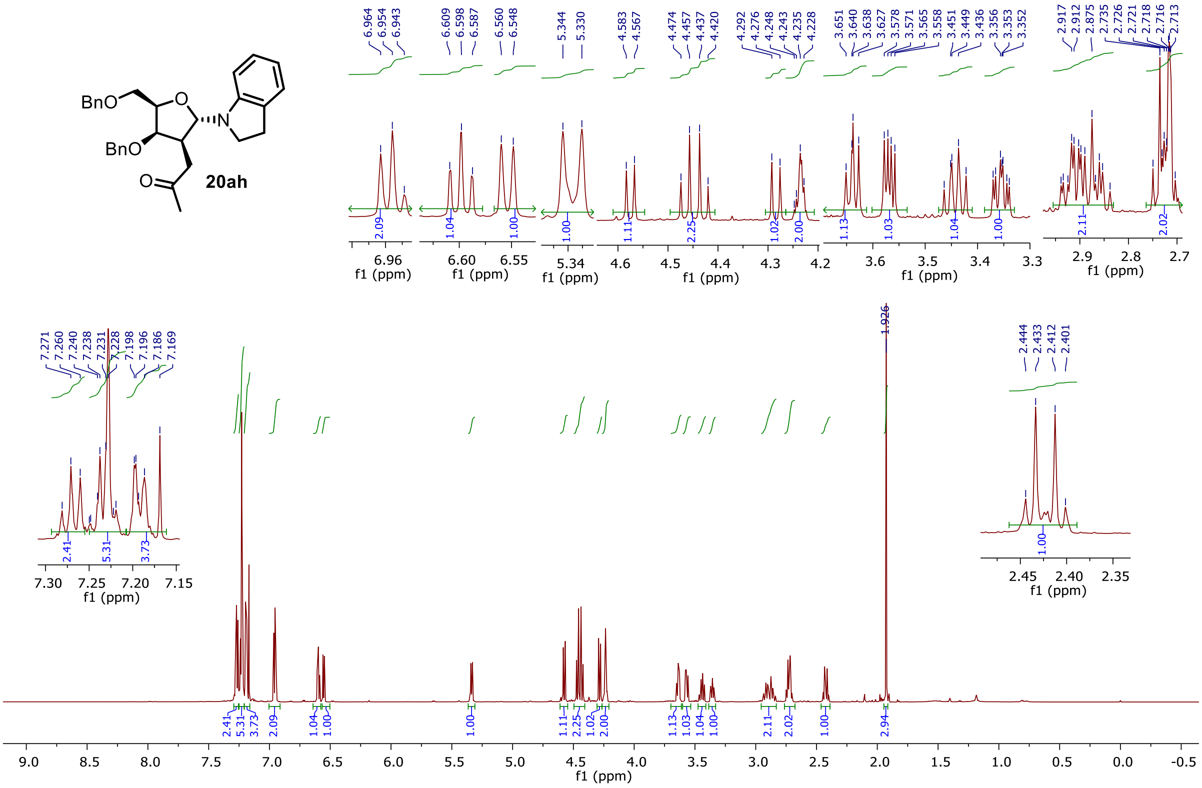

Supplementary Figure 260. ^{1}H spectra for **20ah**

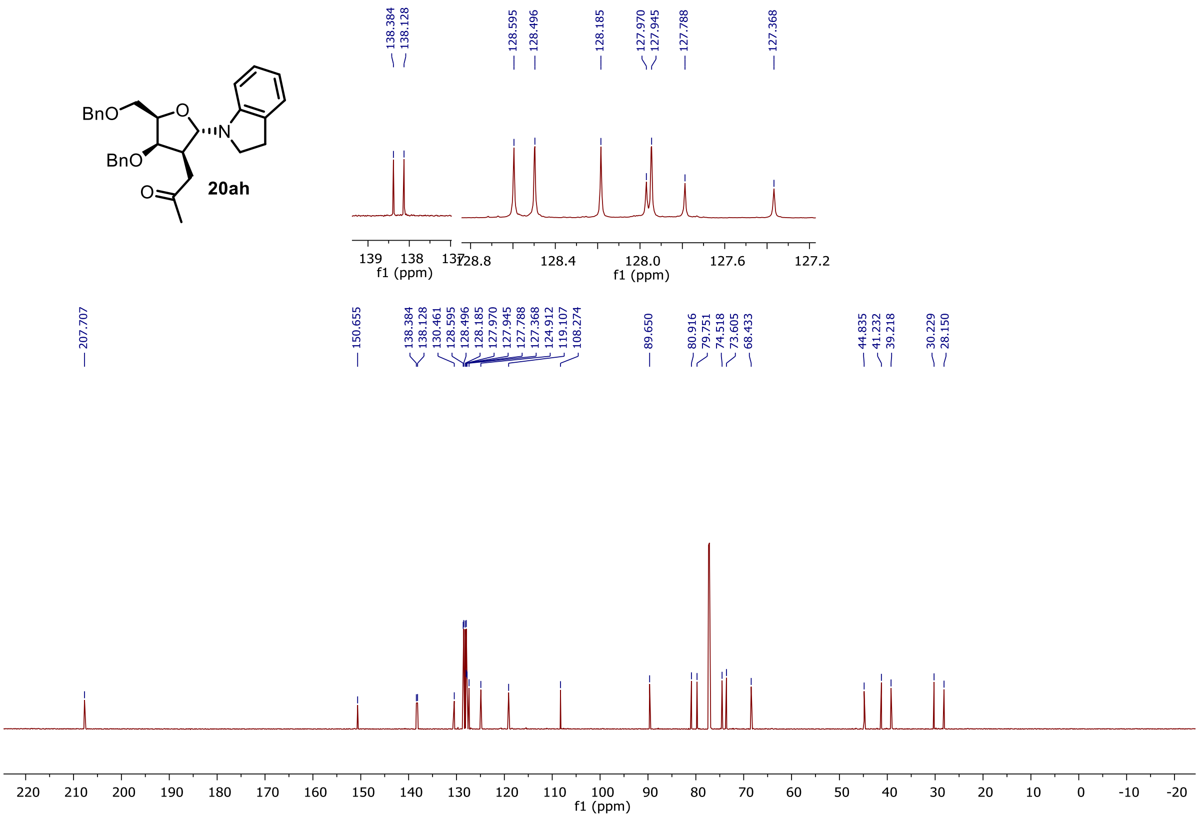

Supplementary Figure 261. ^{13}C spectra for **20ah**

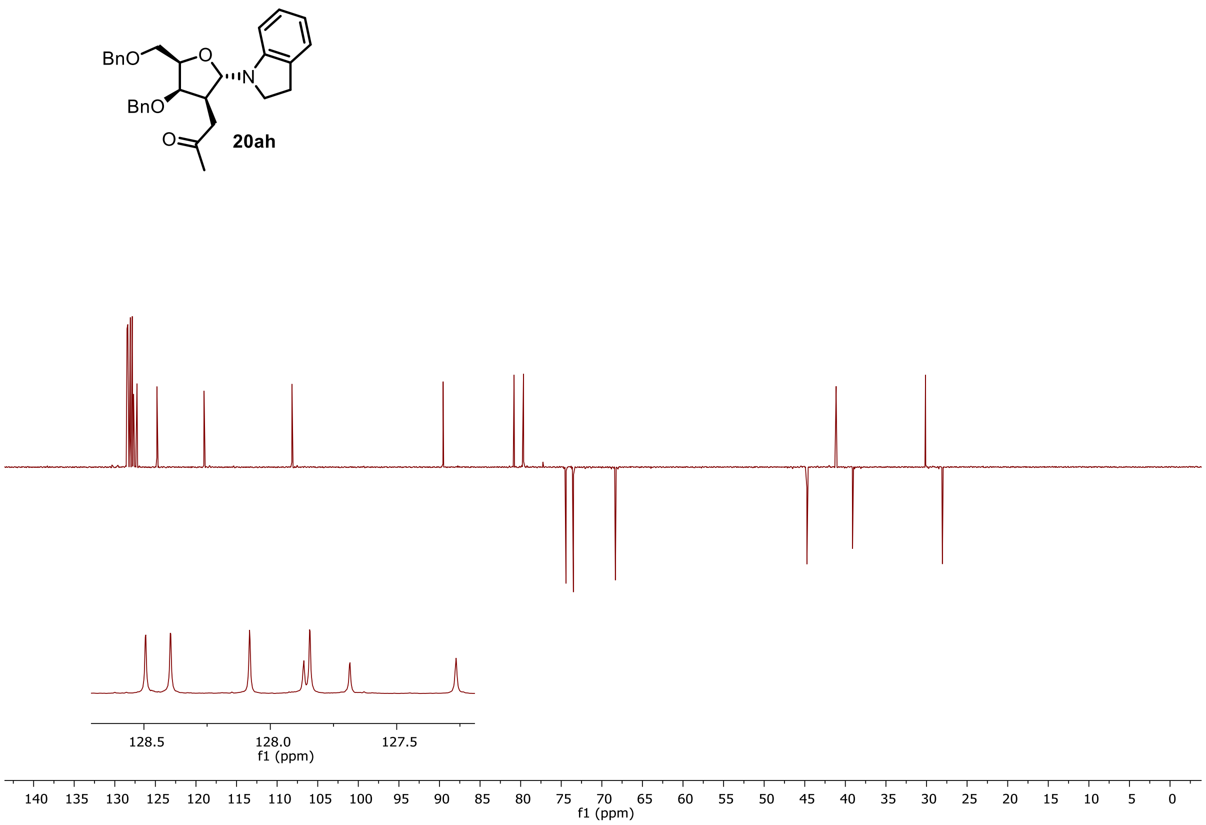

Supplementary Figure 262. DEPT spectra for **20ah**

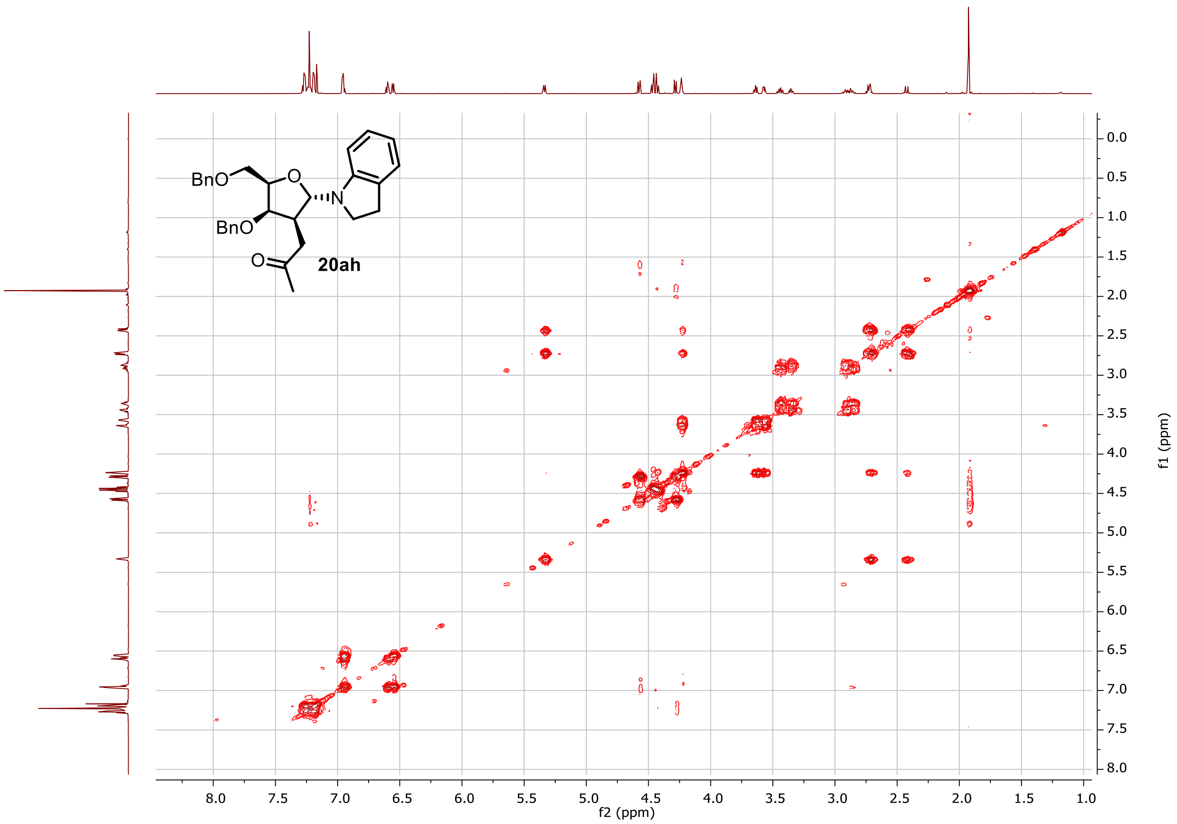

Supplementary Figure 263. COSY spectra for **20ah**

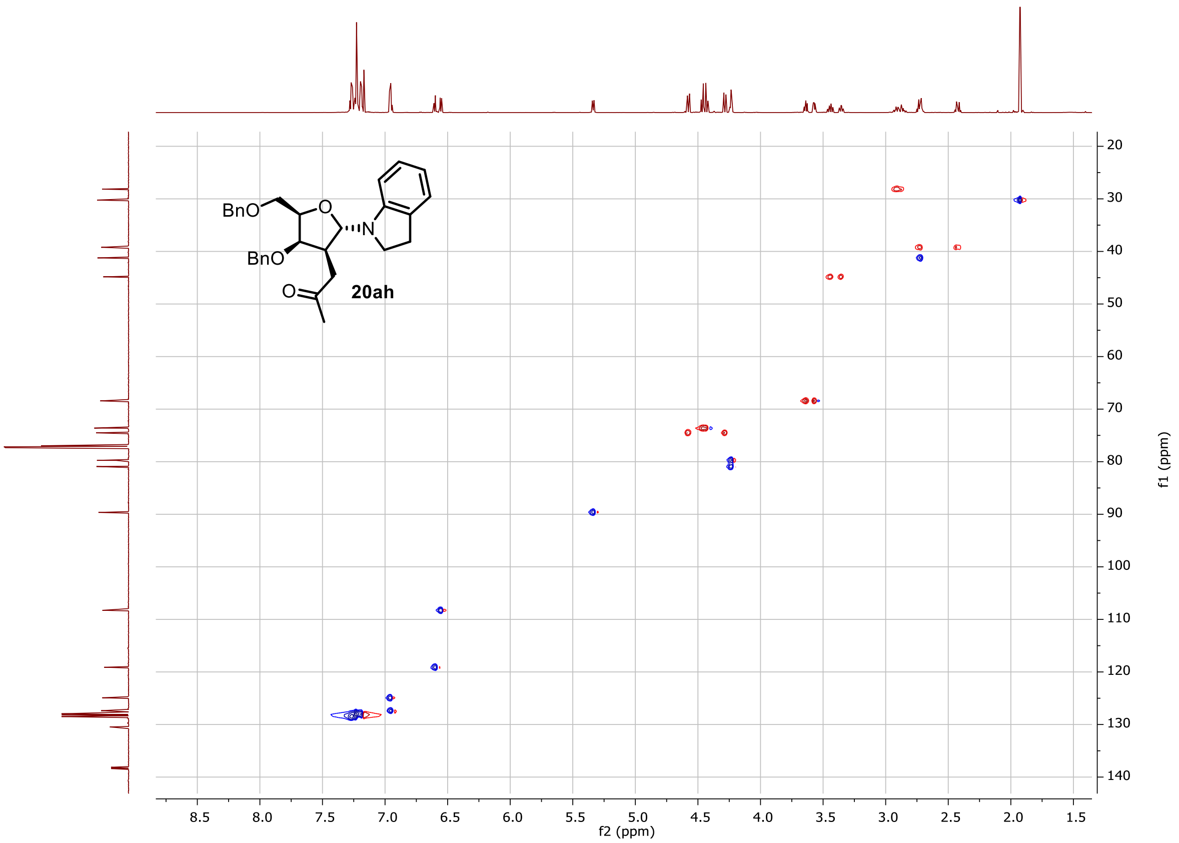

Supplementary Figure 264. HSQC spectra for **20ah**

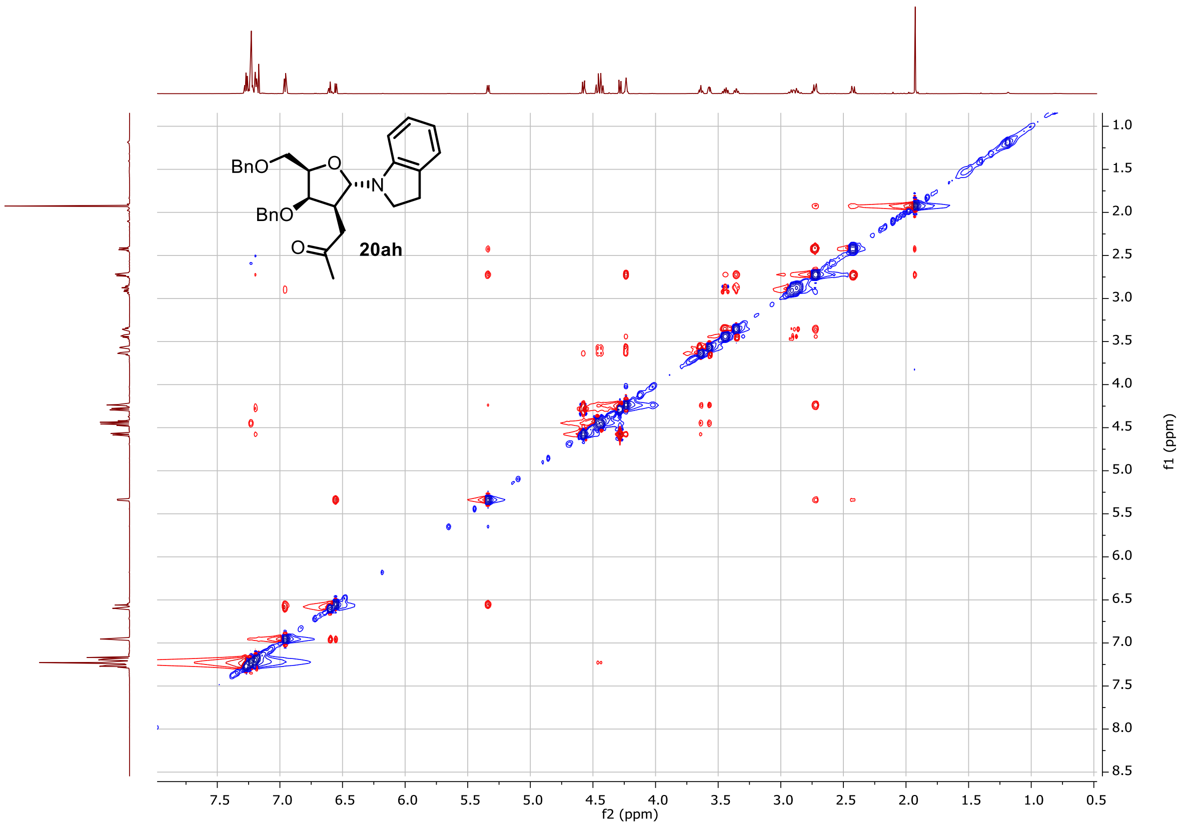

Supplementary Figure 265. NOESY spectra for **20ah**

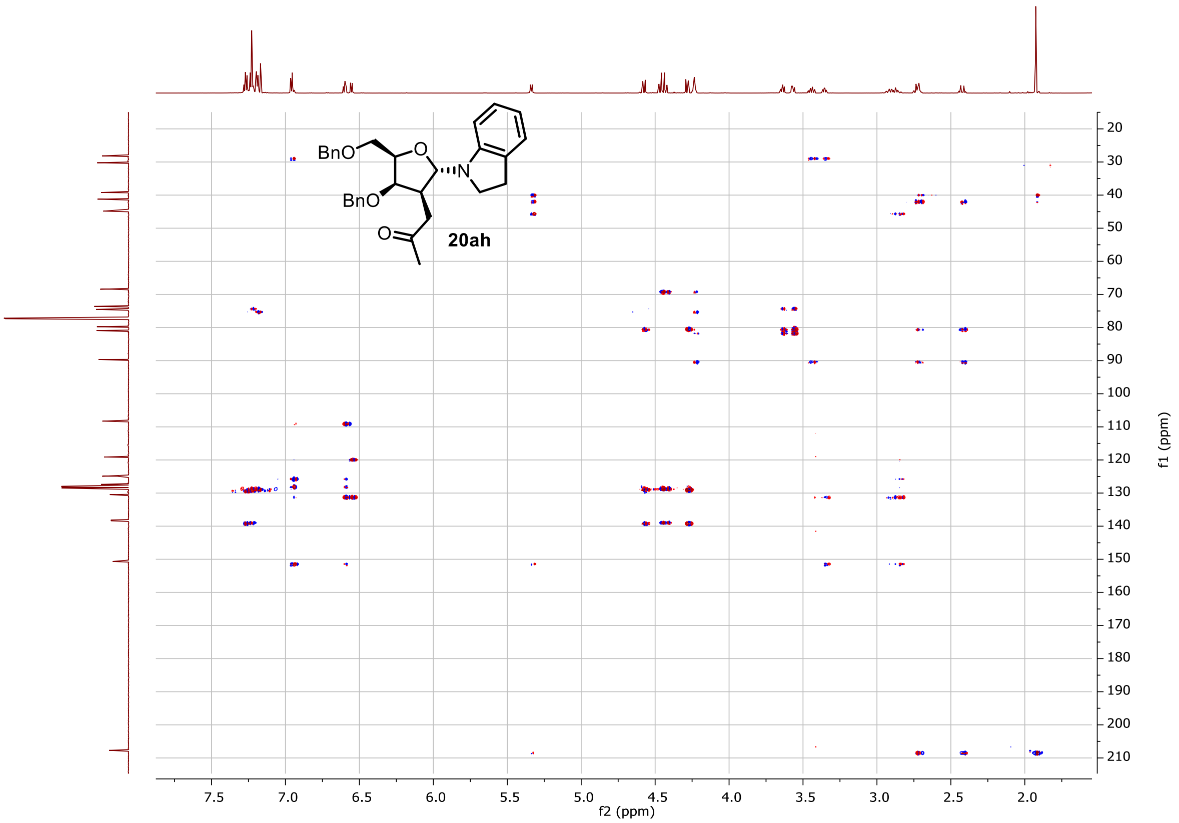

Supplementary Figure 266. HMBC spectra for **20ah**

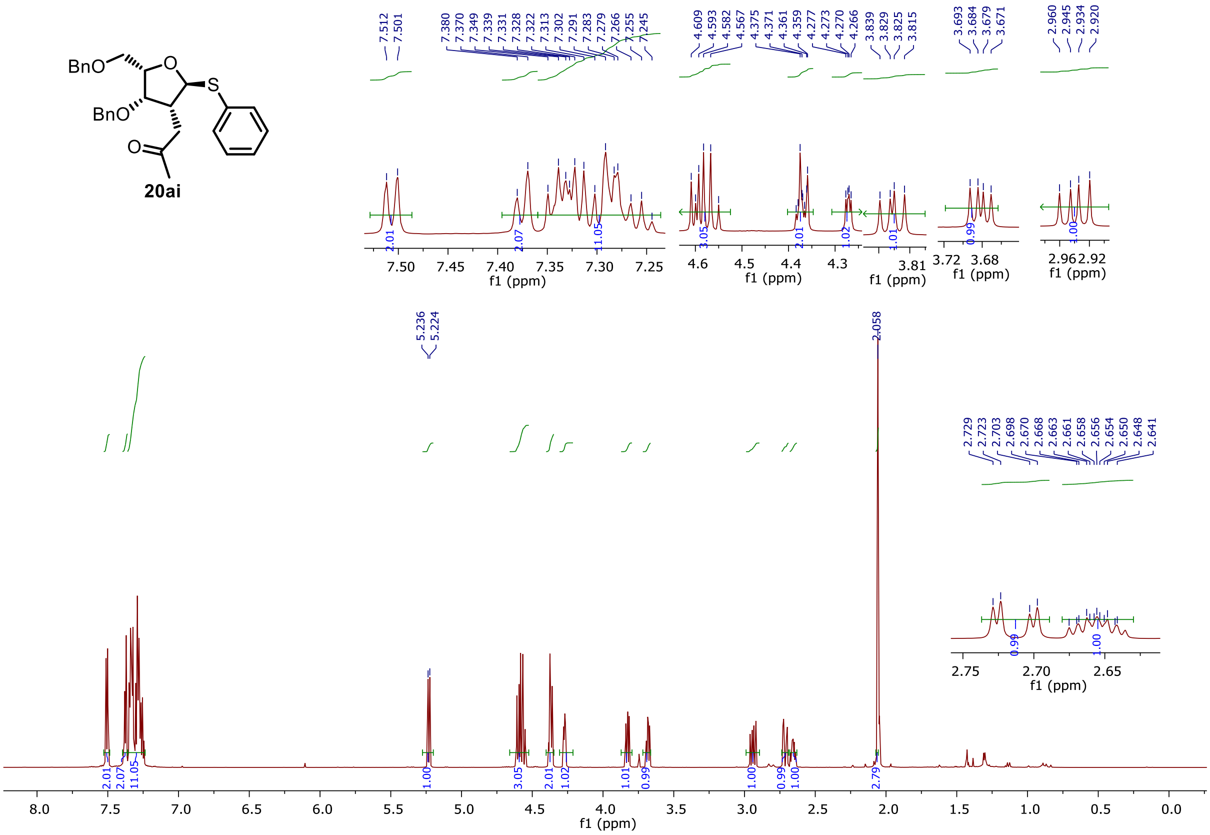

Supplementary Figure 267. ^{1}H spectra for **20ai**

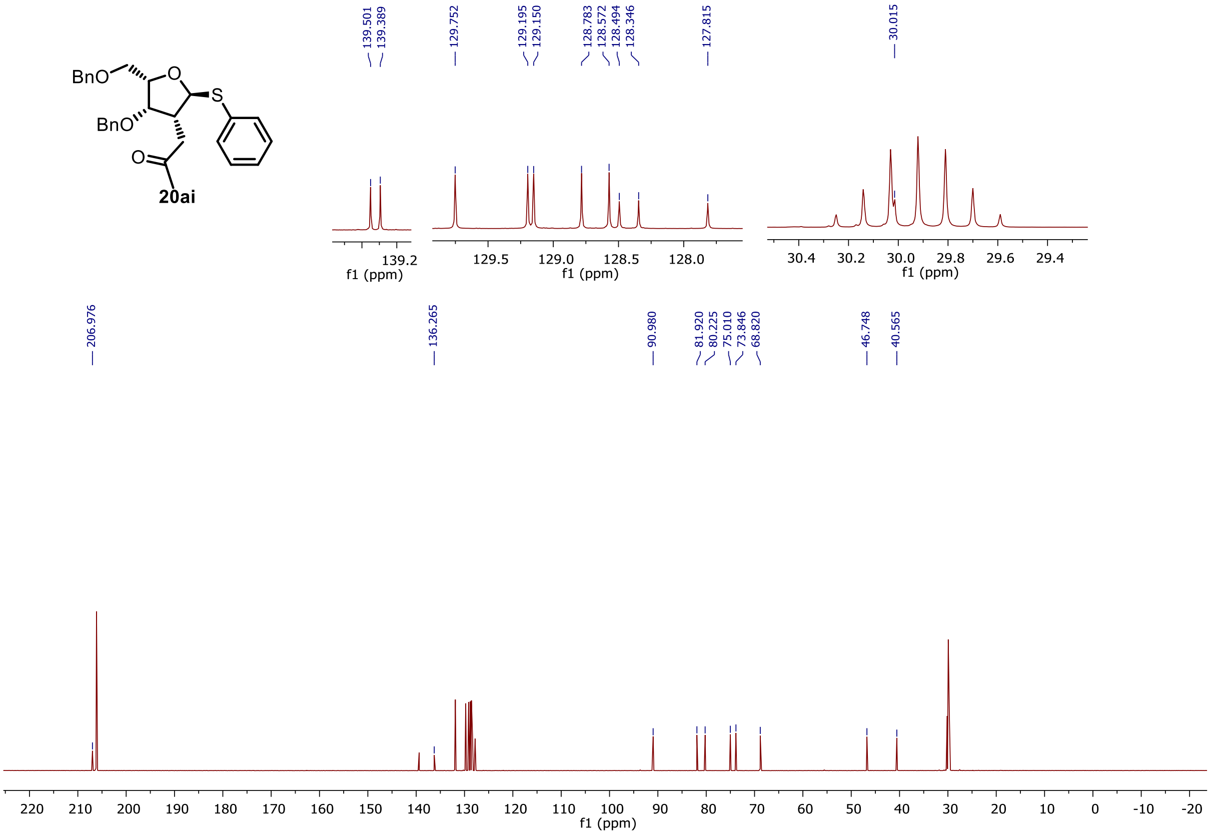

Supplementary Figure 268. ^{13}C spectra for **20ai**

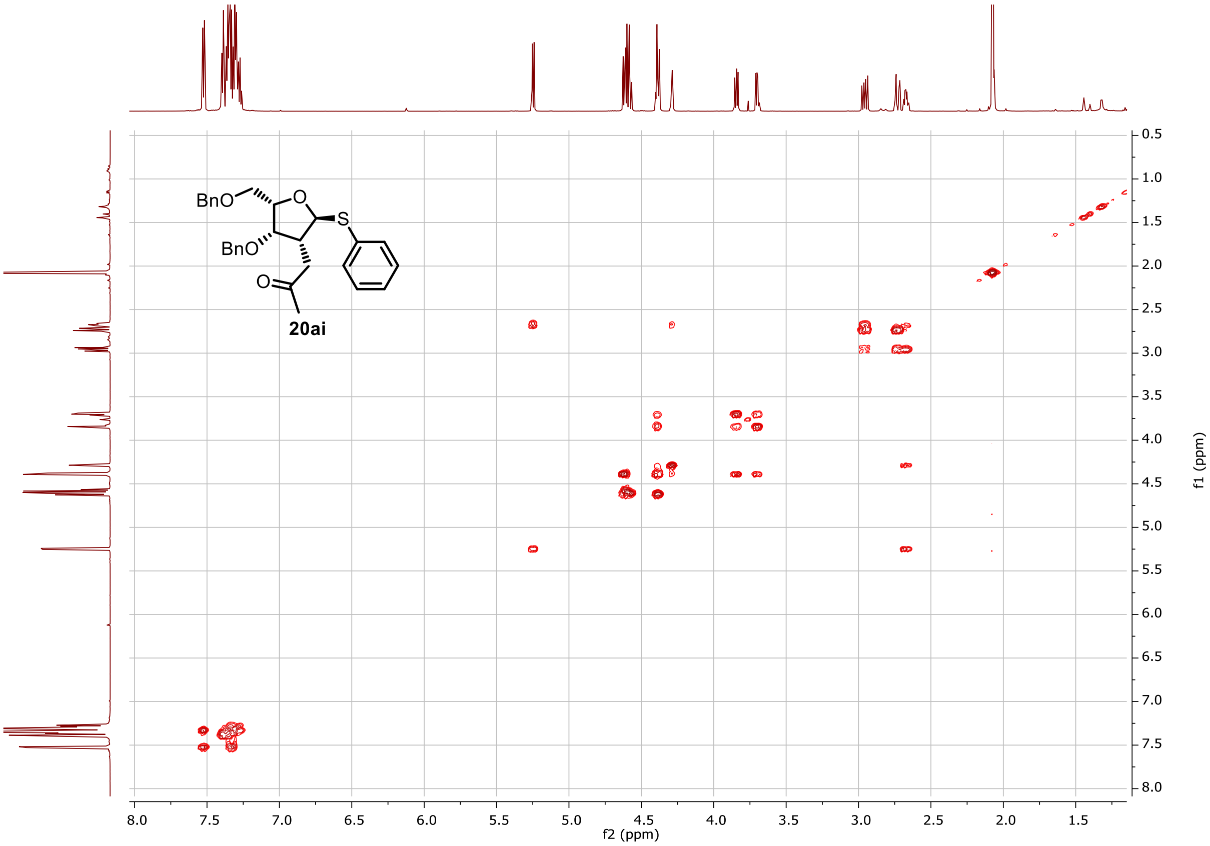

Supplementary Figure 269. COSY spectra for **20ai**

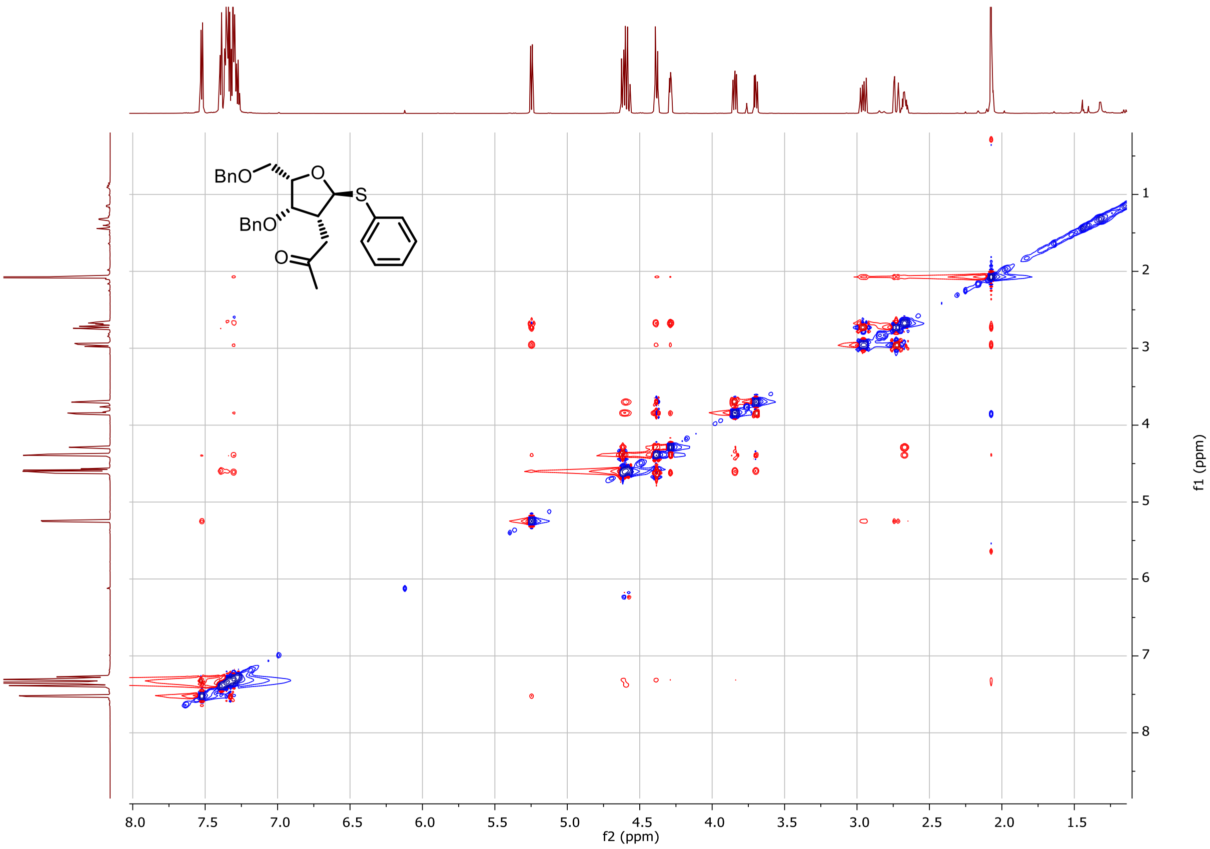

Supplementary Figure 270. NOESY spectra for **20ai**

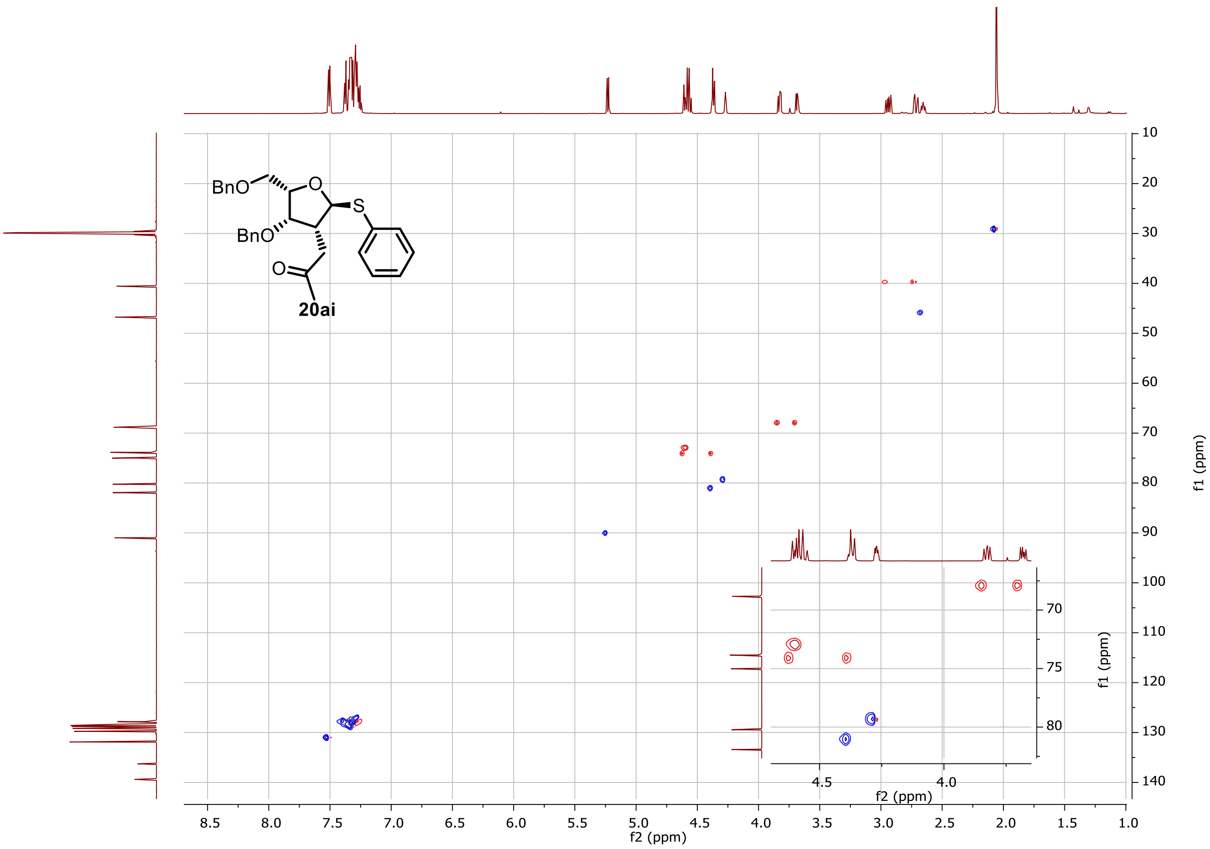

Supplementary Figure 271. HSQC spectra for **20ai**

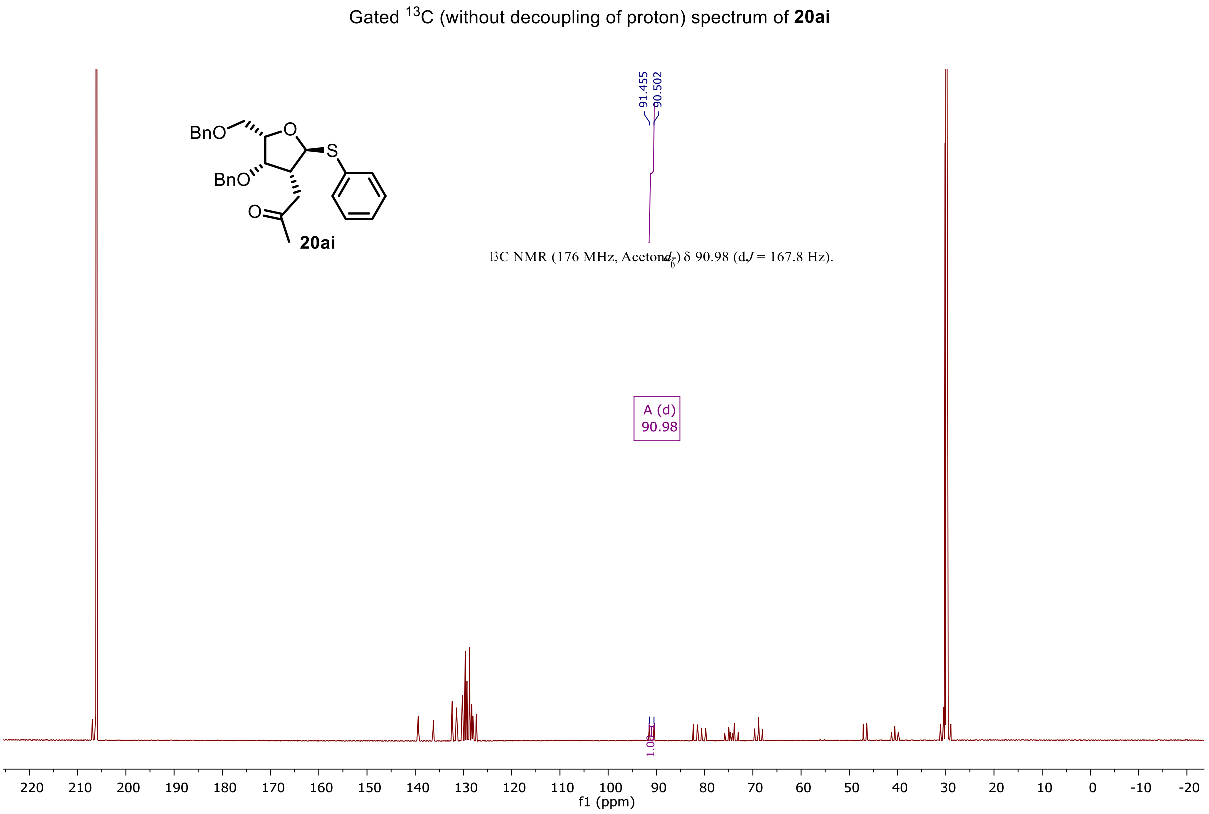

Supplementary Figure 272. Gated ^{13}C (with coupling of proton) spectra for **20ai**

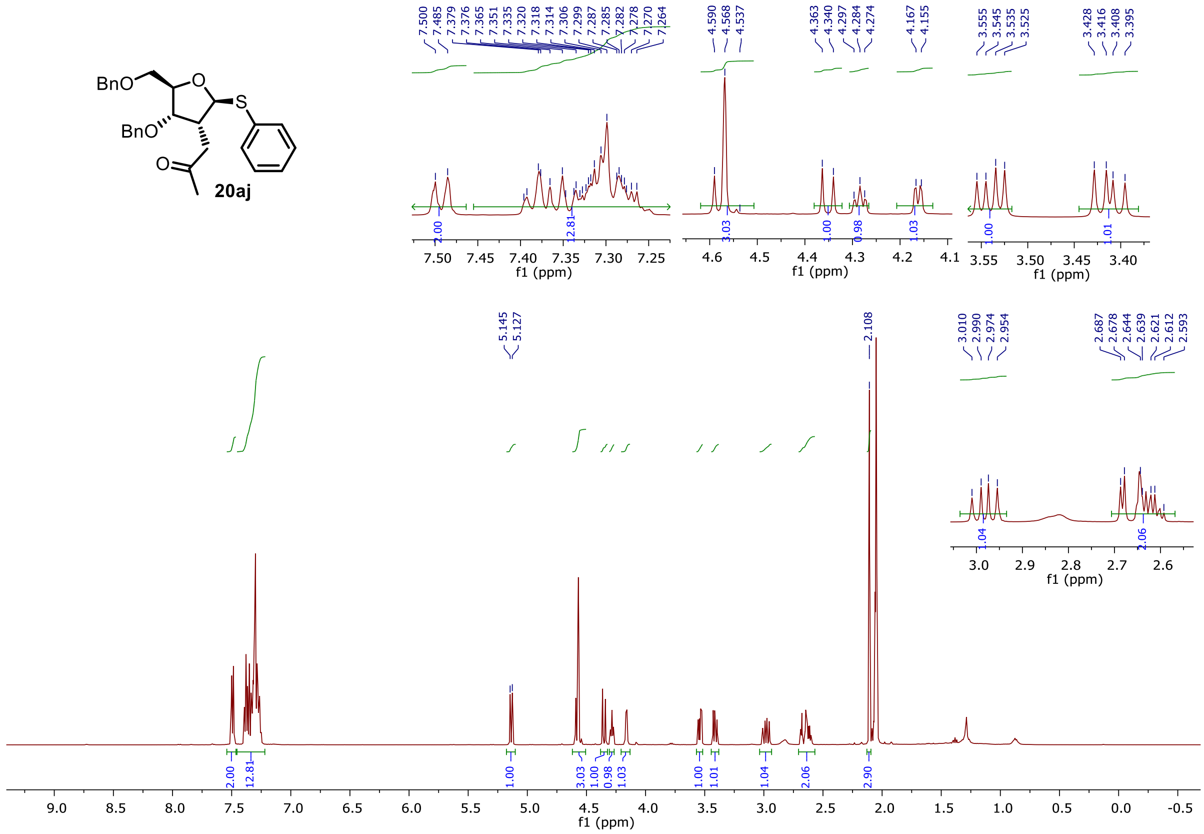

Supplementary Figure 273. ^{1}H spectra for **20aj**

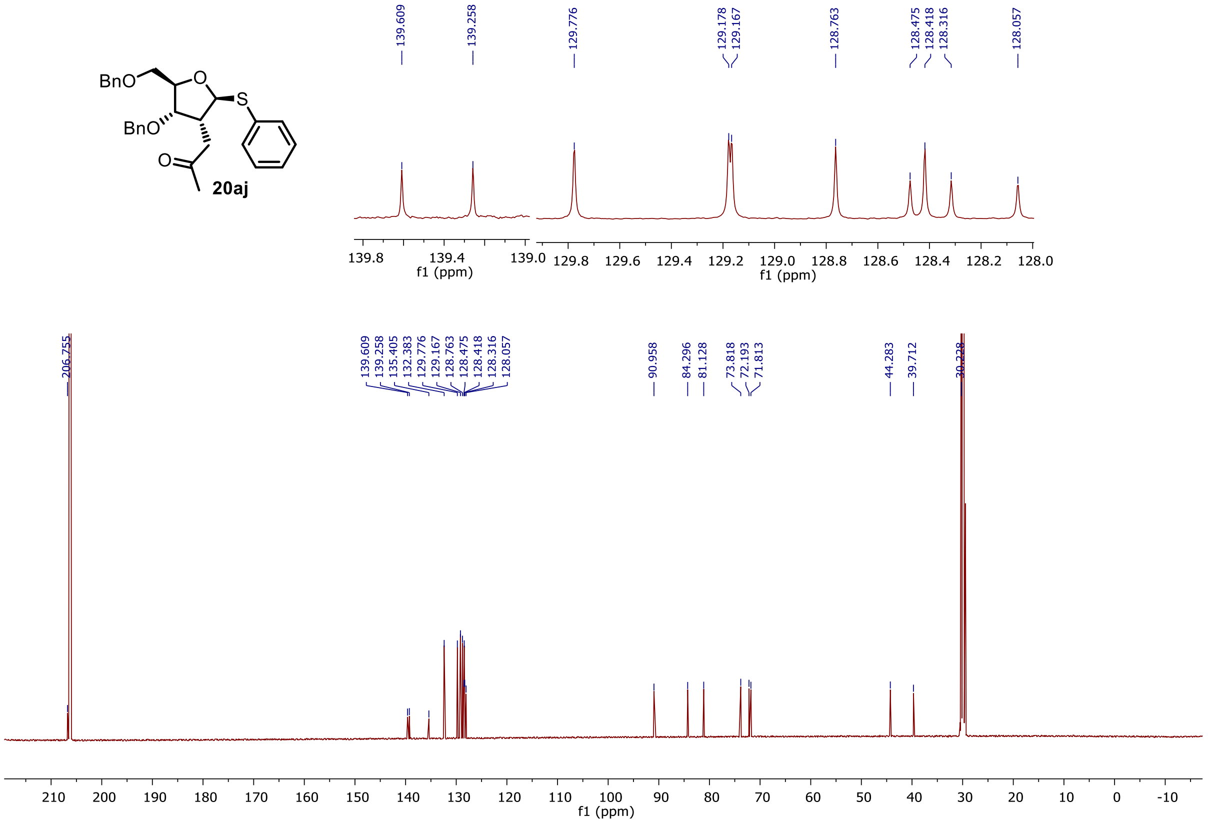

Supplementary Figure 274. ^{13}C spectra for **20aj**

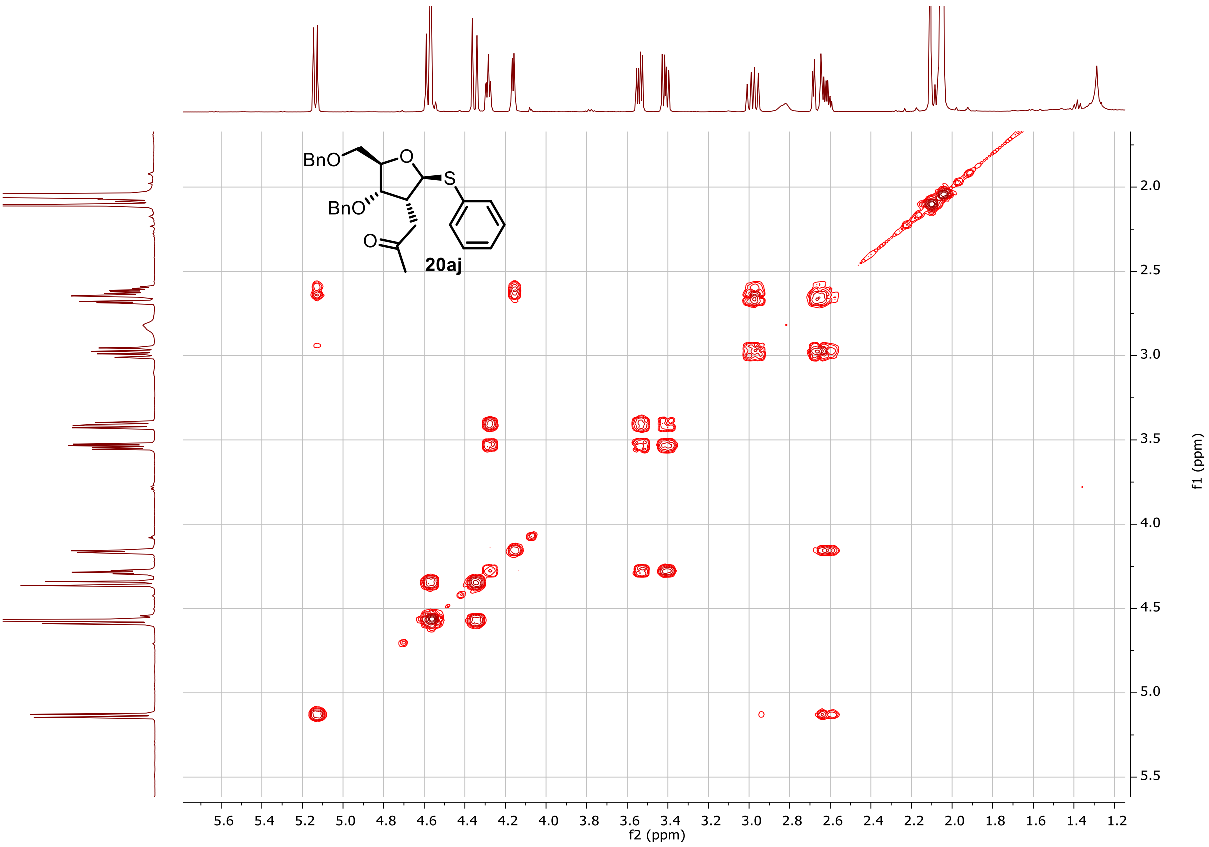

Supplementary Figure 275. COSY spectra for **20aj**

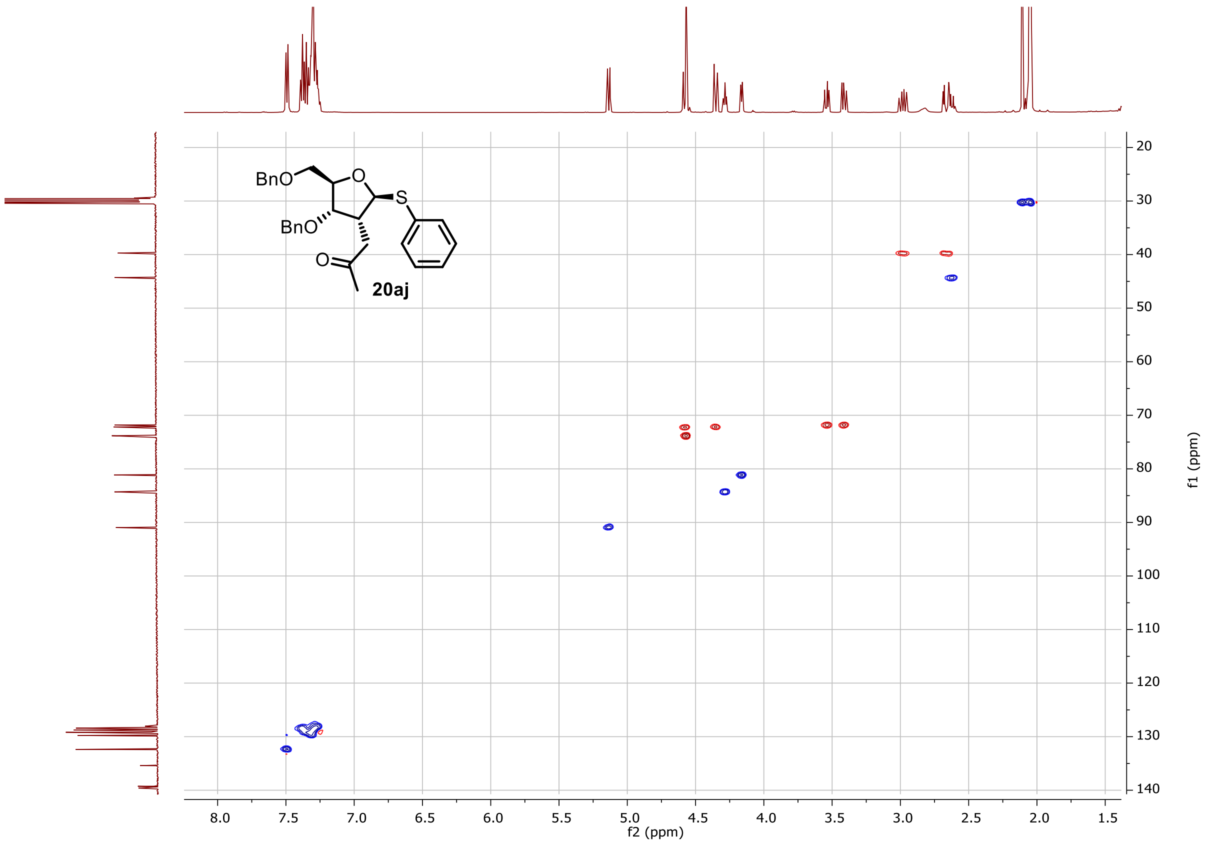

Supplementary Figure 276. HSQC spectra for **20aj**

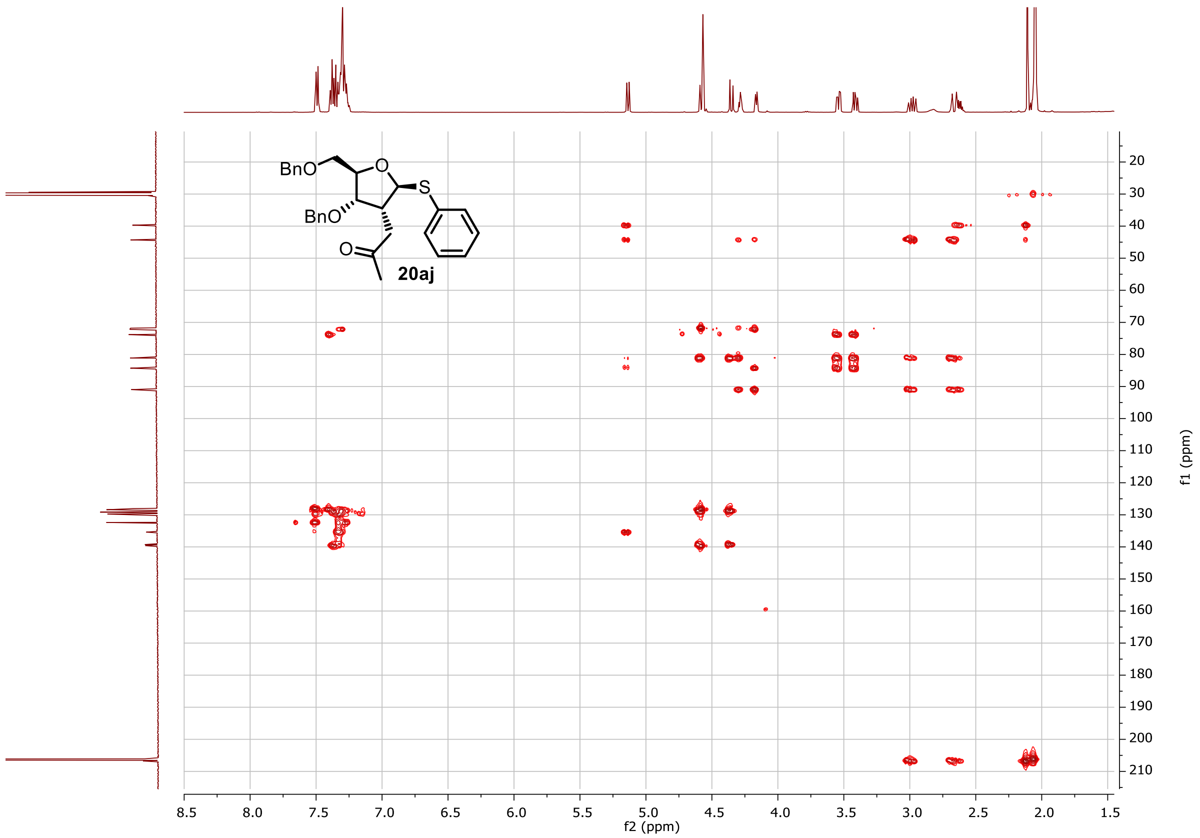

Supplementary Figure 277. HMBC spectra for **20aj**

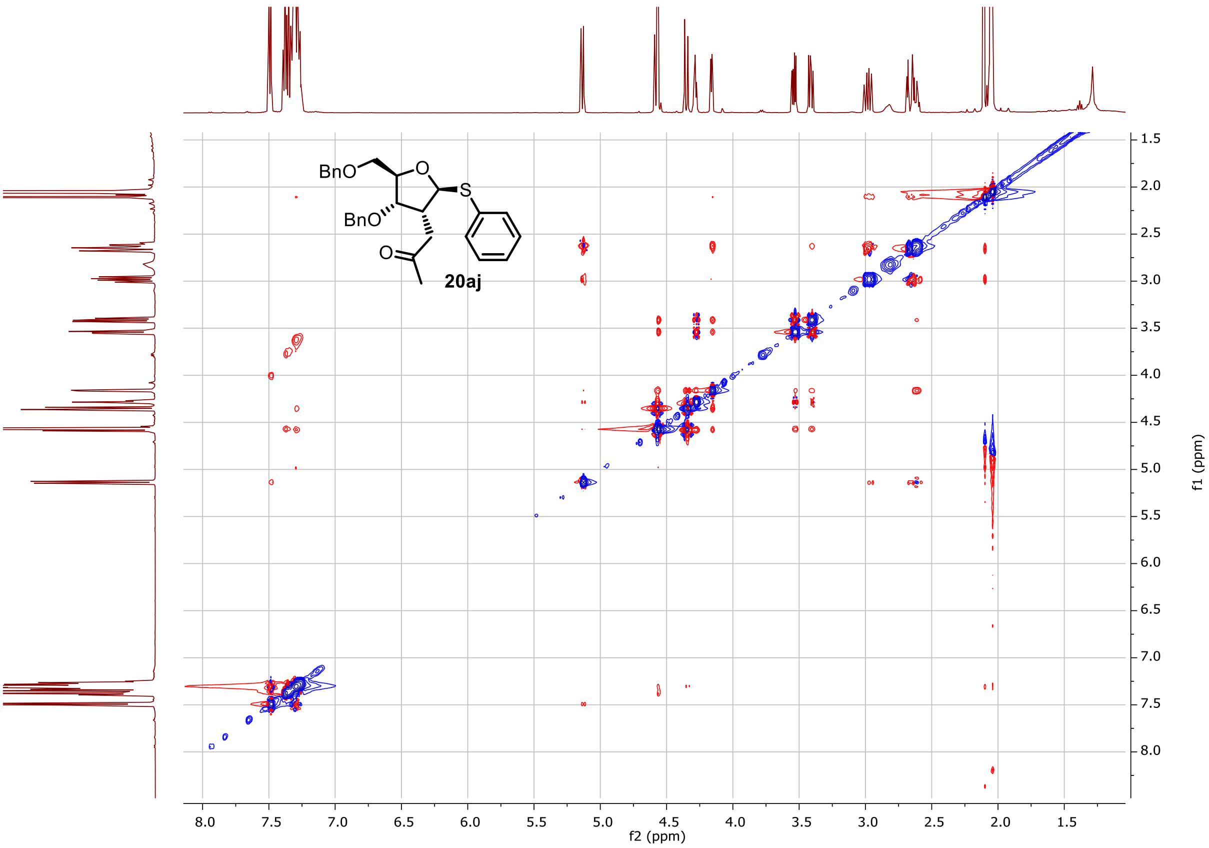

Supplementary Figure 278. NOESY spectra for **20aj**

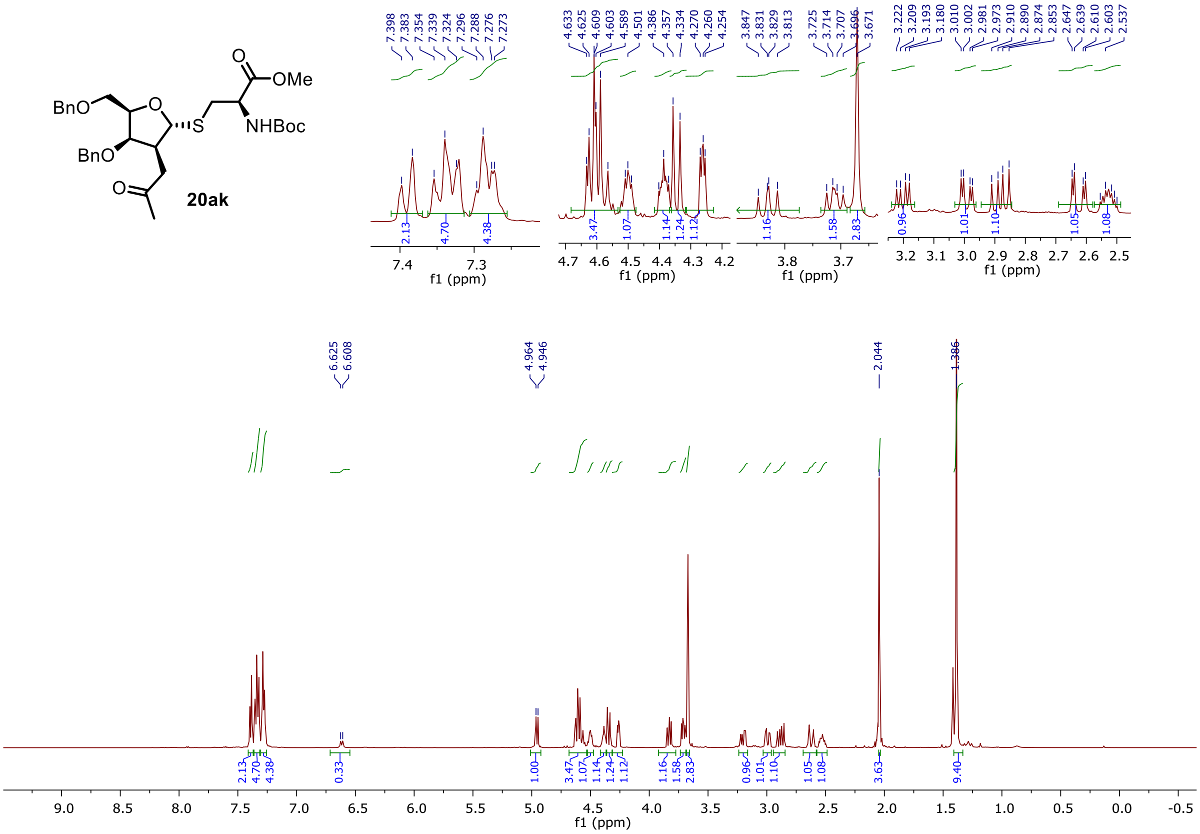

Supplementary Figure 279. ^{1}H spectra for **20ak**

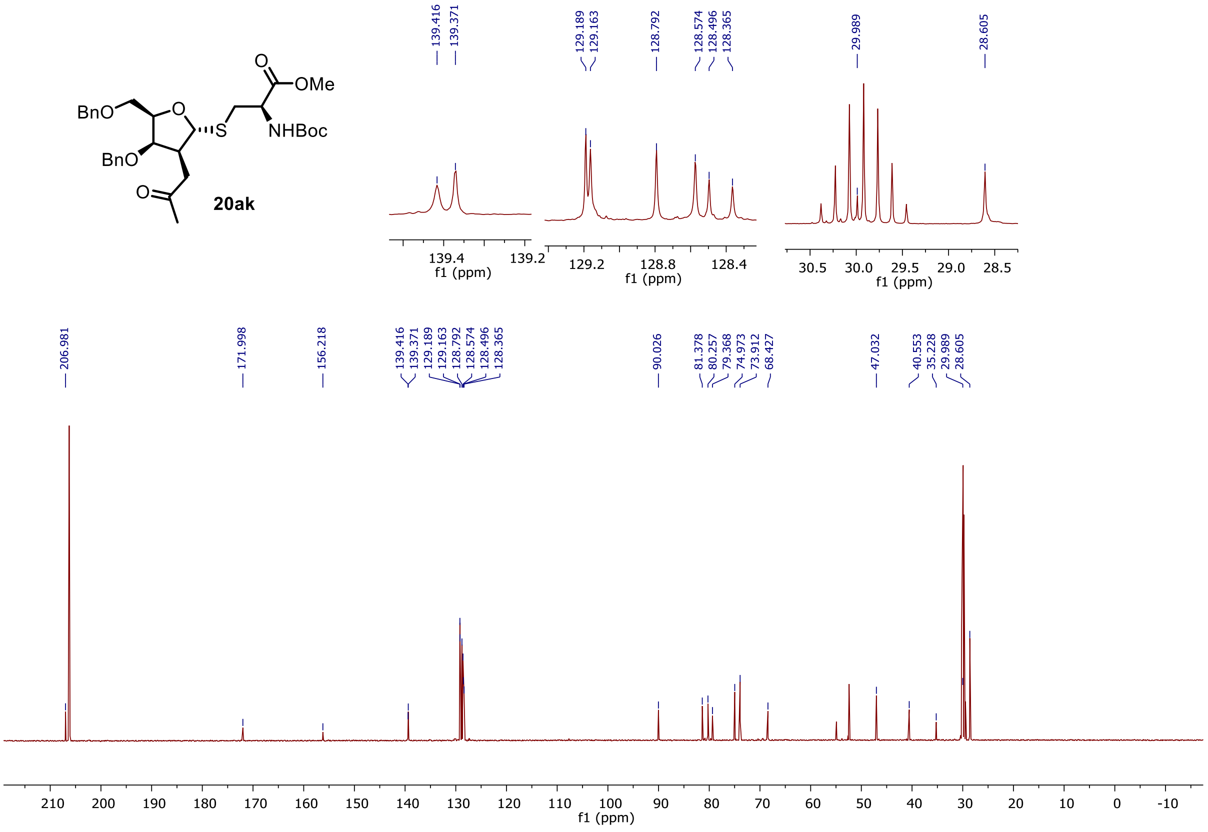

Supplementary Figure 280. ^{13}C spectra for **20ak**

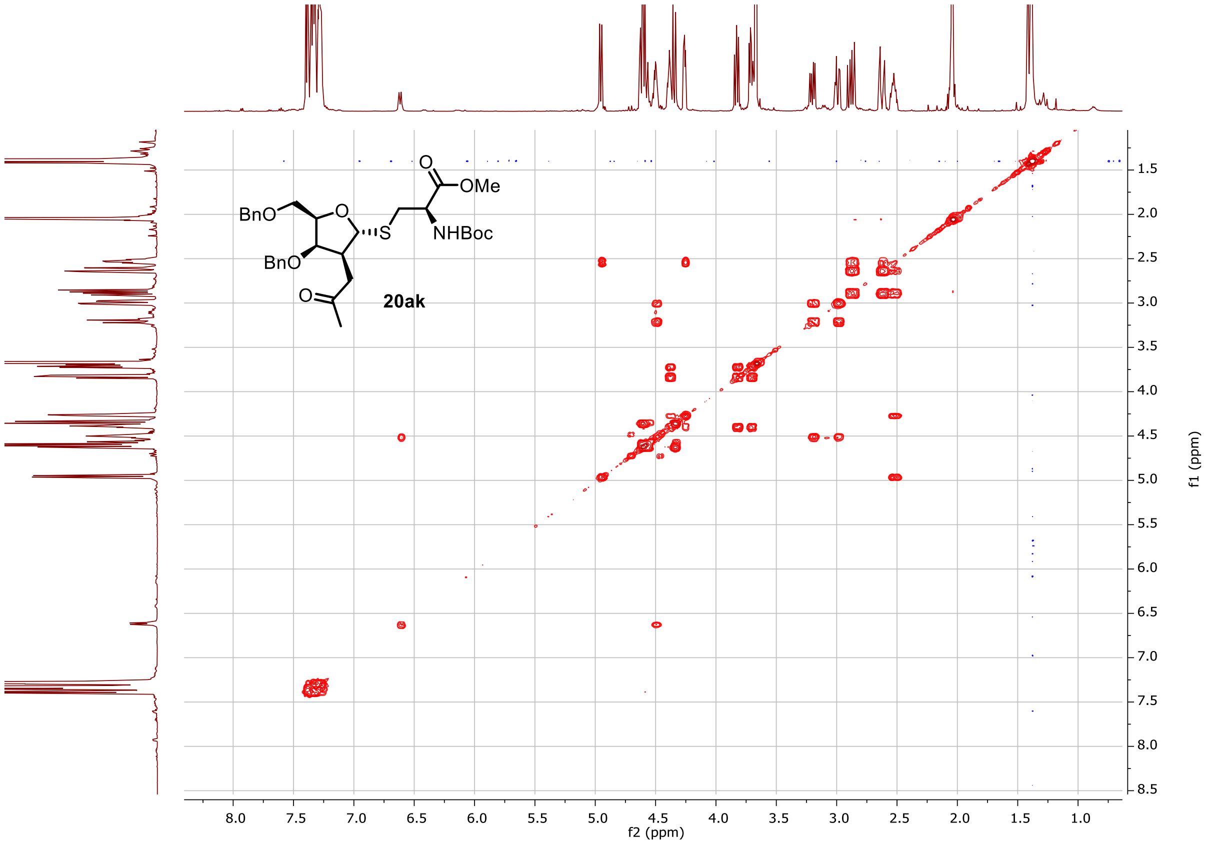

Supplementary Figure 281. COSY spectra for **20ak**

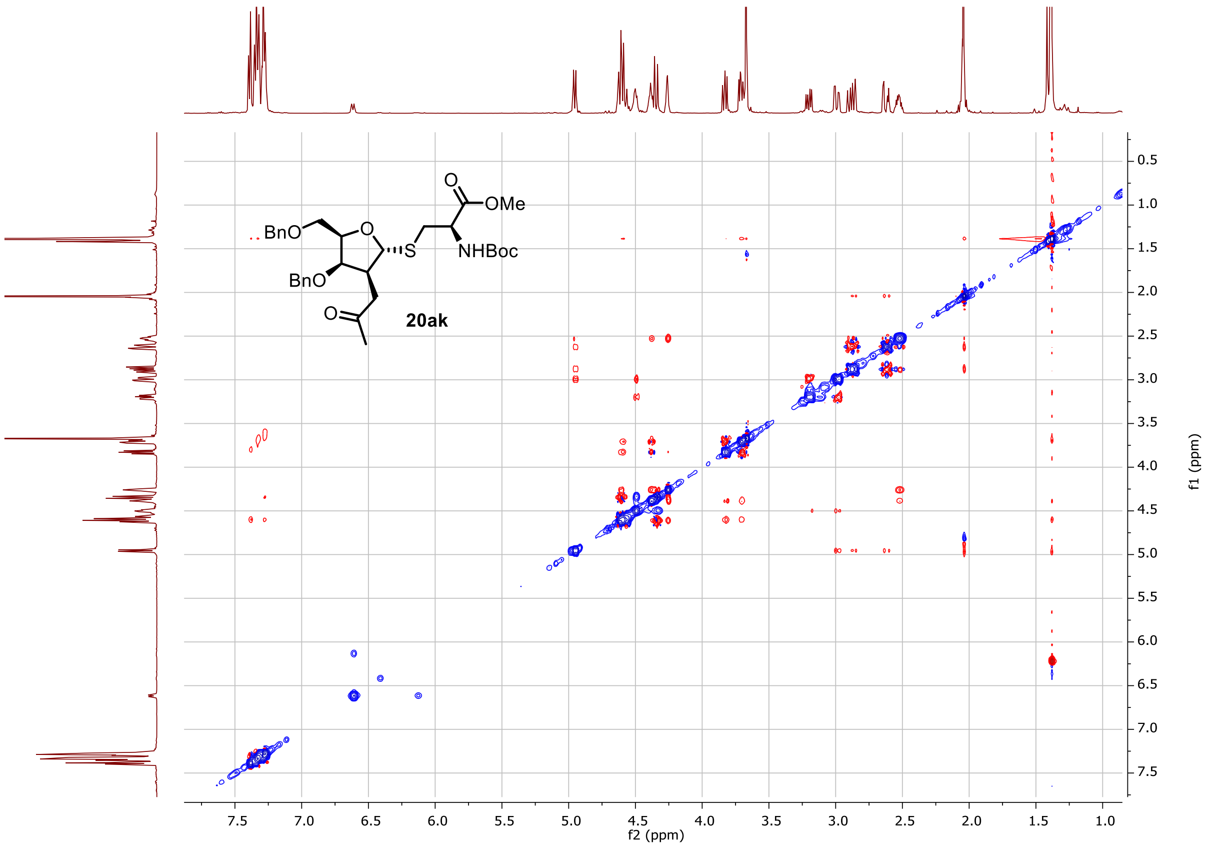

Supplementary Figure 282. NOESY spectra for **20ak**

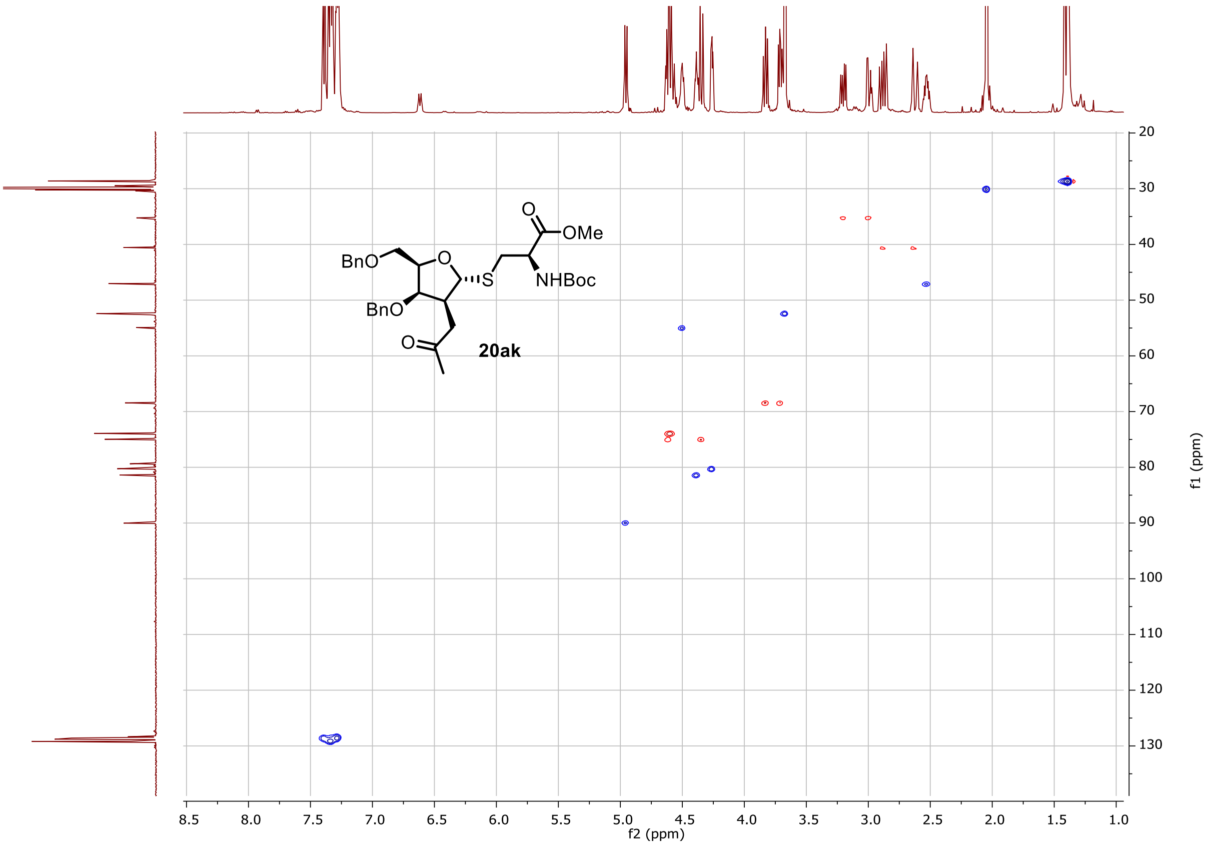

Supplementary Figure 283. HSQC spectra for **20ak**

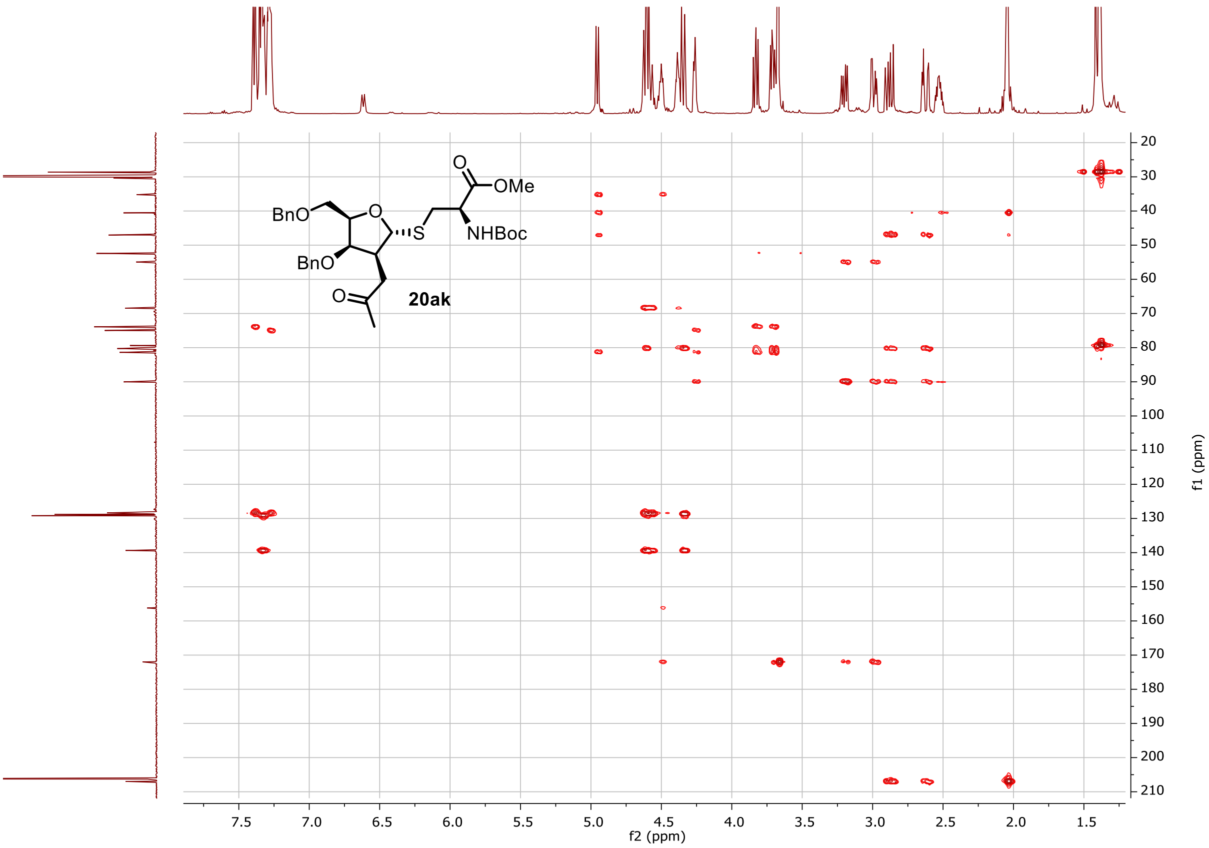

Supplementary Figure 284. HMBC spectra for **20ak**

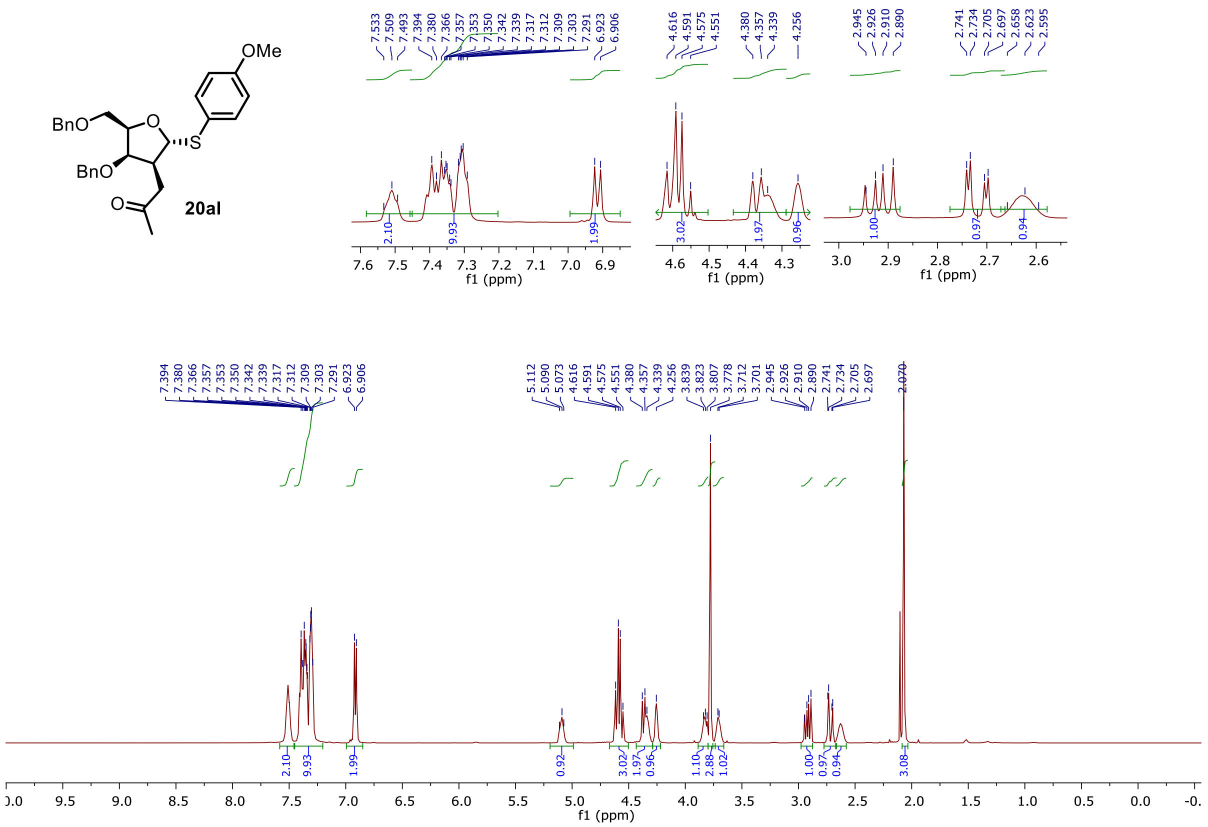

Supplementary Figure 285. ^{1}H spectra for **20al**

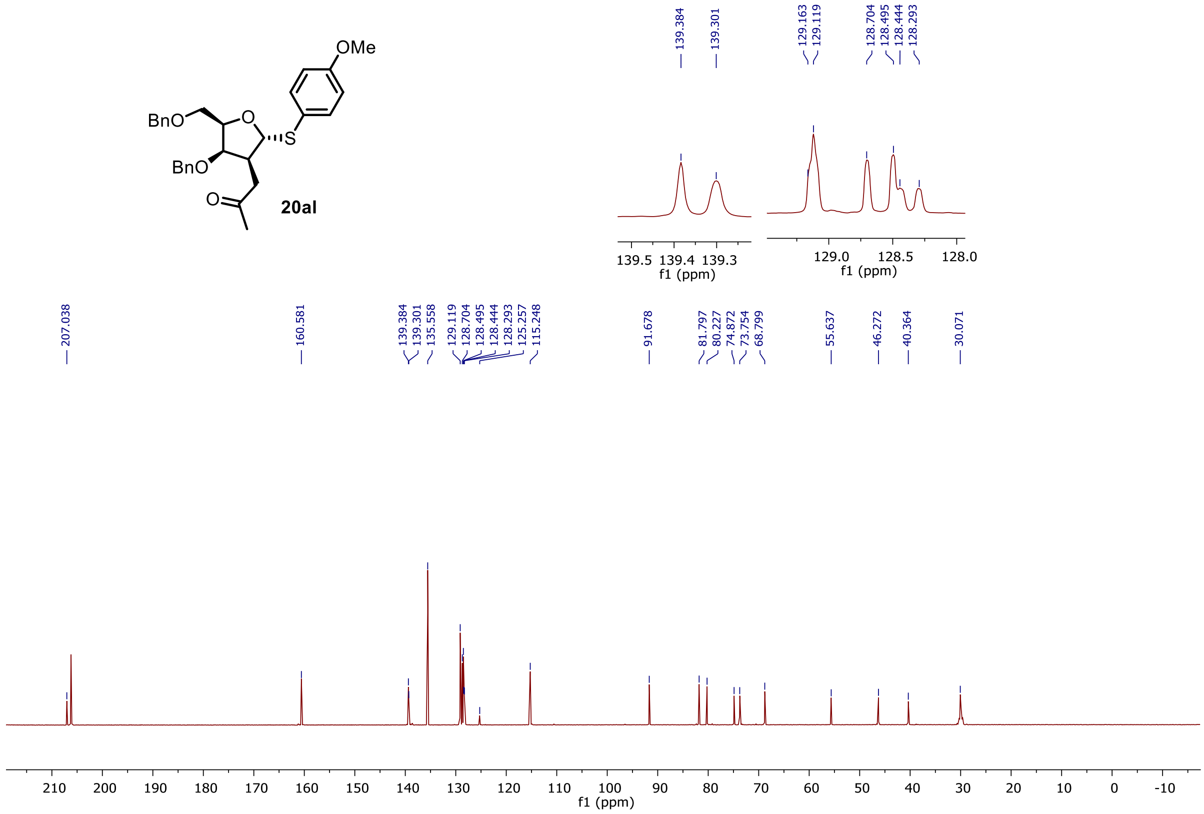

Supplementary Figure 286. ^{13}C spectra for **20al**

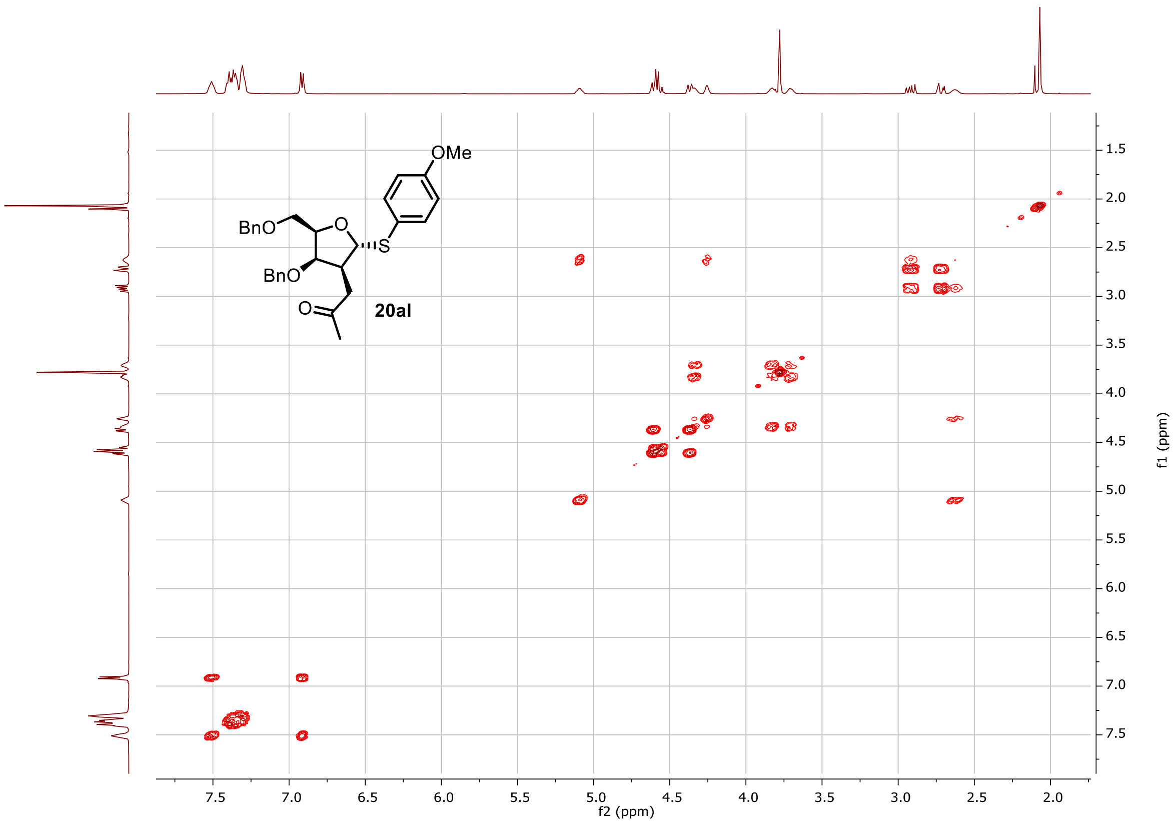

Supplementary Figure 287. COSY spectra for **20al**

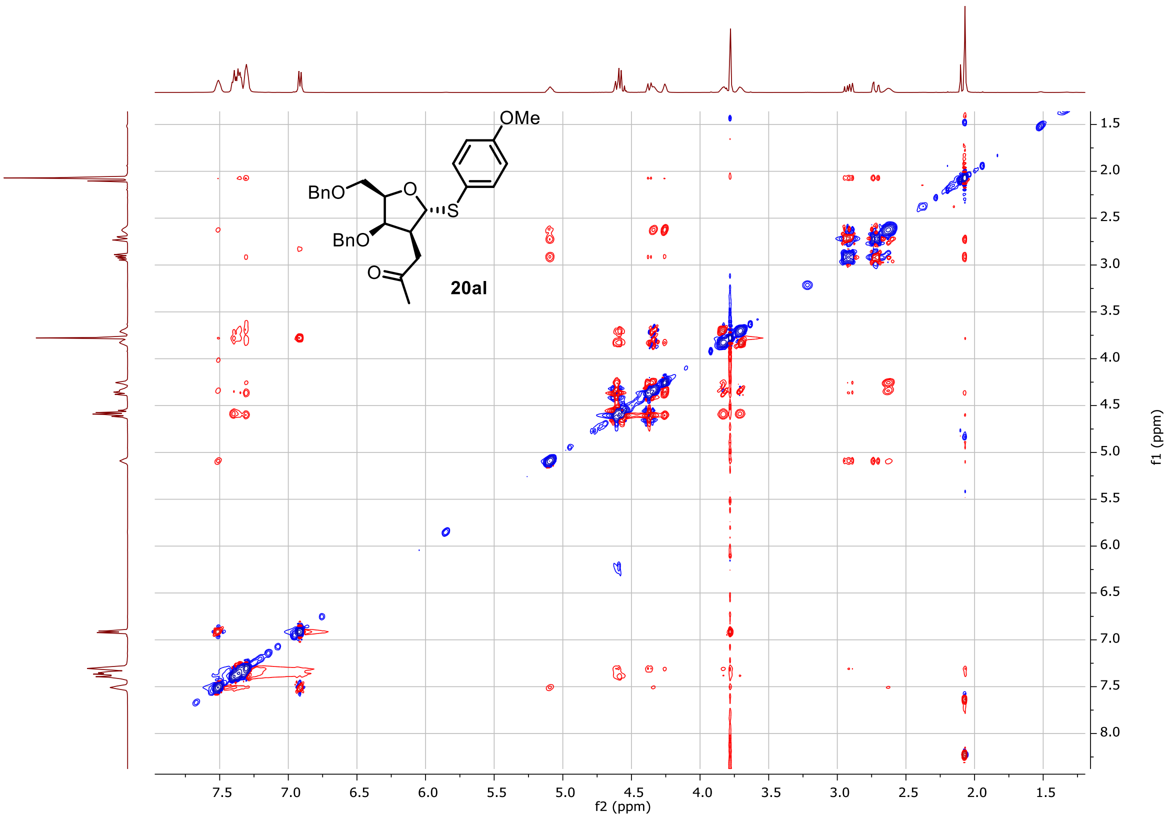

Supplementary Figure 288. NOESY spectra for **20al**

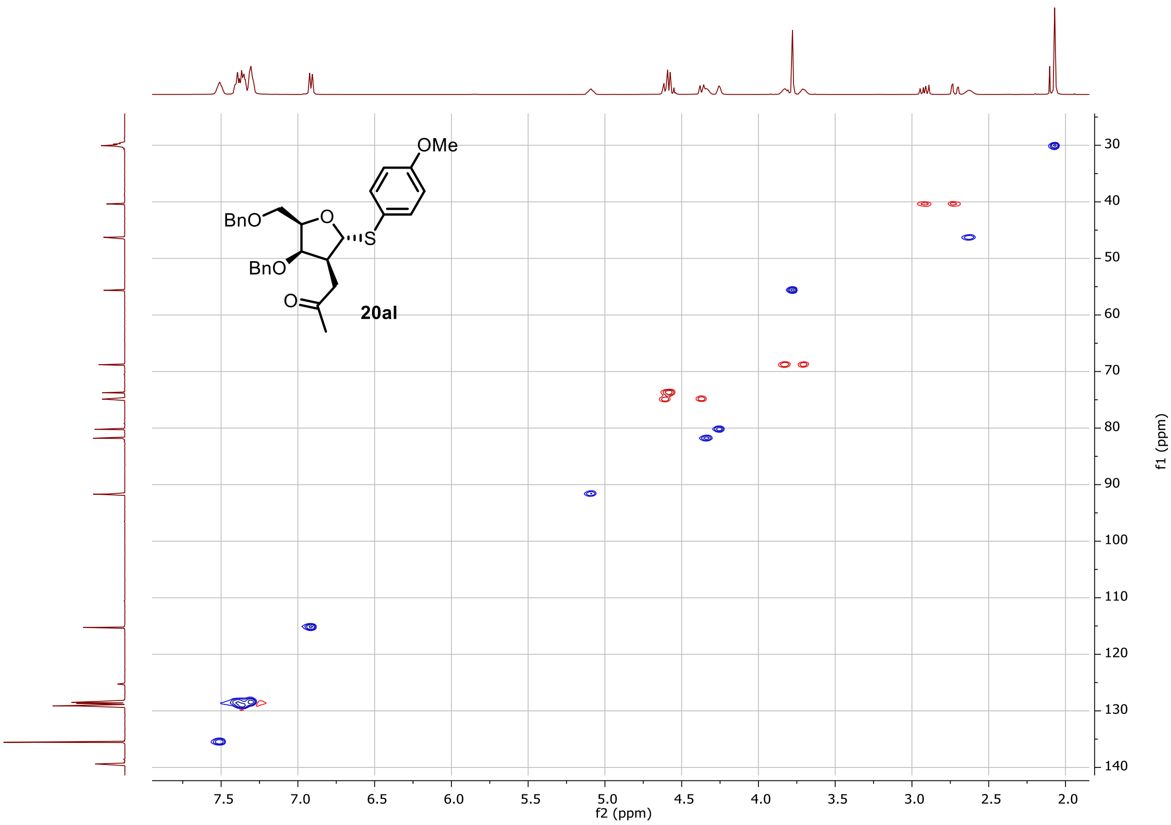

Supplementary Figure 289. HSQC spectra for **20al**

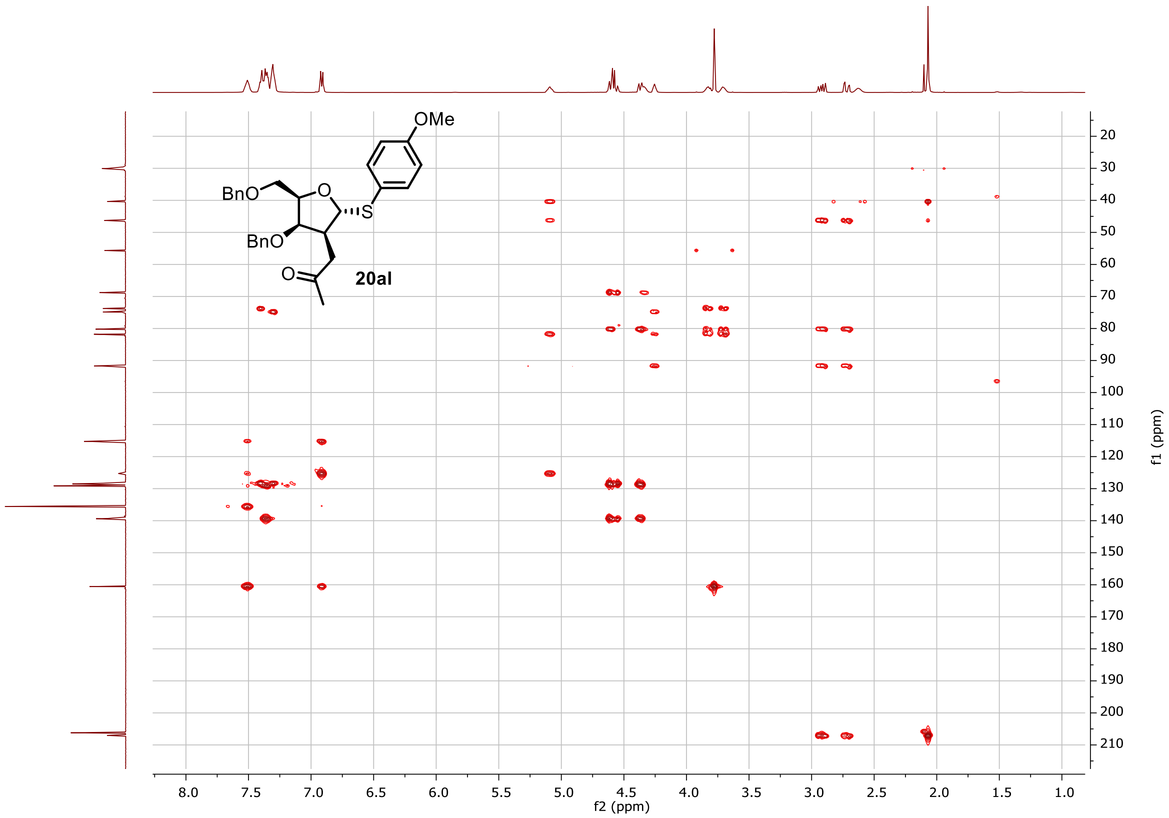

Supplementary Figure 290. HMBC spectra for **20al**

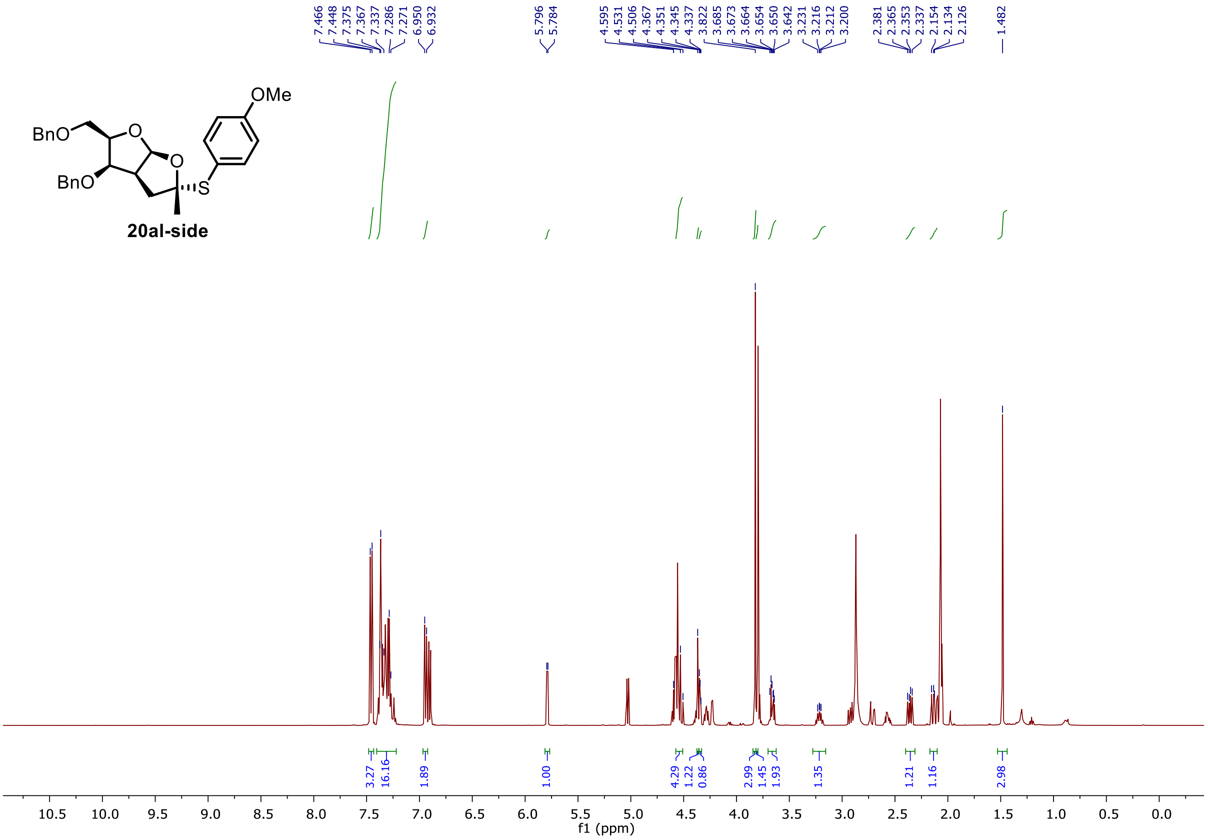

Supplementary Figure 291. ^{1}H spectra for **20al-side** and **20al**

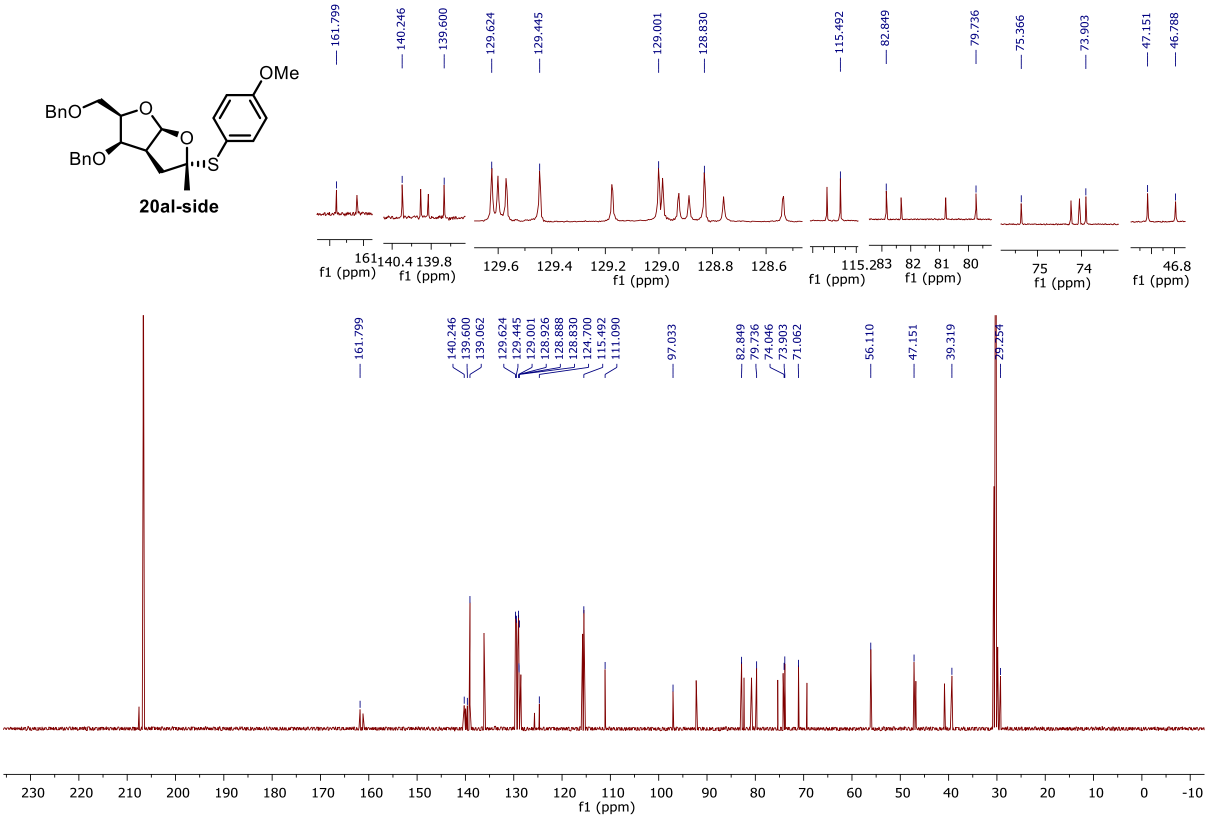

Supplementary Figure 292. ^{13}C spectra for **20al-side** and **20al**

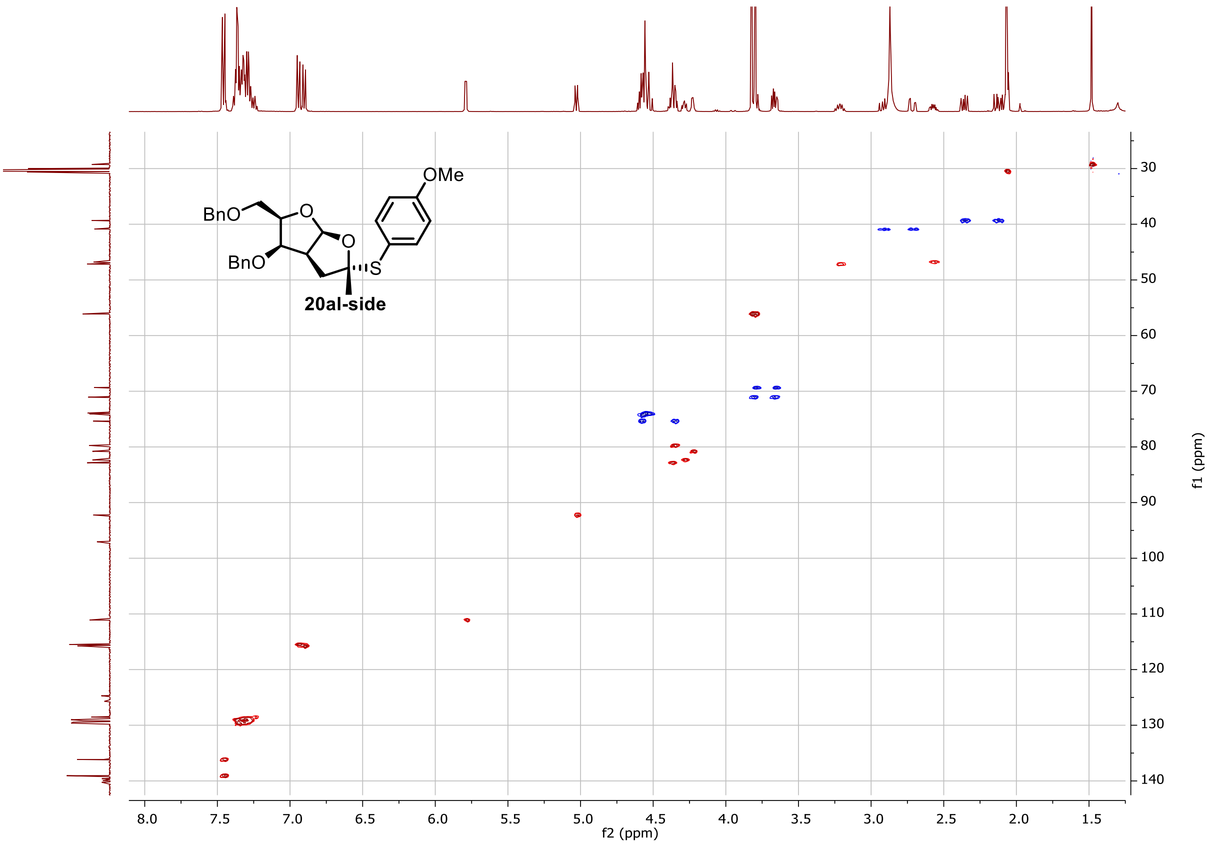

Supplementary Figure 293. HSQC spectra for **20al-side** and **20al**

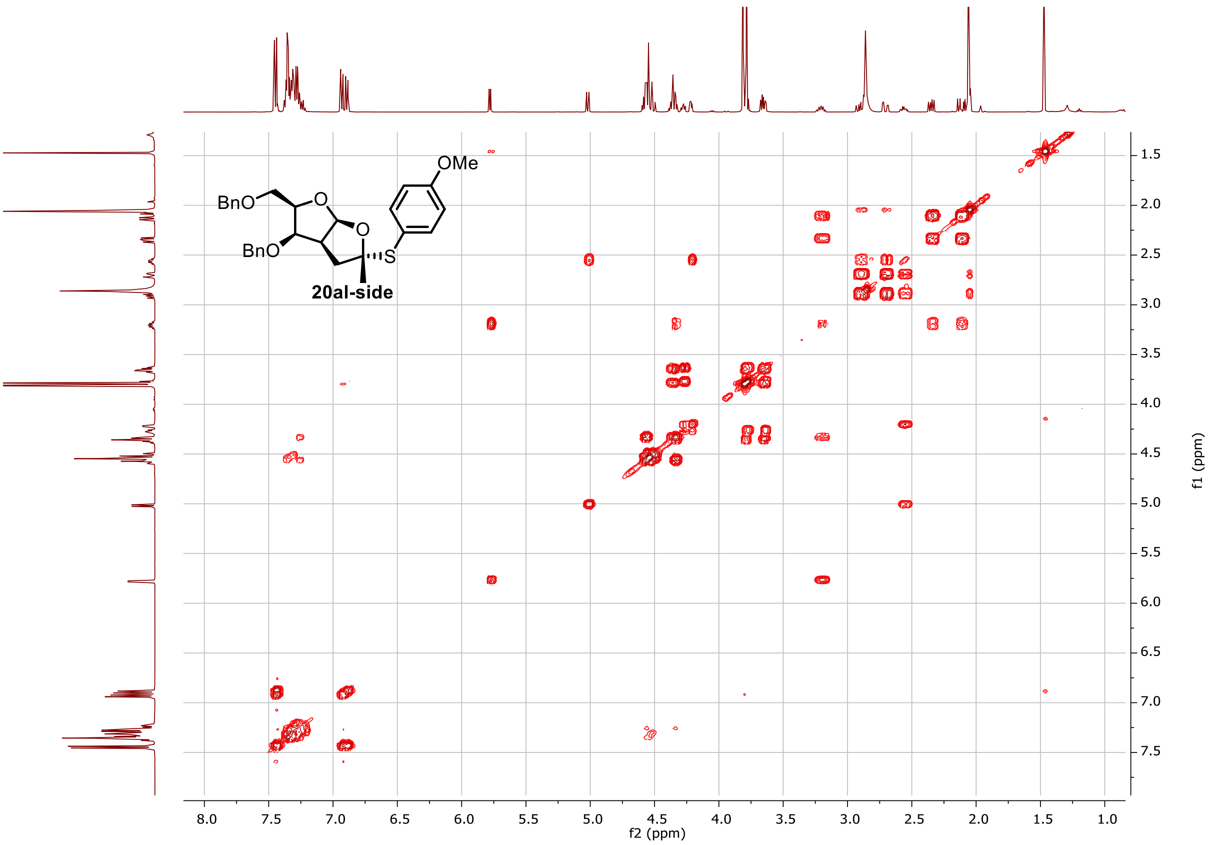

Supplementary Figure 294. COSY spectra for **20al-side** and **20al**

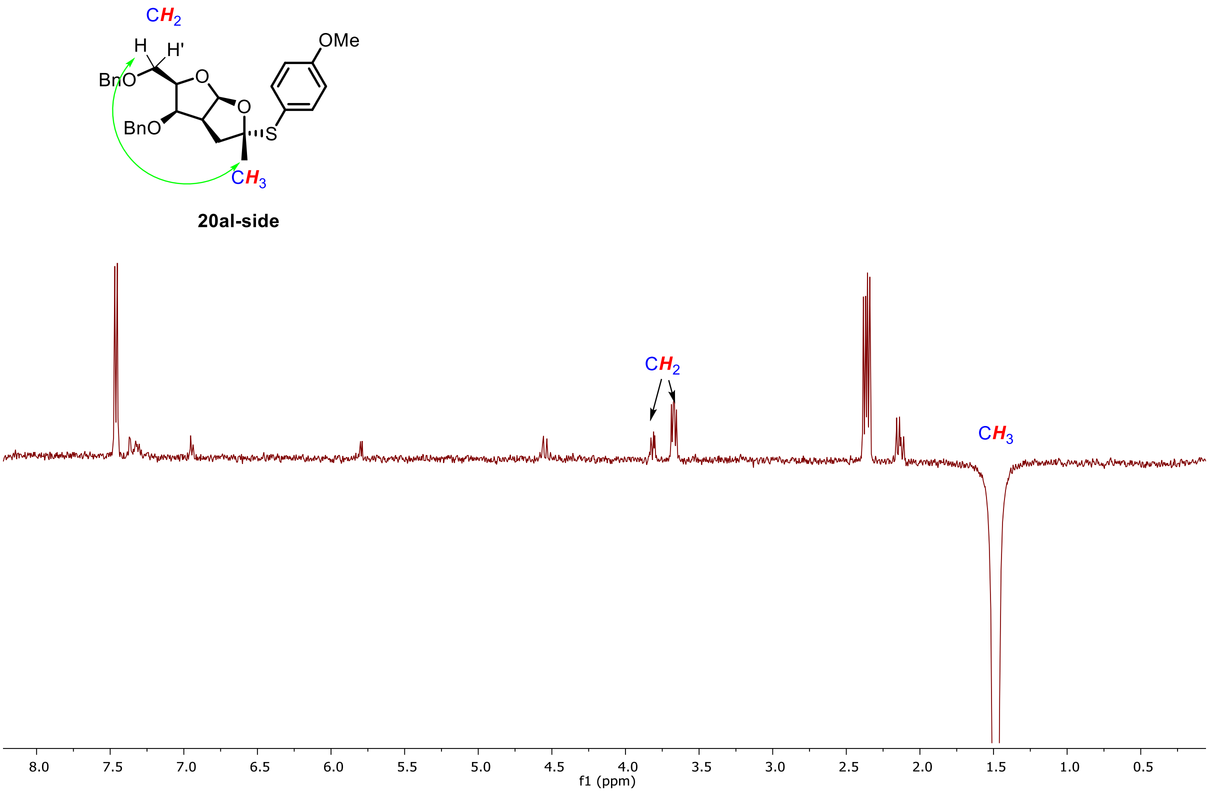

Supplementary Figure 295. 1D NOE spectra for **20al-side** and **20al**

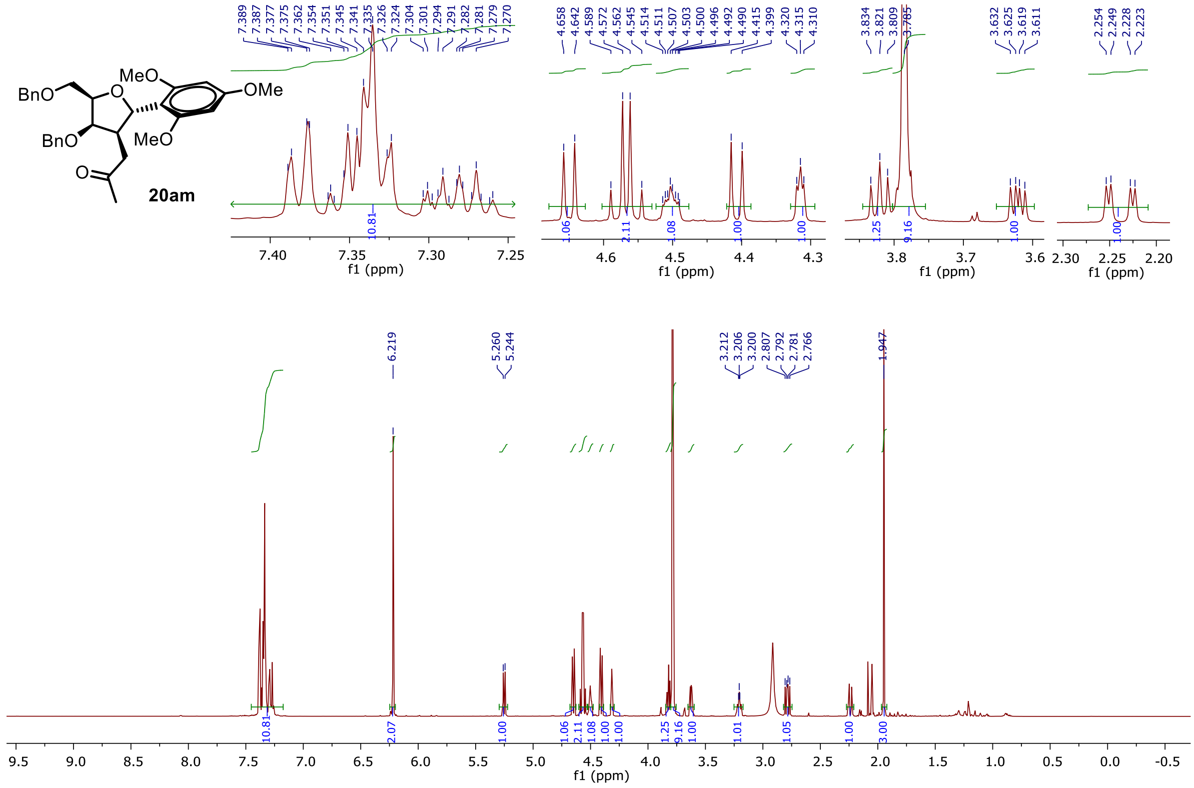

Supplementary Figure 296. ^{1}H spectra for **20am**

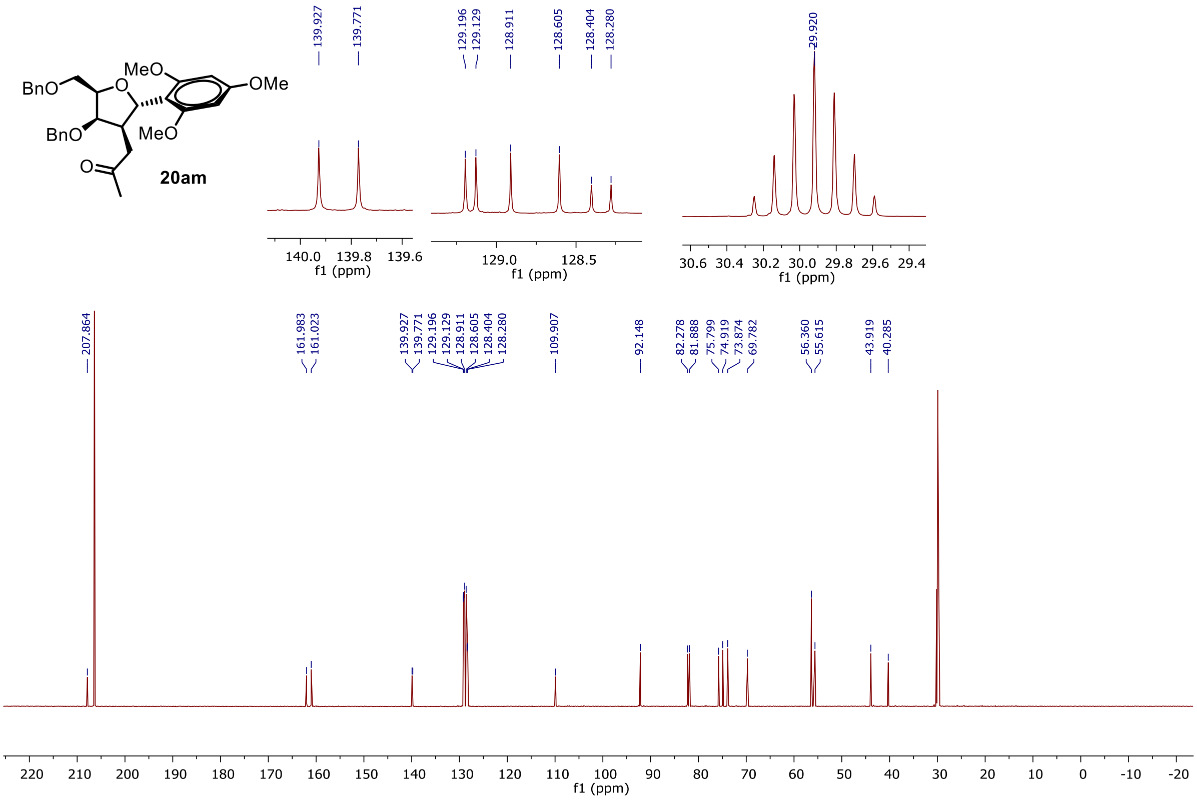

Supplementary Figure 297. ^{13}C spectra for **20am**

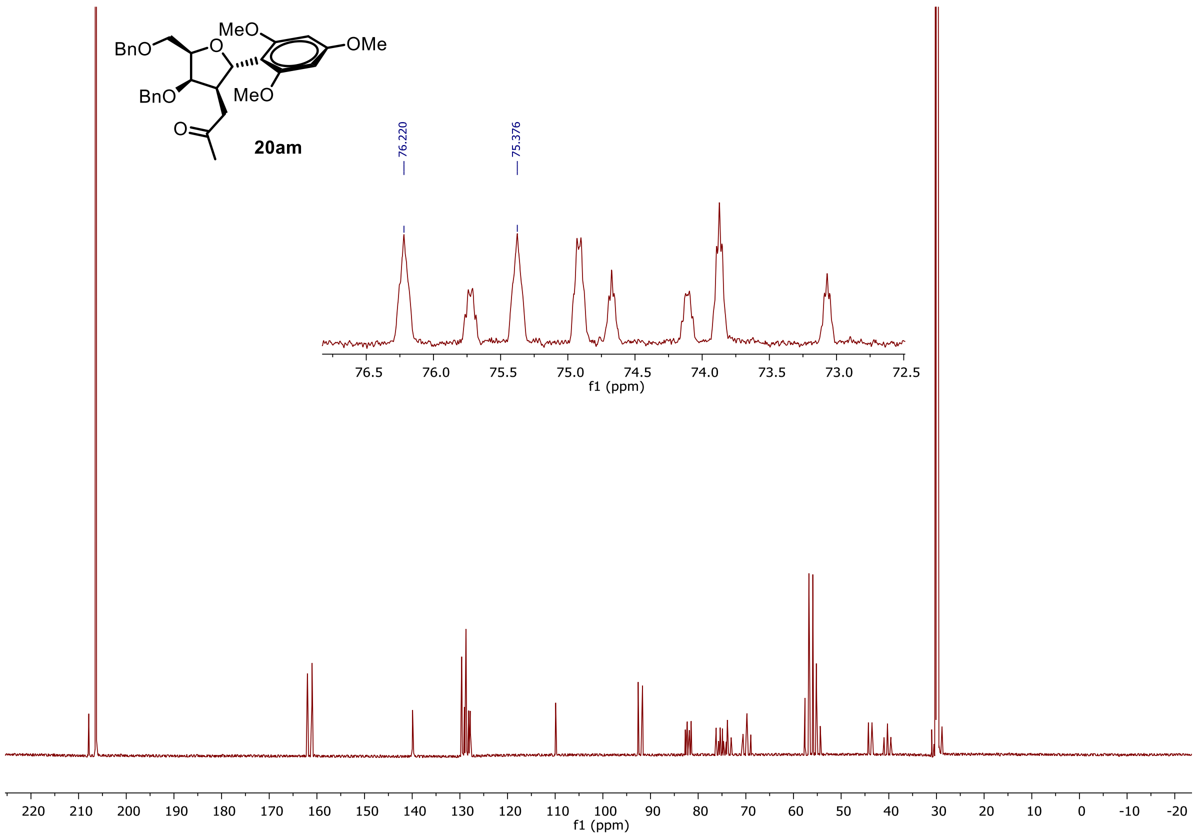

Supplementary Figure 298. Gated ^{13}C (with coupling of proton) spectra for **20am**

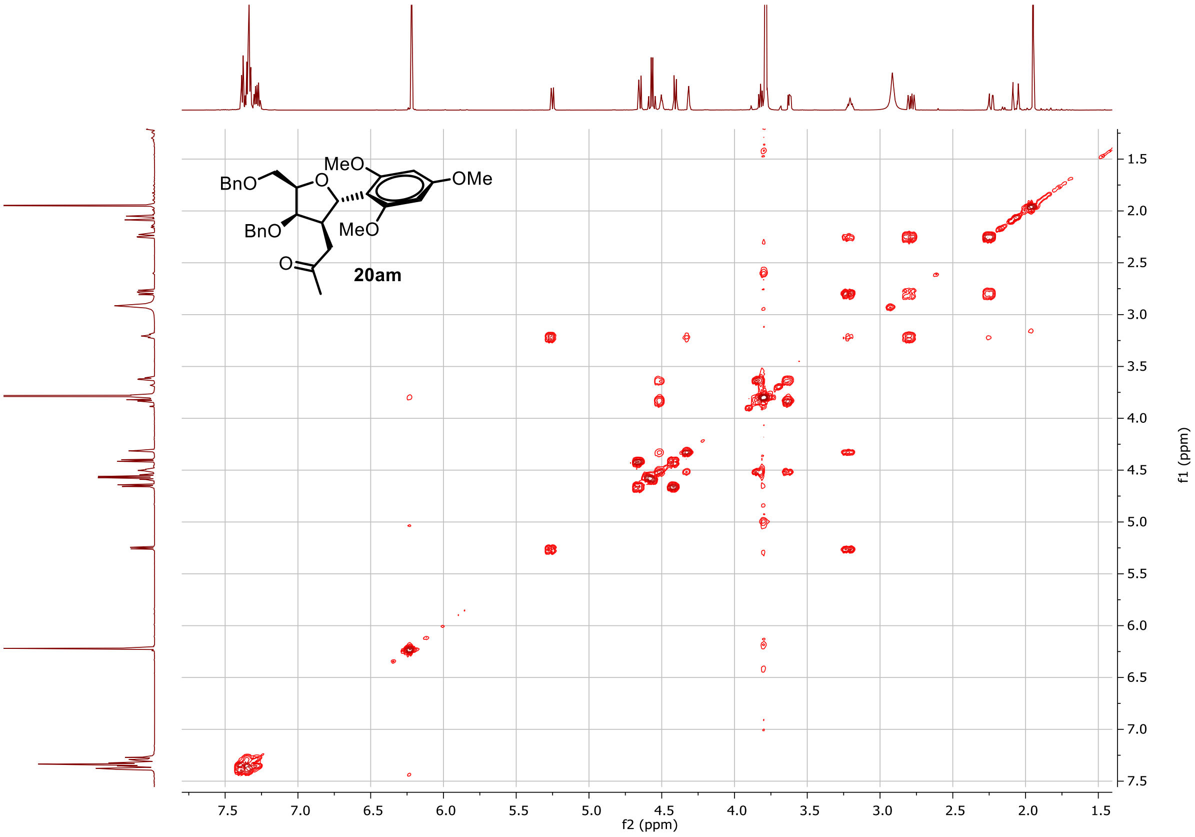

Supplementary Figure 299. COSY spectra for **20am**

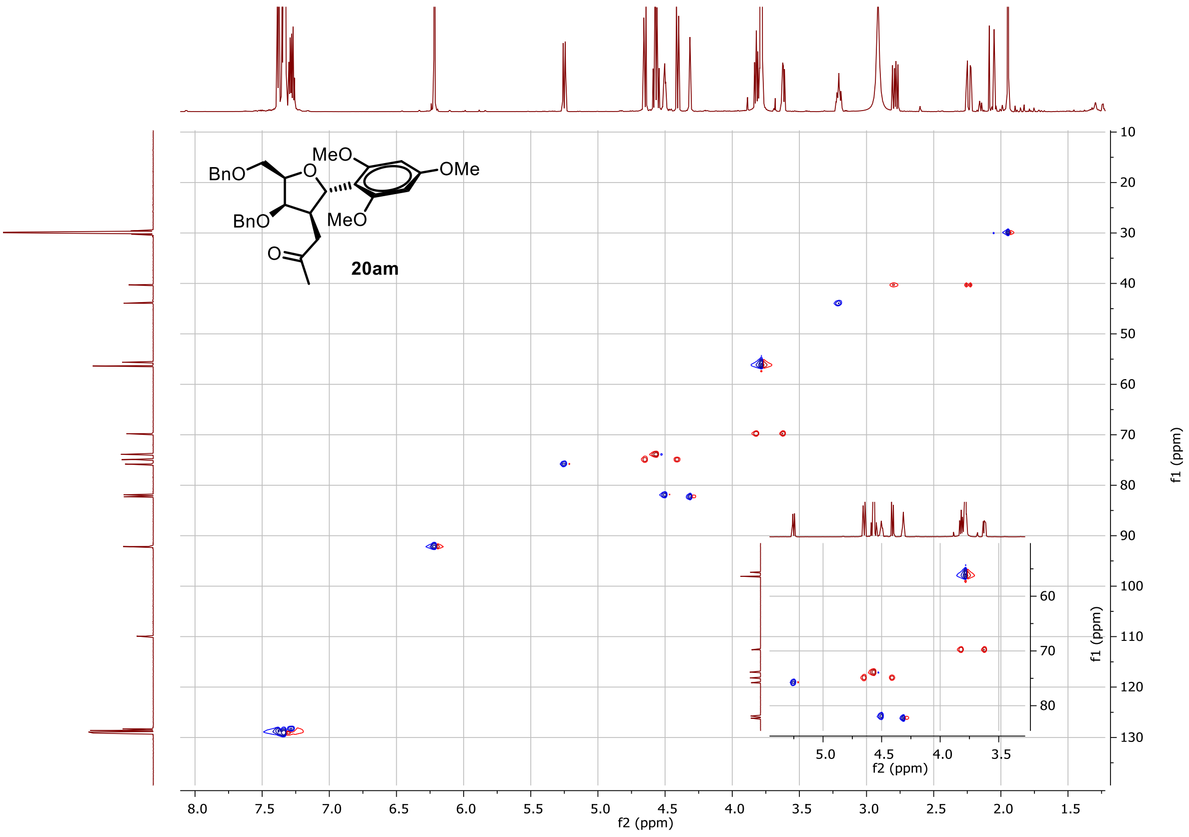

Supplementary Figure 300. HSQC spectra for **20am**

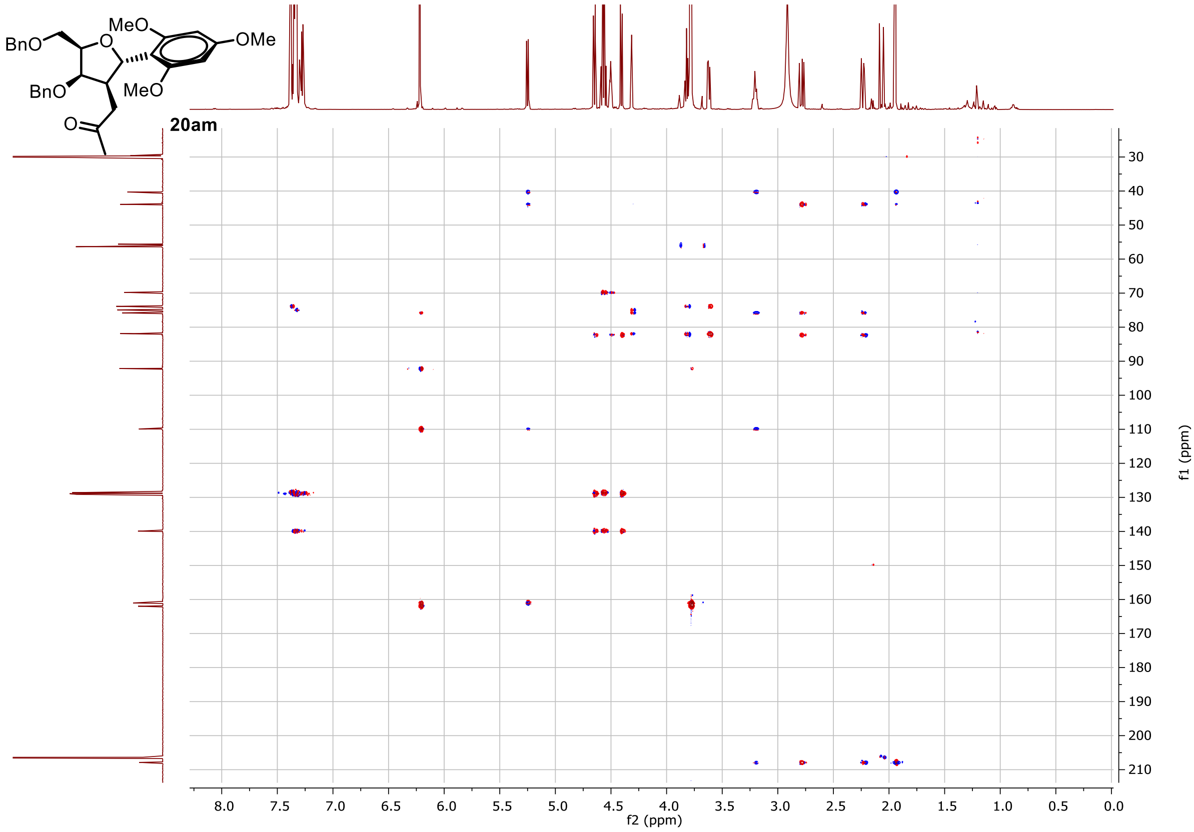

Supplementary Figure 301. HMBC spectra for **20am**

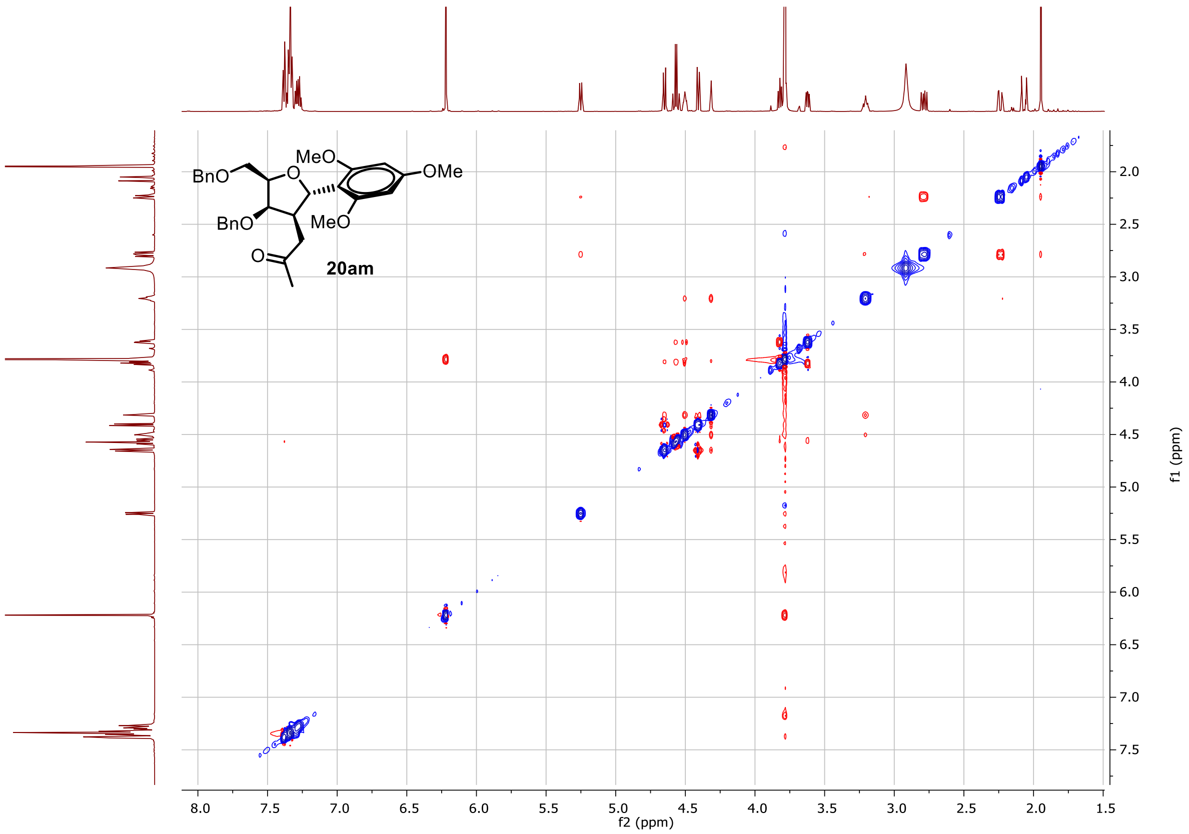

Supplementary Figure 302. NOESY spectra for **20am**

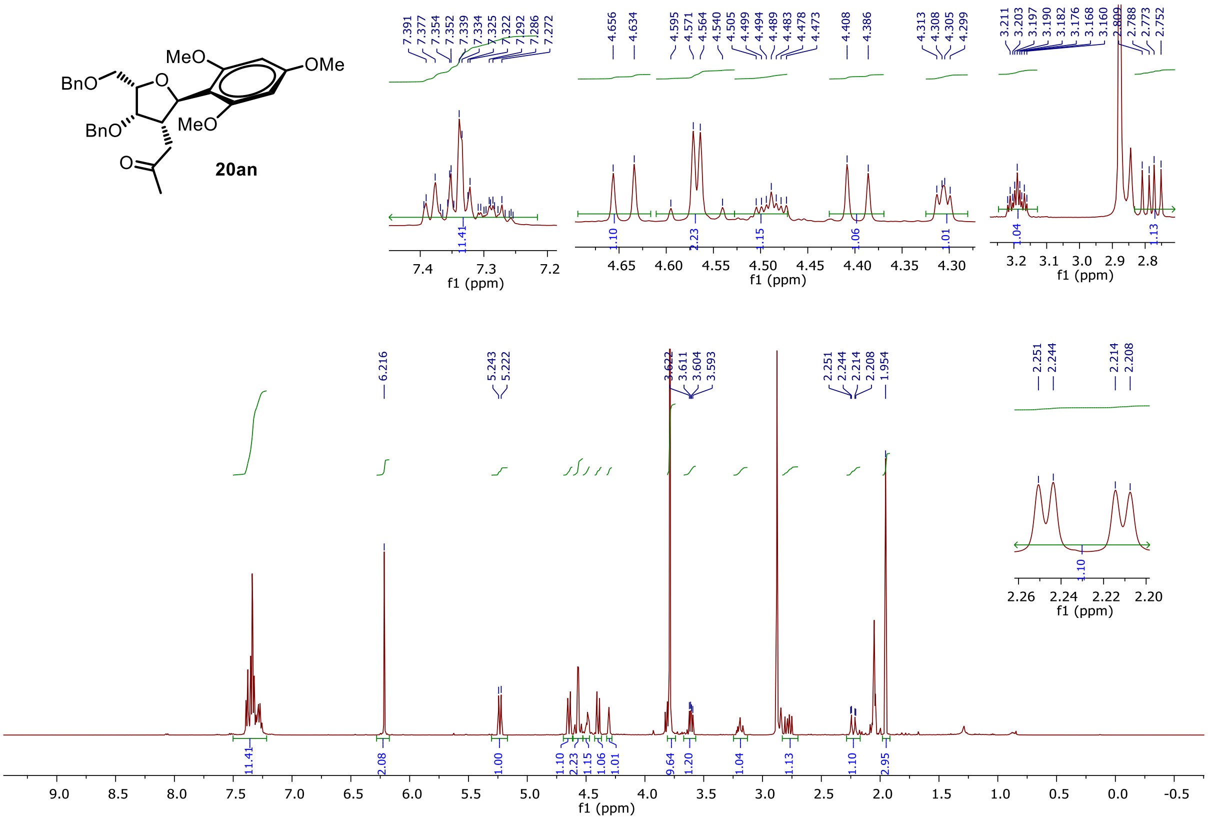

Supplementary Figure 303. ^{1}H spectra for **20an**

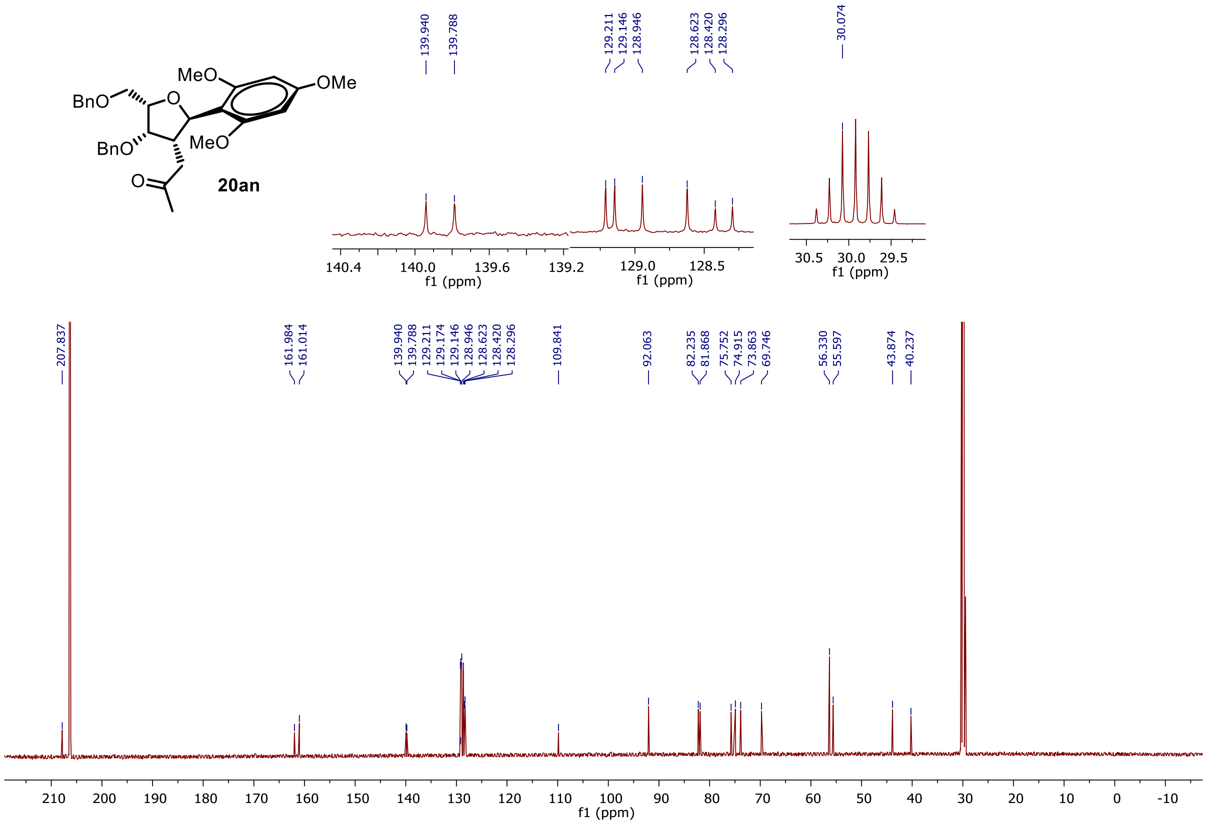

Supplementary Figure 304. ^{13}C spectra for **20an**

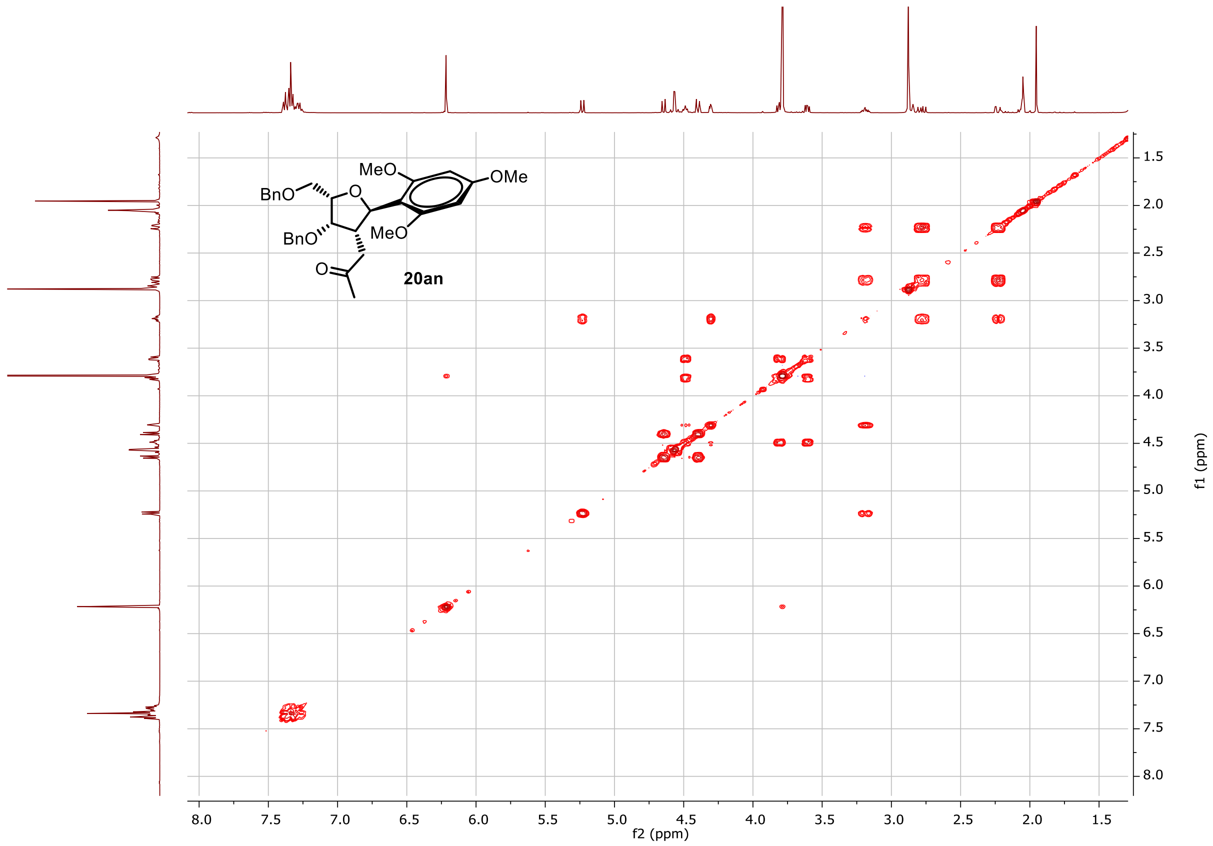

Supplementary Figure 305. COSY spectra for **20an**

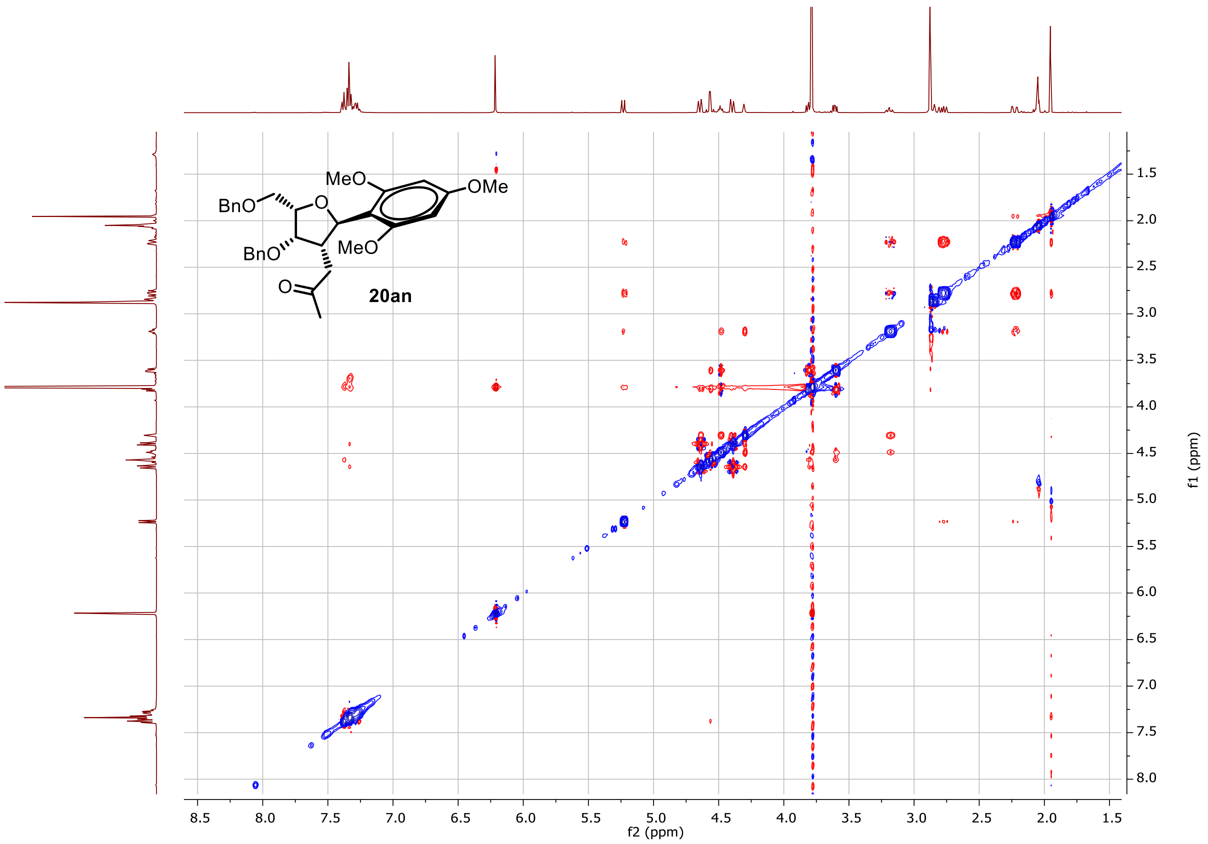

Supplementary Figure 306. NOESY spectra for **20an**

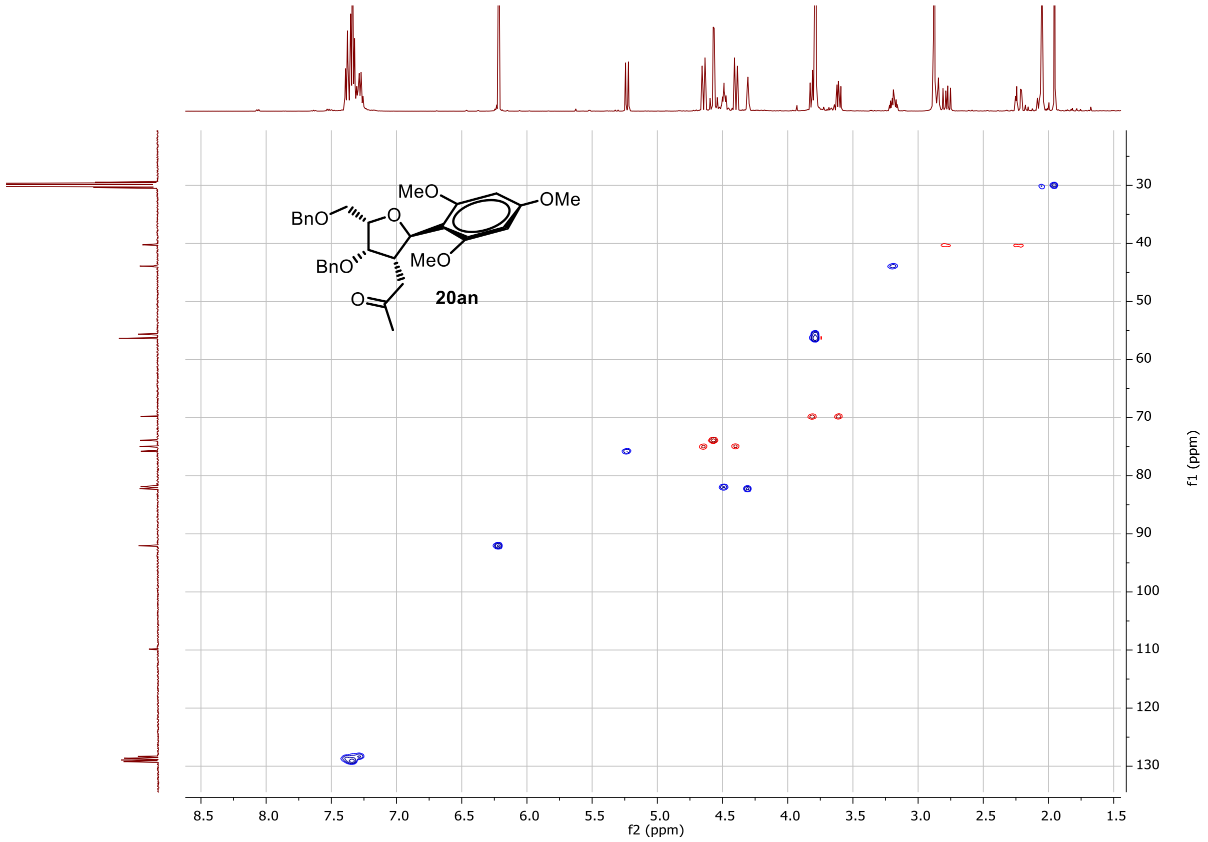

Supplementary Figure 307. HSQC spectra for **20an**

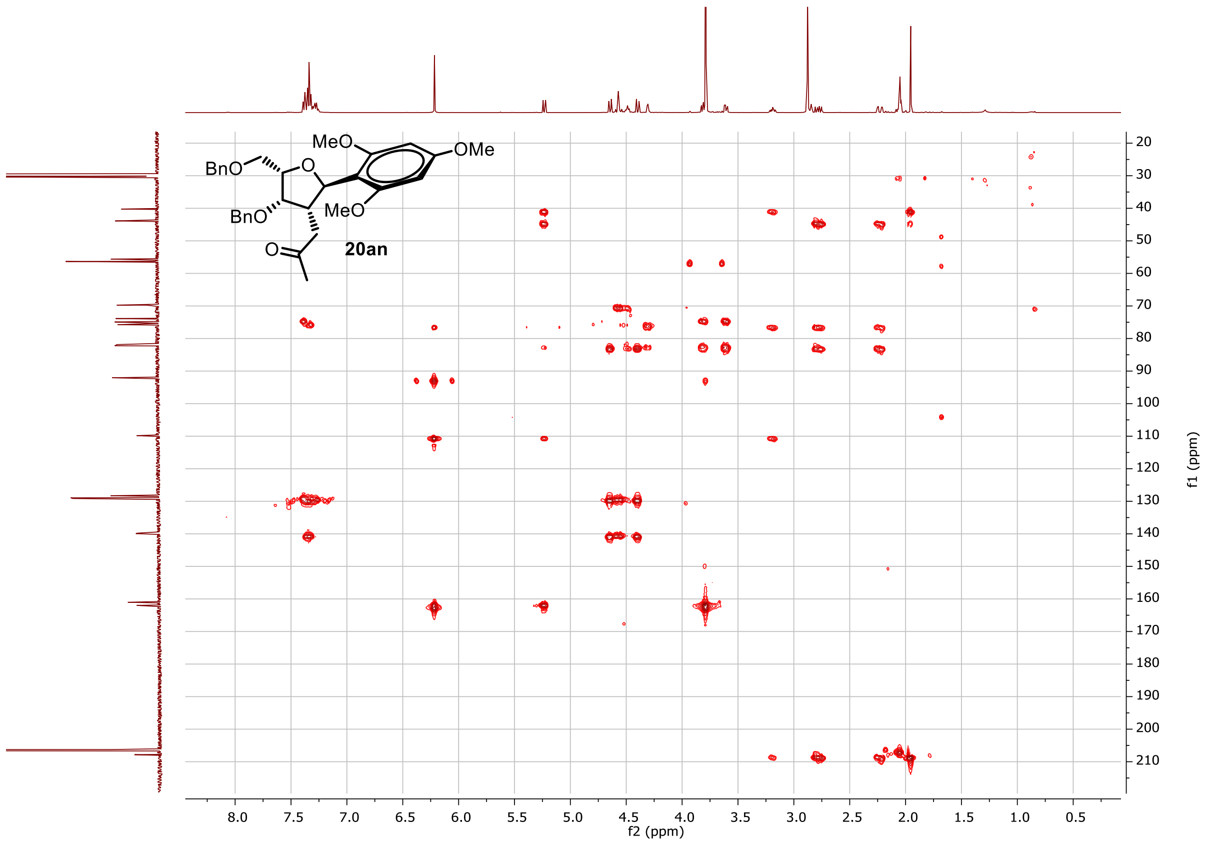

Supplementary Figure 308. HMBC spectra for **20an**

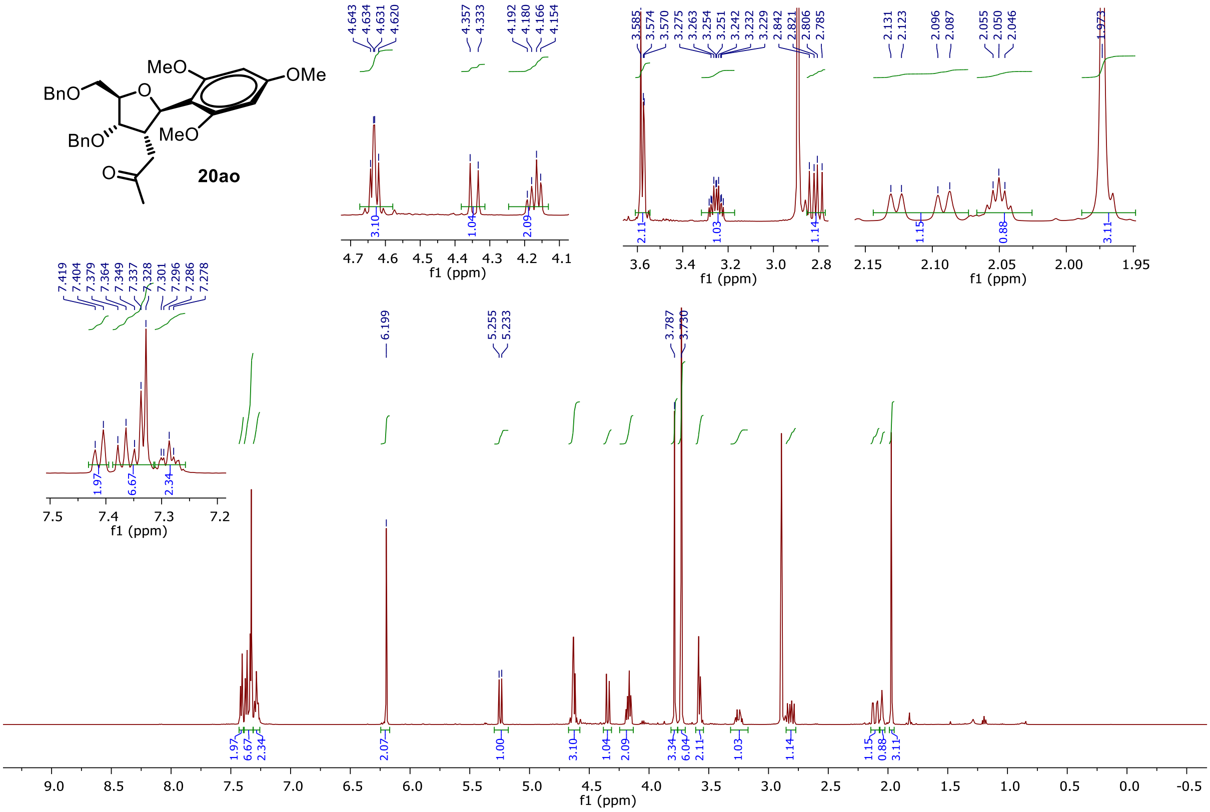

Supplementary Figure 309. ^{1}H spectra for **20ao**

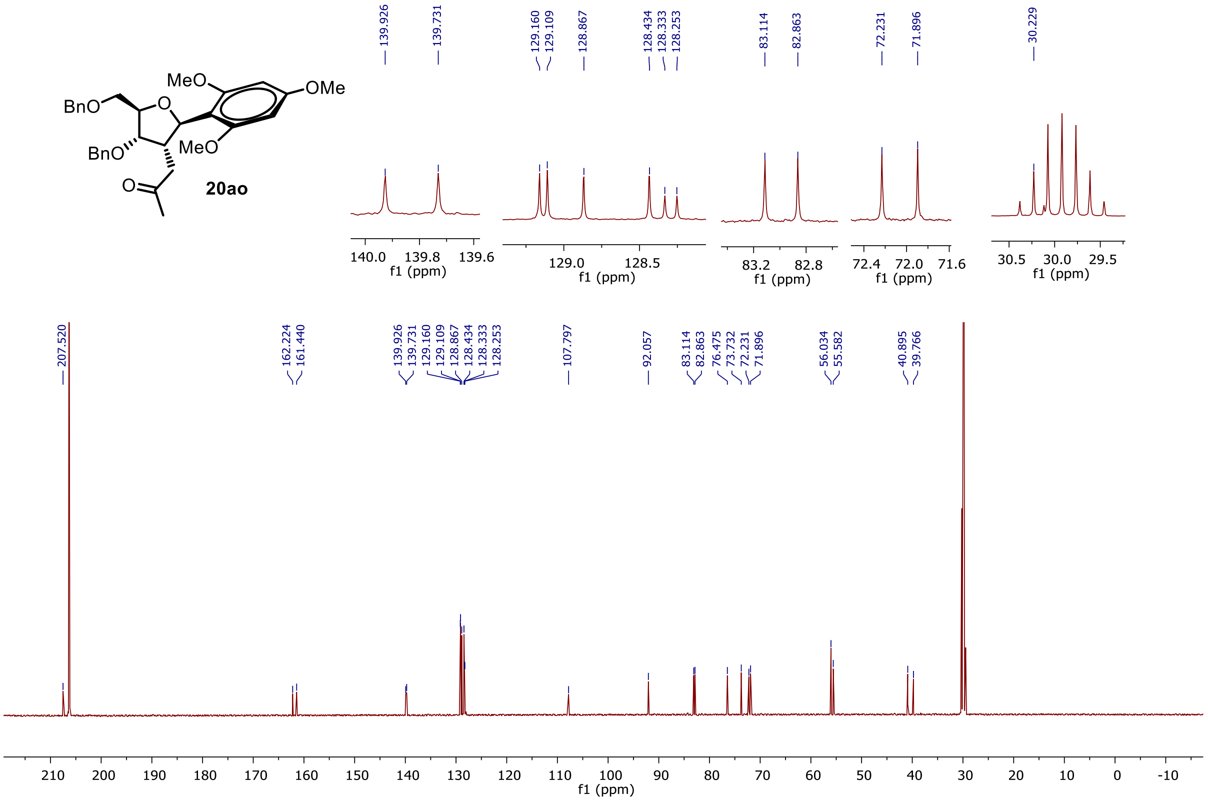

Supplementary Figure 310. ^{13}C spectra for **20ao**

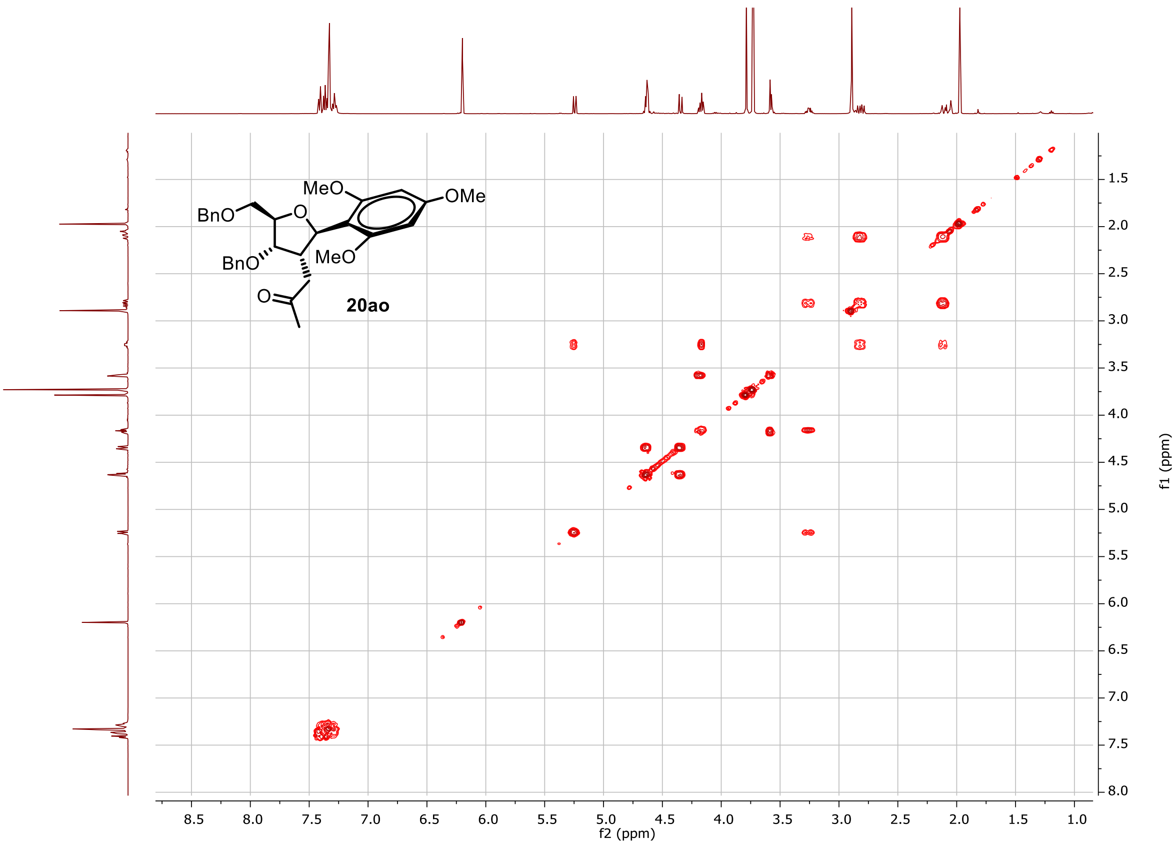

Supplementary Figure 311. COSY spectra for **20ao**

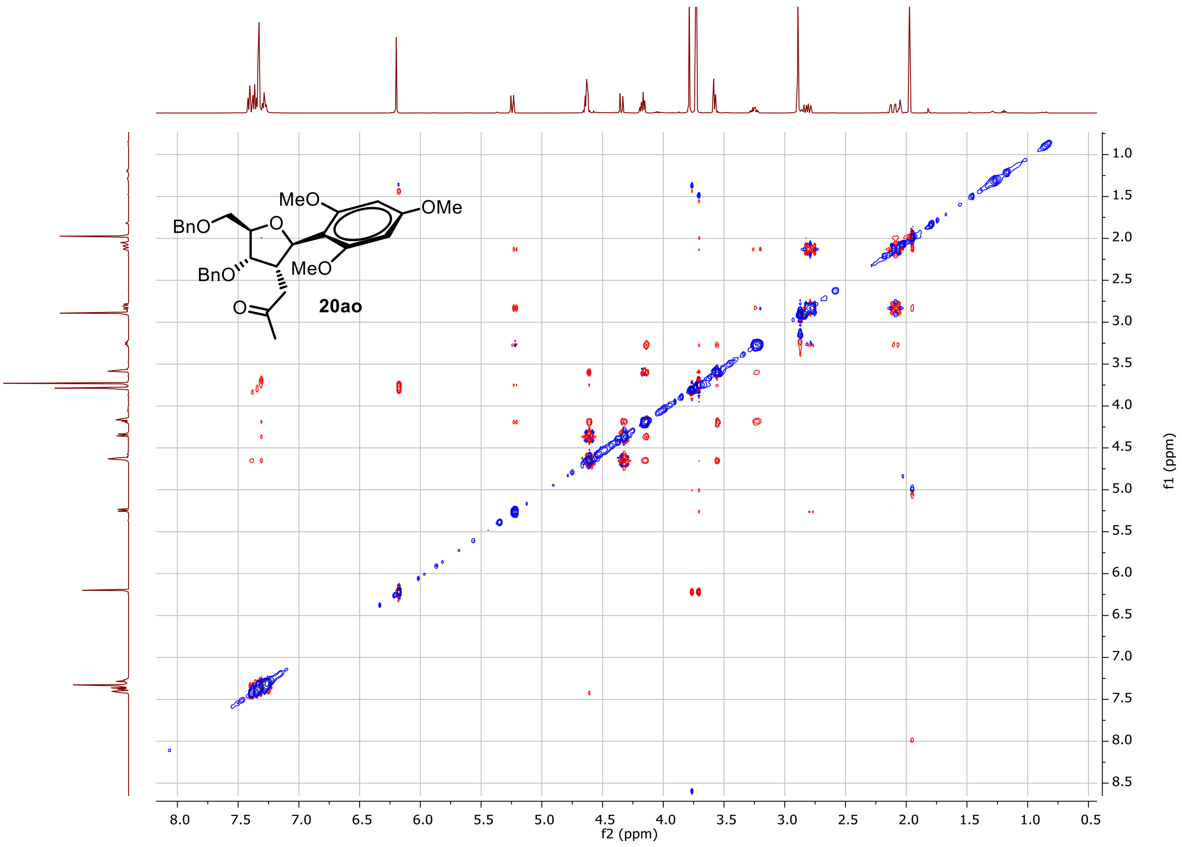

Supplementary Figure 312. NOESY spectra for **20ao**

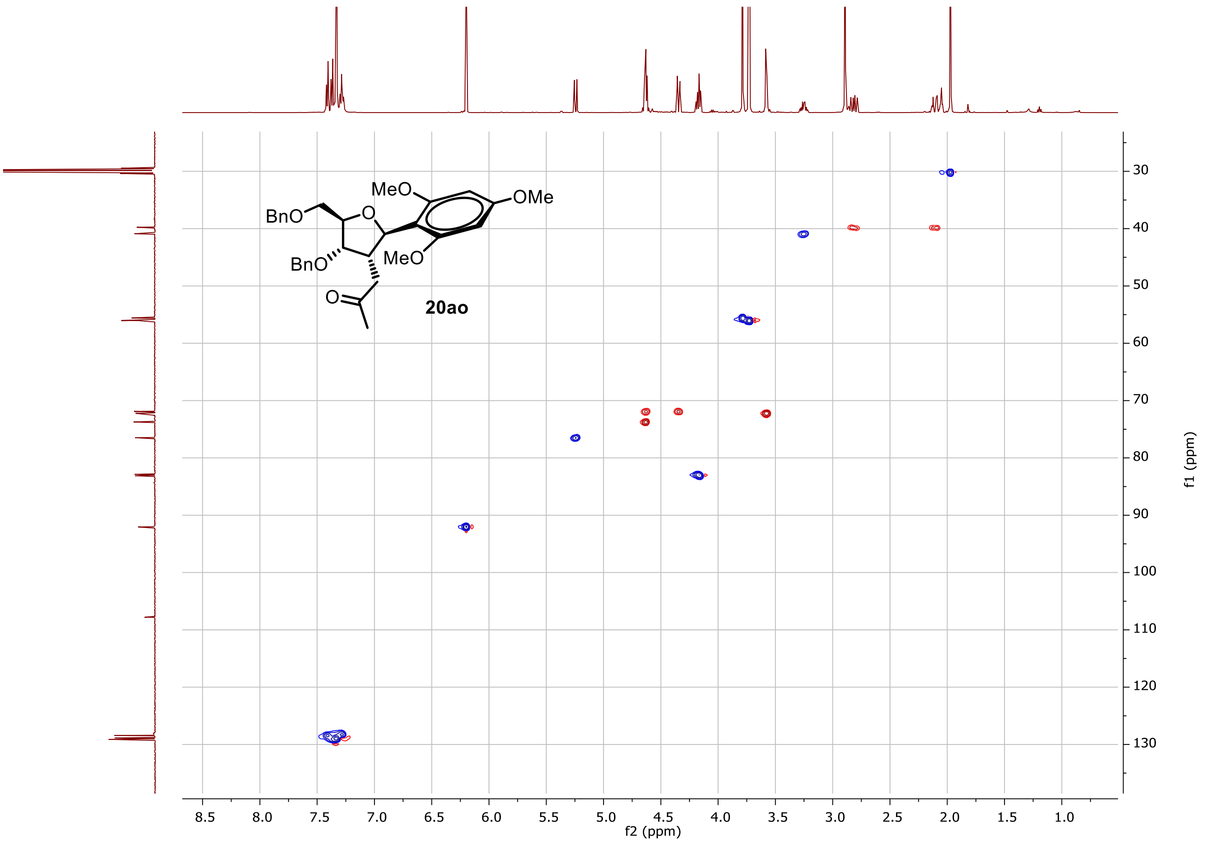

Supplementary Figure 313. HSQC spectra for **20ao**

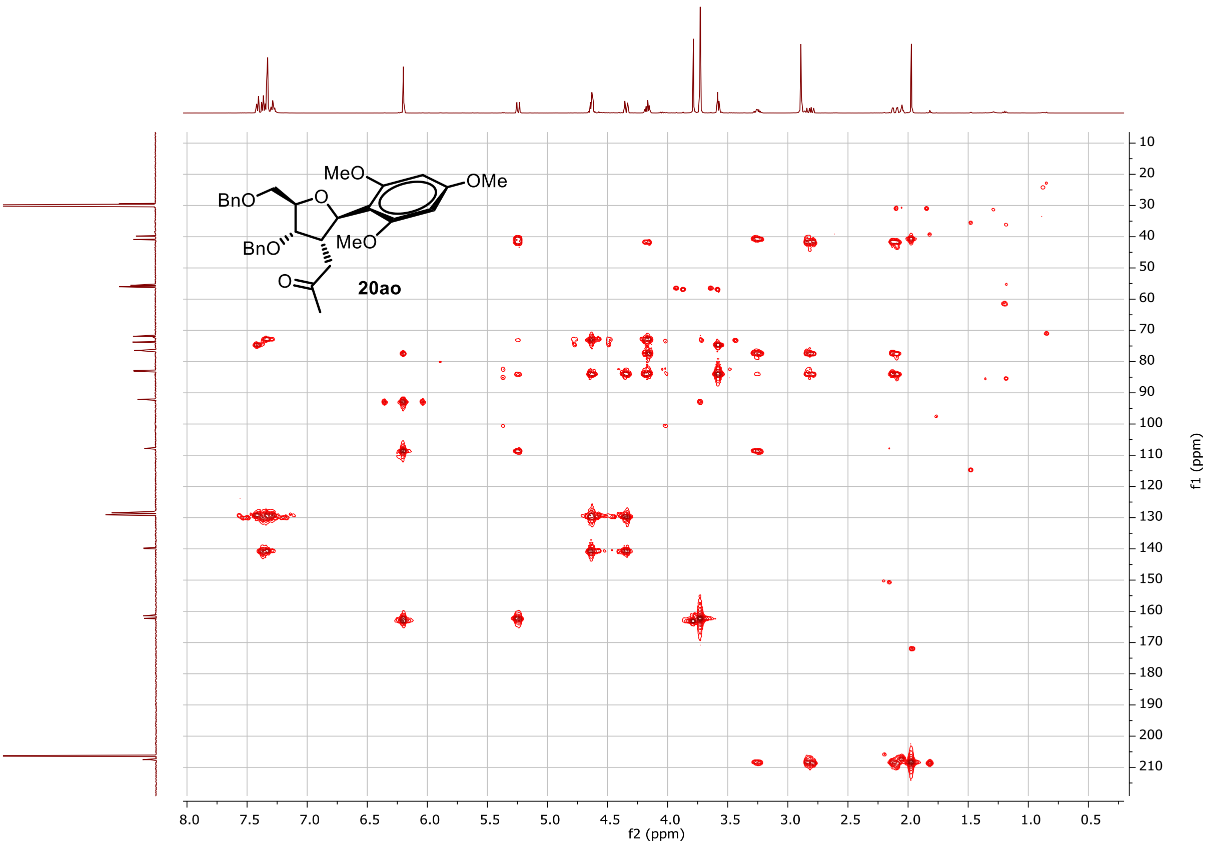

Supplementary Figure 314. HMBC spectra for **20ao**

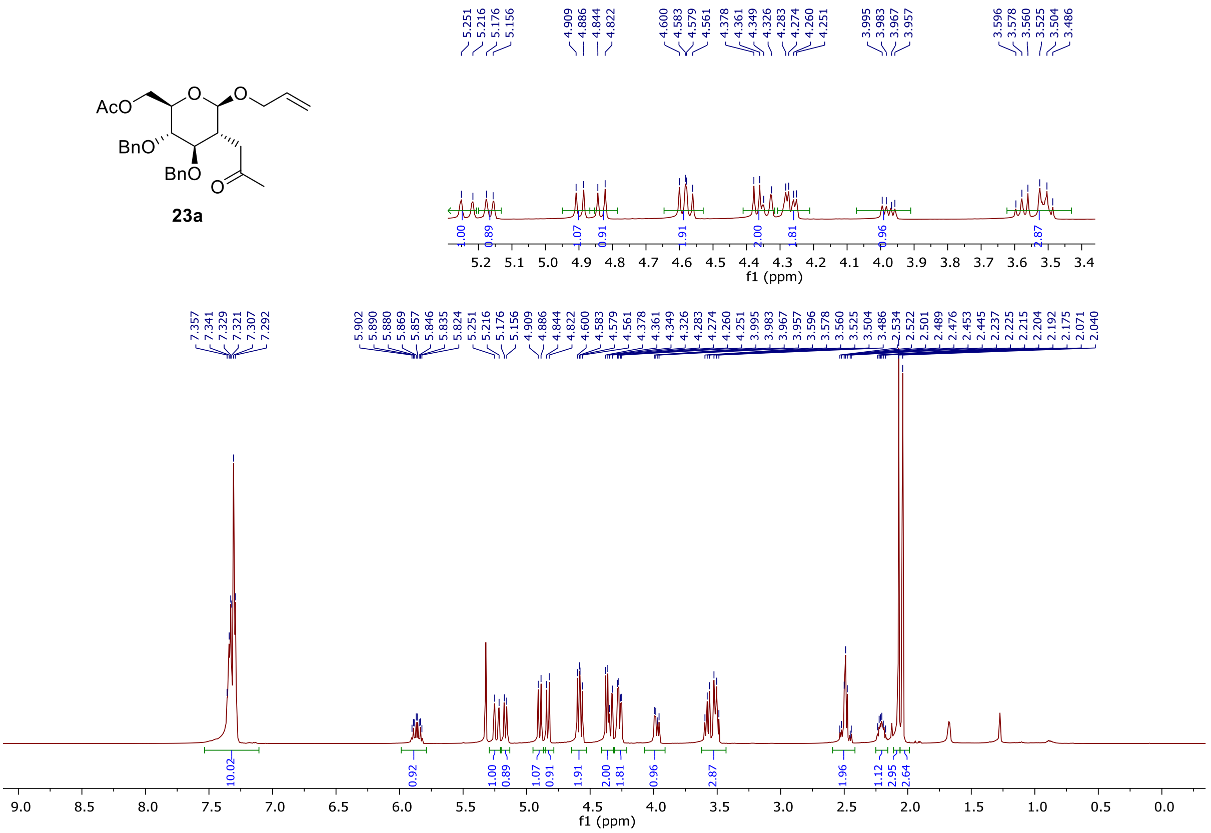

Supplementary Figure 315. ^{1}H spectra for **23a**

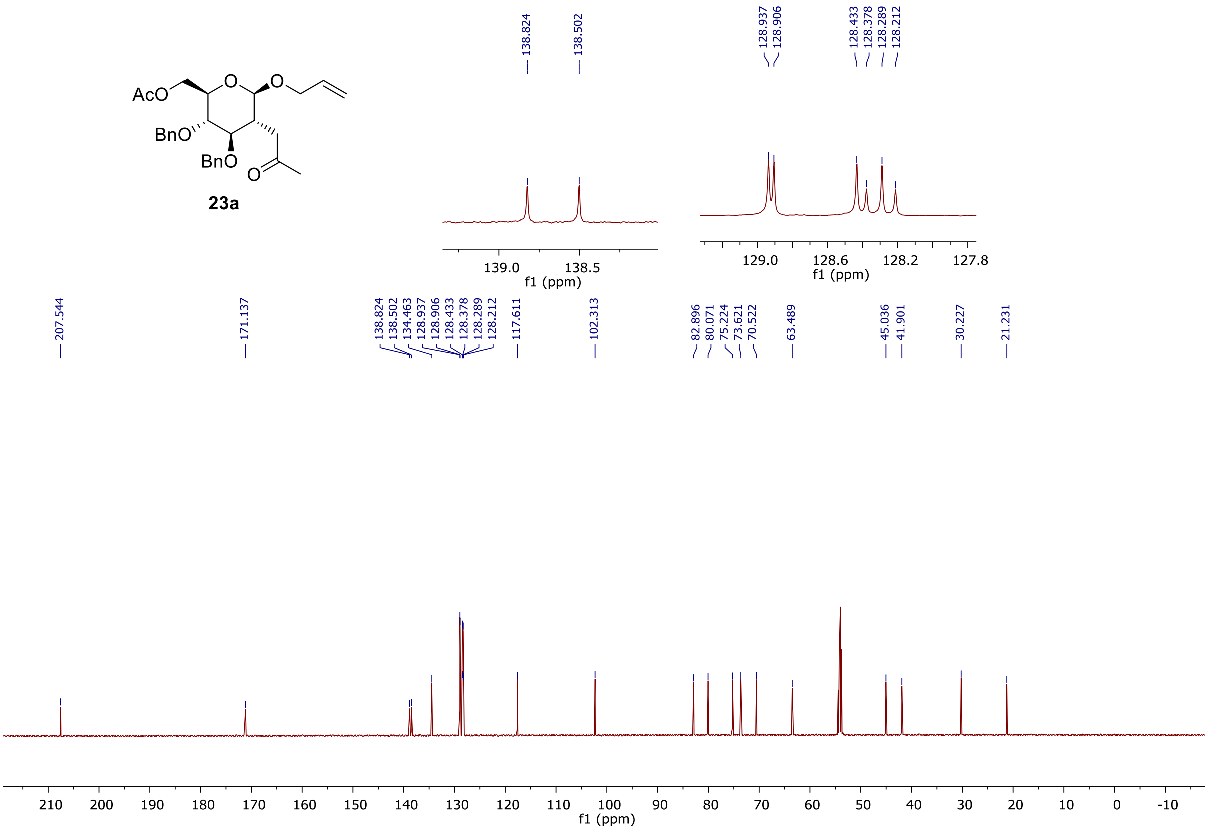

Supplementary Figure 316. ^{13}C spectra for **23a**

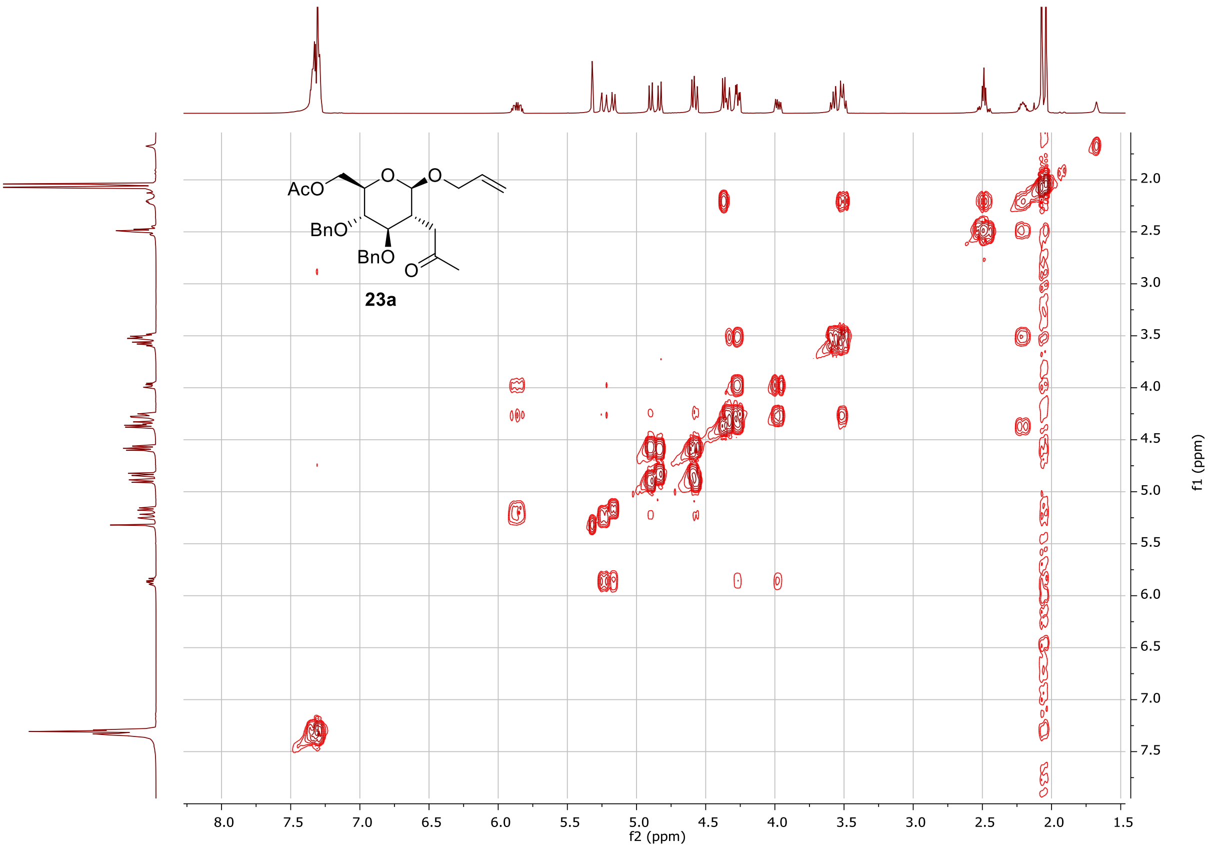

Supplementary Figure 317. COSY spectra for **23a**

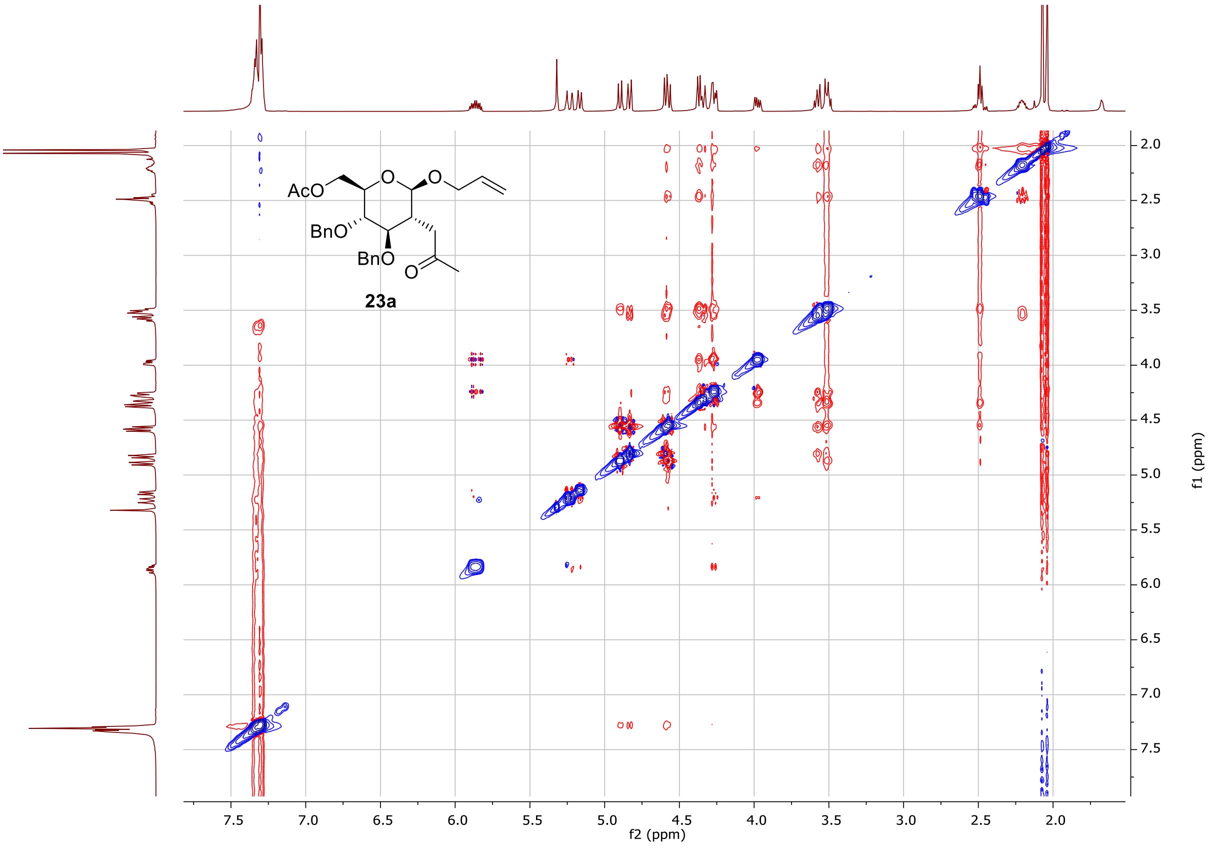

Supplementary Figure 318. NOESY spectra for **23a**

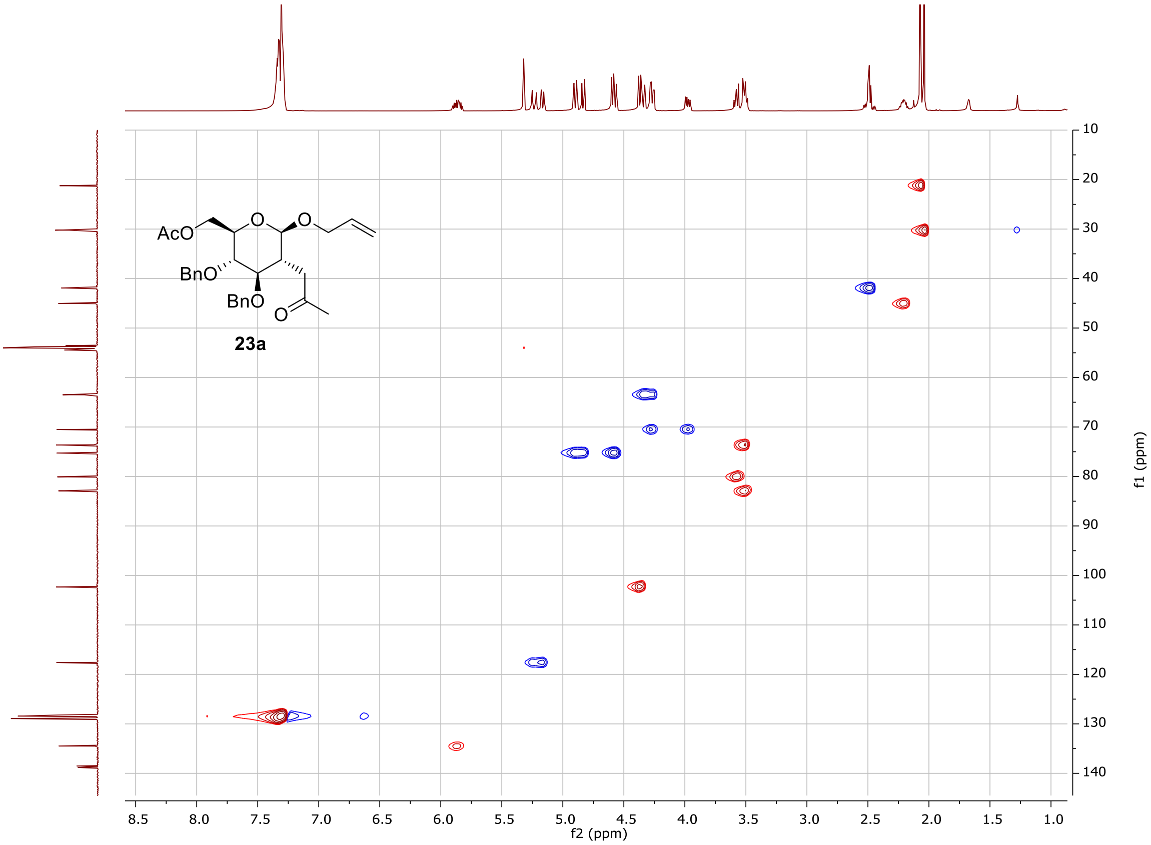

Supplementary Figure 319. HSQC spectra for **23a**

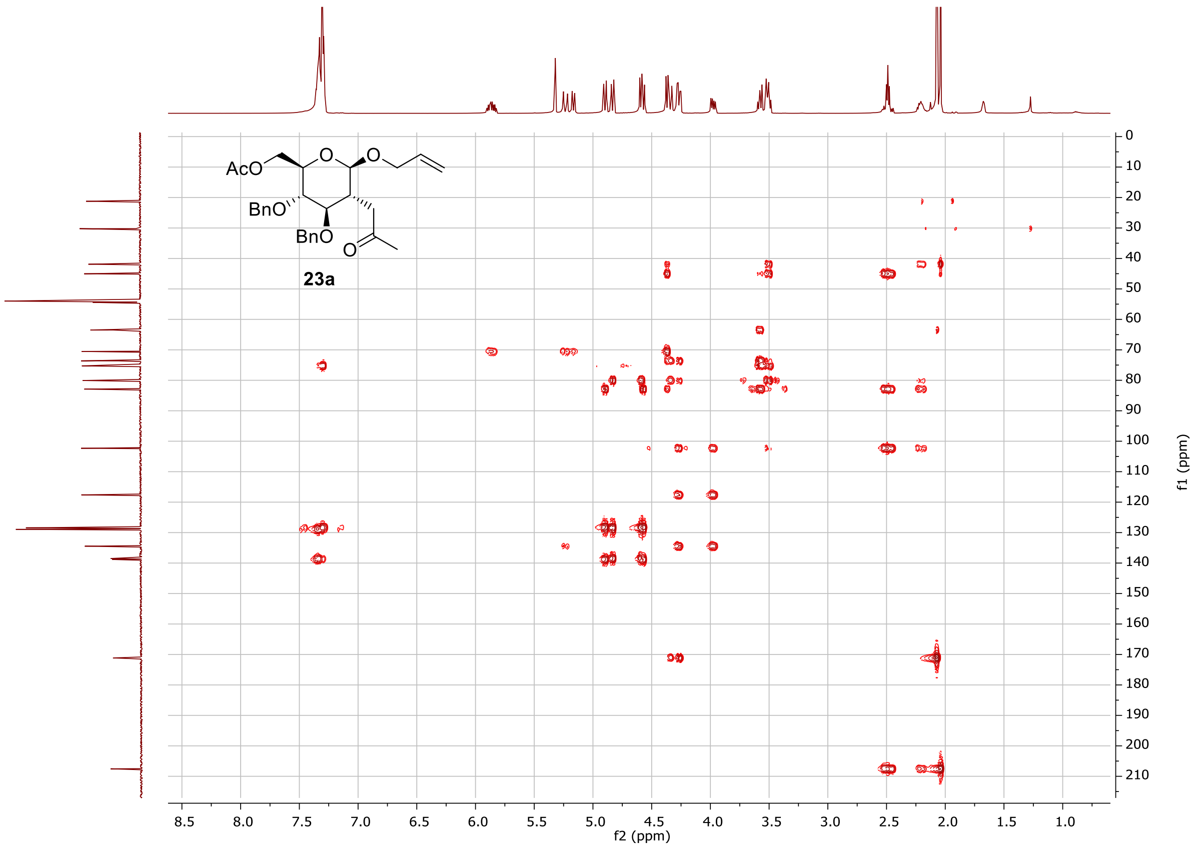

Supplementary Figure 320. HMBC spectra for **23a**

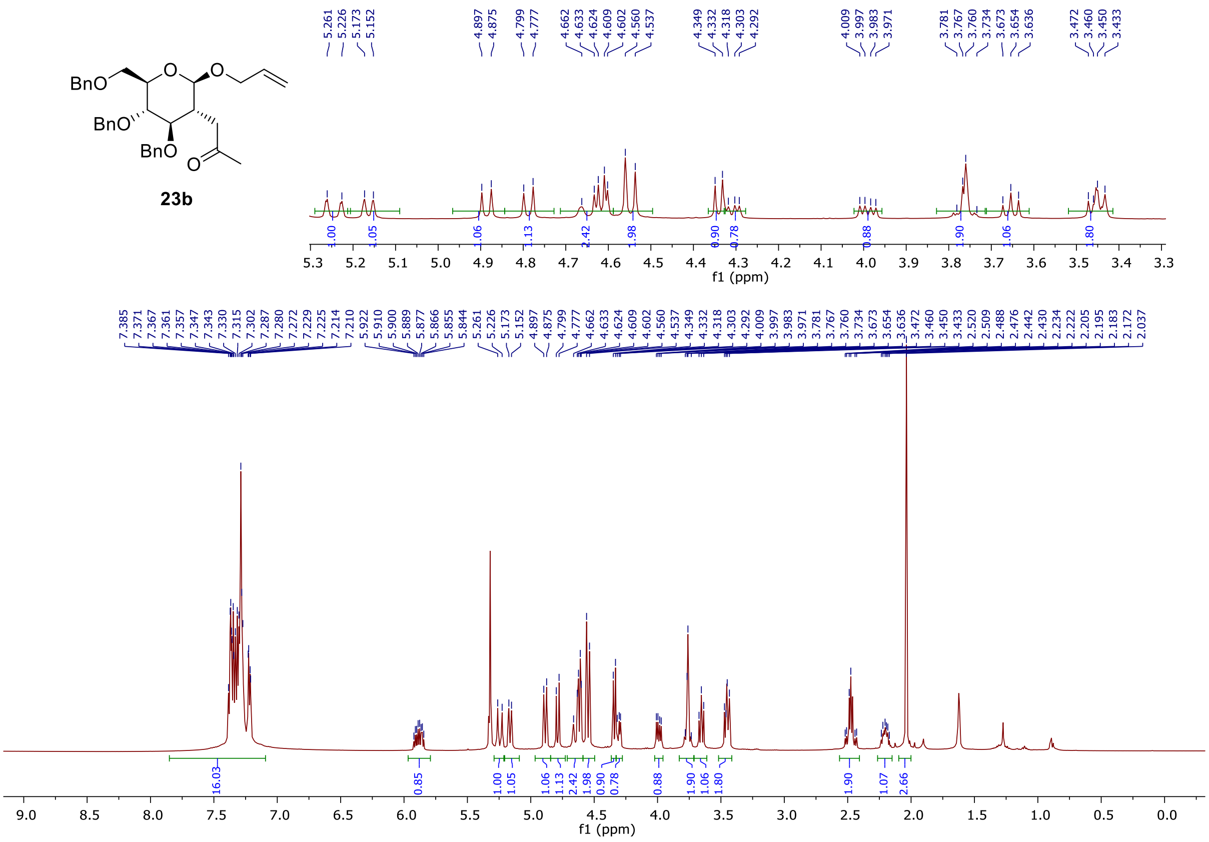

Supplementary Figure 321. ^{1}H spectra for **23b**

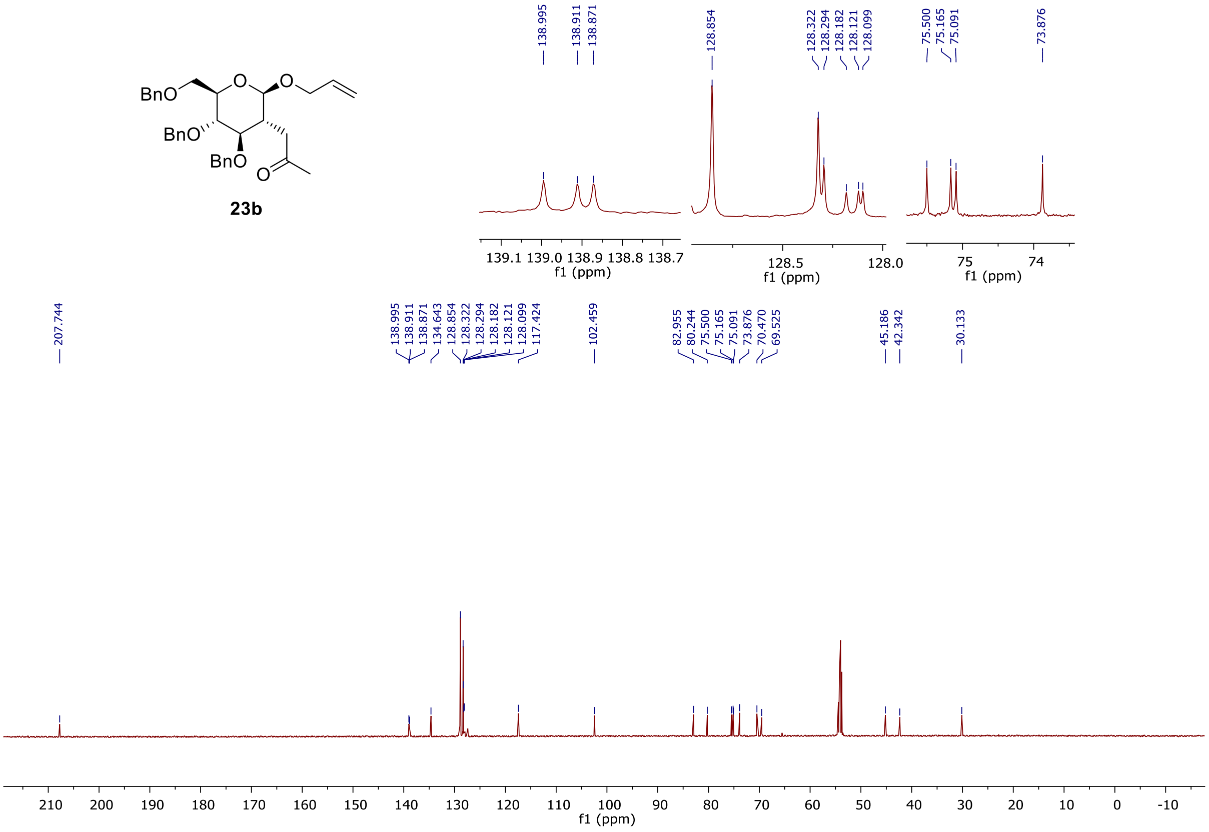

Supplementary Figure 322. ^{13}C spectra for **23b**

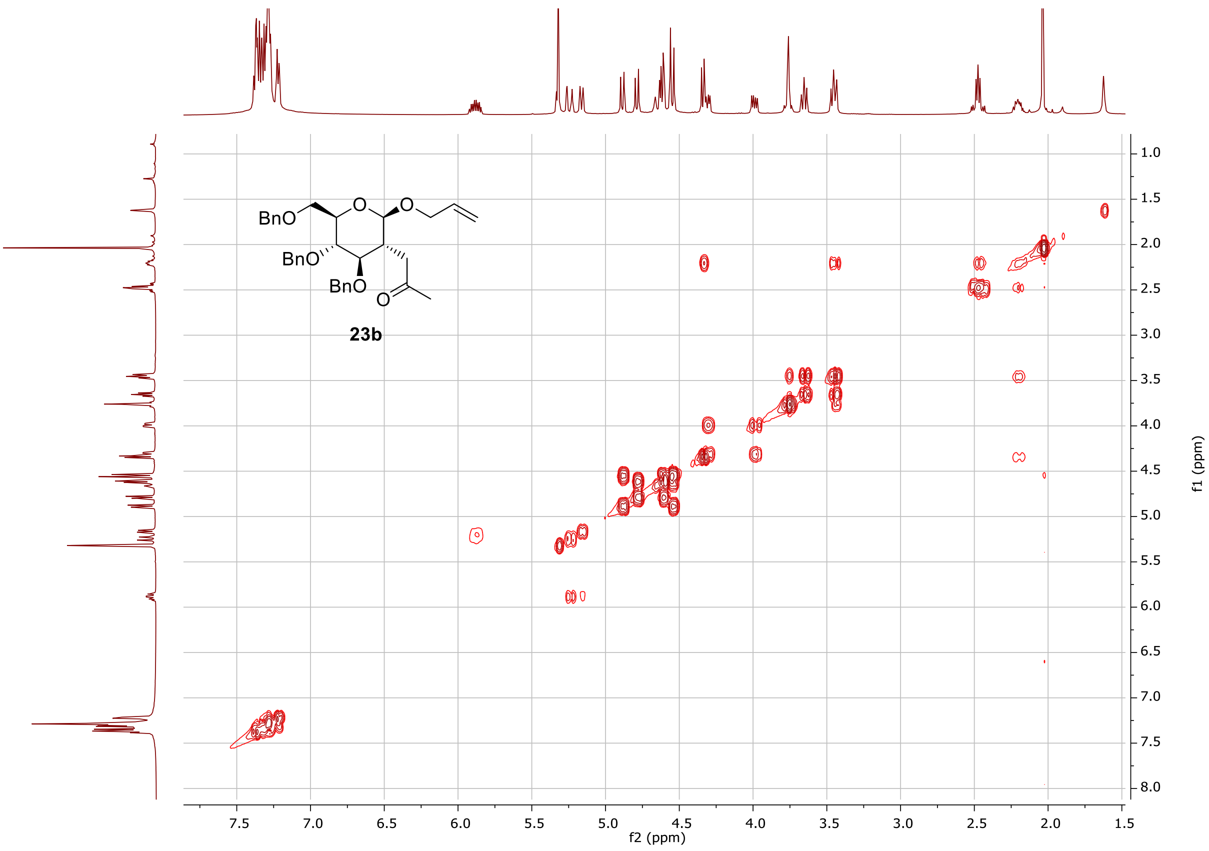

Supplementary Figure 323. COSY spectra for **23b**

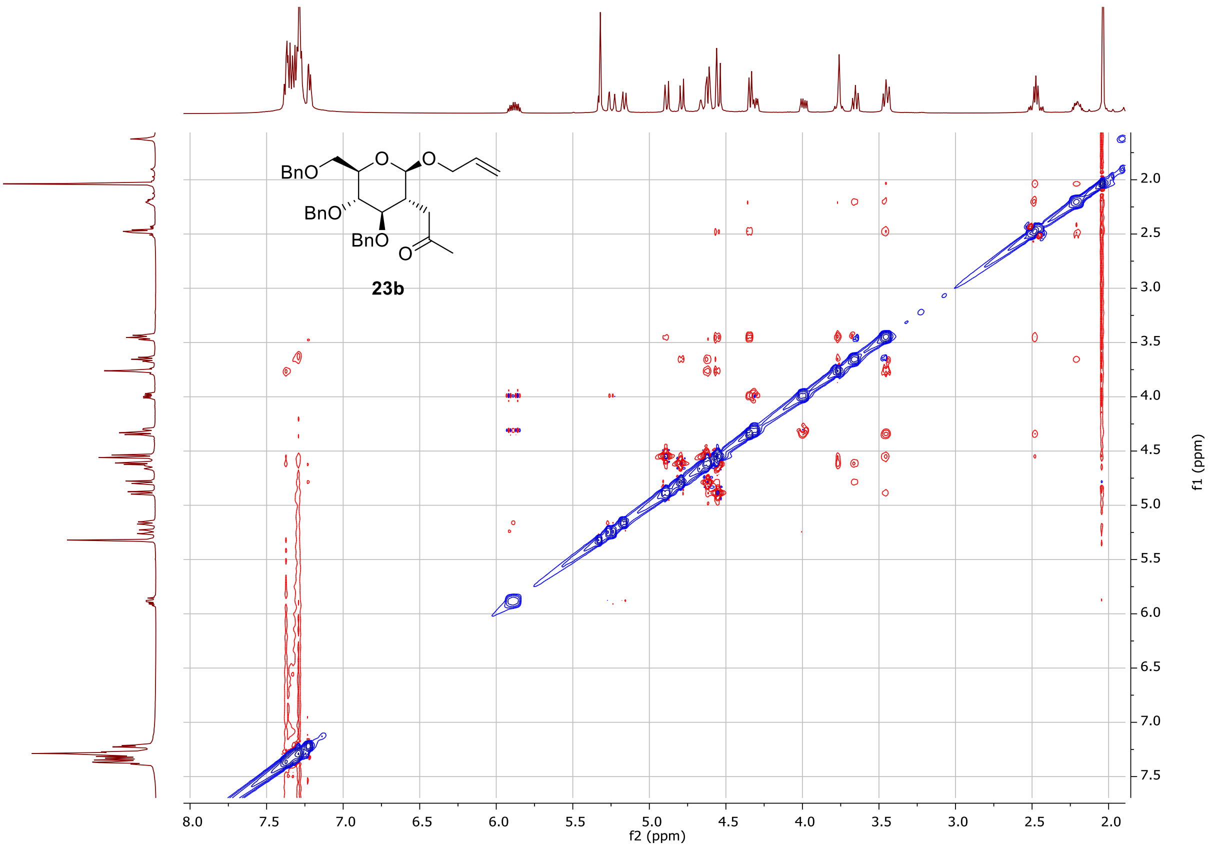

Supplementary Figure 324. NOESY spectra for **23b**

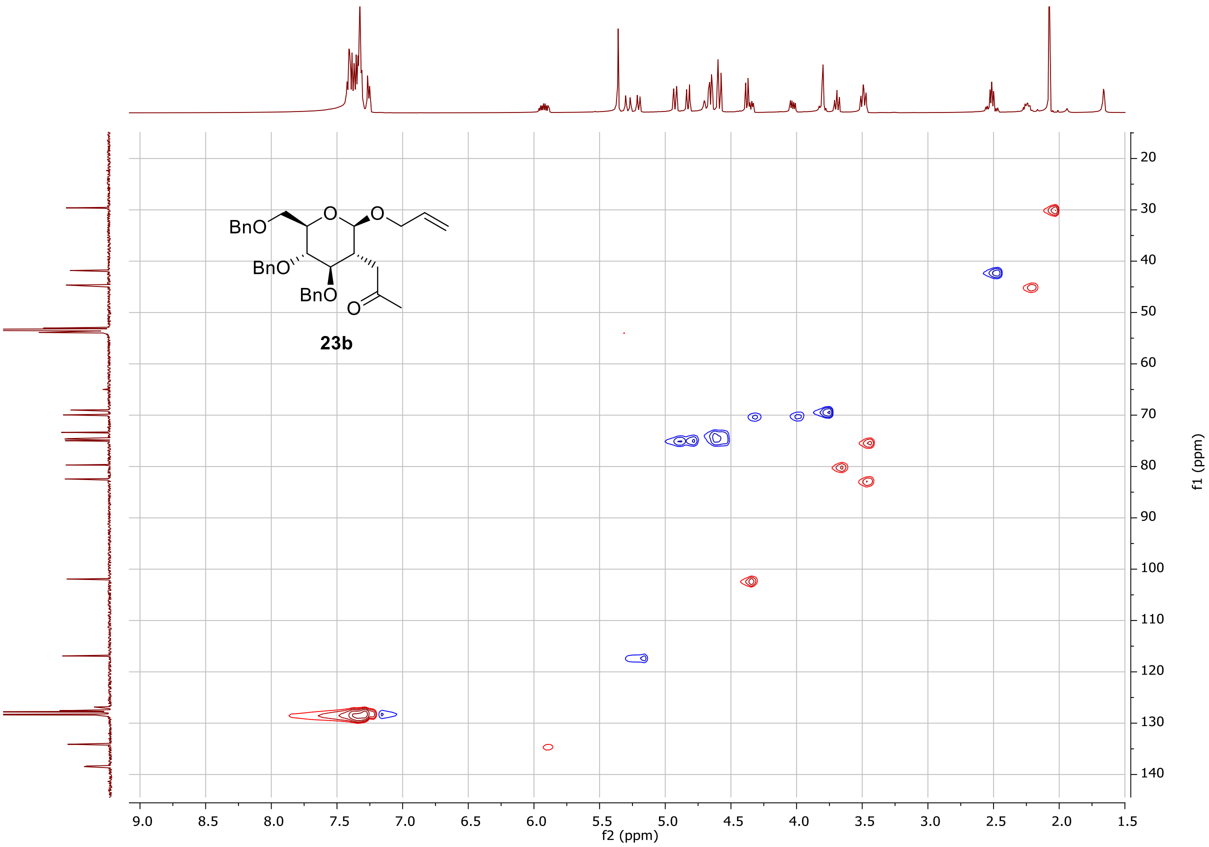

Supplementary Figure 325. HSQC spectra for **23b**

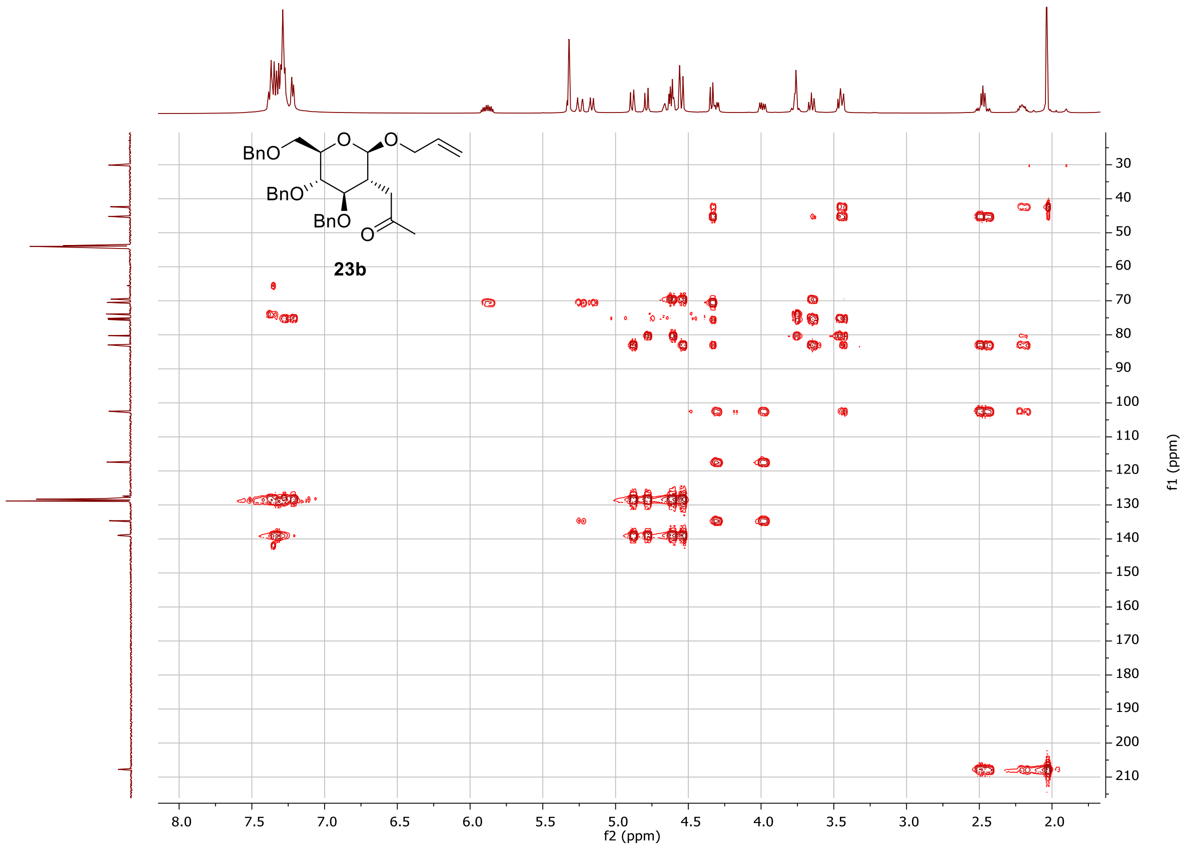

Supplementary Figure 326. HMBC spectra for **23b**

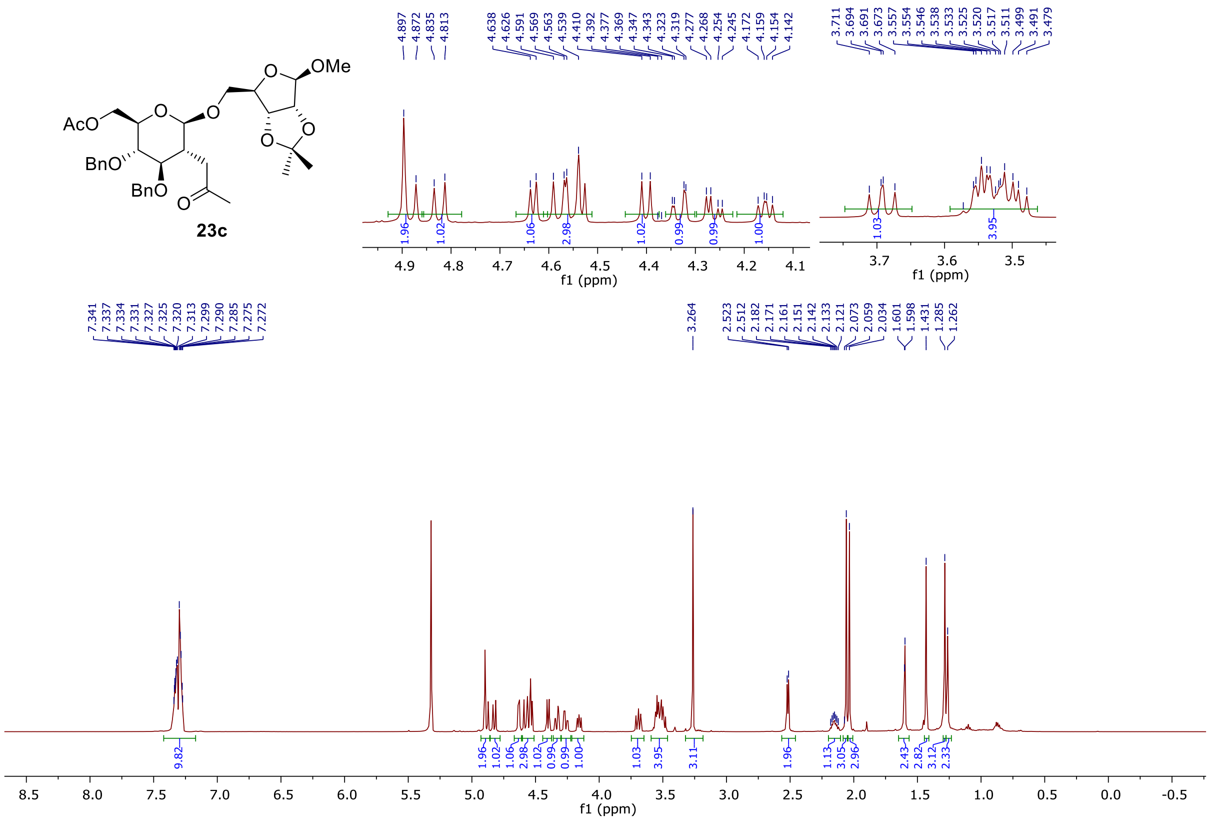

Supplementary Figure 327. ^{1}H spectra for **23c**

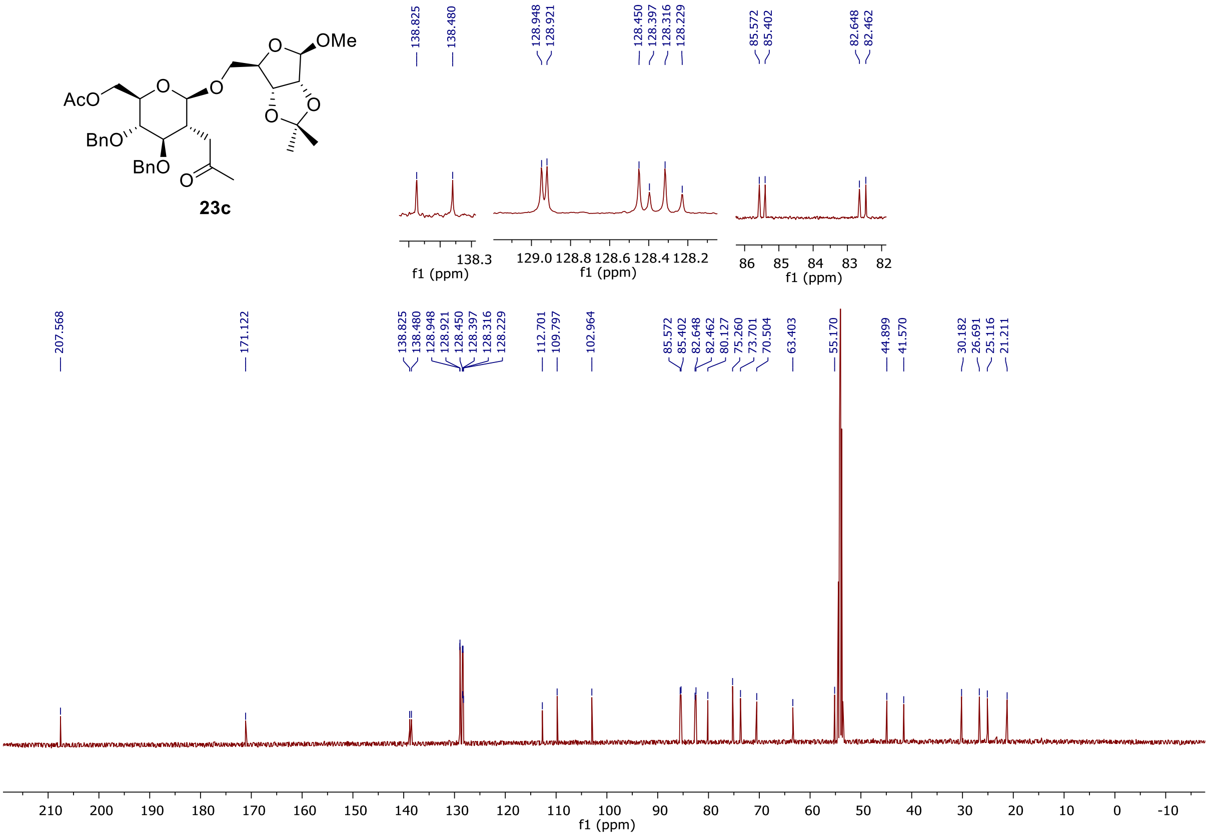

Supplementary Figure 328. ^{13}C spectra for **23c**

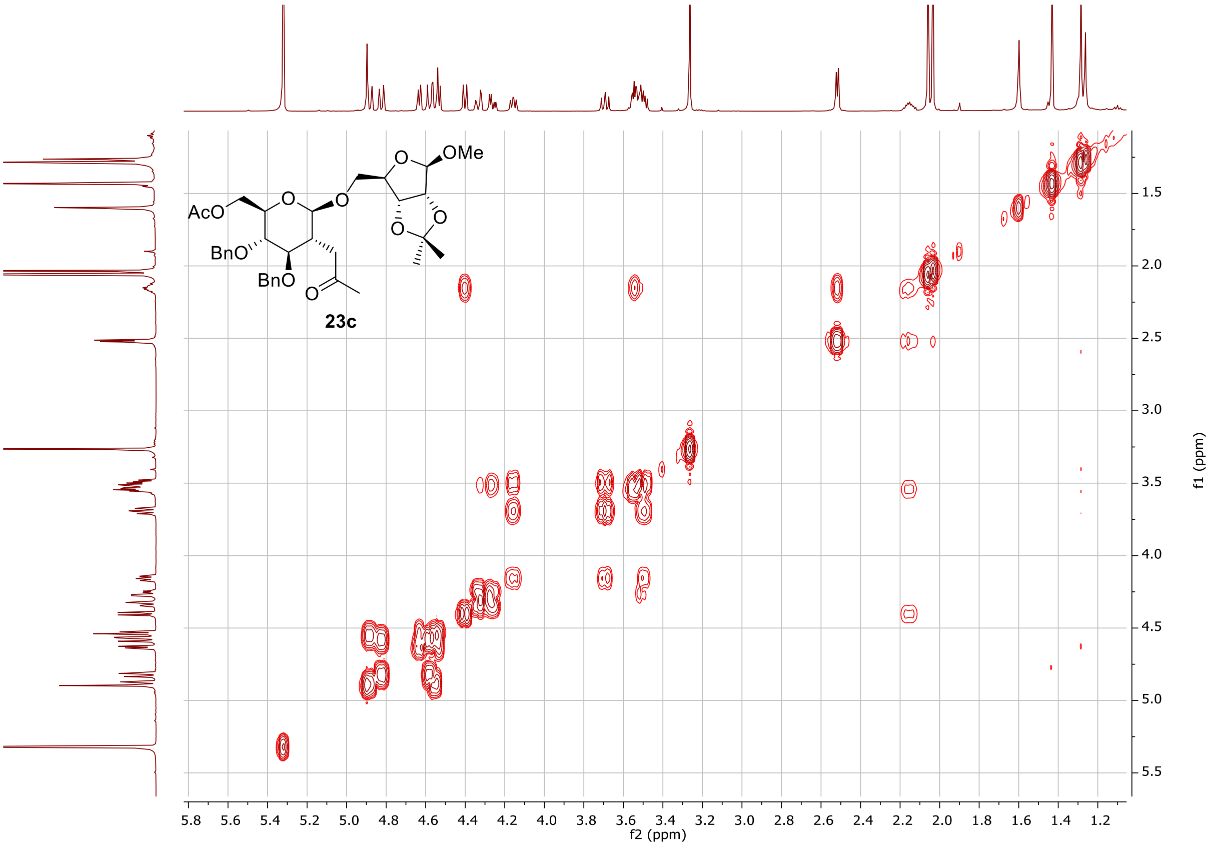

Supplementary Figure 329. COSY spectra for **23c**

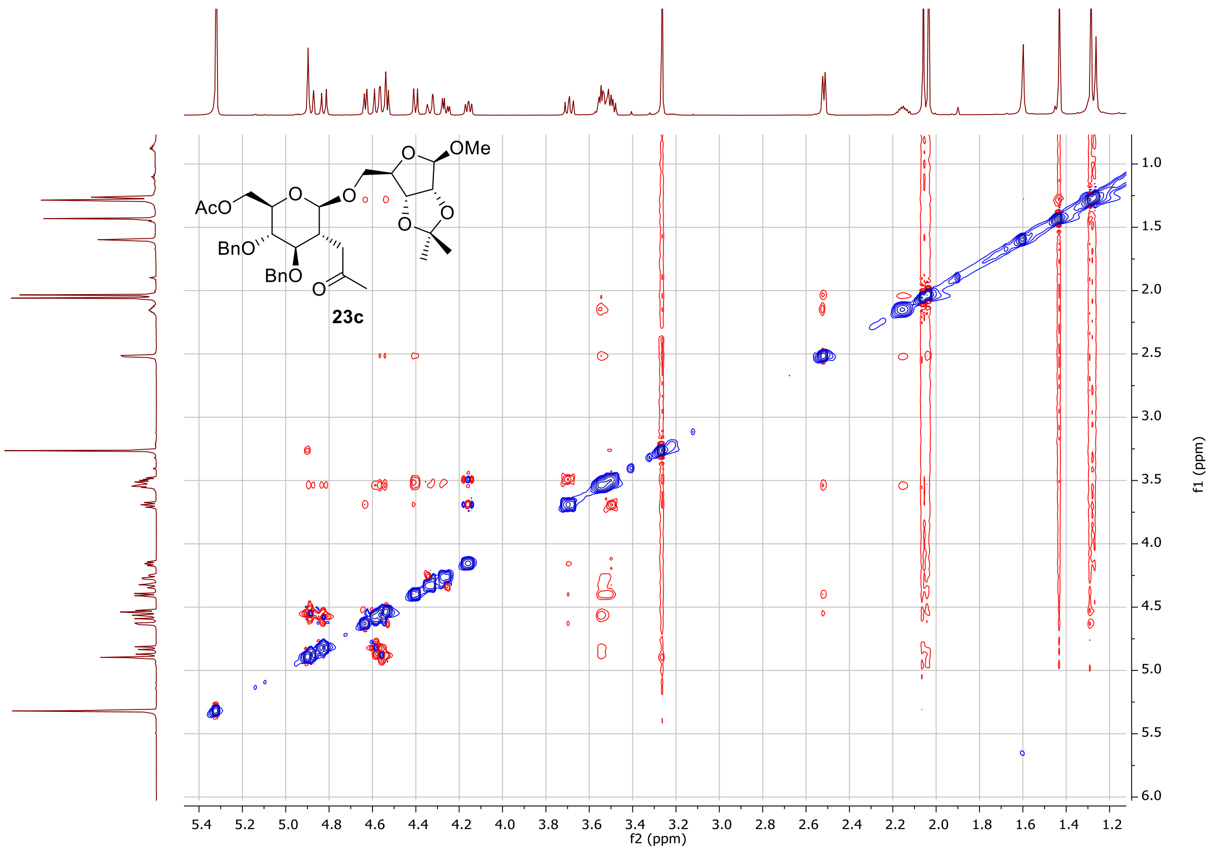

Supplementary Figure 330. NOESY spectra for **23c**

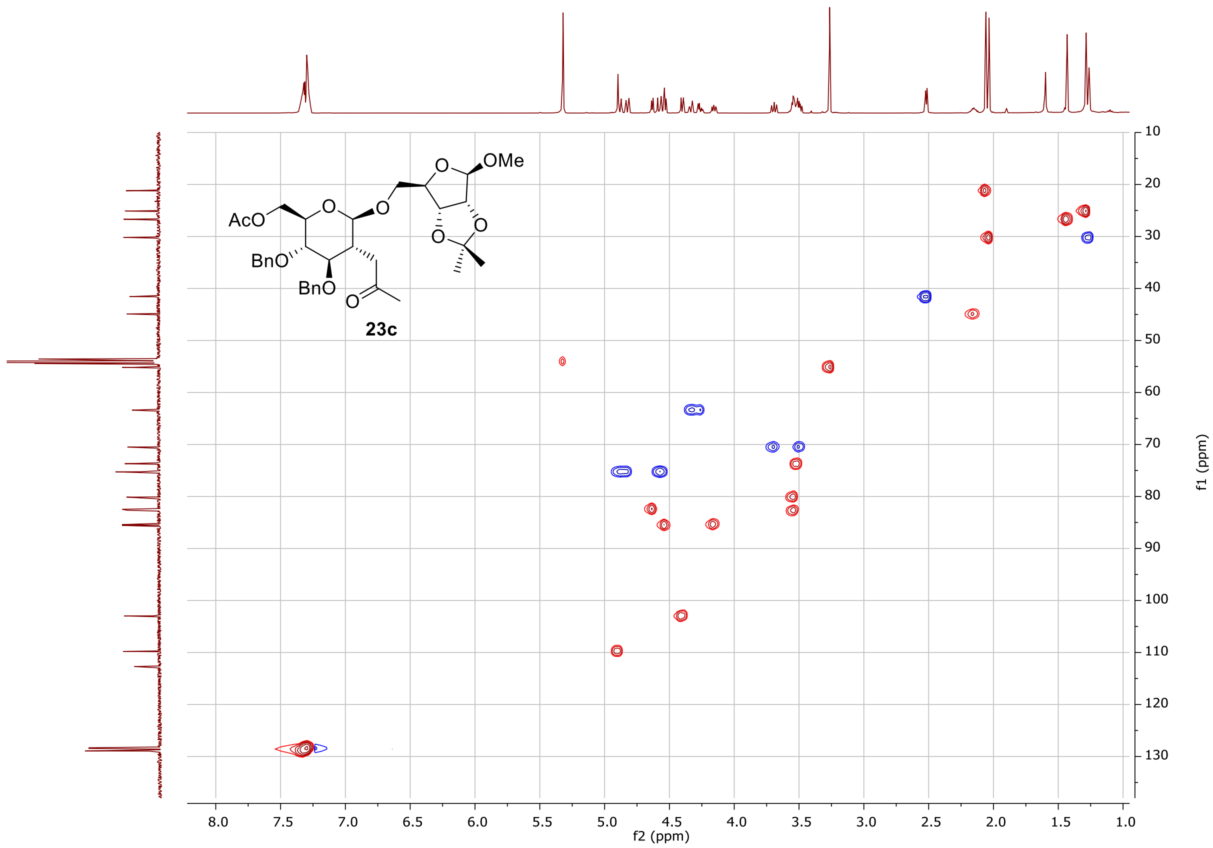

Supplementary Figure 331. HSQC spectra for **23c**

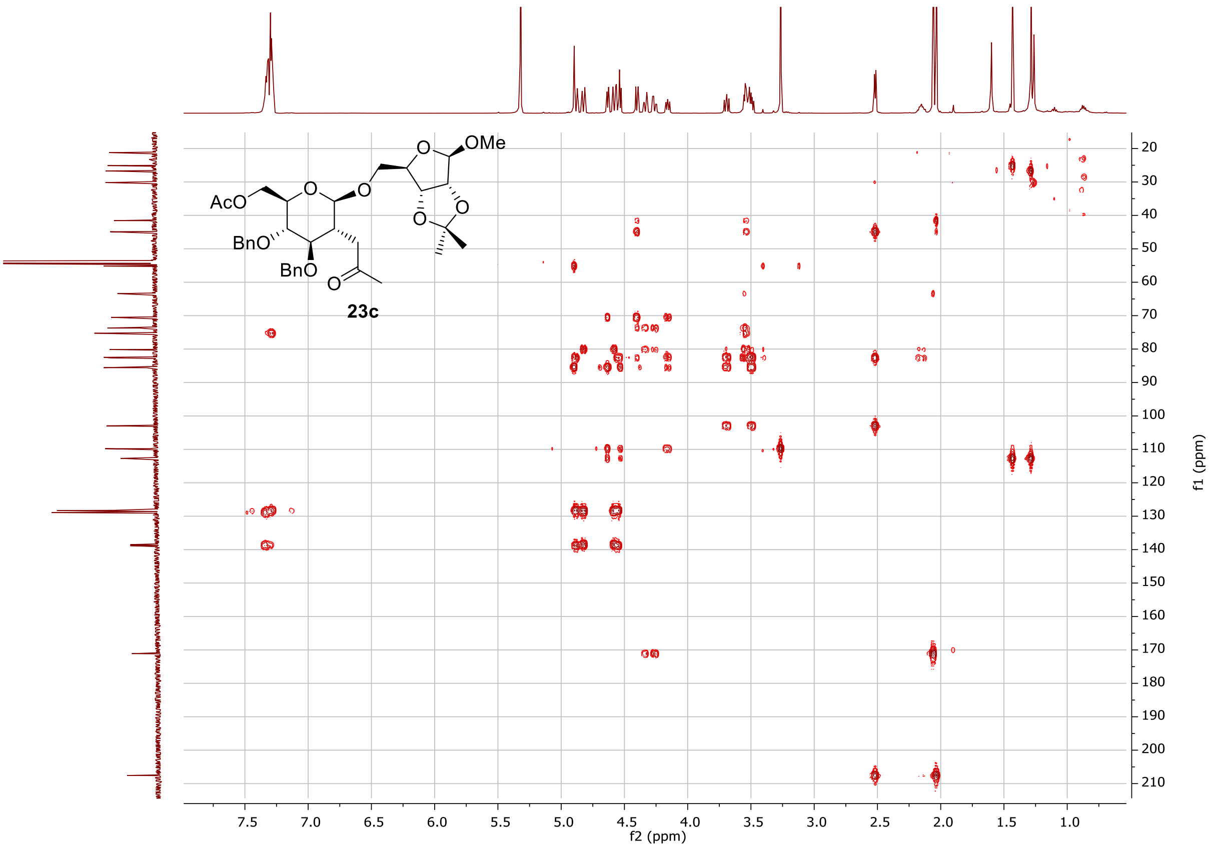

Supplementary Figure 332. HMBC spectra for **23c**

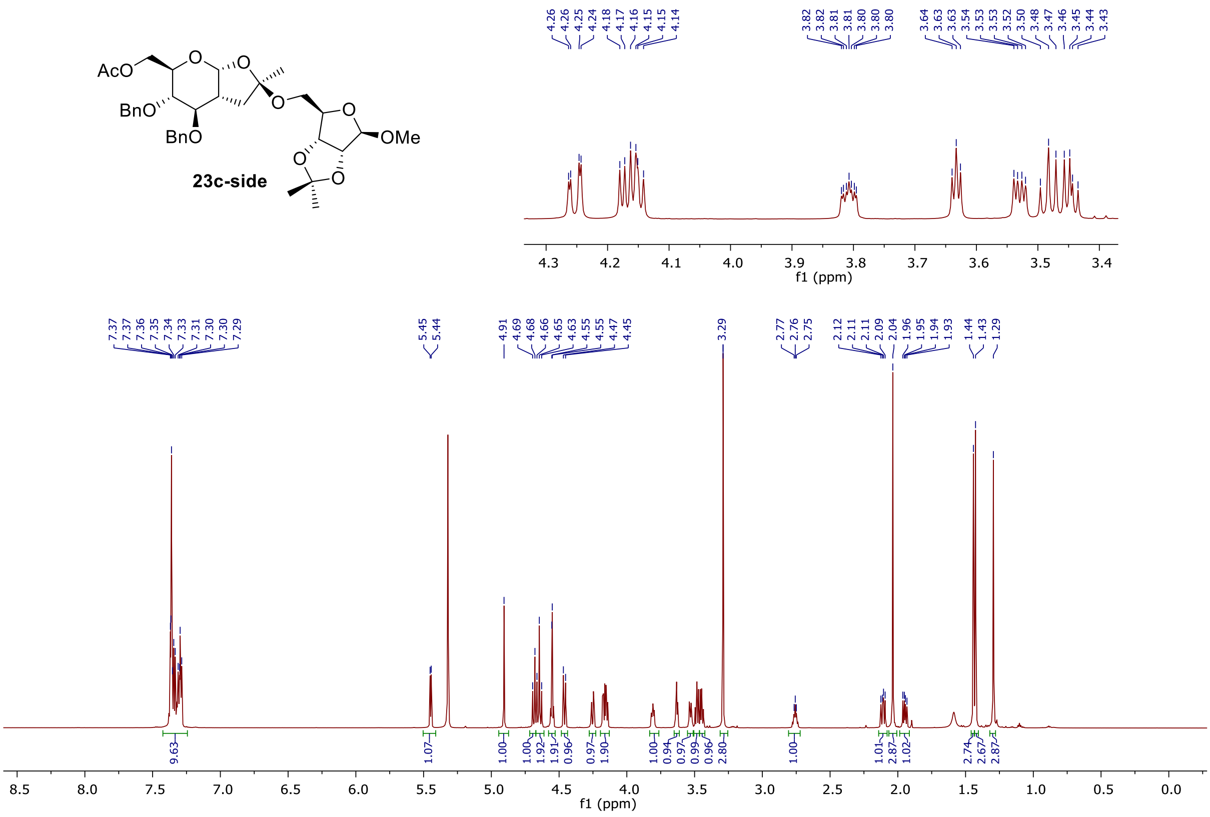

Supplementary Figure 333. ^{1}H spectra for **23c-side**

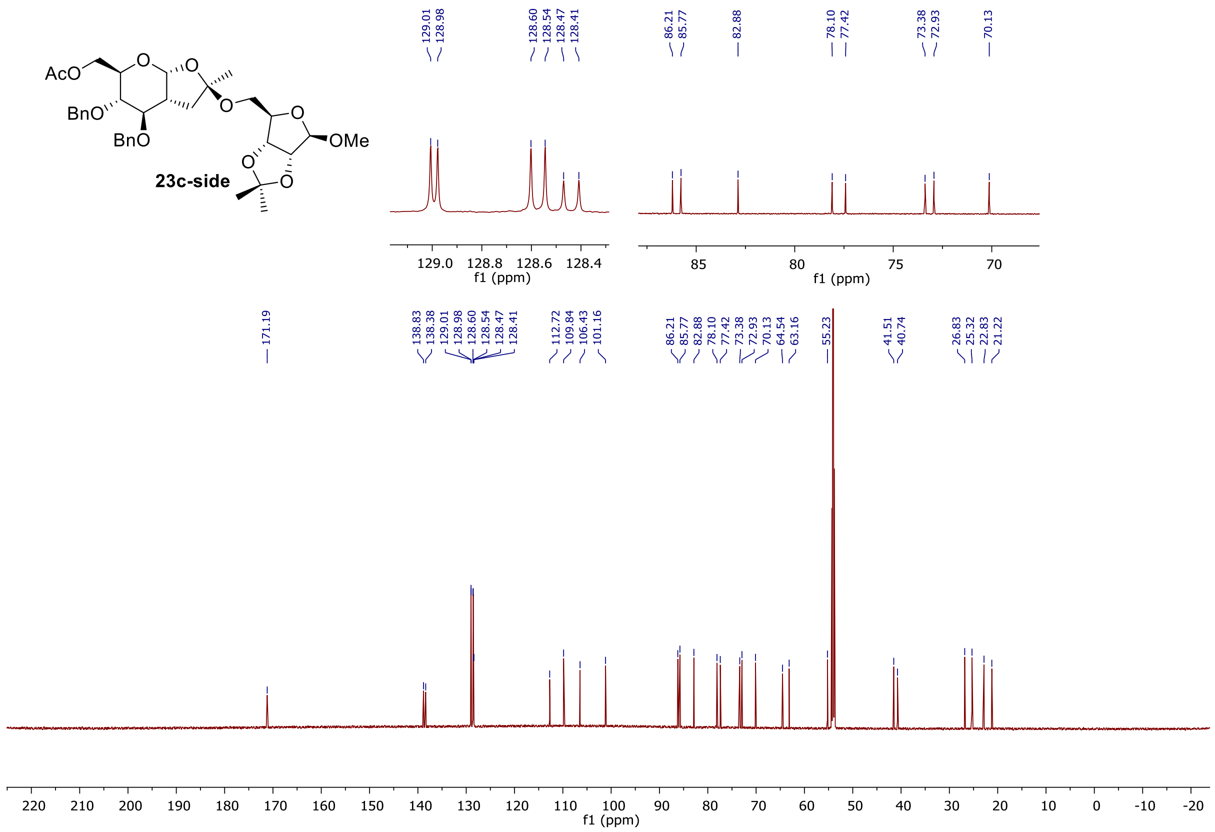

Supplementary Figure 334. ^{13}C spectra for **23c-side**

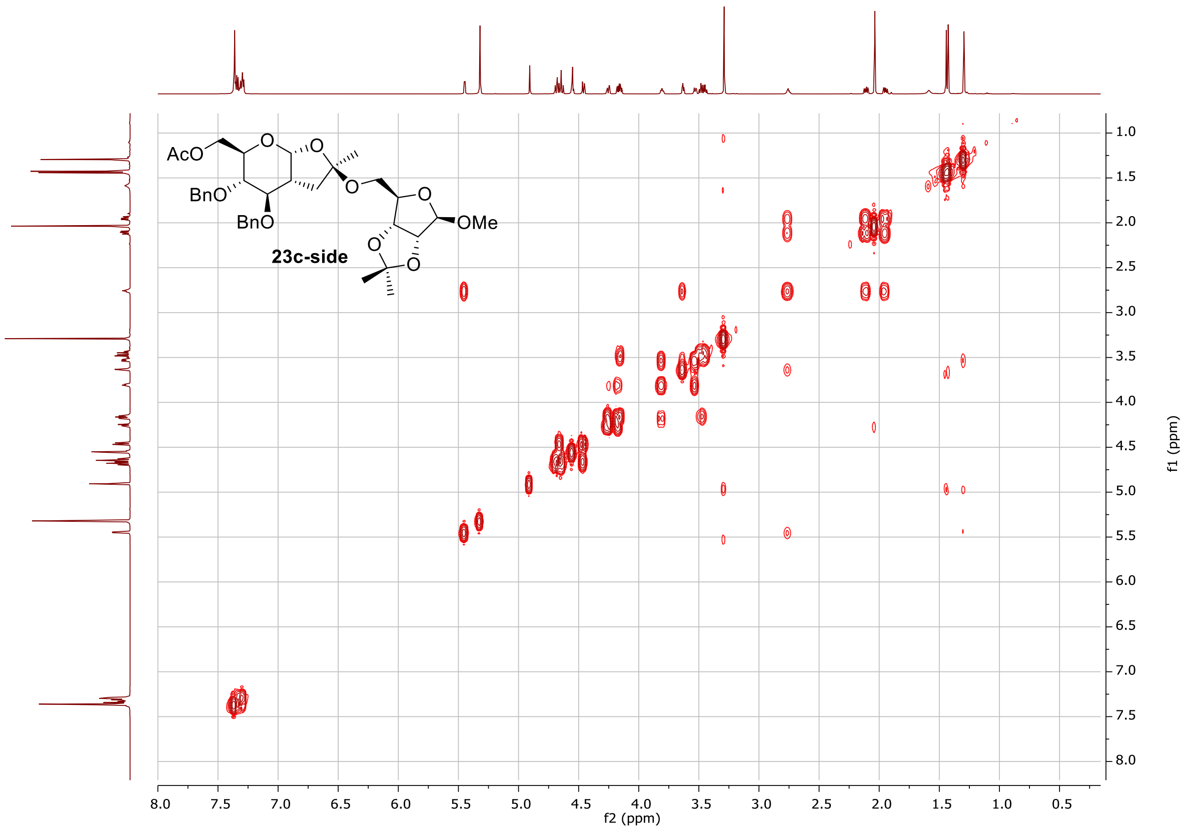

Supplementary Figure 335. COSY spectra for **23c-side**

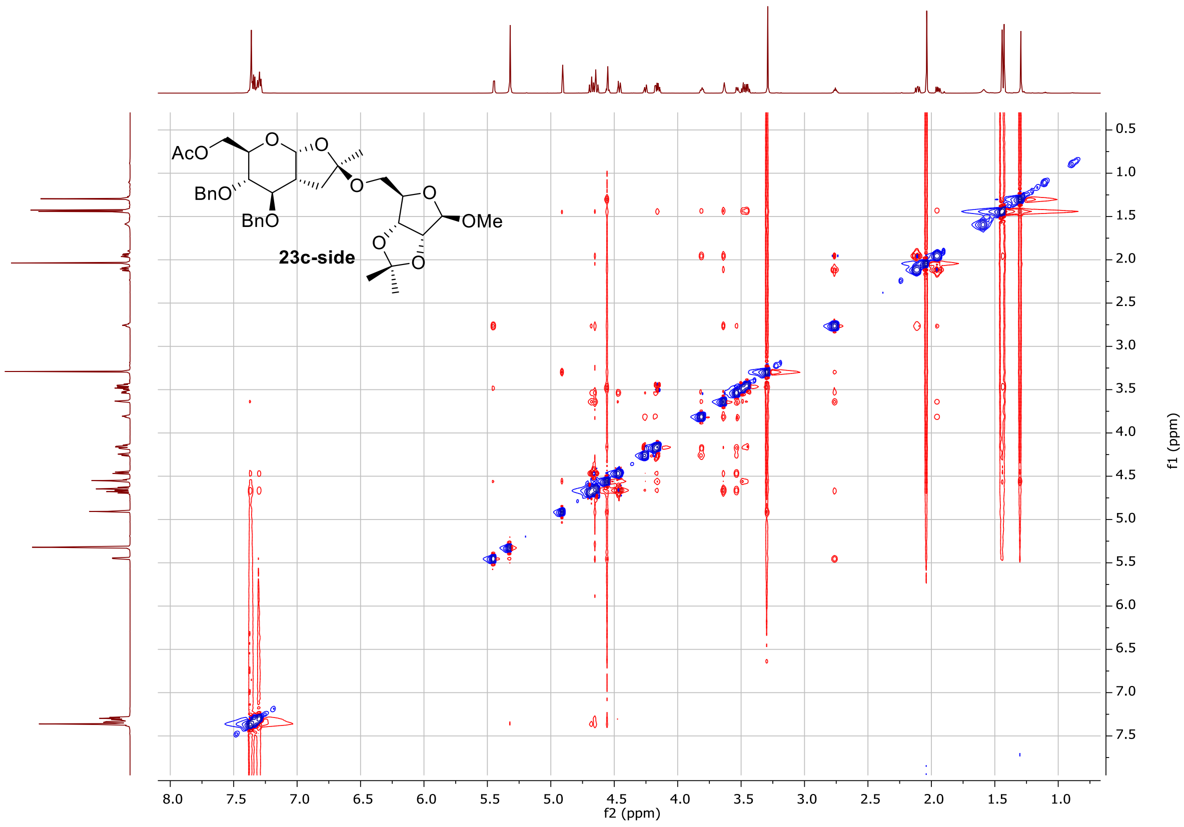

Supplementary Figure 336. NOESY spectra for **23c-side**

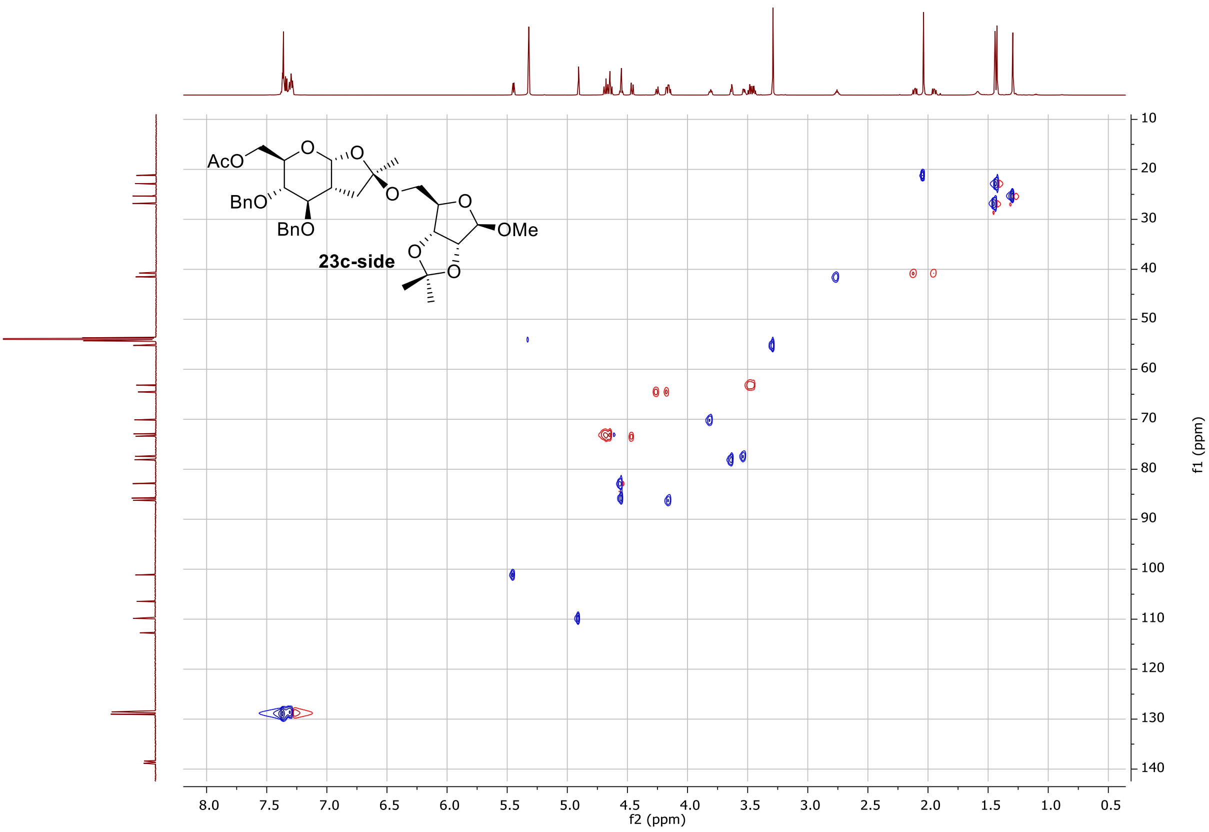

Supplementary Figure 337. HSQC spectra for **23c-side**

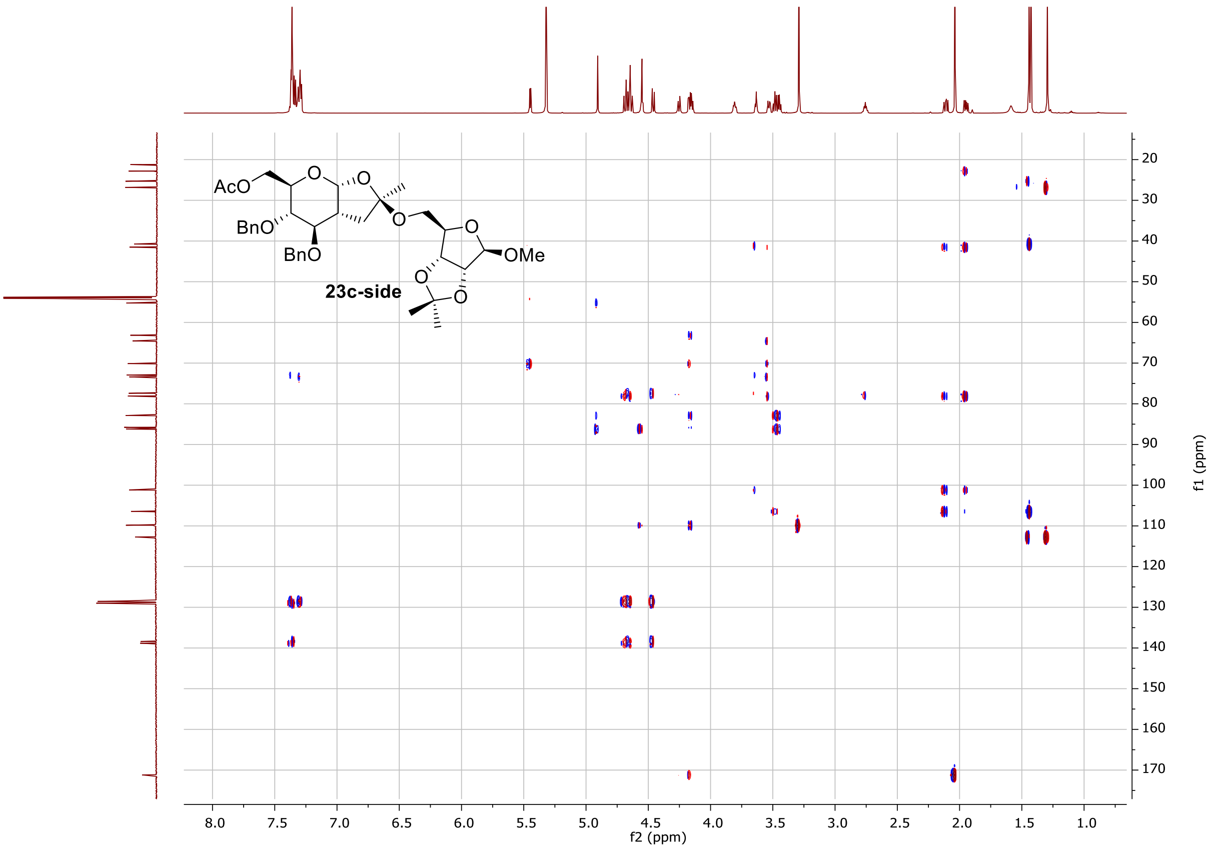

Supplementary Figure 338. HMBC spectra for **23c-side**

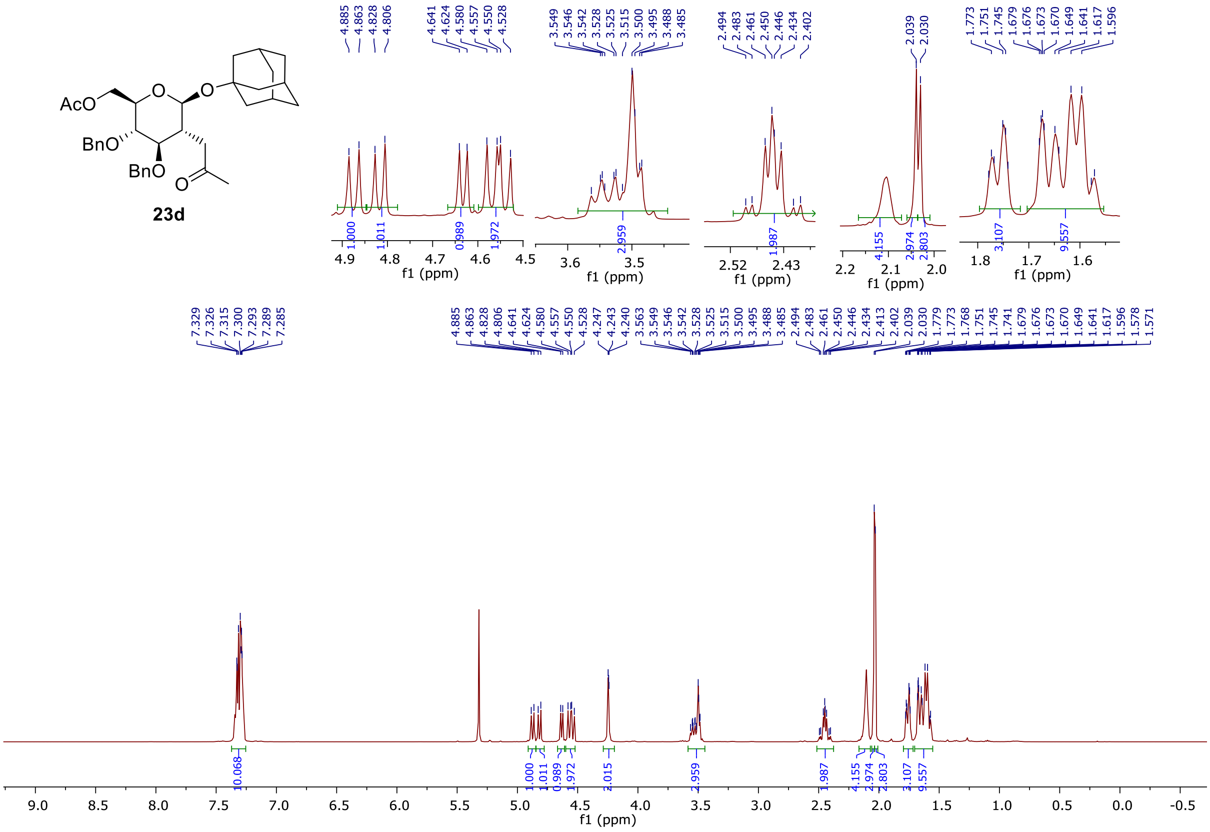

Supplementary Figure 339. ^{1}H spectra for **23d**

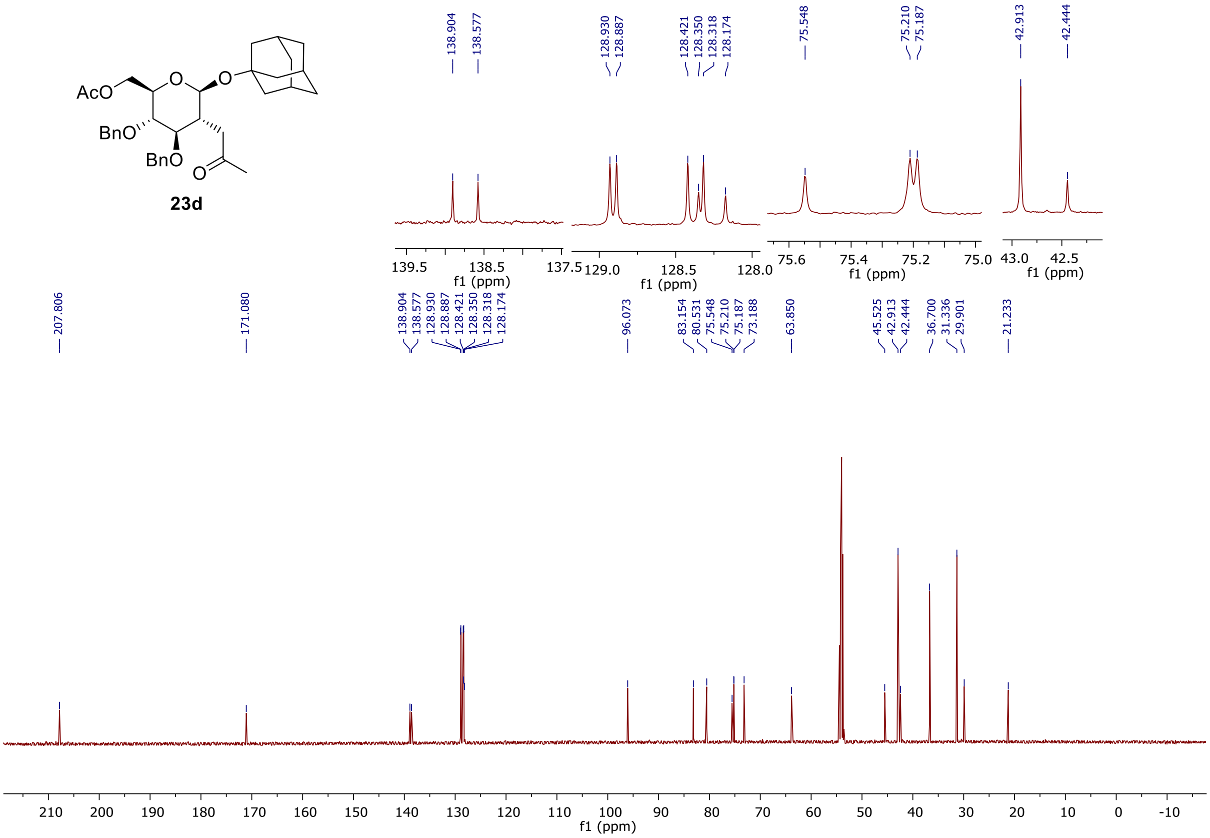

Supplementary Figure 340. ^{13}C spectra for **23d**

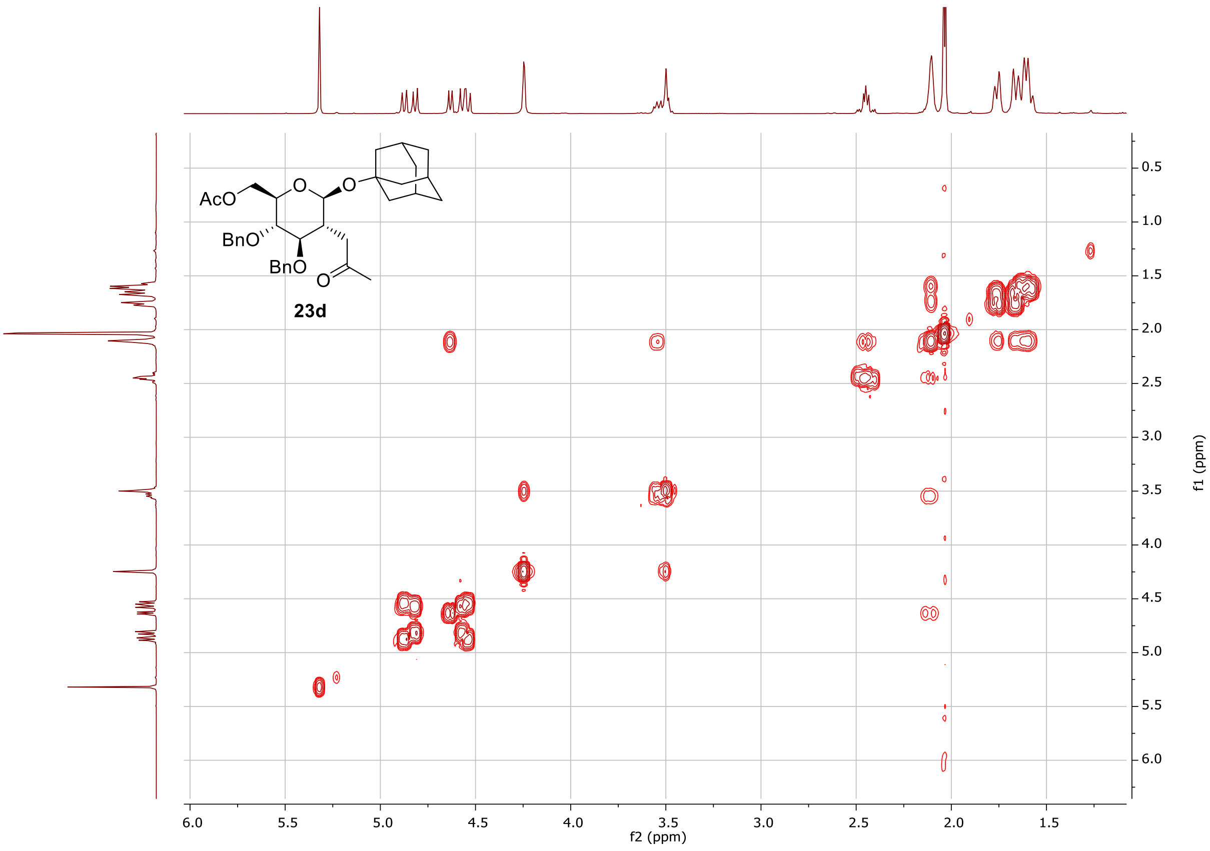

Supplementary Figure 341. COSY spectra for **23d**

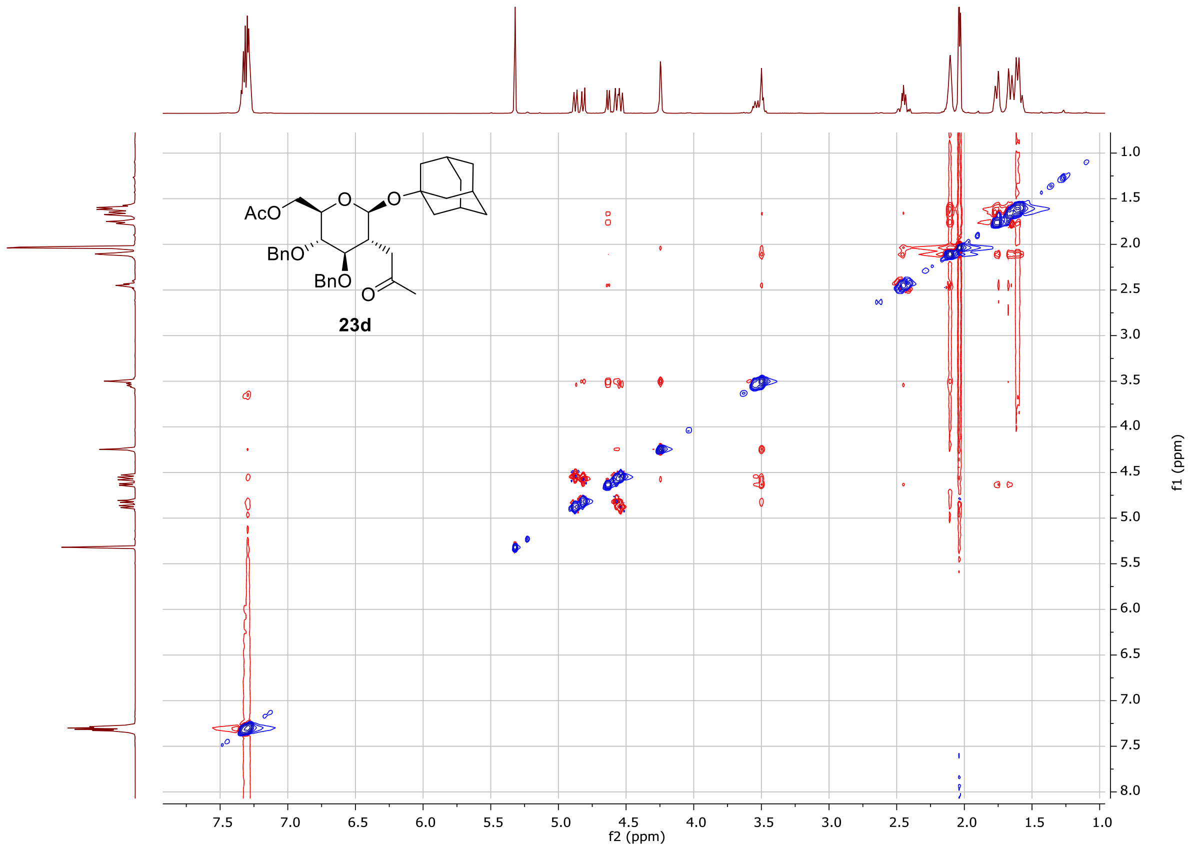

Supplementary Figure 342. NOESY spectra for **23d**

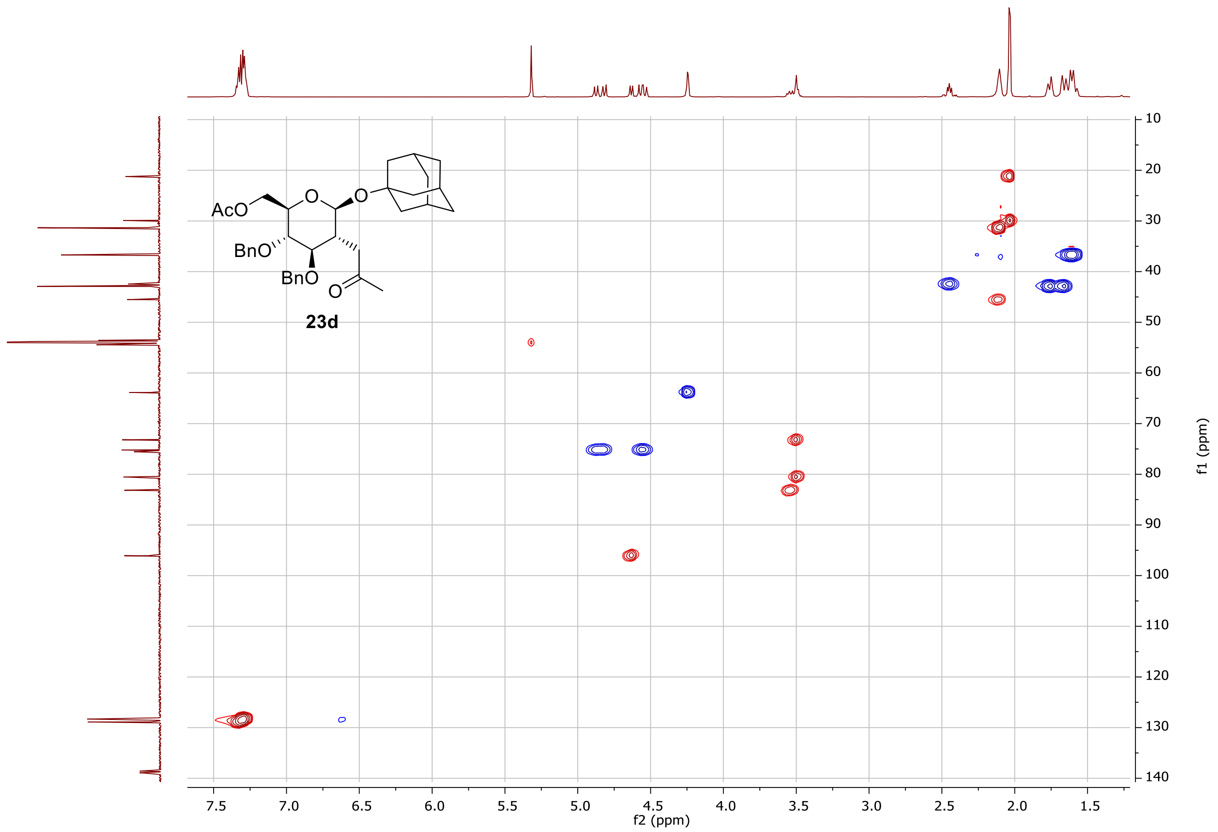

Supplementary Figure 343. HSQC spectra for **23d**

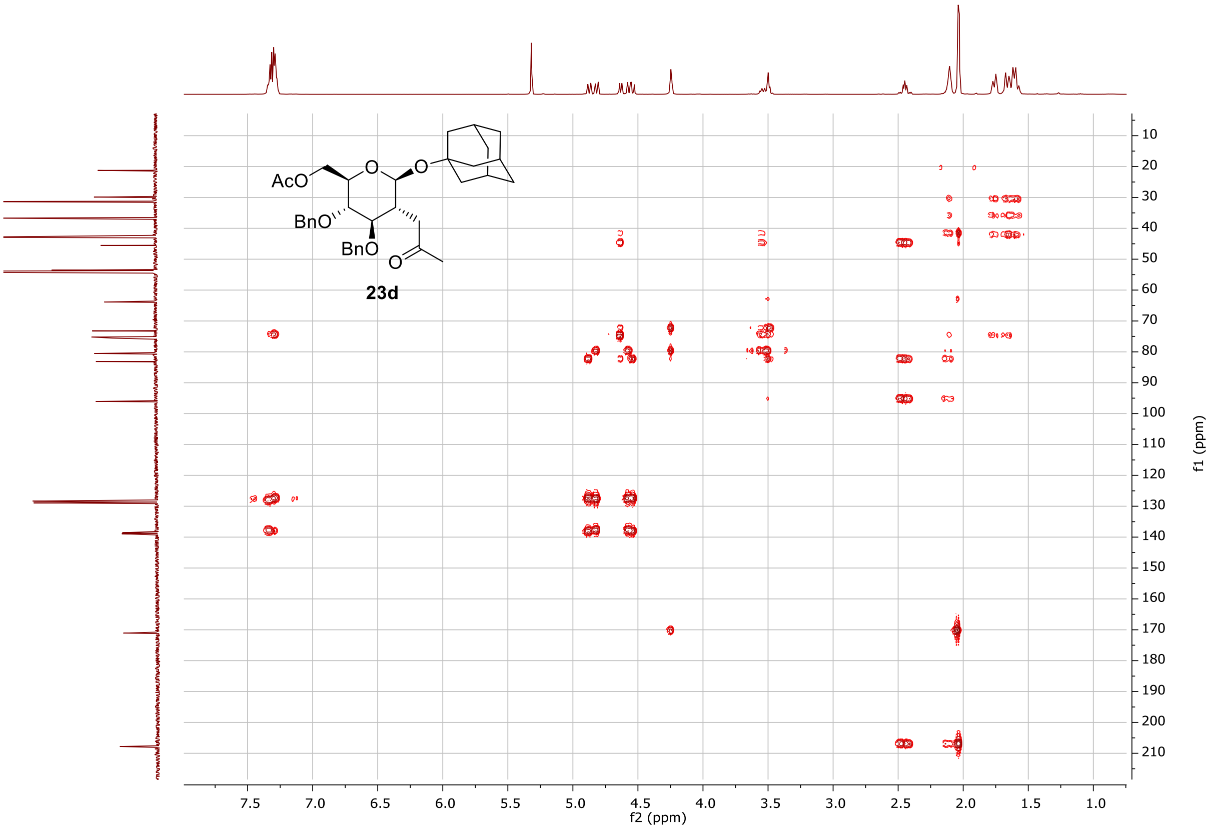

Supplementary Figure 344. HMBC spectra for **23d**

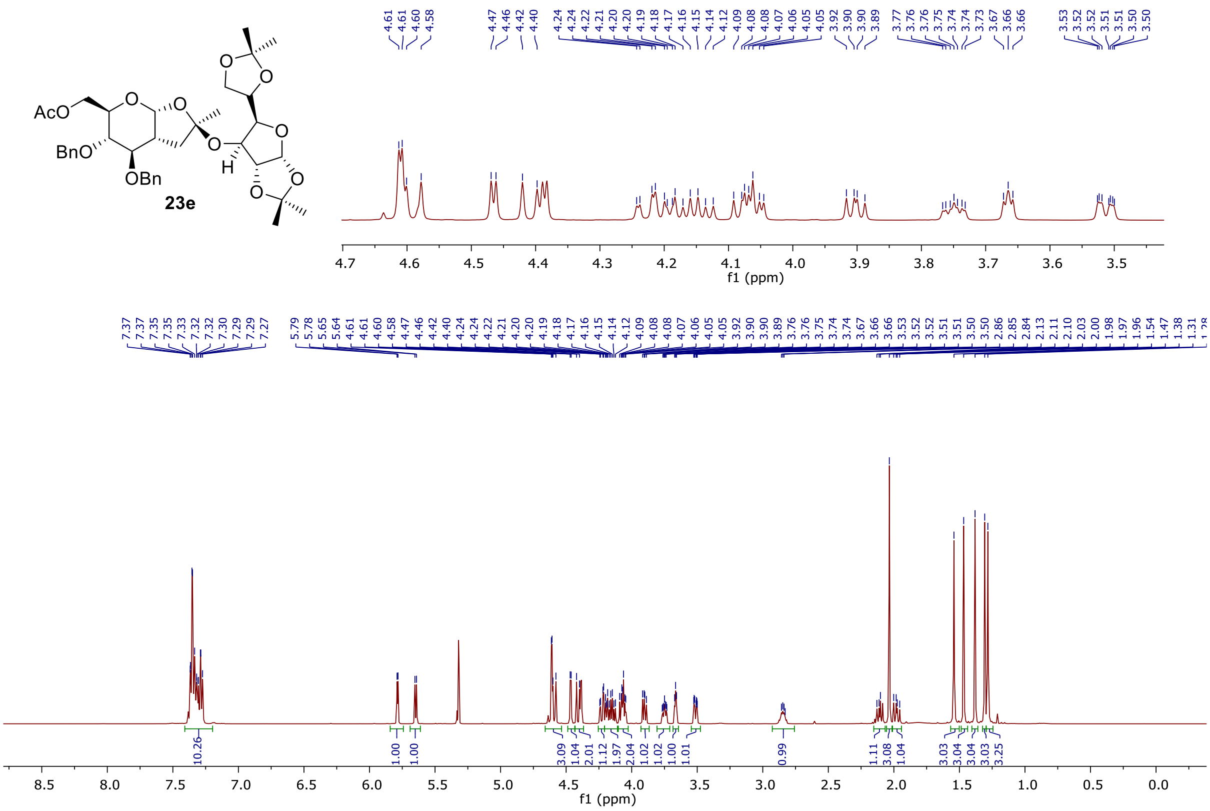

Supplementary Figure 345. ^{1}H spectra for **23e**

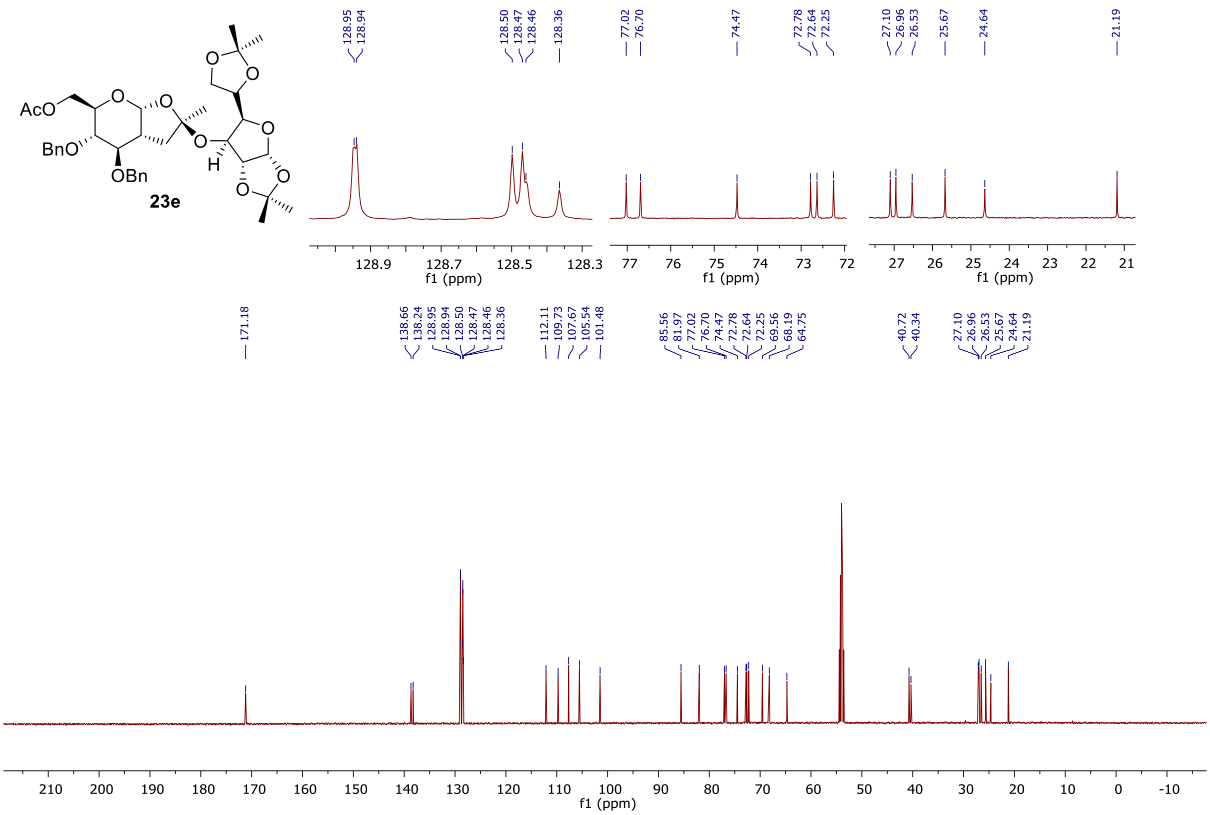

Supplementary Figure 346. ^{13}C spectra for **23e**

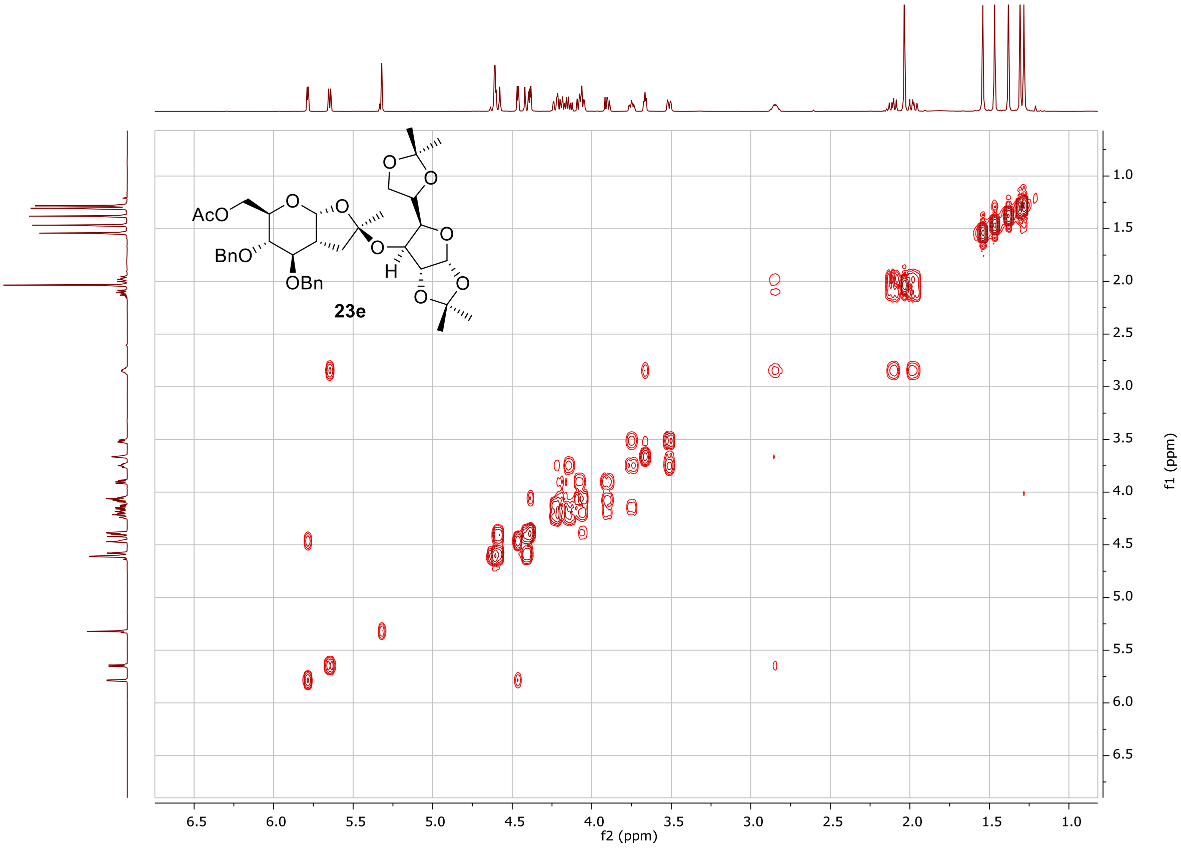

Supplementary Figure 347. COSY spectra for **23e**

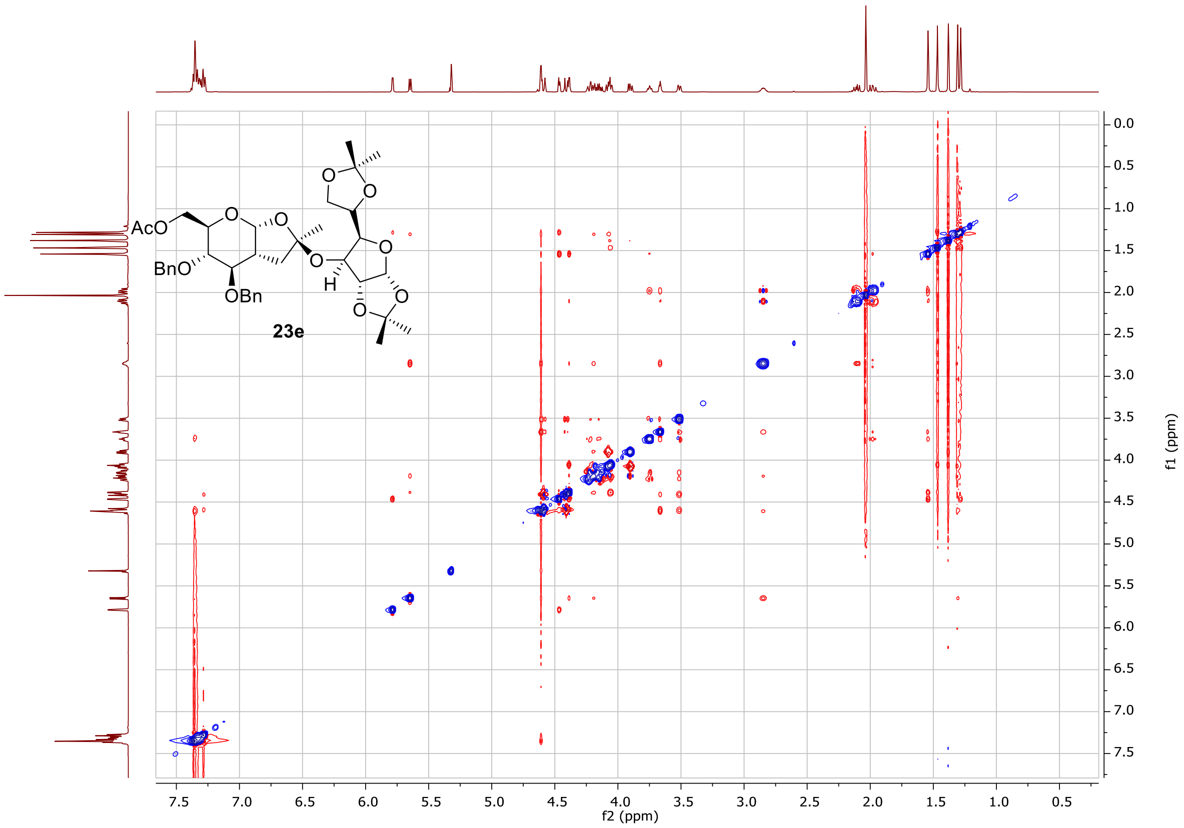

Supplementary Figure 348. NOESY spectra for **23e**

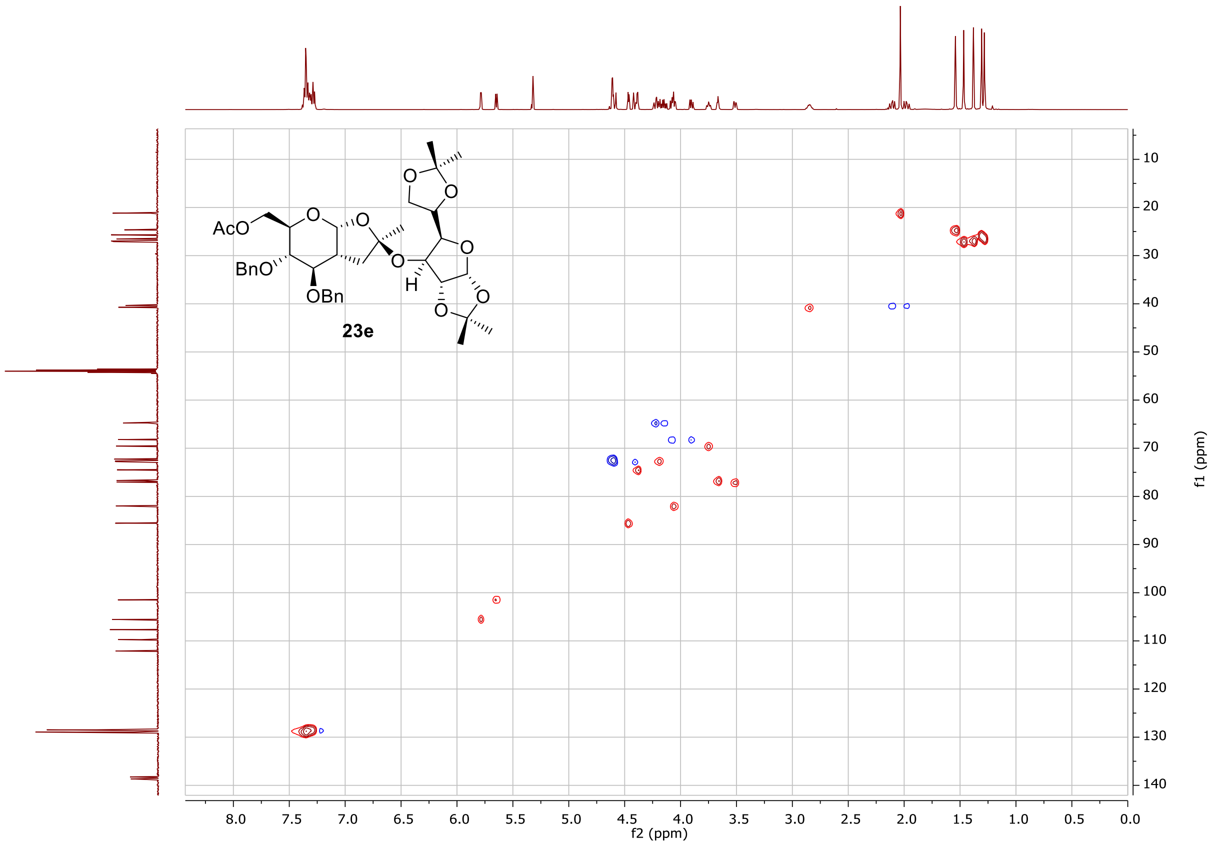

Supplementary Figure 349. HSQC spectra for **23e**

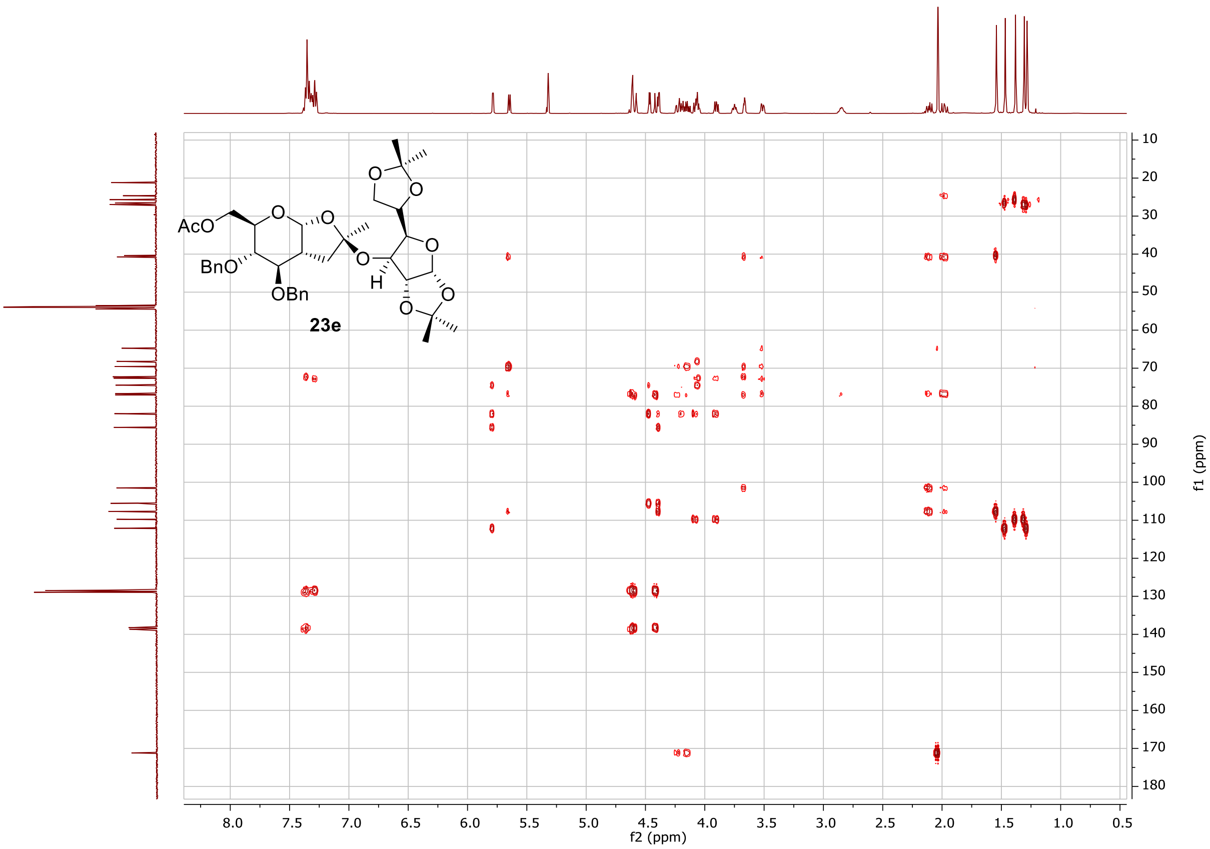

Supplementary Figure 350. HMBC spectra for **23e**

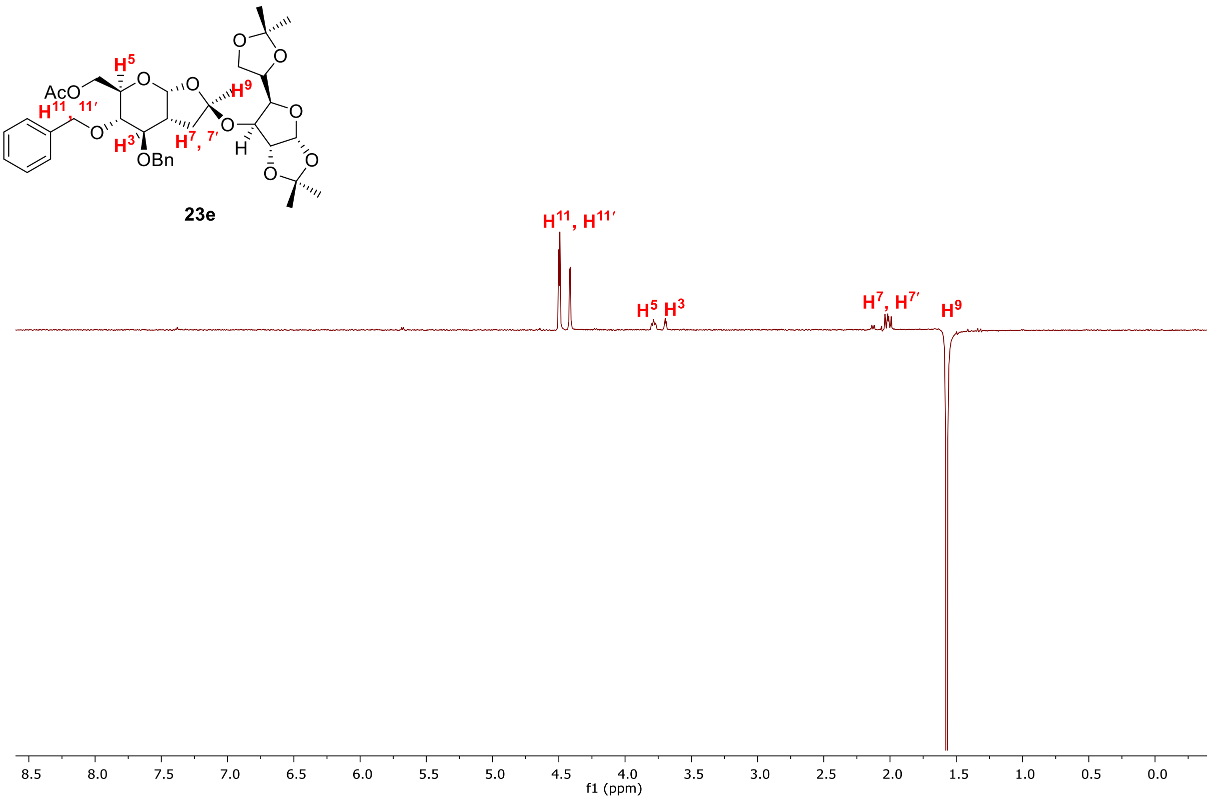

Supplementary Figure 351. 1D NOE spectra for **23e**

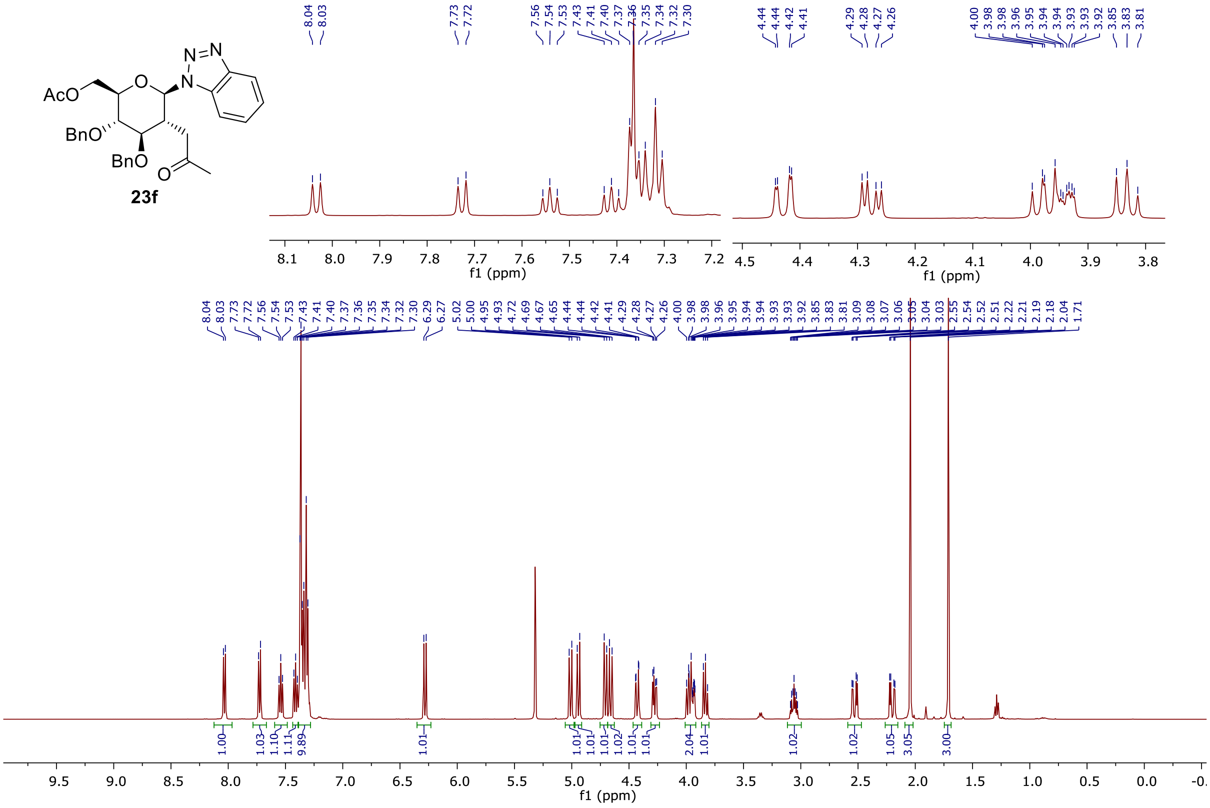

Supplementary Figure 352. ^{1}H spectra for **23f**

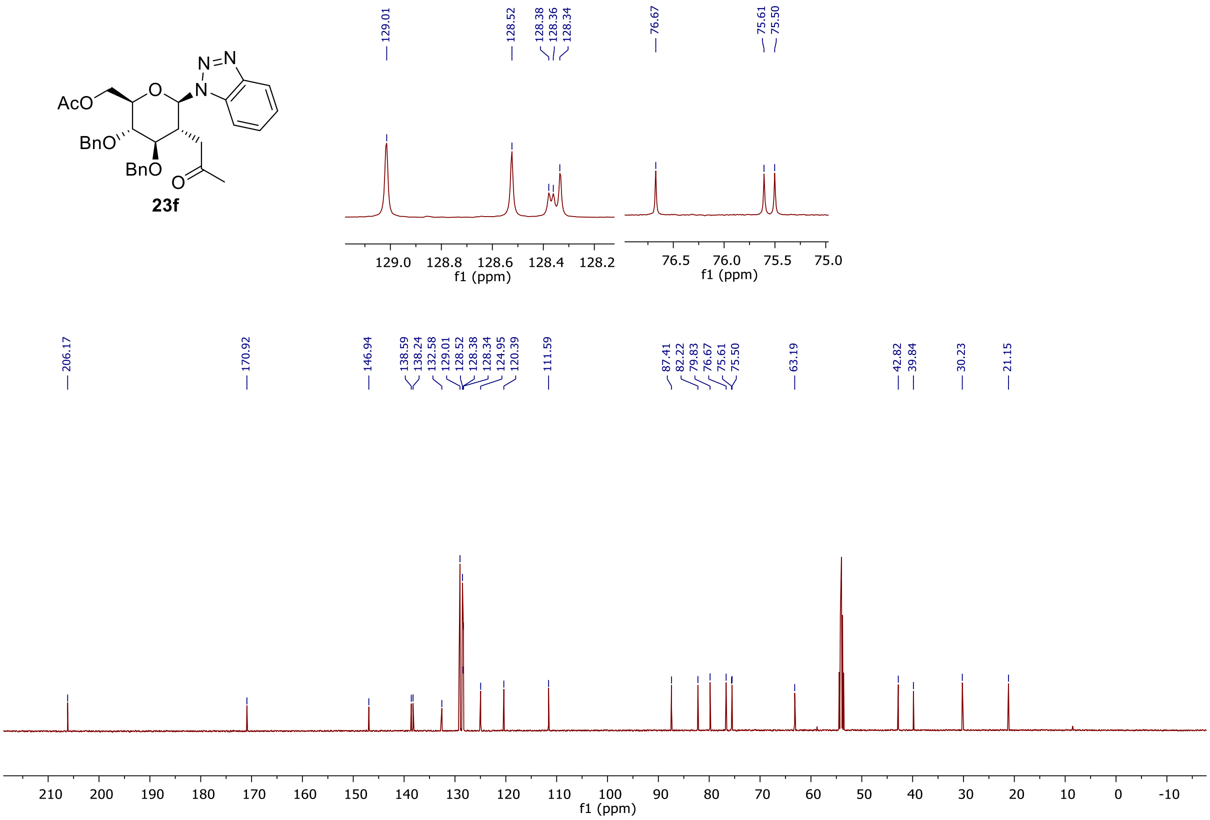

Supplementary Figure 353. ^{13}C spectra for **23f**

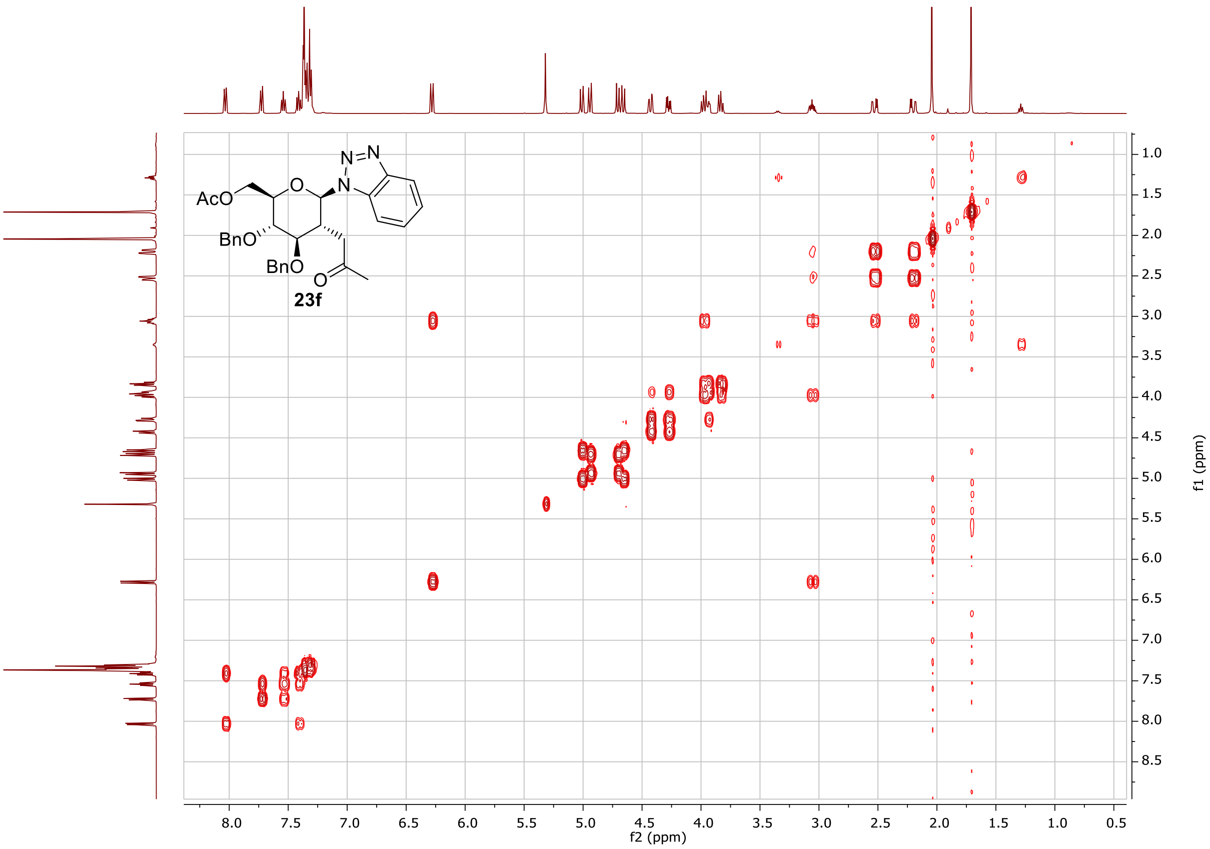

Supplementary Figure 354. COSY spectra for **23f**

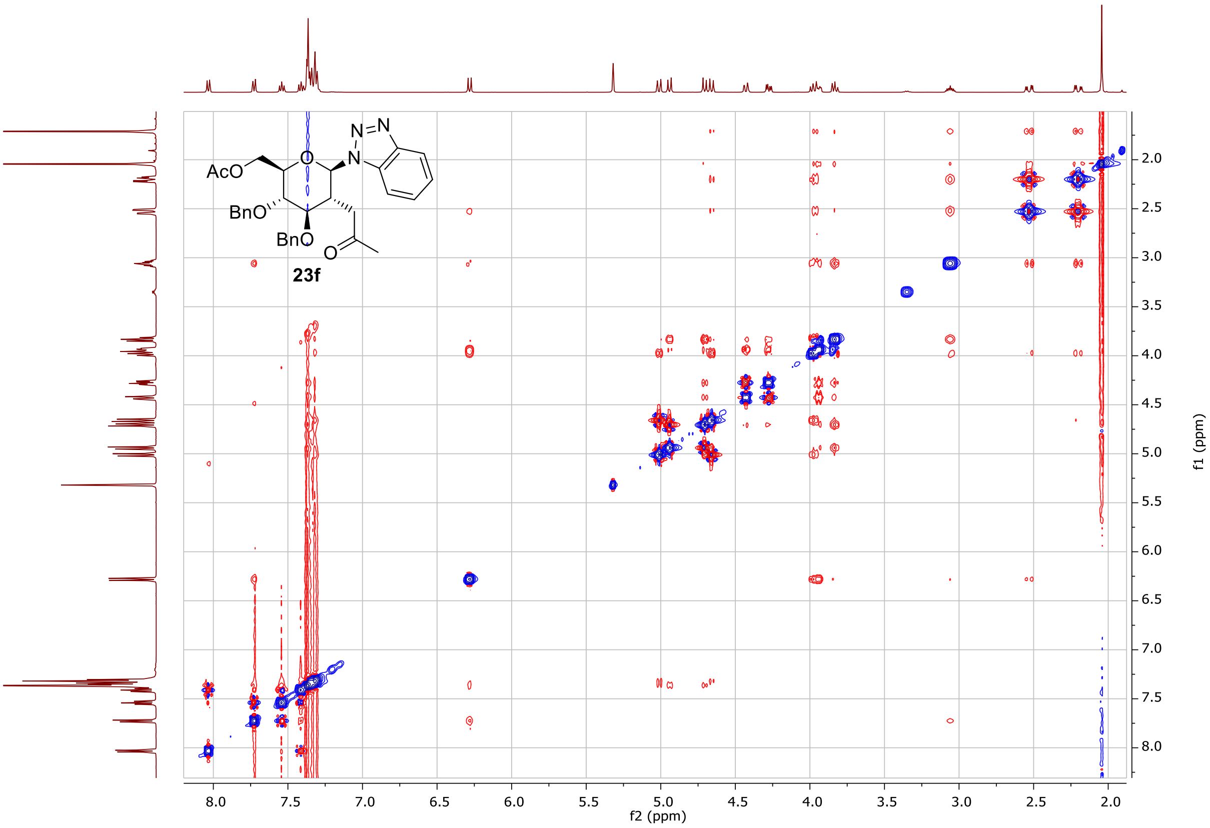

Supplementary Figure 355. NOESY spectra for **23f**

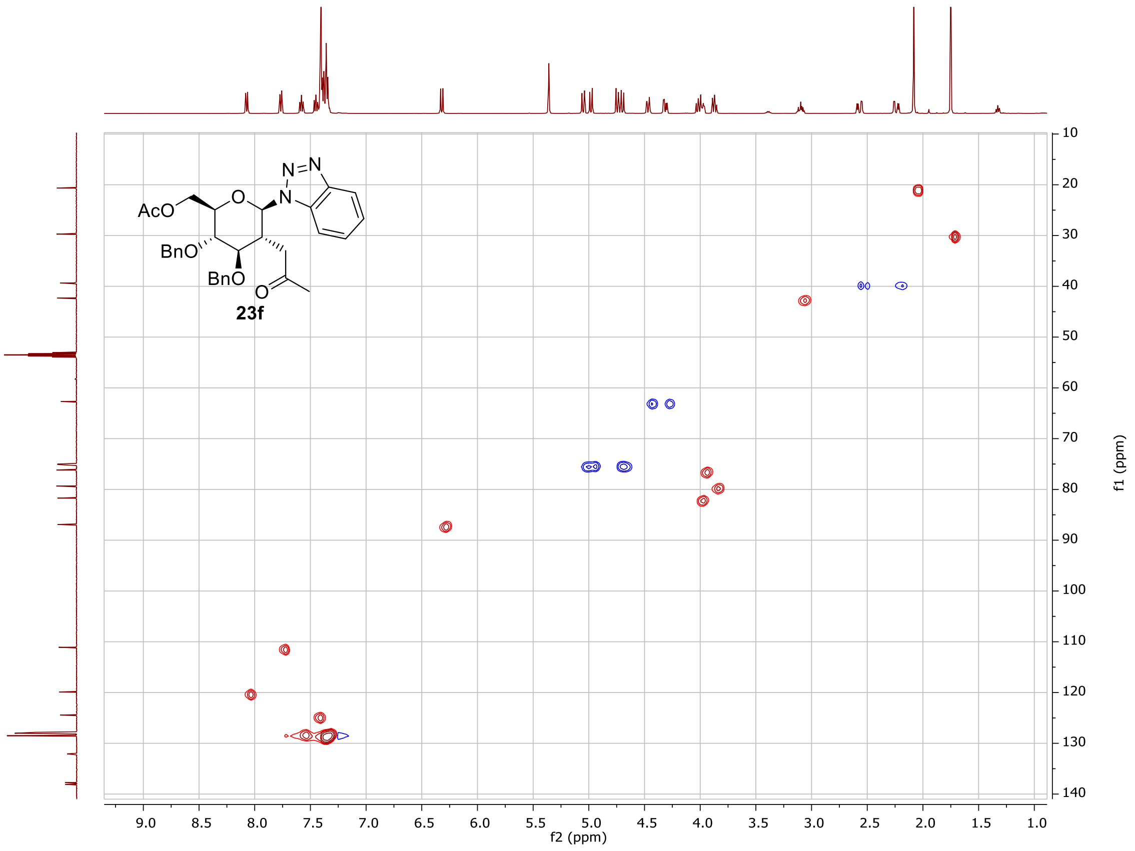

Supplementary Figure 356. HSQC spectra for **23f**

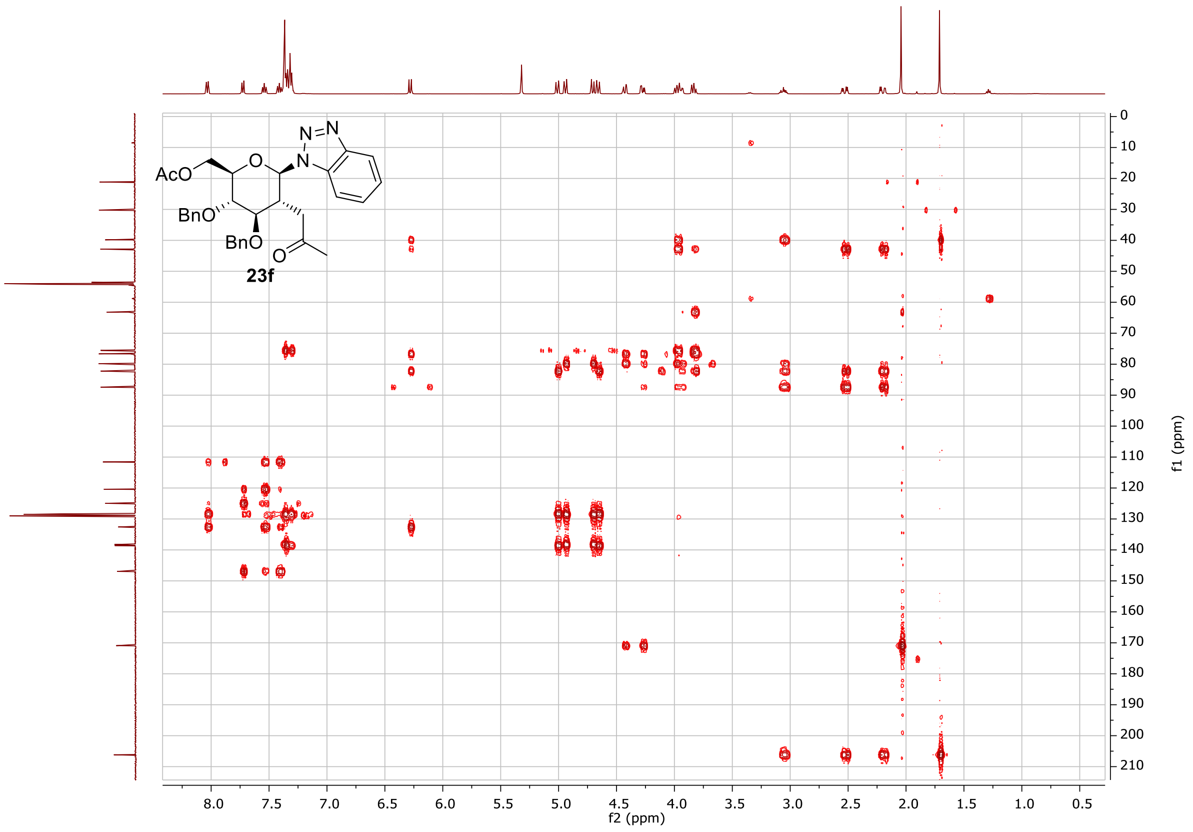

Supplementary Figure 357. HMBC spectra for **23f**

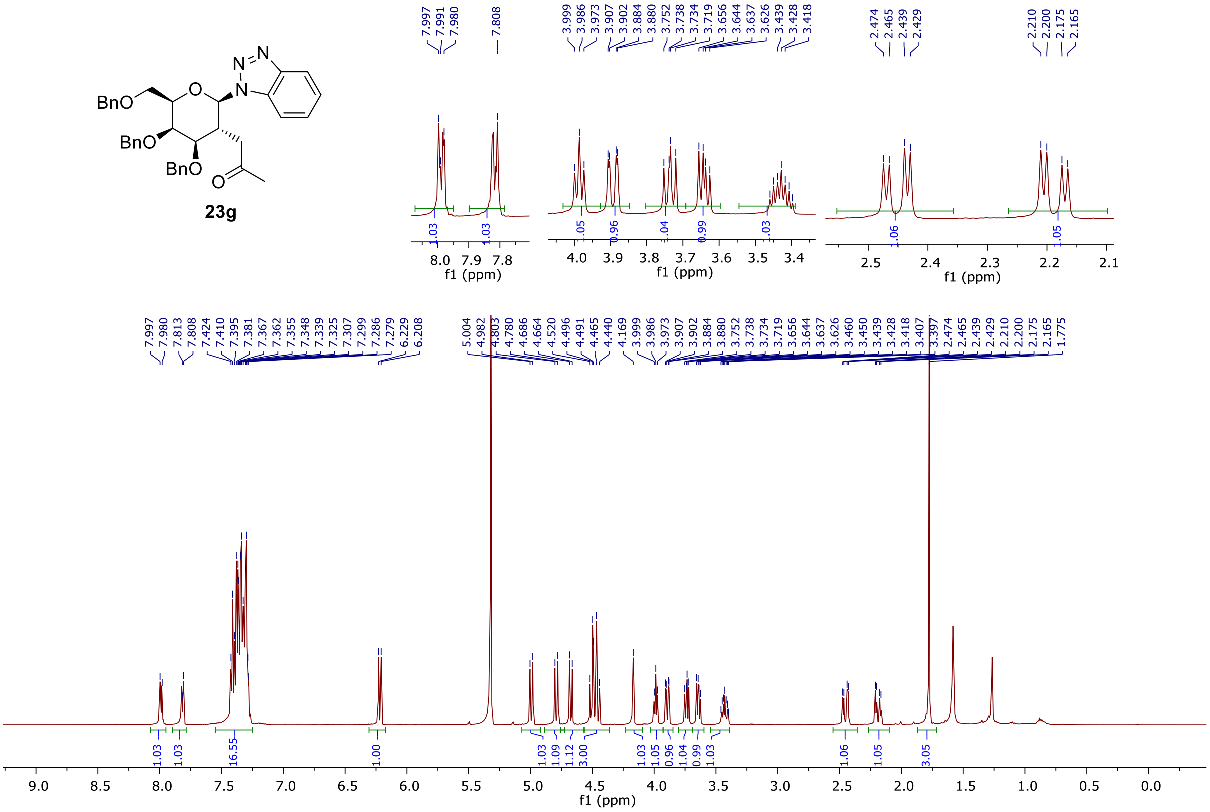

Supplementary Figure 358. ^{1}H spectra for **23g**

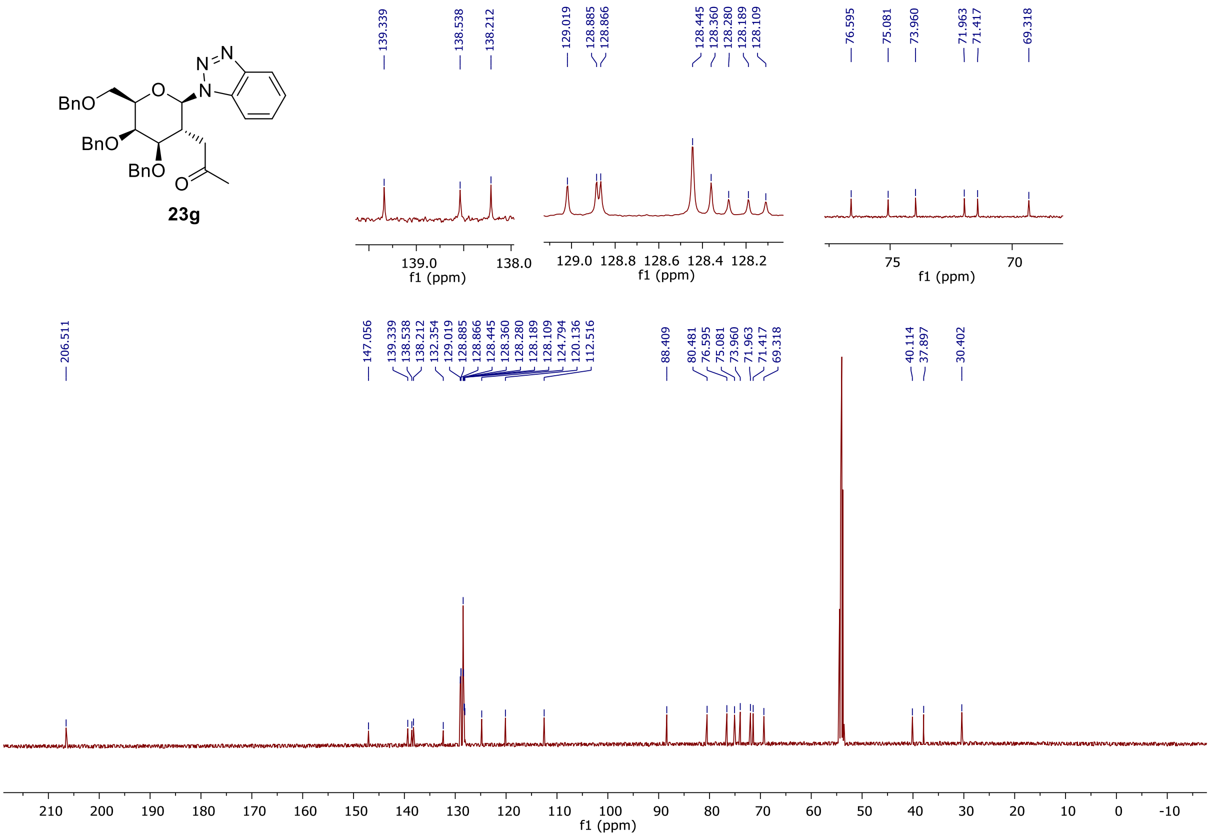

Supplementary Figure 359. ^{13}C spectra for **23g**

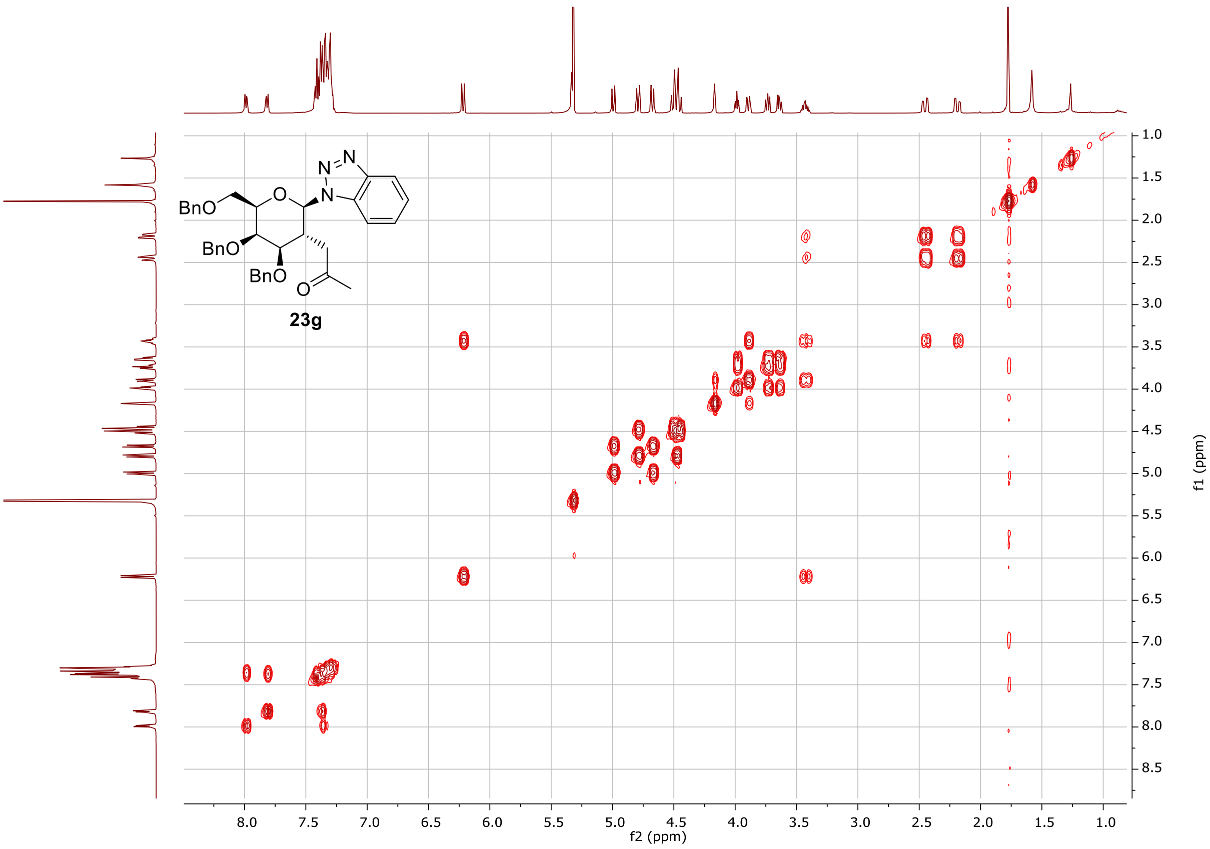

Supplementary Figure 360. COSY spectra for **23g**

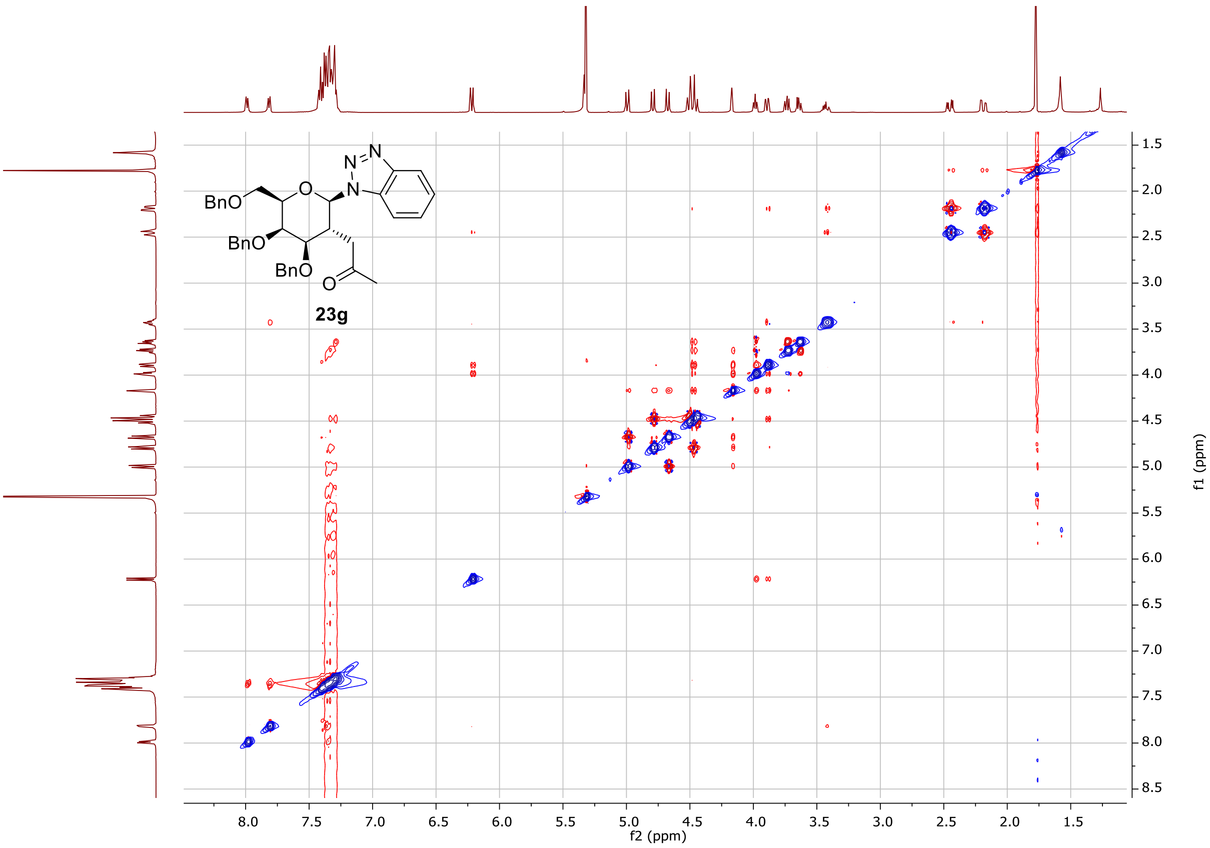

Supplementary Figure 361. NOESY spectra for **23g**

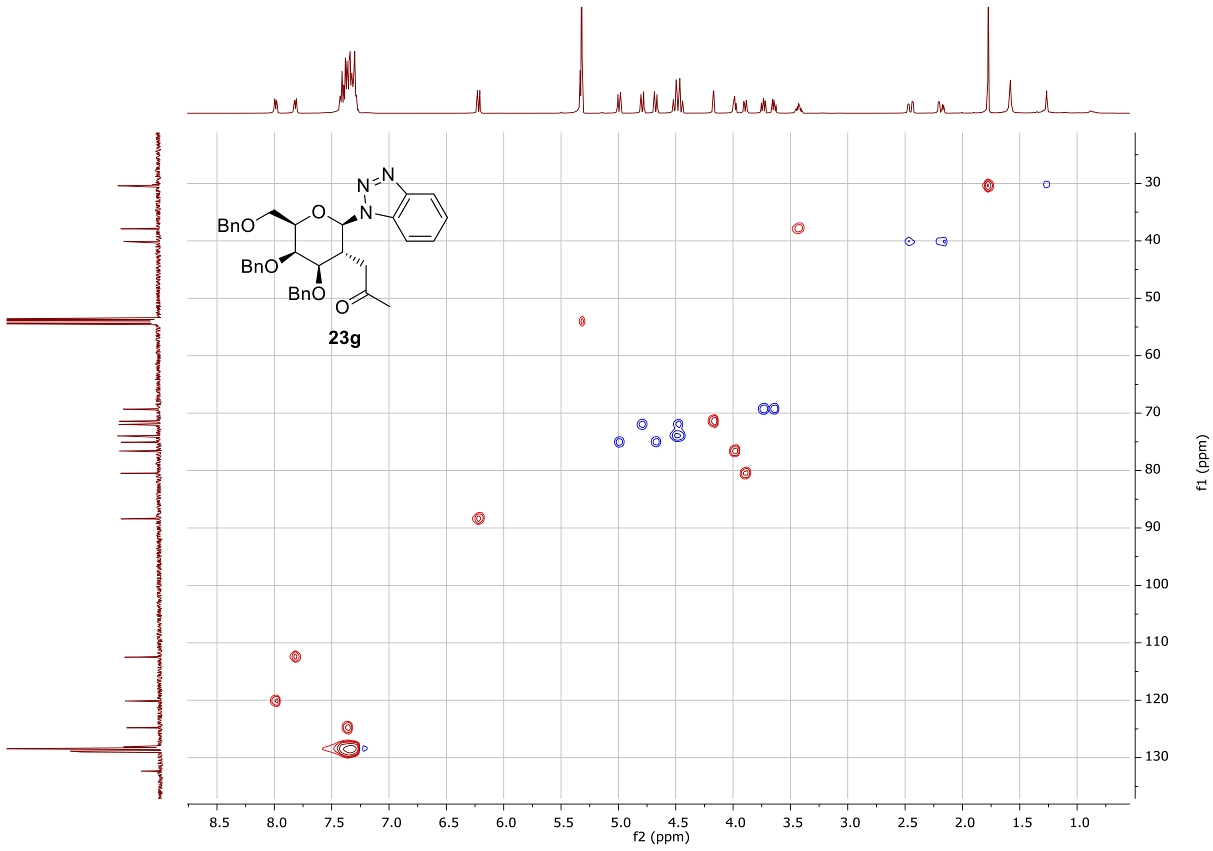

Supplementary Figure 362. HSQC spectra for **23g**

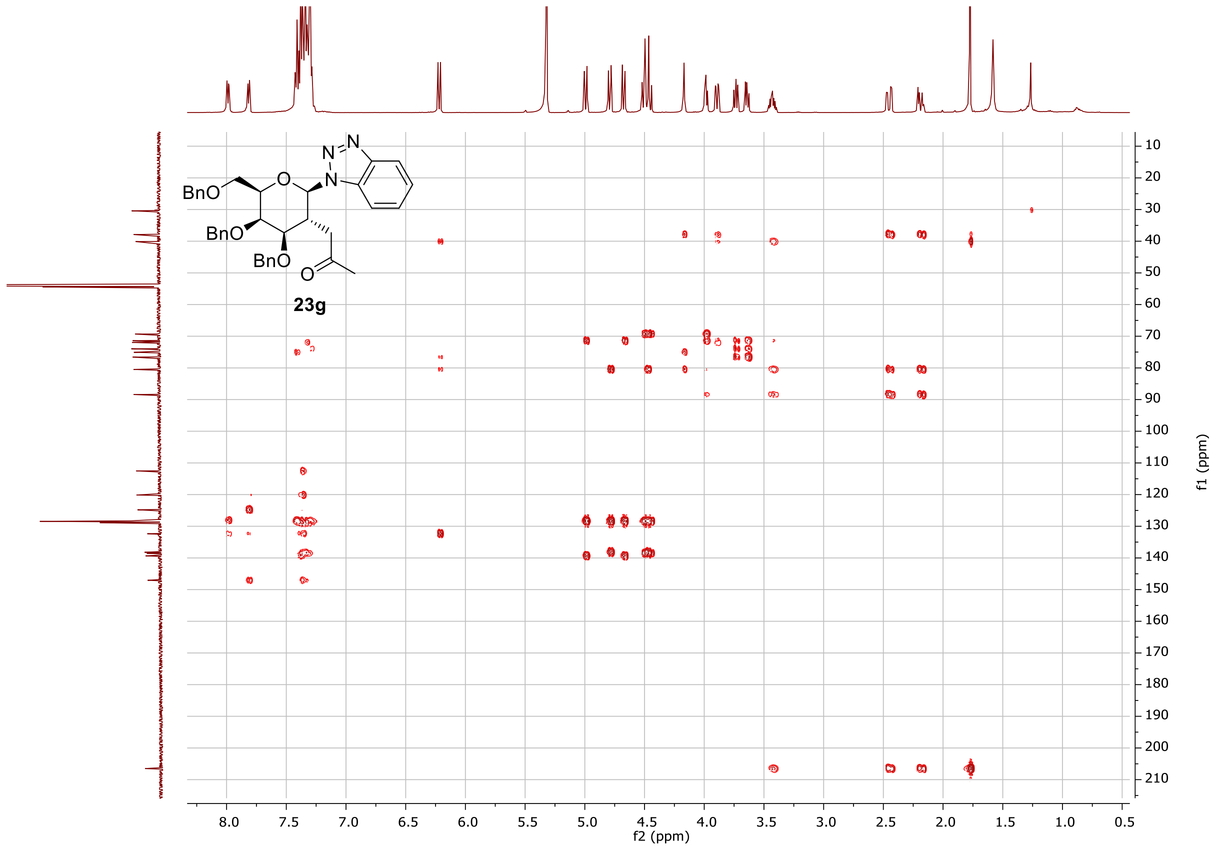

Supplementary Figure 363. HMBC spectra for **23g**

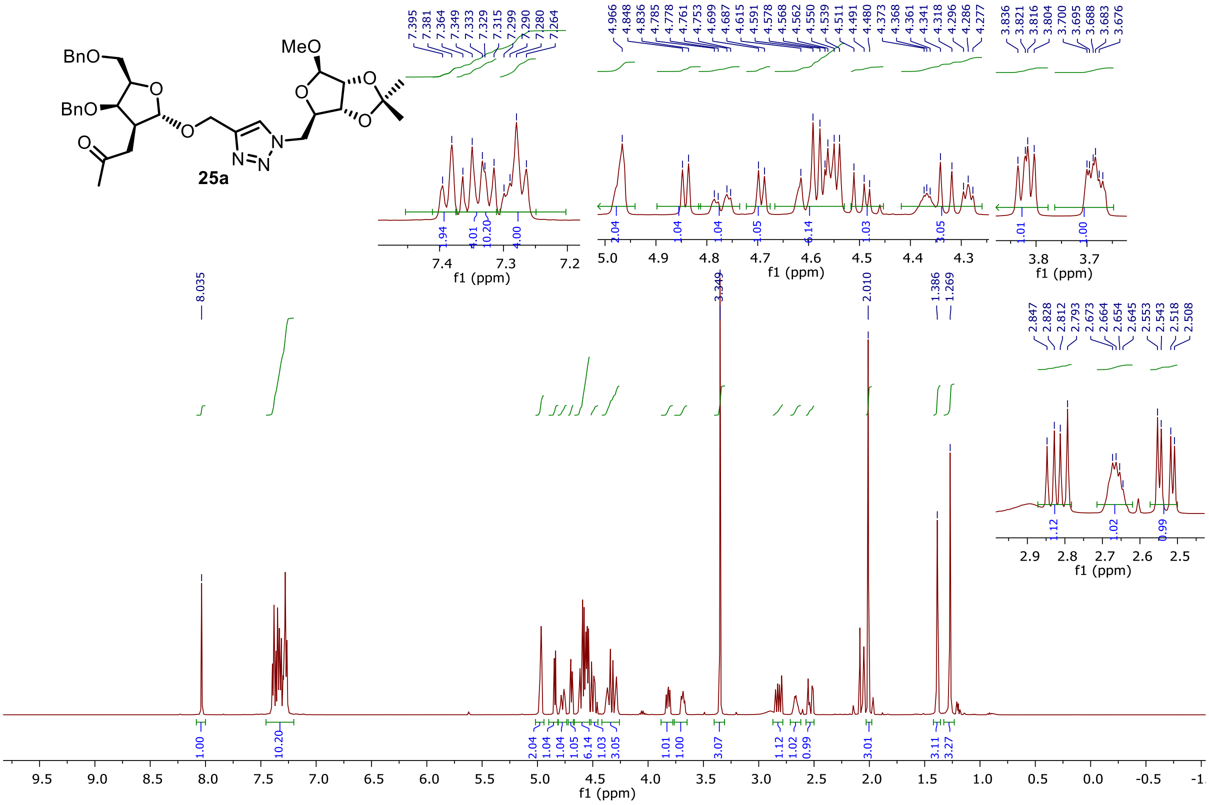

Supplementary Figure 364. ^{1}H spectra for **25a**

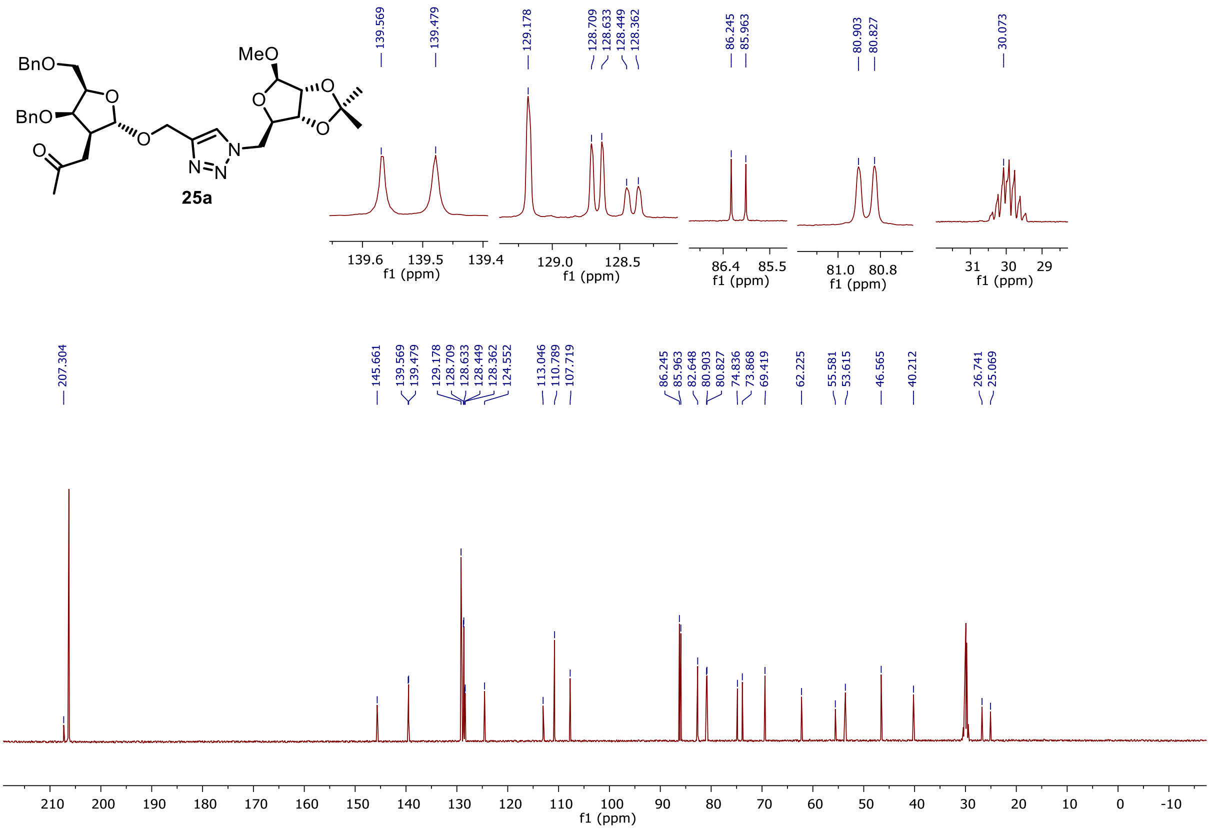

Supplementary Figure 365. ^{13}C spectra for **25a**

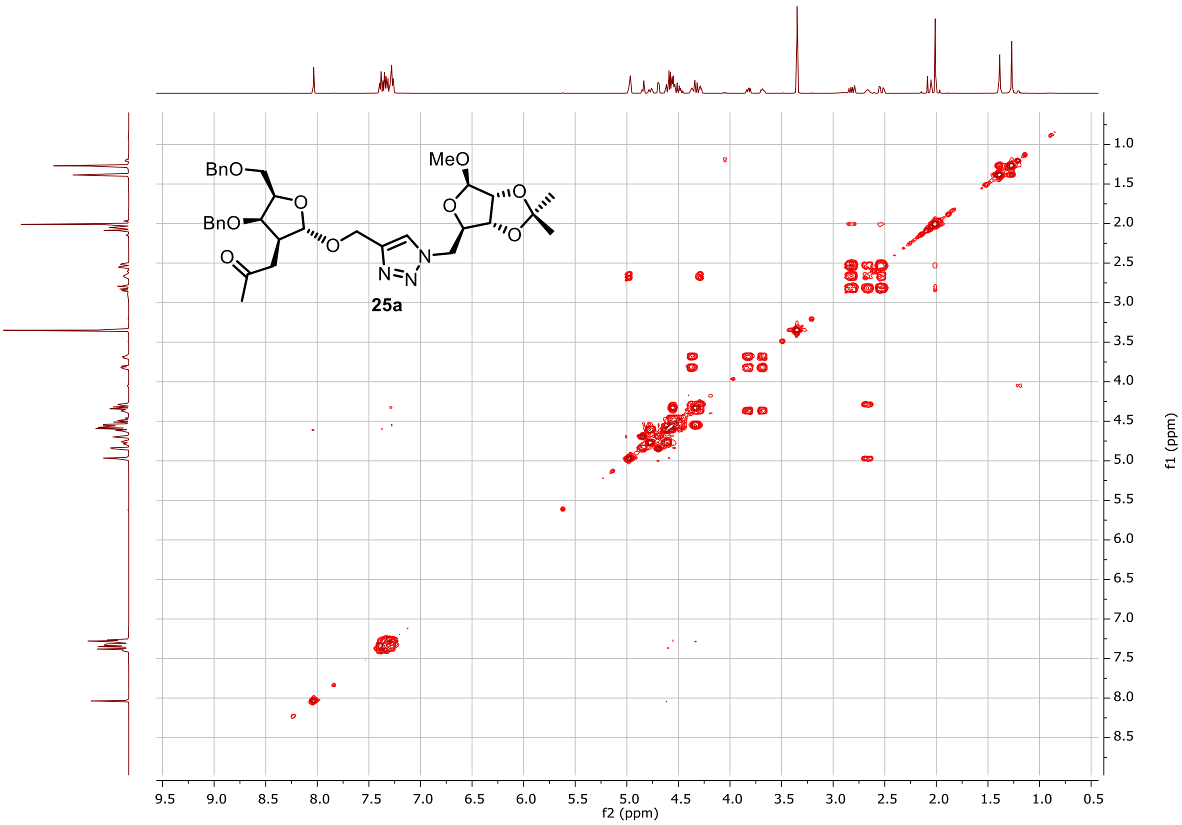

Supplementary Figure 366. COSY spectra for **25a**

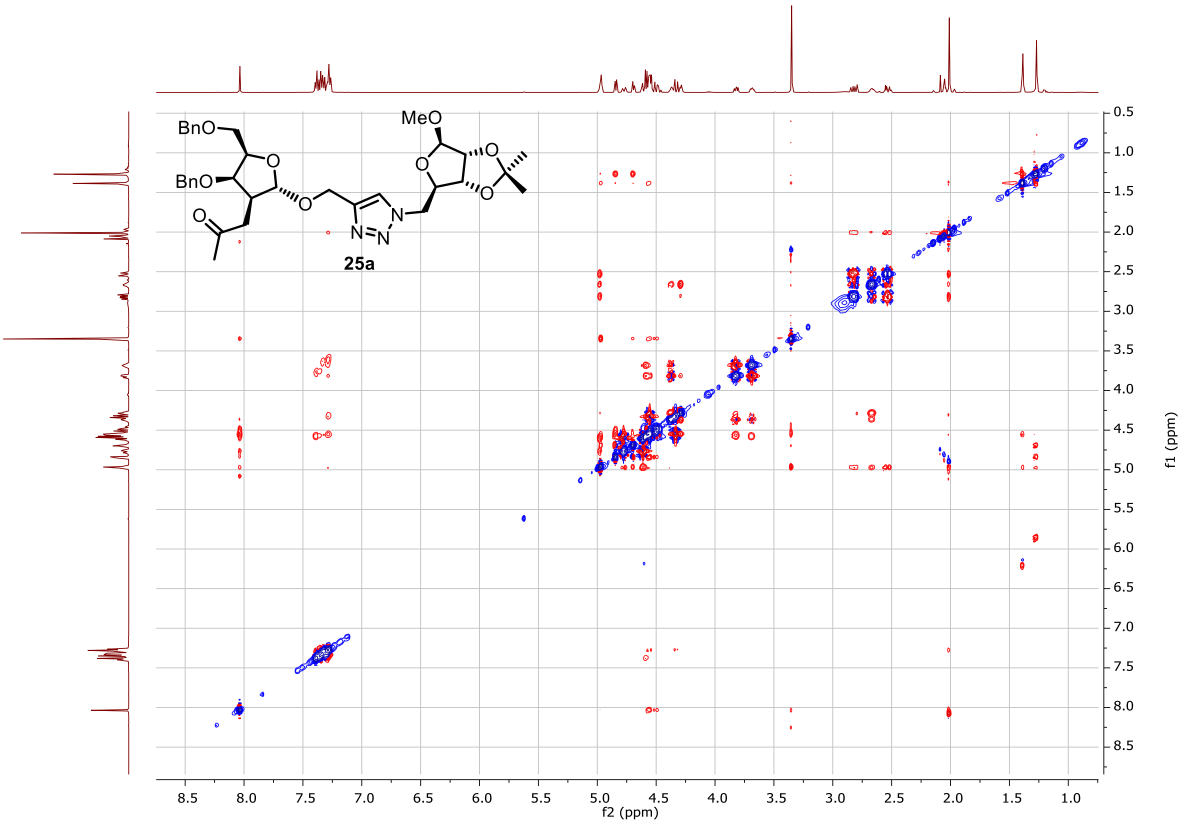

Supplementary Figure 367. NOESY spectra for **25a**

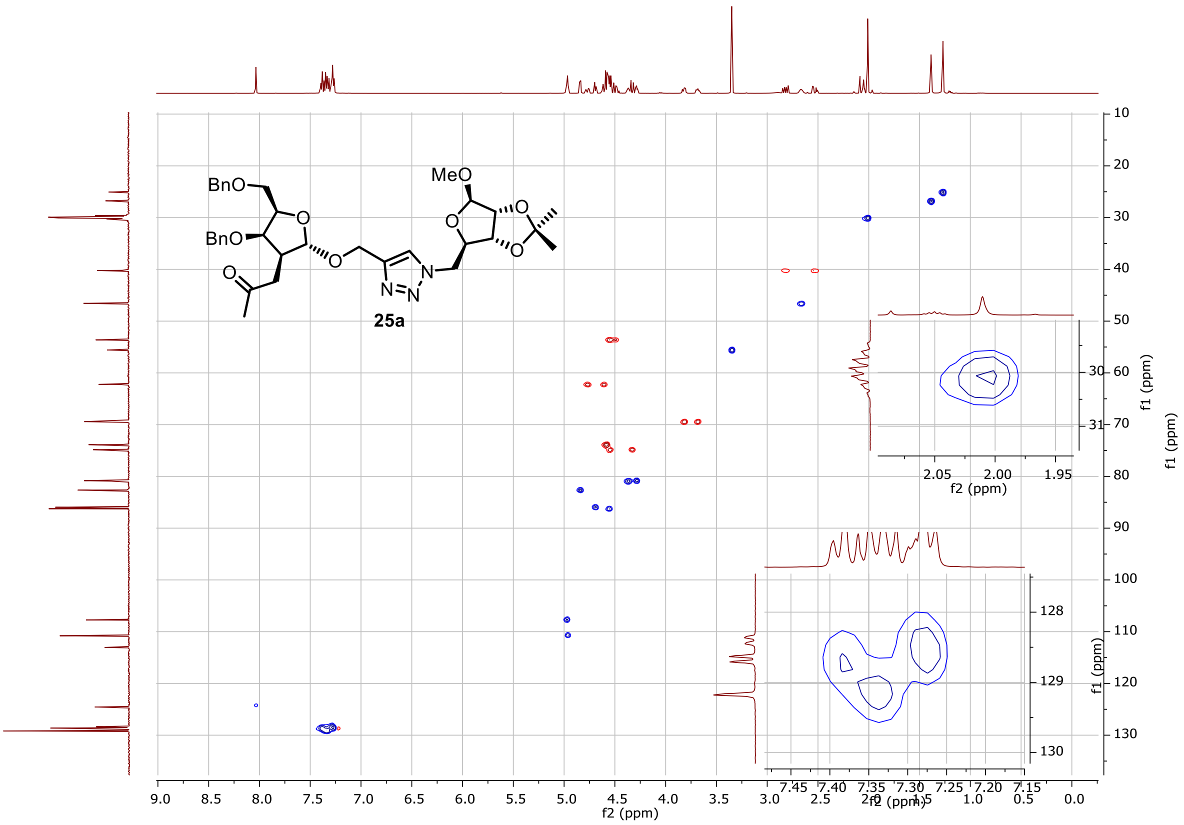

Supplementary Figure 368. HSQC spectra for **25a**

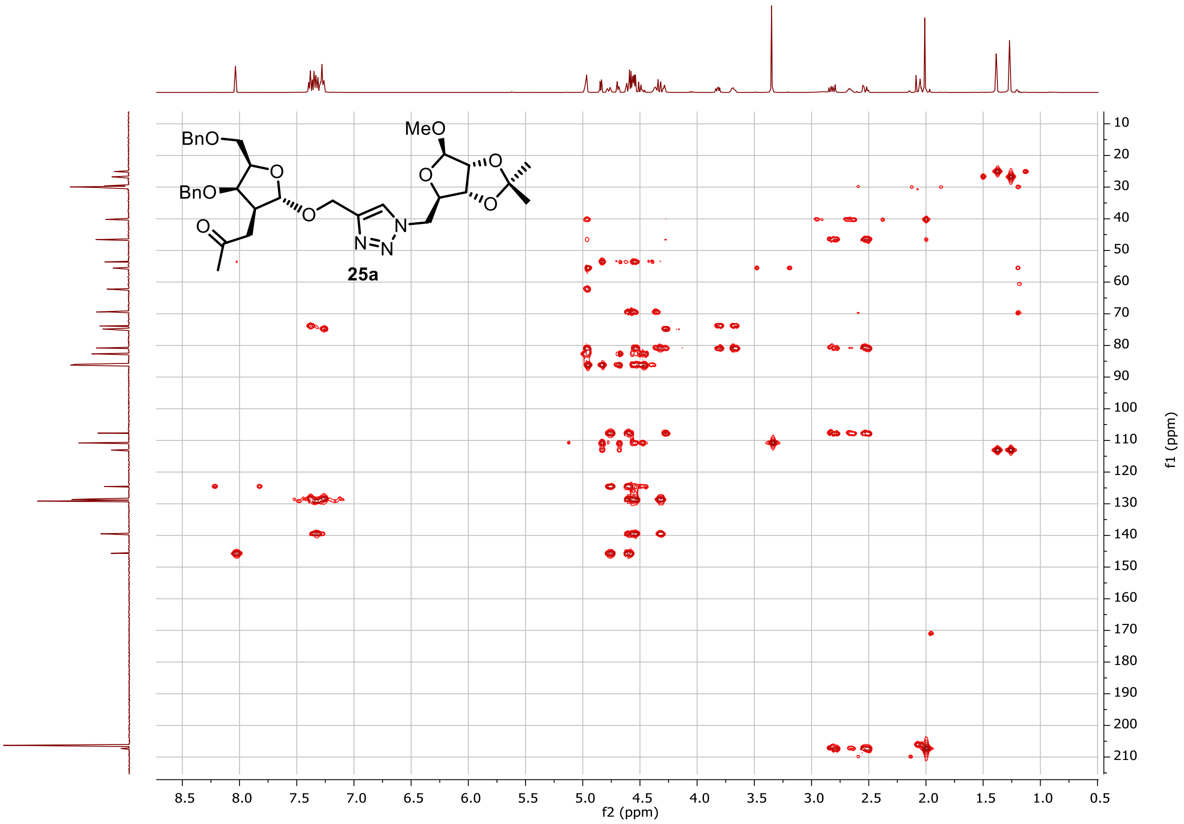

Supplementary Figure 369. HMBC spectra for **25a**

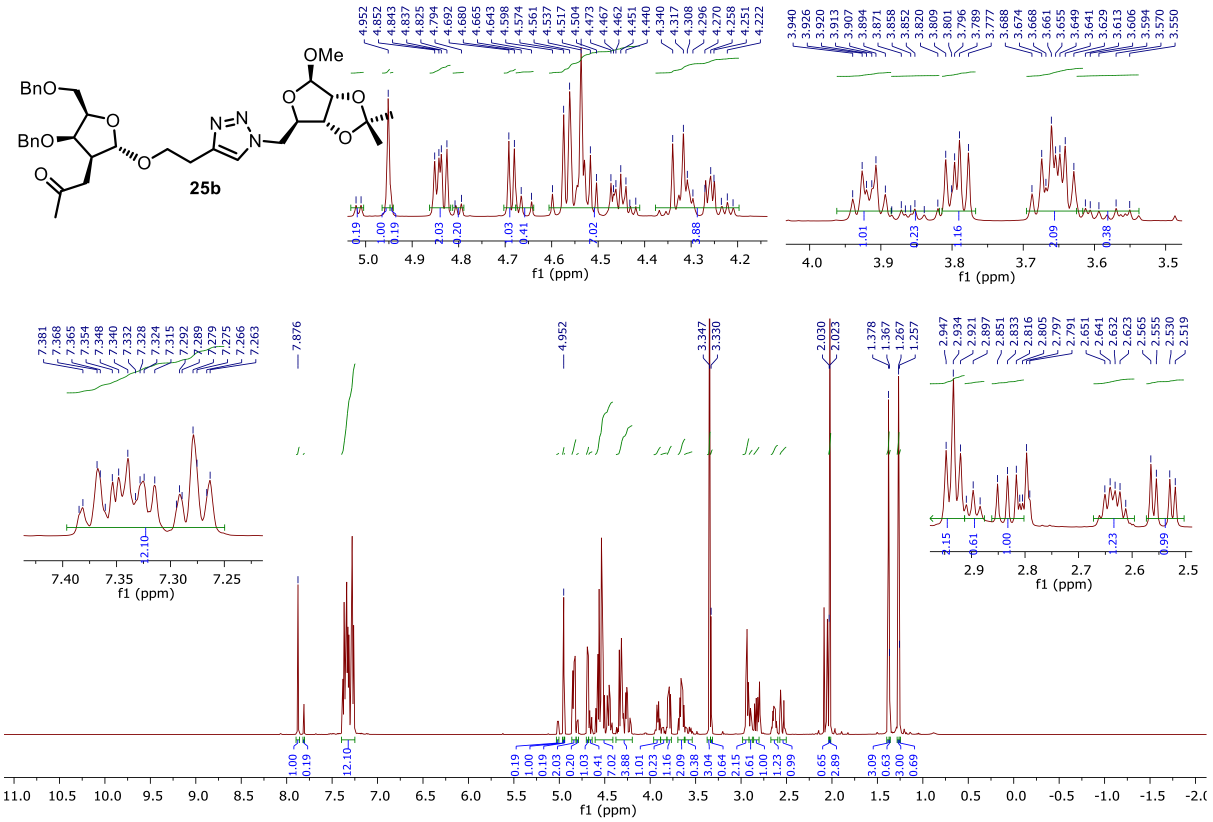

Supplementary Figure 370. ^{1}H spectra for **25b**

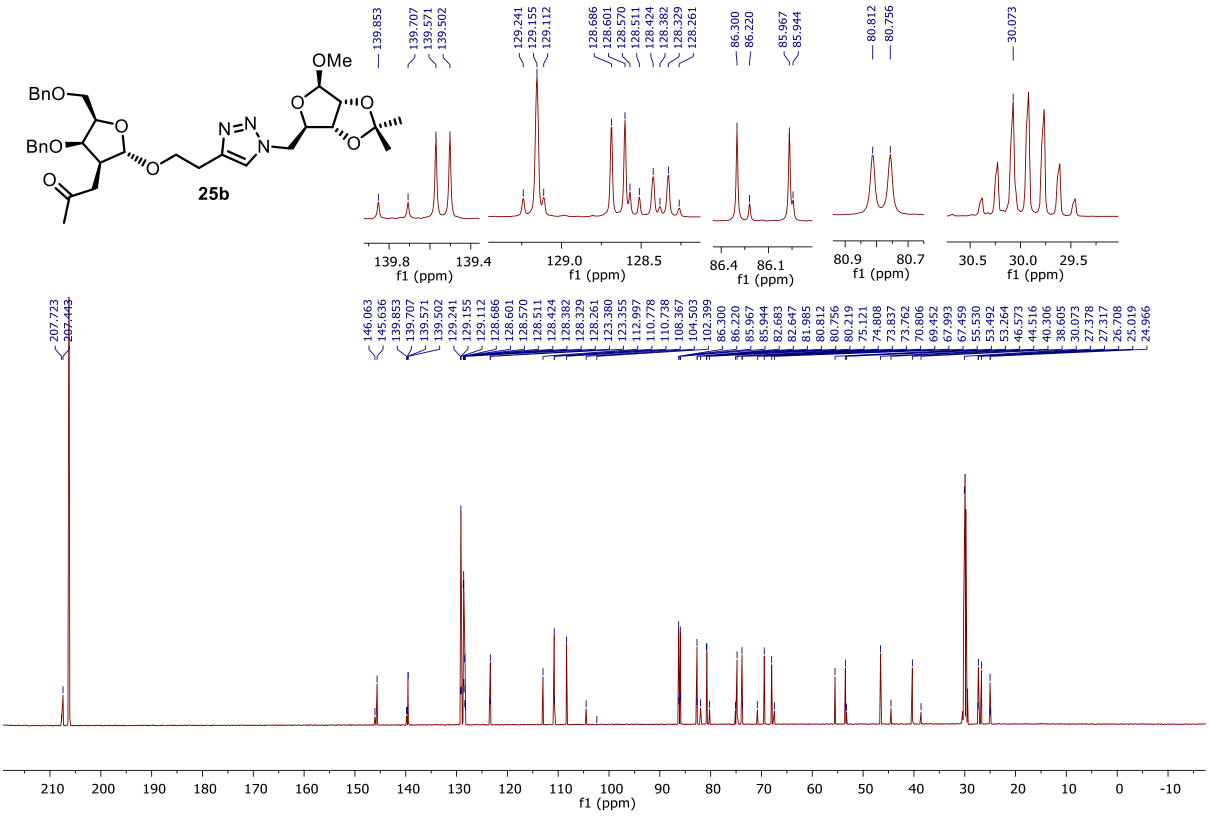

Supplementary Figure 371. ^{13}C spectra for **25b**

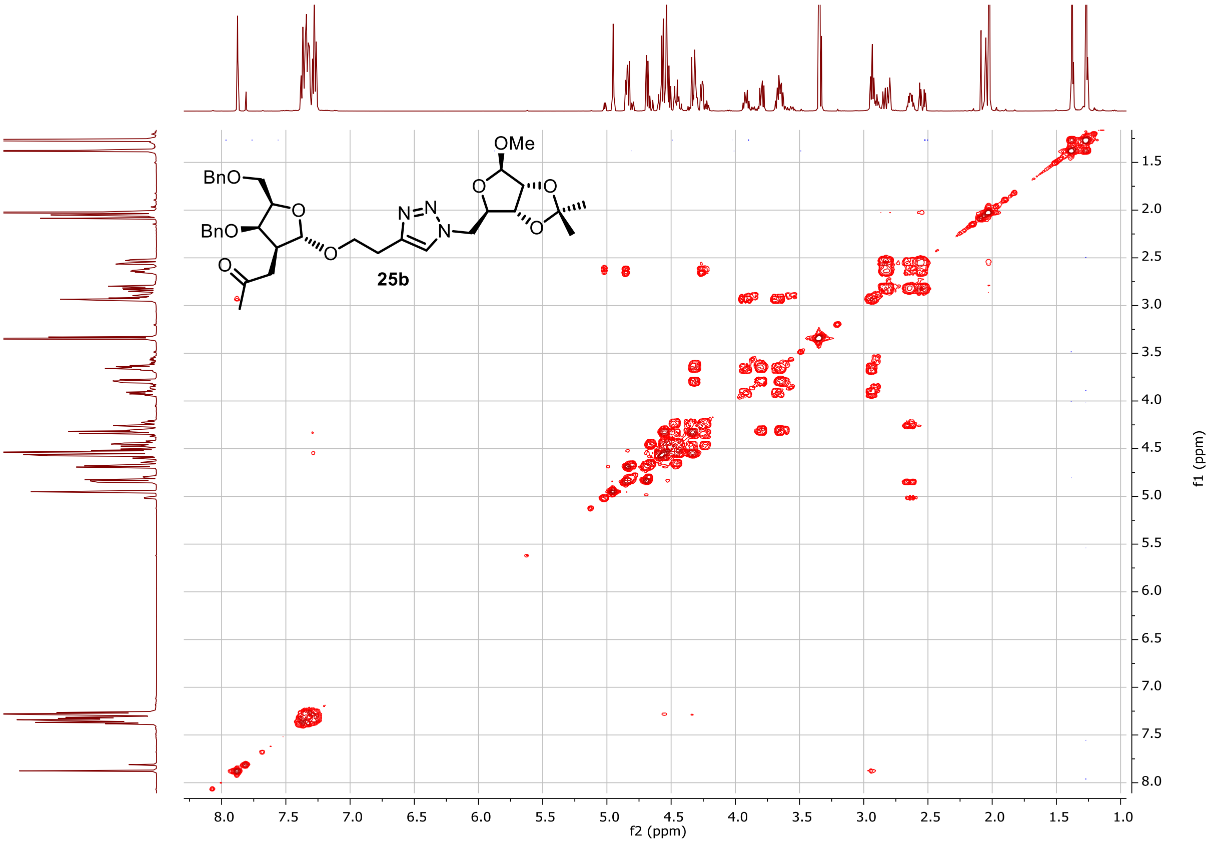

Supplementary Figure 372. COSY spectra for **25b**

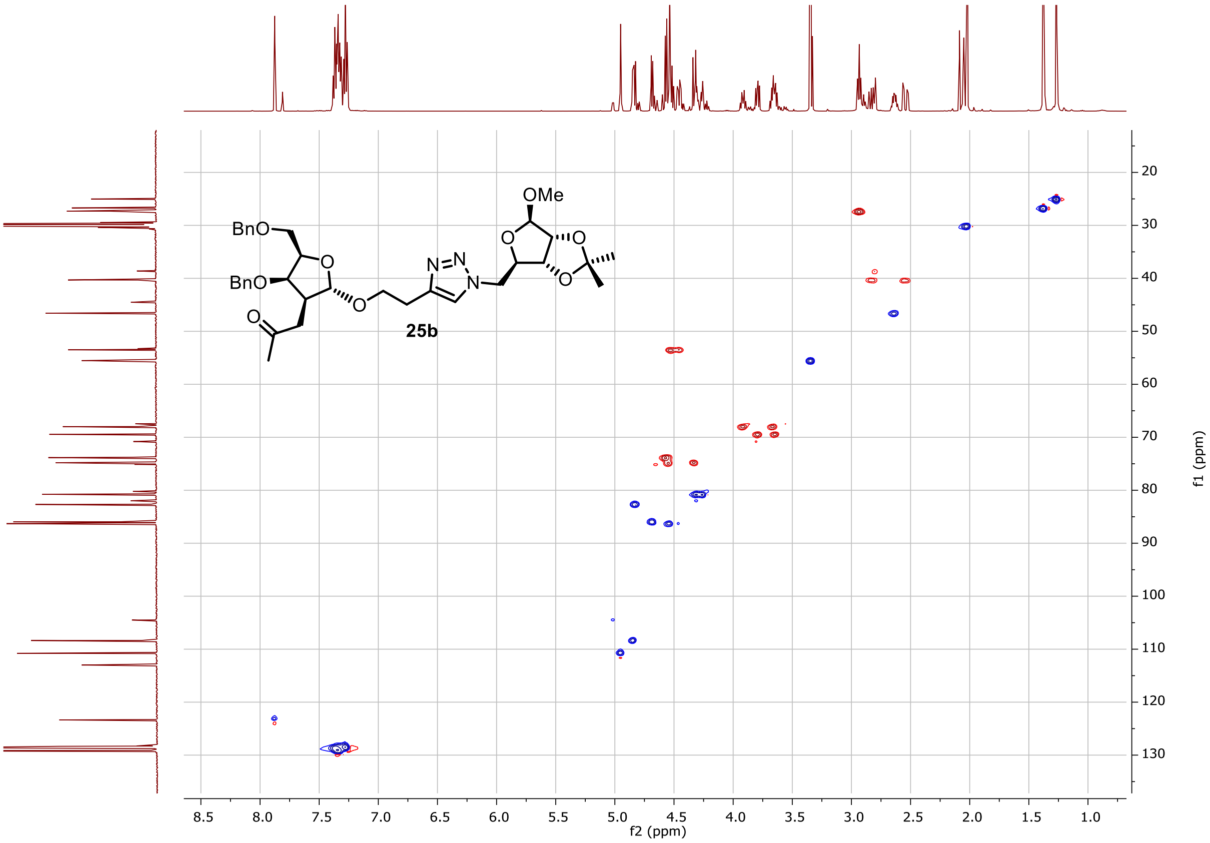

Supplementary Figure 373. HSQC spectra for **25b**

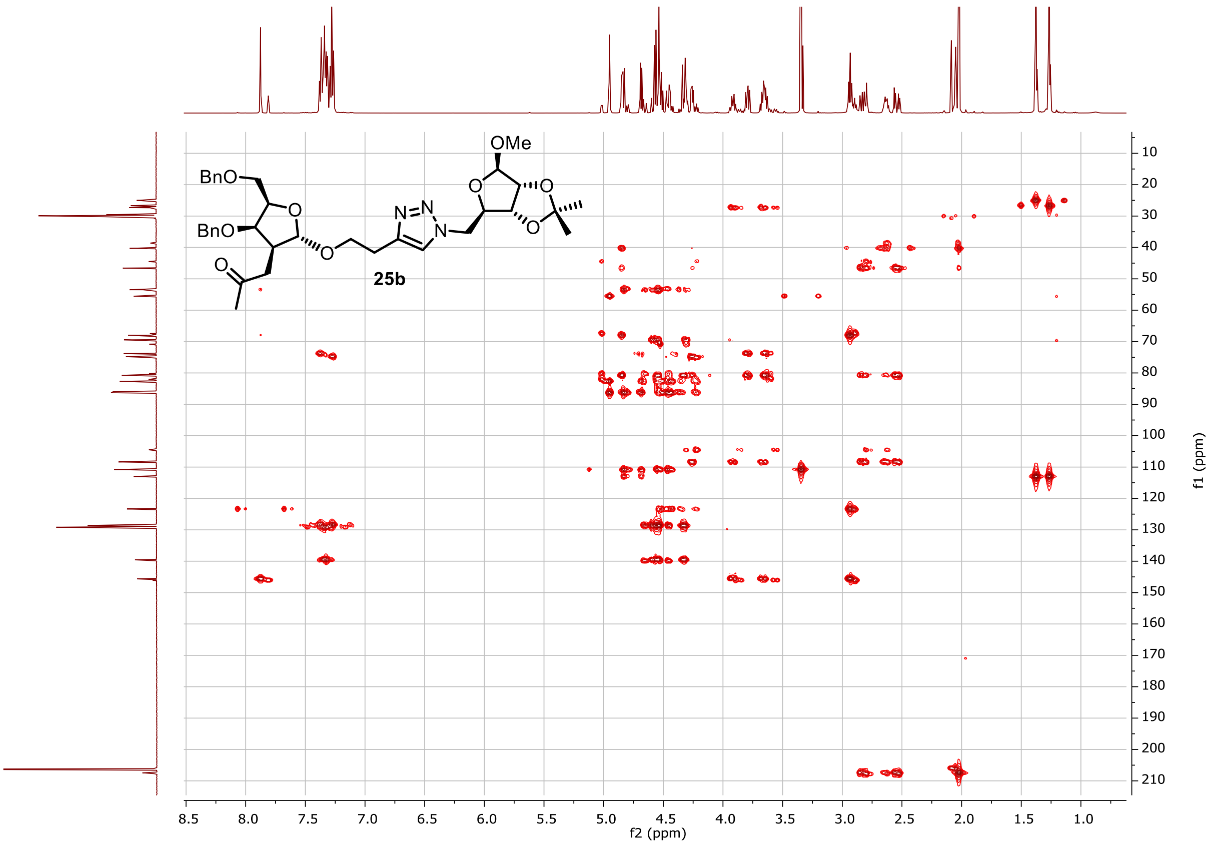

Supplementary Figure 374. HMBC spectra for **25b**

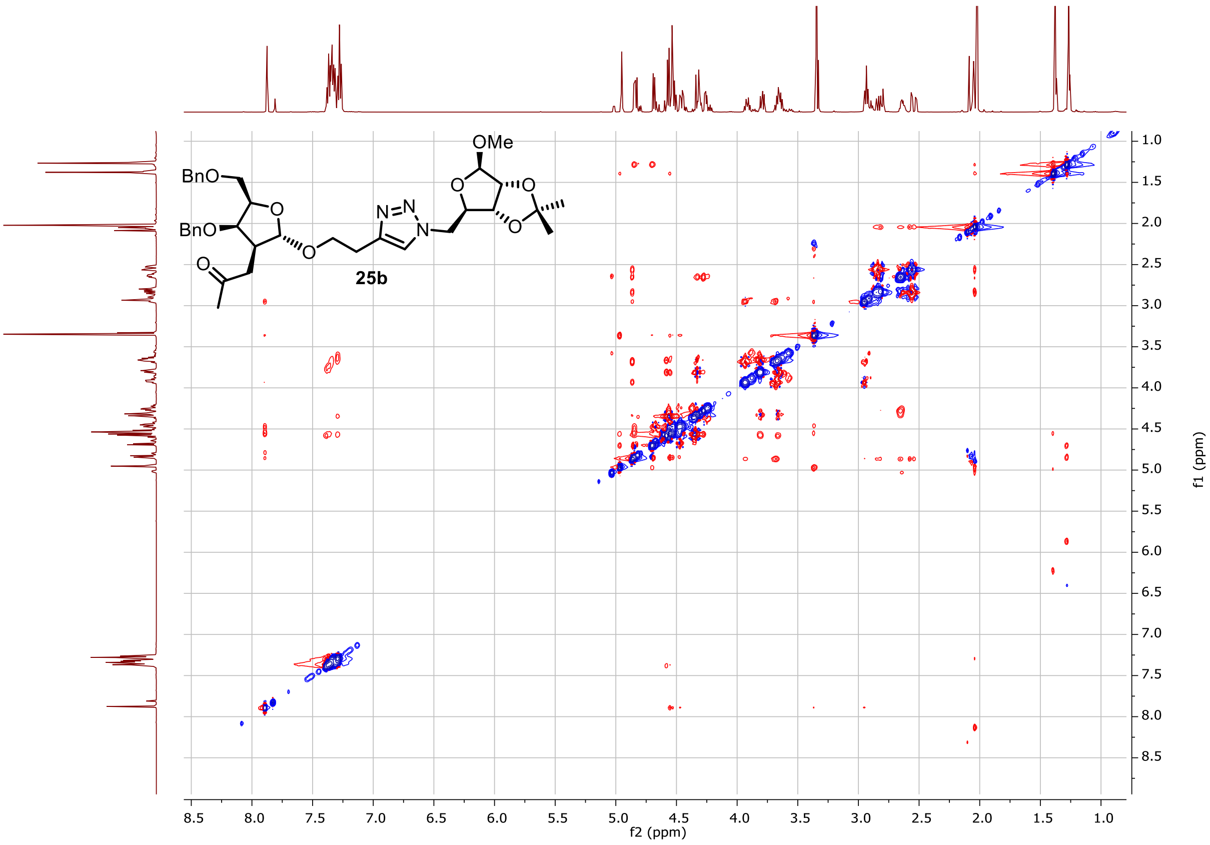

Supplementary Figure 375. NOESY spectra for **25b**

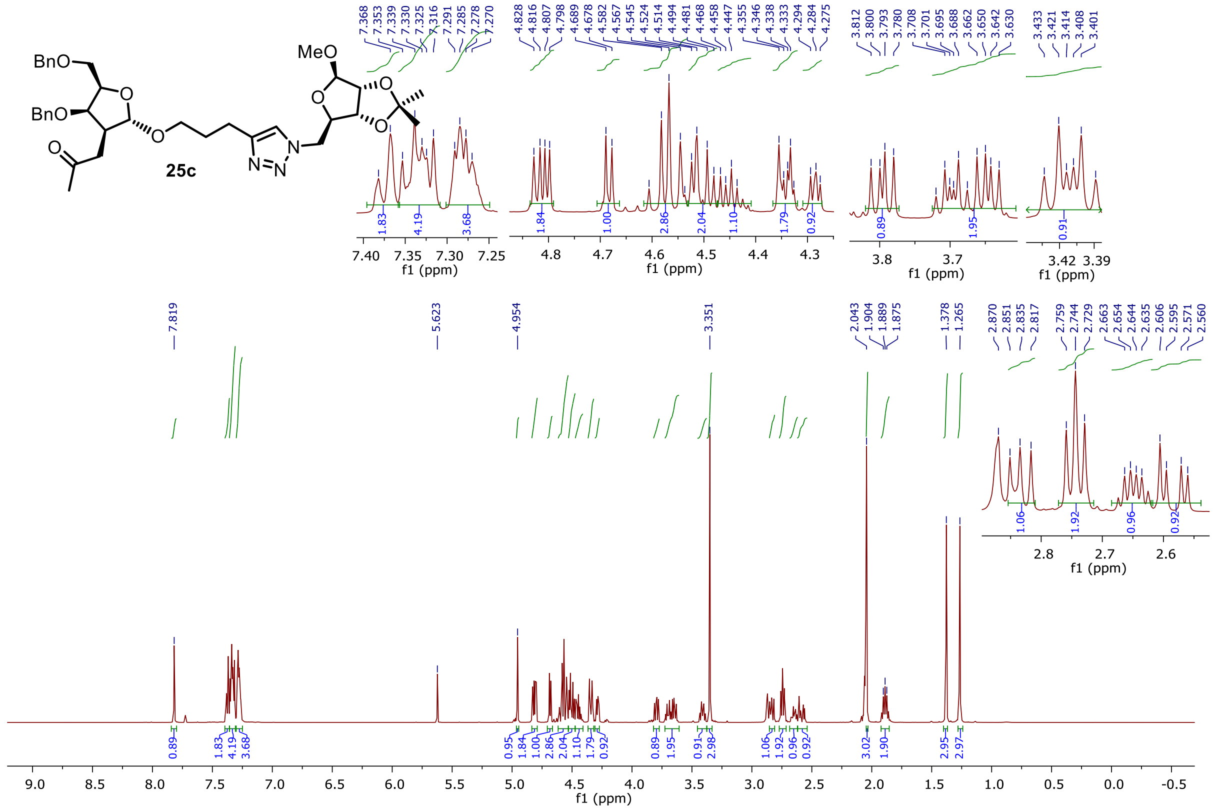

Supplementary Figure 376. ^{1}H spectra for **25c**

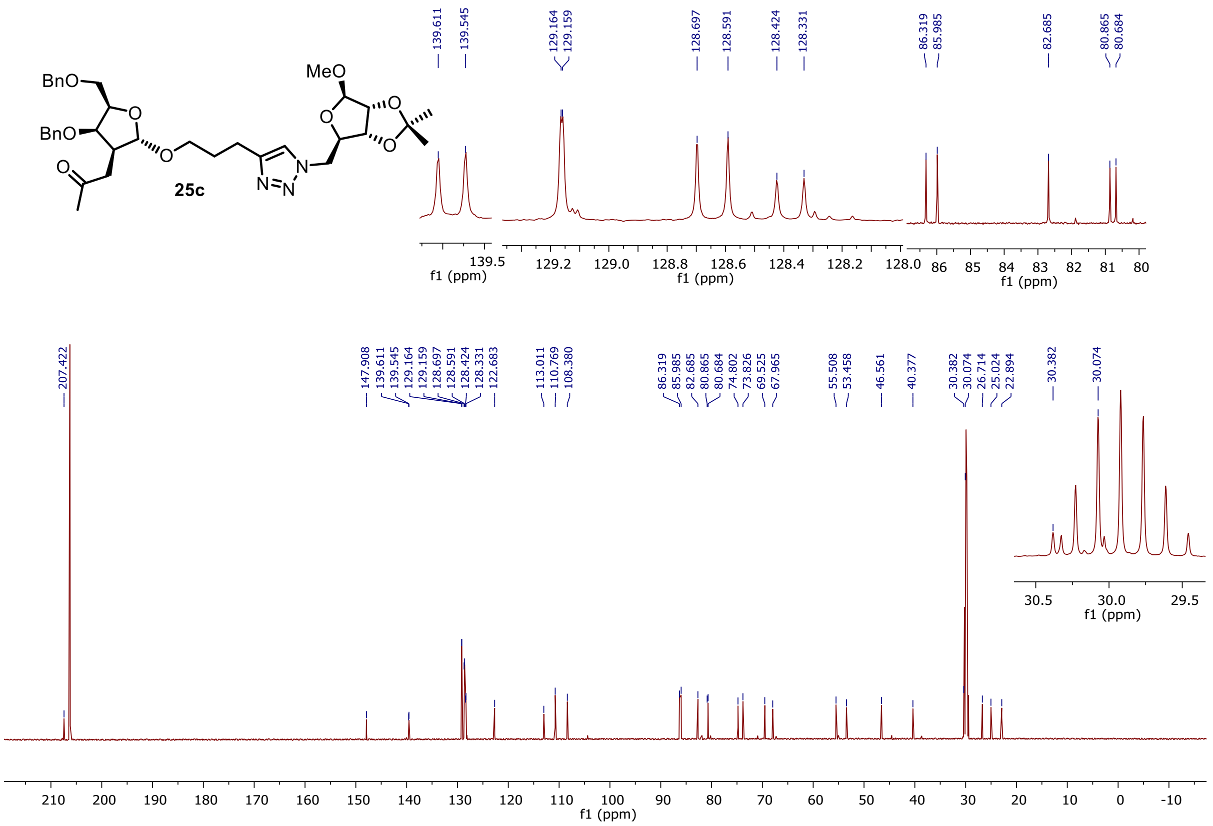

Supplementary Figure 377. ^{13}C spectra for **25c**

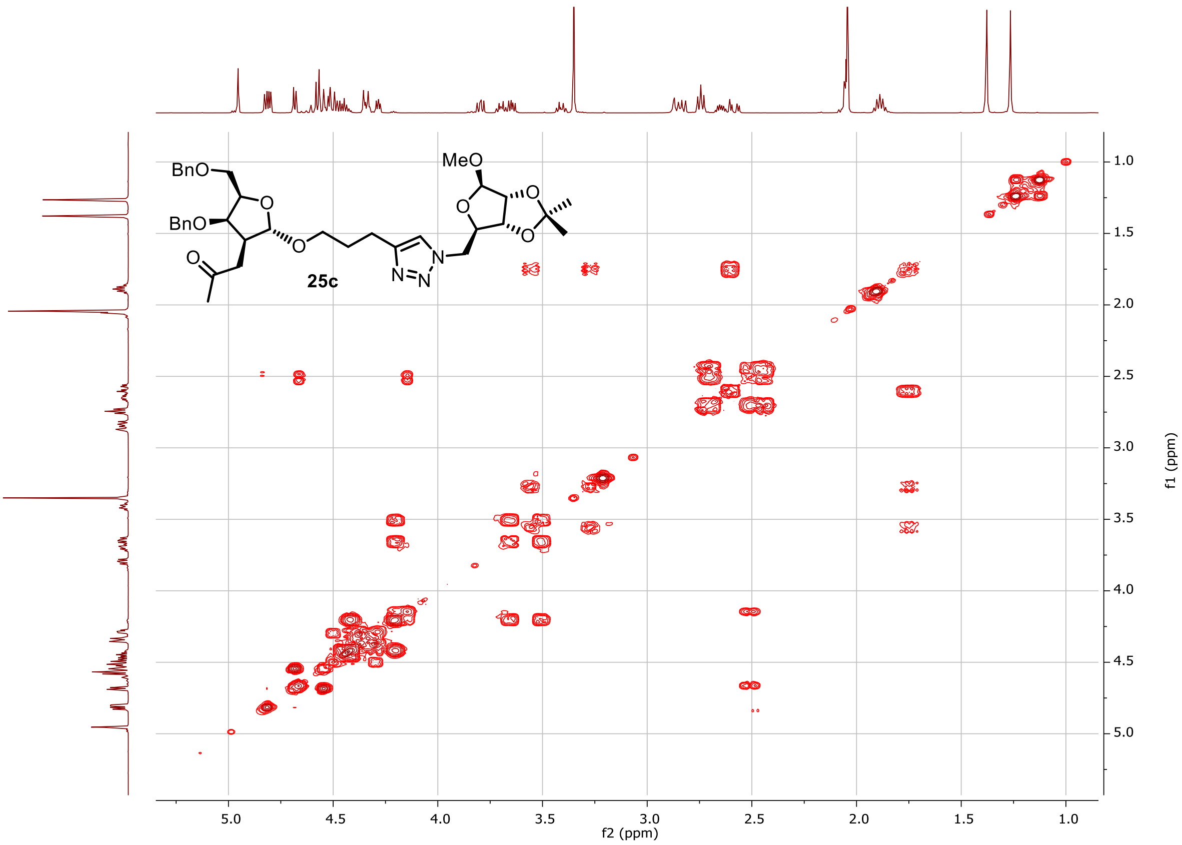

Supplementary Figure 378. COSY spectra for **25c**

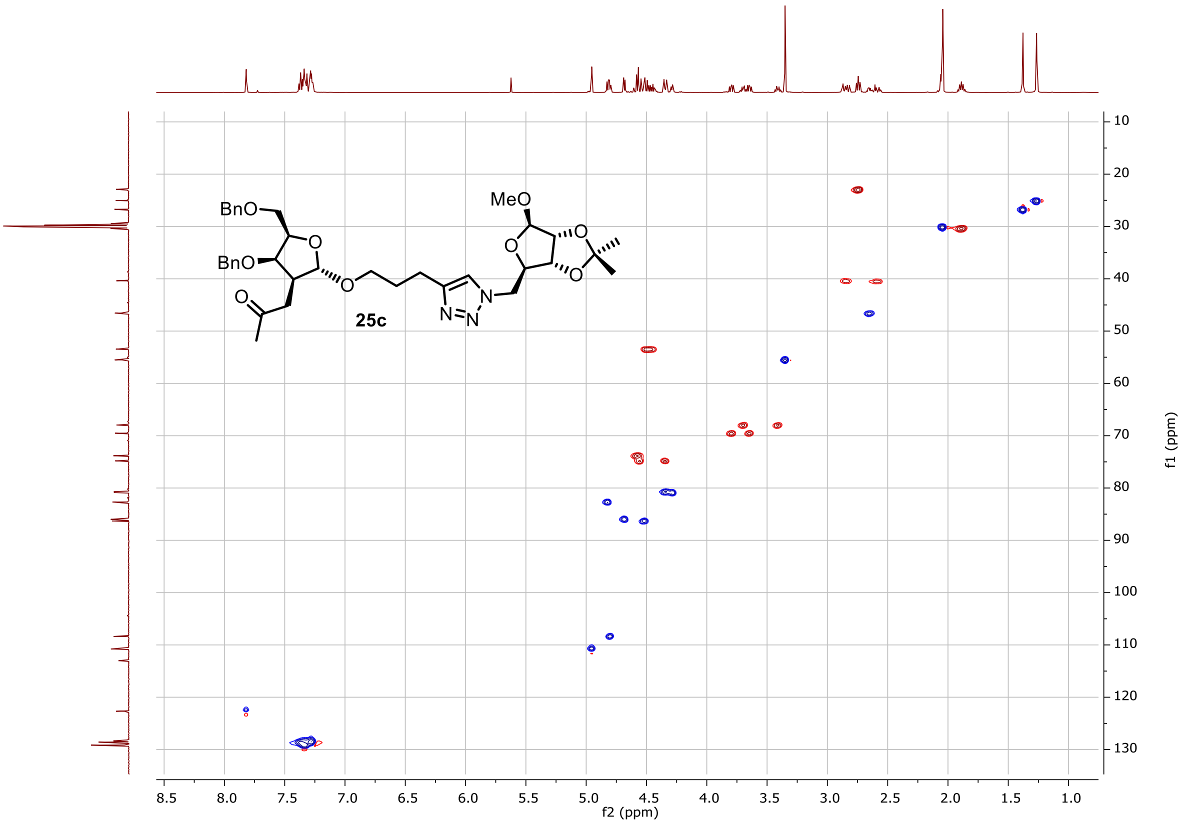

Supplementary Figure 379. HSQC spectra for **25c**

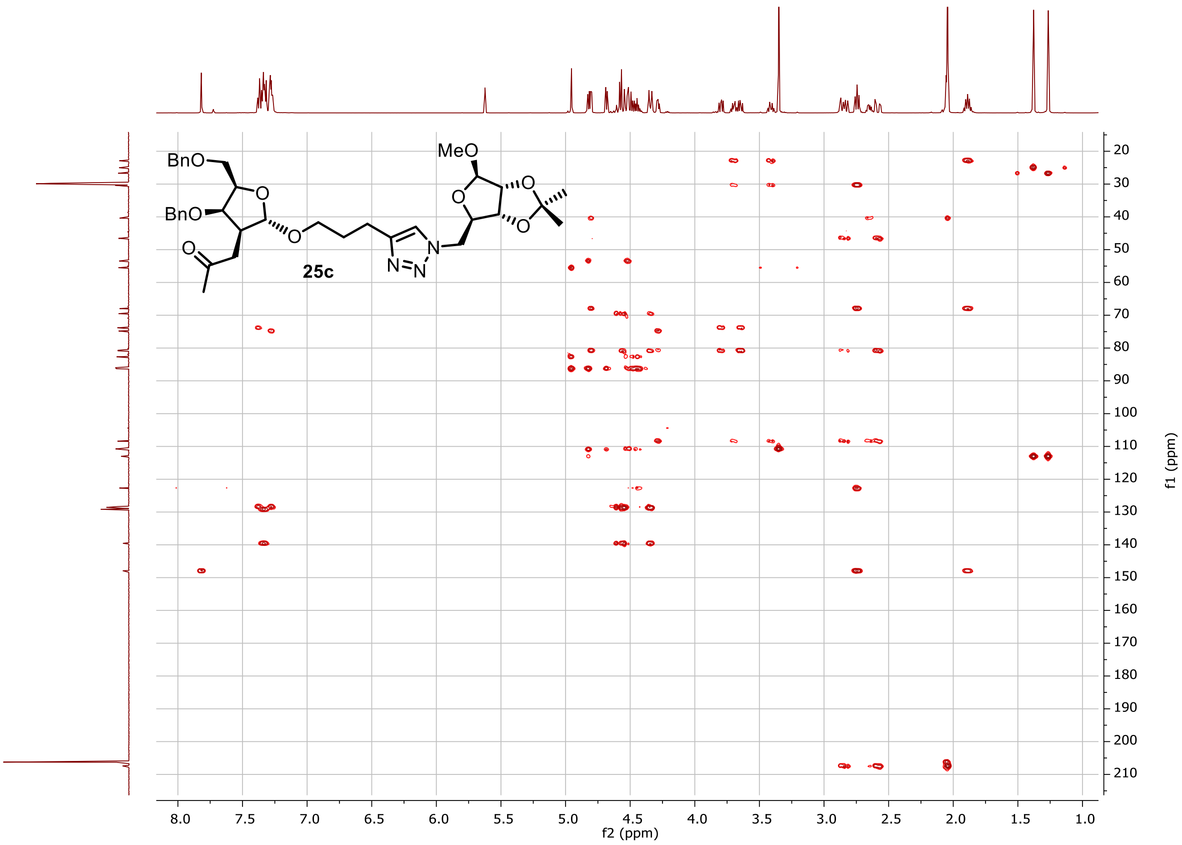

Supplementary Figure 380. HMBC spectra for **25c**

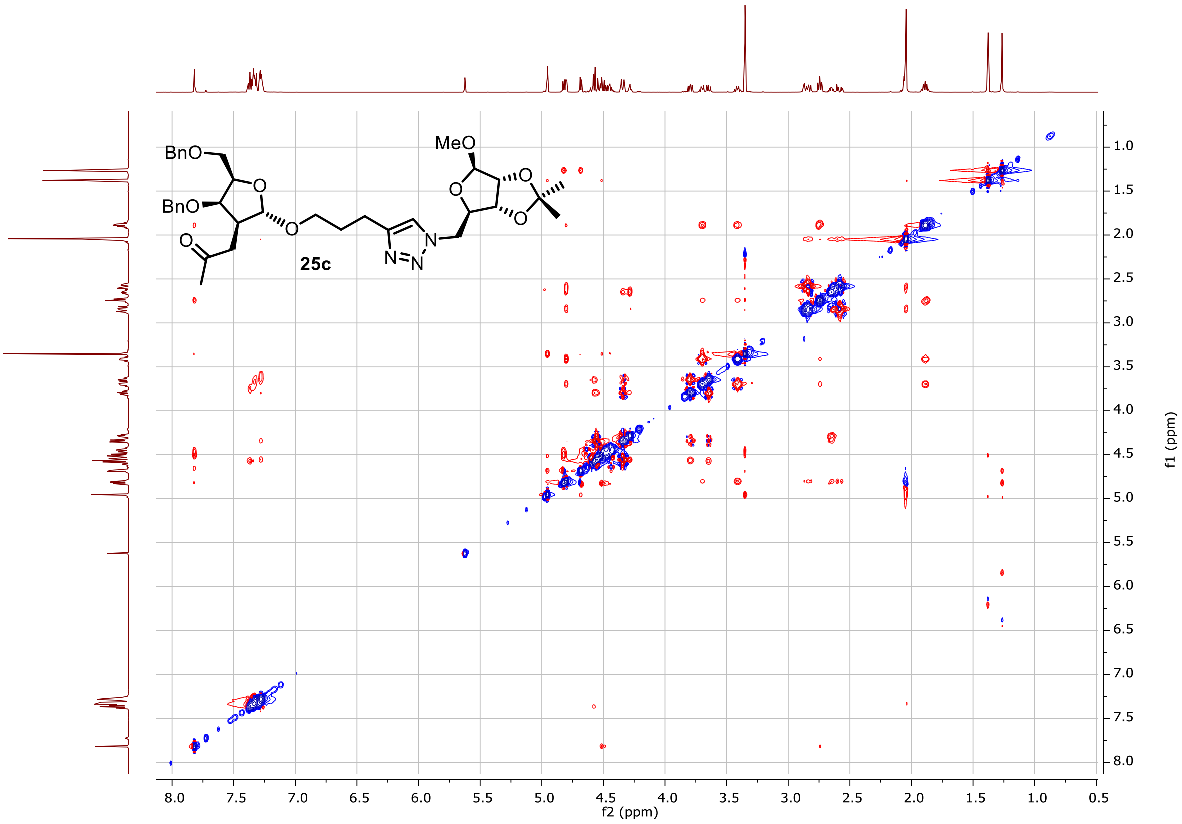

Supplementary Figure 381. NOESY spectra for **25c**

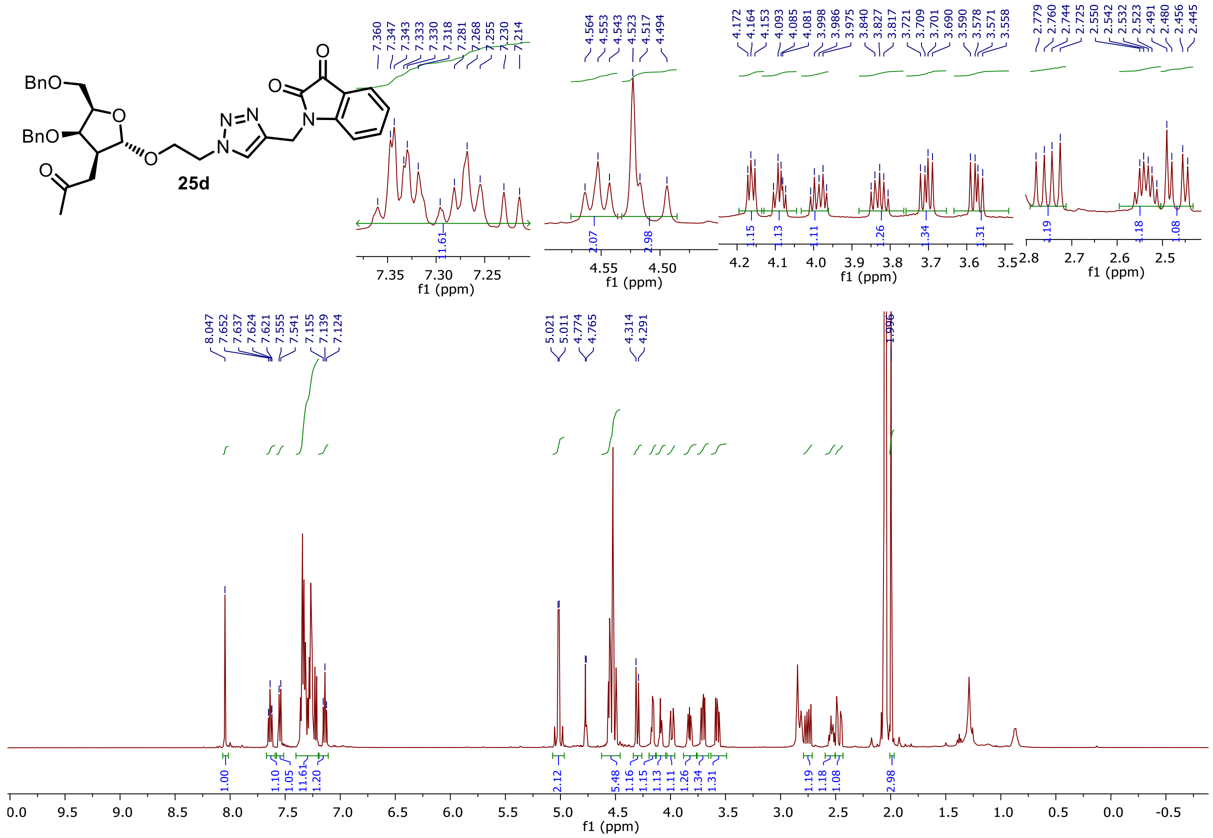

Supplementary Figure 382. ^{1}H spectra for **25d**

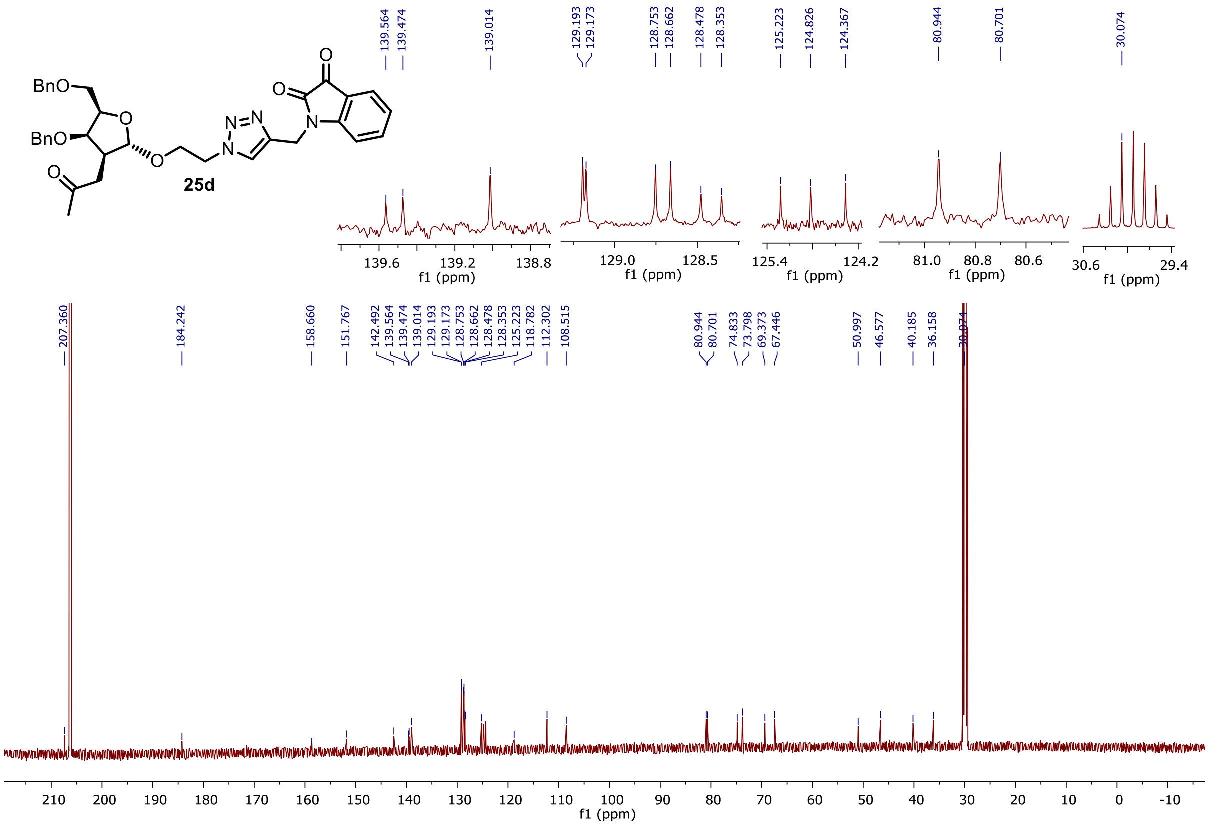

Supplementary Figure 383. ^{13}C spectra for **25d**

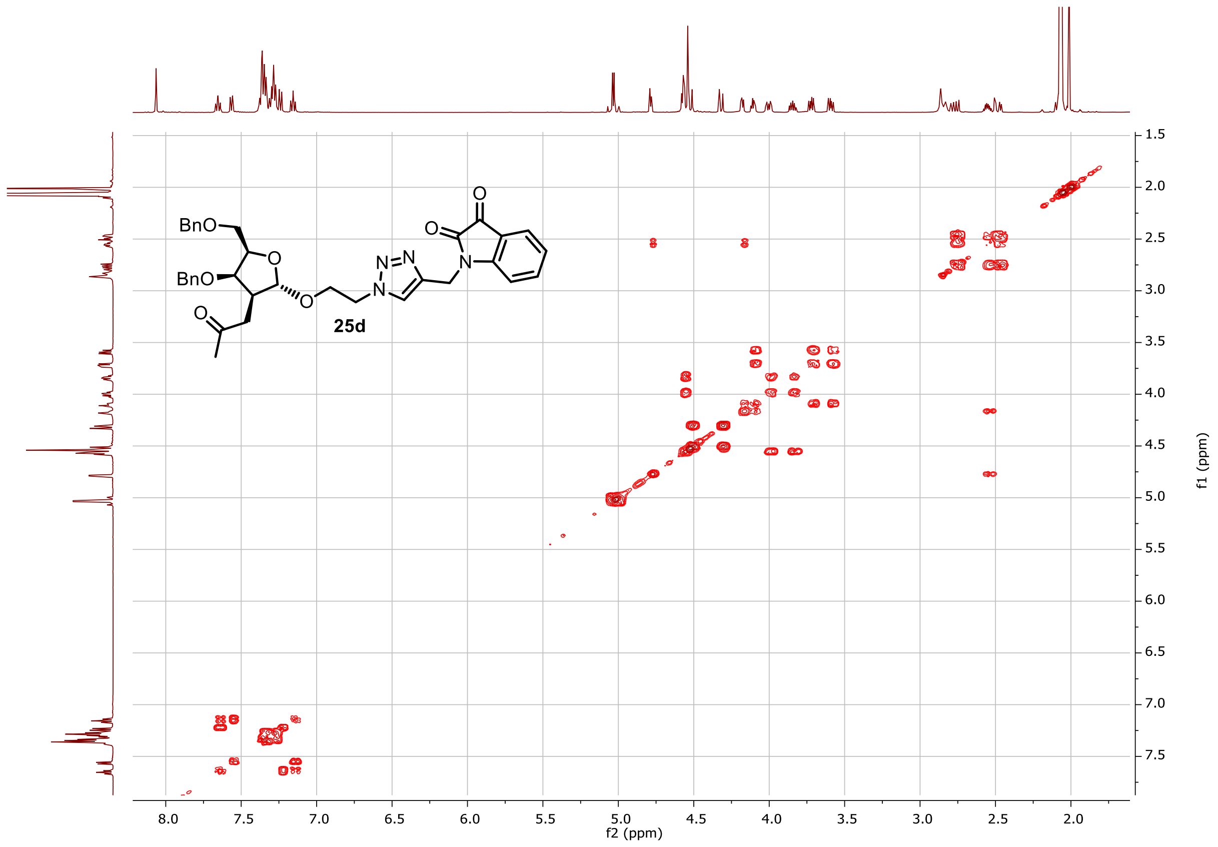

Supplementary Figure 384. COSY spectra for **25d**

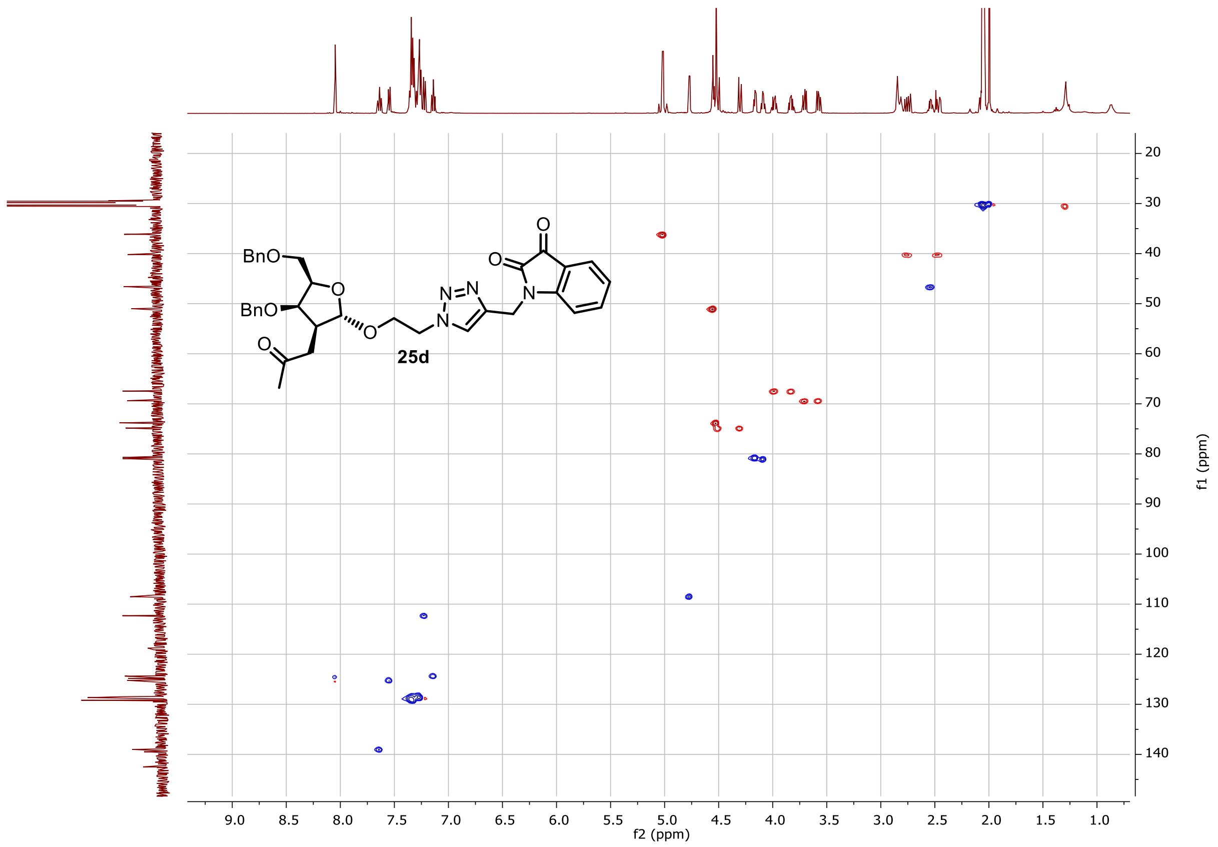

Supplementary Figure 385. HSQC spectra for **25d**

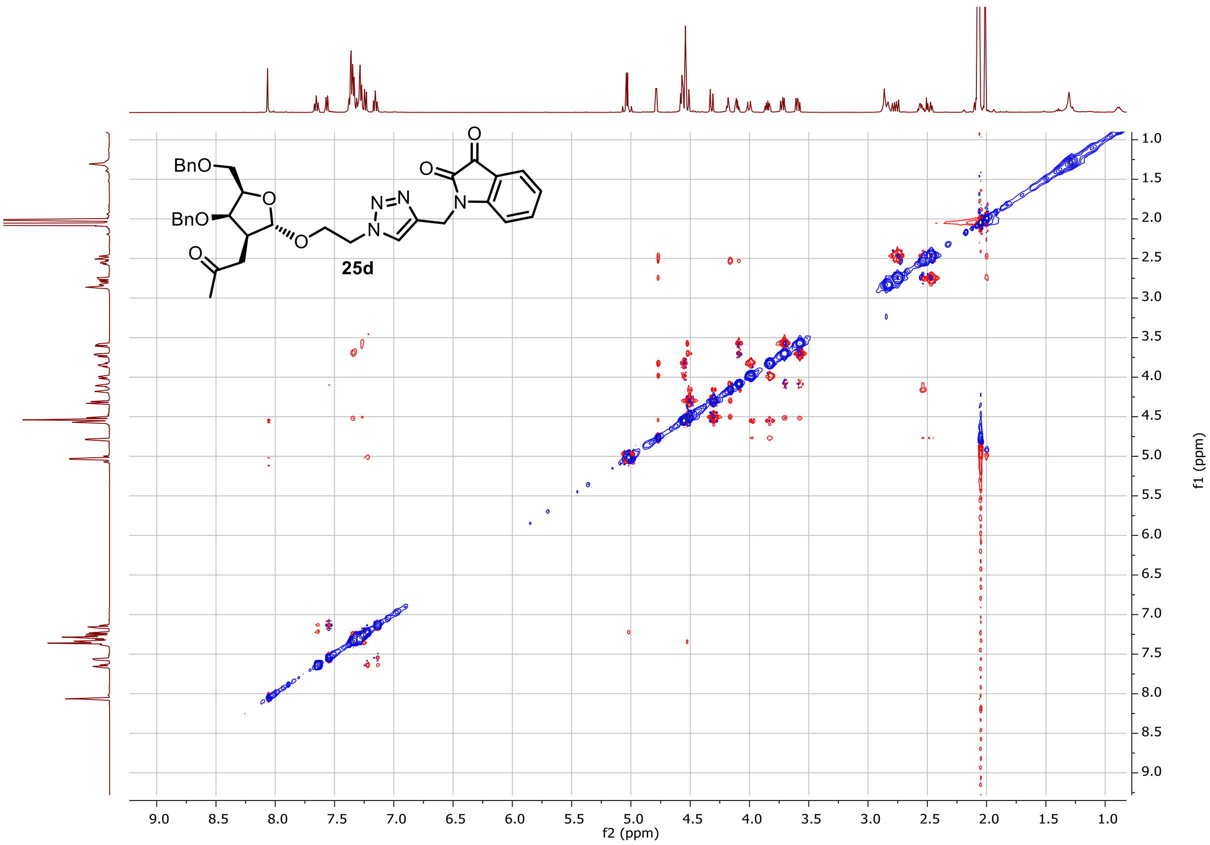

Supplementary Figure 386. NOESY spectra for **25d**

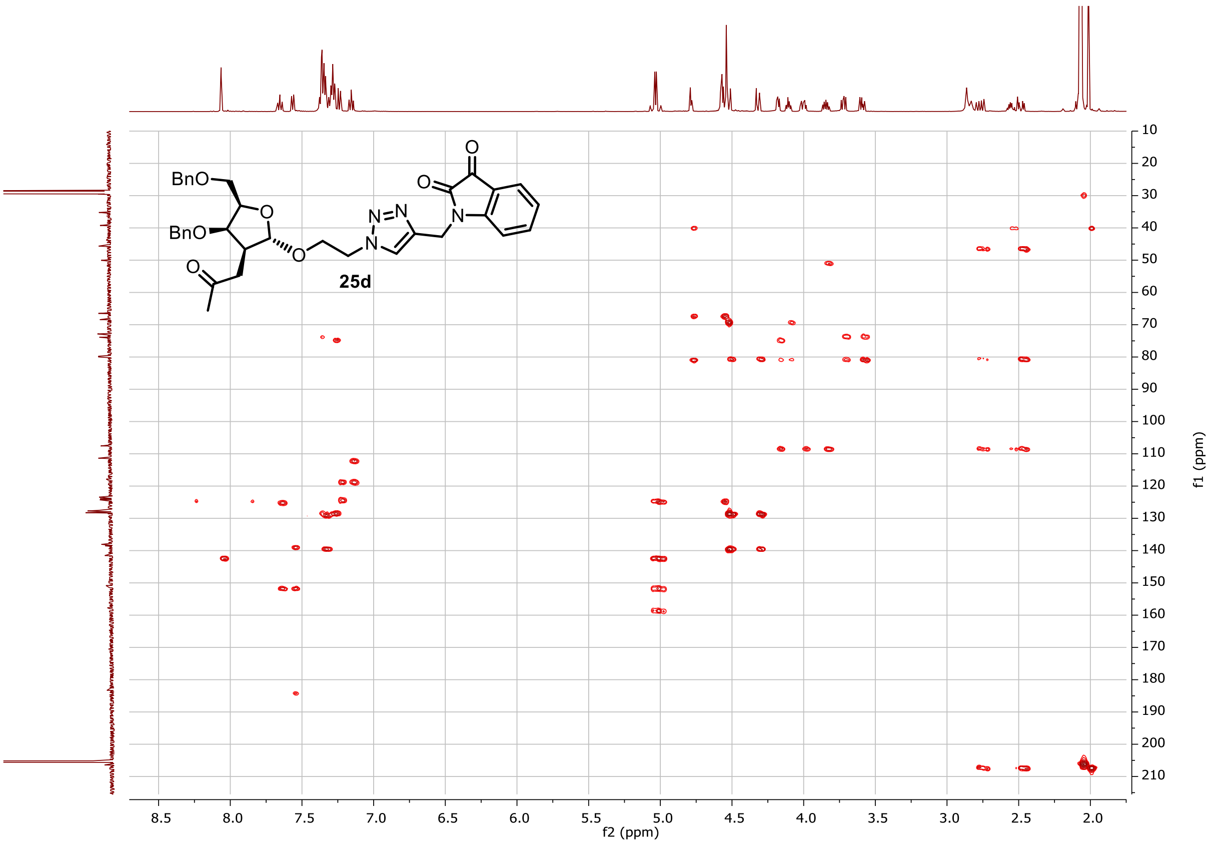

Supplementary Figure 387. HMBC spectra for **25d**

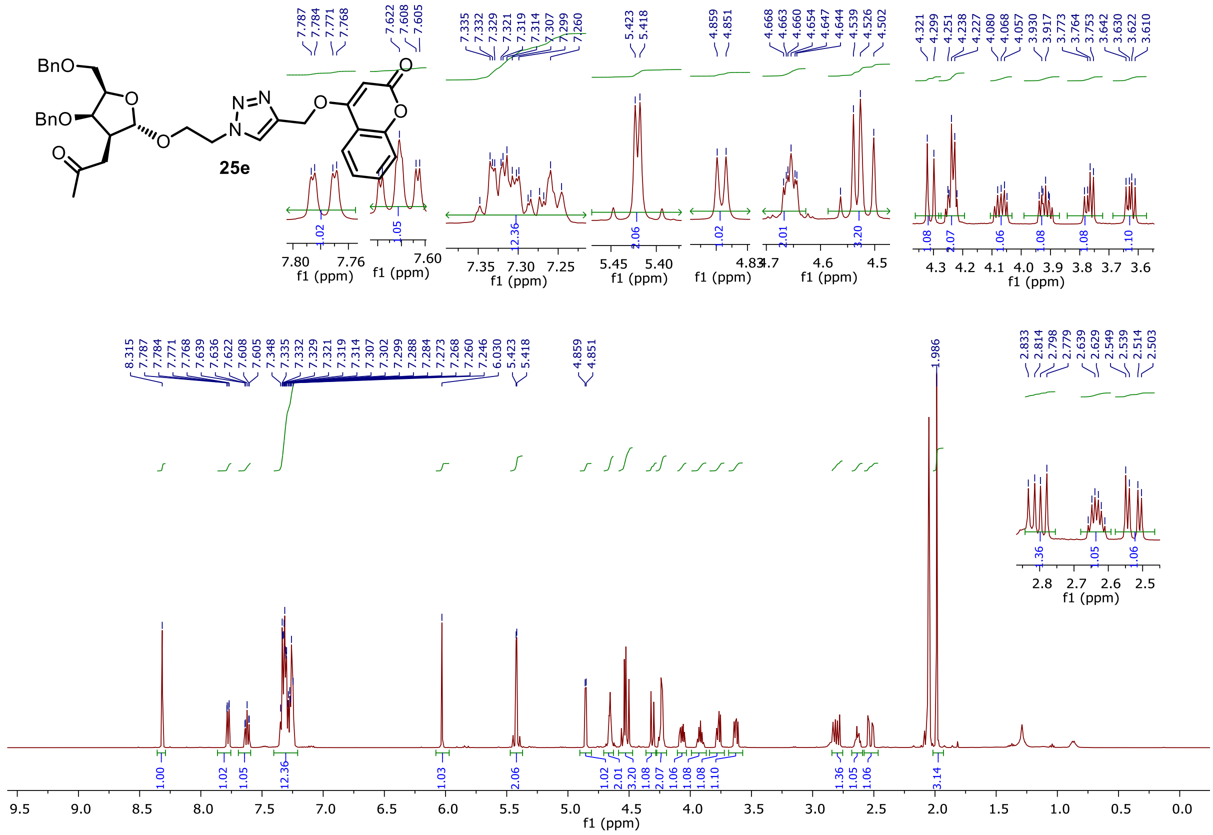

Supplementary Figure 388. ^{1}H spectra for **25e**

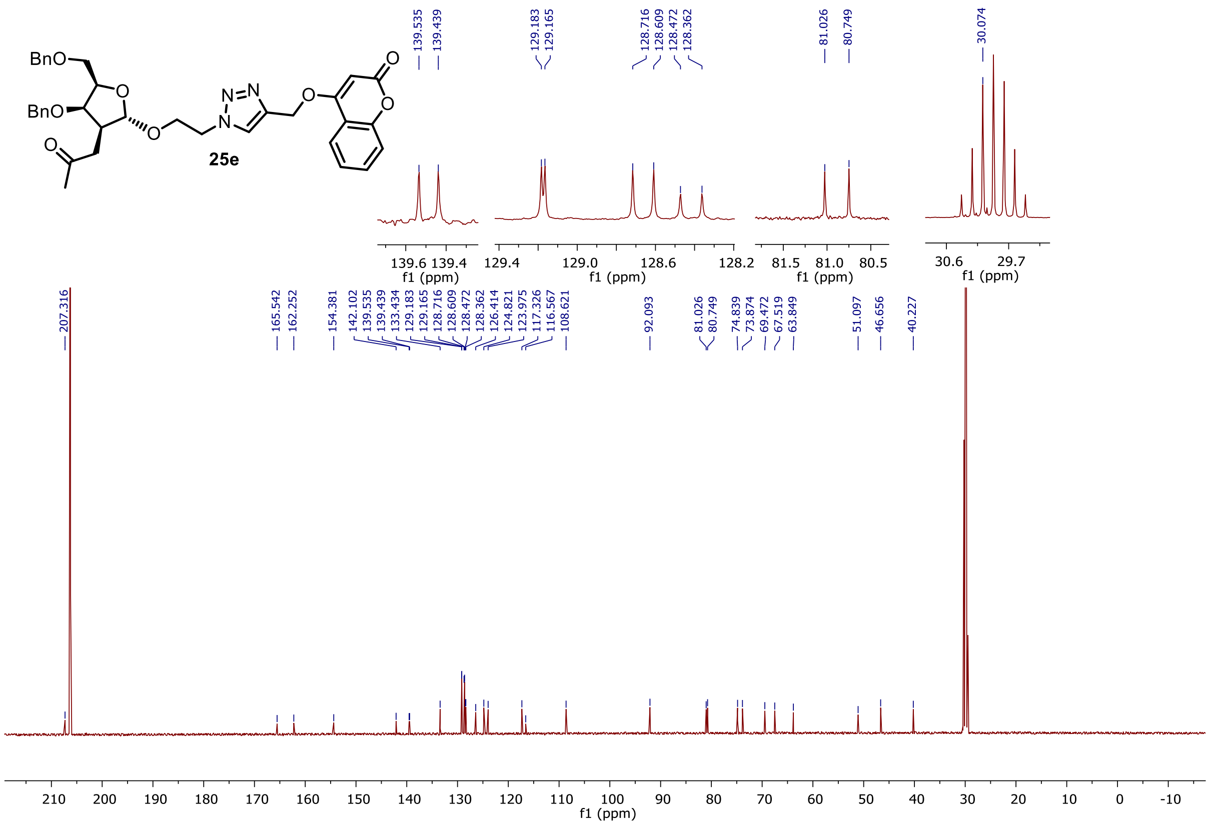

Supplementary Figure 389. ^{13}C spectra for **25e**

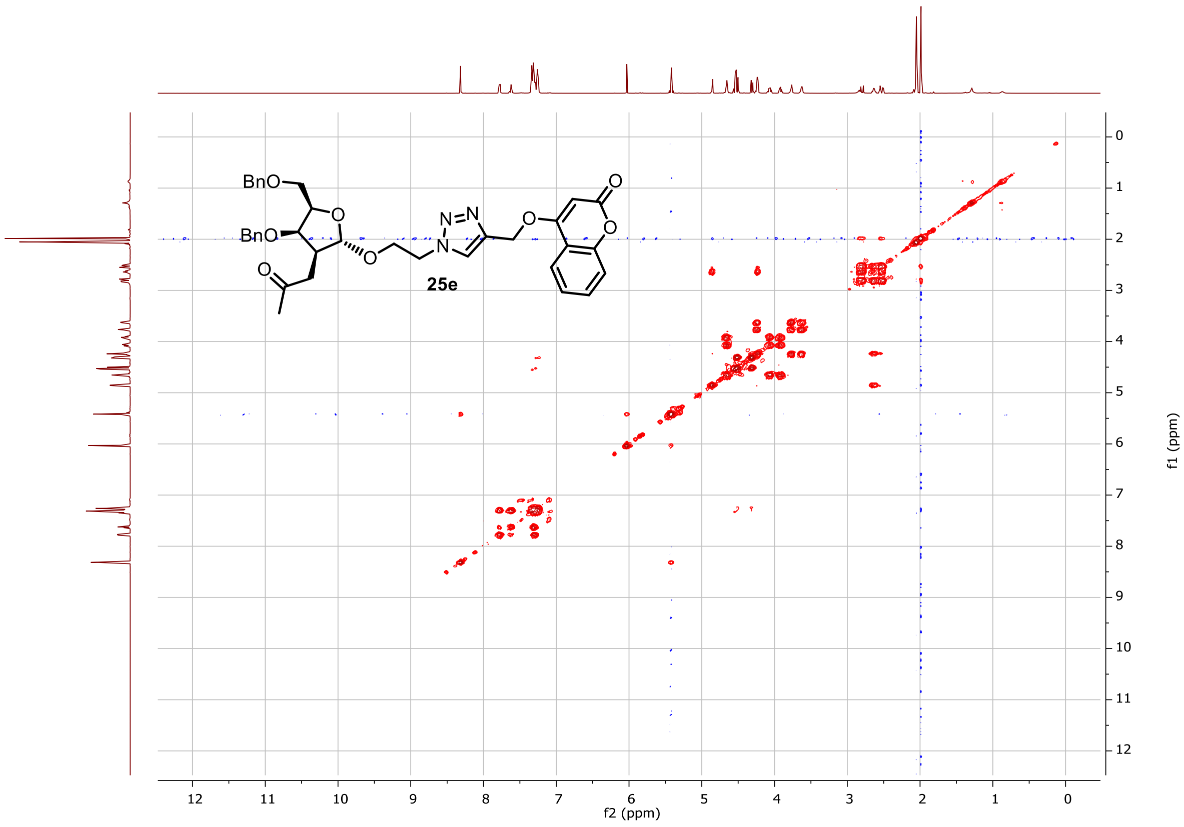

Supplementary Figure 390. COSY spectra for **25e**

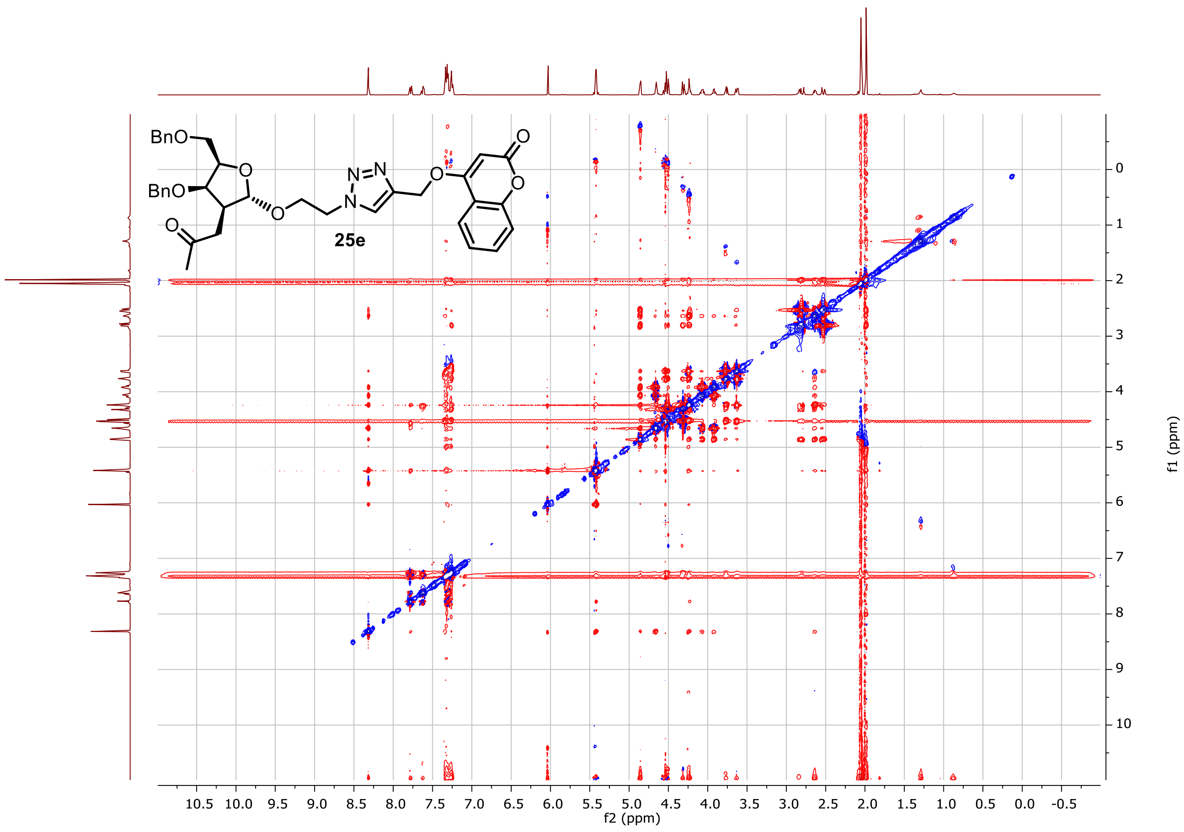

Supplementary Figure 391. NOESY spectra for **25e**

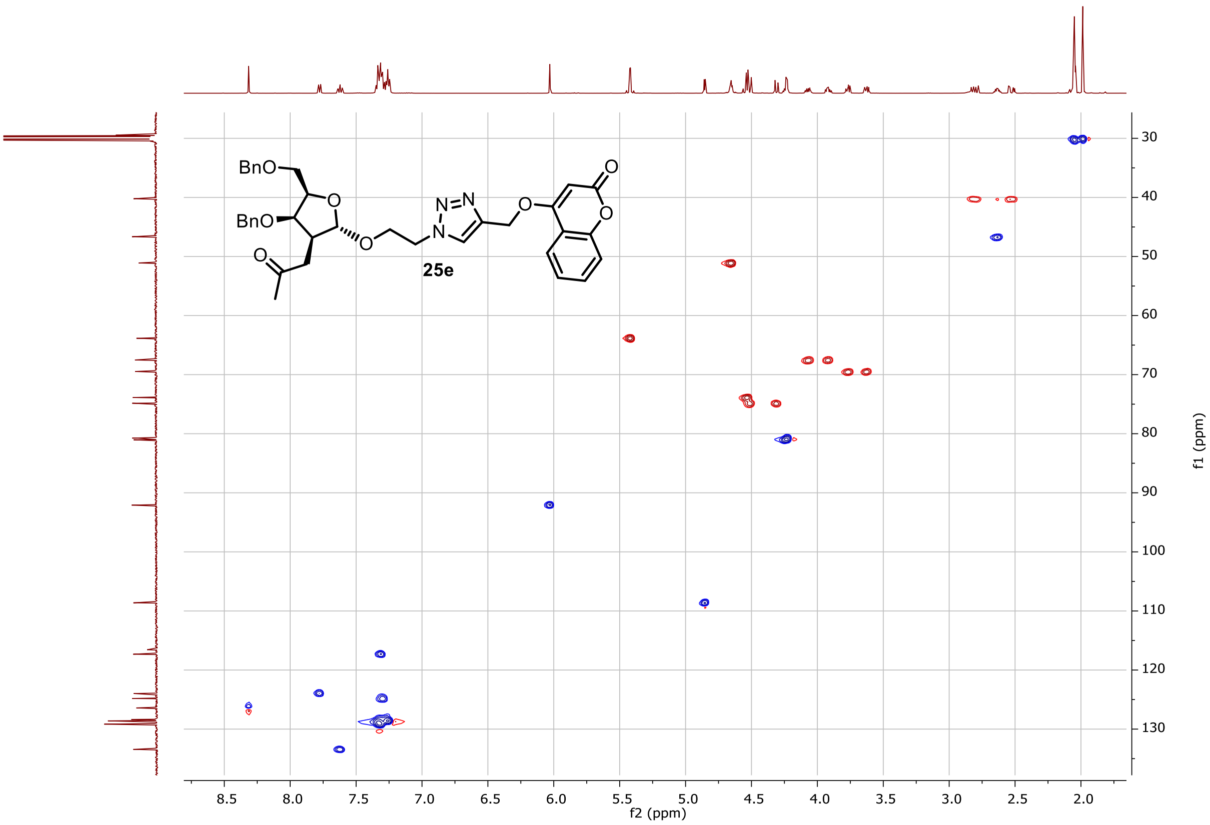

Supplementary Figure 392. HSQC spectra for **25e**

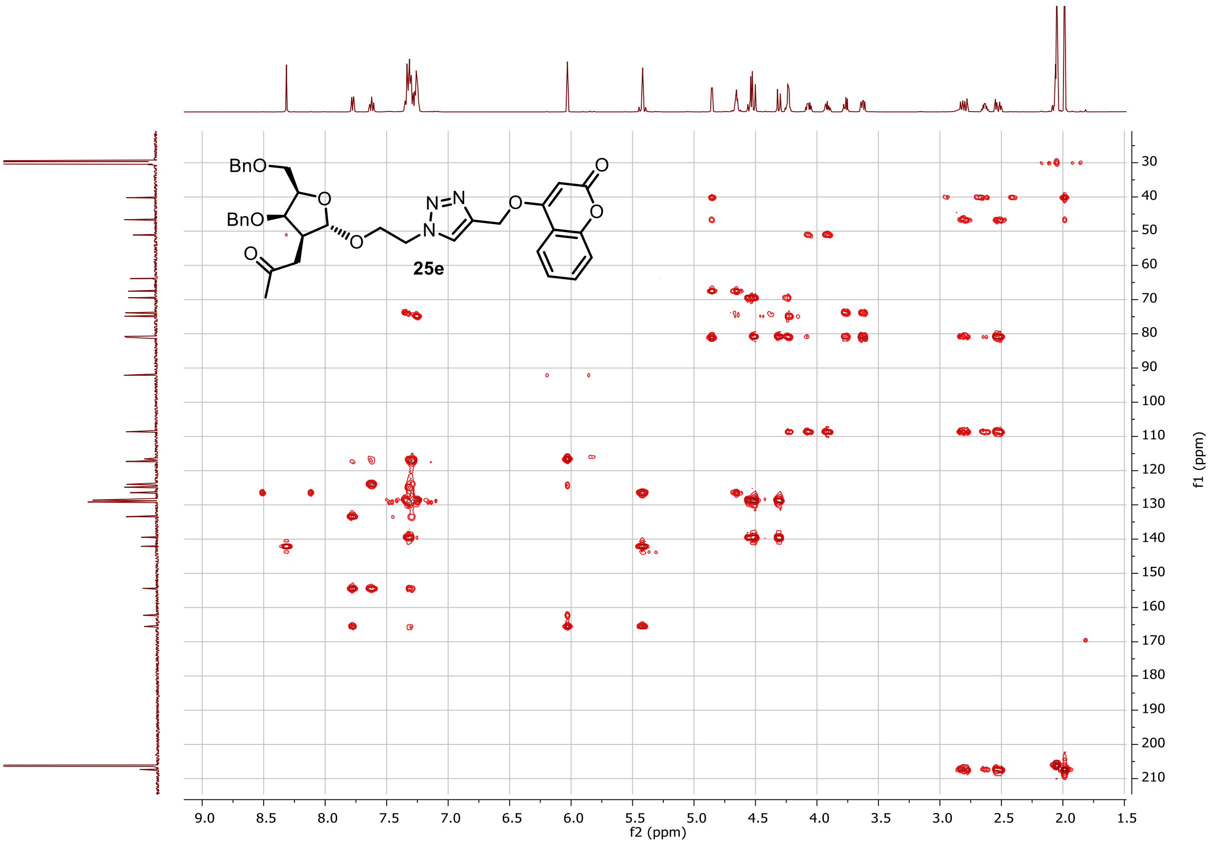

Supplementary Figure 393. HMBC spectra for **25e**

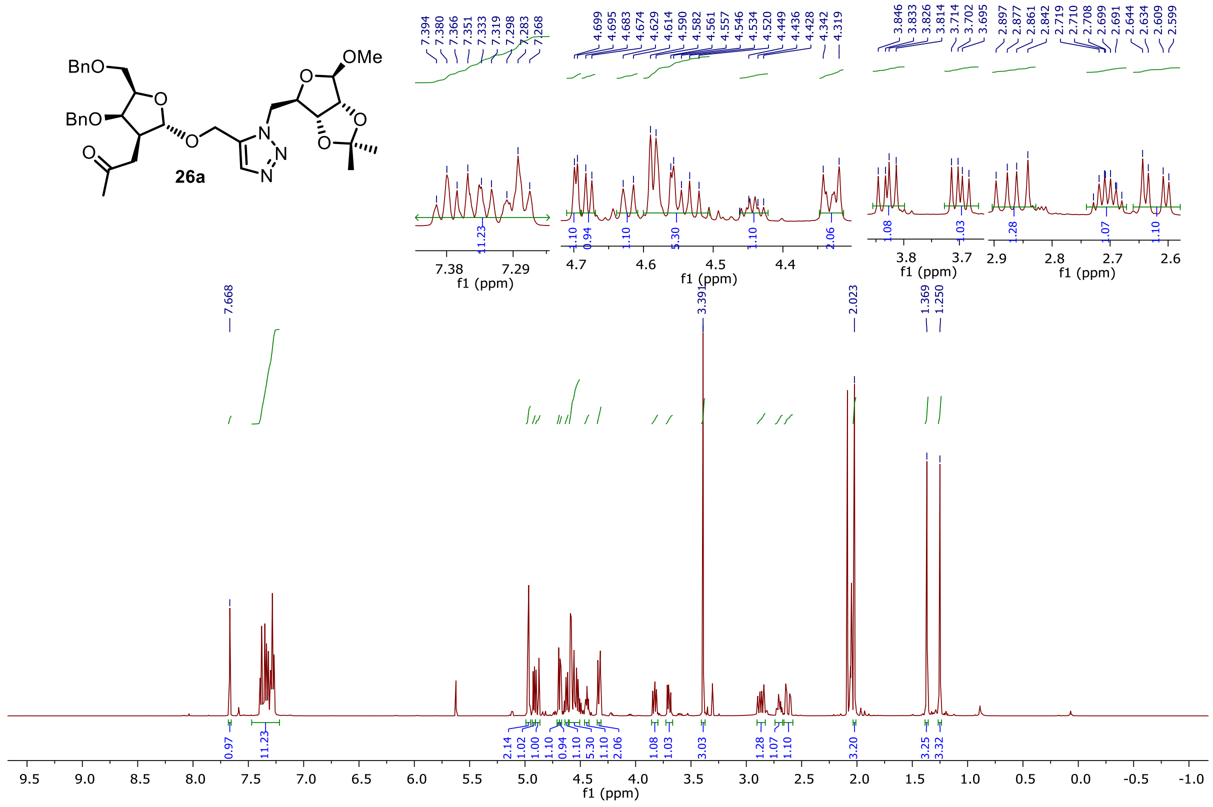

Supplementary Figure 394. ^{1}H spectra for **26a**

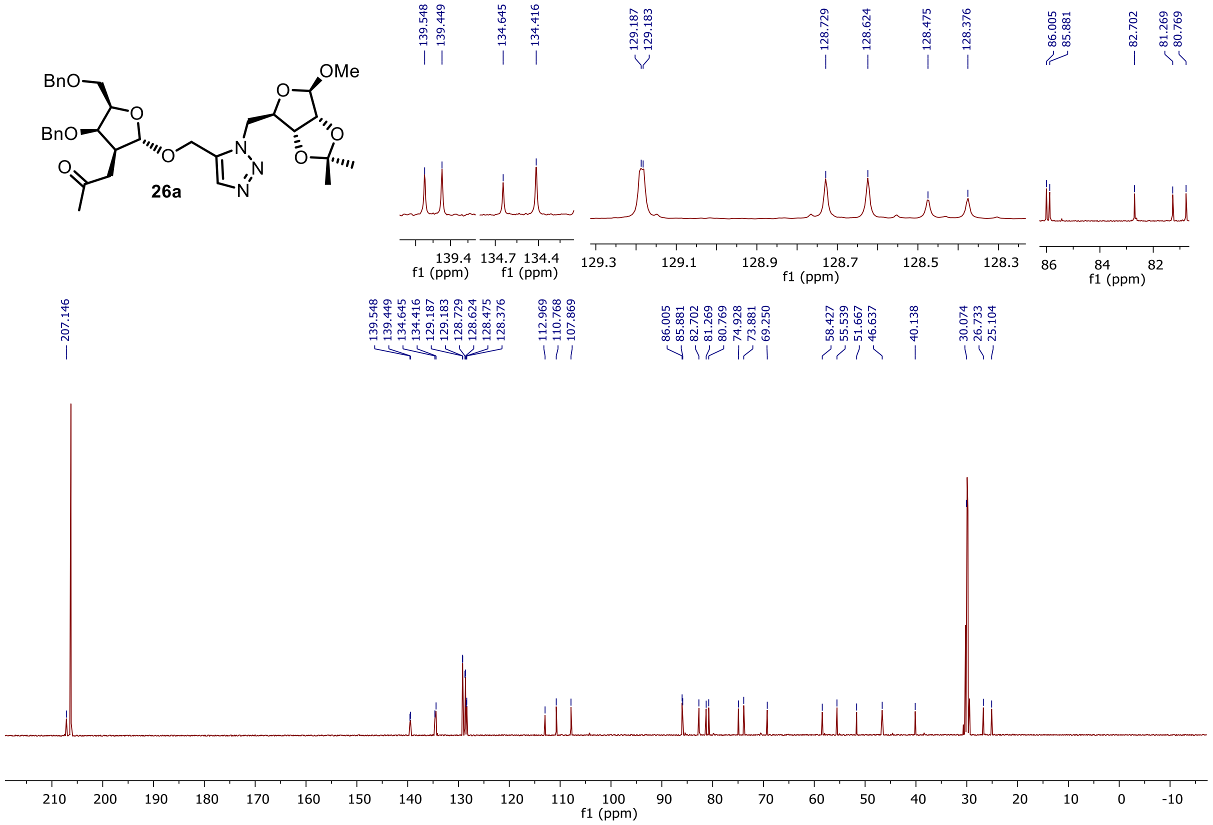

Supplementary Figure 395. ^{13}C spectra for **26a**

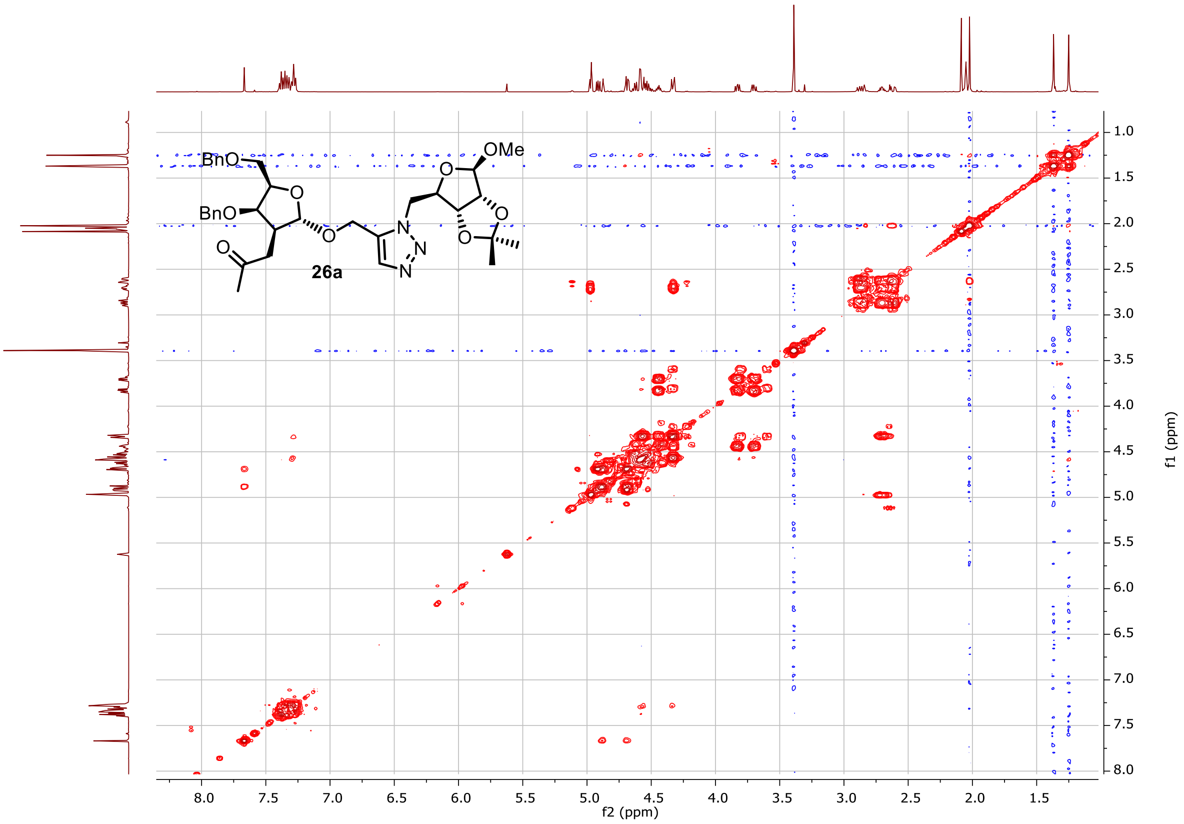

Supplementary Figure 396. COSY spectra for **26a**

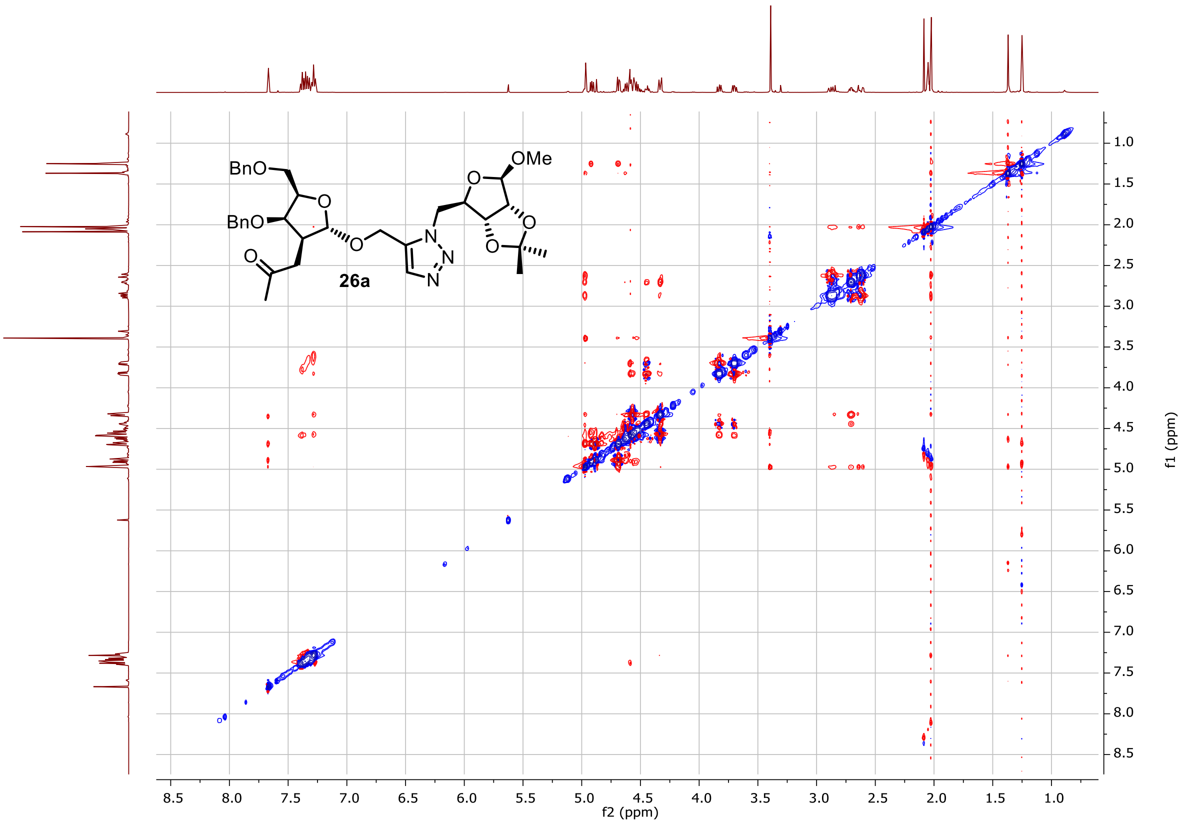

Supplementary Figure 397. NOESY spectra for **26a**

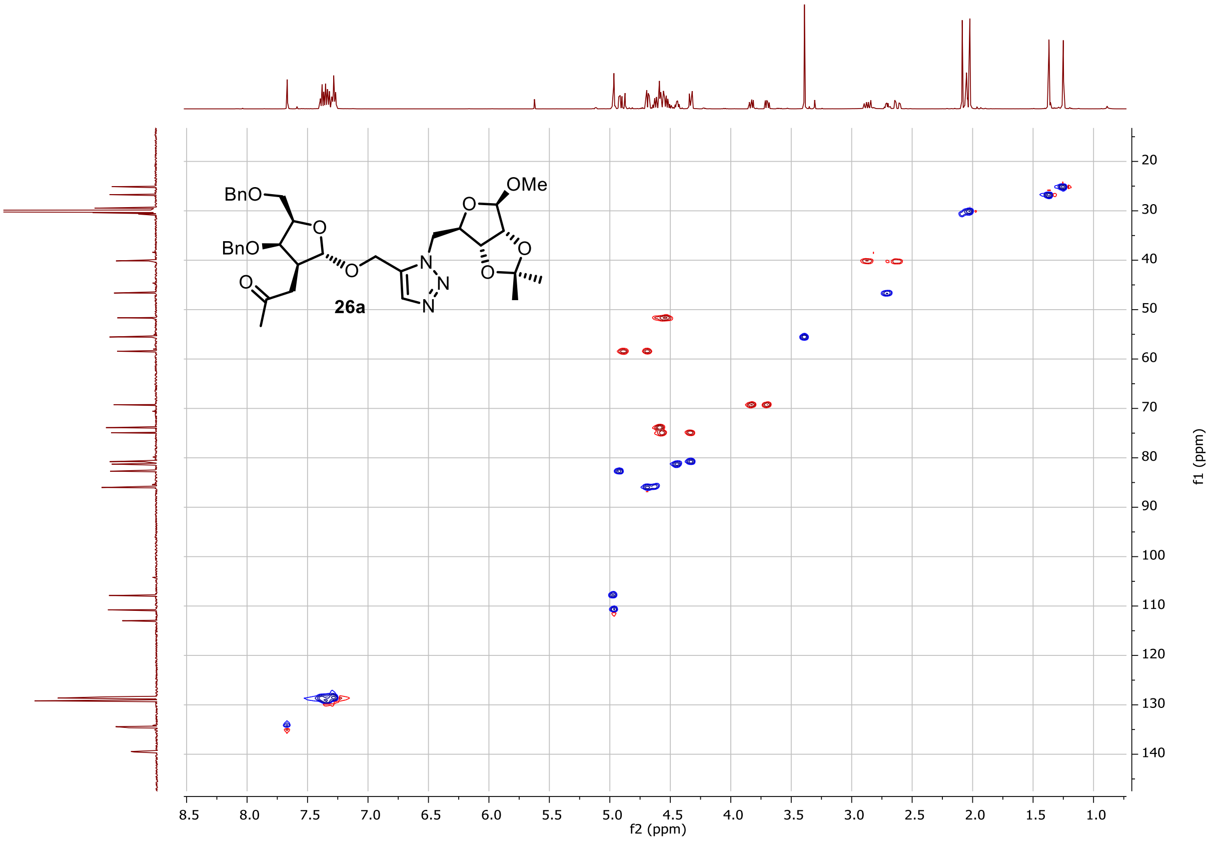

Supplementary Figure 398. HSQC spectra for **26a**

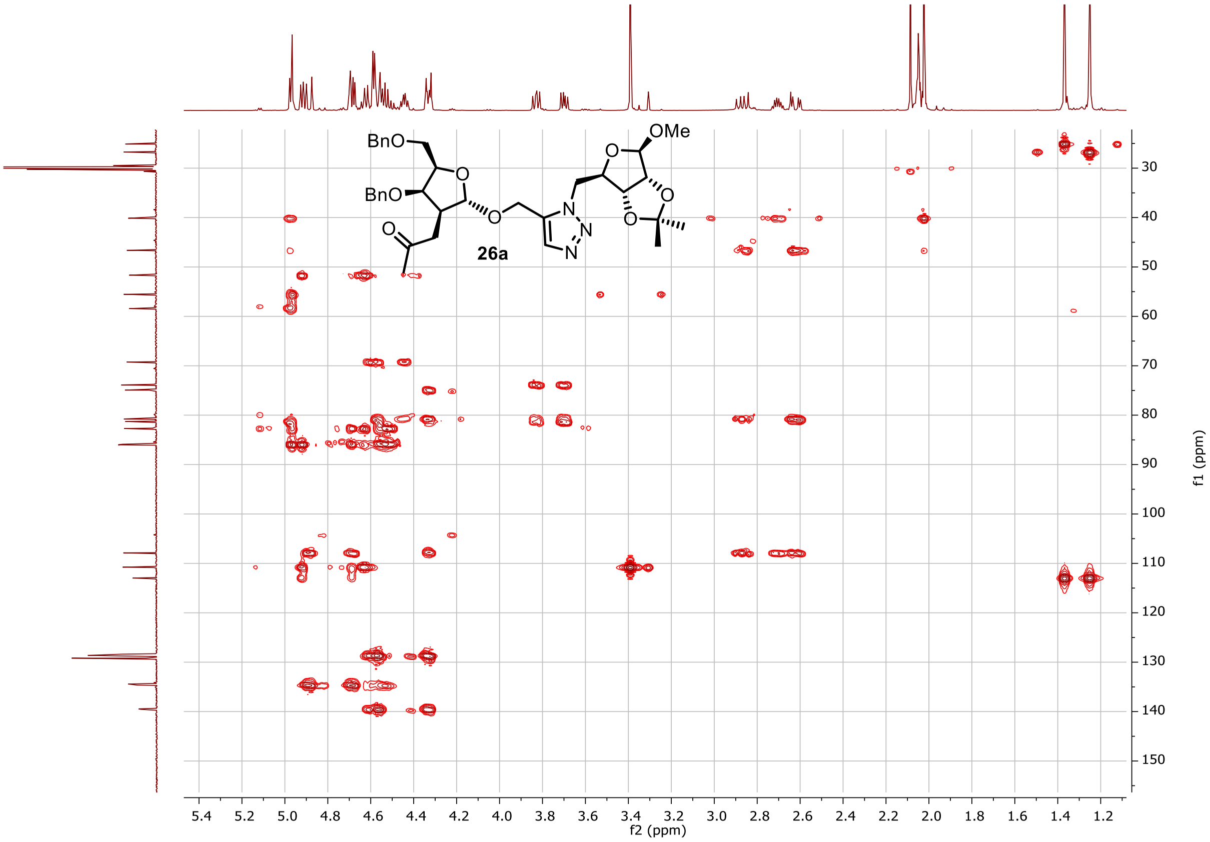

Supplementary Figure 399. HMBC spectra for **26a**

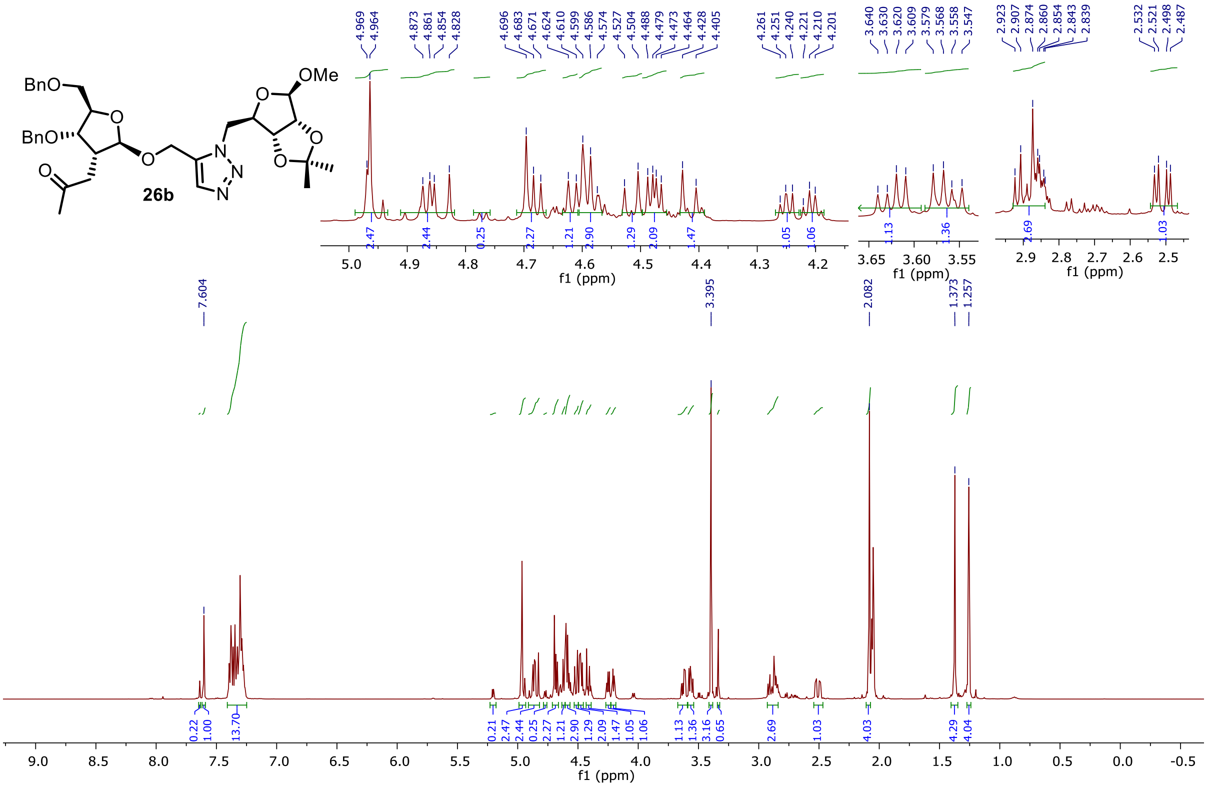

Supplementary Figure 400. ^{1}H spectra for **26b**

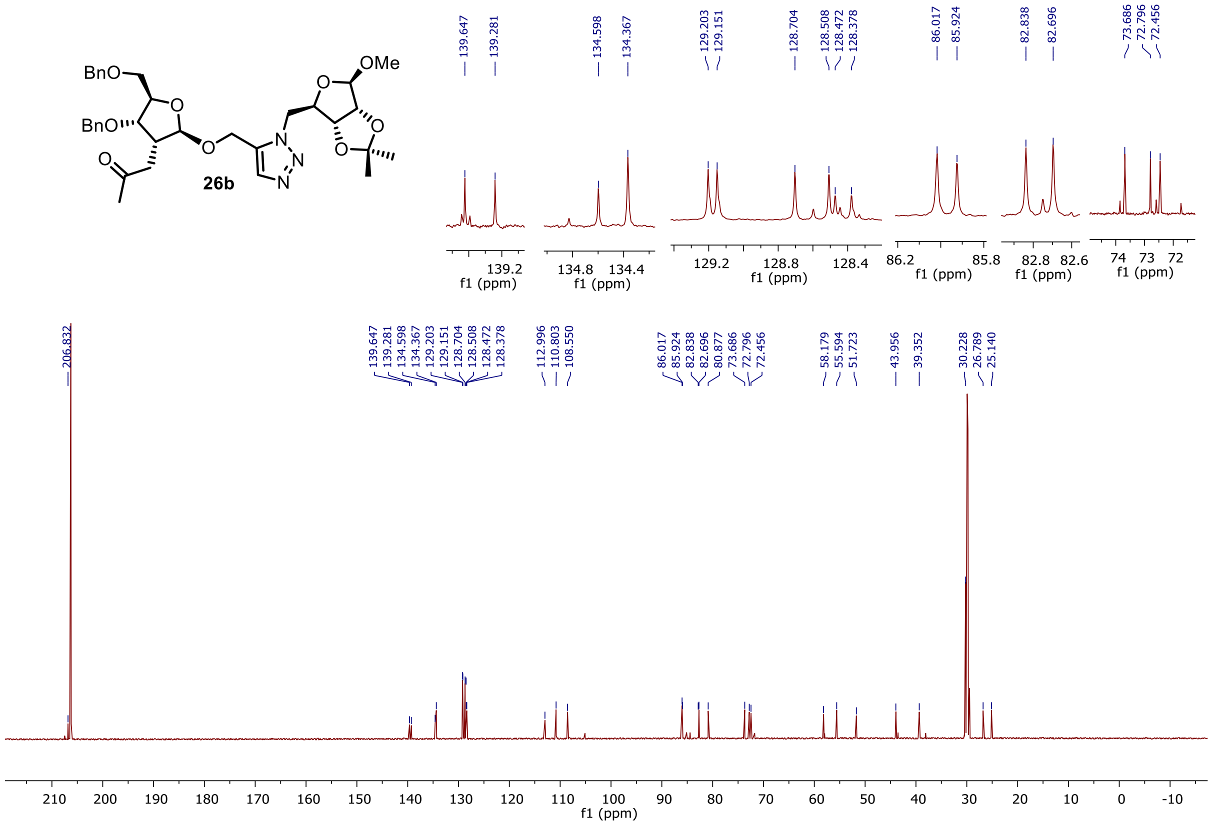

Supplementary Figure 401. ^{13}C spectra for **26b**

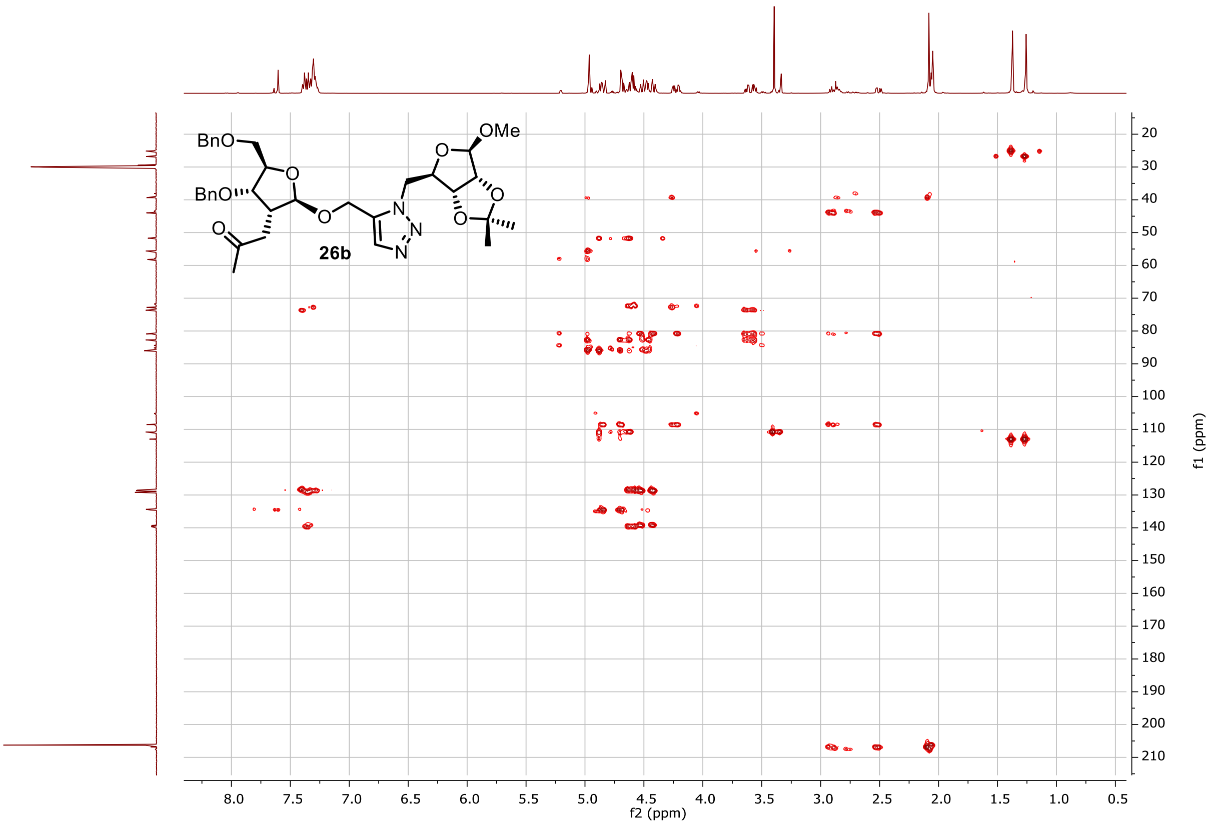

Supplementary Figure 402. HMBC spectra for **26b**

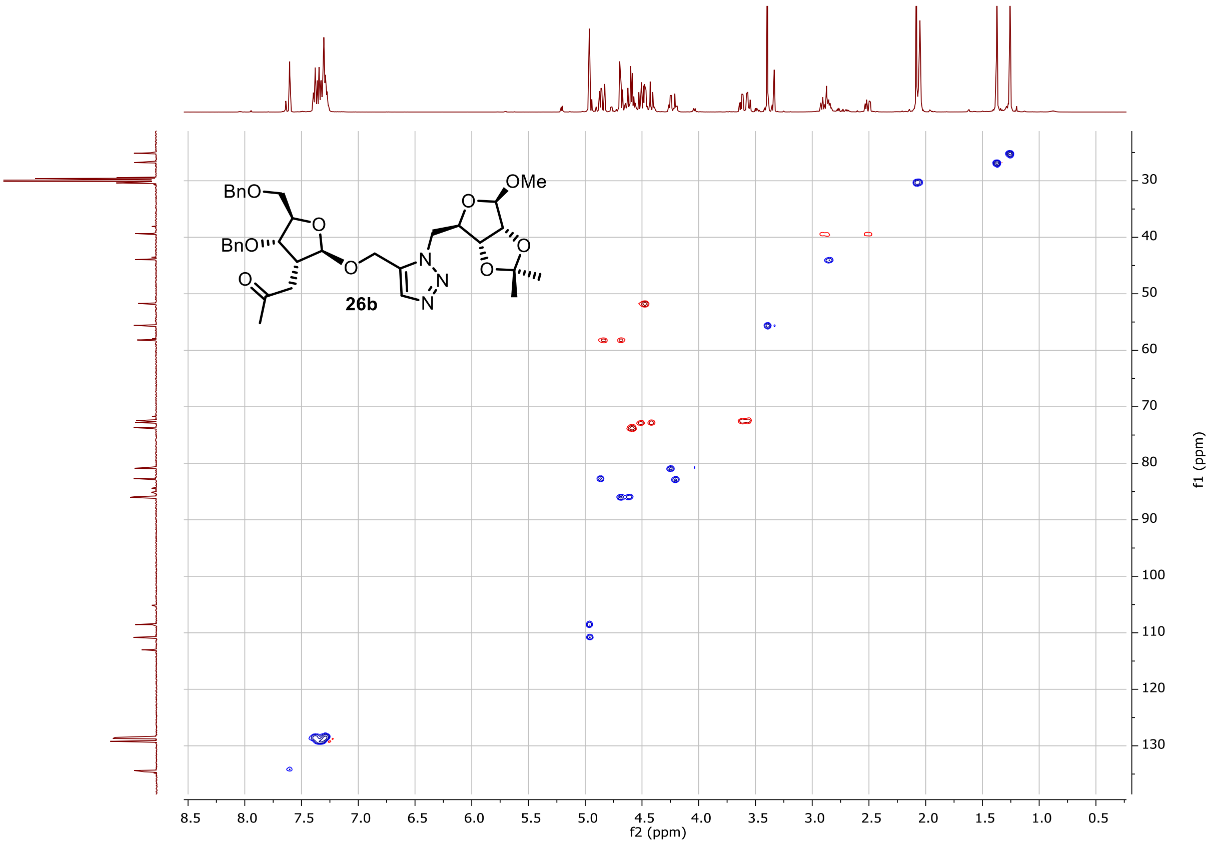

Supplementary Figure 403. HSQC spectra for **26b**

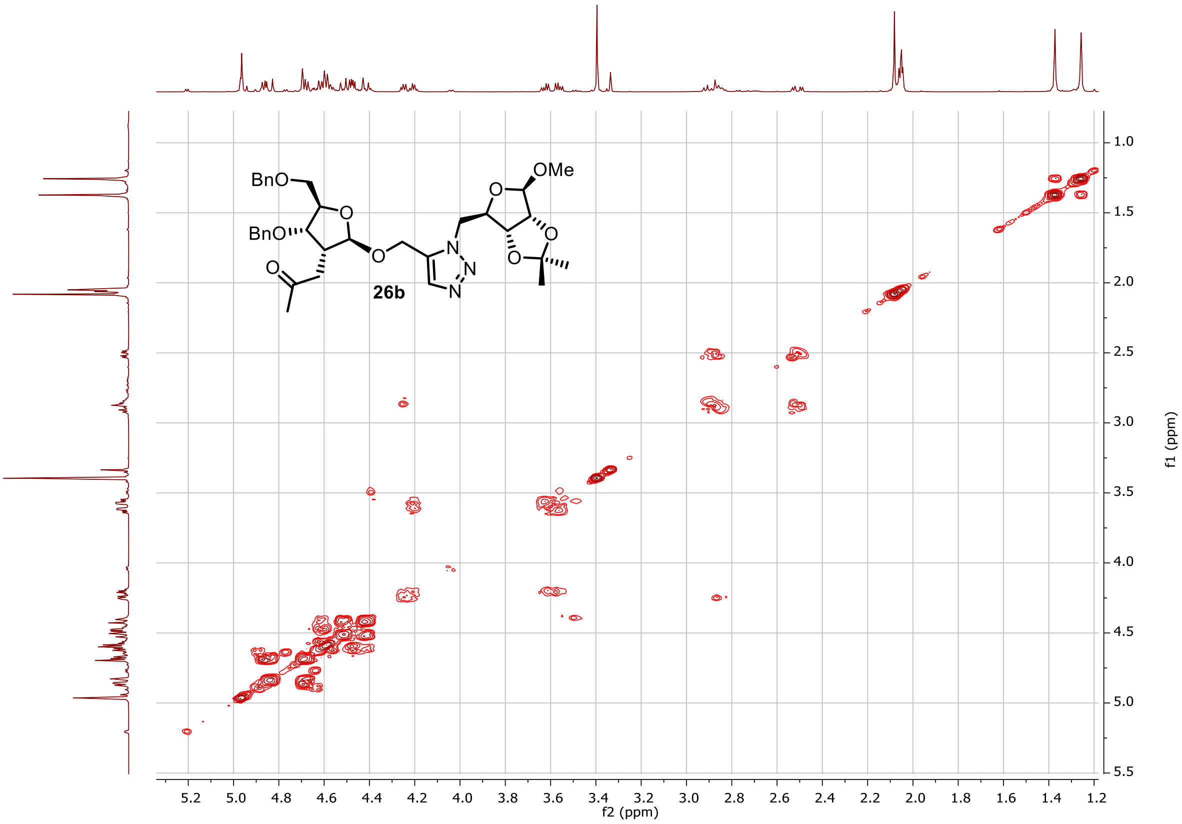

Supplementary Figure 404. COSY spectra for **26b**

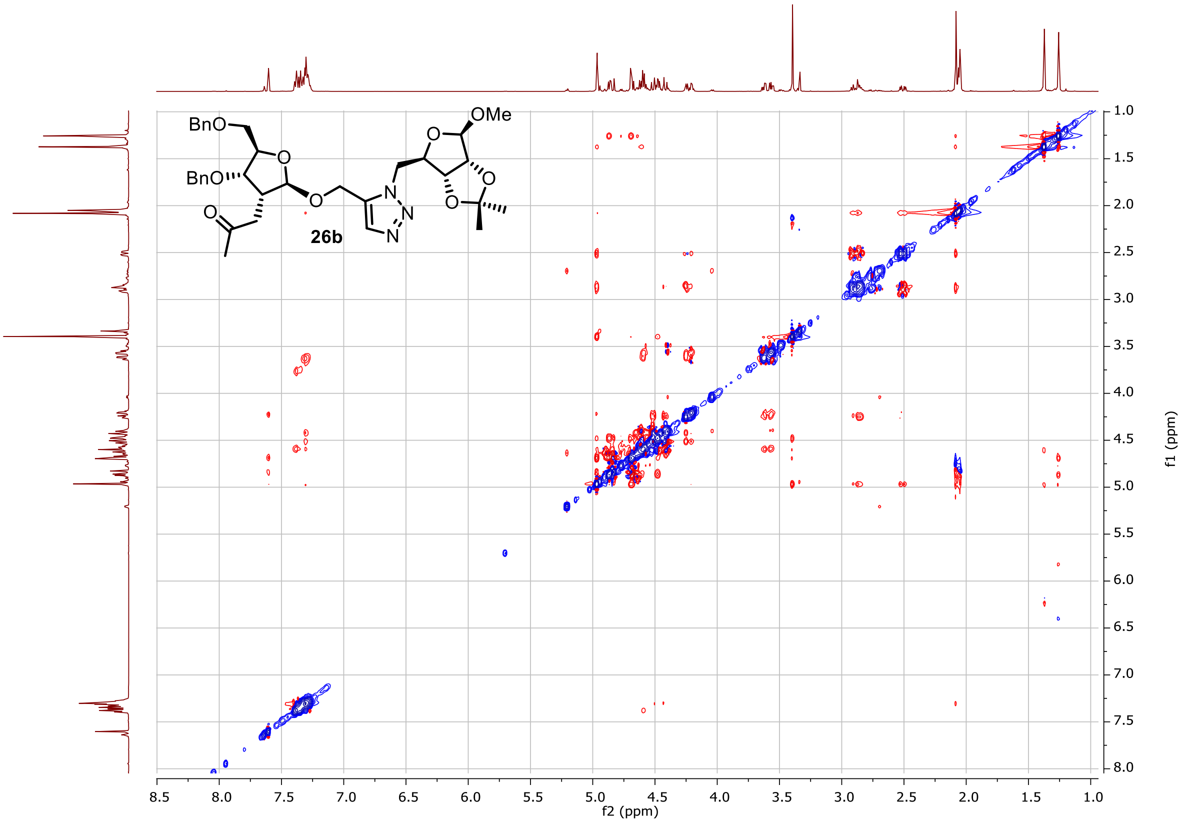

Supplementary Figure 405. NOESY spectra for **26b**

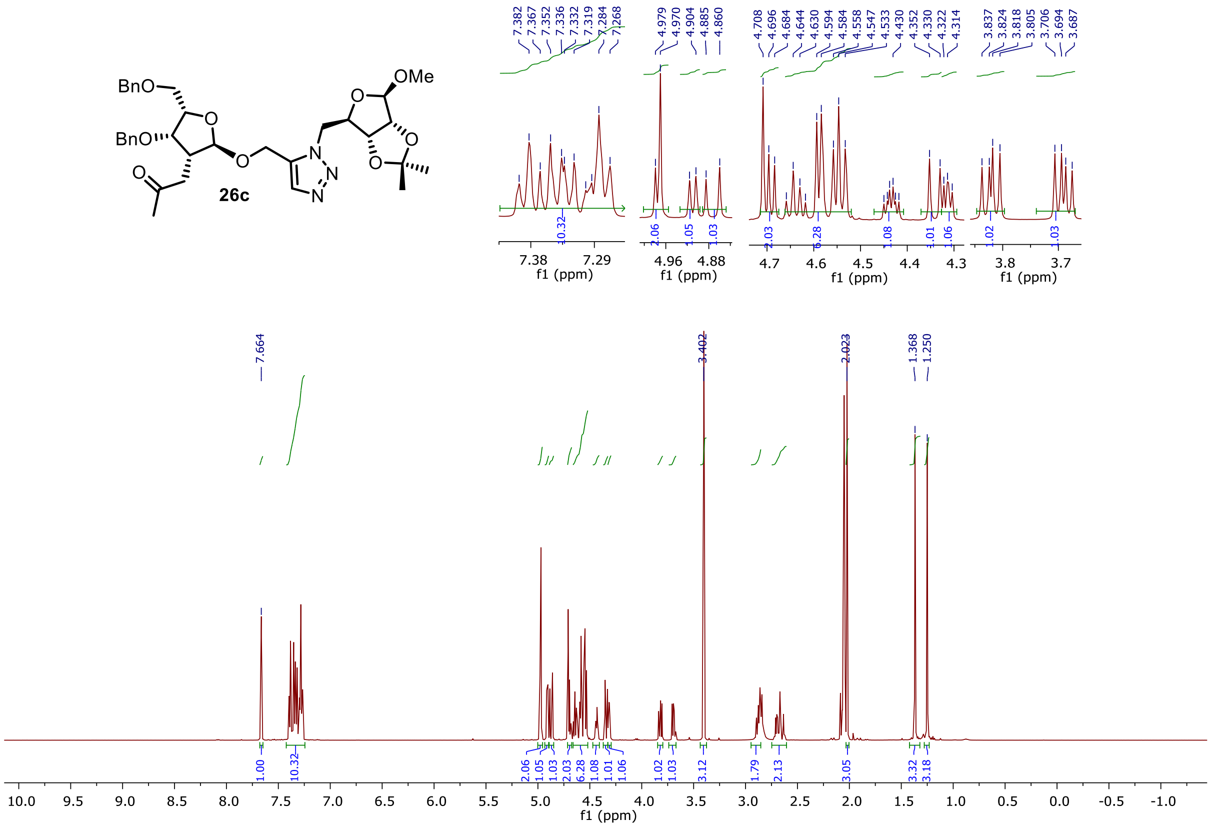

Supplementary Figure 406. ^{1}H spectra for **26c**

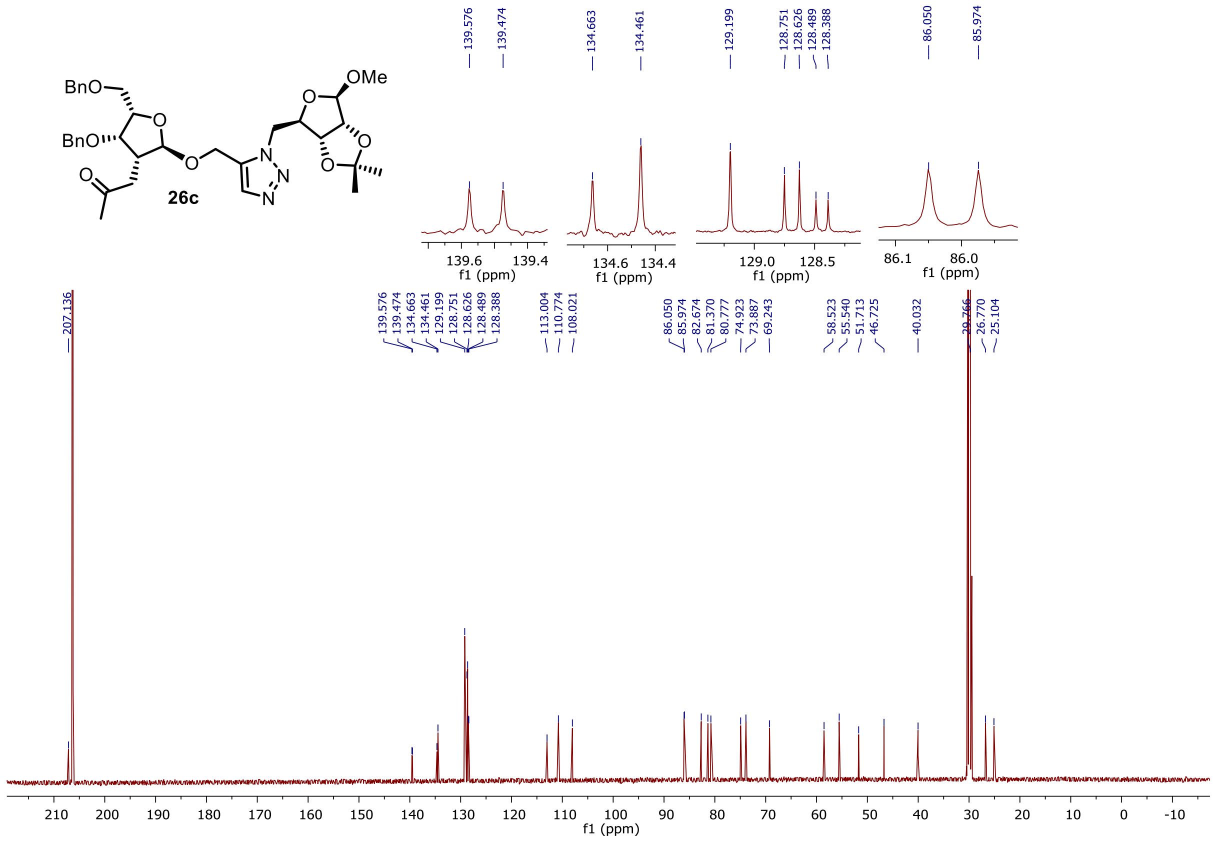

Supplementary Figure 407. ^{13}C spectra for **26c**

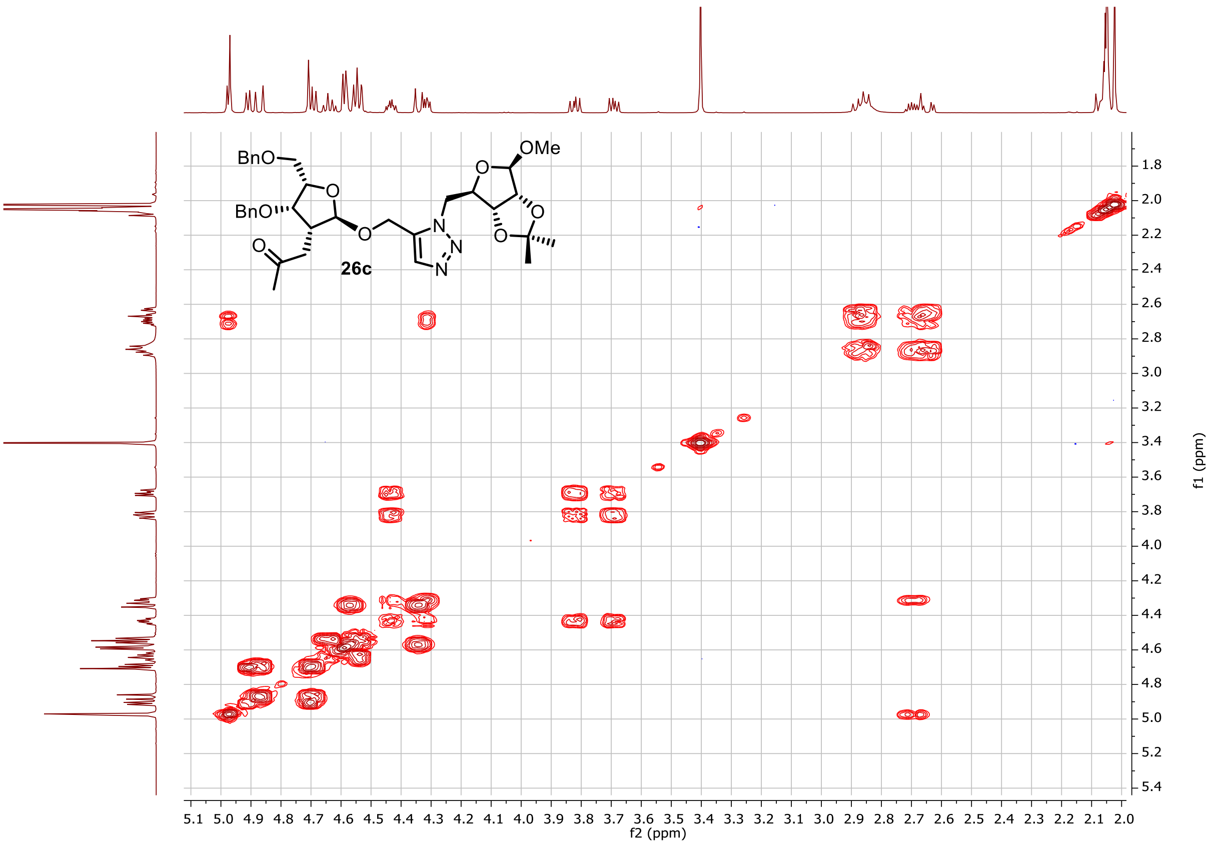

Supplementary Figure 408. COSY spectra for **26c**

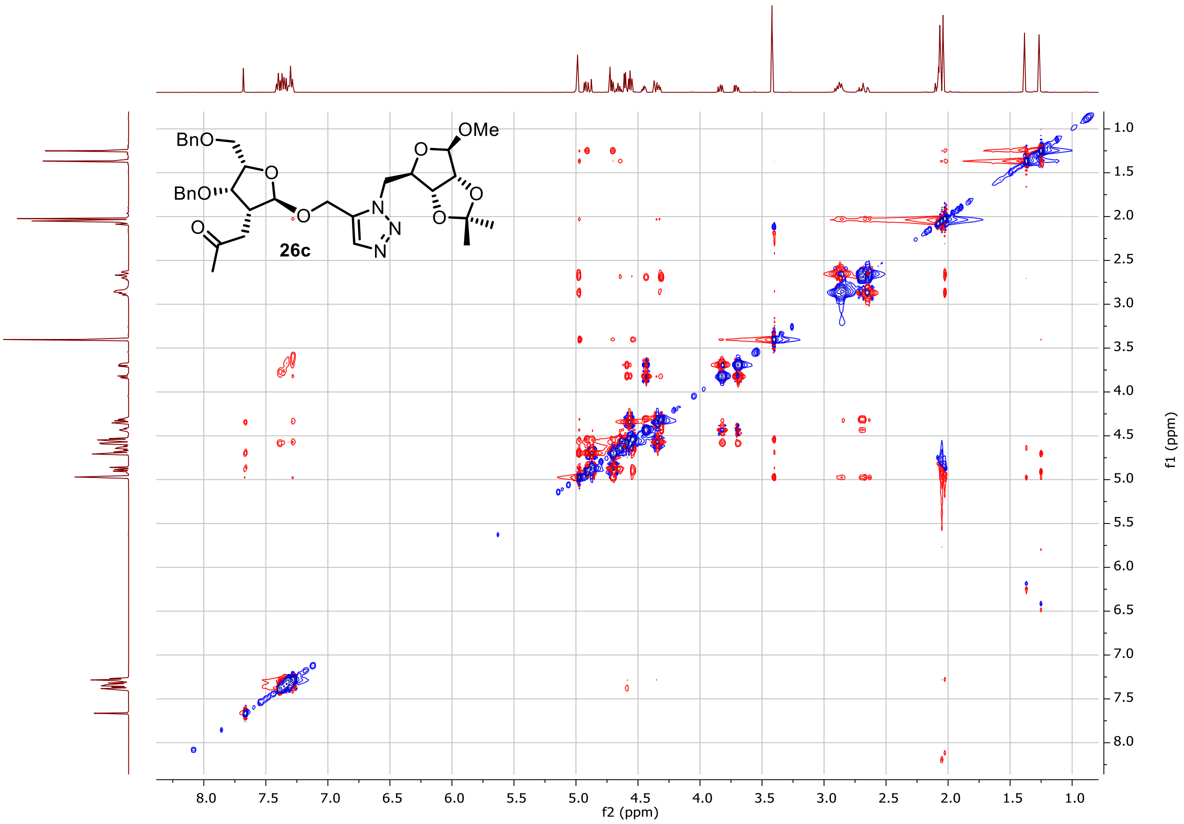

Supplementary Figure 409. NOESY spectra for **26c**

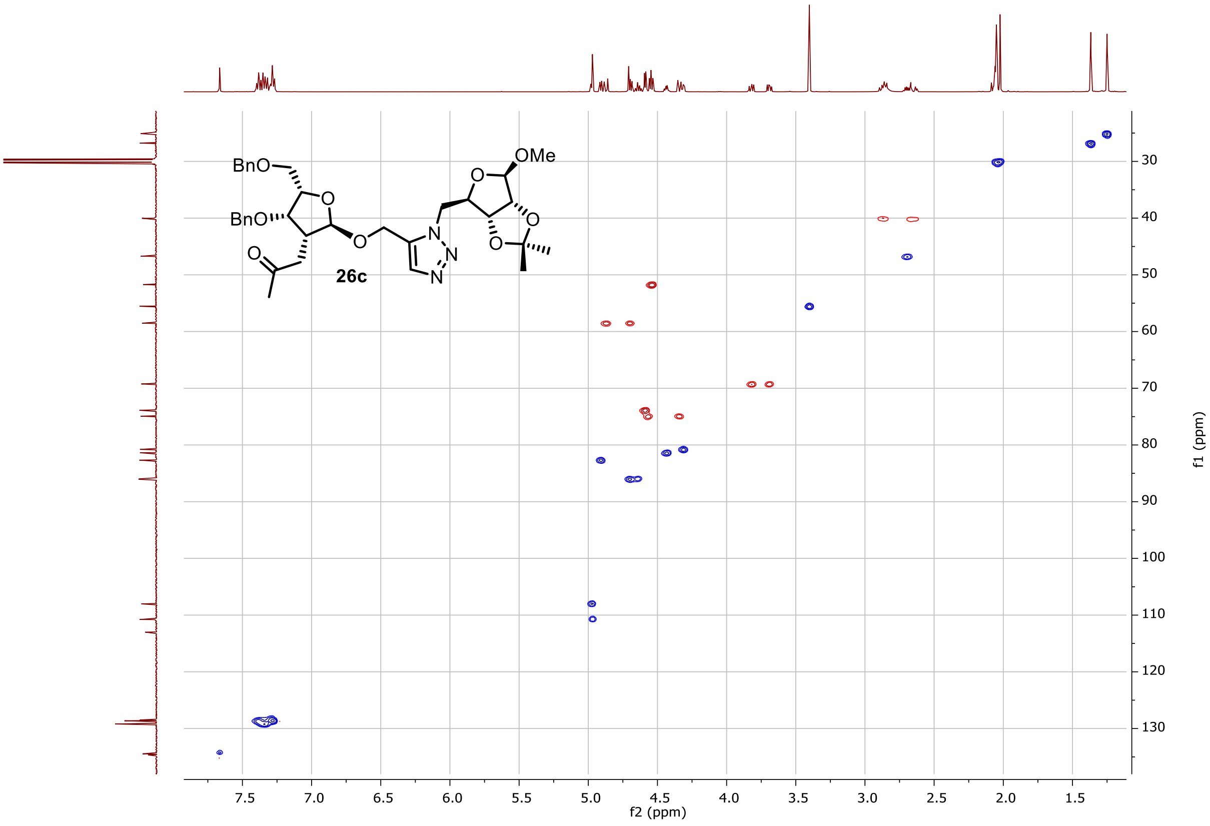

Supplementary Figure 410. HSQC spectra for **26c**

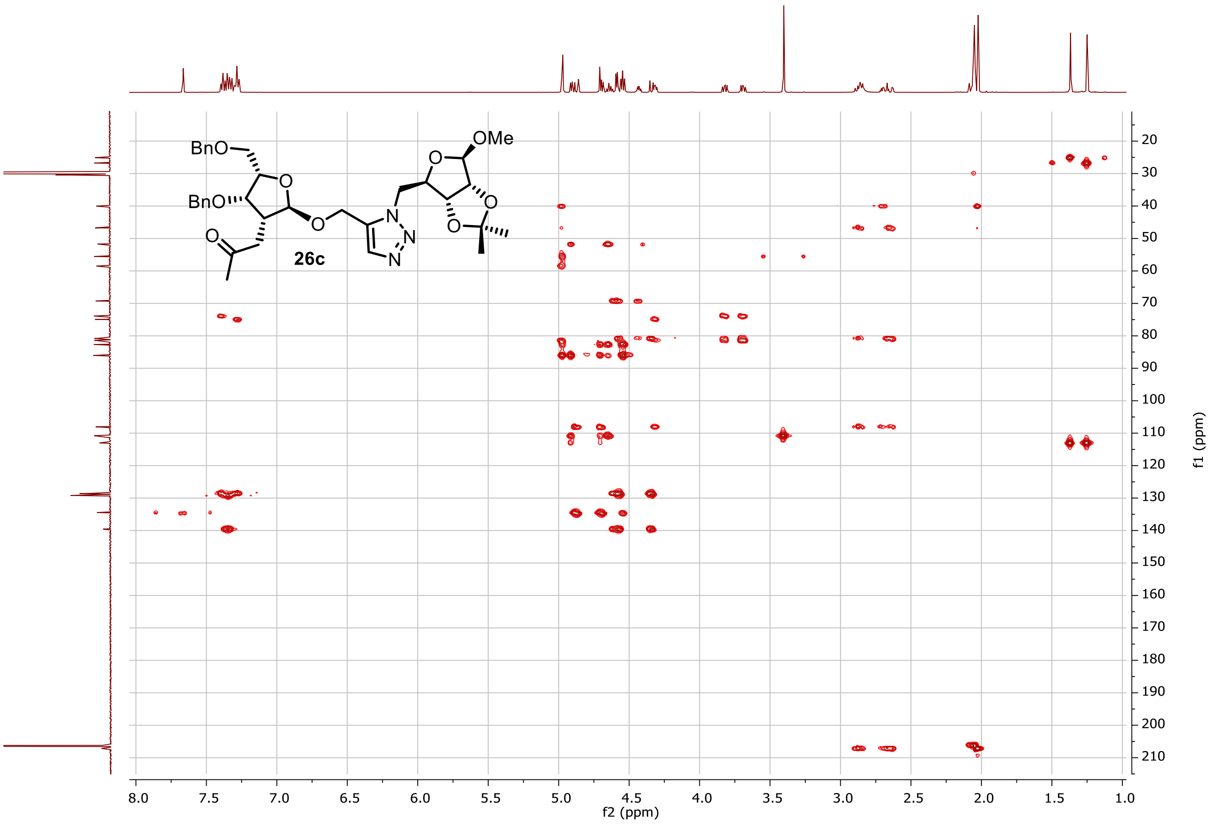

Supplementary Figure 411. HMBC spectra for **26c**

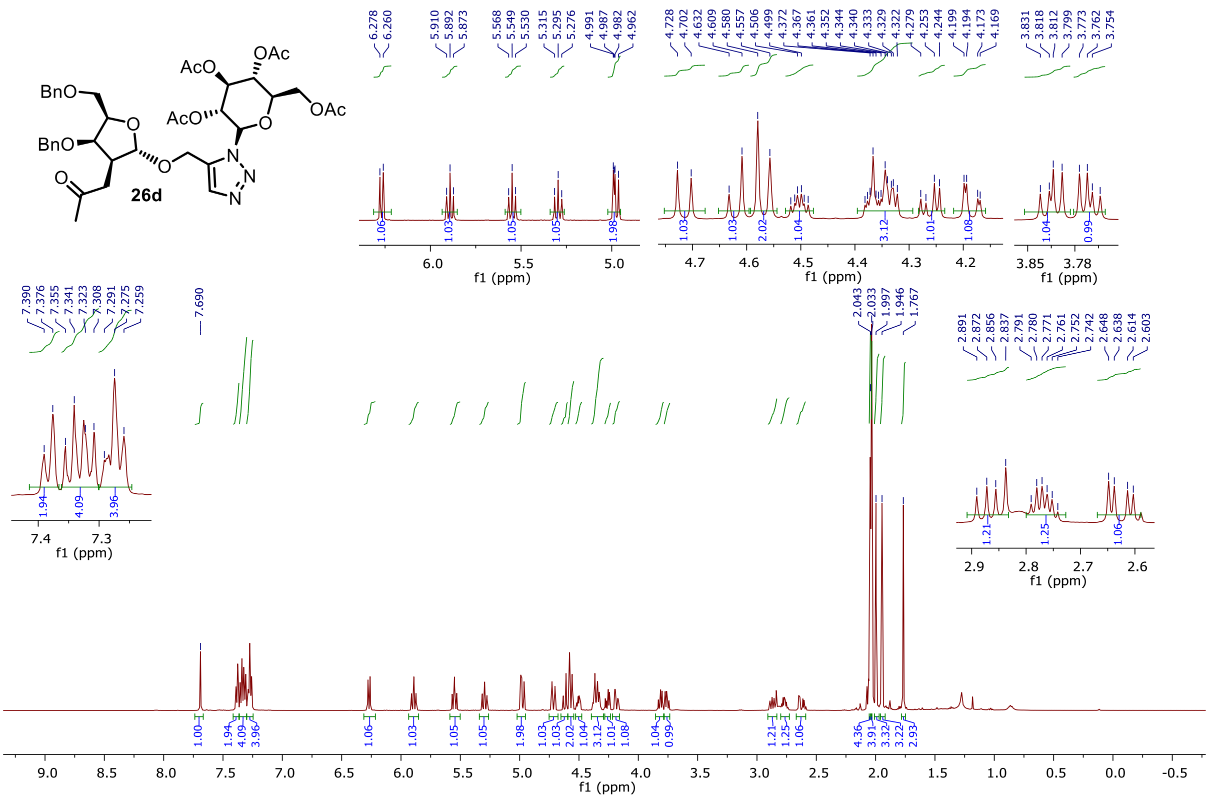

Supplementary Figure 412. ^{1}H spectra for **26d**

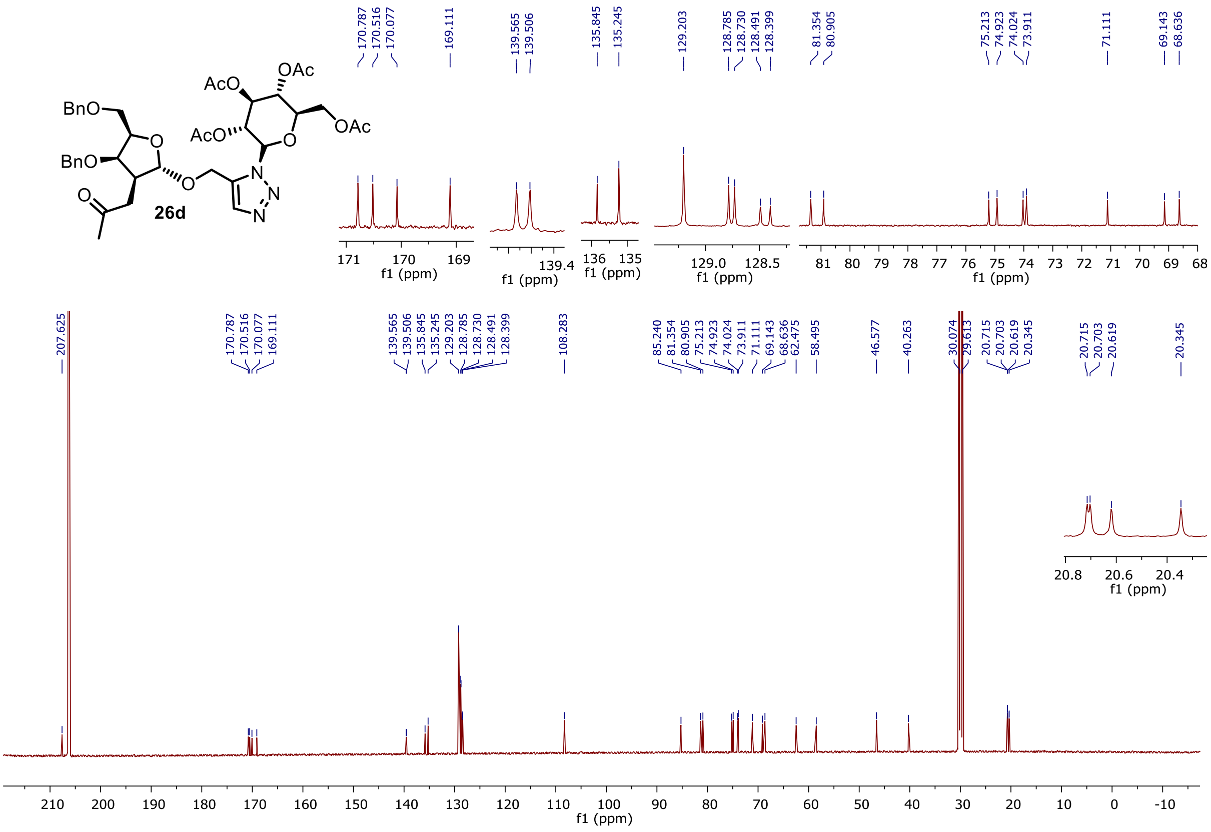

Supplementary Figure 413. ^{13}C spectra for **26d**

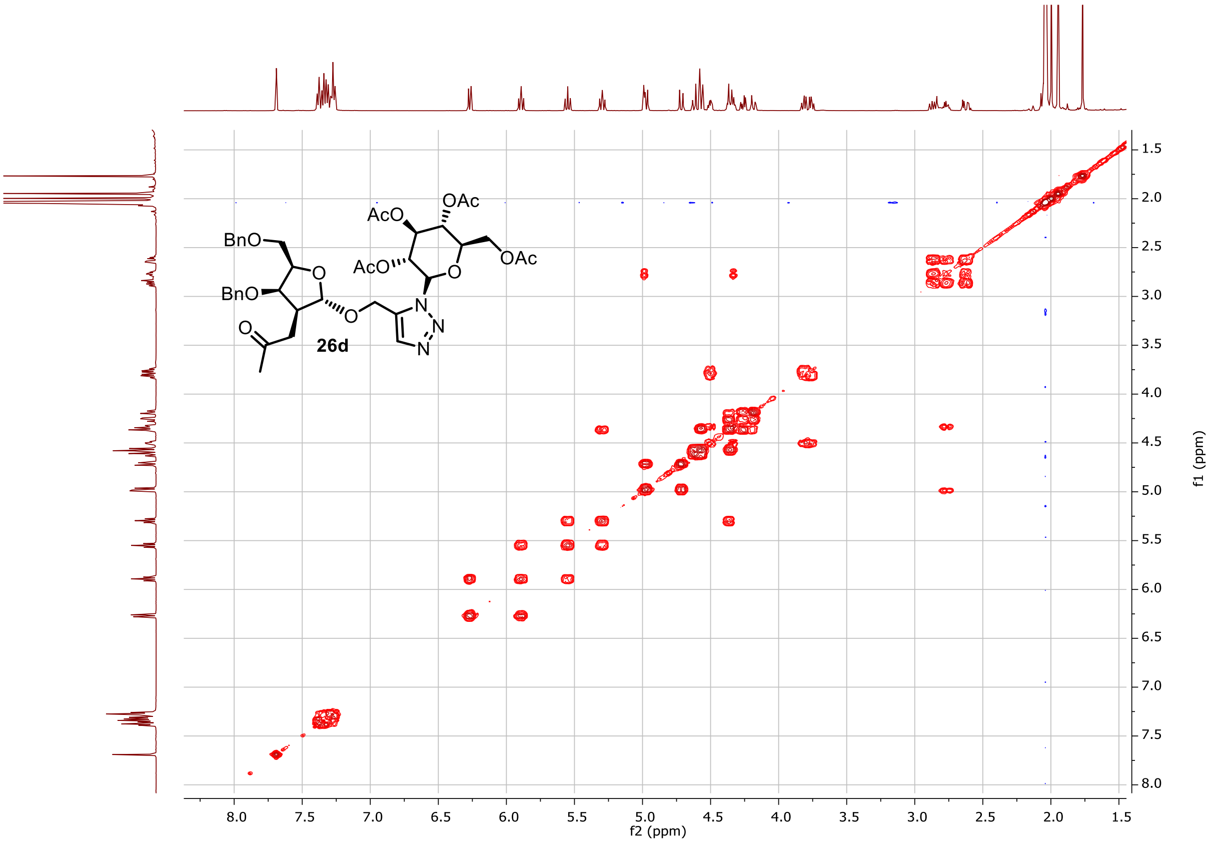

Supplementary Figure 414. COSY spectra for **26d**

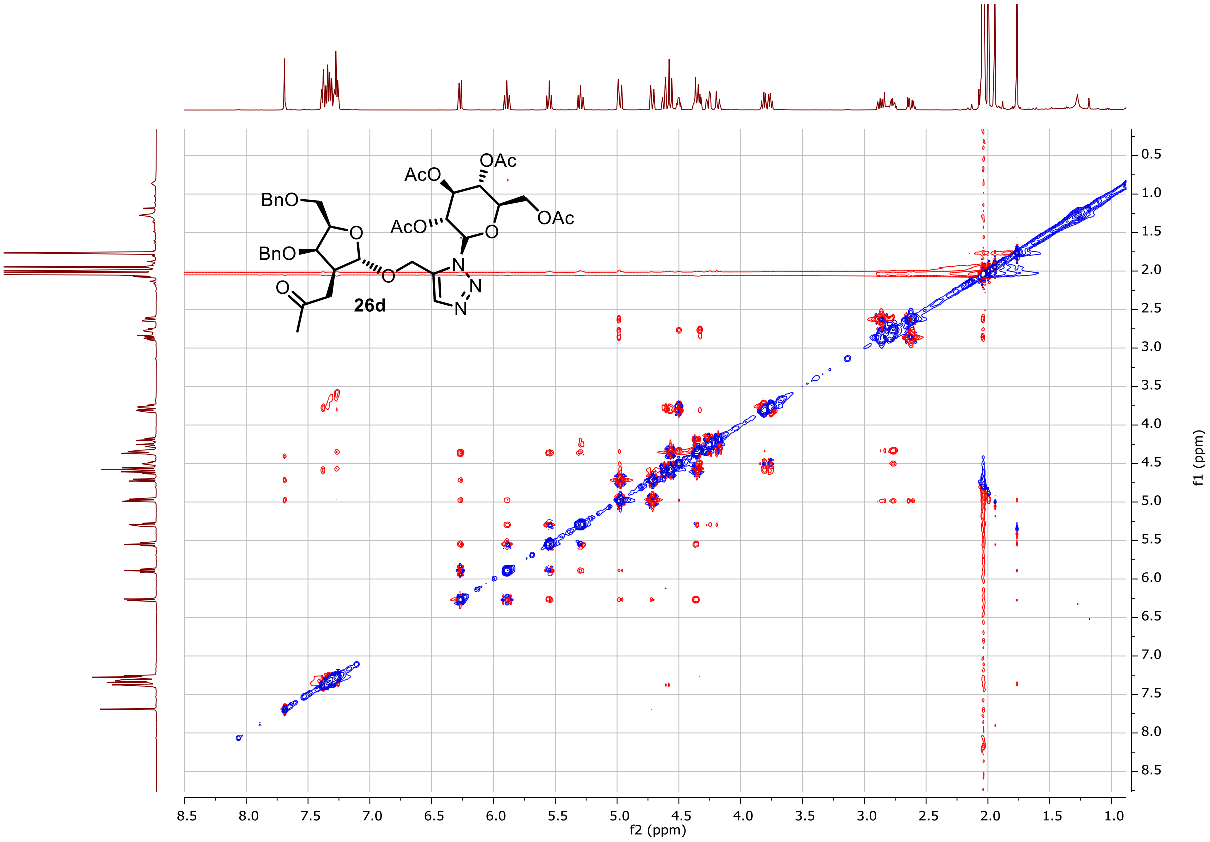

Supplementary Figure 415. NOESY spectra for **26d**

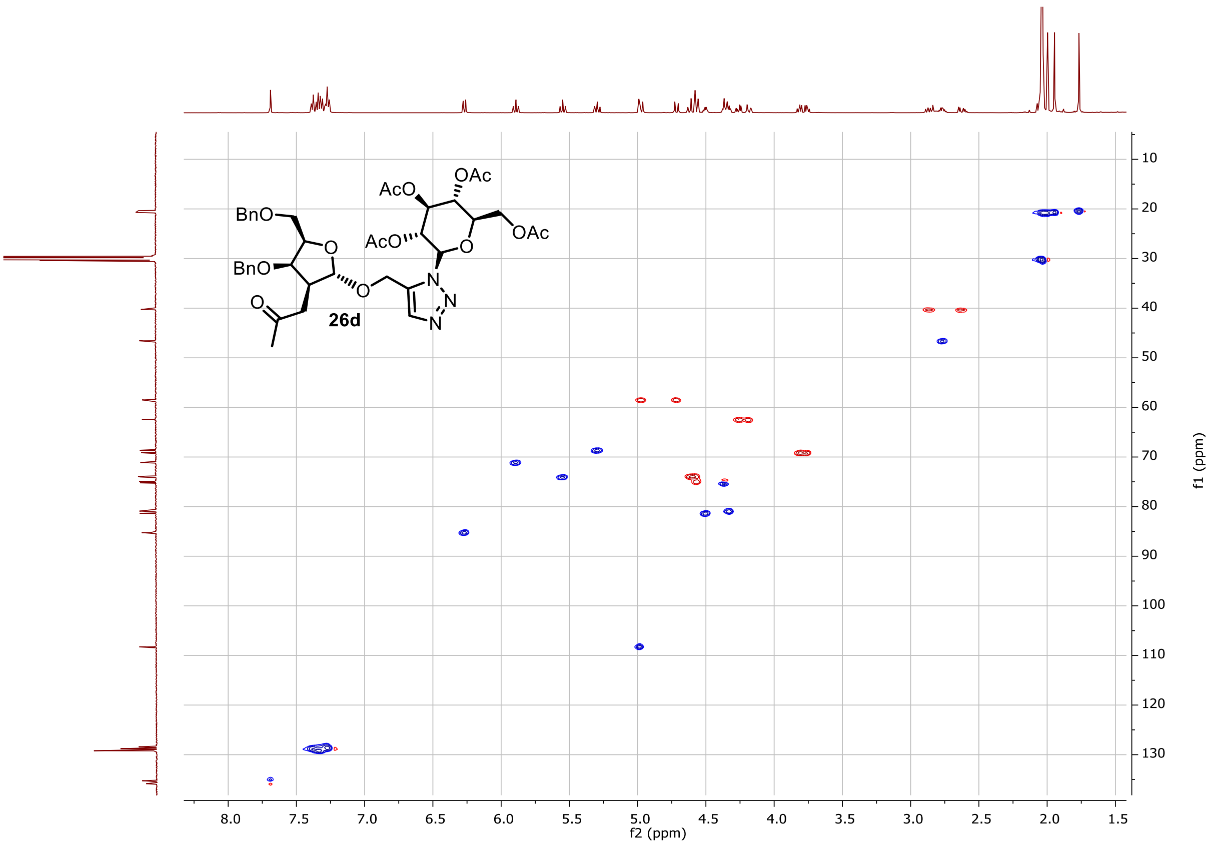

Supplementary Figure 416. HSQC spectra for **26d**

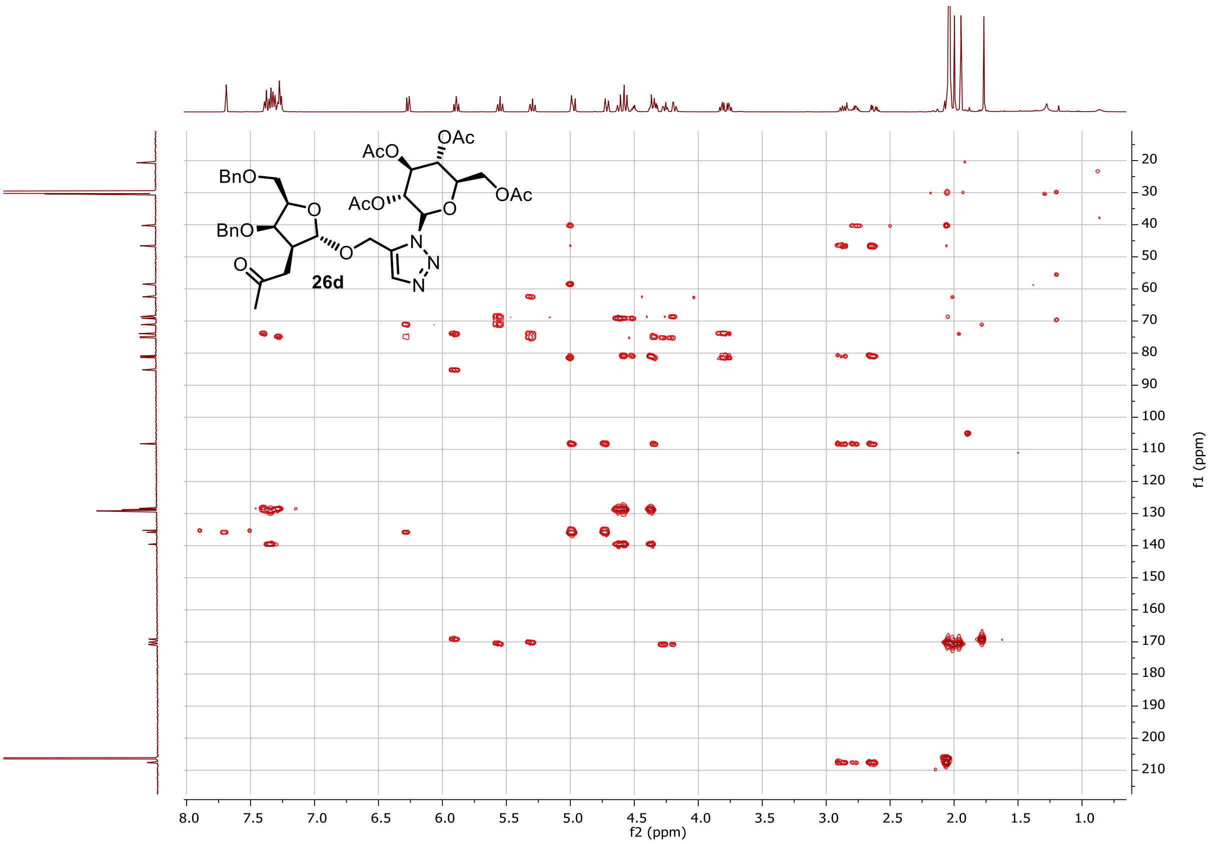

Supplementary Figure 417. HMBC spectra for **26d**

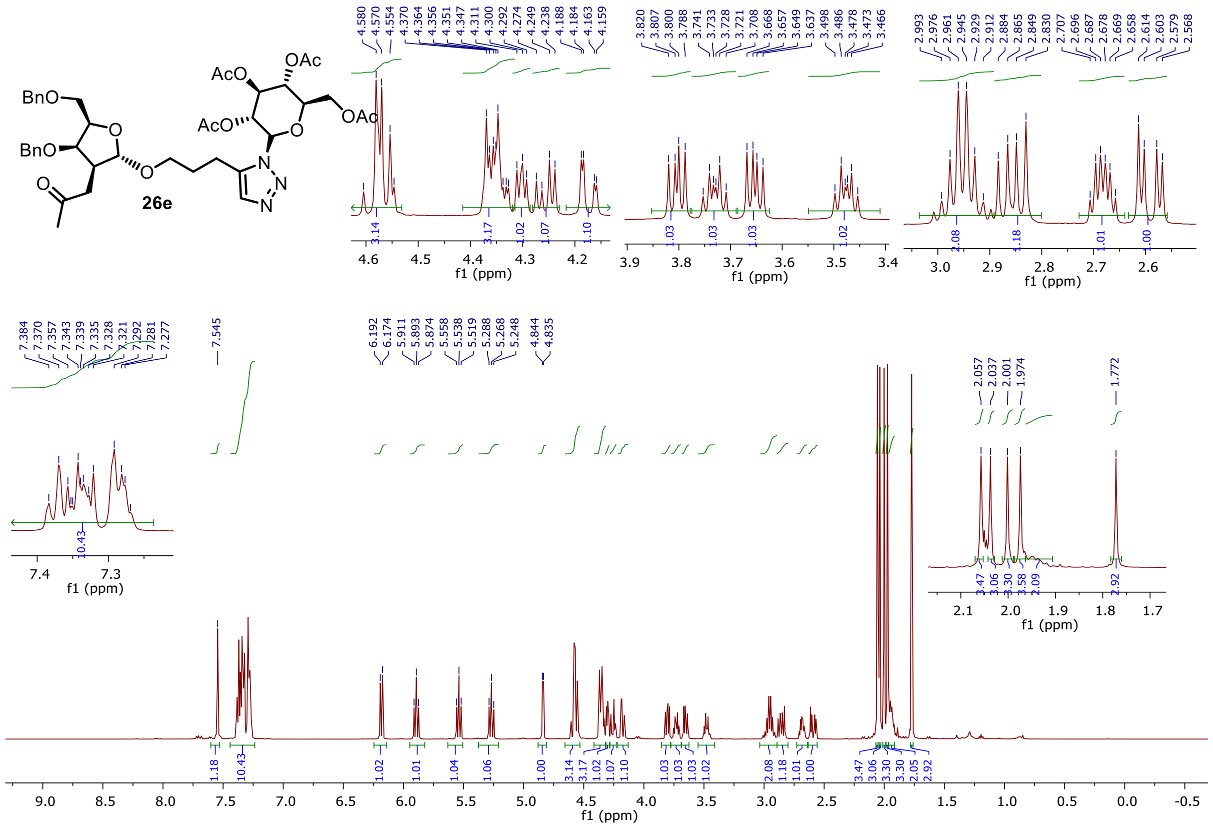

Supplementary Figure 418. ^{1}H spectra for **26e**

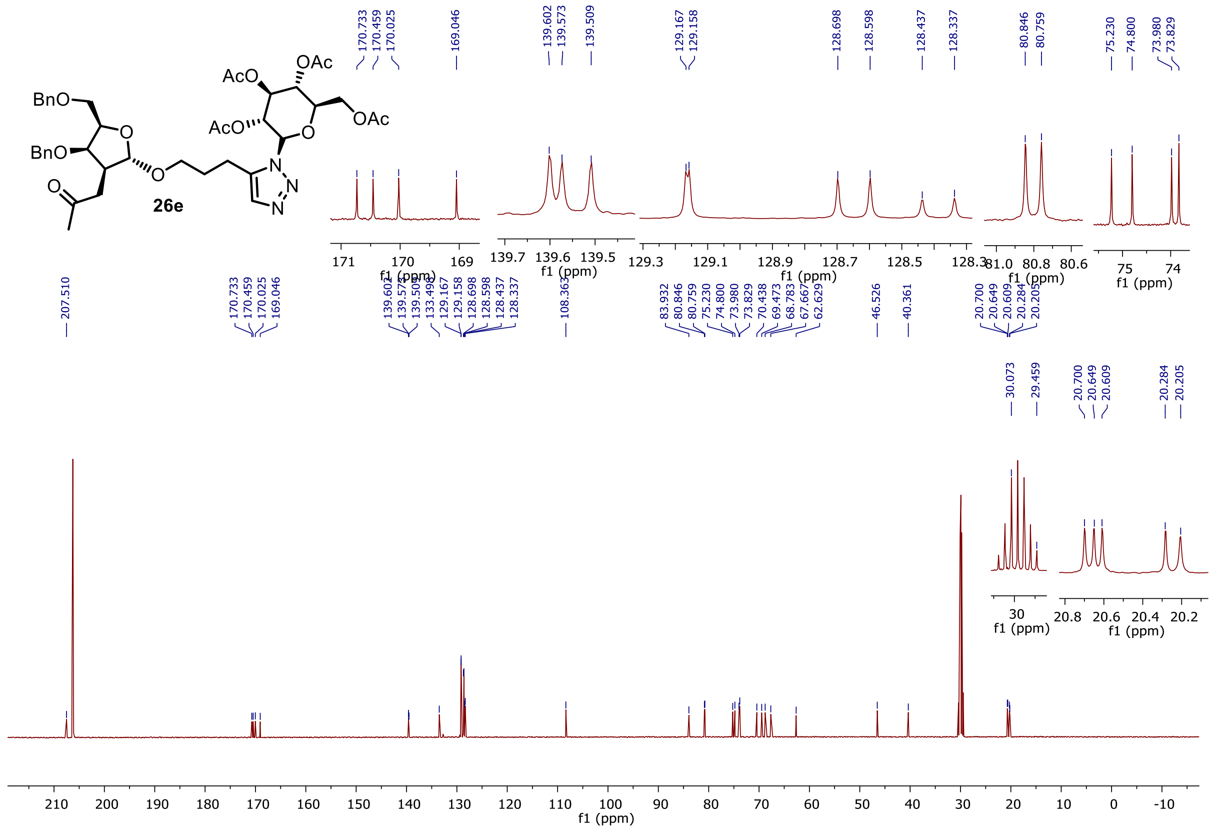

Supplementary Figure 419. ^{13}C spectra for **26e**

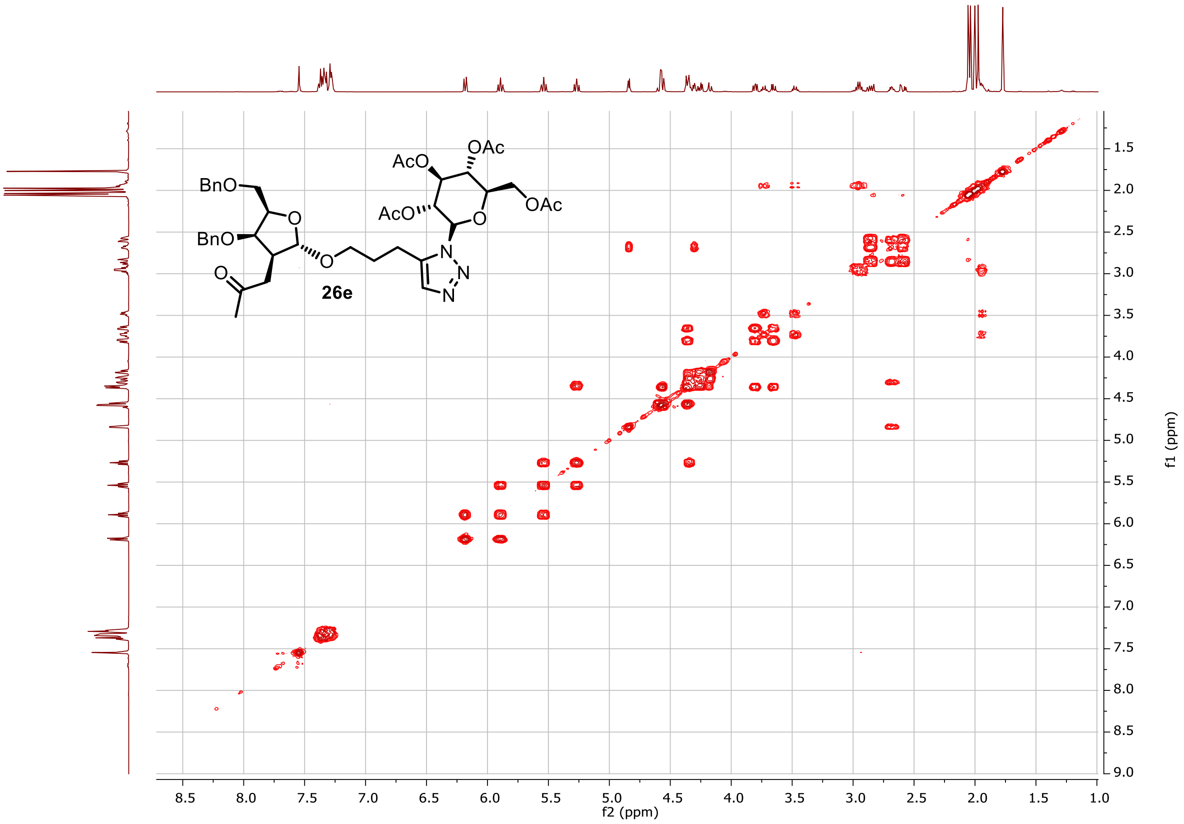

Supplementary Figure 420. COSY spectra for **26e**

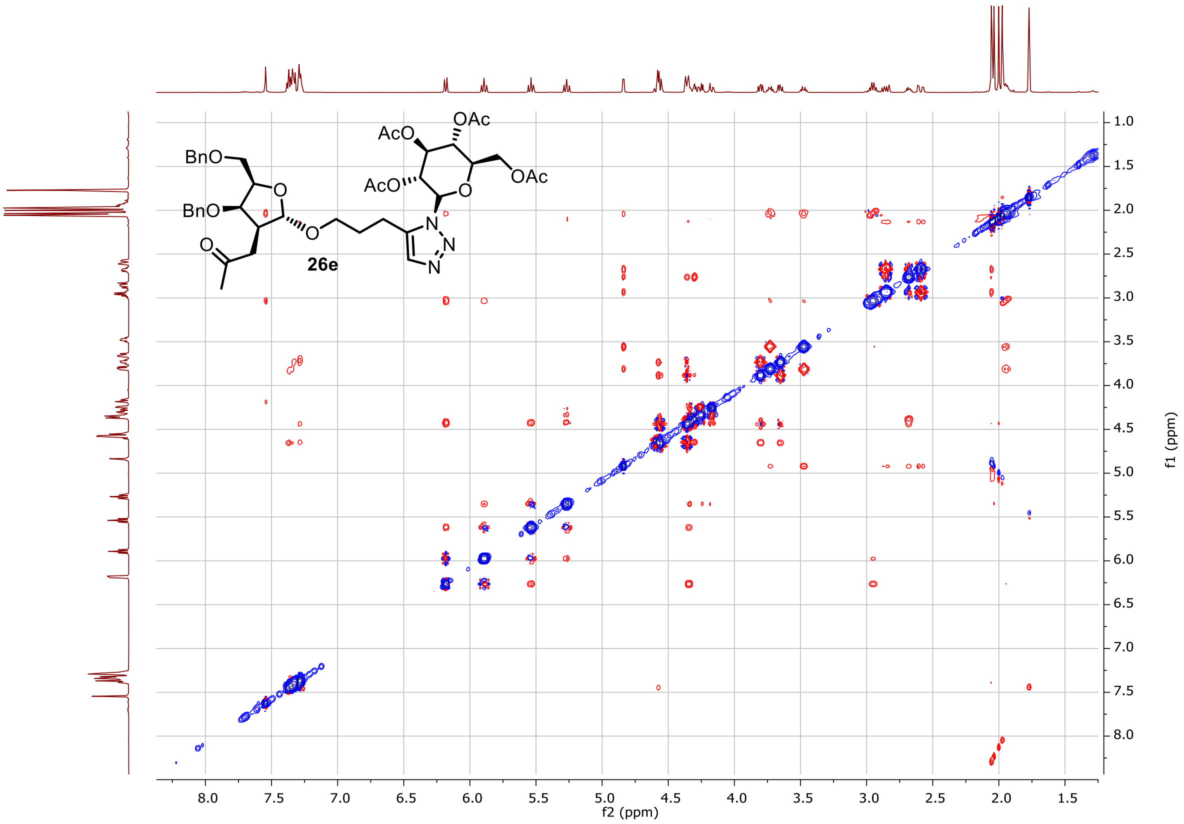

Supplementary Figure 421. NOESY spectra for **26e**

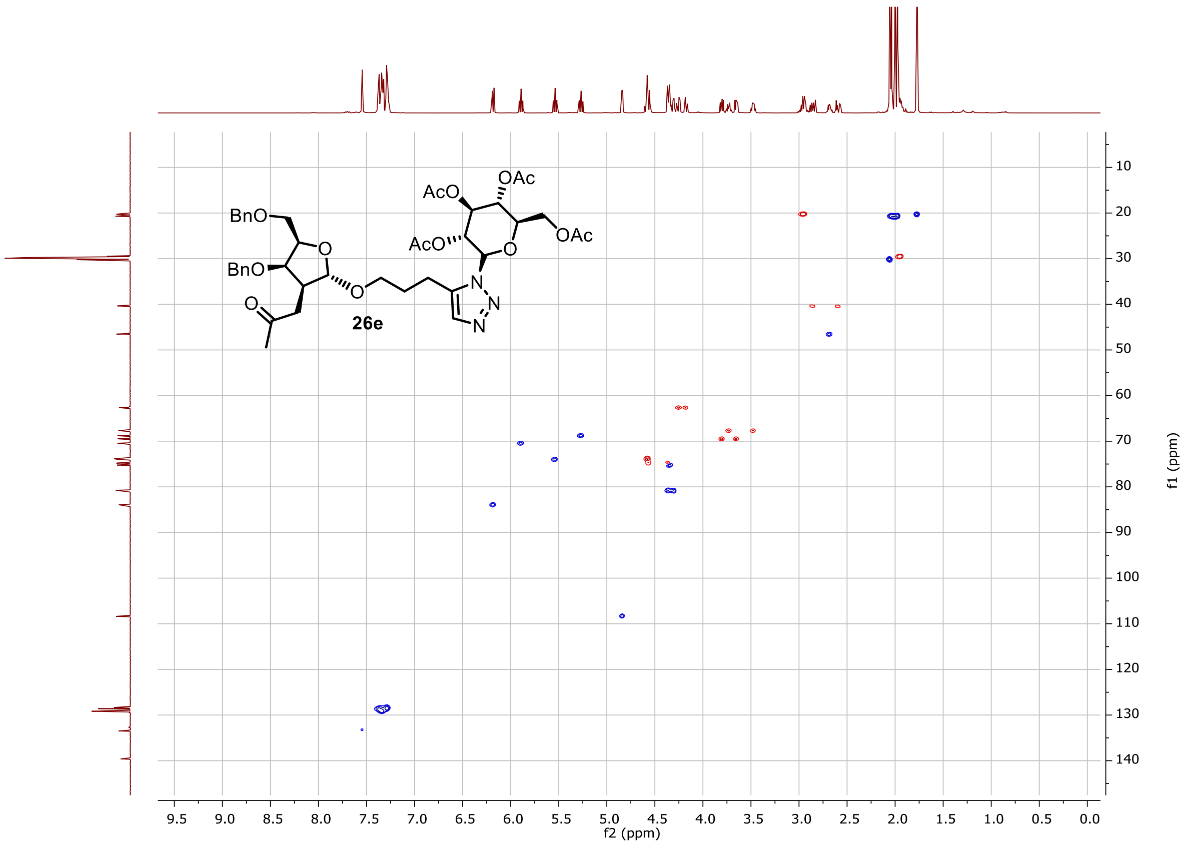

Supplementary Figure 422. HSQC spectra for **26e**

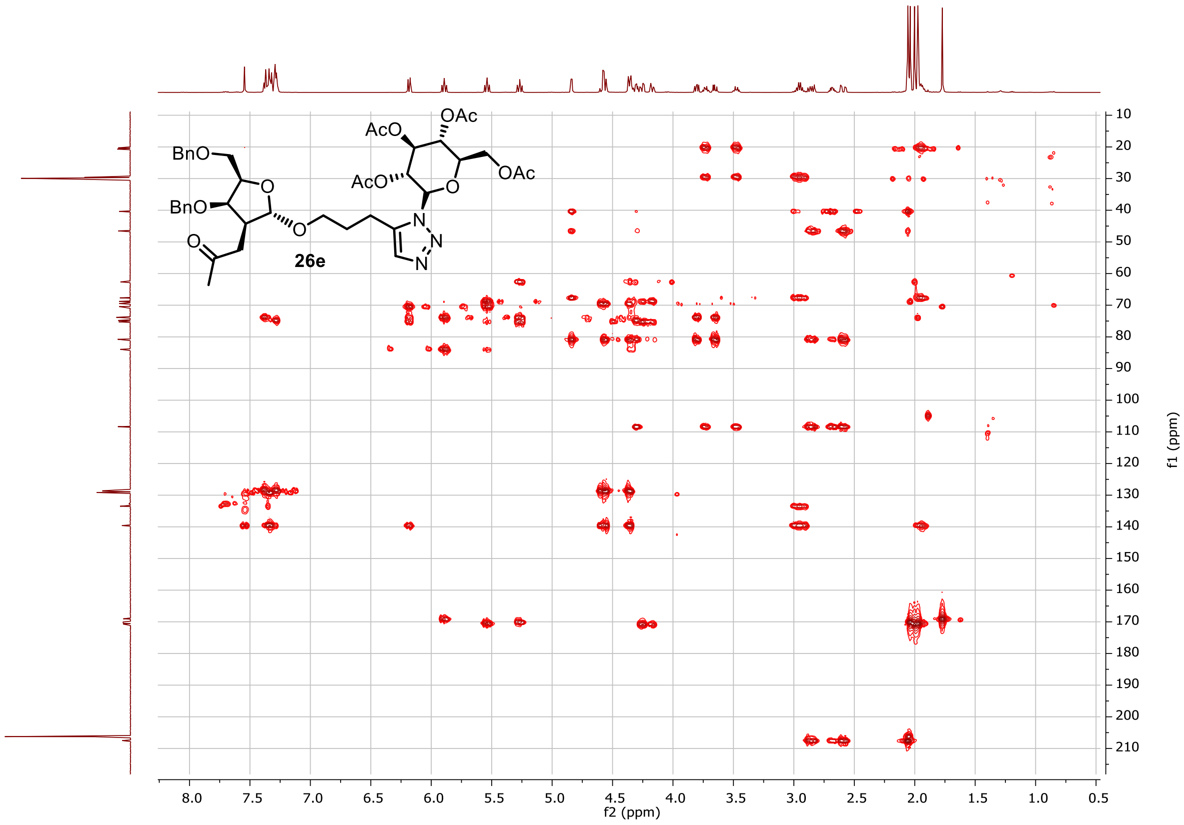

Supplementary Figure 423. HMBC spectra for **26e**

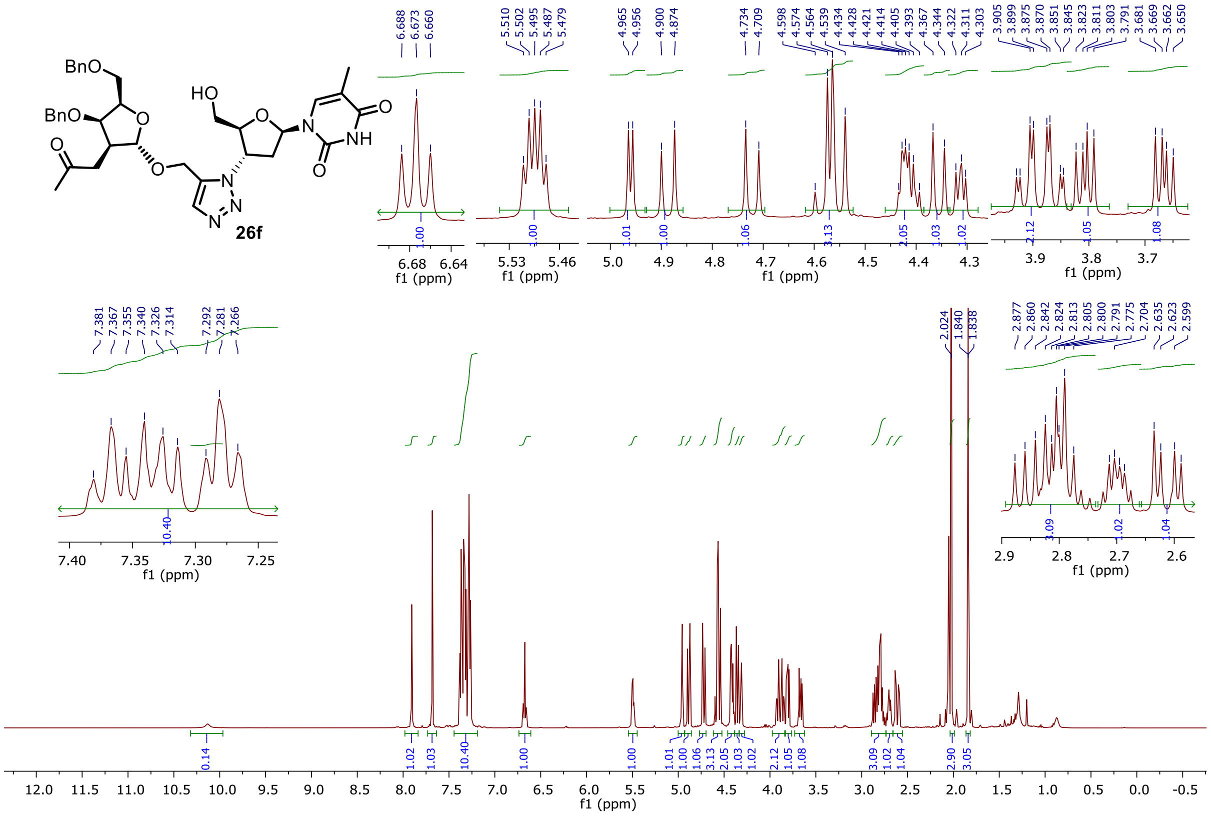

Supplementary Figure 424. ^{1}H spectra for **26f**

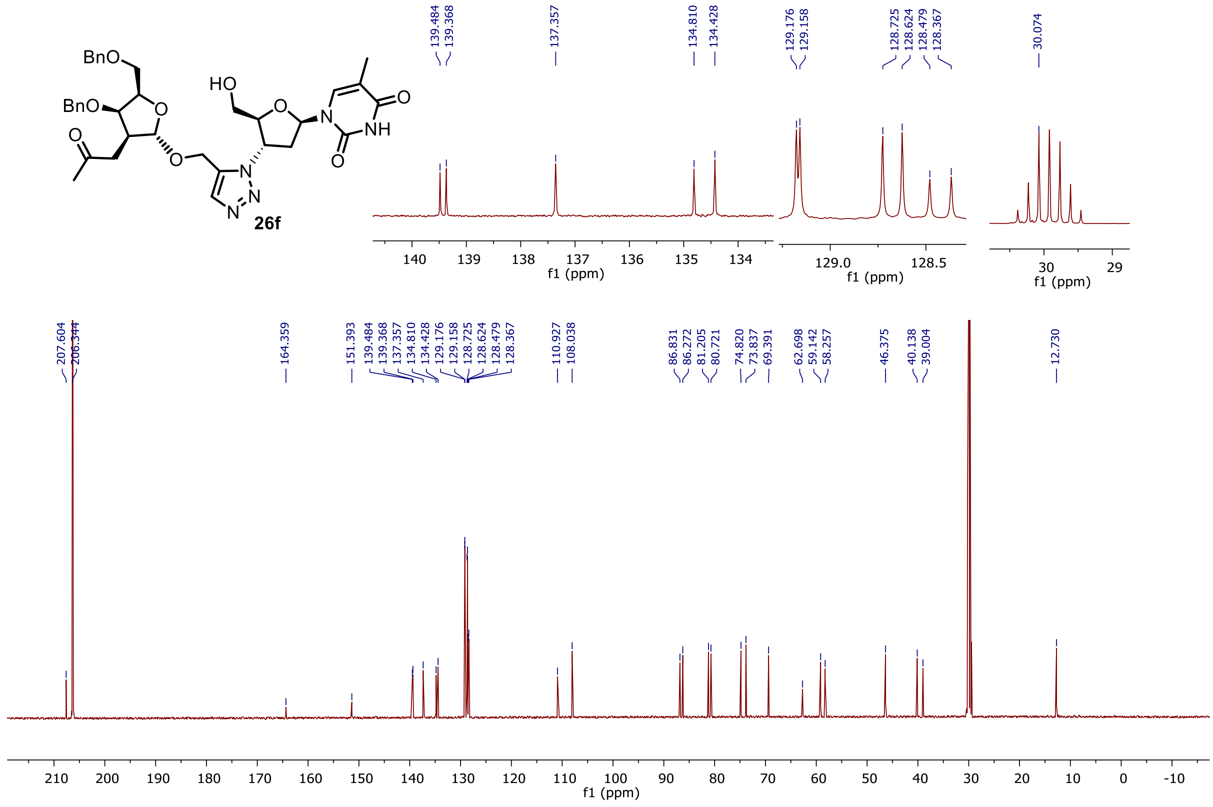

Supplementary Figure 425. ^{13}C spectra for **26f**

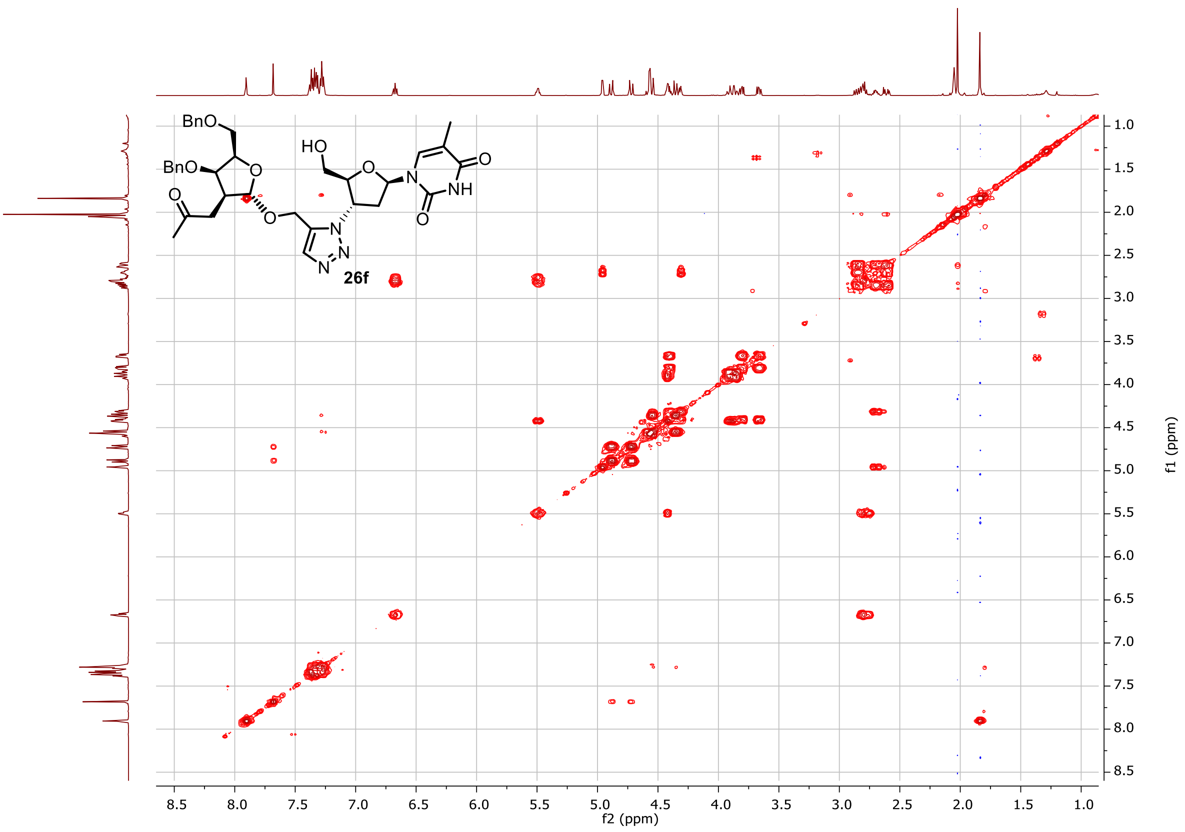

Supplementary Figure 426. COSY spectra for **26f**

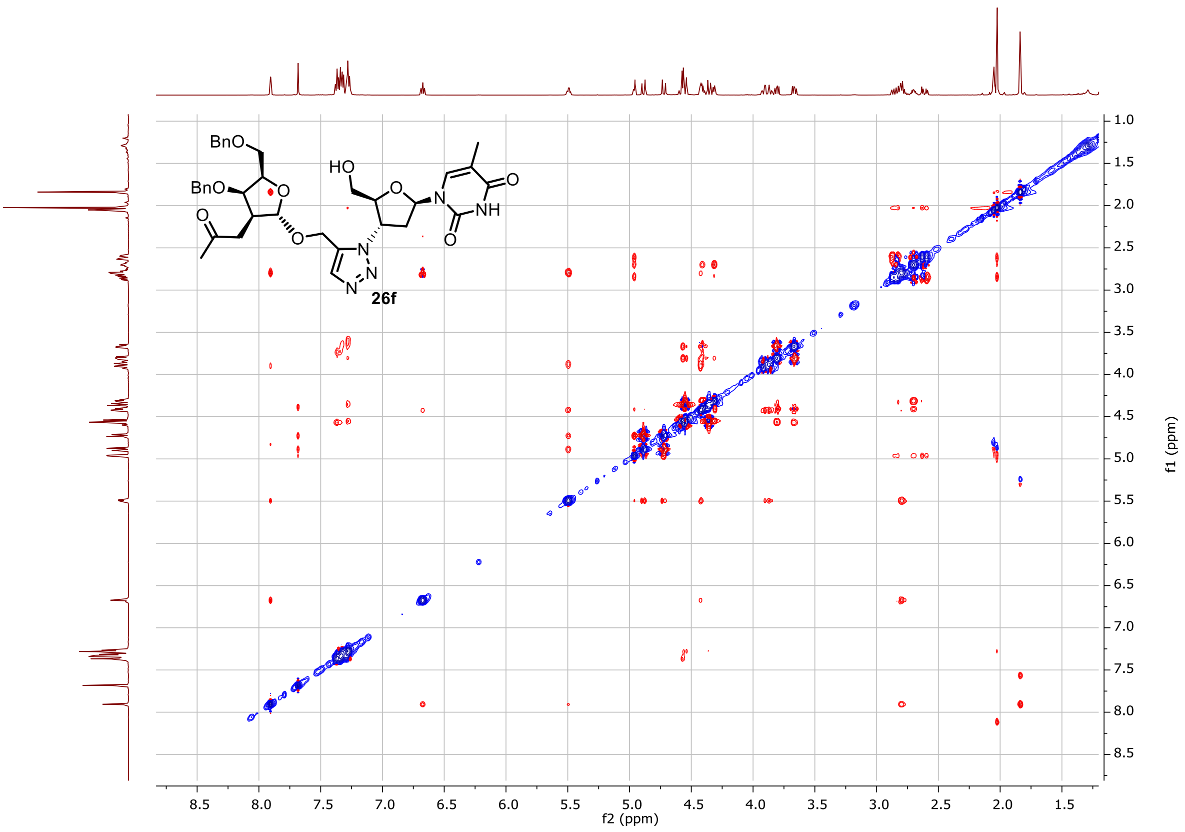

Supplementary Figure 427. NOESY spectra for **26f**

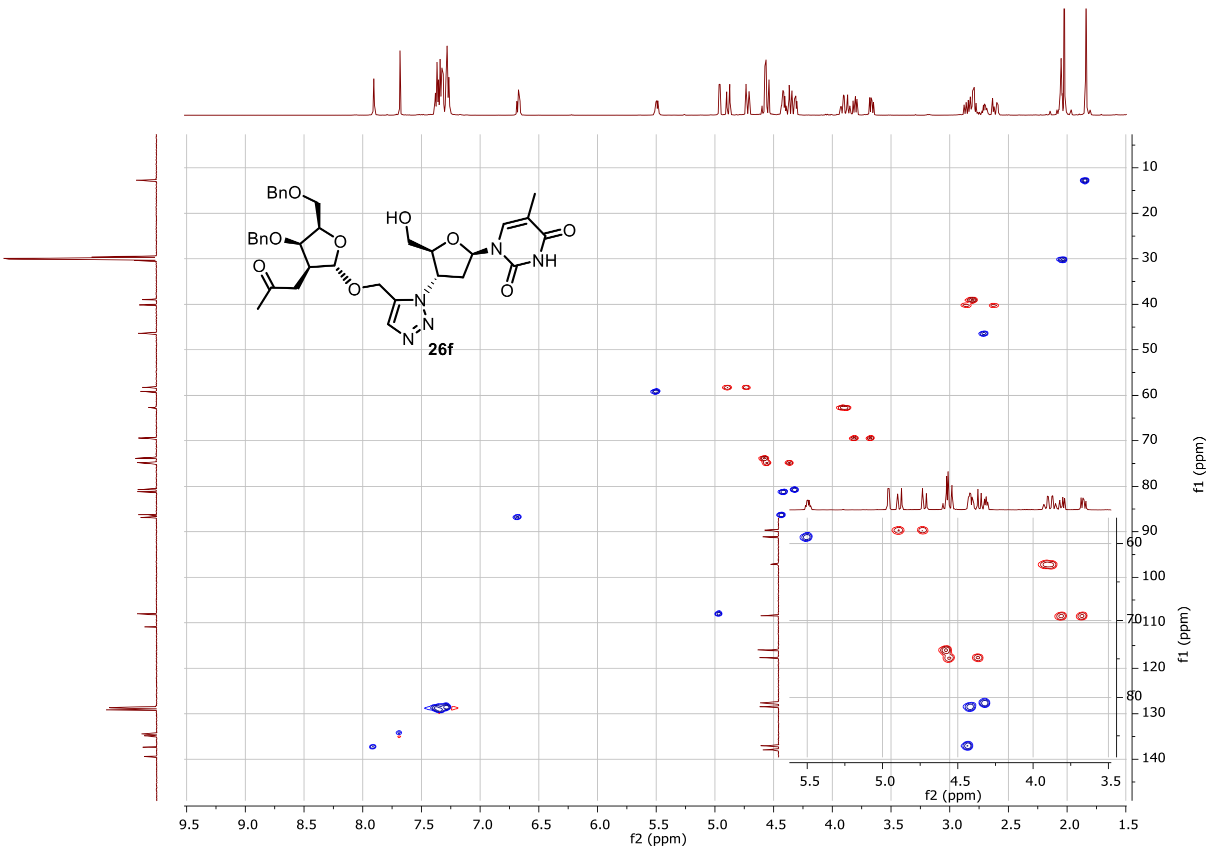

Supplementary Figure 428. HSQC spectra for **26f**

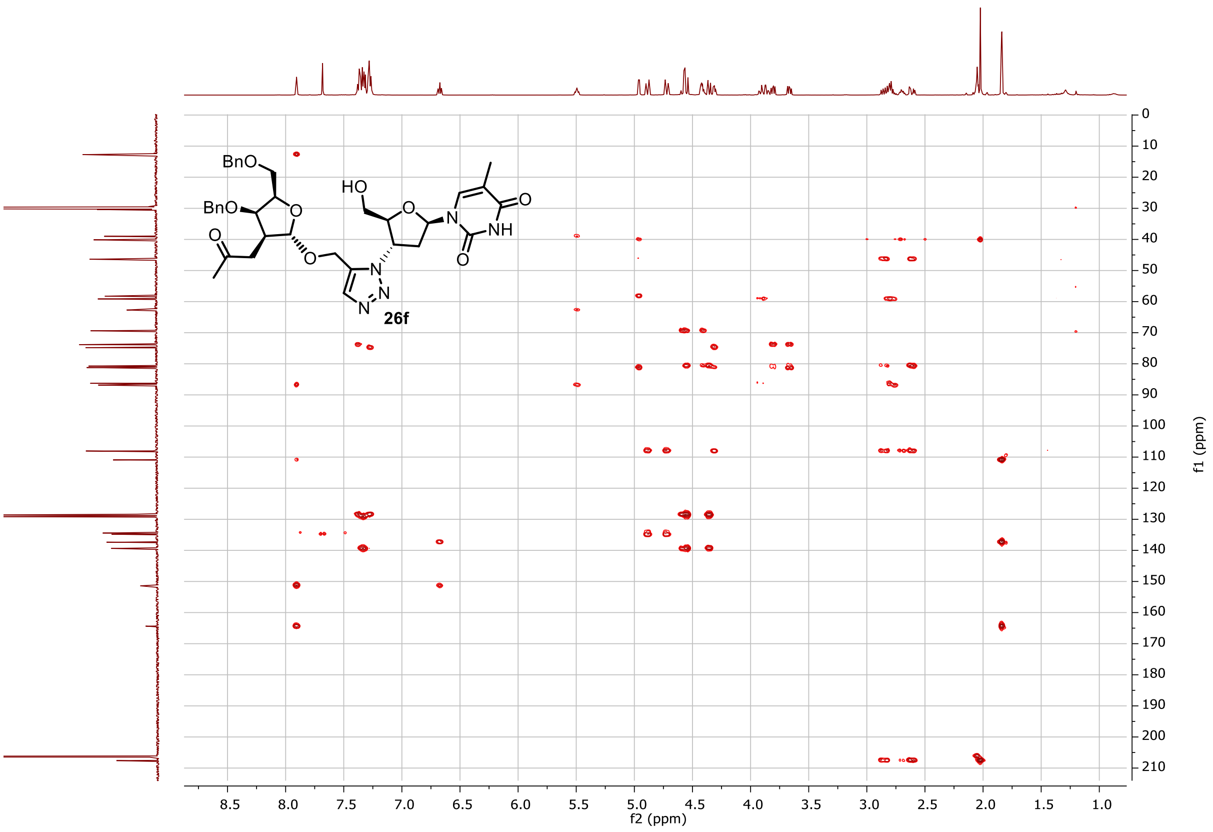

Supplementary Figure 429. HMBC spectra for **26f**

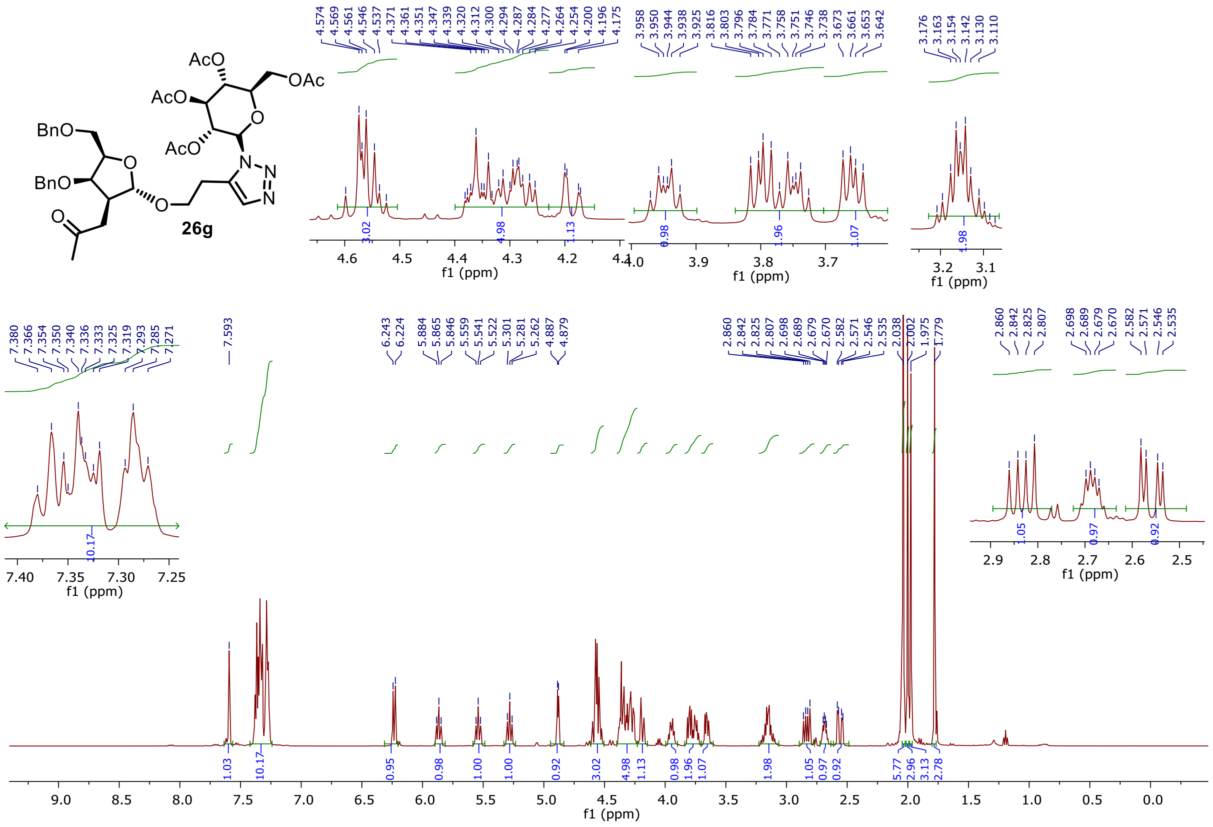

Supplementary Figure 430. ^{1}H spectra for **26g**

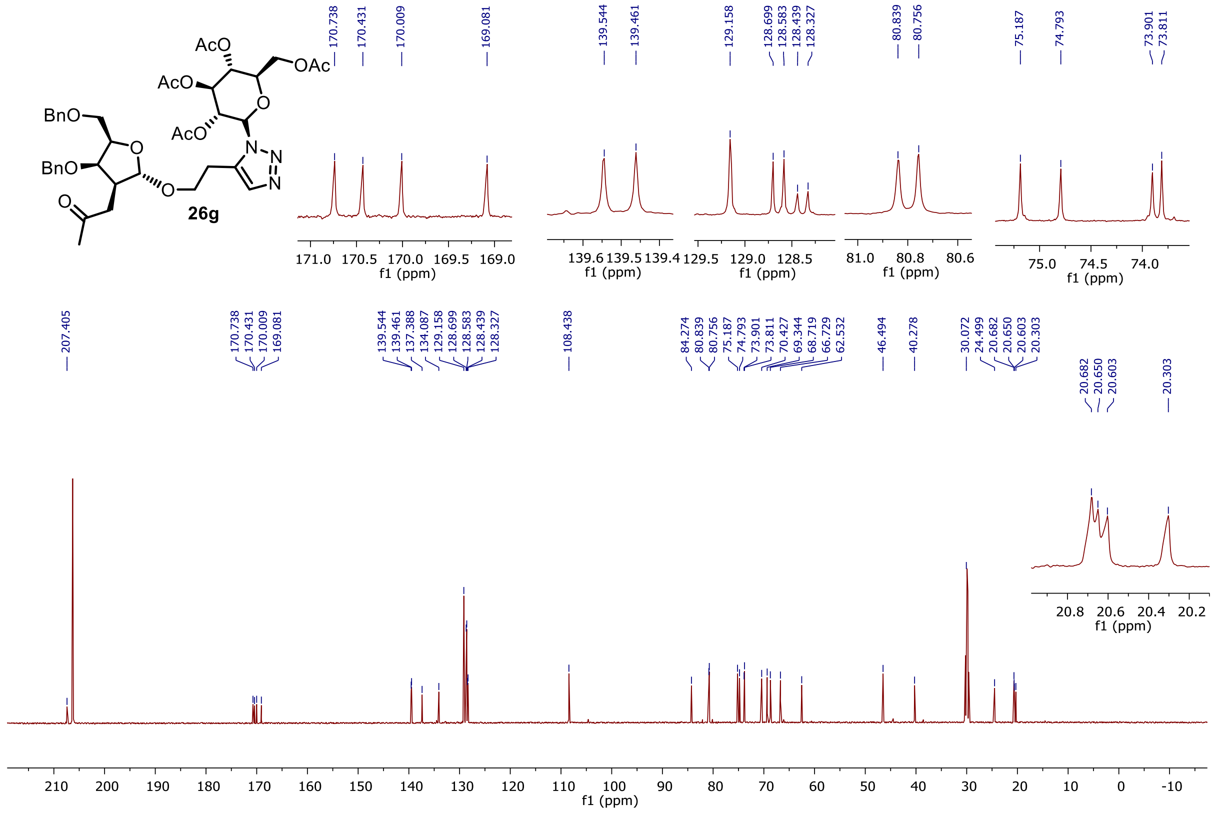

Supplementary Figure 431. ^{13}C spectra for **26g**

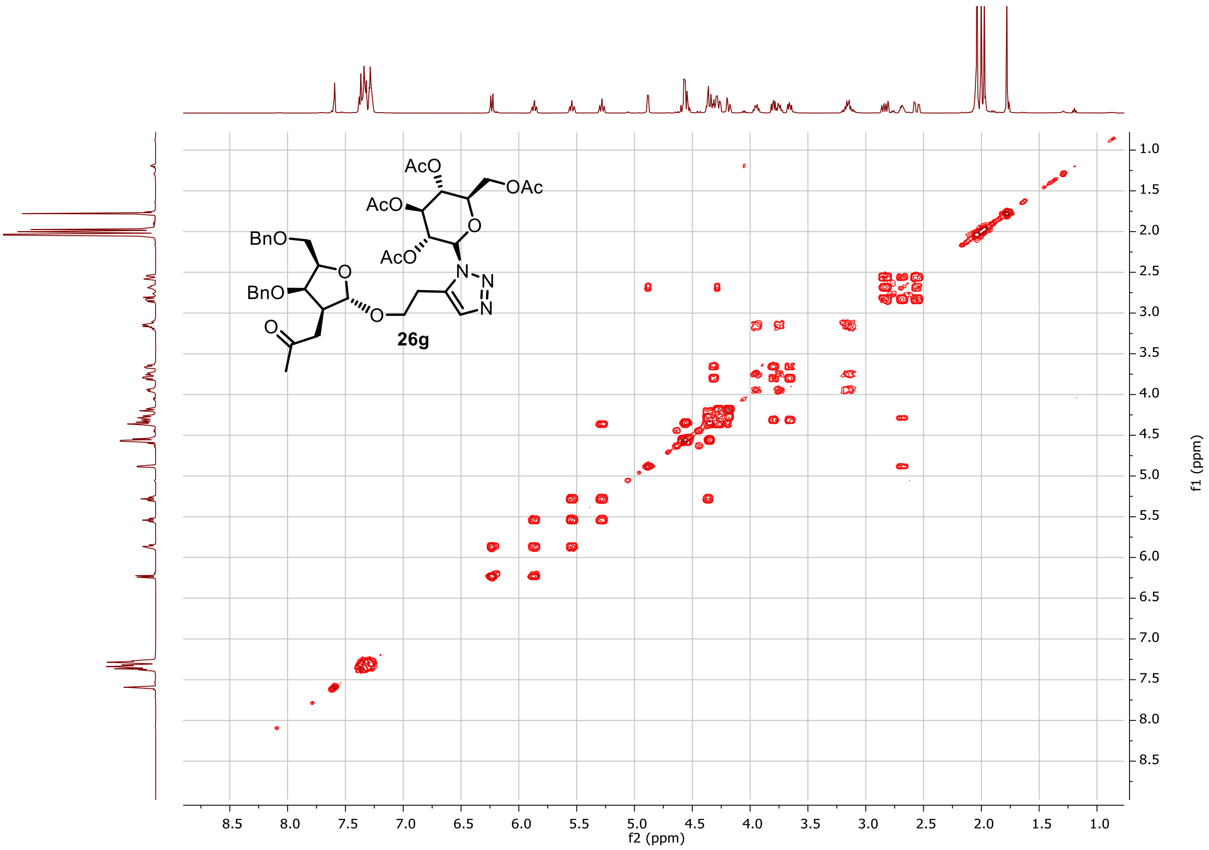

Supplementary Figure 432. COSY spectra for **26g**

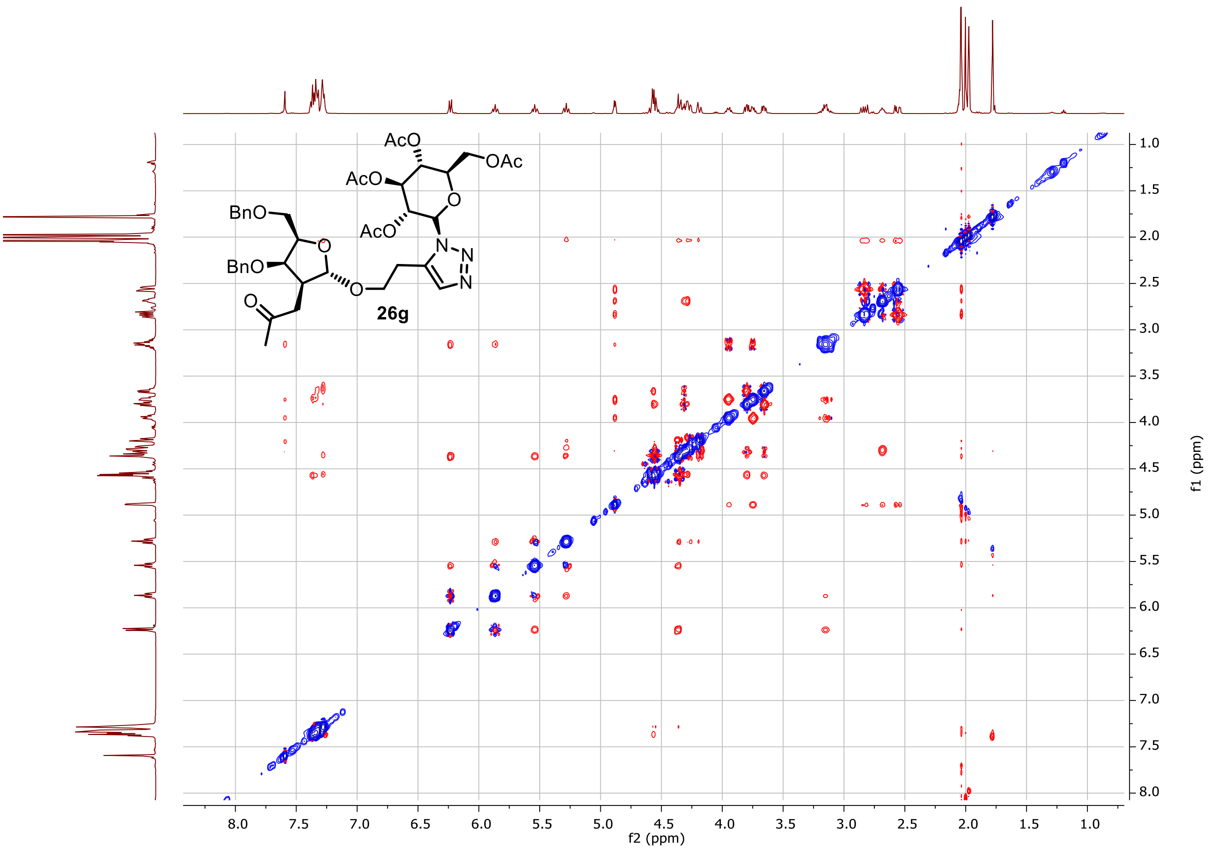

Supplementary Figure 433. NOESY spectra for **26g**

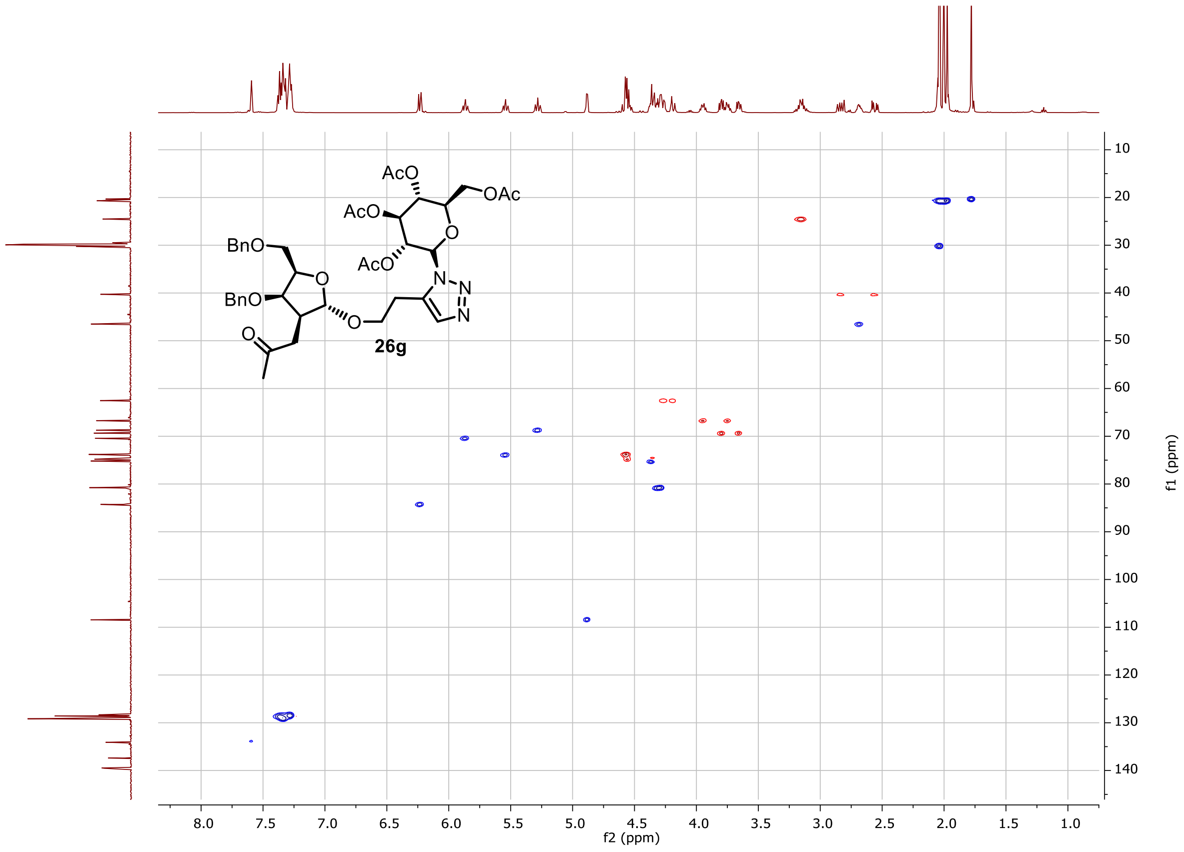

Supplementary Figure 434. HSQC spectra for **26g**

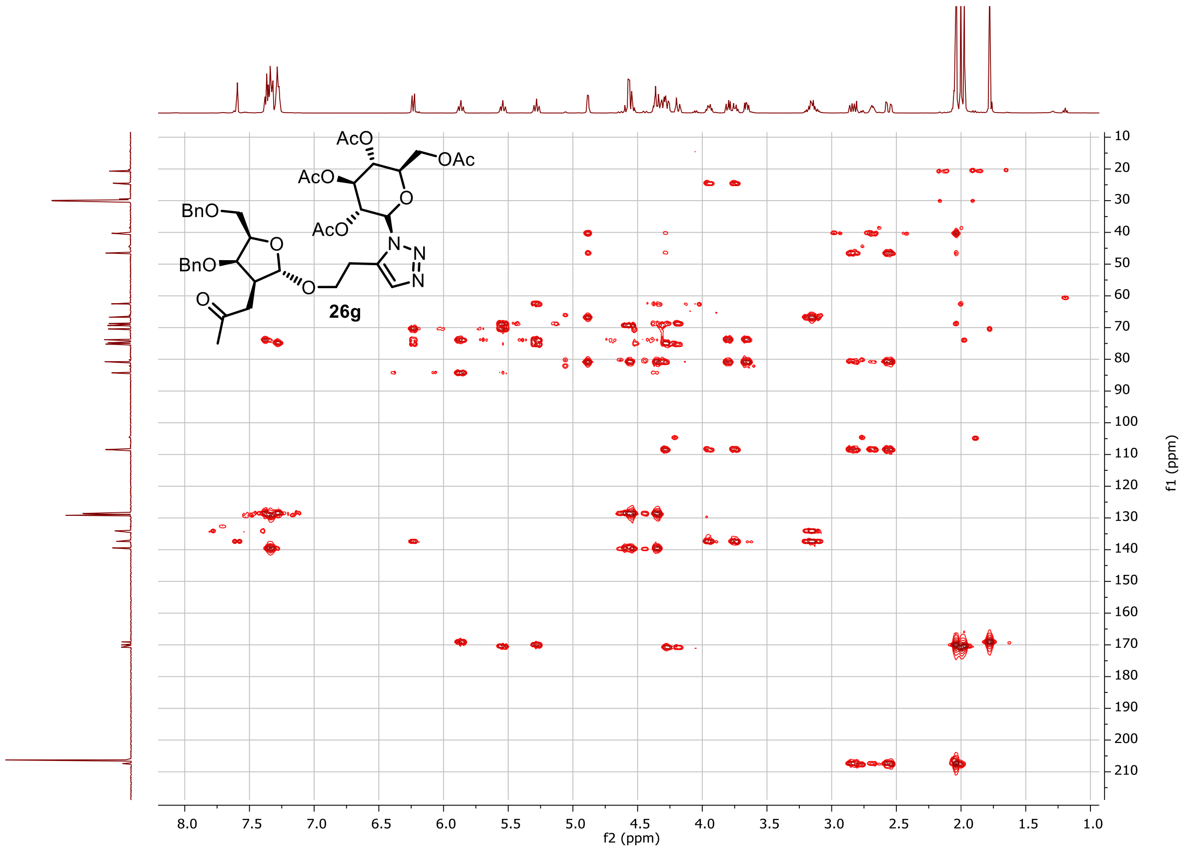

Supplementary Figure 435. HMBC spectra for **26g**

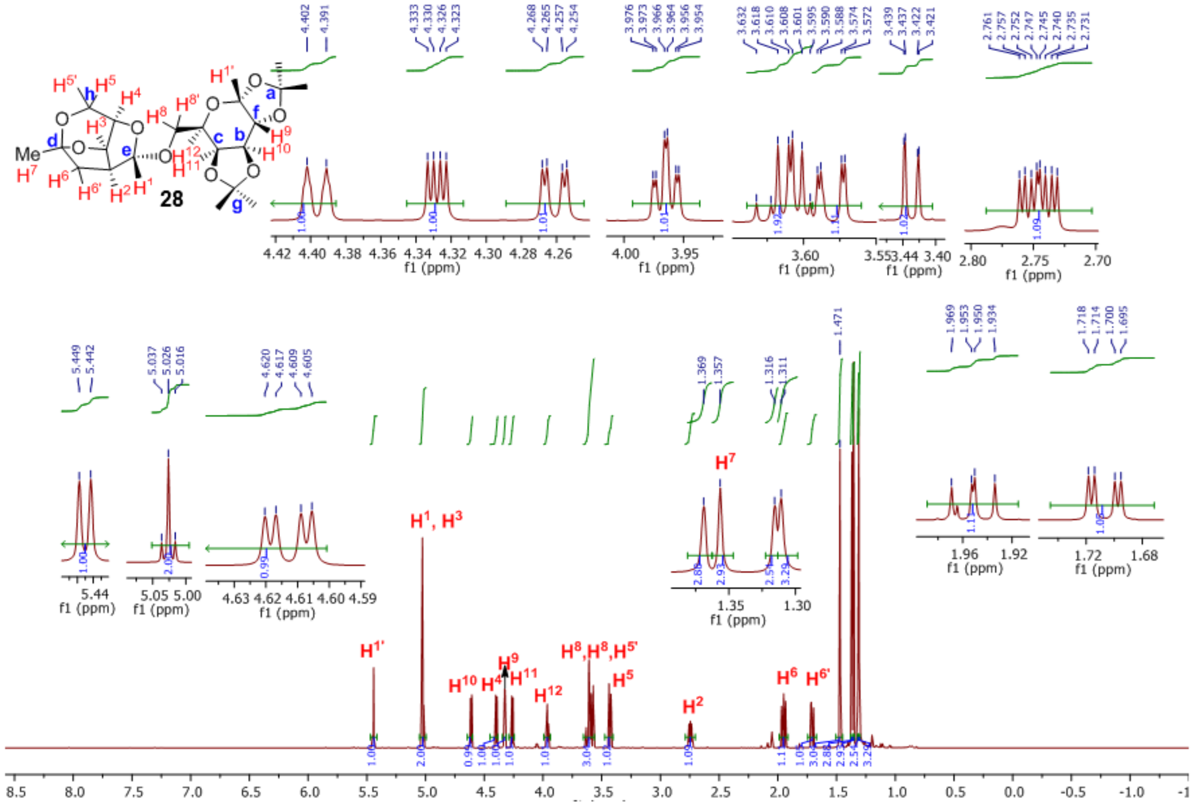

Supplementary Figure 436. ^{1}H spectra for **28**

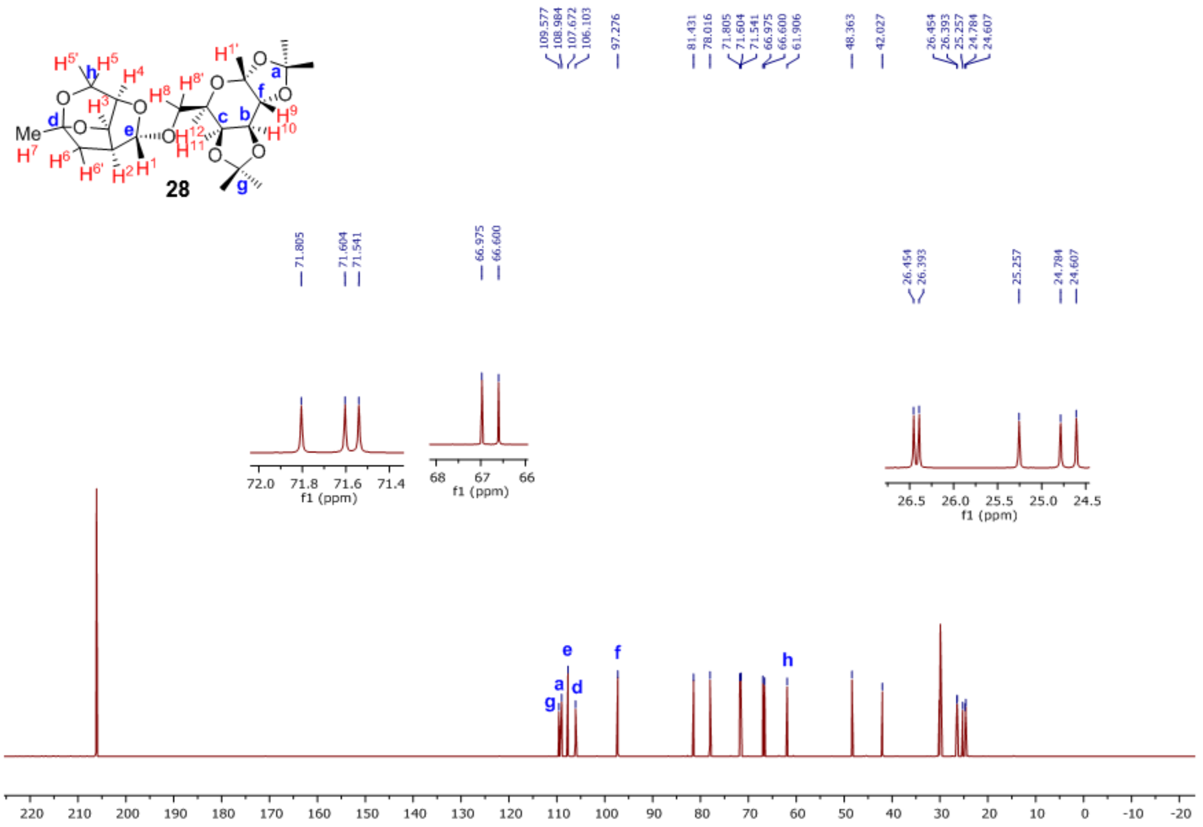

Supplementary Figure 437. ^{13}C spectra for **28**

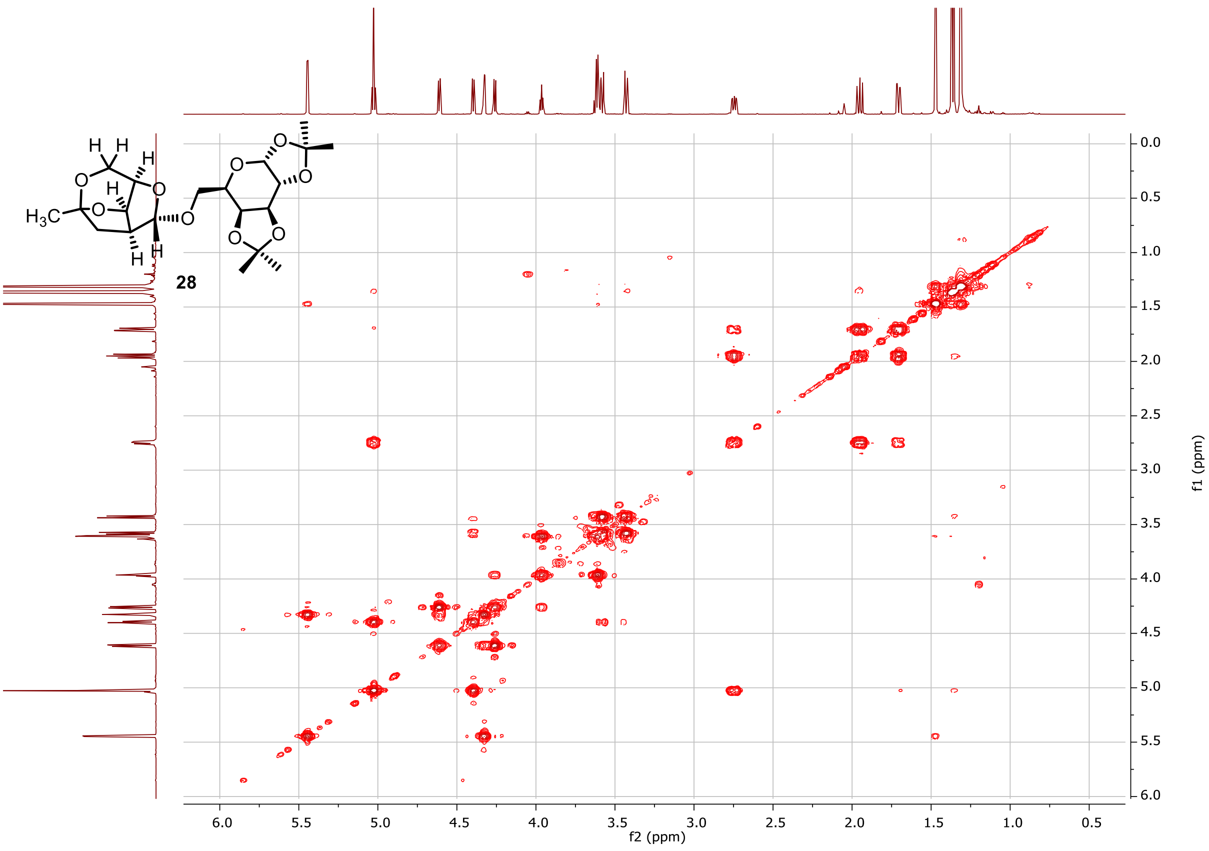

Supplementary Figure 438. COSY spectra for **28**

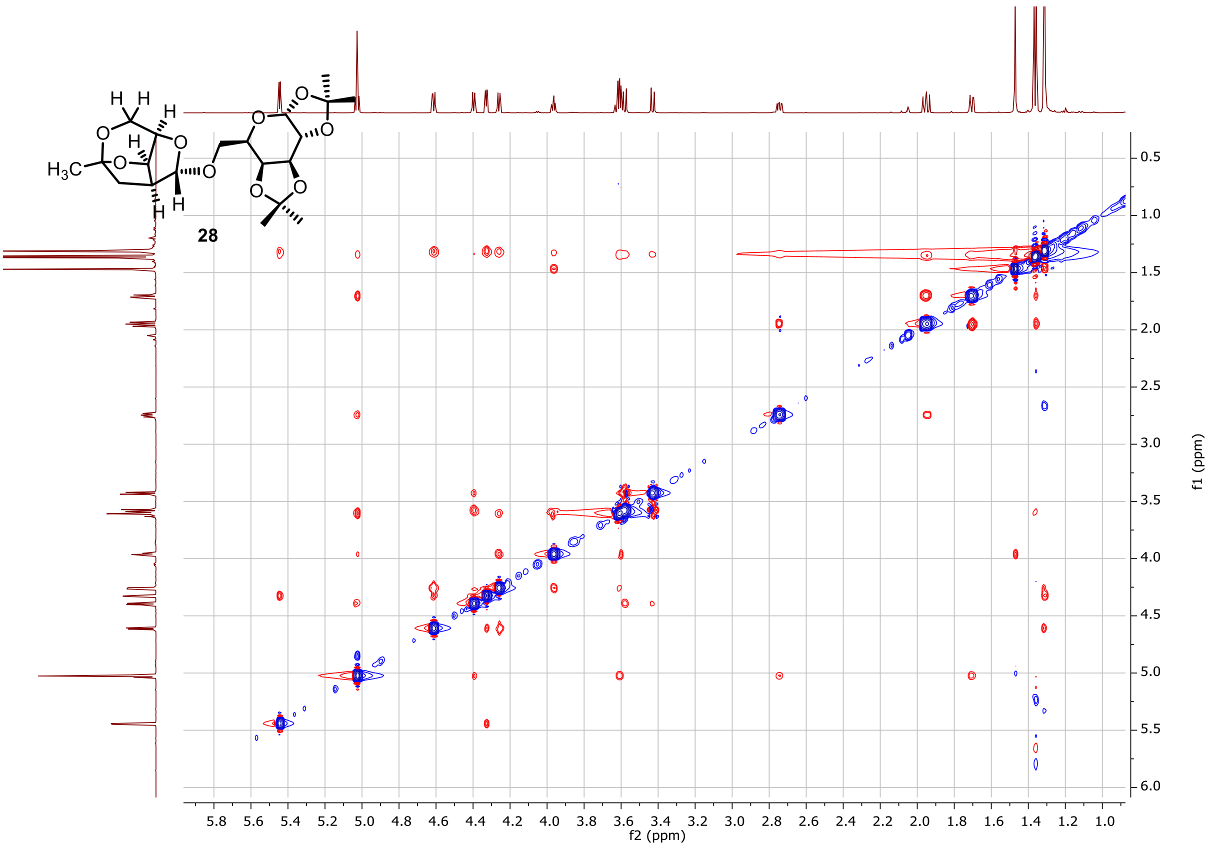

Supplementary Figure 439. NOESY spectra for **28**

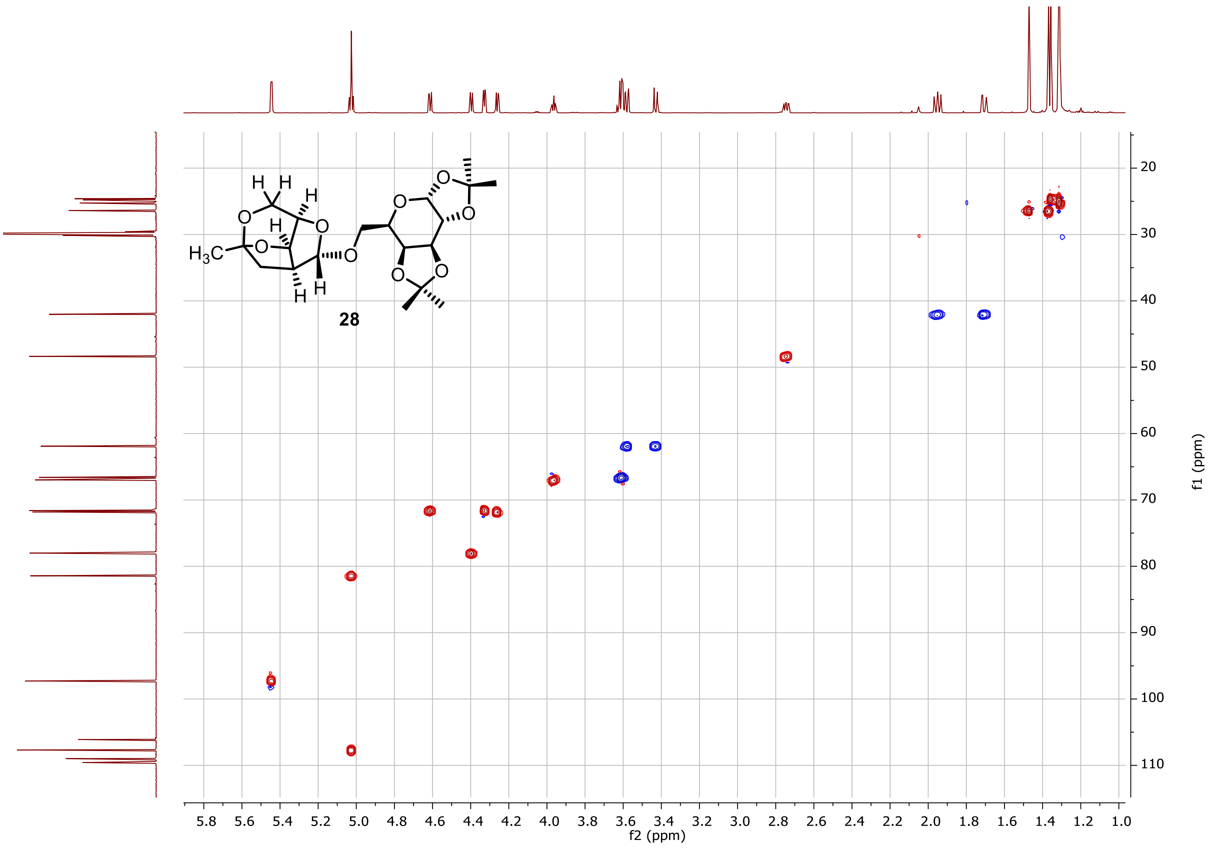

Supplementary Figure 440. HSQC spectra for **28**

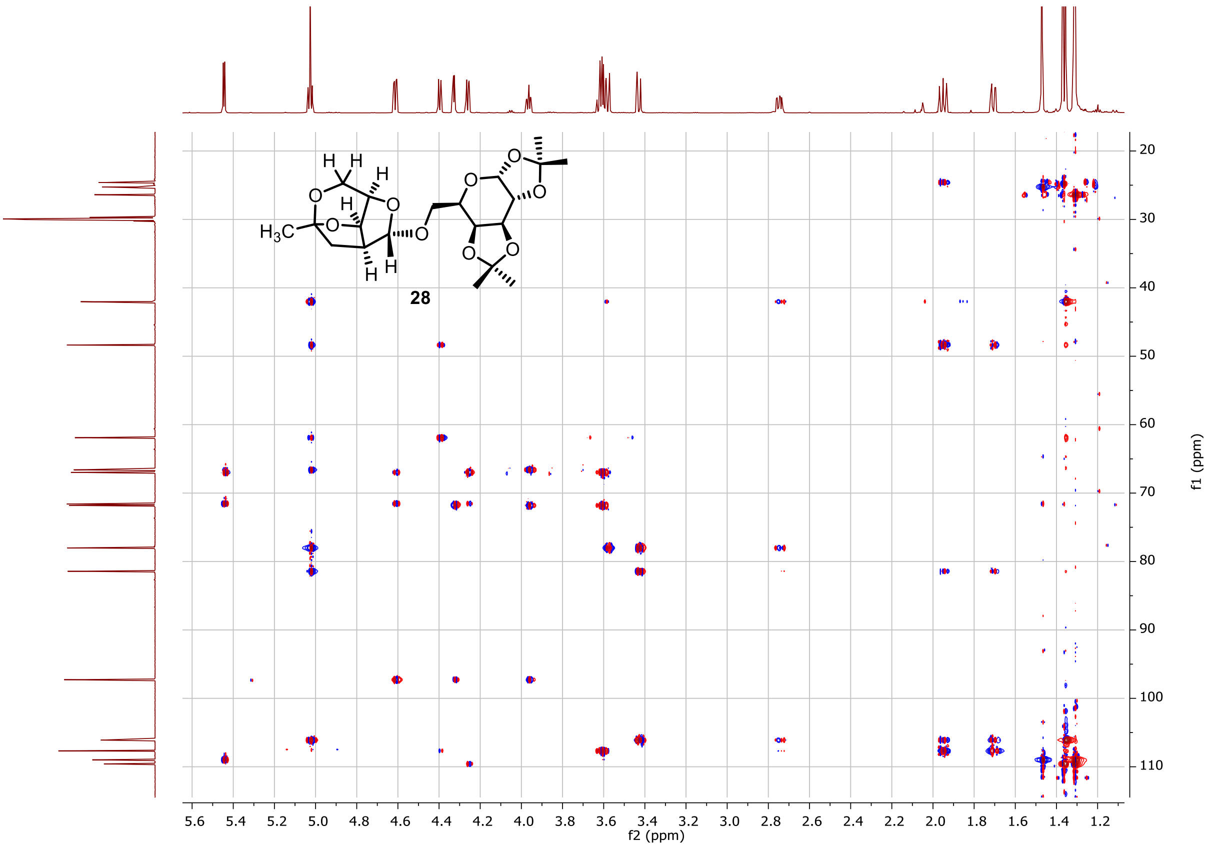

Supplementary Figure 441. HMBC spectra for **28**

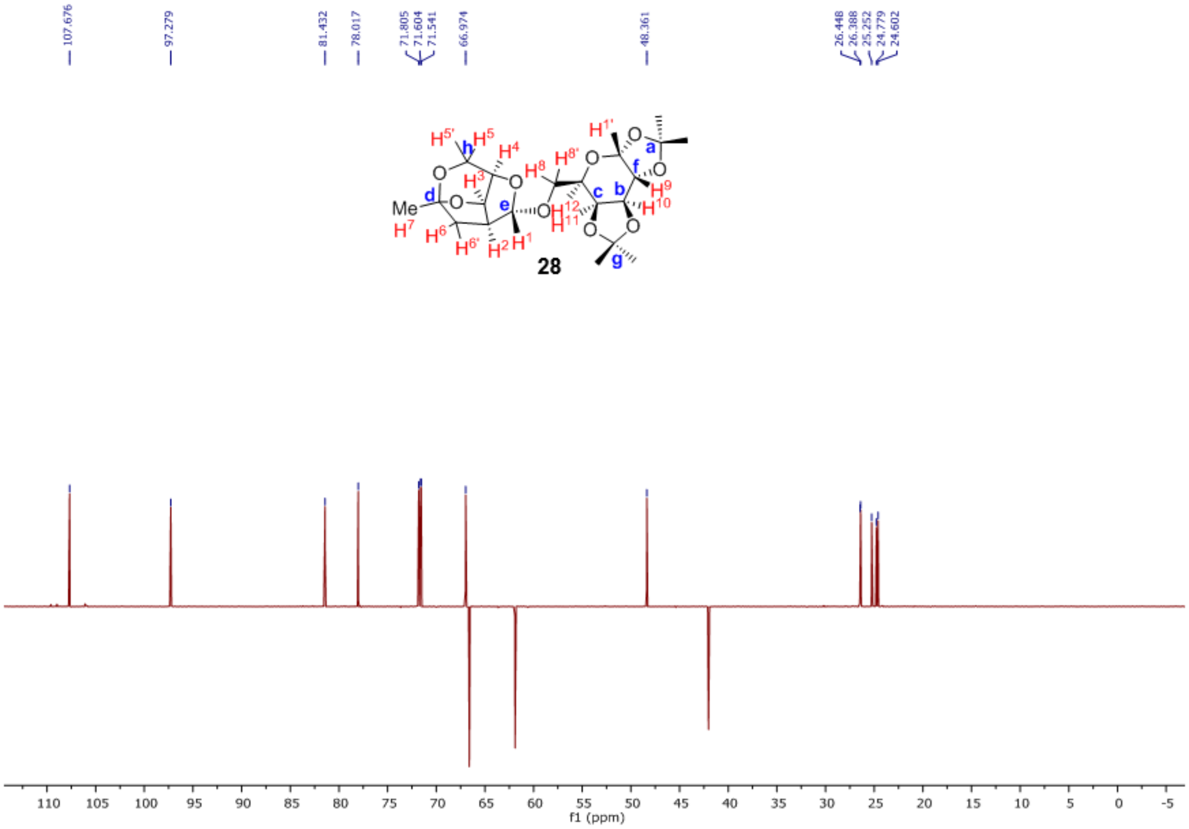

Supplementary Figure 442. DEPT spectra for **28**

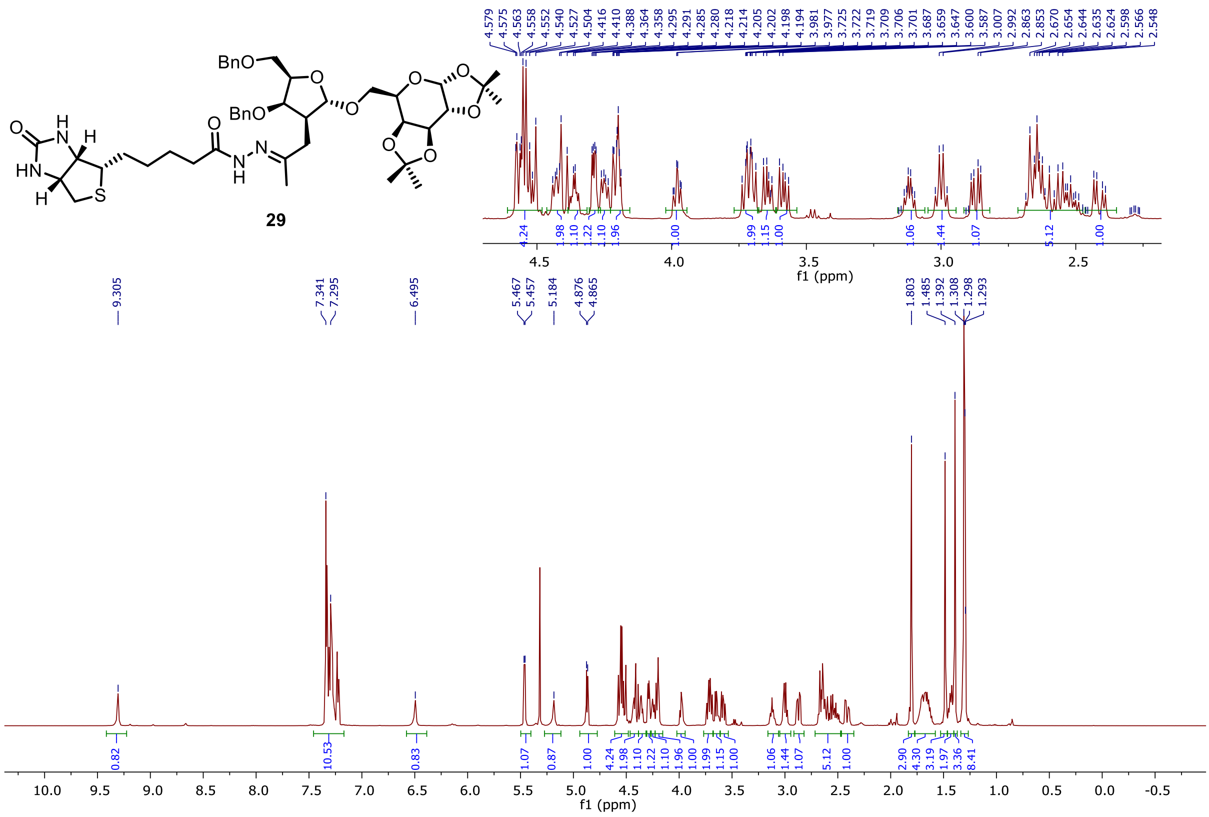

Supplementary Figure 443. ^{1}H spectra for **29**

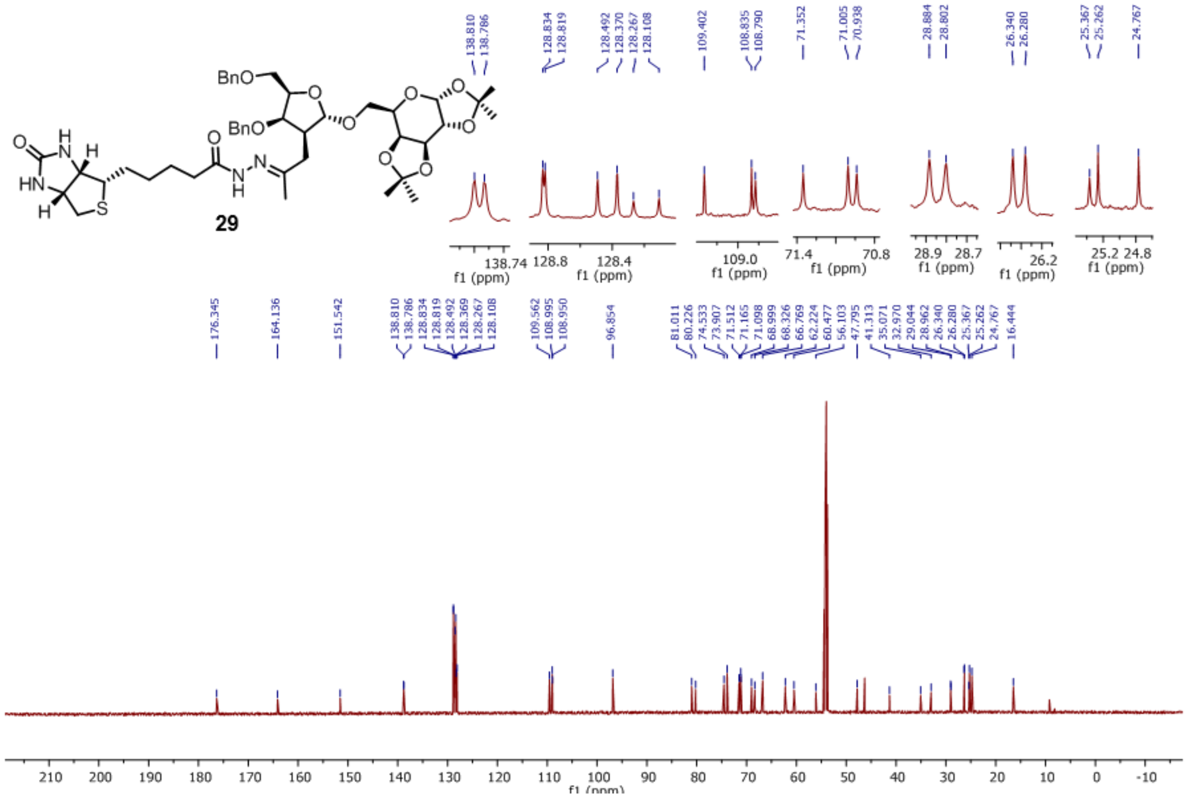

Supplementary Figure 444. ^{13}C spectra for **29**

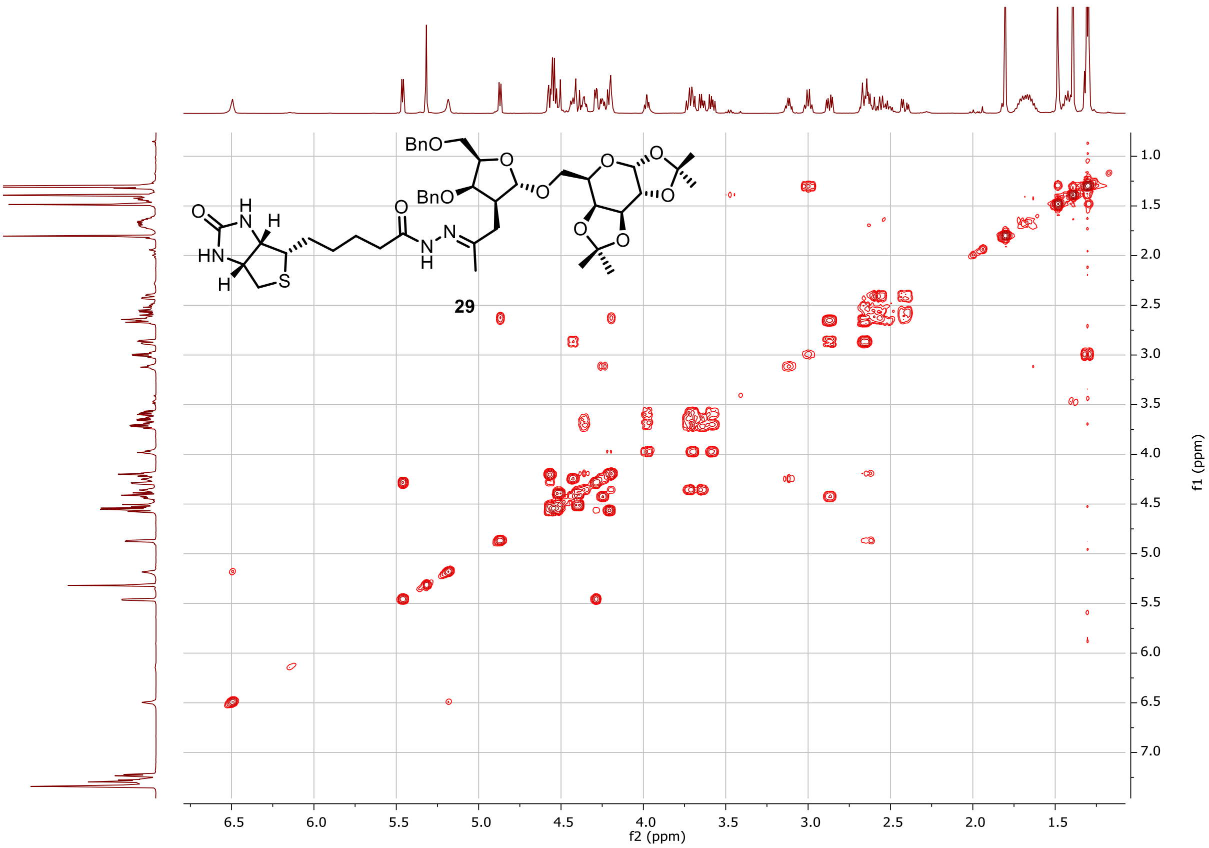

Supplementary Figure 445. COSY spectra for **29**

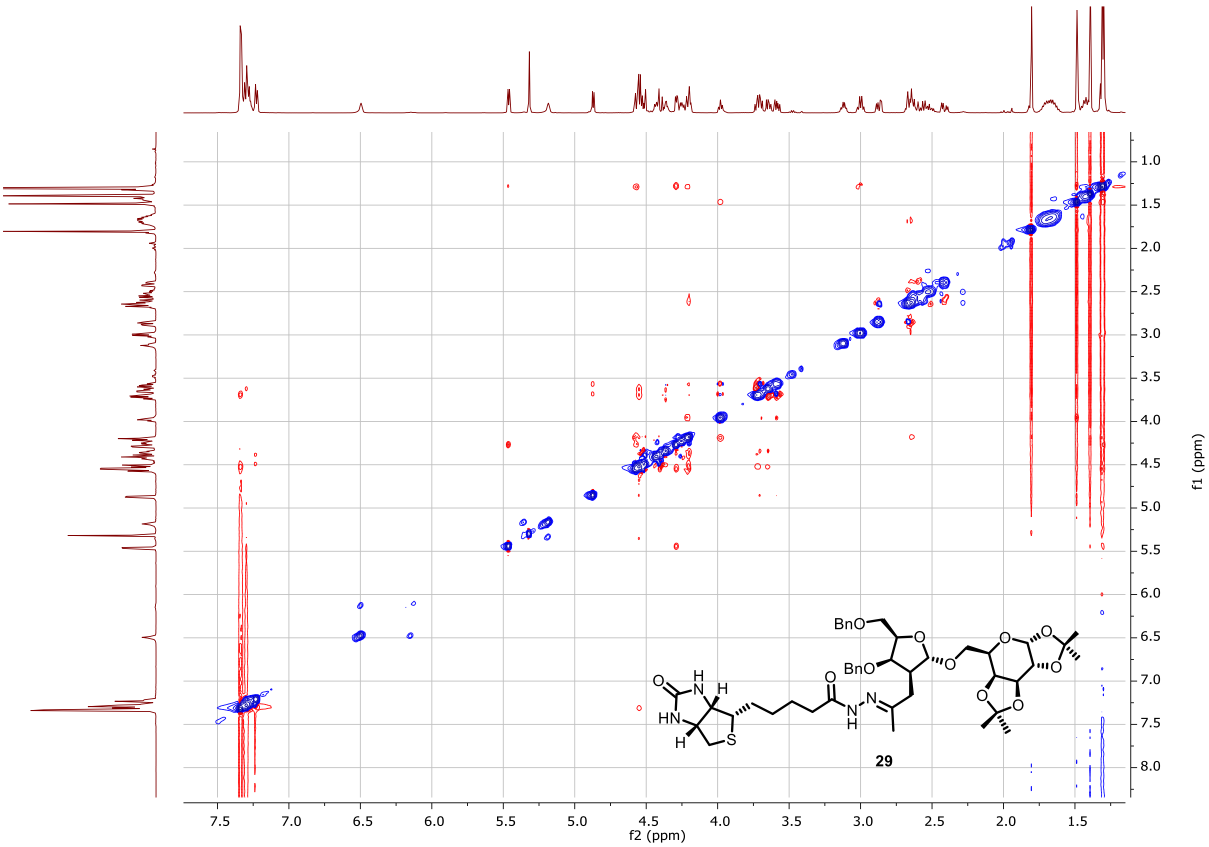

Supplementary Figure 446. NOESY spectra for **29**

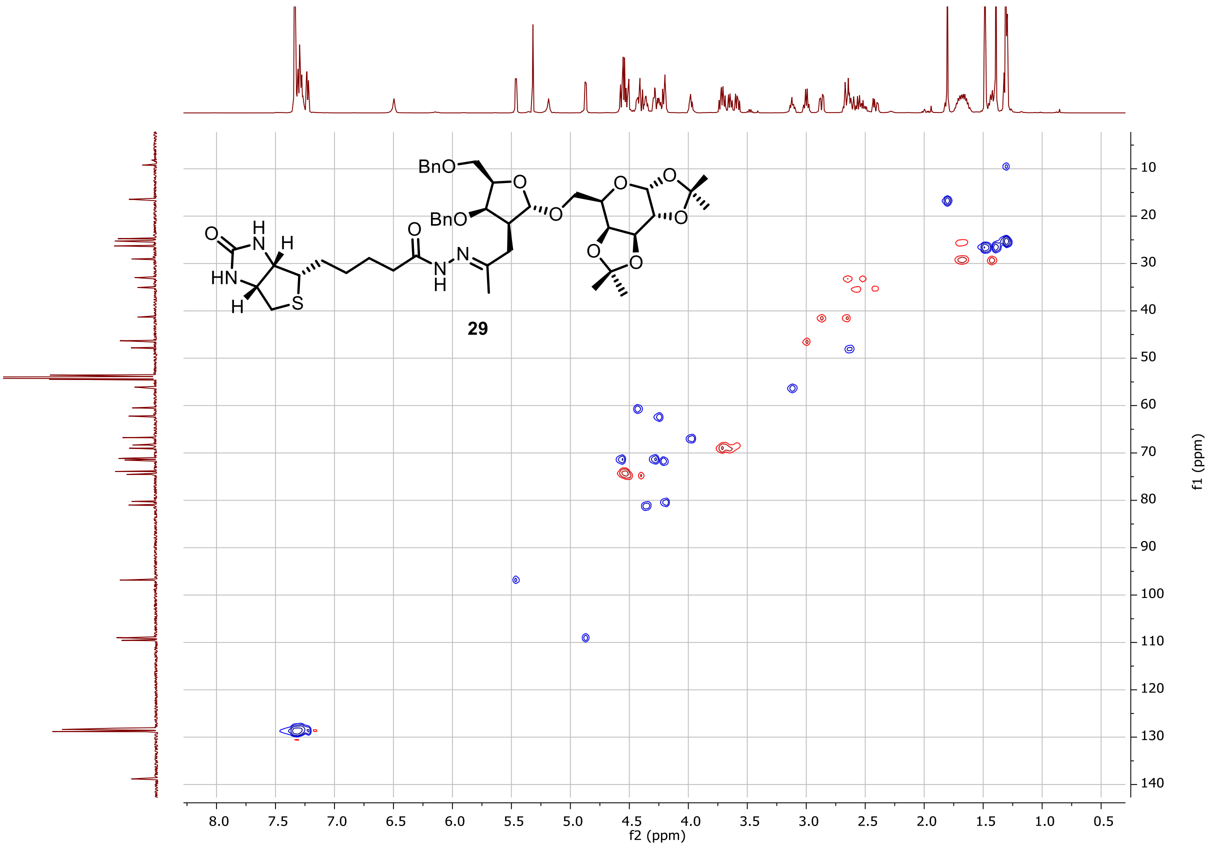

Supplementary Figure 447. HSQC spectra for **29**

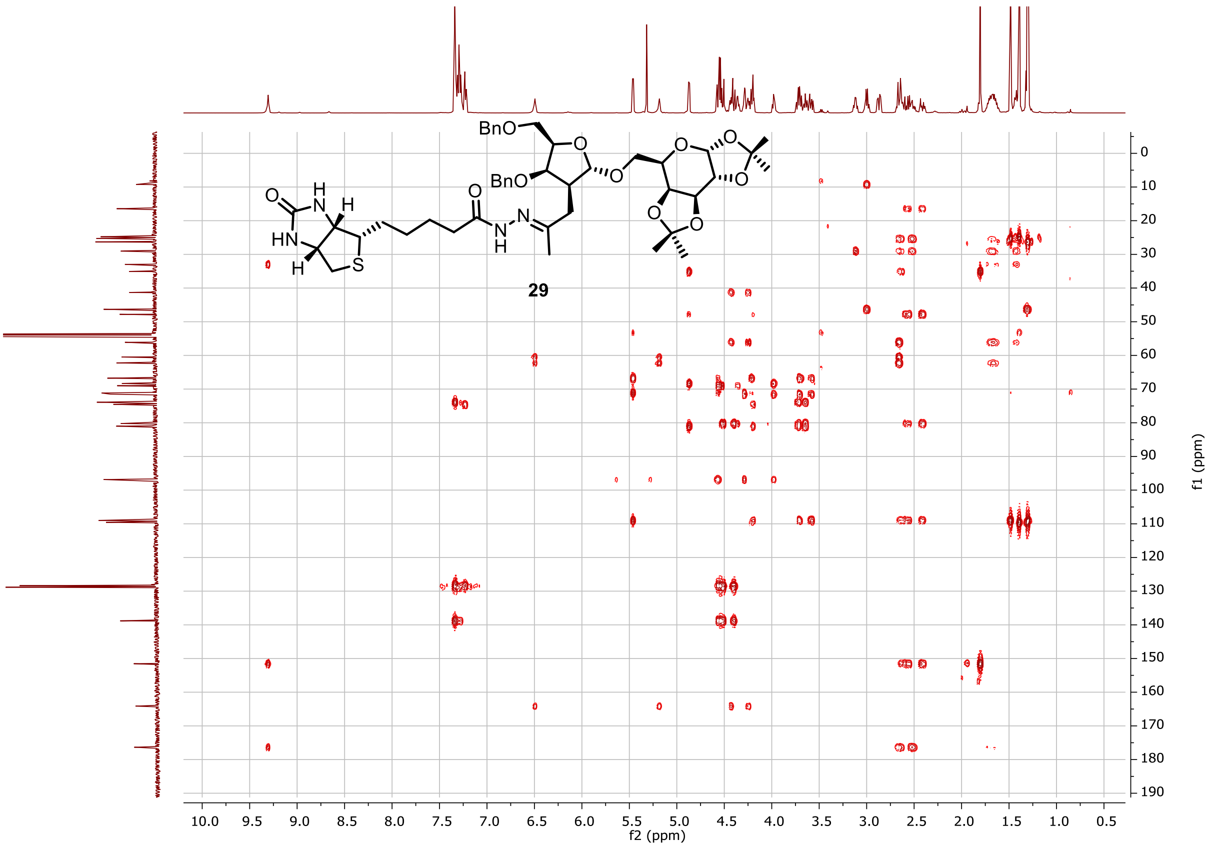

Supplementary Figure 448. HMBC spectra for **29**

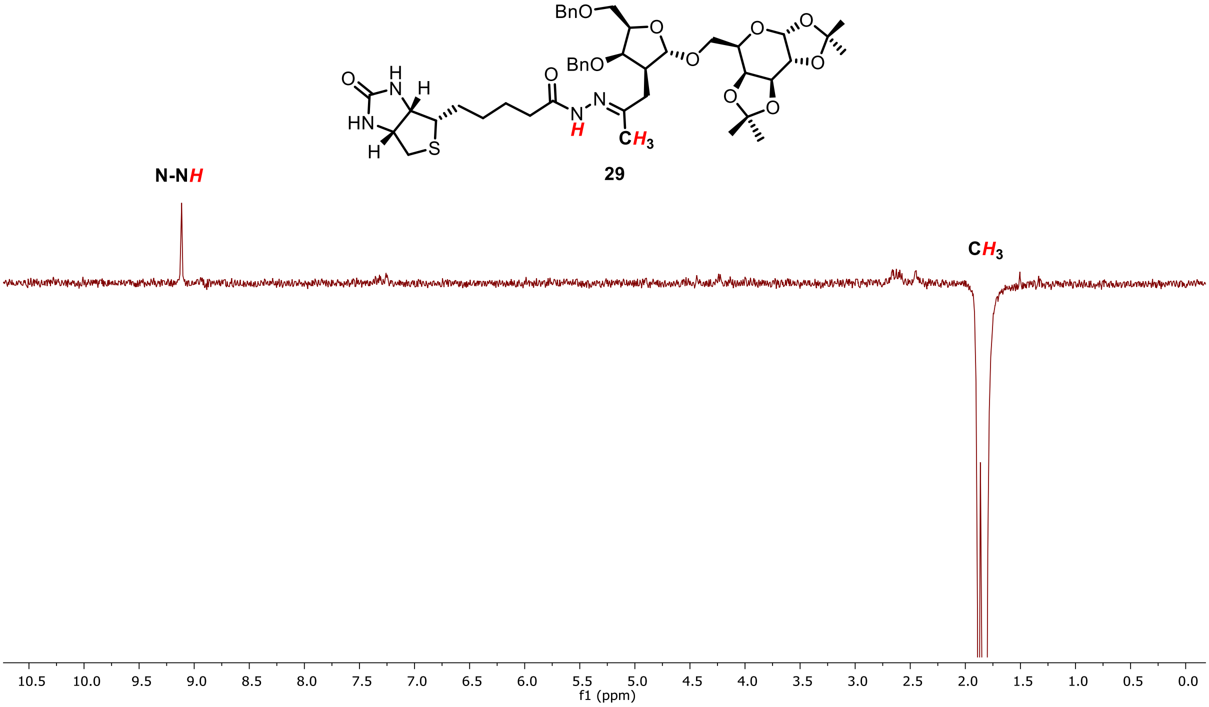

Supplementary Figure 449. 1D NOE spectra for **29**

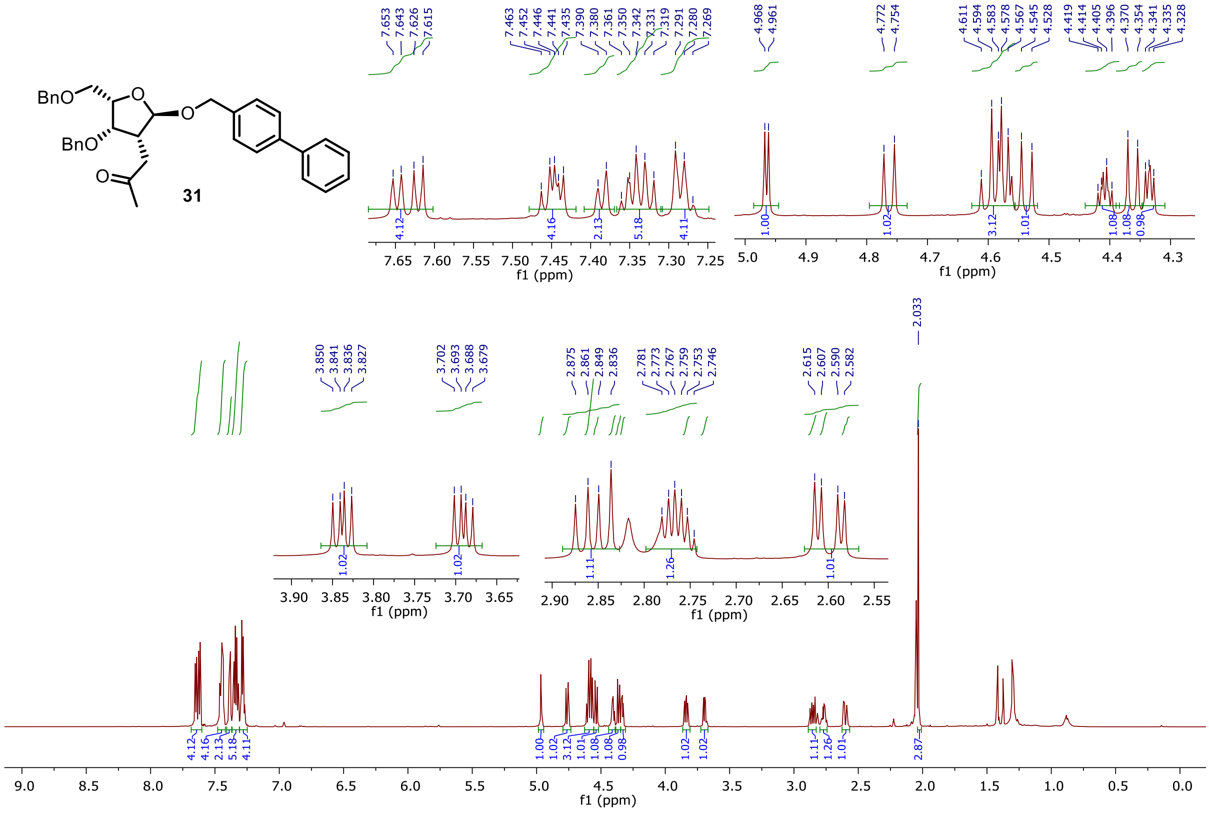

Supplementary Figure 450. ^{1}H spectra for **31**

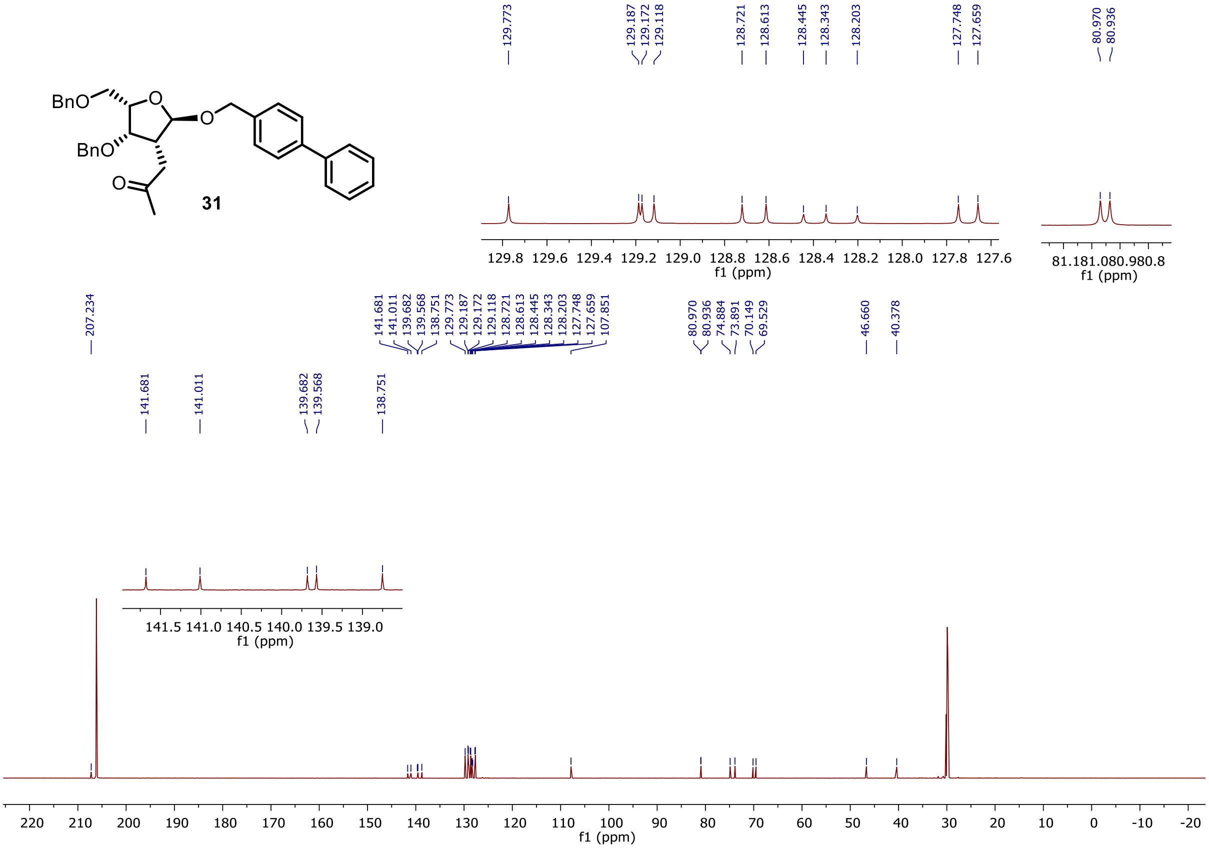

Supplementary Figure 451. ^{13}C spectra for **31**

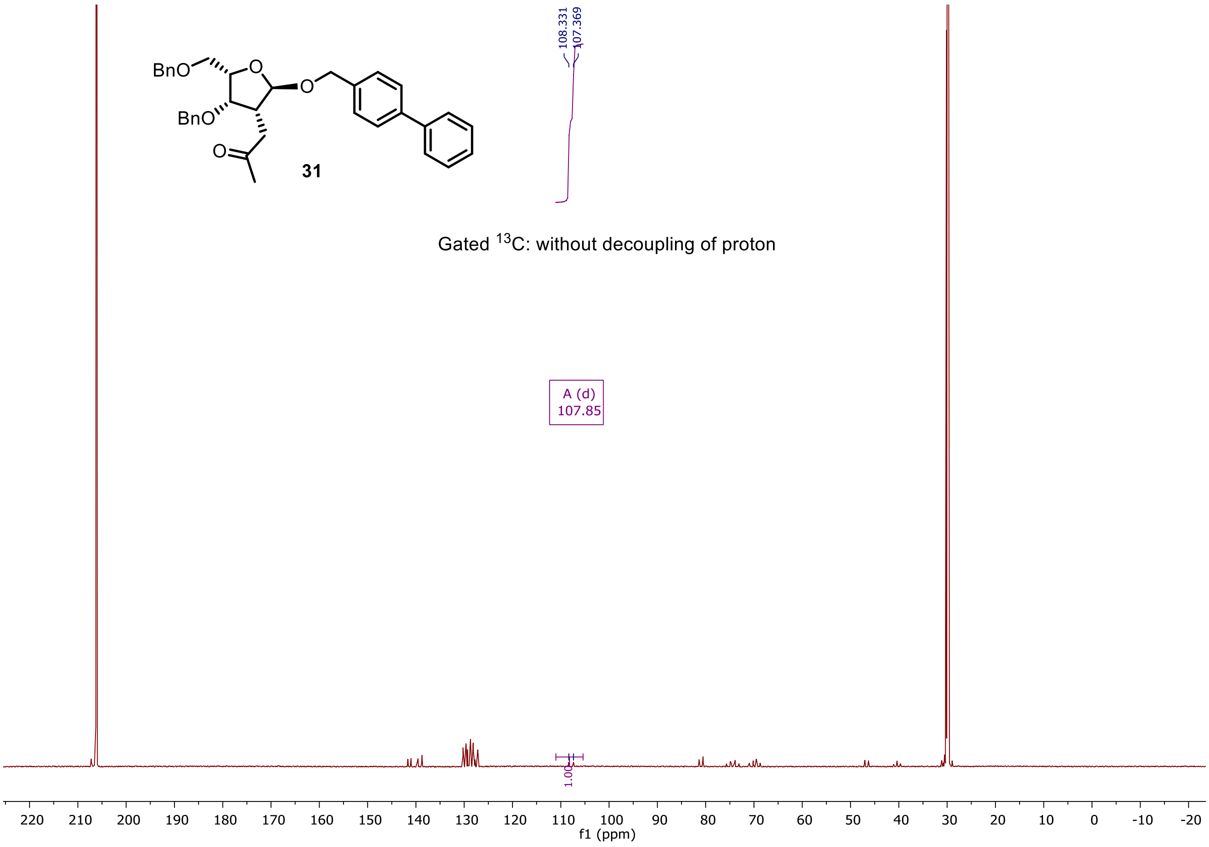

Supplementary Figure 452. Gated ^{13}C (with coupling of proton) spectra for **31**

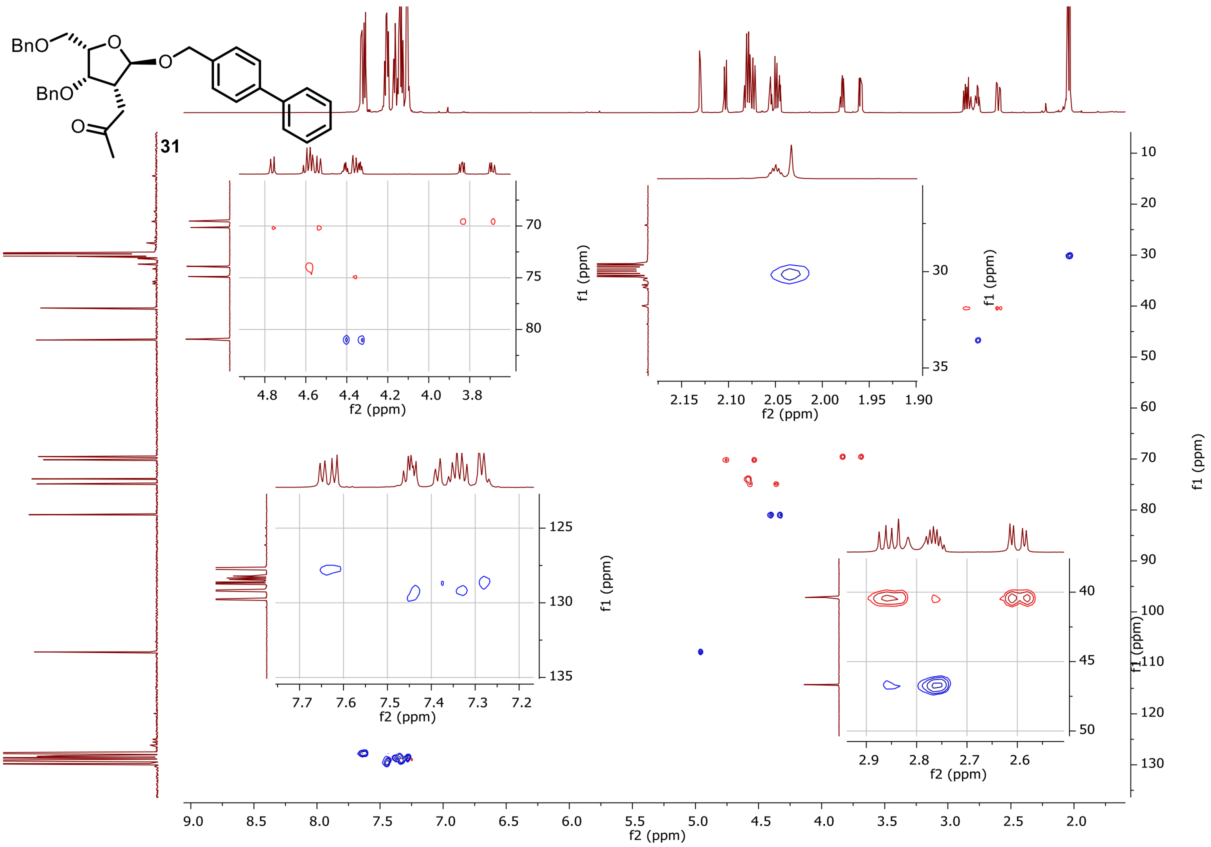

Supplementary Figure 453. HSQC spectra for **31**

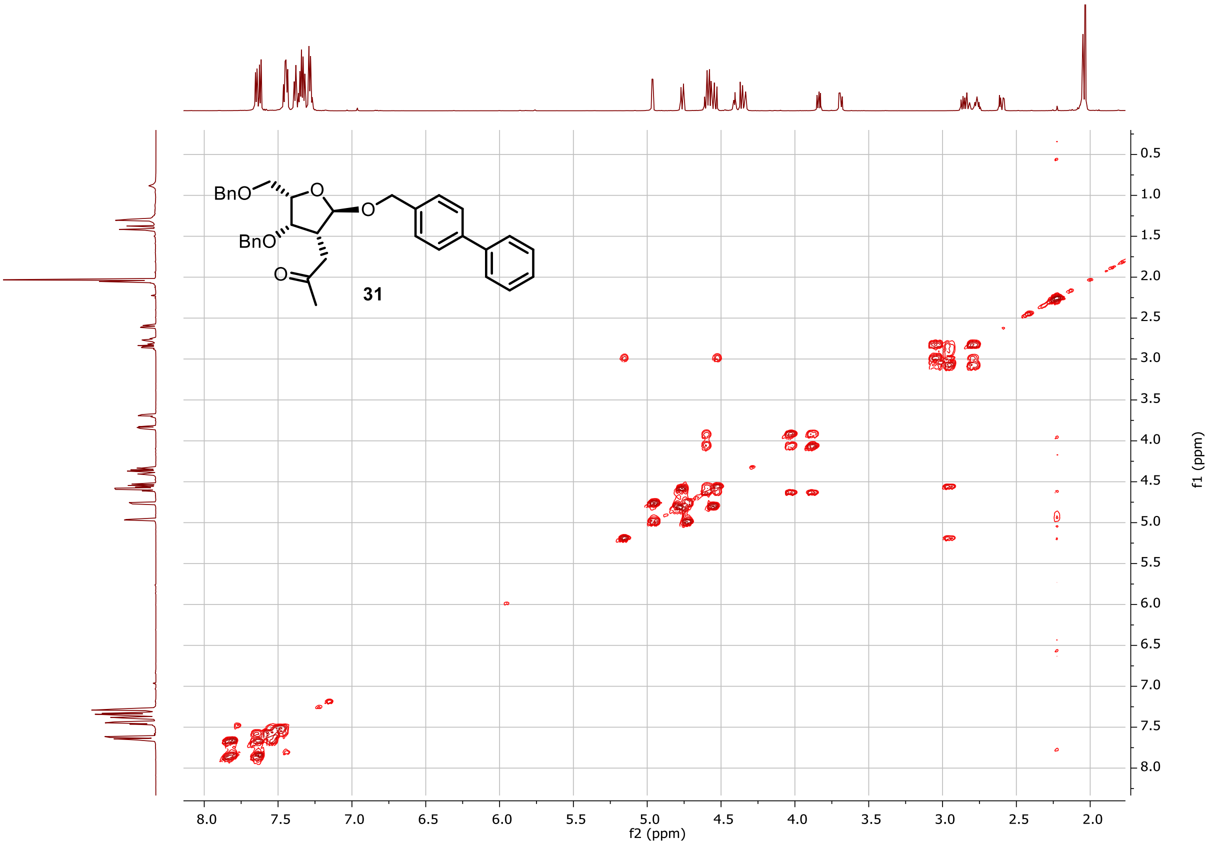

Supplementary Figure 454. COSY spectra for **31**

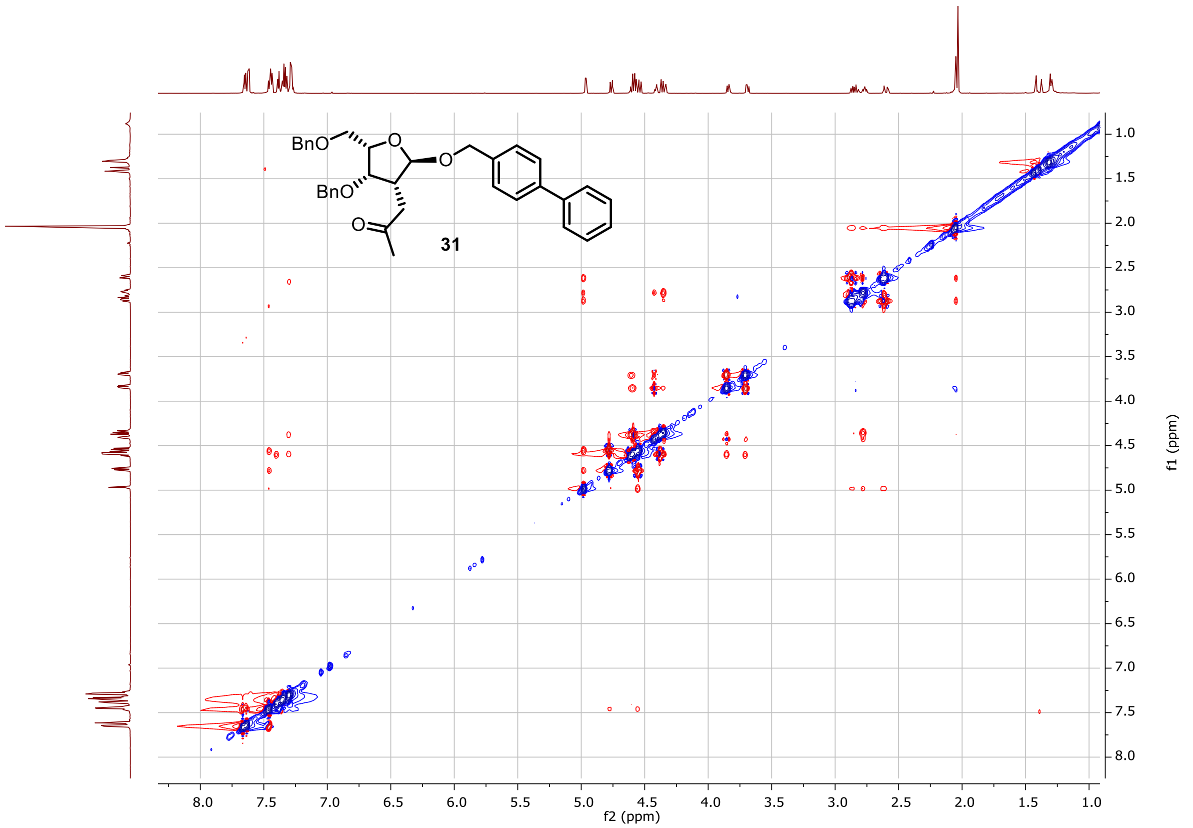

Supplementary Figure 455. NOESY spectra for **31**

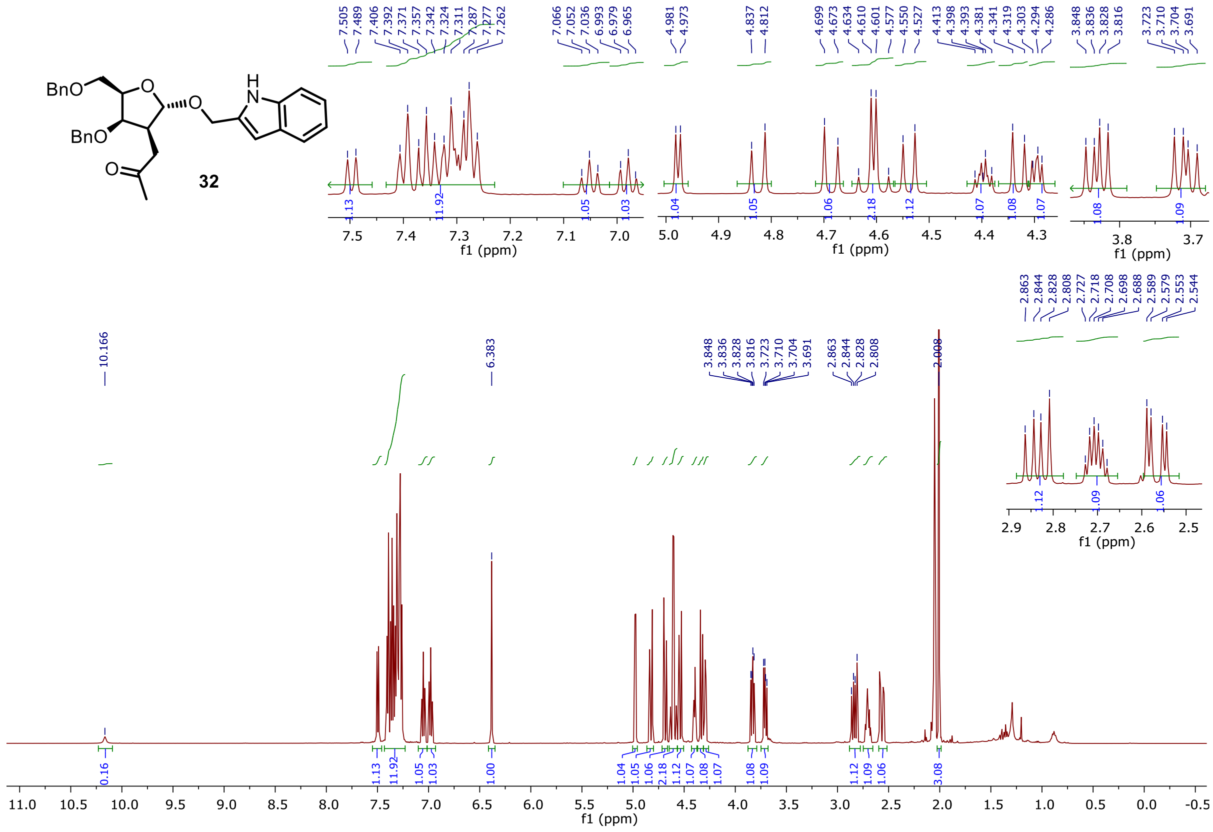

Supplementary Figure 456. ^{1}H spectra for **32**

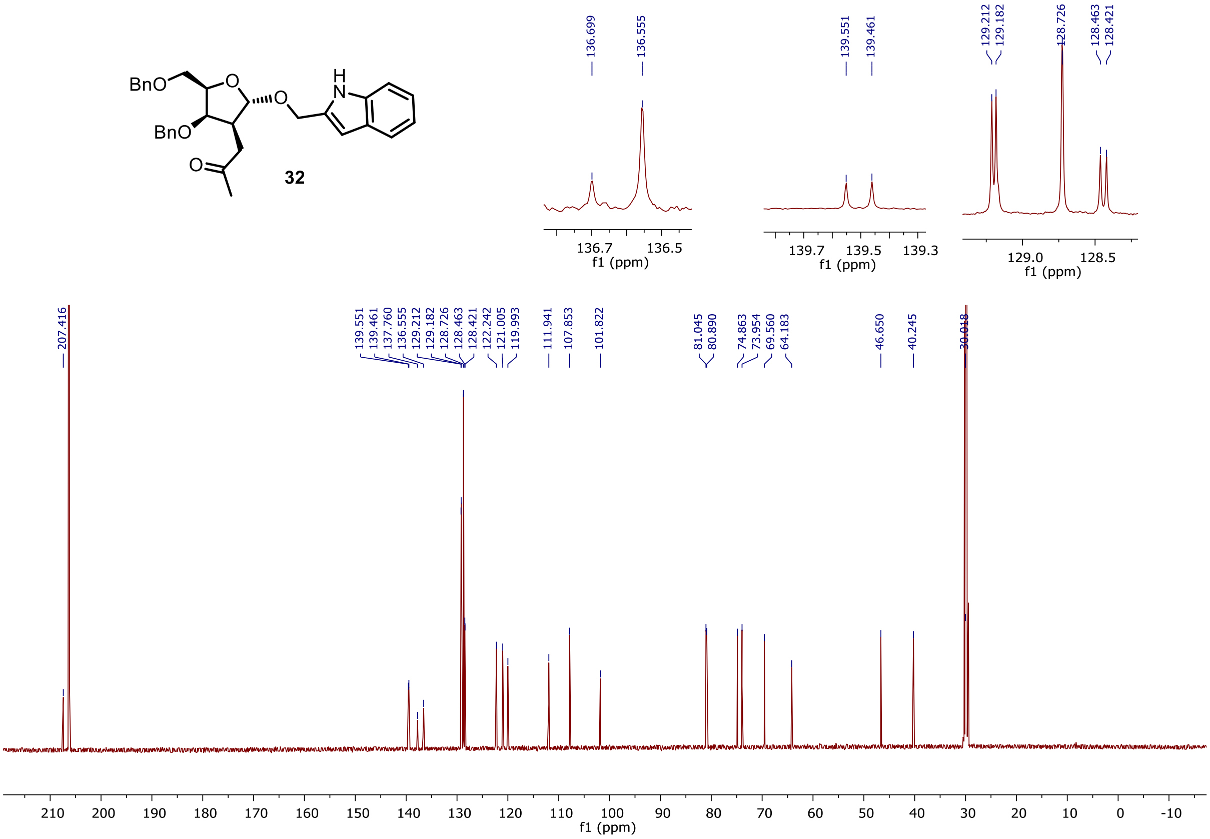

Supplementary Figure 457. ^{13}C spectra for **32**

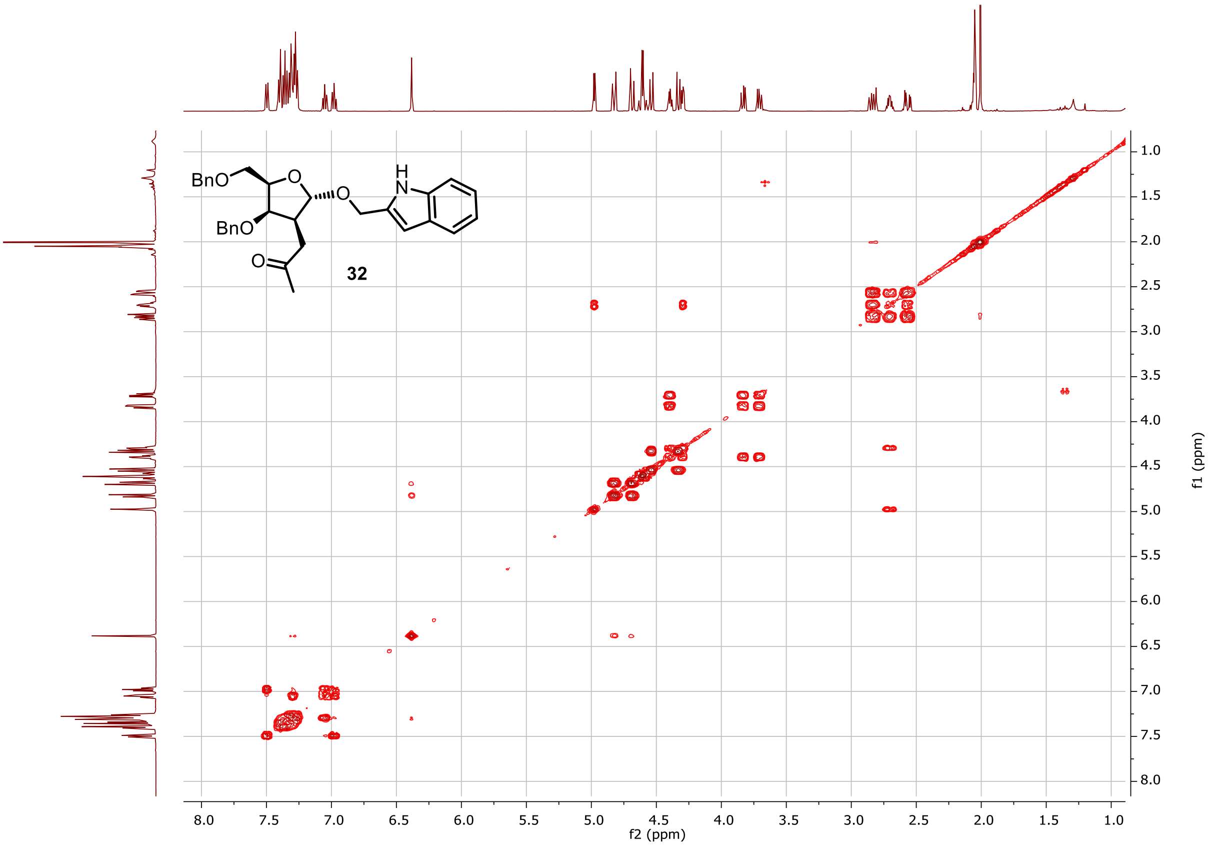

Supplementary Figure 458. COSY spectra for **32**

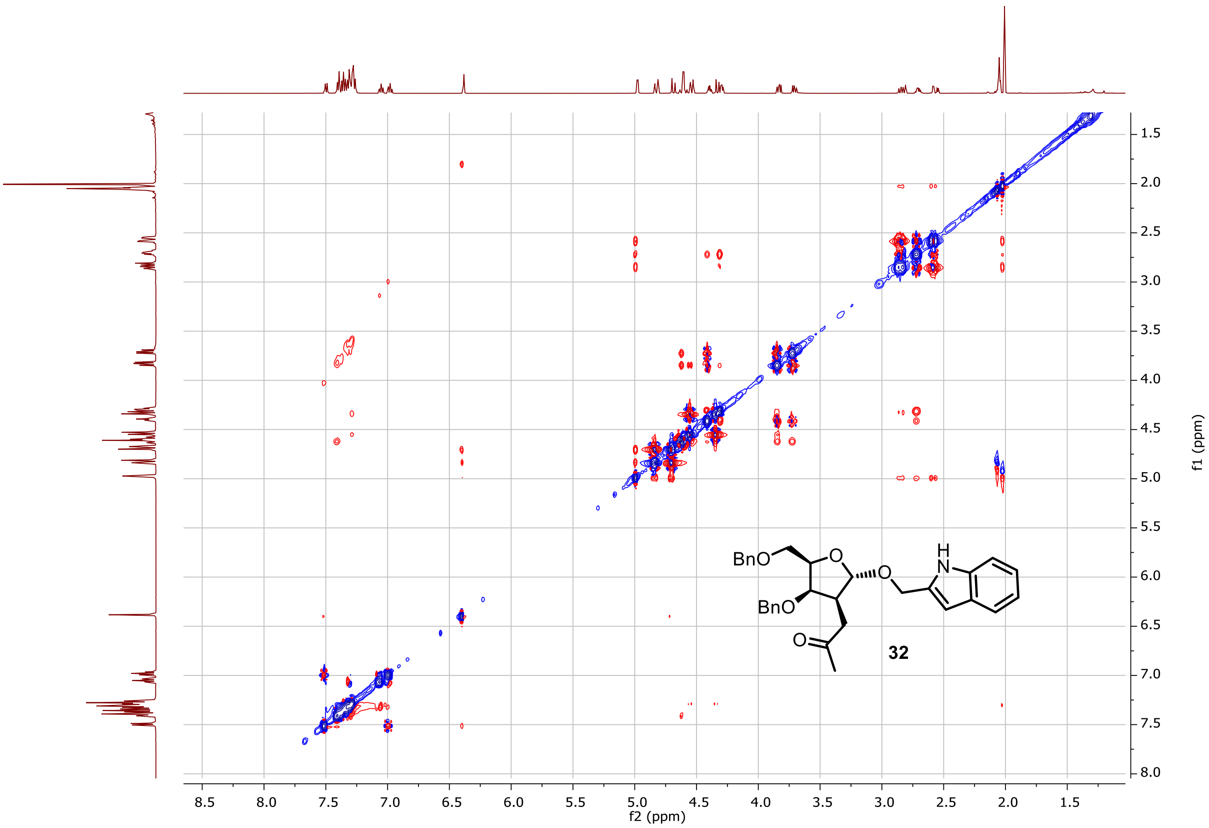

Supplementary Figure 459. NOESY spectra for **32**

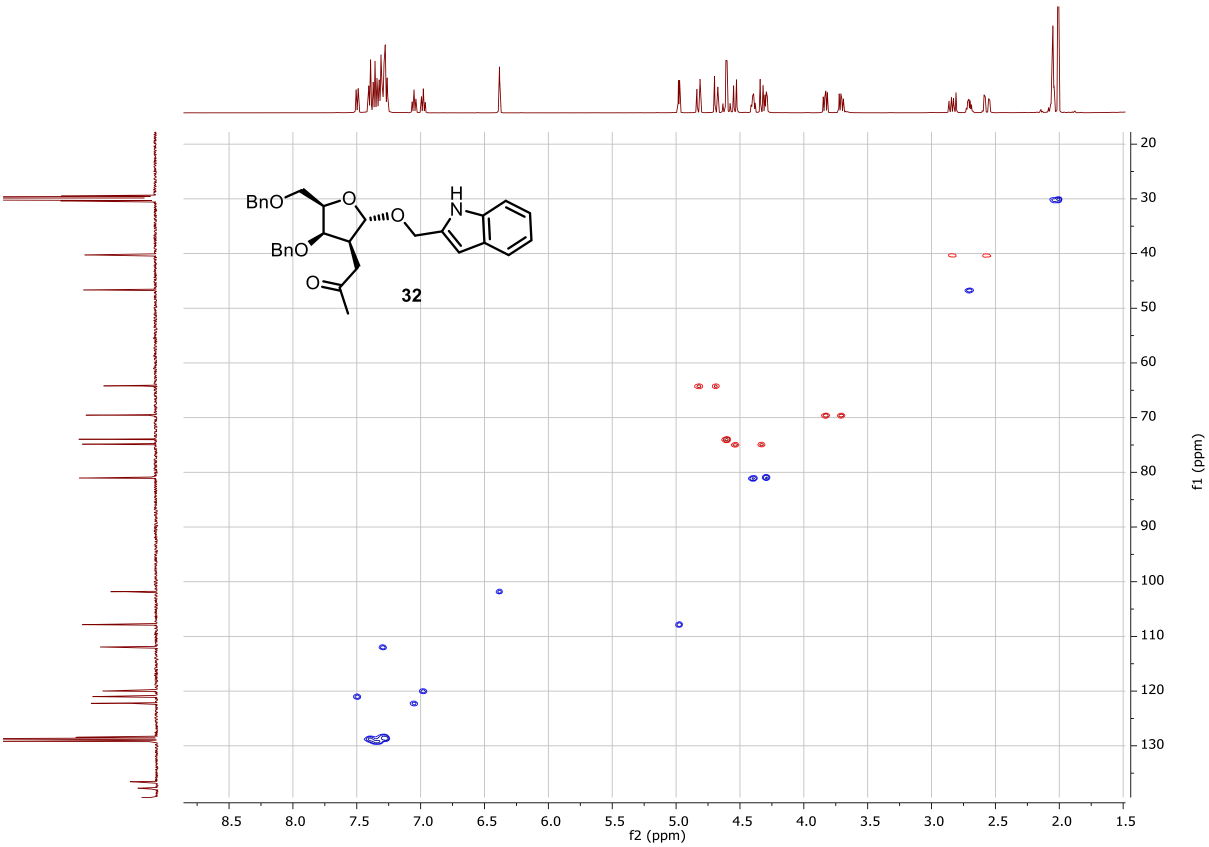

Supplementary Figure 460. HSQC spectra for **32**

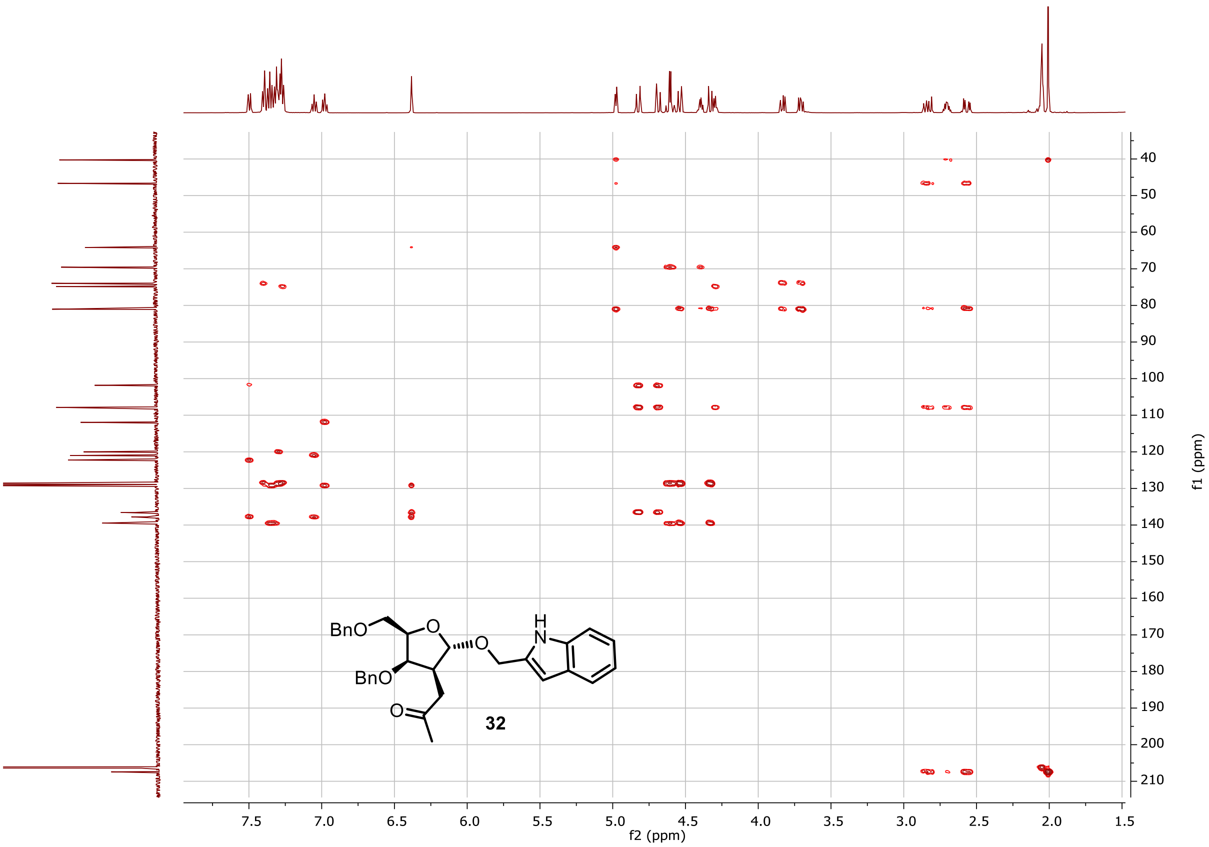

Supplementary Figure 461. HMBC spectra for **32**

Supplementary Methods

General Information: Unless otherwise stated, all reactions were set up under inert atmosphere (argon) utilizing glassware that were oven dried and cooled under argon purging. Silica Gel Flash Column Chromatography was performed on deactivated *Silica gel Merck 60 (particle size 40-63 μm)* (*Triethylamine* (5% v/v) *was used as the deactivating reagent*). Starting materials were purchased directly from commercial suppliers (Sigma Aldrich, Acros, Alfa Aesar, VWR) and used without further purifications unless otherwise stated. All solvents were dried according to standard procedures or brought from commercial suppliers. Reaction solvent (Fluorobenzene) was stored over activated 3Å molecule sieves. Reactions were monitored using thin-layer chromatography (TLC) on *Merck silica gel aluminium plates with F_{254} indicator*. Visualization of the developed plates was performed under UV light (254 nm) or $KMnO_4$ stain or H_2SO_4-EtOH (10% H_2SO_4 v/v). Dry loading was performed on Silica gel 9[1] due to observed product decomposition on normal silica gel.

NMR characterization data (^{1}H NMR, ^{13}C NMR and 2D spectra) were collected at 300 K on a *Bruker DRX400 (400 MHz)*, *Bruker DRX500 (500 MHz)*, *INOVA500 (500 MHz)* and *Bruker DRX700 (700 MHz)* using acetone-d_6, CD_2Cl_2 or $CDCl_3$ as solvent. Data for ^{1}H NMR are reported as follows: chemical shift (δ ppm), multiplicity (s = singlet, d = doublet, t = triplet, q = quartet, m = multiplet, br = broad), coupling constant (Hz), integration with the solvent resonance as internal standard (acetone-d_6: δ = 2.05 ppm for ^{1}H, δ = 29.92 ppm for ^{13}C; CD_2Cl_2: δ = 5.32 ppm for ^{1}H, δ = 54.00 ppm for ^{13}C; $CDCl_3$: δ = 7.26 ppm for ^{1}H, δ = 77.16 ppm for ^{13}C).

High resolution mass spectra were recorded on a *LTQ Orbitrap* mass spectrometer coupled to an *Accela HPLC*-System (HPLC column: *Hypersyl GOLD*, 50 mm x 1 mm, particle size 1.9 μm, ionization method: electron spray ionization). Optical rotations were measured in a *Schmidt + Haensch Polartronic HH8* polarimeter equipped with a sodium lamp source (589 nm), and are reported as follows: $[\alpha]_D^{T\,°C}$ (*c* = g/100 mL, solvent). Melting point ranges were taken from solids which were obtained from the solvents as indicated. They were determined on a BÜCHI Melting Point B-540 Apparatus. The microwave reaction was conducted on the Discover SP-Microwave Synthesizer and 10 mL tube with a proper cap was used.

The ratio of anomers was determined by ^{1}H-NMR and HSQC analysis of the crude reaction mixture via integration of characteristic signals of the anomeric proton in the ^{1}H NMR spectra. Chemical yields refer to isolated substances after flash column chromatography combined yield of both anomers reported. NMR yields were determined using dibromomethane or 1,3,5-trimethoxybenzene as internal standard.

General method for optimization for furanosylation (see supplementary table 1). A mixture of catalyst, cyclopropanated sugar **19a** and glycosyl acceptor **21a** was sealed in a dry tube under argon. The mixture was stirred at room temperature or in a preheated oil bath. After cooling to room temperature, the reaction mixture was analyzed by crude ^{1}H NMR with CH_2Br_2 as the internal standard.

General method for optimization for furanosylation (see supplementary table 2). A mixture of catalyst, cyclopropanated sugar **22a** and glycosyl acceptor allyl alcohol was sealed in a dry tube under argon. The mixture was stirred at room temperature or in a preheated oil bath. After cooling to room temperature, the reaction mixture was analyzed by crude ^{1}H NMR with CH_2Br_2 as the internal standard.

General procedure for thiourea catalyzed strain release glycosylation of furanosides.

Method A: An oven dried tube with a stirrer bar was charged with strained cyclopropanated furanoside **19a**, ***ent*-19a** or **19b** (70.5 mg, 0.2 mmol, 1.0 equiv.), and glycosyl acceptor **21** (0.4 mmol, 2.0 equiv.). Then the tube was purged with argon and sealed with a rubber stopper. After that, anhydrous fluorobenzene (1.1 mL) and a solution of catalyst **A** (100 µL, 4 mM, 0.002 equiv., freshly prepared) was added. The tube was further sealed with parafilm and immersed in a preheated 50 ℃ oil bath for 16 h. Upon completion of the reaction, the reaction mixture was subsequently dry loaded onto silica gel 9 and subjected to flash column chromatography with deactivated silica gel for purification.

Method B: An oven dried tube (10 mL) with a stirrer bar was charged with strained cyclopropanated furanoside **19a**, ***ent*-19a** (70.5 mg, 0.2 mmol, 1.0 equiv.), glycosyl acceptor **21c** (0.4 mmol, 2.0 equiv.) and Schreiner's thiourea (1.0 mg, 20 µmol, 0.01 equiv.). The tube was purged with argon and anhydrous fluorobenzene (1.2 mL) was added under argon flow. Then the tube was sealed with a proper cap and placed in the microwave reactor at 50 ℃ for 4 h (power: 250 W, stirring: high). Upon completion of the reaction, the reaction mixture was subsequently dry loaded onto silica gel 9 and subjected to flash column chromatography with deactivated silica gel for purification.

Method C: An oven dried tube with a stirrer bar charged with pyrimidine **21e** (0.4 mmol, 2.0 equiv.) was purged with argon and sealed with a rubber septum. To the tube Bis(trimethylsilyl)acetamide (100 µL, 2.0 equiv.) and anhydrous CH_3CN (1.5 mL) was added. Then the mixture was heated in the 75 ℃ oil bath until the mixture became a clear solution. After that, CH_3CN was removed under high pump to afford a residue. When complete removal of the solvent, the tube was refilled with argon and added with strained cyclopropanated furanoside **19a** (70.5 mg, 0.2 mmol, 1.0 equiv.) dissolved in anhydrous fluorobenzene (1.1 mL) and catalyst **A** (100 µL, 10 mM, 0.005 equiv.). Then the tube was further sealed with parafilm and placed in the preheated 50 ℃ oil bath for 16 h. Upon completion of the reaction, the reaction mixture was subsequently dry loaded onto silica gel 9 and subjected to flash column chromatography with deactivated silica gel for purification.

Method D: An oven dried tube with a stirrer bar was charged with strained cyclopropanated furanoside **19a**, ***ent*-19a** or **19b** (105.8 mg, 0.3 mmol, 1.5 equiv.), Trimethoxybenzene (0.2 mmol, 1.0 equiv.), catalyst **A** (2.0 mg, 1.0 µmol) and $LiClO_4$ (21.3 mg, 0.2 mmol, 1.0 equiv.). The tube was purged with argon and sealed with a rubber stopper. After that, anhydrous fluorobenzene (1.2 mL) was added. The tube was further sealed with parafilm and immersed in a preheated 80 ℃ oil bath for 16. Upon completion of the reaction, the reaction mixture was subsequently dry loaded onto silica gel 9 and subjected to flash column chromatography with deactivated silica gel for purification.

General procedure for thiourea catalyzed strain release glycosylation of pyranosides.

Method E: An oven dried tube with a stirrer bar was charged with strained cyclopropanated pyranoside **22a**, **22b** or **22c** (0.1 mmol, 1.0 equiv.), glycosyl acceptor **21** (0.2 mmol, 2.0 equiv.), catalyst **A** (2 mg, 1 mol%). After that, the tube was purged with argon and dry fluorobenzene (1 mL) was added then the tube was sealed and stirred at room temperature for 12 h. Upon completion of the reaction, the reaction mixture was subsequently dry loaded onto silica gel 9 and subjected to flash column chromatography with deactivated silica gel for purification.

Method F: An oven dried tube with a stirrer bar was charged with strained cyclopropanated pyranoside **22a** (0.1 mmol, 1.0 equiv.), glycosyl acceptor **21c** (0.2 mmol, 2.0 equiv.), catalyst **A** (2 mg, 1 mol%) and 4Å molecular sieves (43 mg). After that, the tube was purged with argon and dry fluorobenzene (1 mL) was added then the tube was sealed and immersed in the preheated 70℃ for 12 h. Upon completion of the reaction, the reaction mixture was subsequently dry loaded onto silica gel 9 and subjected to flash column chromatography with deactivated silica gel for purification.

General procedure for the multicatalytic diversification. (see supplementary figure 4)

Method G: An oven dried tube with a stirrer bar was charged with strained cyclopropanated furanoside **19a**, ***ent*-19a**, **19b** (70.5 mg, 0.2 mmol, 1.0 equiv.), and glycosyl acceptor (0.4 mmol, 2.0 equiv.). Then the tube was purged with argon and sealed with a rubber stopper. After that, anhydrous fluorobenzene (1.1 mL) and a solution of catalyst **A** (100 µL, 4 mM) was added. The tube was further sealed with parafilm and immersed in a preheated 50 ℃ oil bath for 16 h. Upon completion of the reaction, the solvent was removed under reduced pressure to give a residue. Then $Cp^*RuCl(PPh_3)_2$ (5 mol%) was added to the residue. The tube was purged with argon and added with azide (0.4 mmol, 2.0 equiv.) toluene (2.0 mL) solution, sealed and heated at 80 ℃ for 4 h. The mixture was absorbed onto silica 9 and purified by silica gel column chromatography (dry loading).

Method H: An oven dried tube with a stirrer bar was charged with strained cyclopropanated furanoside **19a** (70.5 mg, 0.2 mmol, 1.0 equiv.), and glycosyl acceptor (0.4 mmol, 2.0 equiv.). Then the tube was purged with argon and sealed with a rubber stopper. After that, anhydrous fluorobenzene (1.1 mL) and a solution of catalyst **A** (100 µL, 4 mM) was added. The tube was further sealed with parafilm and immersed in a preheated 50 ℃ oil bath for 16 h. Upon completion of the reaction, the solvent was removed under reduced pressure to give a residue.

To the residue, azide (0.4 mmol, 2.0 equiv.), CH_2Cl_2 (1.0 mL) and H_2O (1.0 mL) was added together with $CuSO_4•5H_2O$ (1.0 mg) and sodium ascorbate (1.9 mg). The resulting suspension was stirred at room temperature for 6 h. The mixture was diluted with 5 mL of dichloromethane and 5 mL of water. The organic phases were separated, dried with sodium sulfate, and concentrated at reduced pressure and purified by column chromatography (dry loading).

Gram-scale reaction. An oven dried round flask with a stirrer bar charged with strained cyclopropanated furanoside **19a** (1.0 g, 2.84 mmol, 1.0 equiv.), and glycosyl acceptor **21a** (1.48 g, 5.67 mmol, 2.0 equiv.) and catalyst **A** (2.9 mg, 0.05 mol%) was purged with argon and sealed with a rubber stopper. Then anhydrous fluorobenzene (10 mL) was added. The flask was further sealed with parafilm and immersed in a preheated 50 ℃ oil bath for 24 h. Upon completion of the reaction, the reaction mixture was subsequently dry loaded onto silica gel 9 and subjected to flash column chromatography with deactivated silica gel for purification using 9:1-3:1 Pentane/Ethyl acetate as eluent to give the product (1.30 g) in 75% yield as a pale yellow syrup.

Method for control experiments and counteranion effect. (see supplementary table 3) An oven dried tube with a stirrer bar was charged with strained cyclopropanated furanoside **19a** (35.2 mg, 0.1 mmol, 1.0 equiv.), and glycosyl acceptor **21a** (0.2 mmol, 2.0 equiv.) and 1 mol% catalyst. Then the tube was purged with argon and sealed with a rubber stopper. After that, anhydrous fluorobenzene (0.6 mL) was added. The tube was further sealed with parafilm and immersed in a preheated 50 ℃ oil bath for 16 h. After cooling to room temperature, the mixture was measured by crude 1H NMR with CH_2Br_2 as the internal standard.

Preparation of stock solution for catalyst A and glycosyl acceptor 21a.
Stock solution **A** (0.02 mM): Catalyst **A** (10 mg, 0.0050 mmol) was dissolved in deuterated fluorobenzene (250 µL) in a vial.
Stock solution **B** (1.0 M): glycosyl acceptor **21a** (650.0 mg, 2.5 mmol) was weighted into a 5 mL vial, then deuterated fluorobenzene was added until the total volume was up to 2.5 mL.

Method for *in situ* NMR reaction monitoring for furanosylation with 19a. (see supplementary table 4-5, supplementary figure 6-9) To a dry NMR tube, cyclopropanated sugar **19a** (35.2 mg, 0.1 mmol) was added. Then solution **B** (200 µL), internal standard CH_2Br_2 (10.5 µL, 26.0 mg, 0.15 mmol) and deuterated fluorobenzene (280 µL) were injected subsequently. The prepared mixture was ready for use. The solution **A** (10 µL) was injected into the above mixture before measurement. After that, the tube was placed in the NMR spectrometer. Two experiments were performed at room temperature and 50℃.

Method for initial rate kinetics. To a dry NMR tube, cyclopropane **19a** (35.2 mg, 0.1 mmol) was added. Then solution **B** (200 µL), internal standard dibromomethane (10.5 µL, 26.0 mg, 0.15 mmol) and deuterated fluorobenzene (280 µL) was injected subsequently. The prepared mixture was ready for use. The solution **A** (10 µL) was injected into the above mixture before measurement. After that, the initial rate data was monitored by ^{1}H NMR analysis at 50 ℃.

For other experiments, corresponding amount of cyclopropane **19a**, glycosyl acceptor **21a** and catalyst **A** was added. Then the volume of deuterated fluorobenzene was adjusted to make the total volume of the tube reach to 0.5 mL. The detailed information for these experiments are listed (see supplementary table 6).

Mathematic analysis for initial rate kinetics. The data points are fitted by polynomial equation. The initial rate was calculated using the 1st derivative of the fitted equation with the time parameter of 0s. The fitting equations are listed. (see supplementary table 7). The graphs for initial rates are plotted (see supplementary figure 10).

Method for calculations for reaction order. (see supplementary table 8, supplementary figure 11) Here we use the equation ln(rate) = *x* ln[conc.] + *constant*. The reaction order for each reactant is determined by the slope of the ln(rate) vs ln[conc.] graph. *x* denotes the reaction order, [conc.] represents the concentration.

Method for in situ characterization for intermediate 27a. (see supplementary figure 12-15) Compound **19a** (35.2 mg, 0.1 mmol), **21a** (0.1 M, 200 µL) and deuterated fluorobenzene (200 µL) was added to a NMR tube, the solution was cooled down in the ice-water cooling bath. Before measuring, catalyst solution (0.002 M, 100 µL) was added to the mixture. Then the tube was recorded on the NMR spectrometer at room temperature.

Method for sequential addition control experiment. (see supplementary figure 16) To a dried NMR tube were added compound **19a** (35.2 mg, 0.1 mmol) and catalyst **A** (0.4 mg, 0.2 mol%). Then deuterated fluorobenzene (0.5 mL) and dioxane (12 µL, as internal standard) were added. The tube was left at room temperature for 8 h. After that, acceptor **21a** (52 mg, 0.2 mmol) was added and the tube was sealed and placed in the 50 ℃ for 12 h.

Method for characterization for intermediate 27b. (see supplementary figure 17-21) To a dried NMR tube were added compound **19a** (35.2 mg, 0.1 mmol), catalyst **A** (0.4 mg, 0.2 mol%) and deuterated fluorobenzene (0.5 mL). The tube was left at room temperature for 8 h then recorded on the NMR spectrometer at room temperature.

Synthetic methods and characterization data

Synthesis of starting material cyclopropanated sugar: 1,2-cyclopropanated-1,2-dexoy-furanoses (**19a**, ***ent*-19a**, **19b**) 1,2-cyclopropanated-1,2-deoxy-pyranosides (**22a**, **22b**, **22c**) were synthesized according to the known procedure in the literatures except the tosylation step (see supplementary figure 1 and 2).[2-5]

Synthesis of **19a-2**: To a suspension of D-xylose (42.0 g, 280 mmol) and anhydrous copper(II) sulfate (70.0 g, 440 mmol) in acetone (800 mL), concentrated sulfuric acid (4 mL) was added. The reaction mixture was stirred overnight, neutralized with sodium hydrogen carbonate, and filtered off, and the filter cake was washed thoroughly with acetone. The combined organic layers were evaporated and dried in vacuo. The formed 1,2,3,5-di-*O*-isopropylidene-α-D-xylofuranose was dissolved in 250 mL of a 0.2% aqueous hydrochloric acid and stirred at rt

until the TLC indicated the complete conversion to the desired mono-isopropylidene derivative. After neutralization the reaction mixture was concentrated to a small volume and extracted with ethyl acetate. The organic layer was dried with sodium sulfate, filtered off, and evaporated to obtain the product as white solid (32.0 g, 89% yield).

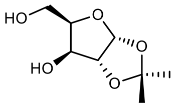

19a-2

^{1}H NMR (400 MHz, DMSO-d_6) δ 5.80 (d, J = 3.8 Hz, 1 H), 5.11 (d, J = 4.8 Hz, 1 H), 4.59 (t, J = 5.6 Hz, 1 H), 4.37 (d, J = 3.8 Hz, 1 H), 4.06-3.92 (m, 2 H), 3.61 (dt, J = 11.3, 5.6 Hz, 1 H), 3.50 (dt, J = 11.5, 5.7 Hz, 1 H), 1.37 (s, 3 H), 1.23 (s, 3 H) ppm. The spectra data is in accordance to the literature.[3]

Synthesis of **19a-3**: A 1 M solution of 1,2-isopropylidene-α-D-ribofuranose (15.0 g, 78.9 mmol) in absolute DMF (75 mL) was added dropwise at rt to a 1.3 M suspension of sodium hydride (60% in mineral oil, 2.3 equiv) in absolute DMF (100 mL) over 30 min. After stirring the mixture until release of H_2 stopped, it was cooled to 10°C, and benzyl bromide (24.0 mL, 2.5 eq) in 25 mL DMF was added dropwise over 1 h. The reaction mixture was allowed to reach rt and was stirred for 12 h, the colour of crude mixture became brown yellow. Then the reaction was quenched by careful addition of water (1% v/v). Then addition of water 150 mL, and extracted three times with EtOAc (150 mL), and the combined organic layers were washed with brine, dried over $MgSO_4$, filtered, and concentrated. The residue obtained was chromatographed (EtOAc/cyclohexane 1:9 to 3:7) to give the desired product as a syrup (27.0 g, 91% yield).

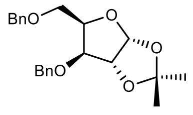

19a-3

^{1}H NMR (400 MHz, $CDCl_3$) δ 7.38-7.20 (m, 10 H), 5.94 (d, J = 3.8 Hz, 1 H), 4.67 (d, J = 12.0 Hz, 1 H), 4.65-4.60 (m, 2 H), 4.52 (dd, J = 12.0, 3.2 Hz, 2 H), 4.42 (td, J = 6.1, 3.2 Hz, 1 H), 3.99 (dd, J = 3.3, 0.6 Hz, 1 H), 3.85-3.68 (m, 2 H), 1.49 (s, 3 H), 1.32 (s, 3 H) ppm. The spectra data is in accordance to the literature.[3]

Synthesis of **19a-4**: Under nitrogen atmosphere, a solution of the 1,2-*O*-isopropylidene-xylofuranose derivatives (1.1 g, 3 mmol) in 6 mL of dry CH_2Cl_2 at 0 ˚C was treated with allyltrimethylsilane (4.5 mmol) and BF_3•OEt_2 (4.5 mmol) in dry CH_2Cl_2 was added at 0 ℃ for 15 min. The reaction mixture was warmed to room temperature and allowed to react overnight. The reaction mixture was treated with a saturated aqueous solution of $NaHCO_3$ (6 mL) and extracted with CH_2Cl_2 (3 × 6 mL). The organic phase was dried with Na_2SO_4, concentrated in vacuo, and purified by column chromatography on silica gel (petroleum ether/ethyl acetate = 3:1) to afford product as colorless syrup (800.0 mg, 75% yield).

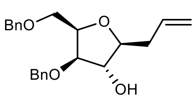

19a-4

^{1}H NMR (400 MHz, $CDCl_3$) δ 7.32-7.15 (m, 10 H), 5.78 (dddd, J = 16.8, 10.2, 7.6, 6.5 Hz, 1 H), 5.12-4.96 (m, 2 H), 4.55 (dd, J = 12.0, 4.2 Hz, 2 H), 4.46 (dd, J = 12.0, 3.0 Hz, 2 H), 4.21-4.13 (m, 1 H), 3.95 (dd, J = 4.7, 2.6 Hz, 1 H), 3.84 (dd, J = 5.1, 2.6 Hz, 1 H), 3.71-3.59 (m, 3 H), 2.47-2.39 (m, 1 H), 2.36-2.27 (m, 1 H) ppm. The spectra data is in accordance to the literature.[3]

Synthesis of **19a-5**: To a solution of 3-*C*-(3,5-Di-*O*-benzyl-α-D-xylofuranosyl)-propene (1.0 mmol) and pyridine (10.0 equiv.) was added anhydrous DCE (3 mL). The mixture was stirred

at 0 deg. Then TsCl (5.0 equiv.) was added slowly and stirred at the same temperature for 10 min. After that, the reaction mixture was allowed to stir at 70 deg for 18 h. The reaction was stopped when the starting material was consumed completely. The mixture was diluted by CH_2Cl_2 (2 mL) and washed with 10 wt% HCl (2 mL), water and brine, then dried over Na_2SO_4. The organic phase was concentrated in vacuum and flash column chromatography (Ethyl acetate/Pentane 1:19 to 1:9 (v/v)) to afford product a pale yellow syrup (426.0 mg, 84% yield).

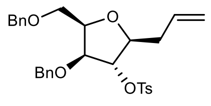

19a-5

^{1}H NMR (400 MHz, $CDCl_3$) δ 7.79 (d, *J* = 8.4 Hz, 2 H), 7.36-7.24 (m, 12 H), 5.64-5.54 (m, 1 H), 4.96-4.89 (m, 2 H), 4.66 (d, *J* = 2.8 Hz, 1 H), 4.60-4.55 (m, 2 H), 4.49 (d, *J* = 12 Hz, 1 H), 4.41 (d, *J* = 12.0 Hz, 1 H), 4.19-4.16 (m, 1 H), 4.10 (d, *J* = 7.6 Hz, 1 H), 3.94-3.91 (m, 1 H), 3.71 (d, *J* = 6.4 Hz, 2 H), 2.46 (s, 3 H), 2.34-2.29 (m, 2 H) ppm. MS (ESI): 531.0 (M+Na). $[\alpha]_D^{20}$ = -50.5 (c = 0.97, CH_2Cl_2). The spectra data is in accordance to the literature.[3]

Synthesis of **19a-6**: To a solution of **19a-5** (15.3 g, 30 mmol) and $Hg(OAc)_2$ (2.87 g, 9 mmol) in acetone/water (220 mL, 4:1 v/v) was added dropwise a solution of Jones reagent (2 M, 40 mL) at 0 °C. The dark greenish brown mixture was stirred for 23 h at rt and then poured into water (60 mL). The aqueous mixture was extracted with CH_2Cl_2 (120 mL). The organic layer was successively washed with water twice. water phase was extracted with CH_2Cl_2, dried over anhydrous Na_2SO_4 The filtrate was concentrated in vacuo and the residue was purified by silica gel flash column chromatrography to afford the desired compound as a syrup (11.9 g, 76% yield).

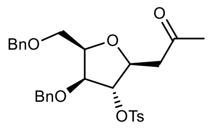

19a-6

^{1}H NMR (400 MHz, $CDCl_3$) δ 7.81 (d, *J* = 8.4 Hz, 2 H), 7.36-7.27 (m, 10 H), 7.22-7.20 (m, 2 H), 4.78 (dd, *J* = 2.9, 1.4 Hz, 1 H); 4.56-4.46 (m, 3 H), 4.38 (d, *J* = 11.6 Hz, 1 H), 4.32-4.28 (m, 1 H), 4.19-4.15 (m, 1 H), 4.10 (dd, *J* = 4.0, 1.4 Hz, 1 H), 3.70-3.62 (m, 2 H), 2.77 (dd, *J* = 16.2, 7.6 Hz, 1 H), 2.63 (dd, *J* = 16.3, 5.5 Hz, 1 H), 2.44 (s, 3 H), 2.09 (s, 3 H) ppm. MS (ESI): 547.0 (M+Na). $[\alpha]_D^{20}$ = -75.2 (c = 0.88, CH_2Cl_2). The spectra data is in accordance to the literature.[3]

Synthesis of **19a**: To a solution of **19a-6** (3.2 g) in DMSO (75 mL) was added K_2CO_3 (5.9 g). The mixture was stirred at 60 °C for 18 h. After cooled to room temperature, the reaction mixture was extracted with ethyl acetate (50 mL), and washed with brine. The organic phase was dried with Na_2SO_4, concentrated in vacuo, and purified by column chromatography on silica gel (petroleum ether/ethyl acetate = 6:1) to afford the product as colorless syrup (1.2 g, 58% yield).

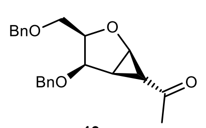

19a

^{1}H NMR (400 MHz, Acetone-d_6) δ 7.38-7.26 (m, 10 H), 4.62-4.58 (m, 2 H), 4.55-4.47 (m, 3 H), 4.43 (d, *J* = 11.6 Hz, 1 H), 4.06 (dd, *J* = 5.7, 1.2 Hz, 1 H), 3.70 (dd, *J* = 10.8, 4.0 Hz, 1 H), 3.49 (dd, *J* = 10.8, 4.0 Hz, 1 H), 2.60-2.57 (m, 1 H), 2.54-2.53 (m, 1 H), 2.19 (s, 3 H) ppm; ^{13}C NMR (100 MHz, Acetone-d_6) δ 203.22, 139.85, 139.32, 129.22, 129.08, 128.57, 128.42, 128.37, 128.20, 89.08, 79.47, 73.64, 71.45, 69.98, 68.89, 37.22, 33.80, 30.75 ppm. MS (ESI): 375.0 (M+Na). $[\alpha]_D^{20}$ = -101.2 (c = 0.48, CH_2Cl_2). The spectra data is in accordance to the literature.[3]

Synthesis of ***ent*-19a-2**: To a suspension of L-xylose (17.8 g, 118 mmol) and anhydrous copper(II) sulfate (37.8 g, 237 mmol) in acetone (260 mL), concentrated sulfuric acid (1.8 mL) was added. The reaction mixture was stirred overnight, neutralized with sodium hydrogen carbonate, and filtered off, and the filter cake was washed thoroughly with acetone. The combined organic layers were evaporated and dried in vacuo. The formed 1,2,3,5-di-*O*-isopropylidene-α-L-xylofuranose was dissolved in 115 mL of a 0.2% aqueous hydrochloric acid and stirred at rt until the TLC indicated the complete conversion to the desired mono-isopropylidene derivative. After neutralization the reaction mixture was concentrated to a small volume and extracted with ethyl acetate. The organic layer was dried with sodium sulfate, filtered off, and evaporated to obtain the product as white solid (19.0 g, 84% yield).

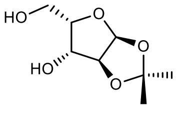

***ent*-19a-2**

^{1}H NMR (400 MHz, DMSO-d_6) δ 5.80 (d, *J* = 3.8 Hz, 1 H), 5.11 (d, *J* = 4.8 Hz, 1 H), 4.59 (t, *J* = 5.6 Hz, 1 H), 4.37 (d, *J* = 3.8 Hz, 1 H), 4.06-3.92 (m, 2 H), 3.61 (dt, *J* = 11.3, 5.6 Hz, 1 H), 3.50 (dt, *J* = 11.5, 5.7 Hz, 1 H), 1.37 (s, 3 H), 1.23 (s, 3 H) ppm.

Synthesis of ***ent*-19a-3**: A 1 M solution of 1,2-isopropylidene-α-L-ribofuranose (15.0 g, 78.9 mmol) in absolute DMF (75 mL) was added dropwise at rt to a 1.3 M suspension of sodium hydride (60% in mineral oil, 2.3 equiv) in absolute DMF (100 mL) over 30 min. After stirring the mixture until release of H_2 stopped, it was cooled to 10°C, and benzyl bromide (24.0 mL, 2.5 eq) in 25 mL DMF was added dropwise over 1 h. The reaction mixture was allowed to reach rt and was stirred for 12 h, the colour of crude mixture became brown yellow. Then the reaction was quenched by careful addition of water (1% v/v). Then addition of water 150 mL, and extracted three times with EtOAc (150 mL), and the combined organic layers were washed with brine, dried over $MgSO_4$, filtered, and concentrated. The residue obtained was chromatographed (EtOAc/cyclohexane 1:9 to 3:7) to give the desired product as a syrup (27.0 g, 91% yield).

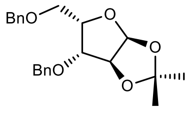

***ent*-19a-3**

^{1}H NMR (400 MHz, $CDCl_3$) δ 7.38-7.20 (m, 10 H), 5.94 (d, *J* = 3.8 Hz, 1 H), 4.67 (d, *J* = 12.0 Hz, 1 H), 4.65-4.60 (m, 2 H), 4.52 (dd, *J* = 12.0, 3.2 Hz, 2 H), 4.42 (td, *J* = 6.1, 3.2 Hz, 1 H), 3.99 (dd, *J* = 3.3, 0.6 Hz, 1 H), 3.85-3.68 (m, 2 H), 1.49 (s, 3 H), 1.32 (s, 3 H) ppm.

Synthesis of ***ent*-19a-4**: Under nitrogen atmosphere, a solution of the ***ent*-19a-3** (24.6 g, 66 mmol) in 200 mL of dry CH_2Cl_2 at 0 ˚C was treated with allyltrimethylsilane (11.4 g, 15.8 mL, 100 mmol) and BF_3•OEt_2 (14.3 g, 12.5 mL, 100 mmol) in dry CH_2Cl_2 was added at 0 ℃ for 15 min. The reaction mixture was warmed to room temperature and allowed to react overnight. The reaction mixture was treated with a saturated aqueous solution of $NaHCO_3$ (120 mL) and extracted with CH_2Cl_2 (3 × 120 mL). The organic phase was dried with Na_2SO_4, concentrated in vacuo, and purified by column chromatography on silica gel (petroleum ether/ethyl acetate = 3:1) to afford product as colorless syrup (21.0 g, 89% yield).

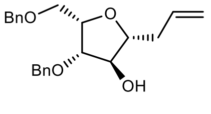

***ent*-19a-4**

^{1}H NMR (400 MHz, $CDCl_3$) δ 7.32-7.15 (m, 10 H), 5.78 (dddd, *J* = 16.8, 10.2, 7.6, 6.5 Hz, 1 H), 5.12-4.96 (m, 2 H), 4.55 (dd, *J* = 12.0, 4.2 Hz, 2 H), 4.46 (dd, *J* = 12.0, 3.0 Hz, 2 H), 4.21-4.13 (m, 1 H), 3.95 (dd, *J* = 4.7, 2.6 Hz,

1 H), 3.84 (dd, J = 5.1, 2.6 Hz, 1 H), 3.71-3.59 (m, 3 H), 2.47-2.39 (m, 1 H), 2.36-2.27 (m, 1 H) ppm.

Synthesis of ***ent*-19a-5**: To a solution of ***ent*-19a-4** (20.0 g, 56 mmol) and pyridine (44.6 g, 564 mmol) was added anhydrous DCE (150 mL). The mixture was stirred at 0 ℃. Then TsCl (53.8 g, 282 mmol) was added slowly and stirred at the same temperature for 10 min. After that, the reaction mixture was allowed to stir at 70 ℃ for 18 h. The reaction was stopped when the starting material was consumed completely. The mixture was diluted by CH_2Cl_2 (120 mL) and washed with 10 wt% HCl (120 mL), water and brine, then dried over Na_2SO_4. The organic phase was concentrated in vacuum and flash column chromatography (Ethyl acetate/Pentane 1:19 to 1:9 (v/v)) to afford product a pale yellow syrup (20.7 g, 72% yield).

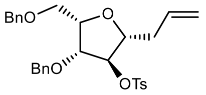

***ent*-19a-5**

^{1}H NMR (400 MHz, $CDCl_3$) δ 7.79 (d, J = 8.4 Hz, 2 H), 7.36-7.24 (m, 12 H), 5.64-5.54 (m, 1 H), 4.96-4.89 (m, 2 H), 4.66 (d, J = 2.8 Hz, 1 H), 4.60-4.55 (m, 2 H), 4.49 (d, J = 12 Hz, 1 H), 4.41 (d, J = 12 Hz, 1 H), 4.19-4.16 (m, 1 H), 4.10 (d, J = 7.6 Hz, 1 H), 3.94-3.91 (m, 1 H), 3.71 (d, J = 6.4 Hz, 2 H), 2.46 (s, 3 H), 2.34-2.29 (m, 2 H) ppm. MS (ESI): 531.0 (M+Na). $[\alpha]_D^{20}$ = +60.7 (c = 0.90, CH_2Cl_2).

Synthesis of ***ent*-19a-6**: To a solution of ***ent*-19a-5** (20.0 g, 39.3 mmol) and $Hg(OAc)_2$ (3.7 g, 11.8 mmol) in acetone/water (270 mL, 4:1 v/v) was added dropwise a solution of Jones reagent (2 M, 45 mL) at 0 ℃. The dark greenish brown mixture was stirred for 23 h at rt and then poured into water (80 mL). The aqueous mixture was extracted with CH_2Cl_2 (160 mL). The organic layer was successively washed with water twice. water phase was extracted with CH_2Cl_2, dried over anhydrous Na_2SO_4 The filtrate was concentrated in vacuo and the residue was purified by silica gel flash column chromatrography to afford the desired compound as a syrup (16.1 g, 78% yield).

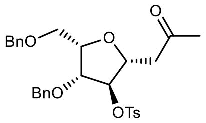

***ent*-19a-6**

^{1}H NMR (400 MHz, $CDCl_3$) δ 7.81 (d, J = 8.4 Hz, 2 H), 7.36-7.27 (m, 10 H), 7.22-7.20 (m, 2 H), 4.78 (dd, J = 2.9, 1.4 Hz, 1 H); 4.56-4.46 (m, 3 H), 4.38 (d, J = 11.6 Hz, 1 H), 4.32-4.28 (m, 1 H), 4.19-4.15 (m, 1 H), 4.10 (dd, J = 4.0, 1.4 Hz, 1 H), 3.70-3.62 (m, 2 H), 2.77 (dd, J = 16.2, 7.6 Hz, 1 H), 2.63 (dd, J = 16.3, 5.5 Hz, 1 H), 2.44 (s, 3 H), 2.09 (s, 3 H) ppm. MS (ESI): 547.0 (M+Na). $[\alpha]_D^{20}$ = +153.6 (c = 0.39, CH_2Cl_2).

Synthesis of ***ent*-19a**: To a solution of ***ent*-19a-6** (16.0 g) in DMSO (300 mL) was added K_2CO_3 (29.0 g). The mixture was stirred at 60 ℃ for 18 h. After cooled to room temperature, the reaction mixture was extracted with ethyl acetate (180 mL), and washed with brine. The organic phase was dried with Na_2SO_4, concentrated in vacuo, and purified by column chromatography on silica gel (petroleum ether/ethyl acetate = 6:1) to afford the product as a white solid (4.5 g, 42% yield).

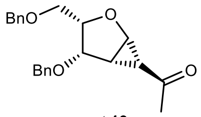

***ent*-19a**

^{1}H NMR (400 MHz, Acetone-d_6) δ 7.38-7.26 (m, 10 H), 4.62-4.58 (m, 2 H), 4.55-4.47 (m, 3 H), 4.43 (d, J = 11.6 Hz, 1 H), 4.06 (dd, J = 5.7, 1.2 Hz, 1 H), 3.70 (dd, J = 10.8, 4.0 Hz, 1 H), 3.49 (dd, J = 10.8, 4.0 Hz, 1 H), 2.60-2.57 (m, 1 H), 2.54-2.53 (m, 1 H), 2.19 (s, 3 H) ppm; ^{13}C NMR (100 MHz,

Acetone-d_6) δ 203.22, 139.85, 139.32, 129.22, 129.08, 128.57, 128.42, 128.37, 128.20, 89.08, 79.47, 73.64, 71.45, 69.98, 68.89, 37.22, 33.80, 30.75 ppm. MS (ESI): 375.0 (M+Na). $[\alpha]_D^{20}$ = +102.2 (c = 0.61, CH_2Cl_2).

Synthesis of **19b-2**: Under nitrogen atmosphere, a solution of the **19b-1** (15.0 g, 40 mmol) in 225 mL of dry CH_2Cl_2 at 0 ˚C was treated with allyltrimethylsilane (6.9 g, 60 mmol) and BF_3•OEt_2 (8.7 g, 60 mmol) in dry CH_2Cl_2 was added at 0 ℃ for 15 min. The reaction mixture was warmed to room temperature and allowed to react overnight. The reaction mixture was treated with a saturated aqueous solution of $NaHCO_3$ (450 mL) and extracted with CH_2Cl_2 (3 × 500 mL). The organic phase was dried with Na_2SO_4, concentrated in vacuo, and purified by column chromatography on silica gel (petroleum ether/ethyl acetate = 3:1) to afford product as colorless syrup (9.6 g, 67% yield).

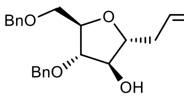

19b-2

^1H NMR (400 MHz, $CDCl_3$) δ 7.42-7.28 (m, 10 H), 5.95-5.77 (m, 1 H), 5.23-5.01 (m, 1 H), 4.63 (d, *J* = 11.8 Hz, 2 H), 4.59-4.46 (m, 2 H), 4.29-4.17 (m, 1 H), 4.13-4.02 (m, 2 H), 3.97 (dt, *J* = 2.8, 1.4 Hz, 1 H), 3.69 (dd, *J* = 10.3, 2.9 Hz, 1 H), 3.57 (ddd, *J* = 10.3, 2.8, 1.1 Hz, 1 H), 3.41 (d, *J* = 9.7 Hz, 1 H), 2.54 -2.31 (m, 2 H). The spectra data is in accordance to the literature.[4]

Synthesis of **19b-3**: To a solution of **19b-2** (9.6 g, 27 mmol) and pyridine (21.2 g, 270 mmol) was added anhydrous DCE (90 mL). The mixture was stirred at 0 ℃. Then TsCl (25.8 g, 135 mmol) was added slowly and stirred at the same temperature for 10 min. After that, the reaction mixture was allowed to stir at 70 ℃. The reaction was stopped when the starting material was consumed completely. The mixture was diluted by CH_2Cl_2 and washed with 10 wt% HCl, water and brine, then dried over Na_2SO_4. The organic phase was concentrated in vacuum and flash column chromatography (Ethyl acetate/Pentane 1:19 to 1:9 (v/v)) to afford product a pale yellow syrup (10.8 g, 78% yield).

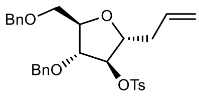

19b-3

^1H NMR (500 MHz, $CDCl_3$) δ 7.80 (d, *J* = 8.0 Hz, 2 H), 7.38-7.31 (m, 10 H), 7.23 (d, *J* = 7.5 Hz, 2 H), 5.72-5.65 (m, 1 H), 5.07-5.03 (m, 2 H), 4.81-4.80 (m, 1 H), 4.54 (s, 2 H), 4.43 (q, *J* = 11.8 Hz, 2 H), 4.19-4.12 (m, 3 H), 3.56-3.52 (m, 2 H), 2.44 (s, 3 H), 2.40-2.36 (m, 2 H) ppm. MS (ESI): 531.0 (M+Na). $[\alpha]_D^{20}$ = +47.8 (c = 1.19, CH_2Cl_2). The spectra data is in accordance to the literature.[4]

Synthesis of **19b-4**: To a solution of **19b-3** (10.8 g, 21.2 mmol) and $Hg(OAc)_2$ (2.0 g) in acetone/water (150 mL, 4:1 v/v) was added dropwise a solution of Jones reagent (2 M, 20 mL) at 0 ℃. The dark greenish brown mixture was stirred for 23 h at rt and then poured into water (40 mL). The aqueous mixture was extracted with CH_2Cl_2 (80 mL). The organic layer was successively washed with water twice. water phase was extracted with CH_2Cl_2, dried over anhydrous Na_2SO_4 The filtrate was concentrated in vacuo and the residue was purified by silica gel flash column chromatrography to afford the desired compound as a syrup (7.4 g, 66% yield).

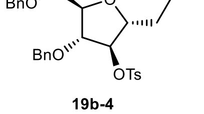

19b-4

^1H NMR (500 MHz, $CDCl_3$) δ 7.72 (d, *J* = 8.3 Hz, 2 H), 7.35-7.26 (m, 10 H), 7.23-7.21 (m, 2 H), 4.95 (d, *J* = 3.1 Hz, 1 H), 4.51-4.46 (m, 3 H), 4.45-4.41 (m, 1 H), 4.41-4.36 (m, 1 H), 3.99-3.96 (m, 1 H), 3.94 (d, *J* = 3.4 Hz, 1 H), 3.53 (dd, *J* = 10.2, 6.0 Hz, 1 H), 3.44 (dd, *J* = 10.2, 5.6 Hz, 1 H), 2.87 (dd, *J*

= 17.9, 6.1 Hz, 1 H), 2.72 (dd, J = 18.0, 7.0 Hz, 1 H), 2.42 (s, 3 H), 2.06 (s, 3 H) ppm. MS (ESI): 547.0 (M+Na). $[\alpha]_D^{20}$ = +36.9 (c = 0.40, CH_2Cl_2). The spectra data is in accordance to the literature.[4]

Synthesis of **19b**: To a solution of **19b-4** (7.4 g, 14 mmol) in DMSO (175 mL) was added K_2CO_3 (13.6 g). The mixture was stirred at 60 ℃ for 18 h. After cooled to room temperature, the reaction mixture was extracted with ethyl acetate (120 mL), and washed with brine. The organic phase was dried with Na_2SO_4, concentrated in vacuo, and purified by column chromatography on silica gel (petroleum ether/ethyl acetate = 6:1) to afford the product as a white solid (2.6 g, 52% yield).

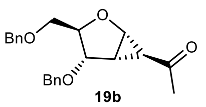
19b

^{1}H NMR (500 MHz, Acetone-d_6) δ 7.39-7.31 (m, 10 H), 4.64 (d, J = 11.9 Hz, 1 H), 4.57-4.49 (m, 3 H), 4.43 (t, J = 5.5 Hz, 1 H), 4.11 (dd, J = 5.5, 1.3 Hz, 1 H), 3.80-3.76 (m, 1 H), 3.57-3.49 (m, 2 H), 2.72 (dd, J = 3.9, 1.3 Hz, 1 H), 2.44-2.41 (m, 1 H), 2.20 (s, 3 H) ppm; ^{13}C NMR (126 MHz, Acetone-d_6) δ 203.81, 139.53, 139.46, 129.15, 129.12, 128.68, 128.43, 128.42, 128.30, 83.87, 81.28, 73.68, 72.12, 70.76, 69.41, 31.98, 30.91, 30.73 ppm. MS (ESI): 375.0 (M+Na). $[\alpha]_D^{20}$ = +142.9 (c = 0.39, CH_2Cl_2). The spectra data is in accordance to the literature.[4]

Synthesis of **22a**: To the solution of tosylated sugar **22a-1** (3.0 g, 5.03 mmol) in DMF (150 mL) was added potassium carbonate (3.53 g). The mixture was stirred at 50 ℃ overnight and then concentrated in vacuo. This residue was extracted with CH_2Cl_2 (150 mL) and water (150 mL), the organic layer was successively washed with water (2×150 mL), brine (2×150mL), dried over anhydrous Na_2SO_4. The filterate was concentrated in vacuo. The residue was purified by silica gel flash column chromatography (Petroleum ether/EtOAc 4:1) to afford the title compound as a white solid (1.3 g, 61% yield).

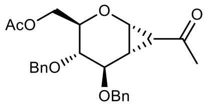
22a

^{1}H NMR (500 MHz, CD_2Cl_2) δ 7.35-7.29 (m, 10 H), 4.70 (dd, J = 11.5, 3.1 Hz, 2 H), 4.58-4.54 (m, 2 H), 4.36-4.31 (m, 1 H), 4.11-4.07 (m, 1 H), 3.81-3.73 (m, 3 H), 3.49-3.46 (m, 1 H), 2.38-2.35 (m, 1 H), 2.22 (s, 3 H), 2.02 (s, 3 H), 1.97-1.92 (m, 1 H) ppm; ^{13}C NMR (126 MHz, CD_2Cl_2) δ 205.12, 171.07, 138.56, 138.47, 128.96, 128.92, 128.47, 128.35, 128.33, 128.32, 75.69, 73.58, 73.55, 71.87, 63.57, 60.34, 54.43, 33.08, 31.42, 26.79, 21.20 ppm. MS (ESI): 447.0 (M+Na); $[\alpha]_D^{20}$ = +33.4 (c = 0.72, CH_2Cl_2). The spectra data is in accordance to the literature.[2]

Synthesis of **22b**: To the solution of tosylated sugar **22b-1** (2.9 g, 4.5 mmol) in MeOH (150 mL) was added potassium carbonate (1.89 g). The mixture was stirred at room temperature overnight then filtrated and concentrated in vacuo. The residue was purified by silica gel flash column chromatography (Petroleum ether/EtOAc 6:1) to afford the title compound as a pale syrup (500.0 mg, 24% yield).

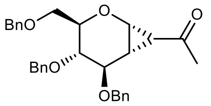
22b

^{1}H NMR (400 MHz, CD_2Cl_2) δ 7.35-7.31 (m, 15 H), 4.73-4.67 (m, 2 H), 4.59-4.54 (m, 2 H), 4.51 (s, 2 H), 3.81-3.76 (m, 2 H), 3.73-3.70 (m, 2 H), 3.60-3.55 (m, 2 H), 2.37-2.34 (m, 1 H), 2.23 (s, 3 H), 1.94-1.89 (m, 1 H) ppm; ^{13}C NMR (126 MHz, CD_2Cl_2) δ 205.32, 138.88 (*two carbons*), 138.64, 128.90, 128.85, 128.85, 128.41, 128.30, 128.24 (two carbons), 128.21, 128.10, 76.49, 76.02,

75.03, 73.81, 73.69, 71.80, 69.92, 60.88, 33.29, 31.41, 27.07 ppm. MS (ESI): 495.2 (M+Na); $[\alpha]_D^{20}$ = +64.6 (c = 1.70, CH_2Cl_2). The spectra data is in accordance to the literature.[2]

Synthesis of **22c**: To the solution of tosylated sugar **22c-1** (16.3 g, 25.3 mmol) in DMSO (250 mL) was added potassium carbonate (17.5 g). The mixture was stirred at 70 ℃ for 23 h then cooled to room temperature and diluted with EtOAc, washed with water, brine and dried over Na_2SO_4. The filtrate was concentrated in vacuo. The residue was purified by silica gel flash column chromatography (Petroleum ether/EtOAc 6:1) to afford the title compound as a white solid (8.4 mg, 70% yield).

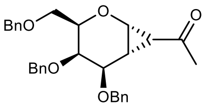

22c

^1H NMR (500 MHz, CD_2Cl_2) δ 7.37-7.30 (m, 15 H), 4.85 (d, *J* = 11.4 Hz, 1 H), 4.77 (d, *J* = 11.9 Hz, 1 H), 4.64 (d, *J* = 11.9 Hz, 1 H), 4.57 (d, *J* = 11.5 Hz, 1 H), 4.50 (d, *J* = 11.8 Hz, 1 H), 4.44 (d, *J* = 11.8 Hz, 1 H), 3.82 (dd, *J* = 7.3, 2.3 Hz, 1 H), 3.74-3.72 (m, 2 H), 3.71-3.60 (m, 3 H), 2.23 (s, 3 H), 2.08-2.01 (m, 2 H) ppm; ^{13}C NMR (126 MHz, CD_2Cl_2) δ 205.22, 139.13, 138.88, 138.78, 128.89, 128.84, 128.80, 128.48, 128.34, 128.15, 128.05, (*two aromatic carbons overlaped in this region*) 76.00, 74.73, 74.10, 73.80, 72.73, 71.81, 69.75, 61.30, 34.37, 31.22, 26.64 ppm. MS (ESI): 495.2 (M+Na); $[\alpha]_D^{20}$ = +41.4 (c = 0.85, CH_2Cl_2). The spectra data is in accordance to the literature.[2]

NaBArF was synthesized using the standard procedure in the literature.[6]
Synthesis of **NaBArF**: A 2.0 M solution of *i*-PrMgCl in THF (100 mL, 0.2 mol) was added by addition funnel over 45 min to a stirred solution of 1-bromo-3,5-bis(trifluoromethyl)benzene (31.3 mL) in THF (150 mL) chilled to -20 °C. After the reaction mixture was warmed from -20 to 0 °C over 1 h, $NaBF_4$ (3.3 g, dried under high pump at 130 °C overnight before use) was quickly added as a solid under a stream of N_2. The mixture then was stirred for 48 h at 23 °C (under argon). The contents were then poured into a solution of Na_2CO_3 (55.0 g) and $NaHCO_3$ (25.0 g) in water (750 mL) (all workup procedures are not airsensitive). This mixture was stirred vigorously for 1 h and then extracted with diethyl ether (4×250 mL). The combined organic layers were washed with brine and dried over anhydrous Na_2SO_4. After filtration of the mixture and rotary evaporation of the filtrate, the crude residue was dried at 100 °C/0.1 Torr for 10 h to yield a tan-yellow solid. Dichloromethane, chilled to -25 °C (3×25 mL), was used to wash all colored contaminant and filter it away from the product, a fine white powder. The product was dried in vacuo to yield pure, anhydrous NaBArF as a hygroscopic white powder (4.2 g, 16% yield).
^1H NMR (400 MHz, Acetone-d_6) δ 7.80-7.77 (m, 8 H), 7.73-7.57 (m, 4 H) ppm. The spectra data is in accordance to the literature.[6]

Kass catalyst **A** (BArF hydrogen bonding) was synthesized in accordance to a known procedure in the literature.[7]
Synthesis of Kass catalyst **A**: To a 3.5 g (3.9 mmol) of sodium tetrakis(3,5-bis(trifluoromethyl)phenyl)borate, 1.0 g (1.9 mmol) of 1-methyl-3-(3-phenylthio-ureido)pyridinium iodide and 100 mL of CH_2Cl_2 were added. This mixture was stirred at room temperature under an argon atmosphere until the solid material was totally dissolved and a cloudy suspension formed. Stirring was then stopped and the solution was left undisturbed until

a white solid precipitated and a clear solution formed. The reaction mixture was then filtered and concentrated under reduced pressure to afford title compound as a yellow solid (3.8 g, 98%). ^{1}H NMR (400 MHz, CD_2Cl_2) δ 9.48 (s, 2 H), 8.24 (d, J = 7.2 Hz, 4 H), 7.94 (t, J = 7.3 Hz, 2 H), 7.74-7.713 (m, 16 H), 7.57 (s, 8 H), 4.37 (s, 6 H) ppm. The spectra data is in accordance to the literature.[7]

1-Methylpyridium tetrakis(3,5-bis(tri-fluoromethyl)phenyl)borate

Preparation of 1-methylpyridium tetrakis(3,5-bis(tri-fluoromethyl)phenyl)borate **F**: To a 10 mL vial, 88 mg (0.1 mmol) of sodium tetrakis(3,5-bis(tri-fluoromethyl)phenyl)borate, 22 mg (0.1 mmol) of 1-Methylpyridium iodide and 2 mL of CH_2Cl_2 were added. This heterogeneous solution was stirred at room temperature under an inert atmosphere until the solids totally dissolved and a cloudy suspension formed. Stirring was then stopped and the resulting mixture was left alone until a white solid precipitated and a clear solution resulted. It was then filtered and concentrated under reduced pressure to afford the product as pale yellow solid, 76.4 mg, yield 80%.

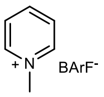

F

^{1}H NMR (500 MHz, CD_2Cl_2) δ 8.54-8.52 (m, 3 H), 8.03 (t, J = 7.1 Hz, 2 H), 7.74-7.70 (m, 8 H), 7.56 (s, 4 H), 4.38 (s, 3 H) ppm; ^{13}C NMR (126 MHz, CD_2Cl_2) δ 162.29 (q, $^1J_{B\text{-}C}$ = 49.4 Hz), 146.93, 144.87-144.72 (m, 1 C), 129.65, 129.41 (qq, J = 31.6, 3.0 Hz), 125.10 (q, $^1J_{F\text{-}C}$ = 272.9 Hz), 118.11-118.02 (m, 1 C), 117.99, 49.70 ppm. HRMS (ESI): Calculated for $C_{38}H_{21}NBF_{24}$ (M+H): 958.13838, Found: 958.13783.

3,3'-(thiocarbonylbis(azanediyl))bis(1-methylpyridin-1-ium) triflate

Preparation of 3,3'-(thiocarbonylbis(azanediyl))bis(1-methylpyridin-1-ium) triflate **K**: AgOTf (27.0 mg, 0.11 mmol) was added to a 10 mL flask containing a suspension of 3,3'-(thiocarbonylbis(azanediyl))bis(1-methylpyridin-1-ium) iodide **J** (25.7 mg, 0.05 mmol) in CH_2Cl_2 (2 mL). The mixture was stirred at room temperature for 2 h. The mixture was filtered through a HPLC filter. The solvent of filtrate was removed under rotavapor and the resulting residue was further dried under high pump to provide the product (5.0 mg, 18% yield).

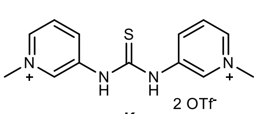

K

^{1}H NMR (700 MHz, CD_3CN) δ 10.07 (s, 2 H), 9.23 (s, 2 H), 8.64 (d, J = 8.6 Hz, 2 H), 8.41 (d, J = 6.0 Hz, 2 H), 7.97 (dd, J = 8.5, 6.1 Hz, 2 H), 4.30 (s, 6 H) ppm; ^{13}C NMR (176 MHz, CD_3CN) δ 181.49, 142.13, 140.63, 140.37, 140.03, 128.54, 121.84 (q, J = 319.8 Hz), 49.71 ppm. HRMS (ESI): Calculated for $C_{15}H_{16}O_6N_4F_6NaS_3$ (M+Na): 581.00284, Found: 581.00285.

6-*O*-(3,5-*O*-di-benzyl-2-*C*-acetylmethyl-2-deoxy-α-D-lyxofuranosyl)-1,2:3,4-di-*O*-isopropylidene-α-D-galactopyranoside

The title product compound is prepared using **Method A** and isolated by flash column chromatography (9:1-3:1 Pentane: Ethyl Acetate) giving a pale yellow syrup (109.8 mg, 0.18 mmol, 90% yield, α/β ratio 93:7).

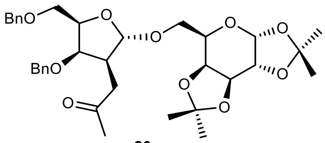

20a

^{1}H (400 MHz, Acetone-d$_6$) δ 7.37-7.28 (m, 10 H), 5.45 (d, *J* = 5.0 Hz, 1 H), 4.87 (d, *J* = 4.5 Hz, 1 H), 4.61 (dd, *J* = 7.9, 2.4 Hz, 1 H), 4.58-4.53 (m, 3 H), 4.36-4.32 (m, 3 H), 4.32-4.28 (m, 2 H), 3.99 (td, *J* = 6.7, 1.7 Hz, 1 H), 3.79 (dd, *J* = 9.7, 6.6 Hz, 1 H), 3.66-3.62 (m, 3 H), 2.83 (dd, *J* = 17.0, 8.9 Hz, 1 H), 2.67-2.56 (m, 2 H), 2.04 (s, 3 H), 1.47 (s, 3 H), 1.37 (s, 3 H), 1.32 (s, 3 H), 1.31 (s, 3 H) ppm; ^{13}C NMR (176 MHz, Acetone-d$_6$) δ 207.25, 139.51, 139.48, 129.12, 129.10, 128.64, 128.56, 128.38, 128.28, 109.50, 108.95, 108.93, 97.20, 80.82, 80.77, 74.80, 73.82, 71.71, 72.55, 71.47, 69.27, 67.93, 66.92, 46.59, 40.26, 30.01, 26.45, 26.44, 25.25, 24.75 ppm. MS (ESI): 635.4 (M+H). $[\alpha]_D^{20}$ = +3.9 (c = 0.47, CH_2Cl_2). The spectra data is in accordance to the literature.[5]

1-*O*-methyl-5-*O*-(3,5-*O*-di-benzyl-2-*C*-acetylmethyl-2-deoxy-α-D-lyxofuranosyl)-2,3-*O*-isopropylidene-β-D-ribofuranoside

The title product compound is prepared using **Method A** and isolated by flash column chromatography (9:1-3:1 Pentane: Ethyl Acetate) giving a pale yellow syrup (92.0 mg, 0.17 mmol, 83% yield, α/β ratio 83:17).

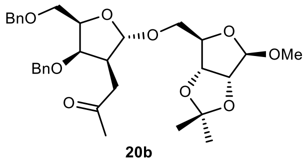

20b

^{1}H NMR (500 MHz, Acetone-*d*$_6$) δ 7.39-7.27 (m, 10 H), 4.88-4.86 (m, 1 H), 4.84 (d, *J* = 4.3 Hz, 1 H), 4.70 (d, *J* = 6.0 Hz, 1 H), 4.57 (d, *J* = 5.6 Hz, 2 H), 4.55 (d, *J* = 5.8 Hz, 2 H), 4.37-4.33 (m, 2 H), 4.29 (dd, *J* = 5.4, 4.0 Hz, 1 H), 4.22 (dd, *J* = 8.9, 6.0 Hz, 1 H), 3.80 (dd, *J* = 9.8, 6.4 Hz, 1 H), 3.66 (td, *J* = 10.3, 5.9 Hz, 2 H), 3.39-3.34 (m, 1 H), 3.26 (s, 3 H), 2.85 (dd, *J* = 17.5, 9.3 Hz, 1 H), 2.71-2.66 (m, 1 H), 2.60 (dd, *J* = 17.5, 5.3 Hz, 1 H), 2.04 (s, 3 H), 1.39 (s, 3 H), 1.27 (s, 3 H) ppm; ^{13}C NMR (126 MHz, Acetone-*d*$_6$) δ 207.30, 139.62, 139.53, 129.18, 129.16, 128.71, 128.60, 128.44, 128.34, 112.63, 110.11, 108.74, 86.07, 85.96, 83.05, 80.83, 80.80, 74.84, 73.83, 70.06, 69.37, 54.86, 46.62, 40.36, 30.07, 26.81, 25.13 ppm. MS (ESI): 579.2 (M+Na). $[\alpha]_D^{20}$ = +11.6 (c = 0.29, CH_2Cl_2). The spectra data is in accordance to the literature.[5]

3-*O*-(3,5-*O*-di-benzyl-2-*C*-acetylmethyl-2-deoxy-α-D-lyxofuranosyl)-1,2:5,6-*O*-di-isopropylidene-α-D-glucofuranoside

The title product compound is prepared using **Method B** and isolated by flash column chromatography (9:1-3:1 Pentane: Ethyl Acetate) giving a white solid (100.2 mg, 0.16 mmol, 81% yield, α only).

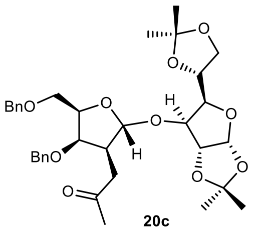

20c

^{1}H NMR (700 MHz, Acetone-*d*$_6$) δ 7.38 (d, *J* = 7.1 Hz, 2 H), 7.35-7.32 (m, 4 H), 7.29-7.28 (m, 4 H), 5.84 (d, *J* = 3.5 Hz, 1 H), 5.01 (d, *J* = 4.3 Hz, 1 H), 4.71 (d, *J* = 3.6 Hz, 1 H), 4.61-4.56 (m, 3 H), 4.39 (td, *J* = 6.1, 3.6 Hz, 1 H), 4.35 (d, *J* = 11.3 Hz, 1 H), 4.29-4.28 (m, 1 H), 4.25-4.22 (m, 1 H), 4.09 (d, *J* = 3.1 Hz, 1 H), 4.07-4.04 (m, 2 H), 3.86 (dd, *J* = 8.4,

5.7 Hz, 1 H), 3.83 (dd, J = 9.8, 6.3 Hz, 1 H), 3.68 (dd, J = 9.8, 5.9 Hz, 1 H), 2.86 (dd, J = 18.9, 10.5 Hz, 1 H), 2.69-2.65 (m, 2 H), 2.04 (s, 3 H), 1.41 (s, 3 H), 1.36 (s, 3 H), 1.32 (s, 3 H), 1.25 (s, 3 H) ppm; ^{13}C NMR (176 MHz, Acetone-d_6) δ 207.08, 139.59, 139.49, 129.18, 129.15, 128.72, 128.59, 128.46, 128.34, 112.15, 109.97, 109.51, 106.31, 85.06, 82.37, 82.08, 81.26, 80.68, 74.90, 73.88, 73.51, 69.32, 67.98, 46.89, 40.15, 30.03, 27.27, 27.12, 26.58, 25.73 ppm; Gated ^{13}C (176 MHz, Acetone-d_6) δ 109.97 (d, J = 171.9 Hz) ppm. MS (ESI): 635.2 (M+Na). $[\alpha]_D^{20}$ = +32.0 (c = 0.67, CH_2Cl_2). The spectra data is in accordance to the literature.[5]

5-*O*-(3,5-*O*-di-benzyl-2-*C*-acetylmethyl-2-deoxy-α-D-lyxofuranosyl)-1,2-*O*-isopropylidene-α-D-xylofuranoside

The title product compound is prepared using **Method A** and isolated by flash column chromatography (Ethyl Acetate) giving a white solid (77 mg, 0.14 mmol, 71% yield, α only).

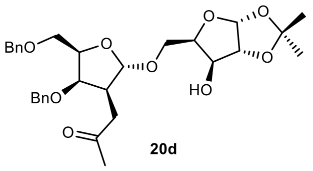

20d

^{1}H NMR (400 MHz, Acetone-d_6) δ 7.38-7.26 (m, 10 H), 5.84 (d, J = 3.7 Hz, 1 H), 4.86 (d, J = 4.3 Hz, 1 H), 4.58-4.53 (m, 3 H), 4.48 (d, J = 3.7 Hz, 1 H), 4.36-4.32 (m, 2 H), 4.30-4.27 (m, 2 H), 4.25-4.21 (m, 1 H), 4.13 (s, 1 H), 3.84-3.78 (m, 2 H), 3.71-3.63 (m, 2 H), 2.87-2.81 (m, 1 H), 2.69-2.63 (m, 1 H), 2.58 (dd, J = 17.2, 5.3 Hz, 1 H), 2.04 (s, 3 H), 1.40 (s, 3 H), 1.26 (s, 3 H) ppm; ^{13}C NMR (176 MHz, Acetone-d_6) δ 207.56, 139.62, 139.55, 129.17, 129.15, 128.71, 128.62, 128.43, 128.33, 111.74, 108.78, 105.87, 86.45, 80.89, 80.79, 80.10, 75.44, 74.86, 73.87, 69.42, 66.54, 46.56, 40.41, 30.30, 27.24, 26.55 ppm. MS (ESI): 565.2 (M+Na); HRMS (ESI): calculated for $C_{30}H_{38}O_9Na$ (M+Na): 565.24080, Found: 565.24014. $[\alpha]_D^{20}$ = +44.0 (c = 0.17, CH_2Cl_2). M.P.: 96-97 ℃.

1-*O*-(3,5-*O*-di-benzyl-2-*C*-acetylmethyl-2-deoxy-α-L-lyxofuranosyl)-4,5-*O*-di-benzyl-2-*C*-acetylmethyl-2-deoxy-α-D-lyxofuranoside

The title product compound is prepared using **Method A** with a modification that glycosyl acceptor (0.2 mmol, 1.0 equiv.) and glycosyl donor (0.3 mmol, 1.5 equiv.) is used and isolated by flash column chromatography (9:1-3:1 Pentane: Ethyl Acetate) giving a pale yellow syrup (44 mg, 0.06 mmol, 30% yield, α only)

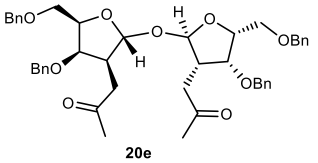

20e

^{1}H NMR (700 MHz, Acetone-d_6) δ 7.36-7.26 (m, 20 H), 5.07 (d, J = 4.5 Hz, 2 H), 4.58-4.54 (m, 6 H), 4.34-4.32 (m, 4 H), 4.29-4.27 (m, 2 H), 3.79 (dd, J = 9.8, 6.3 Hz, 2 H), 3.64 (dd, J = 9.8, 5.9 Hz, 2 H), 2.81 (dd, J = 17.7, 9.6 Hz, 2 H), 2.66-2.63 (m, 2 H), 2.58 (dd, J = 17.7, 4.9 Hz, 2 H), 2.00 (s, 6 H) ppm; ^{13}C NMR (176 MHz, Acetone-d_6) δ 207.28, 139.60, 139.49, 129.17, 129.15, 128.67, 128.59, 128.42, 128.32, 104.85, 80.99, 80.80, 74.84, 73.83, 69.42, 46.53, 40.08, 30.03 ppm; ^{13}C NMR (176 MHz, Acetone-d_6) δ 104.85 (d, J = 168.8 Hz) ppm. MS (ESI): 749.2 (M+Na); HRMS (ESI): calculated for $C_{44}H_{50}O_9Na$ (M+Na): 745.33470, Found: 745.33457. $[\alpha]_D^{20}$ = -61.7 (c = 0.40, CH_2Cl_2).

6-*O*-(3,5-*O*-di-benzyl-2-*C*-acetylmethyl-2-deoxy-α-L-lyxofuranosyl)-2,3:4,5-*O*-di-isopropylidene-β-D-fructopyranoside

The title product compound is prepared using **Method A** and isolated by flash column chromatography (9:1-3:1 Pentane: Ethyl Acetate) giving a pale yellow syrup (93 mg, 0.15 mmol, 76% yield, α only)

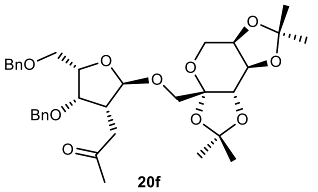

20f

^{1}H NMR (700 MHz, Acetone-d_6) δ 7.38-7.26 (m, 10 H), 4.88 (d, *J* = 3.6 Hz, 1 H), 4.61-4.55 (m, 4 H), 4.37-4.33 (m, 4 H), 4.22 (d, *J* = 8.4 Hz, 1 H), 3.86 (dd, *J* = 13.0, 1.9 Hz, 1 H), 3.83 (dd, *J* = 10.0, 5.9 Hz, 1 H), 3.73 (d, *J* = 10.6 Hz, 1 H), 3.65 (dd, *J* = 10.0, 5.6 Hz, 1 H), 3.57 (d, *J* = 12.9 Hz, 1 H), 3.49 (d, *J* = 10.7 Hz, 1 H), 2.86 (dd, *J* = 17.7, 9.7 Hz, 1 H), 2.78-2.75 (m, 1 H), 2.56 (dd, *J* = 17.7, 4.9 Hz, 1 H), 2.02 (s, 3 H), 1.46 (s, 3 H), 1.36 (s, 3 H), 1.35 (s, 3 H), 1.28 (s, 3 H) ppm; ^{13}C NMR (176 MHz, Acetone-d_6) δ 207.09, 139.72, 139.52, 129.18, 129.14, 128.69, 128.57, 128.44, 128.31, 109.40, 108.90, 108.53, 103.44, 80.67, 80.61, 74.85, 73.82, 71.93, 71.17, 70.97, 69.72, 69.50, 61.73, 46.30, 40.40, 30.14, 26.93, 26.38, 26.02, 24.45 ppm; Gated ^{13}C (176 MHz, Acetone-d_6) δ 108.52 (d, *J* = 167.8 Hz) ppm. MS (ESI): 635.2 (M+Na); HRMS (ESI): calculated for $C_{34}H_{44}O_{10}Na$ (M+Na): 635.28267, Found: 635.28230. $[\alpha]_D^{20}$ = -56.4 (c = 0.21, CH_2Cl_2).

6-*O*-(3,5-*O*-di-benzyl-2-*C*-acetylmethyl-2-deoxy-α-L-lyxofuranosyl)-1,2:3,4-di-*O*-isopropylidene-α-D-galactopyranoside

The title product compound is prepared using **Method A** and isolated by flash column chromatography (9:1-3:1 Pentane: Ethyl Acetate) giving a pale yellow syrup (85 mg, 0.14 mmol, 69% yield, α/β ratio 83:17).

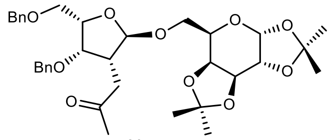

20g

^{1}H NMR (700 MHz, Acetone-d_6) δ 7.38-7.27 (m, 10 H), 5.47 (d, *J* = 4.9 Hz, 1 H), 4.91 (d, *J* = 4.3 Hz, 1 H), 4.59-4.58 (m, 1 H), 4.57-4.55 (m, 3 H), 4.36-4.33 (m, 2 H), 4.32-4.31 (m, 1 H), 4.27-4.24 (m, 2 H), 3.94-3.92 (m, 1 H), 3.80-3.78 (m, 2 H), 3.64 (dd, *J* = 9.7, 5.7 Hz, 1 H), 3.54 (dd, *J* = 11.1, 7.2 Hz, 1 H), 2.86-2.83 (m, 1 H), 2.66-2.61 (m, 2 H), 2.04 (s, 3 H), 1.46 (s, 3 H), 1.35 (s, 3 H), 1.31 (s, 3 H), 1.29 (s, 3 H) ppm; ^{13}C NMR (176 MHz, Acetone-d_6) δ 207.34, 139.65, 139.60, 129.18, 129.16, 128.72, 128.61, 128.42, 128.33, 109.63, 109.00, 108.87, 97.24, 80.84, 80.76, 74.86, 73.86, 72.15, 71.67, 71.43, 69.40, 68.69, 68.25, 46.65, 40.31, 30.14, 26.47, 26.45, 25.25, 24.70 ppm; Gated ^{13}C (176 MHz, Acetone-d_6) δ 108.87 (d, *J* = 169.4 Hz) ppm. MS (ESI): 635.2 (M+Na); HRMS (ESI): calculated for $C_{34}H_{44}O_{10}Na$ (M+Na): 635.28267, Found: 635.28231. $[\alpha]_D^{20}$ = -65.9 (c = 0.44, CH_2Cl_2).

1-*O*-methyl-5-*O*-(3,5-*O*-di-benzyl-2-*C*-acetylmethyl-2-deoxy-α-L-lyxofuranosyl)-2,3-*O*-isopropylidene-β-D-ribofuranoside

The title product compound is prepared using **Method A** and isolated by flash column chromatography (9:1-3:1 Pentane: Ethyl Acetate) giving a pale yellow syrup (84 mg, 0.15 mmol, 73% yield, α/β ratio 75:25).

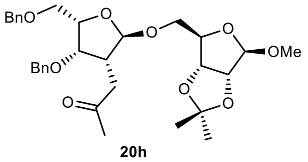

20h

^{1}H NMR (700 MHz, Acetone-d_6) δ 7.37 (d, *J* = 7.3 Hz, 2 H), 7.35-7.32 (m, 4 H), 7.29-7.28 (m, 4 H), 4.88 (d, *J* = 3.5 Hz, 1 H), 4.86 (d, *J* = 4.2 Hz, 1 H), 4.70 (d, *J* = 5.9 Hz, 1 H), 4.60-4.55 (m, 4 H), 4.37-4.34 (m, 2 H), 4.31-4.30 (m, 1 H), 4.21-4.19 (m, 1 H), 3.81 (dd, *J* = 9.8, 6.3 Hz, 1 H), 3.67-3.63 (m, 2 H), 3.44 (dd, *J* = 9.8, 6.3 Hz, 1 H), 3.27 (s, 3 H), 2.85 (dd, *J* = 17.7, 9.5 Hz, 1 H), 2.70-2.68 (m, 1 H), 2.58 (dd, *J* = 17.7, 5.1 Hz, 1 H), 2.04 (s, 3 H), 1.40 (s, 3 H), 1.26 (s, 3 H) ppm; ^{13}C NMR (176 MHz, Acetone-d_6) δ 207.21, 139.64, 139.55, 129.19, 129.18, 128.71, 128.61, 128.46, 128.36, 112.68, 110.17, 108.46, 86.11, 86.07, 83.10, 80.90, 80.87, 74.88, 73.89, 69.93, 69.48, 54.91, 46.60, 40.35, 30.03, 26.87, 25.21

ppm. MS (ESI): 579.2 (M+Na); HRMS (ESI): calculated for $C_{31}H_{40}O_9Na$ (M+Na): 579.25645, Found: 579.25580. $[\alpha]_D^{20}$ = -74.6 (c = 0.31, CH_2Cl_2).

3-*O*-(3,5-*O*-di-benzyl-2-*C*-acetylmethyl-2-deoxy-α-L-lyxofuranosyl)-1,2:5,6-*O*-di-isopropylidene-α-D-glucofuranoside

The title product compound is prepared using **Method B** and isolated by flash column chromatography (9:1-3:1 Pentane: Ethyl Acetate) giving a pale yellow syrup (92 mg, 0.15 mmol, 75% yield, α only).

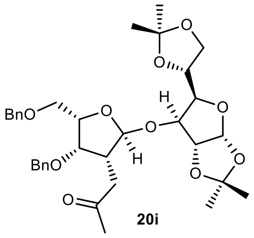

20i

^{1}H NMR (500 MHz, Acetone-d_6) δ 7.38-7.28 (m, 10 H), 5.85 (d, *J* = 3.7 Hz, 1 H), 5.02 (d, *J* = 3.2 Hz, 1 H), 4.62 (d, *J* = 3.8 Hz, 1 H), 4.61-4.54 (m, 3 H), 4.47-4.43 (m, 1 H), 4.38-4.33 (m, 2 H), 4.29-4.25 (m, 1 H), 4.23-4.21 (m, 1 H), 4.18-4.15 (m, 1 H), 4.01 (dd, *J* = 8.3, 6.4 Hz, 1 H), 3.84 (ddd, *J* = 19.5, 9.1, 6.2 Hz, 2 H), 3.66 (dd, *J* = 9.9, 5.8 Hz, 1 H), 2.85 (dd, *J* = 17.6, 8.7 Hz, 1 H), 2.74-2.69 (m, 1 H), 2.59 (dd, *J* = 17.7, 6.0 Hz, 1 H), 2.03 (s, 3 H), 1.41 (s, 3 H), 1.31 (s, 3 H), 1.26 (s, 6 H) ppm; ^{13}C NMR (126 MHz, Acetone-d_6) δ 207.28, 139.64, 139.46, 129.17, 129.14, 128.68, 128.56, 128.45, 128.32, 112.10, 109.14, 106.26, 105.58, 83.10, 81.59, 80.59, 80.57, 78.43, 74.77, 73.77, 73.75, 69.40, 67.43, 46.10, 40.37, 30.07, 27.17, 27.03, 26.52, 25.70 ppm. HRMS (ESI): calculated for $C_{34}H_{44}O_{10}Na$ (M+Na): 635.28267, Found: 635.28276. $[\alpha]_D^{20}$ = -46.8 (c = 0.59, CH_2Cl_2).

6-*O*-(3,5-*O*-di-benzyl-2-*C*-acetylmethyl-2-deoxy-β-D-ribofuranosyl)-1,2:3,4-di-*O*-isopropylidene-α-D-galactopyranoside

The title product compound is prepared using **Method A** and isolated by flash column chromatography (9:1-3:1 Pentane: Ethyl Acetate) giving a pale yellow syrup (87 mg, 0.14 mmol, 71% yield, β/α ratio 91:9)

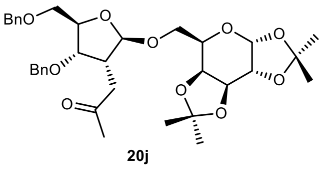

20j

^{1}H NMR (500 MHz, Acetone-d_6) δ 7.39-7.26 (m, 10 H), 5.45 (d, *J* = 5.0 Hz, 1 H), 4.89 (d, *J* = 3.1 Hz, 1 H), 4.59-4.57 (m, 3 H), 4.50 (d, *J* = 11.7 Hz, 1 H), 4.40 (d, *J* = 11.7 Hz, 1 H), 4.32 (dd, *J* = 5.0, 2.4 Hz, 1 H), 4.20 (dd, *J* = 7.9, 1.8 Hz, 1 H), 4.16-4.14 (m, 2 H), 3.90-3.88 (m, 1 H), 3.80 (dd, *J* = 10.4, 5.3 Hz, 1 H), 3.60-3.57 (m, 2 H), 3.46 (dd, *J* = 10.5, 6.8 Hz, 1 H), 2.89 (dd, *J* = 17.5, 8.4 Hz, 1 H), 2.81-2.75 (m, 1 H), 2.50 (dd, *J* = 17.4, 6.0 Hz, 1 H), 2.09 (s, 3 H), 1.46 (s, 3 H), 1.35 (s, 3 H), 1.31 (s, 3 H), 1.29 (s, 3 H) ppm; ^{13}C NMR (126 MHz, Acetone-d_6) δ 207.05, 139.84, 139.39, 129.16, 129.13, 128.73, 128.42, 128.34, 128.26, 109.57, 109.32, 108.99, 97.22, 82.51, 81.39, 73.68, 72.99, 72.65, 71.98, 71.58, 71.42, 68.13, 67.53, 44.15, 39.66, 30.38, 26.47, 26.45, 25.21, 24.70 ppm. MS (ESI): 635.2 (M+Na); HRMS (ESI): calculated for $C_{34}H_{44}O_{10}Na$ (M+Na): 635.28267, Found: 635.28228. $[\alpha]_D^{20}$ = -54.3 (c = 0.13, CH_2Cl_2).

6-*O*-(3,5-*O*-di-benzyl-2-*C*-acetylmethyl-2-deoxy-β-D-ribofuranosyl)-1,2:5,6-*O*-di-isopropylidene-α-D-glucofuranoside

The title product compound is prepared using **Method A** with a modification that 0.5 mol% catalyst **A** is used instead and isolated by flash column chromatography (9:1-3:1 Pentane: Ethyl Acetate) giving a pale yellow syrup (62 mg, 0.10 mmol, 50% yield)

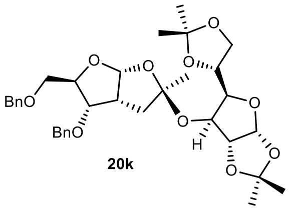

20k

^{1}H NMR (400 MHz, Acetone-d_6) δ 7.36-7.26 (m, 10 H), 5.89 (d, *J* = 5.5 Hz, 1 H), 5.81 (d, *J* = 3.8 Hz, 1 H), 4.60 (d, *J* = 3.8 Hz, 1 H), 4.59-4.50 (m, 3 H), 4.47 (d, *J* = 11.6 Hz, 1 H), 4.27 (d, *J* = 3.3 Hz, 1 H), 4.25-4.20 (m, 1 H), 4.09-4.06 (m, 1 H), 4.06-4.03 (m, 1 H), 4.01 (d, *J* = 8.4 Hz, 1 H), 3.98-3.91 (m, 1 H), 3.87 (dd, *J* = 8.3, 6.1 Hz, 1 H), 3.73 (dd, *J* = 11.1, 1.9 Hz, 1 H), 3.57 (dd, *J* = 11.1, 5.1 Hz, 1 H), 3.35-3.29 (m, 1 H), 2.21 (dd, *J* = 13.6, 8.6 Hz, 1 H), 2.12-2.08 (m, 1 H), 1.58 (s, 3 H), 1.43 (s, 3 H), 1.36 (s, 3 H), 1.29 (s, 3 H), 1.27 (s, 3 H) ppm; ^{13}C NMR (101 MHz, Acetone) δ 139.78, 139.25, 129.22, 129.12, 128.75, 128.58, 128.44, 128.26, 112.02, 110.80, 109.68, 109.65, 105.97, 85.90, 82.07, 79.35, 79.32, 75.00, 73.78, 73.02, 72.66, 70.58, 68.06, 44.75, 37.34, 27.19, 27.17, 26.53, 25.87, 24.49 ppm. MS (ESI): 635.2 (M+Na); HRMS (ESI): calculated for $C_{34}H_{44}O_{10}Na$ (M+Na): 635.28267, Found: 635.28208. $[\alpha]_D^{20}$ = +12.3 (c = 0.13, CH_2Cl_2).

O-(3,5-O-di-benzyl-2-C-acetylmethyl-2-deoxy-α-D-lyxofuranosyl)-N-tertbutyloxycarbonyl-L-serine methyl ester

The title product compound is prepared using **Method A** and isolated by flash column chromatography (9:1-4:1 Pentane: Ethyl Acetate) giving a pale yellow syrup (92 mg, 0.16 mmol, 80% yield, α/β ratio 93:7)

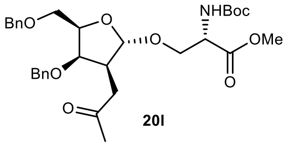

20l

^{1}H NMR (700 MHz, Acetone-d_6) δ 7.39-7.38 (m, 10 H), 6.26 (d, *J* = 8.5 Hz, 1 H), 4.81 (d, *J* = 4.4 Hz, 1 H), 4.61-4.55 (m, 3 H), 4.39-4.32 (m, 3 H), 4.28-4.26 (m, 1 H), 3.88 (qd, *J* = 10.6, 4.0 Hz, 2 H), 3.80 (dd, *J* = 9.7, 6.7 Hz, 1 H), 3.69 (s, 3 H), 3.66 (dd, *J* = 9.7, 5.8 Hz, 1 H), 2.85-2.81 (m, 1 H), 2.66-2.62 (m, 1 H), 2.54 (dd, *J* = 17.6, 5.2 Hz, 1 H), 2.02 (s, 3 H), 1.40 (s, 9 H) ppm; ^{13}C NMR (176 MHz, Acetone) δ 207.19, 171.72, 156.35, 139.57, 139.50, 129.17, 129.16, 128.69, 128.62, 128.44, 128.35, 108.97, 80.90, 80.72, 79.47, 74.86, 73.89, 69.61, 69.19, 55.22, 52.43, 46.62, 40.31, 30.03, 28.62 ppm. MS (ESI): 594.2 (M+Na). $[\alpha]_D^{20}$ = +32.9 (c = 0.62, CH_2Cl_2).

O-(3,5-O-di-benzyl-2-C-acetylmethyl-2-deoxy-α-D-lyxofuranosyl)-N-tertbutyloxycarbonyl-L-threonine methyl ester

The title product compound is prepared using **Method A** with a modification that 0.5 mol% catalyst **A** is used instead and isolated by flash column chromatography (9:1-4:1 Pentane: Ethyl Acetate) giving a pale yellow syrup (97 mg, 0.17 mmol, 83% yield, α/β ratio 94:6)

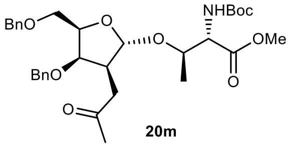

20m

^{1}H NMR (700 MHz, Acetone-d_6) δ 7.38-7.26 (m, 10 H), 5.87 (d, *J* = 9.5 Hz, 1 H), 4.81 (d, *J* = 4.7 Hz, 1 H), 4.58-4.54 (m, 3 H), 4.33-4.31 (m, 2 H), 4.24-4.21 (m, 3 H), 3.79 (dd, *J* = 9.8, 6.5 Hz, 1 H), 3.72 (s, 3 H), 3.64 (dd, *J* = 9.8, 5.9 Hz, 1 H), 2.82-2.78 (m, 1 H), 2.59-2.56 (m, 1 H), 2.47 (dd, *J* = 17.8, 5.1 Hz, 1 H), 2.03 (s, 3 H), 1.42 (s, 9 H), 1.25 (d, *J* = 6.3 Hz, 3 H) ppm; ^{13}C NMR (176 MHz, Acetone-d_6) δ 207.24, 172.13, 156.77, 139.62, 139.53, 129.17, 129.15, 128.69, 128.56, 128.44, 128.33, 109.20, 80.99, 80.63, 79.53, 75.95, 74.85, 73.84, 69.34, 59.22, 52.49, 46.88, 40.19, 30.03, 28.59, 19.21 ppm. MS (ESI): 608.2 (M+Na); HRMS (ESI): calculated for $C_{32}H_{43}O_9NNa$ (M+Na): 608.28300, Found: 608.28269. $[\alpha]_D^{20}$ = +49.4 (c = 0.32, CH_2Cl_2);

4-Bromophenylmethyl 3,5-*O*-di-benzyl-2-*C*-acetylmethyl-α-L-xylofuranoside

The title product compound is prepared using **Method A** and isolated by flash column chromatography (9:1 Pentane: Ethyl Acetate) giving a white solid (100 mg, 0.19 mmol, 92% yield, α/β ratio 86:14)

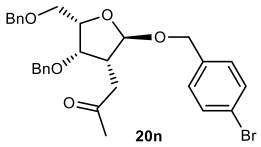

20n

^{1}H NMR (700 MHz, Acetone-d$_6$) δ 7.50 (d, *J* = 8.4 Hz, 2 H), 7.38-7.27 (m, 12 H), 4.93 (d, *J* = 4.2 Hz, 1 H), 4.68 (d, *J* = 12.6 Hz, 1 H), 4.60-4.55 (m, 3 H), 4.47 (d, *J* = 11.9 Hz, 1 H), 4.40-4.38 (m, 1 H), 4.35 (d, *J* = 11.2 Hz, 1 H), 4.33- 4.32 (m, 1 H), 3.83 (dd, *J* = 9.8, 6.3 Hz, 1 H), 3.68 (dd, *J* = 9.8, 5.9 Hz, 1 H), 2.84 (dd, *J* = 17.6, 9.4 Hz, 1 H), 2.77-2.74 (m, 1 H), 2.58 (dd, *J* = 17.6, 5.2 Hz, 1 H), 2.02 (s, 3 H) ppm; ^{13}C NMR (176 MHz, Acetone-d$_6$) δ 207.16, 139.58, 139.48, 138.99, 132.10, 130.42, 129.15, 129.13, 128.67, 128.56, 128.42, 128.31, 121.55, 107.87, 80.92, 80.88, 74.84, 73.85, 69.57, 69.44, 46.58, 40.30, 30.03 ppm; Gated ^{13}C NMR (176 MHz, Acetone-d$_6$) δ 107.86 (d, *J* = 186.9 Hz). MS (ESI): 561.0 (M+Na); HRMS (ESI): Calculated for $C_{29}H_{31}O_5BrNa$ (M+Na): 561.12471, Found: 561.12408. $[\alpha]_D^{20}$ = -63.8 (c = 0.47, CH_2Cl_2). M.P.: 62-63 °C.

Succinimidyl-4,5-*O*-di-benzyl-2-*C*-acetylmethyl-α-D-lyxofuranoside

The title product compound is prepared using **Method A** and isolated by flash column chromatography (1:1 Pentane: Ethyl Acetate) giving a pale yellow syrup (54 mg, 0.12 mmol, 58% yield, α/β ratio 97:3)

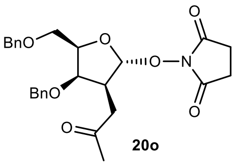

20o

^{1}H NMR (500 MHz, Acetone-*d*$_6$) δ 7.39-7.26 (m, 10 H), 5.31 (d, *J* = 2.0 Hz, 1 H), 4.70-4.66 (m, 1 H), 4.60-4.53 (m, 3 H), 4.43 (dd, *J* = 6.0, 4.6 Hz, 1 H), 4.34 (d, *J* = 11.1 Hz, 1 H), 3.81 (dd, *J* = 10.1, 6.4 Hz, 1 H), 3.64 (dd, *J* = 10.1, 5.8 Hz, 1 H), 2.99-2.95 (m, 1 H), 2.90 (dd, *J* = 17.7, 9.6 Hz, 1 H), 2.72-2.67 (m, 5 H), 2.04 (s, 3 H) ppm; ^{13}C NMR (126 MHz, Acetone-*d*$_6$) δ 206.67, 172.42, 139.50, 139.29, 129.19, 129.14, 128.68 (two carbons), 128.49, 128.35, 111.42, 81.94, 80.06, 74.96, 73.66, 68.71, 44.84, 40.14, 30.01, 26.24 ppm. MS (ESI): 490.0 (M+Na), 957.4 (2M+Na); HRMS (ESI): calculated for $C_{26}H_{29}O_7NNa$ (M+Na): 490.18362, Found: 490.18297. $[\alpha]_D^{20}$ = +96.0 (c = 0.48, CH_2Cl_2).

Cholesteryl 3,5-*O*-di-benzyl-2-*C*-acetylmethyl-2-deoxy-α-L-lyxofuranoside

The title product compound is prepared using **Method A** and isolated by flash column chromatography (9:1-3:1 Pentane: Ethyl Acetate) giving a white solid (111.6 mg, 0.15 mmol, 75% yield, α/β ratio 83:17)

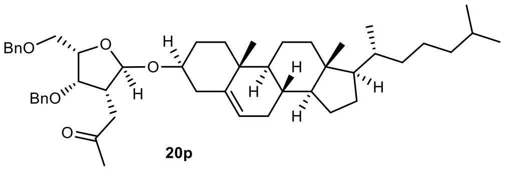

20p

^{1}H NMR (700 MHz, CD_2Cl_2) δ 7.25-7.32 (m, 6 H), 7.30-7.28 (m, 2 H), 7.24 (d, *J* = 7.7 Hz, 2 H), 5.34 (d, *J* = 4.2 Hz, 1 H), 4.96 (d, *J* = 4.9 Hz, 1 H), 4.56 (d, *J* = 11.9 Hz, 1 H), 4.53-4.51 (m, 2 H), 4.35-4.34 (m, 1 H), 4.31 (d, *J* = 11.2 Hz, 1 H), 4.28-4.27 (m, 1 H), 3.73-3.71 (m, 1 H), 3.64-3.62 (m, 1 H), 3.41-3.39 (m, 1 H), 2.80-2.76 (m, 1 H), 2.60-2.58 (m, 1 H), 2.50 (dd, *J* = 17.7, 4.9 Hz, 1 H), 2.32-2.30 (m, 1 H), 2.17-2.13 (m, 1 H), 2.04-2.01 (m, 4 H), 1.98-1.96 (m, 1 H), 1.89-1.93 (m, 3 H), 1.61-1.41 (m, 7 H), 1.40-1.36 (m, 3 H), 1.30-1.26

(m, 1 H), 1.19-0.98 (m, 12 H), 0.96-0.93 (m, 4 H), 0.89-0.87 (m, 6 H), 0.70 (s, 3 H) ppm; ^{13}C NMR (176 MHz, CD_2Cl_2) δ 207.58, 141.35, 138.95, 138.92, 128.89, 128.87, 128.43, 128.35, 128.24, 128.13, 122.15, 106.07, 80.43, 80.20, 78.08, 74.75, 73.93, 69.40, 57.37, 56.78, 50.81, 46.29, 42.89, 40.41, 40.20, 40.09, 39.41, 37.89, 37.32, 36.79, 36.40, 32.54, 32.49, 30.36, 28.80, 28.61, 24.84, 24.40, 23.16, 22.92, 21.64, 19.77, 19.11, 12.22 ppm. HRMS (ESI): Calculated for $C_{49}H_{70}O_5Na$ (M+Na): 761.51155, Found: 761.51204. $[\alpha]_D^{20}$ = -62.3 (c = 0.62, CH_2Cl_2). M.P.: 146-148 °C.

Cholesteryl 3,5-*O*-di-benzyl-2-*C*-acetylmethyl-2-deoxy-α-D-lyxofuranoside

The title product compound is prepared using **Method A** and isolated by flash column chromatography (9:1-4:1 Pentane: Ethyl Acetate) giving a pale yellow syrup (106 mg, 0.14 mmol, 72% yield, α/β ratio 92:8)

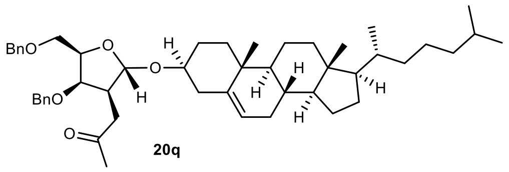

20q

^{1}H NMR (700 MHz, Acetone-d_6) δ 7.37 (d, *J* = 7.6 Hz, 2 H), 7.35-7.31 (m, 4 H), 7.28-7.26 (m, 4 H), 5.33 (s, 1 H), 4.96 (d, *J* = 4.2 Hz, 1 H), 4.60-4.54 (m, 3 H), 4.35-4.33 (m, 2 H), 4.27-4.26 (m, 1 H), 3.82-3.78 (m, 1 H), 3.66-3.63 (m, 1 H), 3.42-3.37 (m, 1 H), 2.83 (dd, *J* = 17.5, 9.5 Hz, 1 H), 2.61-2.60 (m, 1 H), 2.53 (ddd, *J* = 17.5, 5.3, 2.5 Hz, 1 H), 2.38-2.37 (m, 1 H), 2.25-2.24 (m, 1 H), 2.08-2.06 (m, 1 H), 2.03 (s, 3 H), 1.99-1.95 (m, 1 H), 1.87-1.84 (m, 3 H), 1.62-1.60 (m, 1 H), 1.58-1.54 (m, 3 H), 1.52-1.46 (m, 2 H), 1.45-1.37 (m, 4 H), 1.32-1.28 (m, 1 H), 1.23-1.10 (m, 6 H), 1.07-1.02 (m, 6 H), 0.98-0.94 (m, 4 H), 0.90-0.87 (m, 6 H), 0.73-0.72 (m, 3 H) ppm; ^{13}C NMR (176 MHz, Acetone-d_6) δ 207.19, 141.87, 139.65, 139.59, 129.15, 129.13, 128.68, 128.54, 128.40, 128.30, 122.18, 106.62, 80.87, 80.64, 78.22, 74.84, 73.86, 69.53, 57.69, 57.14, 51.19, 46.91, 43.18, 41.43, 40.74, 40.37, 38.09, 37.49, 37.08, 36.71, 32.81, 32.72, 30.03, 29.09, 29.04, 28.77, 25.04, 24.67, 23.25, 22.99, 21.88, 19.88, 19.30, 12.41 ppm. HRMS (ESI): Calculated for $C_{49}H_{70}O_5Na$ (M+Na): 761.51155, Found: 761.51198. $[\alpha]_D^{20}$ = +24.9 (c = 0.40, CH_2Cl_2).

Testosteronyl 3,5-*O*-di-benzyl-2-*C*-acetylmethyl-2-deoxy-α-D-lyxofuranoside

The title product compound is prepared using **Method A** and isolated by flash column chromatography (9:1-3:1 Pentane: Ethyl Acetate) giving a pale yellow syrup (65 mg, 0.10 mmol, 51% yield, α/β ratio 96:4)

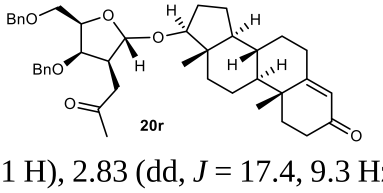

20r

^{1}H NMR (500 MHz, Acetone-d_6) δ 7.38-7.32 (m, 6 H), 7.32-7.27 (m, 4 H), 5.61 (s, 1 H), 4.80 (d, *J* = 4.3 Hz, 1 H), 4.62-4.54 (m, 3 H), 4.35-4.32 (m, 2 H), 4.28-4.26 (m, 1 H), 3.81 (dd, *J* = 9.9, 6.1 Hz, 1 H), 3.65 (dd, *J* = 9.8, 6.0 Hz, 1 H), 3.54 (t, *J* = 8.5 Hz, 1 H), 2.83 (dd, *J* = 17.4, 9.3 Hz, 1 H), 2.66-2.60 (m, 1 H), 2.54 (dd, *J* = 17.4, 5.3 Hz, 1 H), 2.48-2.43 (m, 1 H), 2.41-2.35 (m, 1 H), 2.27-2.23 (m, 2 H), 2.21-2.16 (m, 1 H), 2.04 (s, 3 H), 2.03-1.97 (m, 2 H), 1.86-1.81 (m, 1 H), 1.69-1.53 (m, 4 H), 1.49-1.38 (m, 2 H), 1.36-1.26 (m, 3 H), 1.22 (s, 3 H), 1.10-1.06 (m, 1 H), 1.05-0.94 (m, 1 H), 0.78 (s, 3 H) ppm; ^{13}C NMR (126 MHz, Acetone) δ 207.40, 198.33, 171.16, 139.73, 139.57, 129.17, 129.14, 128.71, 128.48, 128.43, 128.29, 124.29, 107.43, 87.39, 81.00, 80.60, 74.84, 73.76, 69.59, 55.04, 51.55, 46.95, 43.14, 40.52, 39.43, 37.52, 36.69, 36.32, 34.59, 33.29, 32.53, 30.07, 28.35, 23.87, 21.41, 17.70, 12.23

ppm. MS (ESI): 663.2 (M+Na); HRMS (ESI): Calculated for $C_{41}H_{52}O_6Na$ (M+Na): 663.36561, Found: 663.36537. $[\alpha]_D^{20}$ = +90.8 (c = 0.18, CH_2Cl_2).

Cholesteryl 3,5-*O*-di-benzyl-2-*C*-acetylmethyl-2-deoxy-β-D-ribofuranoside

The title product compound is prepared using **Method A** and isolated by flash column chromatography (9:1-3:1 Pentane: Ethyl Acetate) giving a pale yellow syrup (82 mg, 0.11 mmol, 55% yield, β/α ratio 94:6)

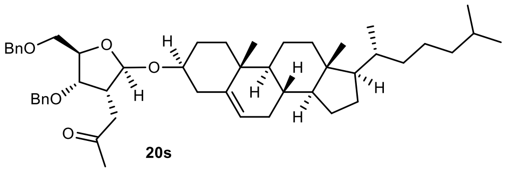

20s

^{1}H NMR (500 MHz, Acetone-d_6) δ 7.39-7.26 (m, 10 H), 5.35 (d, J = 5.0 Hz, 1 H), 4.98 (d, J = 3.5 Hz, 1 H), 4.62-4.55 (m, 2 H), 4.51 (d, J = 11.7 Hz, 1 H), 4.39 (d, J = 11.7 Hz, 1 H), 4.14 (d, J = 3.8 Hz, 2 H), 3.55 (d, J = 5.4 Hz, 2 H), 3.48-3.37 (m, 1 H), 2.87 (dd, J = 17.5, 8.2 Hz, 1 H), 2.72-2.67 (m, 1 H), 2.45 (dd, J = 17.6, 6.3 Hz, 1 H), 2.36-2.32 (m, 1 H), 2.15-2.12 (m, 1 H), 2.09 (s, 3 H), 2.07-2.03 (m, 2 H), 1.99-1.95 (m, 1 H), 1.89-1.80 (m, 3 H), 1.62-1.50 (m, 4 H), 1.50-1.43 (m, 3 H), 1.41-1.37 (m, 2 H), 1.32-1.27 (m, 2 H), 1.20-1.09 (m, 5 H), 1.06-1.02 (m, 3 H), 1.00 (s, 3 H), 0.97-0.95 (m, 4 H), 0.89 (s, 3 H), 0.87 (s, 3 H), 0.72 (s, 3 H) ppm; ^{13}C NMR (126 MHz, Acetone-d_6) δ 207.00, 141.61, 139.71, 139.39, 129.12, 129.10, 128.65, 128.39 (two carbons), 128.25, 122.26, 107.19, 82.27, 81.41, 77.82, 73.68, 73.05, 72.56, 57.65, 57.08, 51.16, 44.39, 43.14, 40.70, 40.32, 39.68, 39.58, 38.17, 37.50, 37.04, 36.69, 32.77, 32.70, 30.63, 30.29, 29.02, 28.76, 25.01, 24.64, 23.23, 22.98, 21.84, 19.85, 19.26, 12.35 ppm. HRMS (ESI): Calculated for $C_{49}H_{70}O_5Na$ (M+Na): 761.51155, Found: 761.51190. $[\alpha]_D^{20}$ = -38.7 (c = 0.51, CH_2Cl_2).

Allyl 3,5-*O*-di-benzyl-2-*C*-acetylmethyl-2-deoxy-α-D-lyxofuranoside

The title product compound is prepared using **Method A** and isolated by flash column chromatography (9:1-3:1 Pentane: Ethyl Acetate) giving a pale yellow syrup (64 mg, 0.15 mmol, 77% yield, α/β ratio 89:11)

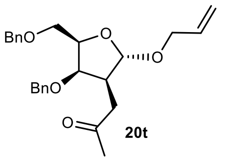

20t

^{1}H NMR (700 MHz, Acetone-d_6) δ 7.38-7.37 (m, 2 H), 7.36-7.32 (m, 4 H), 7.29-7.27 (m, 4 H), 5.90 (ddt, J = 17.1, 10.6, 5.4 Hz, 1 H), 5.24 (dq, J = 17.3, 1.9 Hz, 1 H), 5.10 (dq, J = 10.5, 1.6 Hz, 1 H), 4.85 (d, J = 4.5 Hz, 1 H), 4.61-4.53 (m, 3 H), 4.36-4.32 (m, 2 H), 4.29 (dd, J = 5.7, 3.9 Hz, 1 H), 4.16 (ddt, J = 13.3, 5.1, 1.7 Hz, 1 H), 3.95 (ddt, J = 13.2, 5.7, 1.6 Hz, 1 H), 3.80 (dd, J = 9.8, 6.4 Hz, 1 H), 3.65 (dd, J = 9.8, 5.8 Hz, 1 H), 2.84 (dd, J = 17.7, 9.5 Hz, 1 H), 2.70-2.66 (m, 1 H), 2.56 (dd, J = 17.7, 5.2 Hz, 1 H), 2.03 (s, 3 H) ppm; ^{13}C NMR (176 MHz, Acetone-d_6) δ 207.23, 139.62, 139.53, 136.06, 129.16, 129.14, 128.68, 128.57, 128.42, 128.34, 116.39, 107.67, 80.86, 80.77, 74.83, 73.85, 69.45 (two carbons), 46.54, 40.32, 30.00 ppm; MS (ESI): 433.2 (M+Na); HRMS (ESI): calculated for $C_{25}H_{30}O_5Na$: 433.19855, Found: 433.19807. $[\alpha]_D^{20}$ = +57.1 (c = 0.31, CH_2Cl_2).

Propargyl 3,5-*O*-di-benzyl-2-*C*-acetylmethyl-2-deoxy-α-D-lyxofuranoside

The title product compound is prepared using **Method A** and isolated by flash column chromatography (9:1-3:1 Pentane: Ethyl Acetate) giving a pale yellow syrup (61 mg, 0.15 mmol, 75% yield, α/β ratio 88:12)

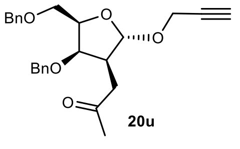

20u

^{1}H NMR (700 MHz, Acetone-d_6) δ 7.37 (d, *J* = 7.0 Hz, 2 H), 7.35-7.32 (m, 4 H), 7.29-7.27 (m, 4 H), 4.99 (d, *J* = 4.3 Hz, 1 H), 4.60-4.55 (m, 3 H), 4.36-4.32 (m, 2 H), 4.30 (dd, *J* = 5.6, 3.9 Hz, 1 H), 4.24 (dd, *J* = 15.8, 2.5 Hz, 1 H), 4.18 (dd, *J* = 15.8, 2.4 Hz, 1 H), 3.81 (dd, *J* = 9.8, 6.2 Hz, 1 H), 3.65 (dd, *J* = 9.8, 5.8 Hz, 1 H), 2.91-2.90 (m, 1 H), 2.85 (dd, *J* = 17.8, 9.7 Hz, 1 H), 2.69-2.65 (m, 1 H), 2.57 (dd, *J* = 17.8, 5.0 Hz, 1 H), 2.04 (s, 3 H) ppm; ^{13}C NMR (176 MHz, Acetone-d_6) δ 207.08, 139.62, 139.50, 129.18, 129.16, 128.71, 128.59, 128.45, 128.34, 106.54, 81.07, 80.78, 80.61, 75.70, 74.88, 73.87, 69.35, 55.32, 46.45, 40.11, 30.03 ppm; MS (ESI): 431.2 (M+Na); HRMS (ESI): calculated for $C_{25}H_{28}O_5Na$:431.18290, Found: 431.18245. $[\alpha]_D^{20}$ = +64.5 (c = 0.24, CH_2Cl_2).

2-Azido-1-ethyl 3,5-*O*-di-benzyl-2-*C*-acetylmethyl-2-deoxy-α-D-lyxofuranoside

The title product compound is prepared using **Method A** and isolated by flash column chromatography (9:1-3:1 Pentane: Ethyl Acetate) giving a pale yellow syrup (61 mg, 0.14 mmol, 69% yield, α/β ratio 87:13)

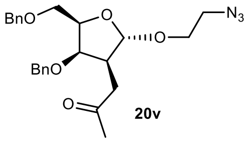

20v

^{1}H NMR (700 MHz, Acetone-d_6) δ 7.37 (d, *J* = 7.0 Hz, 2 H), 7.37-7.32 (m, 4 H), 7.29-7.27 (m, 4 H), 4.88 (d, *J* = 4.4 Hz, 1 H), 4.60-4.55 (m, 3 H), 4.39-4.37 (m, 1 H), 4.35 (d, *J* = 11.3 Hz, 1 H), 4.31-4.30 (m, 1 H), 3.85 (ddd, *J* = 10.9, 6.1, 3.5 Hz, 1 H), 3.81 (dd, *J* = 9.8, 6.4 Hz, 1 H), 3.68-3.61 (m, 2 H), 3.42 (ddd, *J* = 13.3, 7.1, 3.4 Hz, 1 H), 3.36 (ddd, *J* = 13.3, 6.1, 3.5 Hz, 1 H), 2.86 (dd, *J* = 17.7, 9.6 Hz, 1 H), 2.69-2.66 (m, 1 H), 2.59 (dd, *J* = 17.7, 5.0 Hz, 1 H), 2.04 (s, 3 H) ppm; ^{13}C NMR (176 MHz, Acetone-d_6) δ 207.22, 139.65, 139.54, 129.20, 129.18, 128.74, 128.61, 128.47, 128.36, 108.60, 80.97, 80.77, 74.90, 73.88, 69.42, 68.17, 51.50, 46.77, 40.26, 30.03 ppm. MS (ESI): 462.2(M+Na); HRMS (ESI): calculated for $C_{24}H_{29}O_5N_3Na$ (M+Na): 462.19994, Found: 462.19940. $[\alpha]_D^{20}$ = +41.3 (c = 0.53, CH_2Cl_2).

***L*-Menthyl 3,5-*O*-di-benzyl-2-*C*-acetylmethyl-2-deoxy-α-D-lyxofuranoside**

The title product compound is prepared using **Method A** and isolated by flash column chromatography (9:1-3:1 Pentane: Ethyl Acetate) giving a pale yellow syrup (72 mg, 0.14 mmol, 71% yield, α/β ratio 89:11)

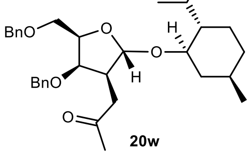

20w

^{1}H NMR (700 MHz, Acetone-d_6) δ 7.38 (d, *J* = 7.2 Hz, 2 H), 7.35-7.32 (m, 4 H), 7.39-7.26 (m, 4 H), 4.89 (d, *J* = 4.6 Hz, 1 H), 4.61-4.55 (m, 3 H), 4.37-4.34 (m, 2 H), 4.27-4.25 (m, 1 H), 3.81 (dd, *J* = 9.8, 6.4 Hz, 1 H), 3.65 (dd, *J* = 9.8, 5.9 Hz, 1 H), 3.25 (td, *J* = 10.6, 4.3 Hz, 1 H), 2.86 (dd, *J* = 17.7, 9.6 Hz, 1 H), 2.66-2.63 (m, 1 H), 2.54 (dd, *J* = 17.7, 5.1 Hz, 1 H), 2.24-2.22 (m, 1 H), 2.19-2.15 (m, 1 H), 2.03 (s, 3 H), 1.65-1.58 (m, 2 H), 1.41-1.36 (m, 1 H), 1.19-1.15 (m, 1 H), 1.03-0.92 (m, 2 H), 0.90-0.87 (m, 6 H), 0.84-0.83 (m, 1 H), 0.78 (d, *J* = 7.1 Hz, 3 H) ppm; ^{13}C NMR (176 MHz, Acetone-d_6) δ 207.28, 139.66, 139.56, 129.15, 129.10, 128.65, 128.48, 128.39, 128.27, 109.79, 80.65, 80.58, 80.55, 74.79, 73.76, 69.56, 49.53, 47.01, 44.20, 40.41, 35.26, 32.33, 30.03, 26.18, 23.93, 22.78, 21.51, 16.55 ppm; ^{13}C NMR (176 MHz, Acetone) δ 109.82 (d, *J* = 168.9 Hz) ppm. MS (ESI): 531.3 (M+Na); HRMS (ESI): calculated for $C_{32}H_{44}O_5Na$ (M+Na): 531.30810, Found: 531.30724. $[\alpha]_D^{20}$ = +18.8 (c = 0.29, CH_2Cl_2).

2-Admantanyl 3,5-*O*-di-benzyl-2-*C*-acetylmethyl-2-deoxy-α-D-lyxofuranoside

The title product compound is prepared using **Method A** and isolated by flash column chromatography (9:1-3:1 Pentane: Ethyl Acetate) giving a pale yellow syrup (93 mg, 0.17 mmol, 82% yield, α/β ratio 78:22)

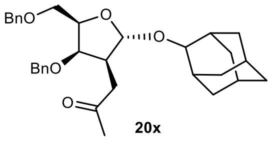

20x

^{1}H NMR (700 MHz, Acetone-d_6) δ 7.38-7.26 (m, 10 H), 4.96 (d, *J* = 4.7 Hz, 1 H), 4.60-4.53 (m, 3 H), 4.37-4.34 (m, 2 H), 4.30 (dd, *J* = 5.5, 3.9 Hz, 1 H), 3.80 (dd, *J* = 9.7, 6.5 Hz, 1 H), 3.70-3.69 (m, 1 H), 3.63 (dd, *J* = 9.8, 5.8 Hz, 1 H), 2.87-2.83 (m, 1 H), 2.70-2.68 (m, 1 H), 2.56 (dd, *J* = 17.5, 5.3 Hz, 1 H), 2.11 (d, *J* = 11.4 Hz, 1 H), 2.04 (d, *J* = 2.8 Hz, 3 H), 2.03-2.02 (m, 1 H), 2.01-1.93 (m, 2 H), 1.85-1.80 (m, 2 H), 1.78-1.71 (m, 2 H), 1.74-1.66 (m, 4 H), 1.47 (d, *J* = 11.3 Hz, 2 H) ppm; ^{13}C NMR (176 MHz, Acetone-d_6) δ 207.35, 139.68, 139.64, 129.16, 129.14, 128.67, 128.58, 128.40, 128.31, 106.67, 80.95, 80.79, 80.67, 74.84, 73.83, 69.56, 46.99, 40.48, 38.36, 37.30, 37.20, 34.43, 32.43, 32.42, 32.31, 30.03, 28.47, 28.25 ppm; ^{13}C NMR (176 MHz, Acetone-d_6) δ 106.67 (d, *J* = 183.1 Hz) ppm. MS (ESI): 527.2 (M+Na); HRMS (ESI): calculated for $C_{32}H_{40}O_5Na$ (M+Na): 527.27680, Found: 527.27595. $[\alpha]_D^{20}$ = +54.4 (c = 0.36, CH_2Cl_2).

1-Admantanyl 3,5-*O*-di-benzyl-2-*C*-acetylmethyl-2-deoxy-α-D-lyxofuranoside

The title product compound is prepared using **Method A** and isolated by flash column chromatography (9:1-3:1 Pentane: Ethyl Acetate) giving a pale yellow syrup (53 mg, 0.11 mmol, 53% yield, α only)

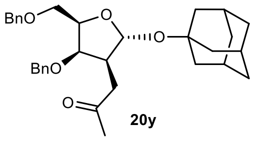

20y

^{1}H NMR (700 MHz, Acetone-d_6) δ 7.38 (d, *J* = 7.6 Hz, 2 H), 7.35-7.32 (m, 4 H), 7.29-7.27 (m, 4 H), 5.17 (d, *J* = 5.4 Hz, 1 H), 4.60-4.55 (m, 3 H), 4.36-4.33 (m, 2 H), 4.23-4.21 (m, 1 H), 3.77 (dd, *J* = 9.6, 6.9 Hz, 1 H), 3.61 (dd, *J* = 9.6, 5.7 Hz, 1 H), 2.84-2.79 (m, 1 H), 2.52-2.47 (m, 2 H), 2.09-2.08 (m, 3 H), 2.04 (s, 3 H), 1.82-1.80 (m, 3 H), 1.74-1.71 (m, 3 H), 1.65-1.59 (m, 6 H) ppm; ^{13}C NMR (176 MHz, Acetone-d_6) δ 207.35, 139.71, 139.67, 129.16, 129.13, 128.77, 128.54, 128.41, 128.29, 101.27, 80.74, 80.71, 74.86, 73.79, 73.76, 69.53, 47.38, 43.58, 40.08, 37.09, 31.54, 29.98 ppm; ^{13}C NMR (176 MHz, Acetone-d_6) δ 101.28 (d, *J* = 166.5 Hz) ppm. MS (ESI): 527.2 (M+Na); HRMS (ESI): calculated for $C_{32}H_{40}O_5Na$ (M+Na): 527.27680, Found: 527.27605. $[\alpha]_D^{20}$ = +36.1 (c = 0.48, CH_2Cl_2).

***N,N*-Di-*tert*-butoxylcarbonyl-9-(3,5-*O*-di-benzyl-2-*C*-acetylmethyl-2-deoxy-α-D-lyxofuranosyl)adenine**

The title product compound is prepared using **Method A** with a modification that glycosyl acceptor (0.24 mmol, 1.2 equiv.) and 0.5 mol% catalyst **A** is used instead and isolated by flash column chromatography (1:1 Pentane: Ethyl Acetate) giving a pale yellow syrup (88 mg, 0.13 mmol, 64% yield, α only)

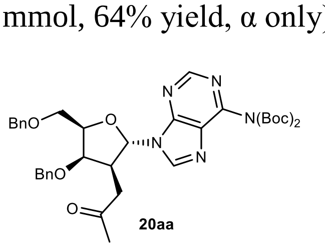

20aa

^{1}H NMR (500 MHz, Acetone-d_6) δ 8.80 (s, 1 H), 8.61 (s, 1 H), 7.39-7.26 (m, 10 H), 6.14 (d, *J* = 8.4 Hz, 1 H), 4.98 (td, *J* = 6.6, 2.7 Hz, 1 H), 4.71 (d, *J* = 11.3 Hz, 1 H), 4.59-4.56 (m, 3 H), 4.52 (d, *J* = 11.3 Hz, 1 H), 3.88 (dd, *J* = 9.5, 6.9 Hz, 1 H), 3.78-3.75 (m, 1 H), 3.73 (dd, *J* = 9.5, 5.9 Hz, 1 H), 3.04 (dd, *J* = 18.5, 8.7 Hz, 1 H), 2.81 (dd, *J* = 18.3, 5.2 Hz, 1 H), 1.96 (s, 3 H), 1.43 (s, 18 H) ppm; ^{13}C NMR (126 MHz, Acetone) δ 206.59,

154.06, 152.45, 151.28, 151.09, 145.85, 139.37, 139.33, 130.11, 129.29, 129.18, 128.94, 128.67, 128.64, 128.42, 89.83, 83.86, 83.57, 81.48, 75.15, 73.96, 68.92, 45.23, 39.86, 29.84, 27.92 ppm. MS (ESI): 688.4 (M+H); HRMS (ESI): Calculated for $C_{37}H_{45}O_8N_5Na$ (M+Na): 710.31603, Found: 710.31556. $[\alpha]_D^{20}$ = +23.0 (c = 0.77, CH_2Cl_2).

***N*,*N*-Di-*tert*-butoxylcarbonyl-9-(3,5-*O*-di-benzyl-2-*C*-acetylmethyl-2-deoxy-α-L-lyxofuranosyl)adenine**

The title product compound is prepared using **Method A** with a modification that glycosyl acceptor (0.24 mmol, 1.2 equiv.) and 0.5 mol% catalyst **A** is used instead and isolated by flash column chromatography (1:1 Pentane: Ethyl Acetate) giving a pale yellow syrup (91 mg, 0.13 mmol, 66% yield, α only)

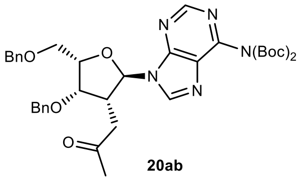

20ab

^{1}H NMR (500 MHz, Acetone-d_6) δ 8.81 (s, 1 H), 8.61 (s, 1 H), 7.39-7.32 (m, 10 H), 6.15 (d, *J* = 8.4 Hz, 1 H), 5.01-4.97 (m, 1 H), 4.71 (d, *J* = 11.3 Hz, 1 H), 4.59-4.56 (m, 3 H), 4.52 (d, *J* = 11.3 Hz, 1 H), 3.89 (dd, *J* = 9.5, 6.9 Hz, 1 H), 3.79-3.71 (m, 2 H), 3.04 (dd, *J* = 18.4, 8.8 Hz, 1 H), 2.81 (dd, *J* = 18.3, 5.2 Hz, 1 H), 1.96 (s, 3 H), 1.44 (s, 18 H) ppm; ^{13}C NMR (126 MHz, Acetone-d_6) δ 206.56, 154.02, 152.41, 151.25, 151.05, 145.81, 139.31, 139.27, 130.07, 129.26, 129.15, 128.90, 128.64, 128.61, 128.39, 89.80, 83.84, 83.53, 81.43, 75.11, 73.92, 68.88, 45.19, 39.84, 29.83, 27.91 ppm. MS (ESI): 688.2 (M+H); HRMS (ESI): Calculated for $C_{37}H_{46}O_8N_5$ (M+H): 688.33409, Found: 688.33394. $[\alpha]_D^{20}$ = -32.9 (c = 0.37, CH_2Cl_2).

***N*,*N*-Di-*tert*-butoxylcarbonyl-9-(3,5-*O*-di-benzyl-2-*C*-acetylmethyl-2-deoxy-β-D-ribofuranosyl)adenine**

The title product compound is prepared using **Method A** with a modification that glycosyl acceptor (0.24 mmol, 1.2 equiv.) and 0.5 mol% catalyst **A** is used instead and isolated by flash column chromatography (1:1 Pentane: Ethyl Acetate) giving a pale yellow syrup (93 mg, 0.14 mmol, 68% yield, β only)

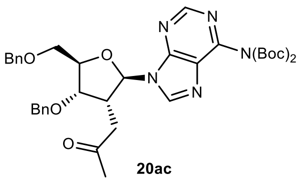

20ac

^{1}H NMR (500 MHz, Acetone-d_6) δ 8.77 (s, 1 H), 8.60 (s, 1 H), 7.43 (d, *J* = 7.1 Hz, 2 H), 7.39-7.34 (m, 6 H), 7.33-7.29 (m, 2 H), 6.24 (d, *J* = 8.9 Hz, 1 H), 4.71 (d, *J* = 11.7 Hz, 1 H), 4.69-4.62 (m, 2 H), 4.50-4.46 (m, 3 H), 3.87 (dd, *J* = 10.3, 4.9 Hz, 1 H), 3.76 (dd, *J* = 10.3, 3.7 Hz, 1 H), 3.64-3.58 (m, 1 H), 3.09 (dd, *J* = 18.3, 8.7 Hz, 1 H), 2.67 (dd, *J* = 18.3, 5.3 Hz, 1 H), 2.00 (s, 3 H), 1.43 (s, 18 H) ppm; ^{13}C NMR (126 MHz, Acetone) δ 206.25, 154.27, 152.55, 151.23, 150.99, 145.01, 139.24, 139.10, 129.57, 129.29, 129.23, 128.79, 128.60, 128.57, 128.51, 89.24, 84.14, 83.85, 81.65, 73.99, 72.24, 71.58, 44.29, 38.97, 30.07, 27.91 ppm. MS (ESI): 688.2 (M+H); HRMS (ESI): Calculated for $C_{37}H_4O_8N_5$ (M+H): 688.33409, Found: 688.33385. $[\alpha]_D^{20}$ = -29.3 (c = 0.58, CH_2Cl_2).

2-*tert*-Butoxycarbonylamino-6-chloro-9-(3,5-*O*-di-benzyl-2-*C*-acetylmethyl-2-deoxy-α-D-lyxofuranosyl)purine

The title product compound is prepared using **Method A** with a modification that glycosyl acceptor (0.24 mmol, 1.2 equiv.) and 0.5 mol% catalyst **A** is used instead and isolated by flash column chromatography (1:1 Pentane: Ethyl Acetate) giving a pale yellow syrup (82 mg, 0.13 mmol, 66% yield, α only)

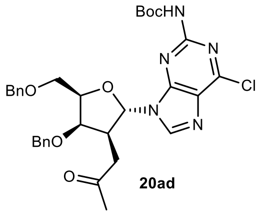

20ad

^{1}H NMR (500 MHz, Acetone-d_6) δ 9.23 (s, 1 H), 8.39 (s, 1 H), 7.39-7.26 (m, 10 H), 5.99 (d, *J* = 7.9 Hz, 1 H), 5.16-5.12 (m, 1 H), 4.70 (d, *J* = 11.2 Hz, 1 H), 4.65-4.57 (m, 3 H), 4.50 (d, *J* = 11.2 Hz, 1 H), 3.87 (dd, *J* = 9.8, 7.0 Hz, 1 H), 3.73 (dd, *J* = 9.8, 5.8 Hz, 1 H), 3.68-3.65 (m, 1 H), 3.00 (d, *J* = 7.1 Hz, 2 H), 1.99 (s, 3 H), 1.51 (s, 9 H) ppm; ^{13}C NMR (126 MHz, Acetone) δ 206.94, 153.58, 153.44, 151.31, 150.86, 145.10, 139.51, 139.43, 129.33, 129.26, 129.15, 128.95, 128.62, 128.60, 128.36, 90.43, 83.42, 81.54, 80.83, 75.12, 73.82, 68.90, 45.47, 40.16, 29.92, 28.44 ppm. MS (ESI): 622.2 (M+H); HRMS (ESI): Calculated for $C_{32}H_{36}O_6N_5ClNa$ (M+Na): 644.22463, Found: 644.22420. $[\alpha]_D^{20}$ = +20.6 (c = 0.20, CH_2Cl_2).

***N*⁴-(2-Methylpropionyl)-1-(3,5-*O*-di-benzyl-2-*C*-acetylmethyl-2-deoxy-α-D-lyxofuranosyl)cytosine**

The title product compound is prepared using **Method C** and isolated by flash column chromatography (1:1 Pentane: Ethyl Acetate) giving a pale yellow syrup (53 mg, 0.10 mmol, 50% yield, α only)

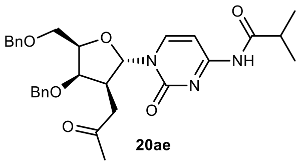

20ae

^{1}H NMR (500 MHz, Acetone-d_6) δ 9.67 (s, br, 1 H), 8.08 (d, *J* = 7.5 Hz, 1 H), 7.39-7.28 (m, 11 H), 6.03-6.01 (m, 1 H), 4.80-4.78 (m, 1 H), 4.65 (d, *J* = 11.3 Hz, 1 H), 4.62-4.58 (m, 2 H), 4.46 (d, *J* = 11.3 Hz, 1 H), 4.41-4.40 (m, 1 H), 3.84 (dd, *J* = 9.7, 6.8 Hz, 1 H), 3.72 (dd, *J* = 9.7, 5.9 Hz, 1 H), 2.96-2.90 (m, 3 H), 2.88-2.82 (m, 1 H), 2.01 (s, 3 H), 1.17 (dd, *J* = 6.9, 2.1 Hz, 6 H) ppm; ^{13}C NMR (126 MHz, Acetone-d_6) δ 206.82, 178.30, 163.60, 156.09, 145.79, 139.42, 139.32, 136.31, 129.27, 129.19, 128.92, 128.67, 128.60, 128.43, 96.90, 91.08, 83.50, 81.64, 75.10, 73.94, 69.10, 47.47, 39.84, 36.65, 19.51, 19.44 ppm; ^{13}C NMR (126 MHz, Acetone-d_6) δ 91.08 (d, *J* = 170.0 Hz) ppm. MS (ESI): 534.2 (M+H); HRMS (ESI): Calculated for $C_{30}H_{36}O_6N_3$ (M+H): 534.25986, Found: 534.25926. $[\alpha]_D^{20}$ = -8.9 (c = 0.23, CH_2Cl_2).

1-(3,5-*O*-di-benzyl-2-*C*-acetylmethyl-2-deoxy-α-D-lyxofuranosyl)-1*H*-benzo[*d*][1,2,3]triazole

The title product compound is prepared using **Method A** with a modification that glycosyl acceptor (0.24 mmol, 1.2 equiv.) and 0.5 mol% catalyst **A** is used instead and isolated by flash column chromatography (9:1-3:1 Pentane: Ethyl Acetate) giving a pale yellow syrup (59 mg, 0.13 mmol, 63% yield, α only)

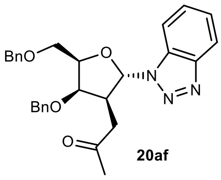

20af

^{1}H NMR (700 MHz, Acetone-d_6) δ 8.03 (d, *J* = 8.3 Hz, 1 H), 7.89 (d, *J* = 8.4 Hz, 1 H), 7.57-7.5 (m, 1 H), 7.44-7.38 (m, 1 H), 7.39-7.26 (m, 10 H), 6.41 (d, *J* = 7.9 Hz, 1 H), 4.79-4.76 (m, 1 H), 4.72 (d, *J* = 11.1 Hz, 1 H), 4.66-4.64 (m, 1 H), 4.61-4.56 (m, 2 H), 4.52 (d, *J* = 11.2 Hz, 1 H), 3.92 (dd,

J = 9.8, 6.5 Hz, 1 H), 3.86-3.83 (m, 1 H), 3.74 (dd, J = 9.8, 6.0 Hz, 1 H), 3.09 (dd, J = 18.3, 9.5 Hz, 1 H), 2.71 (dd, J = 18.3, 4.7 Hz, 1 H), 2.00 (s, 3 H) ppm; ^{13}C NMR (176 MHz, Acetone-d_6) δ 206.71, 147.38, 139.43, 139.32, 133.60, 129.30, 129.17, 128.99, 128.66, 128.63, 128.48, 128.39, 125.04, 120.48, 111.69, 91.89, 83.32, 80.97, 75.23, 73.95, 69.07, 44.93, 39.84, 30.03 ppm. MS (ESI): 494.2 (M+Na); HRMS (ESI): Calculated for $C_{28}H_{29}O_4N_3Na$ (M+Na): 494.20503, Found: 494.20450. $[\alpha]_D^{20}$ = +60.0 (c = 0.37, CH_2Cl_2).

1-(3,5-*O*-di-benzyl-2-*C*-acetylmethyl-2-deoxy-β-D-ribofuranosyl)-1*H*-benzo[*d*][1,2,3]triazole

The title product compound is prepared using **Method A** with a modification that glycosyl acceptor (0.24 mmol, 1.2 equiv.) and 0.5 mol% catalyst **A** is used instead and isolated by flash column chromatography (9:1-3:1 Pentane: Ethyl Acetate) giving a pale yellow syrup (85 mg, 0.18 mmol, 90% yield, β/α ratio 87:13)

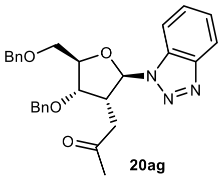

^{1}H NMR (700 MHz, Acetone-d_6) δ 8.03-7.99 (m, 2 H), 7.40-7.29 (m, 12 H), 6.44 (d, J = 8.3 Hz, 1 H), 4.70 (d, J = 11.5 Hz, 1 H), 4.60-4.59 (m, 3 H), 4.52 (d, J = 11.5 Hz, 1 H), 4.49-4.47 (m, 1 H), 3.79-7.76 (m, 2 H), 3.68 (dd, J = 10.4, 4.2 Hz, 1 H), 3.13 (dd, J = 18.1, 9.4 Hz, 1 H), 2.54 (dd, J = 18.2, 4.9 Hz, 1 H), 2.06 (d, J = 3.7 Hz, 3 H) ppm; ^{13}C NMR (176 MHz, Acetone-d_6) δ 206.34, 147.45, 139.29, 139.13, 133.38, 129.22, 129.18, 128.83, 128.58, 128.51, 128.38, 128.29, 124.98, 120.35, 112.45, 92.56, 84.50, 80.76, 73.92, 72.58, 71.62, 42.96, 38.90, 30.05 ppm. MS (ESI): 494.0 (M+Na); HRMS (ESI): Calculated for $C_{28}H_{29}O_4N_3Na$ (M+Na): 494.20503, Found: 494.20449. $[\alpha]_D^{20}$ = -64.2 (c = 0.67, CH_2Cl_2).

***N*-(3,5-*O*-di-benzyl-2-*C*-acetylmethyl-2-deoxy-α-D-lyxofuranosyl)-indoline**

The title product compound is prepared using **Method A** with a modification that glycosyl acceptor (0.24 mmol, 1.2 equiv.) is used instead and isolated by flash column chromatography (4:1 Pentane: Ethyl Acetate) giving a pale yellow syrup (64 mg, 0.14 mmol, 68% yield, α only)

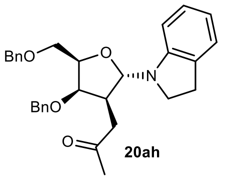

^{1}H NMR (700 MHz, $CDCl_3$) δ 7.28-7.16 (m, 10 H), 6.97-6.64 (m, 2 H), 6.61-6.58 (m, 1 H), 6.55 (d, J = 8.1 Hz, 1 H), 5.34 (d, J = 9.2 Hz, 1 H), 4.58 (d, J = 11.4 Hz, 1 H), 4.47-4.42 (m, 2 H), 4.28 (d, J = 11.4 Hz, 1 H), 4.25-4.22 (m, 2 H), 3.65-3.63 (m, 1 H), 3.57 (dd, J = 9.3, 4.9 Hz, 1 H), 3.45-3.43 (m, 1 H), 3.36-3.35 (m, 1 H), 2.92-2.83 (m, 2 H), 2.76-2.71 (m, 2 H), 2.44-2.40 (m, 1 H), 1.93 (s, 3 H) ppm; ^{13}C NMR (176 MHz, $CDCl_3$) δ 207.71, 150.66, 138.38, 138.13, 130.46, 128.60, 128.50, 128.18, 127.97, 127.95, 127.79, 127.37, 124.91, 119.11, 108.27, 89.65, 80.92, 79.75, 74.52, 73.61, 68.43, 44.84, 41.23, 39.22, 30.23, 28.15 ppm. MS (ESI): 472.2 (M+H); HRMS (ESI): Calculated for $C_{30}H_{34}O_4N$ (M+H): 472.24824, Found: 472.24762. $[\alpha]_D^{20}$ = +3.9 (c = 0.47, CH_2Cl_2).

Phenyl 3,5-*O*-di-benzyl-2-*C*-acetylmethyl-1-deoxy-1-thio-α-L-lyxofuranoside

The title product compound is prepared using **Method A** and isolated by flash column chromatography (20:1-9:1 Pentane: Ethyl Acetate) giving a pale yellow syrup (66 mg, 0.14 mmol, 71% yield, α only)

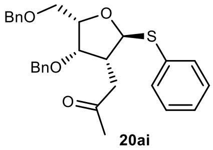

20ai

^{1}H NMR (700 MHz, Acetone-d_6) δ 7.50 (d, *J* = 7.7 Hz, 2 H), 7.37 (d, *J* = 7.0 Hz, 2 H), 7.35-7.24 (m, 11 H), 5.23 (d, *J* = 8.4 Hz, 1 H), 4.61-4.55 (m, 3 H), 4.38-4.36 (m, 2 H), 4.28-4.27 (m, 1 H), 3.83 (dd, *J* = 9.7, 6.9 Hz, 1 H), 3.68 (dd, *J* = 9.7, 5.7 Hz, 1 H), 2.94 (dd, *J* = 18.1, 10.3 Hz, 1 H), 2.71 (dd, *J* = 18.1, 3.8 Hz, 1 H), 2.67-2.64 (m, 1 H), 2.06 (s, 3 H) ppm; ^{13}C NMR (176 MHz, Acetone-d_6) δ 206.98, 139.50, 139.39, 136.27, 129.75, 129.20, 129.15, 128.78, 128.57 (two carbons), 128.49, 128.35, 127.82, 90.98, 81.92, 80.22, 75.01, 73.85, 68.82, 46.75, 40.57, 30.01 ppm; Gated ^{13}C NMR (176 MHz, Acetone-d_6) δ 107.86 (d, *J* = 167.8 Hz) ppm. MS (ESI): 485.2 (M+Na); HRMS (ESI): Calculated for $C_{28}H_{30}O_4NaS$ (M+Na): 485.17570, Found: 485.17464. $[\alpha]_D^{20}$ = +152.2 (c = 0.38, CH_2Cl_2).

Phenyl 3,5-*O*-di-benzyl-2-*C*-acetylmethyl-1-deoxy-1-thio-β-D-ribofuranoside

The title product compound is prepared using **Method A** with a modification that the reaction is performed at room temperature and isolated by flash column chromatography (20:1-9:1 Pentane: Ethyl Acetate) giving a pale yellow syrup (55 mg, 0.12 mmol, 59% yield, β/α ratio 92:8)

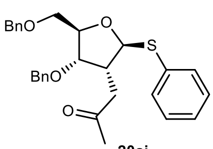

20aj

^{1}H NMR (500 MHz, Acetone-*d_6*) δ 7.51-7.48 (m, 2 H), 7.38-7.26 (m, 13 H), 5.14 (d, *J* = 9.2 Hz, 1 H), 4.60-4.54 (m, 3 H), 4.35 (d, *J* = 11.7 Hz, 1 H), 4.30-4.27 (m, 1 H), 4.16 (dd, *J* = 5.7, 1.7 Hz, 1 H), 3.54 (dd, *J* = 10.1, 4.8 Hz, 1 H), 3.41 (dd, *J* = 10.1, 6.5 Hz, 1 H), 2.98 (dd, *J* = 17.7, 9.8 Hz, 1 H), 2.69-2.59 (m, 2 H), 2.11 (s, 3 H) ppm; ^{13}C NMR (126 MHz, Acetone-*d_6*) δ 206.75, 139.61, 139.26, 135.40, 132.38, 129.78, 129.18, 129.17, 128.76, 128.47, 128.42, 128.32, 128.06, 90.96, 84.30, 81.13, 73.82, 72.19, 71.81, 44.28, 39.71, 30.23 ppm. MS (ESI): 485.0 (M+Na); HRMS (ESI): Calculated for $C_{28}H_{30}O_4NaS$ (M+Na): 485.17570, Found: 485.17510. $[\alpha]_D^{20}$ = -146.2 (c = 0.57, CH_2Cl_2).

***S*-(2-*C*-Acetylmethyl-3,5-di-*O*-benzyl-2-deoxy-α-D-lyxofuranosyl)-*N*-(*tert*-butoxycarbonyl)-L-cysteine Methyl ester**

The title product compound is prepared using **Method A** with a modification that glycosyl acceptor (0.24 mmol, 1.2 equiv.) is used instead and isolated by flash column chromatography (3:1 Pentane: Ethyl Acetate) giving a pale yellow syrup (75 mg, 0.13 mmol, 64% yield, α only)

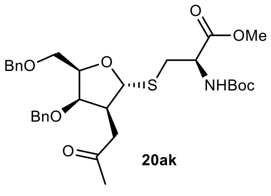

20ak

^{1}H NMR (500 MHz, Acetone-*d_6*) δ 7.39 (d, *J* = 7.0 Hz, 2 H), 7.36-7.28 (m, 4 H), 7.29-7.27 (m, 4 H), 6.61 (d, *J* = 8.5 Hz, 1 H), 4.95 (d, *J* = 8.7 Hz, 1 H), 4.64-4.58 (m, 3 H), 4.52-4.50 (m, 1 H), 4.39-4.35 (m, 1 H), 4.34 (d, *J* = 17.5 Hz, 1 H), 4.27-4.25 (m, 1 H), 3.83 (dd, *J* = 9.4, 7.7 Hz, 1 H), 3.73-3.69 (m, 1 H), 3.67 (s, 3 H), 3.20 (dd, *J* = 14.6, 6.3 Hz, 1 H), 2.99 (dd, *J* = 14.6, 4.1 Hz, 1 H), 2.88 (dd, *J* = 18.1, 10.3 Hz, 1 H), 2.62 (dd, *J* = 18.1, 3.8 Hz, 1 H), 2.55-2.51 (m, 1 H), 2.04 (s, 3 H), 1.39 (s, 9 H) ppm; ^{13}C NMR (126 MHz, Acetone-*d_6*) δ 206.98, 172.00, 156.22, 139.42, 139.37, 129.19, 129.16, 128.79, 128.57, 128.50, 128.36, 90.03, 81.38, 80.26, 79.37, 74.97, 73.91, 68.43, 54.99, 47.03, 40.55, 35.23, 29.99, 28.61 ppm. MS (ESI): 610.0 (M+Na); HRMS (ESI): Calculated for $C_{31}H_{41}O_8NNaS$ (M+Na): 610.24451, Found: 610.24397. $[\alpha]_D^{20}$ = +25.6 (c = 0.63, CH_2Cl_2).

4-Methoxyphenyl 3,5-*O*-di-benzyl-2-*C*-acetylmethyl-1-deoxy-1-thio-α-D-lyxofuranoside

The title product compound is prepared using **Method A** and isolated by flash column chromatography (20:1-9:1 Pentane: Ethyl Acetate) giving a pale yellow syrup (48.0 mg, 0.10 mmol, 49% yield, α/β 94:6)

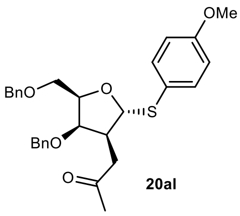

^{1}H NMR (500 MHz, Acetone-d_6) δ 7.54-7.49 (m, 2 H), 7.40-7.29 (m, 10 H), 6.91 (d, *J* = 8.5 Hz, 2 H), 5.11-5.07 (m, 1 H), 4.62-4.55 (m, 3 H), 4.38-4.34 (m, 2 H), 4.26 (s, 1 H), 3.84-3.81 (m, 1 H), 3.78 (s, 3 H), 3.72-3.70 (m, 1 H), 2.92 (dd, *J* = 17.7, 10.0 Hz, 1 H), 2.72 (dd, *J* = 18.1, 3.7 Hz, 1 H), 2.66-2.59 (m, 1 H), 2.07 (s, 3 H) ppm; ^{13}C NMR (126 MHz, Acetone-d_6) δ 207.04, 160.58, 139.38, 139.30, 135.56, 129.16, 129.12, 128.70, 128.49, 128.44, 128.29, 125.26, 115.25, 91.68, 81.80, 80.23, 74.87, 73.75, 68.80, 55.64, 46.27, 40.36, 30.07 ppm. MS (ESI): 527.2 (M+Na); HRMS (ESI): Calculated for $C_{29}H_{32}O_5NaS$ (M+Na): 515.18627, Found: 515.18548. $[\alpha]_D^{20}$ = +105.0 (c = 0.81, CH_2Cl_2).

(2*R*, 3*aS*, 4*R*, 5*R*, 6*aS*)-4-benzyloxy-5-benzyloxymethyl-2-methyl-(2-(4-methoxyphenylthio))-hexahydrofuro[2,3-*b*]furan

The title product compound is prepared using **Method A** and isolated by flash column chromatography (20:1-9:1 Pentane: Ethyl Acetate) giving a pale yellow syrup mixed with **20al** (22% yield calculated by integration of ^{1}H spectrum)

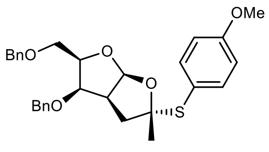

20al-side

^{1}H NMR (500 MHz, Acetone-d_6) δ 7.46 (d, *J* = 8.8 Hz, 2 H), 7.39-7.22 (m, 10 H), 6.94 (d, *J* = 8.8 Hz, 2 H), 5.79 (d, *J* = 5.7 Hz, 1 H), 4.59-4.50 (m, 4 H), 4.37-4.36 (m, 1 H), 4.36-4.33 (m, 1 H), 3.82 (s, 3 H), 3.82-3.78 (m, 1 H), 3.69-3.65 (m, 1 H), 3.25-3.18 (m, 1 H), 2.36 (dd, *J* = 13.9, 7.9 Hz, 1 H), 2.15-2.09 (m, 1 H), 1.48 (s, 3 H) ppm;

^{13}C NMR (126 MHz, acetone-d_6) δ 161.80, 140.25, 139.60, 139.06, 129.62, 129.45, 129.00, 128.93, 128.89, 128.83, 124.70, 115.49, 111.09, 97.03, 82.85, 79.74, 74.05, 73.90, 71.06, 56.11, 47.15, 39.32, 29.25 ppm. MS (ESI): 527.2 (M+Na); HRMS (ESI): Calculated for $C_{29}H_{32}O_5NaS$ (M+Na): 515.18627, Found: 515.18548.

1-Deoxy-1-(*C*-2’,4’,6’-trimethoxyphenyl)-4,5-di-*O*-benzyl-2-*C*-acetylmethyl-α-D-lyxofuranoside

The title product compound is prepared using **Method D** and isolated by flash column chromatography (9:1-3:1 Pentane: Ethyl Acetate) giving a pale yellow syrup (40 mg, 0.08 mmol, 76% yield, α/β ratio 85:15)

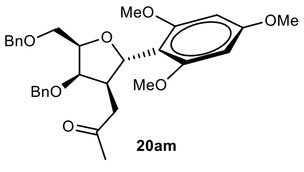

^{1}H NMR (700 MHz, Acetone-d_6) δ 7.39-7.27 (m, 10 H), 6.22 (s, 2 H), 5.25 (d, *J* = 10.9 Hz, 1 H), 4.65 (d, *J* = 11.2 Hz, 1 H), 4.59-4.54 (m, 2 H), 4.51-4.49 (m, 1 H), 4.41 (d, *J* = 11.2 Hz, 1 H), 4.31 (t, *J* = 3.6 Hz, 1 H), 3.84-3.80 (m, 1 H), 3.79 (s, 9 H), 3.62 (dd, *J* = 9.4, 5.4 Hz, 1 H), 3.21-3.20 (m, 1 H), 2.79 (dd, *J* = 18.1, 10.5 Hz, 1 H), 2.24 (dd, *J* = 18.1, 3.5 Hz, 1 H), 1.95 (s, 3 H) ppm; ^{13}C NMR (176 MHz, Acetone) δ 207.86, 161.98, 161.02, 139.93, 139.77, 129.20, 129.13, 128.91, 128.60, 128.40, 128.28, 109.91, 92.15, 82.28, 81.89, 75.80, 74.92, 73.87, 69.78, 56.36, 55.61, 43.92, 40.29, 30.03 ppm; ^{13}C NMR (176 MHz, Acetone-d_6) δ 75.80 (d, *J* = 148.6 Hz) ppm. MS (ESI): 527.2 (M+Na); HRMS (ESI): Calculated for $C_{31}H_{36}O_7Na$ (M+Na): 543.23532, Found: 543.23479. $[\alpha]_D^{20}$ = +28.4 (c = 0.49, CH_2Cl_2).

1-Deoxy-1-(*C*-2’,4’,6’-trimethoxyphenyl)-4,5-di-*O*-benzyl-2-*C*-acetylmethyl-α-L-lyxofuranoside

The title product compound is prepared using **Method D** and isolated by flash column chromatography (9:1-3:1 Pentane: Ethyl Acetate) giving a pale yellow syrup (61 mg, 0.12 mmol, 58% yield, α/β ratio 85:15)

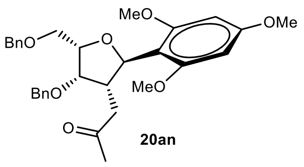

^{1}H NMR (500 MHz, Acetone-d_6) δ 7.40-7.27 (m, 10 H), 6.22 (s, 2 H), 5.23 (d, *J* = 10.9 Hz, 1 H), 4.65 (d, *J* = 11.2 Hz, 1 H), 4.60-4.54 (m, 2 H), 4.51-4.47 (m, 1 H), 4.40 (d, *J* = 11.2 Hz, 1 H), 4.32-4.29 (m, 1 H), 3.83-3.80 (m, 1 H), 3.79 (s, 9 H), 3.61 (dd, *J* = 9.2, 5.4 Hz, 1 H), 3.21-3.16 (m, 1 H), 2.78 (dd, *J* = 18.1, 10.6 Hz, 1 H), 2.23 (dd, *J* = 18.1, 3.5 Hz, 1 H), 1.95 (s, 3 H) ppm; ^{13}C NMR (126 MHz, Acetone-d_6) δ 207.84, 161.98, 161.01, 139.94, 139.79, 129.21, 129.15, 128.95, 128.62, 128.42, 128.30, 109.84, 92.06, 82.23, 81.87, 75.75, 74.91, 73.86, 69.75, 56.33, 55.60, 43.87, 40.24, 30.07 ppm. MS (ESI): 1063.4 (2M+Na); HRMS (ESI): Calculated for $C_{31}H_{36}O_7Na$ (M+Na): 543.23532, Found: 543.23483. $[\alpha]_D^{20}$ = -26.6 (c = 0.15, CH_2Cl_2).

1-Deoxy-1-(*C*-2’,4’,6’-trimethoxyphenyl)-4,5-di-*O*-benzyl-2-*C*-acetylmethyl-β-D-ribofuranoside

The title product compound is prepared using **Method D** and isolated by flash column chromatography (9:1-3:1 Pentane: Ethyl Acetate) giving a pale yellow syrup (57 mg, 0.11 mmol, 55% yield, β/α ratio 88:12)

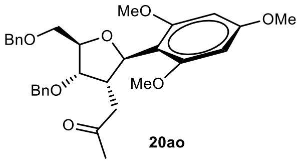

^{1}H NMR (500 MHz, Acetone-d_6) δ 7.41 (d, *J* = 7.3 Hz, 2 H), 7.38-7.32 (m, 6 H), 7.30-7.27 (m, 2 H), 6.20 (s, 2 H), 5.24 (d, *J* = 11.0 Hz, 1 H), 4.64-4.62 (m, 3 H), 4.34 (d, *J* = 11.6 Hz, 1 H), 4.19-4.15 (m, 2 H), 3.79 (s, 3 H), 3.73 (s, 6 H), 3.59-3.57 (m, 2 H), 3.28-3.22 (m, 1 H), 2.81 (dd, *J* = 17.6, 10.5 Hz, 1 H), 2.11 (dd, *J* = 17.8, 4.2 Hz, 1 H), 2.05 (dd, *J* = 17.5, 4.0 Hz, 1 H), 1.97 (s, 3 H) ppm; ^{13}C NMR (126 MHz, Acetone-d_6) δ 207.52, 162.22, 161.44, 139.93, 139.73, 129.16, 129.11, 128.87, 128.43, 128.33, 128.25, 107.80, 92.06, 83.11, 82.86, 76.48, 73.73, 72.23, 71.90, 56.03, 55.58, 40.89, 39.77, 30.23 ppm. MS (ESI): 1063.4 (2M+Na); HRMS (ESI): Calculated for $C_{31}H_{36}O_7Na$ (M+Na): 543.23532, Found: 543.23562. $[\alpha]_D^{20}$ = -47.1 (c = 0.46, CH_2Cl_2).

((2R,3S,4R,5R,6R)-6-(allyloxy)-3,4-bis(benzyloxy)-5-(2-oxopropyl)tetrahydro-2H-pyran-2-yl)methyl acetate

The title product compound is prepared using **Method E** and isolated by flash column chromatography (20:1-9:1 Pentane: Ethyl Acetate) giving a pale yellow syrup (38 mg, 0.079 mmol, 79% yield).

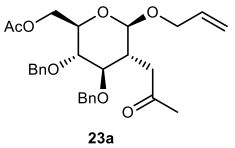

^{1}H NMR (500 MHz, CD_2Cl_2) δ 7.36-7.29 (m, 10 H), 5.86 (ddt, *J* = 16.4, 10.9, 5.7 Hz, 1 H), 5.23 (d, *J* = 17.2 Hz, 1 H), 5.17 (d, *J* = 9.7 Hz, 1 H), 4.90 (d, *J* = 11.2 Hz, 1 H), 4.83 (d, *J* = 10.9 Hz, 1 H), 4.58 (dd, *J* = 10.8, 9.1 Hz, 2 H), 4.38-4.32 (m, 2 H), 4.2-4.25 (m, 2 H), 4.00-3.95 (m, 1 H), 3.60-3.48 (m, 3 H), 2.54-2.44 (m, 2 H), 2.24-2.17 (m, 1 H), 2.07 (s, 3 H), 2.04 (s, 3 H) ppm; ^{13}C NMR (126 MHz, CD_2Cl_2) δ 207.54, 171.14, 138.82, 138.50, 134.46, 128.94, 128.91, 128.43,

128.38, 128.29, 128.21, 117.61, 102.31, 82.90, 80.07, 75.22, 73.62, 70.52, 63.49, 45.04, 41.90, 30.23, 21.23 ppm. MS (ESI): 505.2 (M+Na); HRMS (ESI): Calculated for $C_{28}H_{34}O_7Na$ (M+Na): 505.21967, Found: 505.21866. $[\alpha]_D^{20}$ = -4.8 (c = 2.61, CH_2Cl_2).

1-((2R,3R,4R,5S,6R)-2-(allyloxy)-4,5-bis(benzyloxy)-6-((benzyloxy)methyl)tetrahydro-2H-pyran-3-yl)propan-2-one

The title product compound is prepared using **Method E** and isolated by flash column chromatography (20:1-9:1 Pentane: Ethyl Acetate) giving a pale yellow syrup (23 mg, 0.043 mmol, 43% yield).

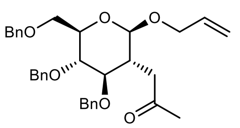

23b

^{1}H NMR (500 MHz, CD_2Cl_2) δ 7.39-7.21 (m, 15 H), 5.93-5.84 (m, 1 H), 5.24 (d, *J* = 17.2 Hz, 1 H), 5.16 (d, *J* = 10.4 Hz, 1 H), 4.89 (d, *J* = 11.2 Hz, 1 H), 4.79 (d, *J* = 10.9 Hz, 1 H), 4.67-4.60 (m, 2 H), 4.55 (d, *J* = 11.7 Hz, 2 H), 4.34 (d, *J* = 8.7 Hz, 1 H), 4.32-4.29 (m, 1 H), 3.99 (dd, *J* = 12.8, 6.1 Hz, 1 H), 3.79-3.76 (m, 2 H), 3.65 (t, *J* = 9.2 Hz, 1 H), 3.48-3.43 (m, 2 H), 2.52-2.43 (m, 2 H), 2.23-2.17 (m, 1 H), 2.04 (s, 3 H) ppm; ^{13}C NMR (126 MHz, CD_2Cl_2) δ 207.74, 138.99, 138.91, 138.87, 134.64, 128.85 (*three carbons*), 128.32 (*two carbons*), 128.29, 128.18, 128.12, 128.10, 117.42, 102.46, 82.96, 80.24, 75.50, 75.17, 75.09, 73.88, 70.47, 69.52, 45.19, 42.34, 30.13 ppm. MS (ESI): 553.2 (M+Na); HRMS (ESI): Calculated for $C_{33}H_{38}O_6Na$ (M+Na): 553.25606, Found: 553.25543. $[\alpha]_D^{20}$ = -2.2 (c = 1.90, CH_2Cl_2).

((2R,3S,4R,5R,6R)-3,4-bis(benzyloxy)-6-(((3aR,4R,6R,6aR)-6-methoxy-2,2-dimethyltetrahydrofuro[3,4-d][1,3]dioxol-4-yl)methoxy)-5-(2-oxopropyl)tetrahydro-2H-pyran-2-yl)methyl acetate

The title product compound is prepared using **Method E** and isolated by flash column chromatography (9:1-3:1 Pentane: Ethyl Acetate) giving a pale yellow syrup (25 mg, 0.04 mmol, 40% yield).

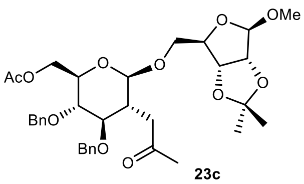

23c

^{1}H NMR (500 MHz, CD_2Cl_2) δ 7.35-7.27 (m, 10 H), 4.90-4.87 (m, 2 H), 4.82 (d, *J* = 10.9 Hz, 1 H), 4.63 (d, *J* = 6.0 Hz, 1 H), 4.60-4.41 (m, 3 H), 4.40 (d, *J* = 8.7 Hz, 1 H), 4.33 (dd, *J* = 11.8, 2.1 Hz, 1 H), 4.26 (dd, *J* = 11.9, 4.4 Hz, 1 H), 4.16 (dd, *J* = 8.6, 6.1 Hz, 1 H), 3.69 (dd, *J* = 10.3, 8.7 Hz, 1 H), 3.56-3.48 (m, 4 H), 3.26 (s, 3 H), 2.52 (d, *J* = 5.6 Hz, 2 H), 2.18-2.13 (m, 1 H), 2.06 (s, 3 H), 2.03 (s, 3 H), 1.60 (s, 3 H), 1.43 (s, 3 H), 1.29 (s, 3 H), 1.26 (s, 3 H) ppm; ^{13}C NMR (126 MHz, CD_2Cl_2) δ 207.57, 171.12, 138.82, 138.48, 128.95, 128.92, 128.45, 128.40, 128.32, 128.23, 112.70, 109.80, 102.96, 85.57, 85.40, 82.65, 82.46, 80.13, 75.26, 73.70, 70.50, 63.40, 55.17, 44.90, 41.57, 30.23, 30.18, 26.69, 25.12, 21.21 ppm. MS (ESI): 651.2 (M+Na); HRMS (ESI): Calculated for $C_{34}H_{44}O_{11}Na$ (M+Na): 651.27758, Found: 651.27703. $[\alpha]_D^{20}$ = -14.3 (c = 1.60, CH_2Cl_2).

((2R,3aR,4R,5S,6R,7aR)-4,5-bis(benzyloxy)-2-(((3aR,4R,6R,6aR)-6-methoxy-2,2-dimethyltetrahydrofuro[3,4-d][1,3]dioxol-4-yl)methoxy)-2-methylhexahydro-4H-furo[2,3-b]pyran-6-yl)methyl acetate

The title product compound is prepared using **Method E** and isolated by flash column chromatography (9:1-3:1 Pentane: Ethyl Acetate) giving a pale yellow syrup (8.1 mg, 0.013 mmol, 13% yield).

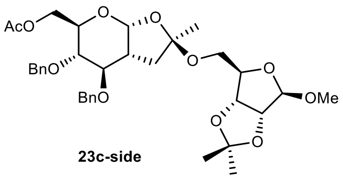

^{1}H NMR (700 MHz, CD_2Cl_2) δ 7.37-7.29 (m, 10 H), 5.45 (d, *J* = 5.9 Hz, 1 H), 4.91 (s, 1 H), 4.69 (d, *J* = 11.7 Hz, 1 H), 4.65 (t, *J* = 12.1 Hz, 2 H), 4.57-4.54 (m, 2 H), 4.46 (d, *J* = 11.3 Hz, 1 H), 4.25 (dd, *J* = 11.9, 2.3 Hz, 1 H), 4.18-4.14 (m, 2 H), 3.81 (ddd, *J* = 8.3, 5.6, 2.3 Hz, 1 H), 3.63 (t, *J* = 4.8 Hz, 1 H), 3.53 (dd, *J* = 9.0, 4.2 Hz, 1 H), 3.50-3.43 (m, 2 H), 3.29 (s, 3 H), 2.78-2.73 (m, 1 H), 2.11 (dd, *J* = 13.4, 8.4 Hz, 1 H), 2.04 (s, 3 H), 1.95 (dd, *J* = 13.4, 8.0 Hz, 1 H), 1.44 (s, 3 H), 1.43 (s, 3 H) ppm; ^{13}C NMR (176 MHz, CD_2Cl_2) δ 171.19, 138.83, 138.38, 129.01, 128.98, 128.60, 128.54, 128.47, 128.41, 112.72, 109.84, 106.43, 101.16, 86.21, 85.77, 82.88, 78.10, 77.42, 73.38, 72.93, 70.13, 64.54, 63.16, 55.23, 41.51, 40.74, 26.83, 25.32, 22.83, 21.22 ppm. MS (ESI): 651.2 (M+Na); HRMS (ESI): Calculated for $C_{34}H_{44}O_{11}Na$ (M+Na): 651.27758, Found: 651.27785. $[\alpha]_D^{20}$ = -0.9 (c = 0.80, CH_2Cl_2).

((2R,3S,4R,5R,6S)-6-(((3R,5R,7R)-adamantan-1-yl)oxy)-3,4-bis(benzyloxy)-5-(2-oxopropyl)tetrahydro-2H-pyran-2-yl)methyl acetate

The title product compound is prepared using **Method E** and isolated by flash column chromatography (20:1-9:1 Pentane: Ethyl Acetate) giving a pale yellow syrup (29 mg, 0.05 mmol, 50% yield).

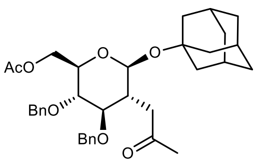

^{1}H NMR (500 MHz, CD_2Cl_2) δ 7.33-7.28 (m, 10 H), 4.87 (d, *J* = 11.1 Hz, 1 H), 4.82 (d, *J* = 10.9 Hz, 1 H), 4.63 (d, *J* = 8.6 Hz, 1 H), 4.57 (d, *J* = 11.3 Hz, 1 H), 4.54 (d, *J* = 11.0 Hz, 1 H), 4.27-4.27 (m, 2 H), 3.57-3.48 (m, 2 H), 2.50-2.40 (m, 2 H), 2.11-2.09 (m, 4 H), 2.04 (s, 3 H), 2.03 (s, 3 H), 1.78-1.74 (m, 3 H), 1.68-1.59 (m, 9 H) ppm; ^{13}C NMR (126 MHz, CD_2Cl_2) δ 207.81, 171.08, 138.90, 138.58, 128.93, 128.89, 128.42, 128.35, 128.32, 128.17, 96.07, 83.15, 80.53, 75.55, 75.21, 75.19, 73.19, 63.85, 45.53, 42.91, 42.44, 36.70, 31.34, 29.90, 21.23 ppm. MS (ESI): 599.2 (M+Na); HRMS (ESI): Calculated for $C_{35}H_{44}O_7Na$ (M+Na): 599.29792, Found: 599.29761. $[\alpha]_D^{20}$ = +7.0 (c = 1.98, CH_2Cl_2).

((2R,3aR,4R,5S,6R,7aR)-4,5-bis(benzyloxy)-2-(((3aR,5R,6S,6aR)-5-((S)-2,2-dimethyl-1,3-dioxolan-4-yl)-2,2-dimethyltetrahydrofuro[2,3-d][1,3]dioxol-6-yl)oxy)-2-methylhexahydro-4H-furo[2,3-b]pyran-6-yl)methyl acetate

The title product compound is prepared using **Method F** and isolated by flash column chromatography (9:1-3:1 Pentane: Ethyl Acetate) giving a pale yellow syrup (48 mg, 0.07 mmol, 70% yield)

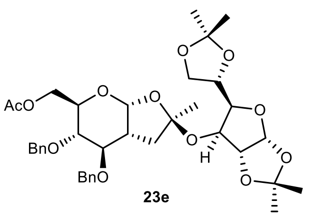

^{1}H NMR (500 MHz, CD_2Cl_2) δ 7.37-7.27 (m, 10 H), 5.79 (d, *J* = 3.7 Hz, 1 H), 5.65 (d, *J* = 6.2 Hz, 1 H), 4.64-4.58 (m, 3 H), 4.47 (d, *J* = 3.8 Hz, 1 H), 4.41 (d, *J* = 11.4 Hz, 1 H), 4.39 (d, *J* = 3.5 Hz, 1 H), 4.24-4.12 (m, 3 H), 4.09-4.05 (m, 2 H), 3.90 (dd, *J* = 8.4, 6.0 Hz, 1 H), 3.77-3.73 (m, 1 H), 3.66 (t, *J* = 3.6 Hz, 1 H), 3.53-3.50 (m, 1 H),

2.86-2.83 (m, 1 H), 2.11 (d, *J* = 13.5, 8.6 Hz, 1 H), 2.03 (s, 3 H), 1.98 (d, *J* = 13.4, 9.6 Hz, 1 H), 1.54 (s, 3 H), 1.47 (s, 3 H), 1.38 (s, 3 H), 1.31 (s, 3 H), 1.28 (s, 3 H) ppm; ^{13}C NMR (126 MHz, CD_2Cl_2) δ 171.18, 138.66, 138.24, 128.95, 128.94, 128.50, 128.47, 128.46, 128.36, 112.11, 109.73, 107.67, 105.54, 101.48, 85.56, 81.97, 77.02, 76.70, 74.47, 72.78, 72.64, 72.25, 69.56, 68.19, 64.75, 40.72, 40.34, 27.10, 26.96, 26.53, 25.67, 24.64, 21.19 ppm. MS (ESI): 707.2 (M+Na); HRMS (ESI): Calculated for $C_{37}H_{48}O_{12}Na$ (M+Na): 707.30380, Found: 707.30372. $[\alpha]_D^{20}$ = -4.2 (c = 3.60, CH_2Cl_2).

((2R,3S,4R,5R,6R)-6-(1H-benzo[d][1,2,3]triazol-1-yl)-3,4-bis(benzyloxy)-5-(2-oxopropyl)tetrahydro-2H-pyran-2-yl)methyl acetate

The title product compound is prepared using **Method E** and isolated by flash column chromatography (9:1-3:1 Pentane: Ethyl Acetate) giving a pale yellow syrup (48 mg, 0.088 mmol, 88% yield).

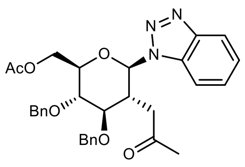

23f

^{1}H NMR (500 MHz, CD_2Cl_2) δ 8.03 (d, *J* = 8.4 Hz, 1 H), 7.73 (d, *J* = 8.4 Hz, 1 H), 7.54 (t, *J* = 7.6 Hz, 1 H), 7.41 (t, *J* = 7.6 Hz, 1 H), 7.37-7.30 (m, 10 H), 6.28 (d, *J* = 10.4 Hz, 1 H), 5.01 (d, *J* = 11.4 Hz, 1 H), 4.94 (d, *J* = 10.8 Hz, 1 H), 4.71 (d, *J* = 10.8 Hz, 1 H), 4.66 (d, *J* = 11.4 Hz, 1 H), 4.43 (dd, *J* = 12.2, 1.8 Hz, 1 H), 4.28 (dd, *J* = 12.1, 4.7 Hz, 1 H), 4.00-3.92 (m, 2 H), 3.83 (t, *J* = 9.2 Hz, 1 H), 3.09-3.03 (m, 1 H), 2.53 (dd, *J* = 18.2, 4.7 Hz, 1 H), 2.20 (dd, *J* = 18.2, 4.7 Hz, 1 H), 2.04 (s, 3 H), 1.71 (s, 3 H) ppm; ^{13}C NMR (126 MHz, CD_2Cl_2) δ 206.17, 170.92, 146.94, 138.59, 138.24, 132.58, 129.01 (*two carbons*), 128.52 (*two carbons*), 128.38, 128.36, 128.34, 124.95, 120.39, 111.59, 87.41, 82.22, 79.83, 76.67, 75.61, 75.50, 63.19, 42.82, 39.84, 30.23, 21.15 ppm. MS (ESI): 566.2 (M+Na); HRMS (ESI): Calculated for $C_{31}H_{33}O_6N_3Na$ (M+Na): 566.22616, Found: 566.22598. $[\alpha]_D^{20}$ = +4.10 (c = 3.0, CH_2Cl_2).

1-((2R,3R,4R,5R,6R)-2-(1H-benzo[d][1,2,3]triazol-1-yl)-4,5-bis(benzyloxy)-6-((benzyloxy)methyl)tetrahydro-2*H*-pyran-3-yl)propan-2-one

The title product compound is prepared using **Method E** and isolated by flash column chromatography (9:1-1:1 Pentane: Ethyl Acetate) giving a pale yellow syrup (32 mg, 0.054 mmol, 54% yield).

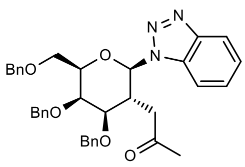

23g

^{1}H NMR (500 MHz, CD_2Cl_2) δ 8.00-7.98 (m, 1 H), 7.83-7.80 (m, 1 H), 7.43-7.28 (m, 17 H), 6.22 (d, *J* = 10.3 Hz, 1 H), 4.99 (d, *J* = 11.1 Hz, 1 H), 4.79 (d, *J* = 11.4 Hz, 1 H), 4.67 (d, *J* = 11.1 Hz, 1 H), 4.53-4.40 (m, 3 H), 4.17 (s, 1 H), 4.00-3.97 (m, 1 H), 3.89 (dd, *J* = 11.1, 2.1 Hz, 1 H), 3.74 (dd, *J* = 9.3, 7.2 Hz, 1 H), 3.64 (dd, *J* = 9.3, 5.8 Hz, 1 H), 3.45-3.10 (m, 1 H), 2.45 (dd, *J* = 17.7, 4.7 Hz, 1 H), 2.19 (dd, *J* = 17.6, 5.0 Hz, 1 H), 1.78 (s, 3 H) ppm; ^{13}C NMR (126 MHz, CD_2Cl_2) δ 206.51, 147.06, 139.34, 138.54, 138.21, 132.35, 129.02, 128.89, 128.87, 128.45 (*two carbons*), 128.36, 128.28, 128.19, 128.11, 124.79, 120.14, 112.52, 88.41, 80.48, 76.60, 75.08, 73.96, 71.96, 71.42, 69.32, 40.11, 37.90, 30.40 ppm. MS (ESI): 614.2 (M+Na); HRMS (ESI): Calculated for $C_{36}H_{37}O_5N_3Na$ (M+Na): 614.26254, Found: 614.26252. $[\alpha]_D^{20}$ = +3.3 (c = 1.0, CH_2Cl_2).

1-*O*-Methyl-2,3-*O*-isopropylidene-5-(deoxy-(4-(3,5-*O*-di-benzyl-2-C-acetylmethyl-α-D-lyxofuranosyl)methyl-1*H*-1,2,3-triazol-1-yl)-α-D-lyxofuranoside

The title product compound is prepared using **Method G** and isolated by flash column chromatography (3:1-1:1 Pentane: Ethyl Acetate) giving a pale yellow syrup (91.2 mg, 0.14 mmol, 72% yield, α/β ratio 92:8)

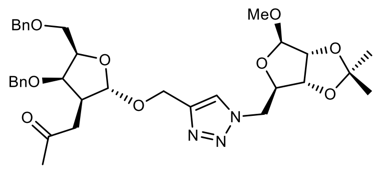

25a

^{1}H NMR (500 MHz, Acetone-d_6) δ 8.04 (s, 1 H), 7.40-7.26 (m, 10 H), 4.98-4.96 (m, 2 H), 4.84 (d, *J* = 6.0 Hz, 1 H), 4.77 (dd, *J* = 12.3, 3.7 Hz, 1 H), 4.69 (d, *J* = 6.0 Hz, 1 H), 4.61-4.54 (m, 6 H), 4.49-4.48 (m, 1 H), 4.37-4.27 (m, 3 H), 3.82 (dd, *J* = 9.2, 6.9 Hz, 1 H), 3.70-3.67 (m, 1 H), 3.35 (s, 3 H), 2.82 (dd, *J* = 17.6, 9.7 Hz, 1 H), 2.67-2.62 (m, 1 H), 2.53 (dd, *J* = 17.6, 5.0 Hz, 1 H), 2.01 (s, 3 H), 1.39 (s, 3 H), 1.27 (s, 3 H) ppm; ^{13}C NMR (126 MHz, Acetone-d_6) δ 207.30, 145.66, 139.57, 139.48, 129.18 (two carbons), 128.71, 128.63, 128.45, 128.36, 124.55, 113.05, 110.79, 107.72, 86.24, 85.96, 82.65, 80.90, 80.83, 74.84, 73.87, 69.42, 62.22, 55.58, 53.61, 46.57, 40.21, 30.07, 26.74, 25.07 ppm. MS (ESI): 638.4 (M+H); HRMS (ESI): Calculated for $C_{34}H_{44}O_9N_3$ (M+H): 638.30721, Found: 638.30673. $[\alpha]_D^{20}$ = +29.9 (c = 0.72, CH_2Cl_2).

1-*O*-Methyl-2,3-*O*-isopropylidene-5-(deoxy-(4-(3,5-*O*-di-benzyl-2-C-acetylmethyl-α-D-lyxofuranosyl)ethyl-1*H*-1,2,3-triazol-1-yl)-α-D-lyxofuranoside

The title product compound is prepared using **Method H** and isolated by flash column chromatography (3:1-1:1 Pentane: Ethyl Acetate) giving a pale yellow syrup (103.0 mg, 0.16 mmol, 79% yield, α/β ratio 86:14)

(*Notice*: *Diastereomer separation was challenging, there is still about 19% diastereomer in the title compound after five repetitions of flash column chromatography, CH_2Cl_2/MeOH and Pentane/Acetone were also tried as the eluent but without better result.*)

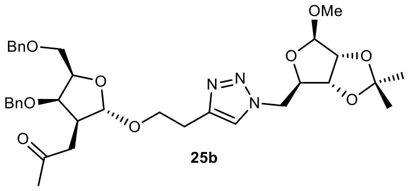

25b

^{1}H NMR (500 MHz, Acetone-d_6) δ 7.88 (s, 1 H), 7.39-7.26 (m, 10 H), 4.95 (s, 1 H), 4.84 (dd, *J* = 8.0, 5.2 Hz, 2 H), 4.69 (d, *J* = 6.0 Hz, 1 H), 4.60-4.41 (m, 6 H), 4.34-4.22 (m, 3 H), 3.94-3.89 (m, 1 H), 3.82-3.77 (m, 1 H), 3.69-3.62 (m, 2 H), 3.35 (s, 3 H), 2.95-2.92 (m, 2 H), 2.86-2.81 (m, 1 H), 2.66-2.62 (m, 1 H), 2.54 (dd, *J* = 17.7, 5.2 Hz, 1 H), 2.02 (s, 3 H), 1.38 (s, 3 H), 1.27 (s, 3 H) ppm; ^{13}C NMR (126 MHz, Acetone-d_6) δ 207.44, 145.64, 139.57, 139.50, 129.15 (two carbons), 128.69, 128.60, 128.42, 128.33, 123.35, 113.00, 110.78, 108.37, 86.30, 85.97, 82.68, 80.81, 80.76, 74.81, 73.84, 69.45, 67.99, 55.53, 53.49, 46.57, 40.31, 30.07, 27.32, 26.71, 25.02 ppm. MS (ESI): 652.4 (M+H); HRMS (ESI): Calculated for $C_{35}H_{46}O_9N_3$ (M+H): 652.32286, Found: 652.32183. $[\alpha]_D^{20}$ = +6.3 (c = 0.50, CH_2Cl_2).

1-*O*-Methyl-2,3-*O*-isopropylidene-5-(deoxy-(4-(3,5-*O*-di-benzyl-2-C-acetylmethyl-α-D-lyxofuranosyl)propyl-1*H*-1,2,3-triazol-1-yl)-α-D-lyxofuranoside

The title product compound is prepared using **Method H** and isolated by flash column chromatography (3:1-1:1 Pentane: Ethyl Acetate) giving a pale yellow syrup (91 mg, 0.14 mmol, 68% yield, α/β ratio 88:12)

(*Notice*: *Diastereomeric separation was challenging, there is still about 5% diastereomer in the title compound after three repetitions of flash column chromatography, CH_2Cl_2/MeOH was also tried as the eluent but without better result.*)

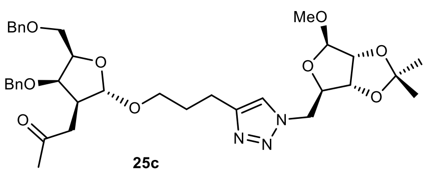

25c

^{1}H NMR (500 MHz, Acetone-*d*$_6$) δ 7.82 (s, 1 H), 7.37-7.27 (m, 10 H), 4.95 (s, 1 H), 4.81 (dd, *J* = 10.0, 5.3 Hz, 2 H), 4.68 (d, *J* = 5.8 Hz, 1 H), 4.61-4.54 (m, 3 H), 4.53-4.47 (m, 2 H), 4.46-4.14 (m, 1 H), 4.35-4.32 (m, 2 H), 4.30-4.27 (m, 1 H), 3.80 (dd, *J* = 9.8, 6.3 Hz, 1 H), 3.71-3.63 (m, 2 H), 3.44-3.40 (m, 1 H), 3.35 (s, 3 H), 2.86-2.81 (m, 1 H), 2.76-2.72 (m, 2 H), 2.67-2.63 (m, 1 H), 2.58 (dd, *J* = 17.4, 5.3 Hz, 1 H), 2.04 (s, 3 H), 1.91-1.87 (m, 2 H), 1.38 (s, 3 H), 1.26 (s, 3 H) ppm; ^{13}C NMR (126 MHz, Acetone-*d*$_6$) δ 207.42, 147.91, 139.61, 139.55, 129.17, 129.16, 128.70, 128.59, 128.42, 128.33, 122.68, 113.01, 110.77, 108.38, 86.32, 85.99, 82.69, 80.87, 80.68, 74.80, 73.83, 69.52, 67.96, 55.51, 53.46, 46.56, 40.38, 30.38, 30.07, 26.71, 25.02, 22.89 ppm. MS (ESI): 666.2 (M+H; HRMS (ESI): Calculated for $C_{36}H_{48}O_9N_3$ (M+H): 666.33851, Found: 666.33754. $[\alpha]_D^{20}$ = +14.1 (c = 0.53, CH_2Cl_2).

N-(1-(2-O-(3,5-O-di-benzyl-2-C-acetylmethyl)-α-D-lyxofuranosyl)ethyl)-1H-1,2,3-triazol-4-yl)methyl-1H-indole-2,3-dione

The title product compound is prepared using **Method H** and isolated by flash column chromatography (3:1-1:1 Pentane: Ethyl Acetate) giving a pale yellow syrup (63.0 mg, 0.10 mmol, 50% yield, α/β ratio 82:18)

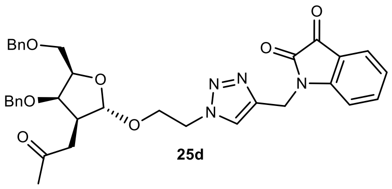

25d

^{1}H NMR (500 MHz, Acetone-*d*$_6$) δ 8.05 (s, 1 H), 7.66-7.62 (m, 1 H), 7.55 (d, *J* = 7.5 Hz, 1 H), 7.36-7.21 (m, 11 H), 7.14 (t, *J* = 7.6 Hz, 1 H), 5.03-5.00 (m, 2 H), 4.77 (d, *J* = 4.2 Hz, 1 H), 4.57-4.54 (m, 2 H), 4.53-4.49 (m, 3 H), 4.30 (d, *J* = 11.3 Hz, 1H), 4.18-4.14 (m, 1 H), 4.10-4.08 (m, 1 H), 4.00-3.97 (m, 1 H), 3.84-3.81 (m, 1 H), 3.71 (dd, *J* = 9.8, 5.9 Hz, 1 H), 3.57 (dd, *J* = 9.8, 6.2 Hz, 1 H), 2.75 (dd, *J* = 17.5, 9.2 Hz, 1 H), 2.55-2.52 (m, 1 H), 2.47 (dd, *J* = 17.5, 5.5 Hz, 1 H), 2.00 (s, 3 H) ppm; ^{13}C NMR (126 MHz, Acetone-*d*$_6$) δ 207.36, 184.24, 158.66, 151.77, 142.49, 139.56, 139.47, 139.01, 129.19, 129.17, 128.75, 128.66, 128.48, 128.35, 125.22, 124.83, 124.37, 118.78, 112.30, 108.51, 80.94, 80.70, 74.83, 73.80, 69.37, 67.45, 51.00, 46.58, 40.18, 36.16, 30.07 ppm. MS (ESI): 647.2 (M+Na); HRMS (ESI): Calculated for $C_{35}H_{36}O_7N_4Na$ (M+Na): 647.24762, Found: 647.24717. $[\alpha]_D^{20}$ = +32.4 (c = 0.30, CH_2Cl_2).

(1-(2-O-(3,5-O-di-benzyl-2-C-acetylmethyl)-α-D-lyxofuranosyl)ethyl)-1H-1,2,3-triazol-4-yl)ethoxy-2H-benzopyran-2-one

The title product compound is prepared using **Method H** and isolated by flash column chromatography (3:1-1:1 Pentane: Ethyl Acetate) giving a white solid (64.3 mg, 0.10 mmol, 50% yield, α/β ratio 87:13)

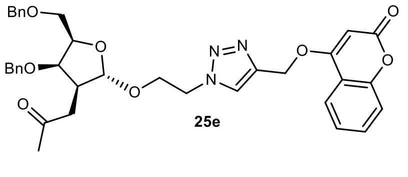

25e

^{1}H NMR (500 MHz, Acetone-*d*$_6$) δ 8.32 (s, 1 H), 7.78 (dd, *J* = 7.9, 1.3 Hz, 1 H), 7.62-7.60 (m, 1 H), 7.34-7.26 (m, 12 H), 6.03 (s, 1 H), 5.42 (d, *J* = 2.6 Hz, 2 H), 4.85 (d, *J* = 4.3 Hz, 1 H), 4.67-4.64 (m, 2 H), 4.54-4.50 (m, 3 H), 4.31 (d, *J* = 11.3

Hz, 1 H), 4.26-4.22 (m, 2 H), 4.08-4.05 (m, 1 H), 3.93-3.91 (m, 1 H), 3.77 (dd, *J* = 9.8, 5.6 Hz, 1 H), 3.63-3.61 (m, 1 H), 2.81 (dd, *J* = 17.7, 9.3 Hz, 1 H), 2.64-2.62 (m, 1 H), 2.53 (dd, *J* = 17.7, 5.3 Hz, 1 H), 1.99 (s, 3 H) ppm; ^{13}C NMR (126 MHz, Acetone-d_6) δ 207.32, 165.54, 162.25, 154.38, 142.10, 139.53, 139.44, 133.43, 129.18, 129.17, 128.72, 128.61, 128.47, 128.36, 126.41, 124.82, 123.97, 117.33, 116.57, 108.62, 92.09, 81.03, 80.75, 74.84, 73.87, 69.47, 67.52, 63.85, 51.10, 46.66, 40.23, 30.07 ppm. MS (ESI): 662.2 (M+Na); HRMS (ESI): Calculated for $C_{36}H_{37}O_8N_3Na$ (M+Na): 662.24729, Found: 662.24776. $[\alpha]_D^{20}$ = +25.8 (c = 0.27, CH_2Cl_2). M.P.: 98-100 ℃.

1-*O*-Methyl-2,3-*O*-isopropylidene-5-(deoxy-(5-(3,5-*O*-di-benzyl-2-C-acetylmethyl-α-D-lyxofuranosyl)methyl-1*H*-1,2,3-triazol-1-yl)-α-D-lyxofuranoside

The title product compound is prepared using **Method G** and isolated by flash column chromatography (3:1-1:1 Pentane: Ethyl Acetate) giving a pale yellow syrup (100 mg, 0.16 mmol, 78% yield, α/β ratio 87:13)

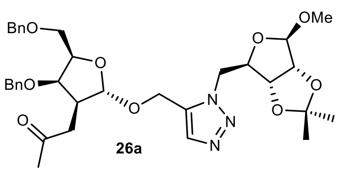

^{1}H NMR (500 MHz, Acetone-d_6) δ 7.67 (s, 1 H), 7.40-7.26 (m, 10 H), 4.98-4.95 (m, 2 H), 4.92 (d, *J* = 6.0 Hz, 1 H), 4.89 (d, *J* = 12.6 Hz, 1 H), 4.70 (d, *J* = 2.3 Hz, 1 H), 4.68 (d, *J* = 4.3 Hz, 1 H), 4.65-4.61 (m, 1 H), 4.59-4.52 (m, 5 H), 4.46-4.42 (m, 1 H), 4.35-4.31 (m, 2 H), 3.83 (dd, *J* = 9.7, 6.4 Hz, 1 H), 3.70 (dd, *J* = 9.7, 5.9 Hz, 1 H), 3.39 (s, 3 H), 2.87 (dd, *J* = 17.7, 9.6 Hz, 1 H), 2.72-2.69 (m, 1 H), 2.62 (dd, *J* = 17.7, 4.9 Hz, 1 H), 2.02 (s, 3 H), 1.37 (s, 3 H), 1.25 (s, 3 H) ppm; ^{13}C NMR (126 MHz, Acetone-d_6) δ 207.15, 139.55, 139.45, 134.65, 134.42, 129.19, 129.18, 128.73, 128.62, 128.47, 128.38, 112.97, 110.77, 107.87, 86.01, 85.88, 82.70, 81.27, 80.77, 74.93, 73.88, 69.25, 58.43, 55.54, 51.67, 46.64, 40.14, 30.07, 26.73, 25.10 ppm. MS (ESI): 638.2 (M+H); HRMS (ESI): Calculated for $C_{34}H_{44}O_9N_3$ (M+H): 638.30721, Found: 638.30618. $[\alpha]_D^{20}$ = +14.8 (c = 0.40, CH_2Cl_2).

1-*O*-Methyl-2,3-*O*-isopropylidene-5-(deoxy-(5-(3,5-*O*-di-benzyl-2-C-acetylmethyl-α-D-lyxofuranosyl)methyl-1*H*-1,2,3-triazol-1-yl)-β-D-ribofuranoside

The title product compound is prepared using **Method G** and isolated by flash column chromatography (3:1-1:1 Pentane: Ethyl Acetate) giving a pale yellow syrup (96.3 mg, 0.15 mmol, 75% yield, β/α ratio 83:17)

(*Notice*: *Diastereomer separation was challenging, there is still about 22% diastereomer in the title compound after three repetitions of flash column chromatography, CH_2Cl_2/MeOH was also tried as the eluent but without better result.*)

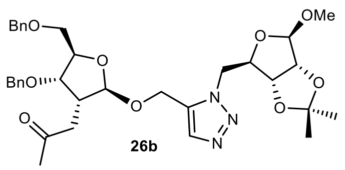

^{1}H NMR (500 MHz, Acetone-d_6) δ 7.60 (s, 1 H), 7.40-7.27 (m, 10 H), 4.97-4.96 (m, 2 H), 4.88-4.83 (m, 2 H), 4.70-4.67 (m, 1 H), 4.63-4.61 (m, 1 H), 4.60-4.56 (m, 3 H), 4.52 (d, *J* = 11.6 Hz, 1 H), 4.49-4.46 (m, 2 H), 4.43-4.40 (m, 1 H), 4.27-4.24 (m, 1 H), 4.22-4.20 (m, 1 H), 3.62 (dd, *J* = 10.2, 5.2 Hz, 1 H), 3.56 (dd, *J* = 10.2, 5.6 Hz, 1 H), 3.40 (s, 3 H), 2.93-2.84 (m, 2 H), 2.51 (dd, *J* = 16.9, 5.5 Hz, 1 H), 2.08 (d, *J* = 2.3 Hz, 3 H), 1.37 (s, 3 H), 1.26 (s, 3 H) ppm; ^{13}C NMR (126 MHz, Acetone-d_6) δ 206.83, 139.65, 139.28, 134.60, 134.37, 129.20, 129.15, 128.70, 128.51, 128.47, 128.38, 113.00, 110.80,

108.55, 86.02, 85.92, 82.84, 82.70, 80.88, 73.69, 72.80, 72.46, 58.18, 55.59, 51.72, 43.96, 39.35, 30.23, 26.79, 25.14 ppm. MS (ESI): 638.2 (M+H); HRMS (ESI): Calculated for $C_{34}H_{44}O_9N_3$ (M+H): 638.30721, Found: 638.30708. $[\alpha]_D^{20}$ = -36.7 (c = 0.41, CH_2Cl_2).

1-*O*-Methyl-2,3-*O*-isopropylidene-5-(deoxy-(5-(3,5-*O*-di-benzyl-2-C-acetylmethyl-α-D-lyxofuranosyl)methyl-1*H*-1,2,3-triazol-1-yl)-α-L-lyxofuranoside

The title product compound is prepared using **Method G** and isolated by flash column chromatography (3:1-1:1 Pentane: Ethyl Acetate) giving a pale yellow syrup (77.1 mg, 0.12 mmol, 62%yield, α/β ratio 88:12)

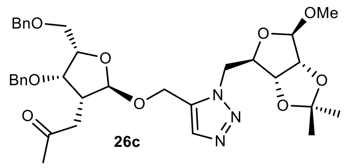

^{1}H NMR (500 MHz, Acetone-d_6) δ 7.66 (s, 1 H), 7.38-7.27 (m, 10 H), 4.98-4.96 (m, 2 H), 4.91 (d, *J* = 6.0 Hz, 1 H), 4.87 (d, *J* = 12.7 Hz, 1 H), 4.71-4.68 (m, 2 H), 4.65-4.53 (m, 6 H), 4.45-4.41 (m, 1 H), 4.34 (d, *J* = 11.3 Hz, 1 H), 4.31-4.30 (m, 1 H), 3.82 (dd, *J* = 9.6, 6.6 Hz, 1 H), 3.69 (dd, *J* = 9.7, 5.9 Hz, 1 H), 3.40 (s, 3 H), 2.90-2.84 (m, 1 H), 2.72-2.62 (m, 2 H), 2.02 (s, 3 H), 1.37 (s, 3 H), 1.25 (s, 3 H) ppm; ^{13}C NMR (126 MHz, Acetone-d_6) δ 207.14, 139.58, 139.47, 134.66, 134.46, 129.20 (two carbons), 128.75, 128.63, 128.49, 128.39, 113.00, 110.77, 108.02, 86.05, 85.97, 82.67, 81.37, 80.78, 74.92, 73.89, 69.24, 58.52, 55.54, 51.71, 46.73, 40.03, 29.77, 26.77, 25.10 ppm. MS (ESI): 638.2 (M+H); HRMS (ESI): Calculated for $C_{34}H_{44}O_9N_3$ (M+H): 638.30721, Found: 638.30633. $[\alpha]_D^{20}$ = -82.0 (c = 0.96, CH_2Cl_2).

1-Deoxy-1-(5-(2-*O*-(3,5-*O*-di-benzyl-2-*C*-acetylmethyl)-α-D-lyxofuranosyl)methyl)-1*H*-1,2,3-triazol-1-yl)-2,3,4,6-tetra-acetyl-α-D-glucopyranoside

The title product compound is prepared using **Method G** and isolated by flash column chromatography (3:1-1:1 Pentane: Ethyl Acetate) giving a pale yellow syrup (64.5 mg, 0.08 mmol, 41% yield, α/β ratio 88:12)

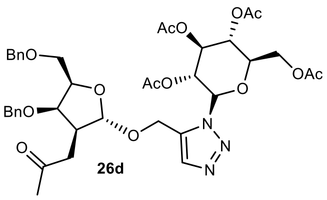

^{1}H NMR (500 MHz, Acetone-d_6) δ 7.69 (s, 1 H), 7.39-7.37 (m, 2 H), 7.36-7.31 (m, 4 H), 7.30-7.25 (m, 4 H), 6.27 (d, *J* = 9.3 Hz, 1 H), 5.89 (t, *J* = 9.4 Hz, 1 H), 5.55 (t, *J* = 9.5 Hz, 1 H), 5.30 (t, *J* = 9.9 Hz, 1 H), 5.00-4.96 (m, 2 H), 4.72 (d, *J* = 12.6 Hz, 1 H), 4.62 (d, *J* = 11.9 Hz, 1 H), 4.57 (d, *J* = 11.2 Hz, 2 H), 4.52-4.48 (m, 1 H), 4.39-4.32 (m, 3 H), 4.26 (dd, *J* = 12.6, 4.8 Hz, 1 H), 4.18 (dd, *J* = 12.6, 2.3 Hz, 1 H), 3.82 (dd, *J* = 9.5, 6.6 Hz, 1 H), 3.76 (dd, *J* = 9.5, 5.8 Hz, 1 H), 2.86 (dd, *J* = 17.4, 9.2 Hz, 1 H), 2.80-2.74 (m, 1 H), 2.63 (dd, *J* = 17.5, 5.3 Hz, 1 H), 2.05 (d, *J* = 1.7 Hz, 3 H), 2.03 (s, 3 H), 2.00 (s, 3 H), 1.95 (s, 3 H), 1.77 (s, 3 H) ppm; ^{13}C NMR (126 MHz, Acetone-d_6) δ 207.62, 170.79, 170.52, 170.08, 169.11, 139.56, 139.51, 135.85, 135.25, 129.20 (two carbons), 128.79, 128.73, 128.49, 128.40, 108.28, 85.24, 81.35, 80.90, 75.21, 74.92, 74.02, 73.91, 71.11, 69.14, 68.64, 62.48, 58.49, 46.58, 40.26, 30.07, 20.71, 20.70, 20.62, 20.35 ppm. MS (ESI): 804.3 (M+Na); HRMS (ESI): Calculated for $C_{39}H_{48}O_{14}N_3$ (M+H): 782.31308, Found: 782.31342. $[\alpha]_D^{20}$ = +25.2 (c = 0.10, CH_2Cl_2).

1-Deoxy-1-(5-(3-*O*-(3,5-*O*-di-benzyl-2-*C*-acetylmethyl)-α-D-lyxofuranosyl)propyl)-1*H*-1,2,3-triazol-1-yl)-2,3,4,6-tetra-acetyl-α-D-glucopyranoside

The title product compound is prepared using **Method G** and isolated by flash column chromatography (3:1-1:1 Pentane: Ethyl Acetate) giving a pale yellow syrup (121.0 mg, 0.15 mmol, 75% yield, α/β ratio 95:5)

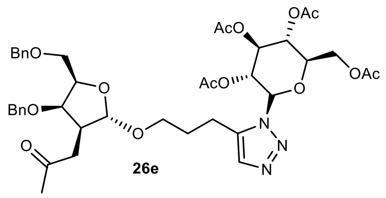

^{1}H NMR (500 MHz, Acetone-d_6) δ 7.55 (s, 1 H), 7.38-7.27 (m, 10 H), 6.18 (d, *J* = 9.3 Hz, 1 H), 5.89 (t, *J* = 9.5 Hz, 1 H), 5.54 (t, *J* = 9.5 Hz, 1 H), 5.27 (t, *J* = 9.8 Hz, 1 H), 4.84 (d, *J* = 4.4 Hz, 1 H), 4.58-4.55 (m, 3 H), 4.37-4.31 (m, 3 H), 4.30-4.29 (m, 1 H), 4.26 (dd, *J* = 12.5, 5.3 Hz, 1 H), 4.17 (dd, *J* = 12.5, 2.1 Hz, 1 H), 3.80 (dd, *J* = 9.8, 6.3 Hz, 1 H), 3.74-3.72 (m, 1 H), 3.65 (dd, *J* = 9.7, 5.9 Hz, 1 H), 3.50-3.46 (m, 1 H), 3.00-2.91 (m, 2 H), 2.86 (dd, *J* = 17.5, 9.2 Hz, 1 H), 2.71-2.65 (m, 1 H), 2.59 (dd, *J* = 17.5, 5.5 Hz, 1 H), 2.06 (s, 3 H), 2.04 (s, 3 H), 2.00 (s, 3 H), 1.97 (s, 3 H), 1.97-1.90 (m, 2 H), 1.77 (s, 3 H) ppm; ^{13}C NMR (126 MHz, Acetone-d_6) δ 207.51, 170.73, 170.46, 170.03, 169.05, 139.60, 139.57, 139.51, 133.50, 129.17, 129.16, 128.70, 128.60, 128.44, 128.34, 108.36, 83.93, 80.85, 80.76, 75.23, 74.80, 73.98, 73.83, 70.44, 69.47, 68.78, 67.67, 62.63, 46.53, 40.36, 30.70, 29.46, 20.70, 20.65, 20.61, 20.28, 20.20 ppm. MS (ESI): 810.2 (M+H); HRMS (ESI): Calculated for $C_{41}H_{52}O_{14}N_3$ (M+H): 81034438, Found: 810.34422. $[\alpha]_D^{20}$ = +14.4 (c = 0.43, CH_2Cl_2).

***N*-1’-(2’-deoxy-3’-(5-(2-*O*-(3,5-*O*-di-benzyl-2-*C*-acetylmethyl)-α-D-lyxofuranosyl)methyl)-1*H*-1,2,3-triazol-1-yl)-β-D-erythro-pentofuranosyl Thymine**

The title product compound is prepared using **Method G** and isolated by flash column chromatography (1:1 Pentane: Ethyl Acetate to pure Ethyl acetate) giving a pale yellow syrup (84.0 mg, 0.12 mmol, 62% yield, α/β ratio 88:12)

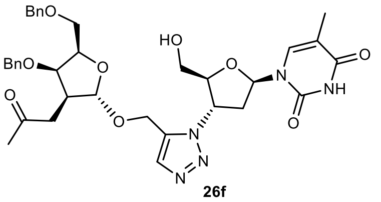

^{1}H NMR (500 MHz, Acetone-d_6) δ 10.13 (s, br, 1 H), 7.90 (d, *J* = 1.0 Hz, 1 H), 7.68 (s, 1 H), 7.39-7.26 (m, 10 H), 6.67 (t, *J* = 6.9 Hz, 1 H), 5.51-5.47 (m, 1 H), 4.96 (d, *J* = 4.3 Hz, 1 H), 4.89 (d, *J* = 13.0 Hz, 1 H), 4.72 (d, *J* = 12.8 Hz, 1 H), 4.60-4.53 (m, 3 H), 4.44-4.39 (m, 2 H), 4.36 (d, *J* = 11.3 Hz, 1 H), 4.33-4.30 (m, 1 H), 3.91-3.84 (m, 2 H), 3.81 (dd, *J* = 9.8, 6.0 Hz, 1 H), 3.67 (dd, *J* = 9.8, 6.0 Hz, 1 H), 2.88-2.80 (m, 3 H), 2.71-2.67 (m, 1 H), 2.61 (dd, *J* = 17.8, 5.7 Hz, 1 H), 2.02 (s, 3 H), 1.84 (d, *J* = 1.0 Hz, 3 H) ppm, (O***H*** could not be found); ^{13}C NMR (126 MHz, Acetone-d_6) δ 207.60, 164.36, 151.39, 139.48, 139.37, 137.36, 134.81, 134.43, 129.18, 129.16, 128.73, 128.62, 128.48, 128.37, 110.93, 108.04, 86.83, 86.27, 81.20, 80.72, 74.82, 73.84, 69.39, 62.70, 59.14, 58.26, 46.38, 40.14, 39.00, 30.07, 12.73 ppm. MS (ESI): 676.2 (M+H); HRMS (ESI): Calculated for $C_{35}H_{42}O_9N_5$ (M+H): 676.29770, Found: 676.29801. $[\alpha]_D^{20}$ = +30.8 (c = 0.64, CH_2Cl_2).

1-Deoxy-1-(5-(2-*O*-(3,5-*O*-di-benzyl-2-*C*-acetylmethyl)-α-D-lyxofuranosyl)ethyl)-1*H*-1,2,3-triazol-1-yl)-2,3,4,6-tetra-acetyl-α-D-glucopyranoside

The title product compound is prepared using **Method G** and isolated by flash column chromatography (3:1-1:1 Pentane: Ethyl Acetate) giving a pale yellow syrup (131.0 mg, 0.16 mmol, 82% yield, α/β ratio 95:5)

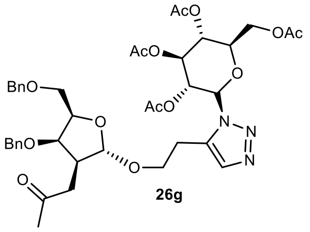

26g

^{1}H NMR (500 MHz, Acetone-d_6) δ 7.59 (s, 1 H), 7.38-7.27 (m, 10 H), 6.23 (d, *J* = 9.5 Hz, 1 H), 5.87 (t, *J* = 9.5 Hz, 1 H), 5.54 (t, *J* = 9.2 Hz, 1 H), 5.28 (t, *J* = 9.8 Hz, 1 H), 4.88 (d, *J* = 4.3 Hz, 1 H), 4.58-4.53 (m, 3 H), 4.37-4.25 (m, 5 H), 4.19 (dd, *J* = 12.4, 1.8 Hz, 1 H), 3.96-3.92 (m, 1 H), 3.82-3.73 (m, 2 H), 3.66 (dd, *J* = 9.8, 5.8 Hz, 1 H), 3.18-3.11 (m, 2 H), 2.83 (dd, *J* = 17.6, 9.1 Hz, 1 H), 2.70-2.67 (m, 1 H), 2.56 (dd, *J* = 17.7, 5.5 Hz, 1 H), 2.04 (d, *J* = 3.7 Hz, 6 H), 2.00 (s, 3 H), 1.97 (s, 3 H), 1.78 (s, 3 H) ppm; ^{13}C NMR (126 MHz, Acetone-d_6) δ 207.40, 170.74, 170.43, 170.01, 169.08, 139.54, 139.46, 137.39, 134.09, 129.16 (two carbons), 128.70, 128.58, 128.44, 128.33, 108.44, 84.27, 80.84, 80.76, 75.19, 74.79, 73.90, 73.81, 70.43, 69.34, 68.72, 66.73, 62.53, 46.49, 40.28, 30.07, 24.50, 20.68, 20.65, 20.60, 20.30 ppm. MS (ESI): 796.2 (M+H); HRMS (ESI): Calculated for $C_{40}H_{50}O_{14}N_3$ (M+H): 796.32873, Found: 796.32742. $[\alpha]_D^{20}$ = +8.6 (c = 1.25, CH_2Cl_2).

(4*aR*,6*S*,7*S*,7*aR*)-6-(6-*O*-1,2:3,4-di-*O*-isopropylidene-α-D-galactopyranosyl)-2-methyltetrahydro-4*H*-2,7-methanofuro[3,2-*d*][1,3]dioxine

To a solution of glycoside **20a** (122.0 g, 0.22 mmol) in CH_2Cl_2 (5 mL) was added 10 wt% Pd-C (63.0 mg, 0.06 mmol), and the resulting mixture was stirred overnight at room temperature under hydrogen (hydrogen balloon). The mixture was filtered through a pad of Celite, and the resulting filtrate was concentrated under reduced pressure. The crude product was purified by silica gel column chromatography (dry loading, using 9:1-3:1 Pentane/Ethyl acetate as eluent) to afford the title compound (71.0 mg) in 86% yield as a syrup.

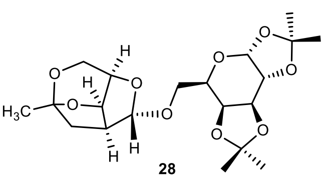

28

^{1}H NMR (700 MHz, Acetone-d_6) δ 5.45 (d, J = 5.1 Hz, 1 H), 5.04-5.01 (m, 2 H), 4.61 (dd, J = 8.0, 2.4 Hz, 1 H), 4.40 (d, J = 7.7 Hz, 1 H), 4.33 (dd, J = 5.1, 2.4 Hz, 1 H), 4.26 (dd, J = 7.9, 1.9 Hz, 1 H), 3.96 (td, J = 6.7, 1.9 Hz, 1 H), 3.63-3.59 (m, 2 H), 3.58 (dd, J = 11.2, 1.4 Hz, 1 H), 3.43 (dd, J = 11.5, 1.2 Hz, 1 H), 2.75 (ddd, J = 11.5, 6.6, 3.0 Hz, 1 H), 1.97-1.93 (m, 1 H), 1.71 (dd, J = 13.2, 3.1 Hz, 1 H), 1.47 (s, 3 H), 1.37 (s, 3 H), 1.36 (s, 3 H), 1.32 (s, 3 H), 1.31 (s, 3 H) ppm; ^{13}C NMR (176 MHz, Acetone-d_6) δ 109.58, 108.98, 107.67, 106.10, 97.28, 81.43, 78.02, 71.80, 71.60, 71.54, 66.97, 66.60, 61.91, 48.36, 42.03, 26.45, 26.39, 25.26, 24.78, 24.61 ppm. MS (ESI): 437.2 (M+Na); HRMS (ESI): Calculated for $C_{20}H_{30}O_9Na$ (M+Na): 437.17820, Found: 437.17830. $[\alpha]_D^{20}$ = -1.0 (c = 0.89, CH_2Cl_2).

***N*'-((*E*)-1-((2*S*,3*S*,4*R*,5*R*)-4-(benzyloxy)-5-((benzyloxy)methyl)-2-(((3*aR*,5*R*,5*aS*,8*aS*,8*bR*)-2,2,7,7-tetramethyltetrahydro-5*H*-bis([1,3]dioxolo)[4,5-b:4',5'-d]pyran-5-yl)methoxy)tetrahydrofuran-3-yl)propan-2-ylidene)-5-((3*aS*,4*S*,6*aR*)-2-oxohexahydro-1*H*-thieno[3,4-*d*]imidazol-4-yl)pentanehydrazide**

To a mixture of biotin hydrazide (38.8 mg, 0.15 mmol, 1.0 equiv.) in 2-propanol (1 mL) was added glycoside **20a** (91.9 mg, 0.15 mmol, 1.0 equiv.). The reaction mixture was stirred at 80 ℃ for 48 h. Then solvent was removed *in vacuo* and the residue was purified by column chromatography (dry loading, using 50:1-10:1 CH_2Cl_2/MeOH as eluent) to afford the title product (85.0 mg) in 66% yield as a white solid (Z/E isomer mixture, the ratio for E/Z is 7:1)

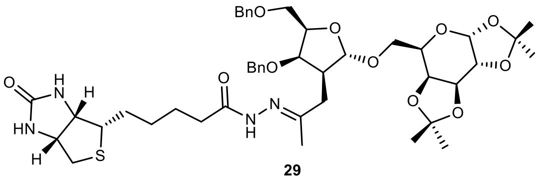

29

^{1}H NMR (500 MHz, CD_2Cl_2) δ 9.31 (s, 1 H), 7.34-7.21 (m, 10 H), 6.50 (s, 1 H), 5.46 (d, J = 5.1 Hz, 1 H), 5.18 (s, br, 1 H), 4.87 (d, J = 5.3 Hz, 1 H), 4.60-4.51 (m, 4 H), 4.45-4.39 (m, 2 H), 4.38-4.35 (m, 1 H), 4.29 (dd, J = 5.1, 2.4 Hz, 1 H), 4.27-4.23 (m, 1 H), 4.22-4.19 (m, 2 H), 4.00-3.95 (m, 1 H), 3.75-3.68 (m, 2 H), 3.66-3.62 (m, 1 H), 3.58 (dd, J = 10.5, 6.3 Hz, 1 H), 3.14-3.10 (m, 1 H), 2.87 (dd, J = 12.8, 4.9 Hz, 1 H), 2.69-2.60 (m, 3 H), 2.60-2.47 (m, 2 H), 2.41 (dd, J = 16.1, 5.6 Hz, 1 H), 1.81 (s, 3 H), 1.74-1.61 (m, 4 H), 1.49 (s, 3 H), 1.46-1.40 (m, 2 H), 1.39 (s, 3 H), 1.33-1.29 (m, 6 H) ppm; ^{13}C NMR (126 MHz, CD_2Cl_2) δ 176.34, 164.14, 151.54, 138.81, 138.79, 128.83, 128.82, 128.49, 128.37, 128.27, 128.11, 109.56, 109.00, 108.95, 96.85, 81.01, 80.23, 74.53, 73.91, 71.51, 71.17, 71.10, 69.00, 68.33, 66.77, 62.22, 60.48, 56.10, 47.80, 41.31, 35.07, 32.97, 29.04, 28.96, 26.34, 26.28, 25.37, 25.26, 24.77, 16.44 ppm. MS (ESI): 876.4 (M+Na); HRMS (ESI): Calculated for

$C_{44}H_{60}O_{11}N_4NaS$ (M+Na): 875.38715, Found: 875.38679. $[\alpha]_D^{20}$ = +26.4 (c = 0.25, CH_2Cl_2). M.P.: 148-149 ℃.

((1,1'-Biphenyl)-4-yl)methyl 3,5-*O*-di-benzyl-2-*C*-acetylmethyl-α-L-lyxofuranoside

A dry tube with a stirrer bar was charged with 2-dicyclohexylphosphino-2',4',6'-triisopropylbiphenyl (XPhos) (2.2 mg, 4.6 µmol) $Pd_2(dba)_3$ (0.75 mg, 1.3 µmol), Na_2CO_3 (9.8 mg, 0.09 mmol), Phenylboronic acid (11.3 mg, 0.09 mmol) and 4-Bromophenylmethyl 3,5-*O*-di-benzyl-2-*C*-acetylmethyl-α-L-xylofuranoside **20n** (25.0 mg, 0.05 mmol). The tube was purged with Argon and sealed with a rubber stopper. Then toluene (0.6 mL) and water (0.2 mL) was added. The reaction mixture was heated to 95 ℃ and stirred for 14 h. After cooling to room temperature, the reaction mixture was filtered and 15 mL CH_2Cl_2 15 mL brine were added. The layers were separated and the aqueous phase extracted twice with 10 mL CH_2Cl_2. The combined organic layers were dried over $MgSO_4$ and the solvent was concentrated under reduced pressure and purified by flash column chromatography (dry loading, using Pentane/Ethyl acetate (3:1 v/v) as eluent) to afford the coupling product (24.1 mg) in 96% yield as a pale yellow solid.

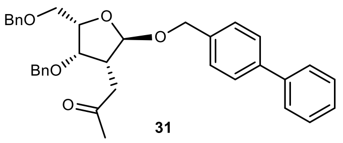

31

^{1}H NMR (700 MHz, Acetone-d$_6$) δ 7.65 (d, *J* = 7.2 Hz, 2 H), 7.62 (d, *J* = 7.7 Hz, 2 H), 7.46-7.44 (m, 4 H), 7.39 (d, *J* = 7.0 Hz, 2 H), 7.36-7.32 (m, 5 H), 7.29-7.27 (m, 4 H), 4.96 (d, *J* = 4.9 Hz, 1 H), 4.76 (d, *J* = 4.9 Hz, 1 H), 4.61-4.57 (m, 3 H), 4.35 (d, *J* = 11.9 Hz, 1 H), 4.42-4.40 (m, 1 H), 4.36 (d, *J* = 10.2 Hz, 1 H), 4.34-4.33 (m, 1 H), 3.84 (dd, *J* = 9.8, 6.3 Hz, 1 H), 3.69 (dd, *J* = 9.8, 5.9 Hz, 1 H), 2.86 (dd, *J* = 17.6, 5.1 Hz, 1 H), 2.78-2.75 (m, 1 H), 2.60 (dd, *J* = 17.6, 5.1 Hz, 1 H), 2.03 (s, 3 H) ppm; ^{13}C NMR (176 MHz, Acetone-d$_6$) δ 207.23, 141.68, 141.01, 139.68, 139.57, 138.75, 129.77, 129.19, 129.17, 129.12, 128.72, 128.61, 128.44, 128.34, 128.20, 127.75, 127.66, 107.85, 80.97, 80.94, 74.88, 73.89, 70.15, 69.53, 46.66, 40.38, 30.14 ppm. Gated ^{13}C NMR (176 MHz, Acetone-d$_6$) δ 107.85 (d, *J* = 169.4 Hz) ppm. MS (ESI): 559.2 (M+Na); HRMS (ESI): HRMS (ESI): Calculated for $C_{35}H_{36}O_5Na$ (M+Na): 559.24550, Found: 559.24479. $[\alpha]_D^{20}$ = -62.1 (c = 0.24, CH_2Cl_2).

2-Indolylmethyl 3,5-*O*-di-benzyl-2-*C*-acetylmethyl-α-D-lyxofuranoside

A 10 mL oven dried round flask charged with carbohydrate fused cyclopropane (70.5 mg, 0.2 mmol, 1.25 equiv.) was purged with argon then BArF hydrogen bonding catalyst **A** solution (100 µL, 4 mM, 0.2 mol%), propargyl alcohol **21u** (22.5 mg, 0.4 mmol, 2.5 equiv.) and anhydrous fluorobenzene (1.1 mL) was added. The flask was immersed and stirred in the preheated 50 ℃ oil bath for 16 h. When the reaction was completed. The solvent was removed under reduced pressure to afford a residue. To the residue, *N*-tosyl-*o*-iodoaniline (59.7 mg, 0.16 mmol, 1 equiv.), $[Cu(Phen)(PPh_3)_2]NO_3$ (10.5 mg, 10 mol%) was added and the flask was purged with argon again. Then toluene (4 mL) was added to form a mixture. After that, anhydrous K_3PO_4 (78.0 mg, 3 equiv.) was added under an argon atmosphere. The flask was sealed with a rubber septum and the reaction mixture was heated at 110 °C for 11 h. When the starting material *N*-tosyl-*o*-iodoaniline was consumed (Monitored by TLC), the reaction mixture was cooled to room temperature, and TBAF (1 M in THF, 7 equiv.) was added and the mixture was further heated at 110 °C for 3 h. The reaction was then diluted with ethyl acetate and washed with water. The aqueous layer was extracted with ethyl acetate, and the combined

organic layers were dried with anhydrous Na_2SO_4 and then concentrated and purified by flash column chromatography (dry loading, using Pentane/Ethyl acetate 8:1-3:1-1:2 (v/v) as eluent) to afford the title product (45 mg) in 56% yield as a syrup.

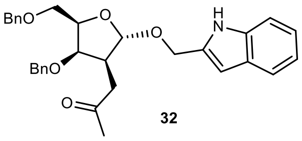

32

^{1}H NMR (500 MHz, Acetone-d_6) δ 10.12 (s, br, 1 H), 7.50 (d, *J* = 7.8 Hz, 1 H), 7.41-7.26 (m, 11 H), 7.05 (t, *J* = 7.2 Hz, 1 H), 6.98 (t, *J* = 7.5 Hz, 1 H), 6.38 (s, 1 H), 4.98 (d, *J* = 4.4 Hz, 1 H), 4.82 (d, *J* = 12.8 Hz, 1 H), 4.69 (d, *J* = 12.7 Hz, 1 H), 4.64-4.57 (m, 2 H), 4.54 (d, *J* = 11.3 Hz, 1 H), 4.41-4.38 (m, 1 H), 4.33 (d, *J* = 11.1 Hz, 1 H), 4.30 (dd, *J* = 5.5, 4.0 Hz, 1 H), 3.83 (dd, *J* = 9.8, 6.0 Hz, 1 H), 3.71 (dd, *J* = 9.7, 6.3 Hz, 1 H), 2.84 (dd, *J* = 17.7, 9.8 Hz, 1 H), 2.73-2.67 (m, 1 H), 2.57 (dd, *J* = 17.7, 4.7 Hz, 1 H), 2.01 (s, 3 H) ppm; ^{13}C NMR (126 MHz, Acetone-d_6) δ 207.42, 139.55, 139.46, 137.76, 136.70, 136.56, 129.21, 129.18, 128.73 (two carbons), 128.46, 128.42, 122.24, 121.01, 119.99, 111.94, 107.85, 101.82, 81.04, 80.89, 74.86, 73.95, 69.56, 64.18, 46.65, 40.25, 30.02 ppm. MS (ESI): 522.0 (M+Na); HRMS (ESI): Calculated for $C_{31}H_{33}O_5NNa$ (M+Na): 522.22509, Found: 522.22426. $[\alpha]_D^{20}$ = +51.3 (c = 0.46, CH_2Cl_2).

Supplementary Discussions

Supplementary Discussion 1: Plausible mechanism for the formation of bicyclic 20k and 23e (see supplementary figure 3)
We propose the following explanation for the observed reactivity differences towards *O*-glycosylation when a bulky hard *O*-acceptor is used to react with either Xylose-derived donors (**A**) or Arabinose/glucose-derived donors (**B**, **C**).

(1) Case for a Sterically Hindered Hard Nu on Xylose derived donors (**A**)
In this instance, the C2 ketone group will form a bicyclic intermediate through anchimeric assistance. The C2 neighboring group effect directs the nucleophilic attack of the *O*-acceptor from the rear face.
In addition, the C5 OBn group also blocks the top face, hence, C2 neighboring group effect and the steric effect from the C5 group cooperatively directs the attack of the acceptor from the rear face. And because the position **a** is more reactive than **b** due to the buildup of positive formal charge on oxygen, the reaction occurs preferentially at position **a**.

(2) Case for a Sterically Hindered Hard Nu on Arabinose/glucose derived acceptors (**B**, **C**)
In this case, the C2 ketone group will also form the bicyclic intermediate through achimeric assistance. While the C2 neighboring group effect directs the nucleophilic attack of the acceptor from the top face, the proximal C5 group also blocks the top face co-currently, forming a *mis-matched* type scenario.
Given the steric dilemma posed to the bulky *O*-acceptor at position **a (pink arrow)**, easing steric congestion by attacking the distal position **b (green arrow)** might possibly be an energetically more favorable pathway to form **20k** or **23e**.

Supplementary Disscussion 2: control experiments and counteranion effect (see supplementary table 3)
In an effort to better understand the mode of catalytic activation, we performed a series of control experiments with various known hydrogen bonding catalysts ranging from thiourea, squaramide and urea. Since H-bonds are directional, in entries 1-3, different thioureas bearing different hydrogen bonding alignments were tested. Comparing entry 1 and 3, a bidentate activation mode by the Schreiner's catalyst **D** is superior to a mono dentate **G** when one N-H group is blocked by a methyl group. A cyclic thiourea catalyst **H** (2-Thiouracil) which distorts the bidentate binding capability also gave 83% yield. This shows that thiourea can catalyze this reaction regardless of a mono dentate or bidentate activation mode.[12]

We then employed a H-bonding squaramide catalyst **E** having a similar pK_a value as the Schreiner's thiourea **D** (Entry 4). The difference between entry 1 and entry 4 is the spatial distance between the 2 N-H groups. This change in H-bonding distance disturbs the reaction and results in only 2% yield. The usage of urea **C** gave negligible yield, showing the cruciality of the thiourea moiety.

Using McGarrigle and Galan et al. reported control experiments,[13] we tested $Et_3N{\bullet}HCl$ and Meldrum acid **I**, both having acidity and pK_a of a slightly higher and lower value in comparison

with **D** respectively. In comparison to **D**, in both cases, reactions were inferior to thiourea catalysis.[15]

In order to get more insight into how the counteranion affect the catalysis of catalyst **A**, we synthesized and executed the reactions with compound **J** and **K**, bearing the iodide and triflate counteranions respectively. Due to its insolubility, **J** could not catalyze the glycosylation. Triflate **K** catalyzed the same reaction with rather high yields and anomeric selectivity, albeit with slightly inferior yield compared to catalyst **A**.

Supplementary Notes

Supplementary Note 1: Blank reaction

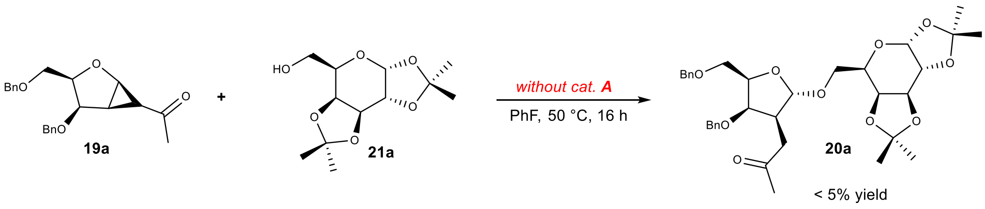

We noticed that traces of impurities present in the synthesis of **19a**, ***ent*-19a** and **19b** have significant effects on the reproducibility of the methodology. Control experiments were meticulously conducted on every batch of strained cyclopropane donor to ensure that background reaction in the absence of catalyst gives less than 5% NMR yield, to confirm the batch quality.
If background reaction gives more than 5% NMR yield, the batch should be repurified till less than 5% yield is observed when the strained cyclopropane donor is subjected to the catalytic conditions without any catalyst.

Supplementary Note 2: Reaction monitoring at 50°C (see supplementary table 5, supplementary figure 7 and 9) Intermediate **27a** could not be observed when the reaction monitoring was performed at 50 °C possibly due to an even shorter lifetime under elevated temperatures.

Supplementary Note 3: Comparison of the chemical shift of ^{13}C with the known compound **(+)-No. 2106**[16] are as follows. The data for **26** is displayed in the front, the data for **(+)-No. 2106** is listed in the back. C**1**: 78.0 ppm, 77.2 ppm; C**2**: 61.9 ppm, 61.3 ppm; C**3**: 24.6 ppm, 22.3 ppm; C**4**: 107.7 ppm, 106.3 ppm.

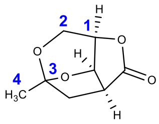

(+)-No.2016

Supplementary Note 4: *C*-glycosylation. For the C-glycosylation reactions, mesitylene, 1,3-dimethylbenzene, 3-cyanocoumarin and anthracene had been tried. All nucleophiles did not present any reactivity.

Supplementary References

1. Nagy, V., Agócs, A., Turcsi, E. & Deli, J. Isolation and purification of acid-labile carotenoid 5,6-epoxides on modified silica. *Phytochemical Analysis* **20**, 143-148 (2009).

2. Ma, X., Tang, Q., Ke, J., Zhang, J., Wang, C., Wang, H., Li, Y. & Shao, H. Straightforward and highly diastereoselective synthesis of 2,2-di-substituted perhydrofuro[2,3-b]pyran (and furan) derivatives promoted by $BiCl_3$. *Chem. Commun*. **49**, 7085-7087 (2013).

3. Tian, Q., Dong, L., Ma, X., Xu, L., Hu, C., Zou, W. & Shao, H. Stereoselective synthesis of 2-*C*-branched (acetylmethyl)oligosaccharides and glycoconjugates: Lewis acid-catalyzed glycosylation from 1,2-cyclopropaneacetylated sugars. *J. Org. Chem*. **76**, 1045-1053 (2011).

4. Shen, X., Xia, J., Liang, P., Ma, X., Jiao, W. & Shao, H. $Zn(OTf)_2$ promoted rearrangement of 1,2-cyclopropanated sugars with amines: a convenient method for the synthesis of 3-polyhydroxyalkyl-substituted pyrrole derivatives. *Org. Biomol. Chem*. **13**, 10865-10873 (2015).

5. Wang, C., Ma, X., Zhang, J., Tang, Q., Jiao, W. & Shao, H. Methanesulfonic-acid-catalysed ring opening and glycosylation of 1,2-(acetylcyclopropane)-annulated D-lyxofuranose. *Eur. J. Org. Chem*. 4592-4599 (2014).

6. Yakelis, N. A. & Bergman, R. G. Safe preparation and purification of sodium tetrakis[(3,5-trifluoromethyl)phenyl]borate ($NaBArF_{24}$): reliable and sensitive analysis of water in solutions of fluorinated tetraarylborates. *Orgnometallics* **24**, 3579-3581 (2005).

7. Fan, Y. & Kass, S. R. Electrostatically enhanced thioureas *Org. Lett*. **18**, 188-191 (2016).

8. Li, X., Deng, H., Luo, S. & Cheng, J. Organocatalytic three-component reactions of pyruvate, aldehyde and aniline by hydrogen-bonding catalysts. *Eur. J. Org. Chem.* 4350-4356 (2008).

9. Data from Bordwell pKa Table. https://www.chem.wisc.edu/areas/reich/pkatable/

10. Kryachko, E., Nguyen, M. T. & Zeegers-Huyskens, T. Thiouracils: acidity, basicity, and interaction with water. *J. Phys. Chem. A* **105**, 3379-3387 (2001).

11. Zabka, M. & Sebesta, R. Experimental and theoretical studies in hydrogen-bonding organocatalysis. *Molecules* **20**, 15500-15524 (2015).

12. Supady, A., Hecht, S. & Baldauf, C. About underappreciated yet active conformations of thiourea organocatalysts. *Org. Lett*. **19**, 4199-4202 (2017).

13. Balmond, E. I., Coe, D. M., Galan, M. C. & McGarrigle, E. M. α-Selective organocatalytic synthesis of 2-deoxygalactosides. *Angew. Chem. Int. Ed*. **51**, 9152-9155 (2012).

14. Kolthoff, I. M., Chantooni Jr, M. K. & Bhowmik, S. Dissociation constants of uncharged and monovalent cation acids in dimethyl sulfoxide. *J. Am. Chem. Soc.* **90**, 23-28 (1968).

15. Madarász, Á., Dósa, Z., Varga, S., Soós, T., Csámpai, A. & Pápai, I. Thiourea derivatives as brønsted acid organocatalysts. *ACS Catal*. **6**, 4379-4387 (2016).

16. Gréaud, S., Lusseau, J. & Landais, Y. Acyl radical addition to activated olefins: a stereocontrolled route to polysubstituted tetrahydrofurans and lactones, and application to the total synthesis of (+)-No. 2016 A. *Eur. J. Org. Chem.* 1323-1330 (2017).
